# Bis-Cycloruthenated Complexes in Visible Light-Induced C–H Alkylation with Epoxides

**DOI:** 10.1021/jacs.4c14835

**Published:** 2025-02-04

**Authors:** Kurt Bentley, Mishra Deepak Hareram, Gang-Wei Wang, Alexander A. V. Millman, Ignacio Perez-Ortega, Luke M. Nichols, Cassandre C. Bories, Lauren E. Walker, Adam W. Woodward, Alexander P. Golovanov, Louise S. Natrajan, Igor Larrosa

**Affiliations:** † Department of Chemistry, School of Natural Science, University of Manchester, Oxford Road, Manchester M13 9PL, U.K.; ‡ State Key Laboratory of Applied Organic Chemistry & College of Chemistry and Chemical Engineering, Lanzhou University, Lanzhou 730000, China

## Abstract

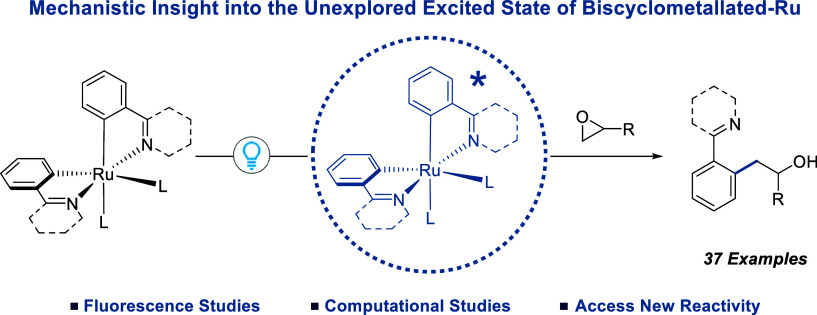

Bis-cycloruthenated complexes (BCRCs) of the type [Ru(N^C)_2_L_2_] are proposed to be key reactive intermediates in the Ru(II)-catalyzed directed C–H functionalization of arenes. While the exceptional ground state reactivity of BCRCs toward a number of electrophiles has been explored, their reactivity upon photoexcitation is still unknown. Herein, we report studies on the photoexcitation of BCRCs that establish their capability to access chemically useful excited states. Remarkably, photoexcited BCRCs demonstrate greatly increased reactivity toward the electron transfer processes required for alkyl halide activation, overcoming current limitations of their ground-state reactivity. We have demonstrated this reactivity by expanding upon the current chemical space occupied by Ru-catalyzed C–H functionalization to include *ortho*-alkylation with epoxides.

## Introduction

The convergence of organometallic catalysis and photocatalysis, commonly referred to as metallaphotocatalysis, represents a burgeoning field of immense scientific interest, captivating both chemical academic and industrial researchers alike.^1−3^ Light-induced processes play a crucial role in facilitating or triggering elementary steps within the catalytic cycle, through photosensitization or direct light absorption by organometallic complexes.^4,5^ These methods of light-induced activation offer new avenues for exploring asymmetric catalysis,^6−8^ energy transfer (EnT),^9,10^ and single electron transfer (SET) reactions.^11,12^ Traditional reactivity of transition metal catalysts can also be redirected through visible light excitation to significantly more reactive species, enabling access to previously unachievable chemistry. In some cases, a combination of photocatalytic and nonphotocatalytic steps can be performed by the same transition metal in so-called dual-function catalysis.^13^

**Figure 1:**
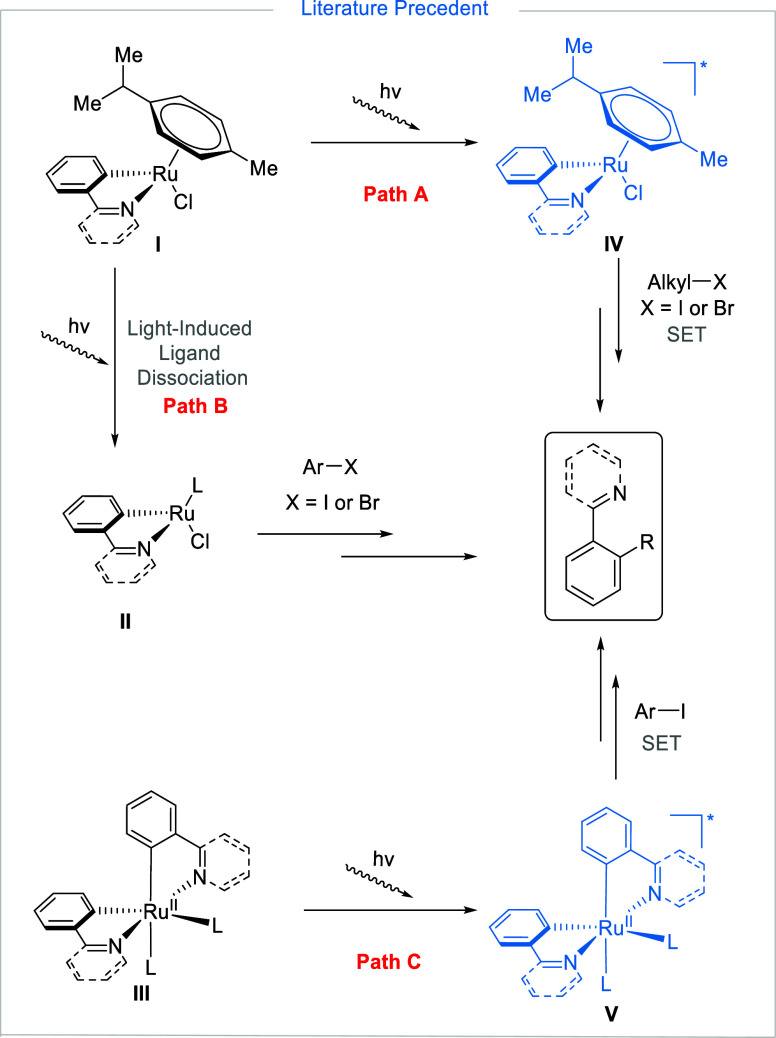
Various mechanistic pathways have been proposed to explain experimental observations of visible light-induced ruthenium-catalyzed *ortho*-functionalization.

While the use of Ru as a photocatalyst in the form of Ru(bpy)_3_ salts and their derivatives is well-known,^14^ the potential use of Ru species as dual-function catalysts, mediating C–H functionalization, and photoexcitation steps has only emerged in the past few years. Initial reports on the topic of photocatalyzed C–H alkylation using *p*-cymene-containing Ru catalysts proposed that monocyclometalated species (**I**) undergo photoexcitation by visible light to **IV**, followed by SET to alkyl halides (Figure 1, Path A).^15,16^ In follow-up Ru-catalyzed C–H arylations, it was instead proposed that the role of the visible light activation may be in facilitating the removal of the *p*-cymene ligand in **I** to form the “true” active catalytic species (**II**) which would then react without further light activation (Figure 1, Path B).^17,18^ Subsequently, similar photocatalyzed C–H functionalization processes were instead proposed to proceed through bis-cyclometalated Ru species (**III**) undergoing photoexcitation to **V**, followed by SET to aryl or benzyl halides (Figure 1, Path C).^19,20^ However, no direct experimental evidence for the existence of this photoexcitation step or the involvement of BCRC **III** in the reaction was provided. In addition, we have previously reported that BCRCs of the type **III** directly react thermally with Ar–I at room temperature.^21^ Catalytically, similar levels of reactivity at room temperature or near room temperature have been shown in the absence of photoexcitation, by simply using a *p*-cymene-free monocyclometalated Ru catalyst (RuBnN),^22,23^ and more recently using the dicationic complex [(*^t^*BuCN)_5_Ru(H_2_O)](BF_4_)_2_ (RuAqua).^24^ This casts into doubt the proposed role of visible light activation in paths A and C (Figure 1).

Herein, we report organometallic and catalytic studies that prove that bis-cyclometalated species can undergo photoexcitation and that these excited states can be harnessed to improve the scope of electrophiles that can be used in Ru-catalyzed C–H functionalization to include previously unreactive alkyl epoxides.

## Results and Disscussion

**Figure 2:**
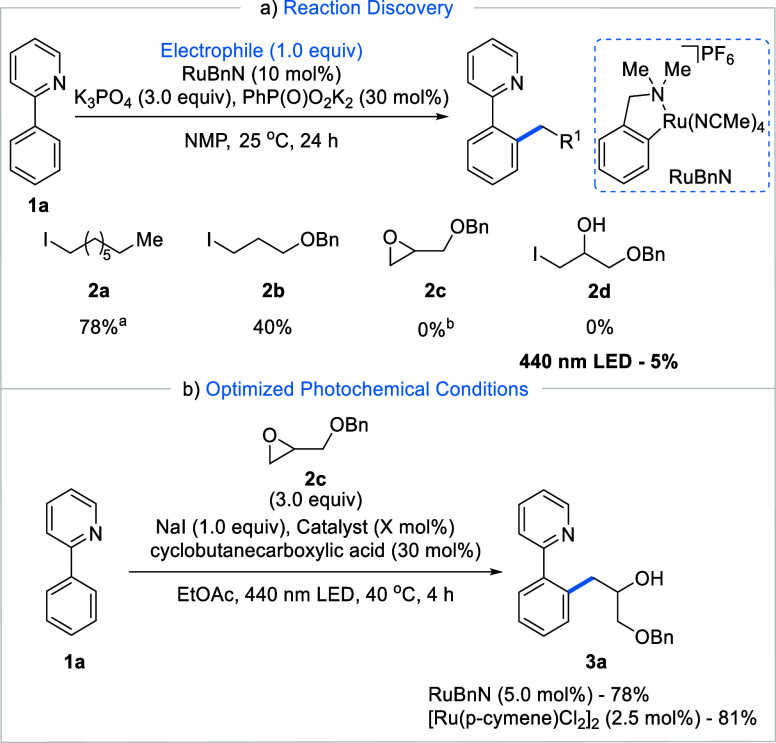
(a) Optimization process. (b) Optimized conditions for the photochemical Ru-catalyzed *ortho* C–H activation with epoxides. ^a^See our previous report on Ru-catalyzed alkylations.^23^^b^With 2-ethylbutanoic acid (30 mol %) and NaI (1.0 equiv).

We have recently reported a Ru-catalyzed *meta*-C–H alkylation with benzylic epoxides, in the presence of iodide salts.^25^ Our mechanistic studies indicated that in situ formed secondary benzyl iodide species, resulting from epoxide ring opening by iodide, are the active electrophilic species. We speculated that by applying the same concept to aliphatic epoxides, we would achieve *ortho*-alkylation, a process only known in palladium catalysis.^26−28^ We have previously demonstrated C–H alkylation with primary alkyl halides such as **2a** and **2b** to proceed at room temperature in NMP with the RuBnN catalyst (Figure 2a).^23^ Therefore, we were surprised to observe that the application of a range of conditions similar to those for the alkylation of **1a** with epoxide **2c** and NaI led to no reactivity. Similarly, the use of the putative electrophile formed upon epoxide opening by iodide, alkyl iodide **2d**, resulted in no product formation. To our delight, visible light irradiation of this reaction system led to the formation of product **3a** in trace quantities (5%). Further optimization revealed a robust protocol for the photocatalyzed *N*-directed *ortho*-alkylation of **1a** with epoxide **2c**, affording **3a** in 78% yield after only 4 h (Figure 2b, see the SI for optimization details) and replacing NMP with the greener solvent EtOAc. Equivalent results were obtained when air-stable [Ru(*p*-cymene)Cl_2_]_2_ was used as the catalyst instead of RuBnN.

**Figure 3:**
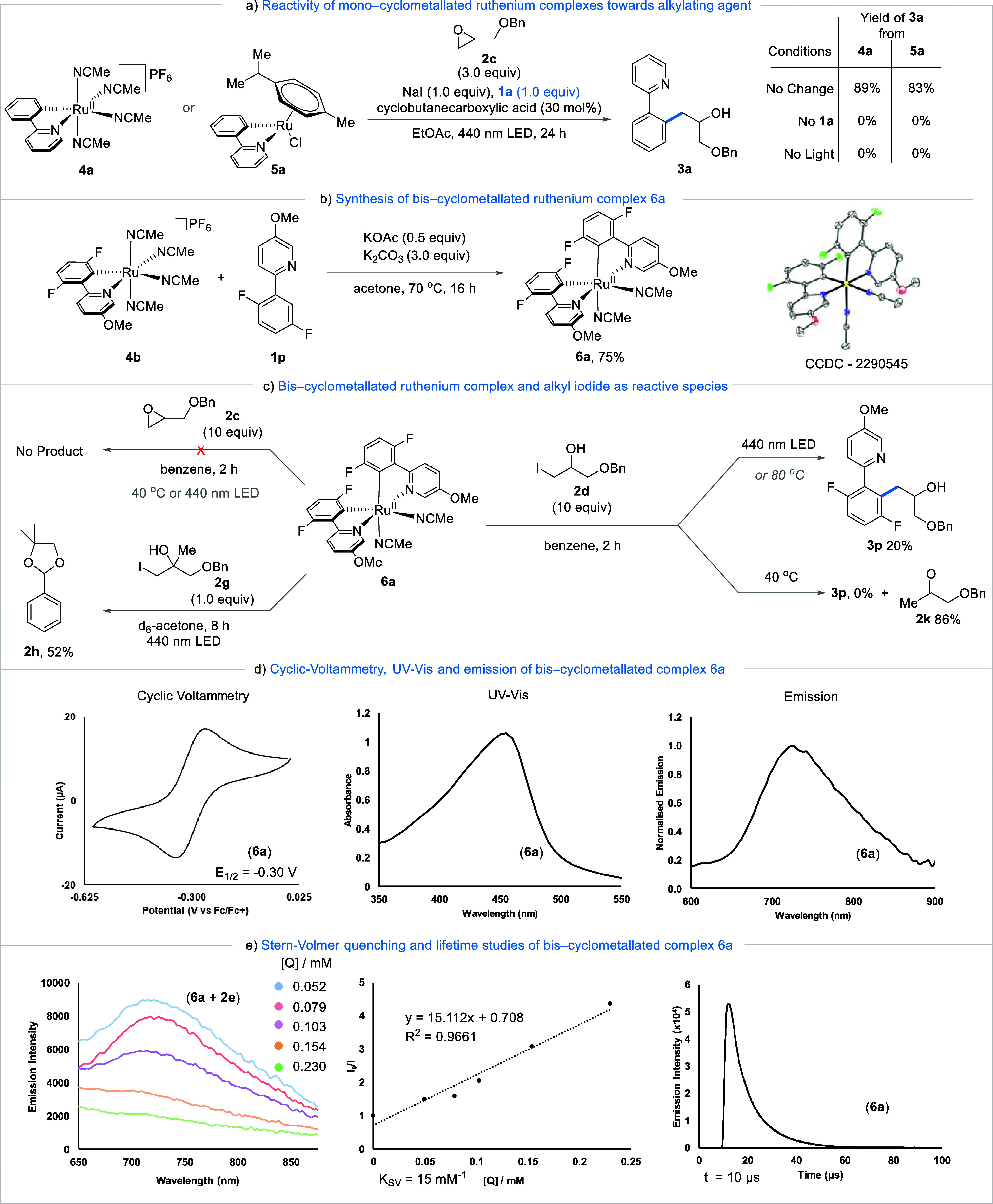
(a) Stoichiometric reactions with the monocyclometalated Ru(II) complexes **4a** and **5a**. (b) Synthesis of bis-cyclometalated Ru(II) complex **6a**. (c) Stoichiometric reaction of bis-cyclometalated Ru(II) complex **6a**, performed on a 0.018 mmol scale. (d) UV–Vis of complex **6a** and cyclic voltammetry of reactants. (e) Stern–Volmer quenching studies of complex **6a** with **2e** and a typical lifetime kinetic trace of **6a**. All fitted kinetic traces are given in the SI.

Given that simple photo-induced *p*-cymene-removal cannot be invoked to explain the role of visible light in switching on the reaction of **1a** with **2c** when using RuBnN as a catalyst, we hypothesized that photoexcitation of catalytic intermediates followed by reaction with alkyl iodide **2d** was taking place. In the first instance, we assessed the reactivity of the monocyclometalated cationic Ru species **4a** (Figure 3a). When 1 equiv of complex **4a** was reacted under the standard reaction conditions but in the absence of **1a**, the desired product **3a** was not observed, even after 24 h of irradiation. Similarly, the *p*-cymene-containing complex **5a** failed to lead to any product **3a**. These experiments indicate that the photoactivation of a monocyclometalated Ru complex (path A in Figure 1) is not in operation. Instead, when the reactions were repeated in the presence of 1 equiv of 2-phenylpyridine **1a**, product **3a** was formed in 89 and 83% yields starting from **4a** and **5a**, respectively. Importantly, under these conditions, light irradiation was still essential for the formation of **3a**, with no product detected in its absence (see SI for additional stoichiometric experiments with **4a**). We have previously shown that the addition of 1 equiv of **1a** to **4a** leads to the in situ formation of bis-cyclometalated Ru species.^21^ Thus, these results are suggestive of a photoexcitation of bis-cyclometalated Ru species as key to the reaction with **2c**. Due to the highly reactive nature of bis-cyclometalated Ru species, their chemical and physical properties are not well studied. To address this, we synthesized BCRC **6a** from the corresponding monocyclometalated Ru(II) complex **4b** (Figure 3b) and probed its reactivity toward electrophilic species **2c** and **2d**. No reaction was observed when complex **6a** was mixed with the epoxide **2c** at 40 °C or under light irradiation. Instead, the reaction of **6a** with alkyl iodide **2d**, resulting from iodide ring opening of epoxide **2c**, under light irradiation led to the formation of the desired product **3p** (Figure 3c). Complex **6a** also led to product formation by reaction with epoxide **2c** when NaI and the carboxylic acid additives were added (see Supporting Information). However, no product was formed when light irradiation was replaced with heating at 40 °C, although product formation could be observed at significantly higher temperatures of 80 °C. Instead, we observed HI elimination to form ketone **2k**, monocyclometalated **4b,** and free **1p**. Finally, when reacting **6a** with an alkyl iodide that cannot eliminate (**2g**), we obtained cyclization product **2h** indicative of radical formation followed by 1,5-HAT. Taken together, these experiments are strongly supportive of a photoexcitation of catalytic intermediate **6a** which then undergoes an electron transfer with in situ generated alkyl iodide **2d**, consistent with path C (Figure 1).

To further our understanding of the electron transfer process taking place between BCRC **6a** and alkyl iodide, we conducted cyclic voltammetry measurements (Figure 3d). BCRC **6a** showed a reversible redox event at −0.30 V versus Fc/Fc^+^. This event can be attributed to the Ru(II) and Ru(III) redox couple. This species is significantly more reducing than the corresponding monocyclometalated Ru(II) complex **4b**, for which the Ru(II/III) reversible redox event was observed at +0.53 V versus Fc/Fc^+^ (see SI). While epoxide **2c** did not show any redox event up to −2.0 V versus Fc/Fc^+^, the alkyl iodide **2d** showed an irreversible reduction at −1.6 V versus Fc/Fc^+^ (see SI for cyclic voltammetry spectra), indicating that **6a** in its ground state cannot directly reduce **2d**. To test our hypothesis further, we carried out several photo- and electrochemical experiments. The UV–Vis absorption spectrum of BCRC **6a** showed a λ_max_ of 455 nm (Figure 3d). Irradiation at 460 nm leads to an emission at 720 nm (see SI for further fluorescence experiments). On the basis of these spectroscopic and electrochemical measurements, we applied the Rhem–Weller formalism^29,30^ to estimate the redox potential of the BCRC **6a** in the excited state (E*(Ru(II)*/Ru(III)) = −2.21 V (vs Fc/Fc^+^)). This large redox potential suggests that the photoexcited BCRC **6a** is a capable SET reductant strong enough to reduce alkyl iodide **2d** (*E*_p/2_ = −1.6 vs −2.21 V (vs Fc/Fc^+^)). Further evidence for the proposed photoexcited process was obtained by examining the quenching of the emissions of **6a** with alkyl iodide **2e** (Figure 3e), with a Stern–Volmer plot revealing a linear relationship with a *K*_SV_ value of 15 mM^–1^. We also found no change in the absorption spectrum while quenching the emission. This linear relationship and lack of change within the absorption spectra indicate that the BCRC is undergoing dynamic quenching with the alkyl iodide (**2e**). Furthermore, we measured the photoexcited state lifetime of **6a** at 77 K revealing a relatively long lifetime of 10 μs when the complex was excited at 430 nm. On the other hand, no lifetime could be recorded at room temperature, due to weak emission and/or too short lifetime.

**Figure 4:**
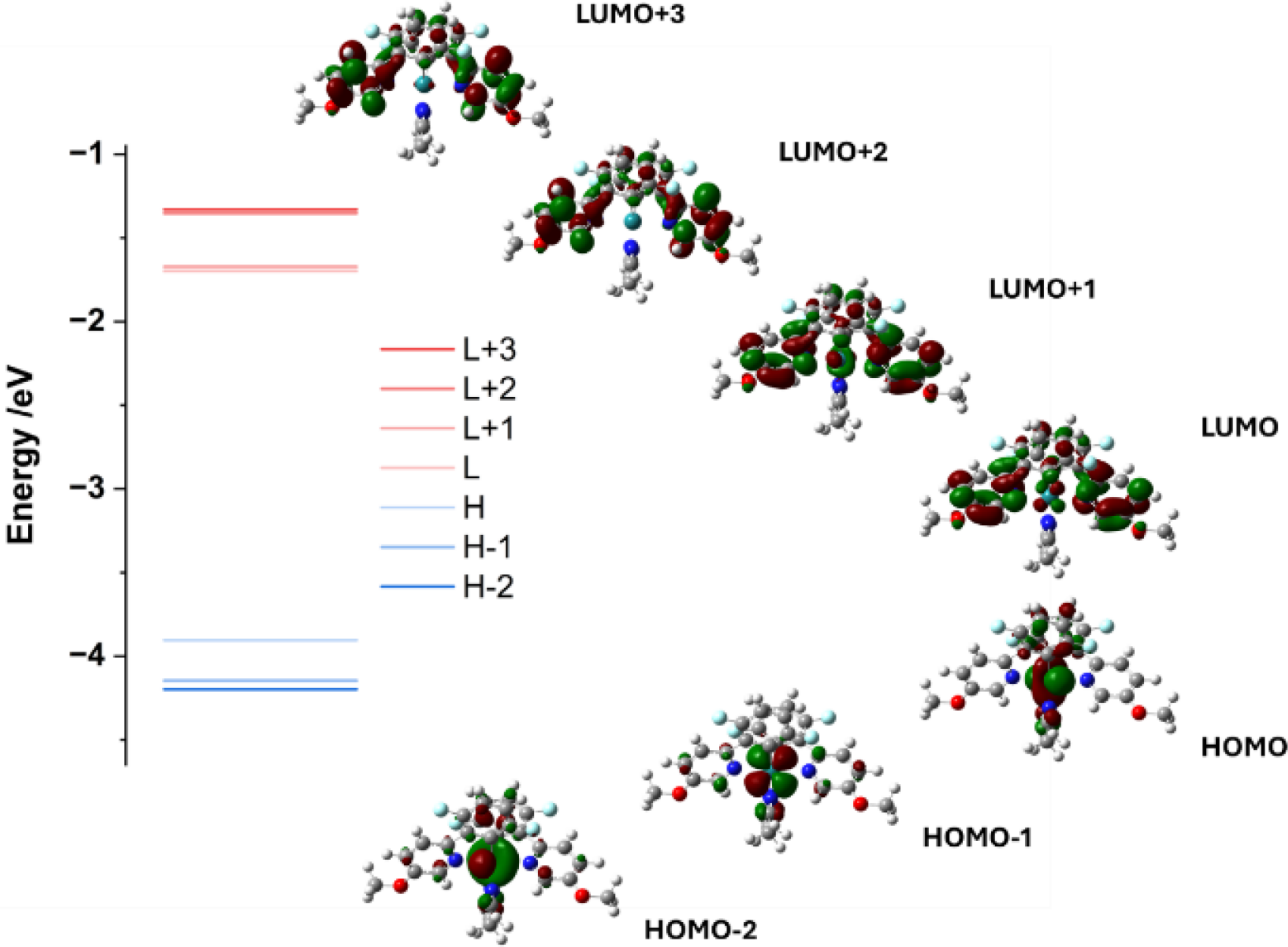
Frontier molecular orbitals overlaid on the optimized structure of Ru complex **6a** (isovalue = 0.035) and a plot of their relative energies (H = HOMO, highest occupied molecular orbital; L = LUMO, lowest unoccupied molecular orbital).

In order to explore the nature of the photoexcitation processes, we performed TDDFT calculations with the M06L method and incorporating an approximation of EtOAc as solvent. The calculations showed good agreement with the experimentally observed bond angles in the single crystal data and absorption spectra of **6a**. Additionally, comparison of the calculated HOMO/LUMO energy gap and the experimentally observed one shows good agreement. Analysis of the frontier molecular orbitals involved in the MLCT transitions illustrates a distinctive shift of electron density from the metal center to the phenylpyridine ligands (Figure 4).^31^ The CN triple bond of the two bound acetonitriles also appears as a small donor source (see SI for full transition contribution).

**Figure 5:**
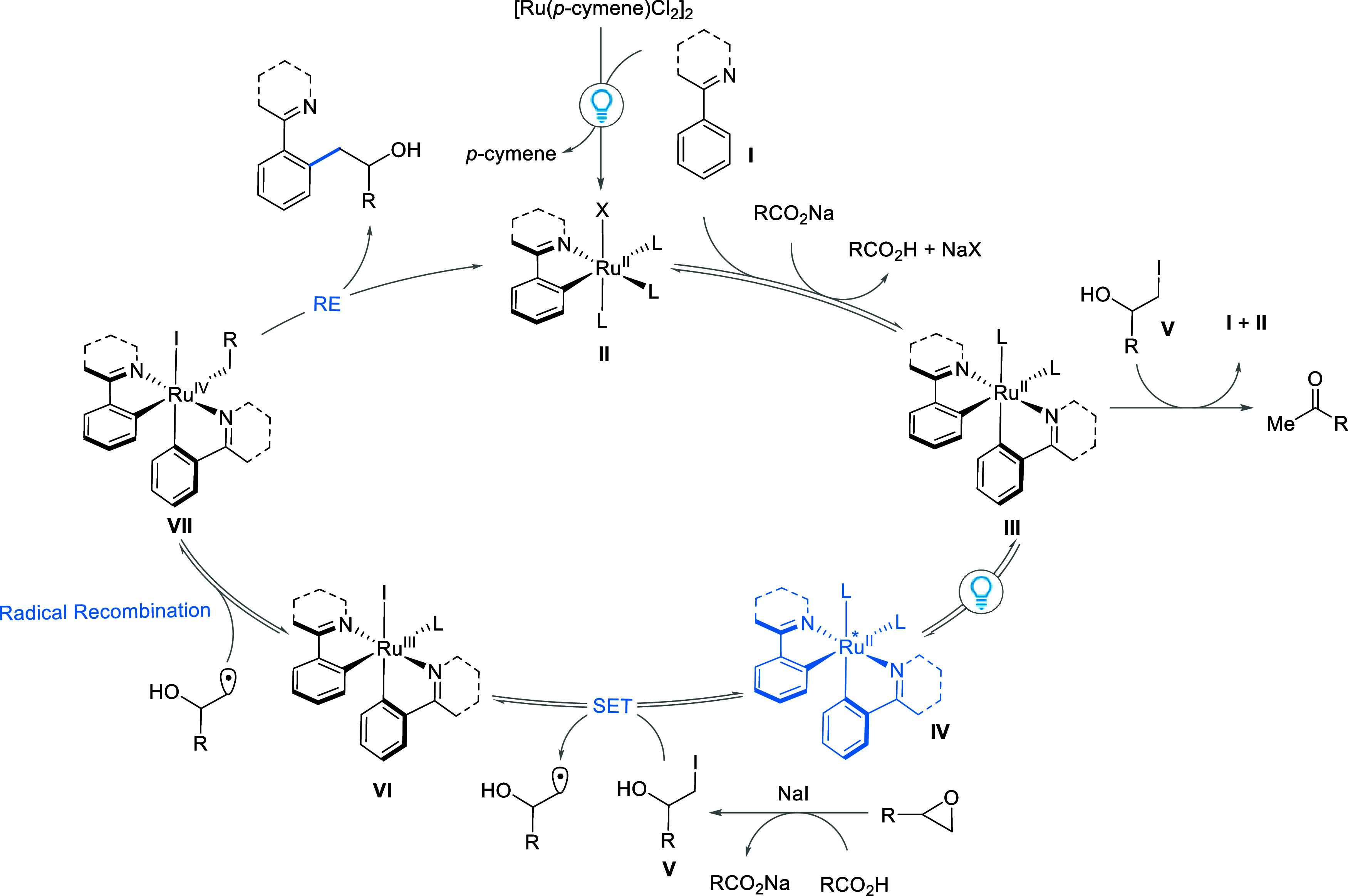
Proposed reaction mechanism (X = Cl or I).

Based on our experimental observations, we propose a catalytic cycle (Figure 5) starting with the initial light-assisted activation and formation of a monocyclometalated Ru(II) complex **II** via concerted metalation deprotonation (CMD) or base-assisted internal electrophilic substitution (BIES). A second CMD or BIES affords the key reactive bis-cyclometalated Ru(II) complex **III** which upon irradiation with light forms the highly reducing excited state complex **IV**. This excited state BCRC undergoes an outer sphere electron transfer, as evident by static versus dynamic quenching experiment (see SI), with alkyl iodide **V** to form an iodide bound Ru(III) species **VI**. Upon radical recombination with the alkyl radical, **VI** is converted into a high valent Ru(IV) species **VII** which undergoes reductive elimination to release the product and regenerate **II**. Under thermal conditions, an elimination pathway from BCRC species **III** is significantly more accessible, resulting in a rapid elimination with the Ru–C bond acting as a base in a concerted 6-membered transition state (see SI). When compared with simple alkyl iodides, we speculate that the presence of the hydroxyl group leads to more facile elimination. Under photochemical conditions, SET occurs from an excited state BCRC and becomes accessible and outcompetes thermal elimination. These observations were supported by in situ NMR measurements under light irradiation using the NMR Torch (see SI).^32^

**Figure 6:**
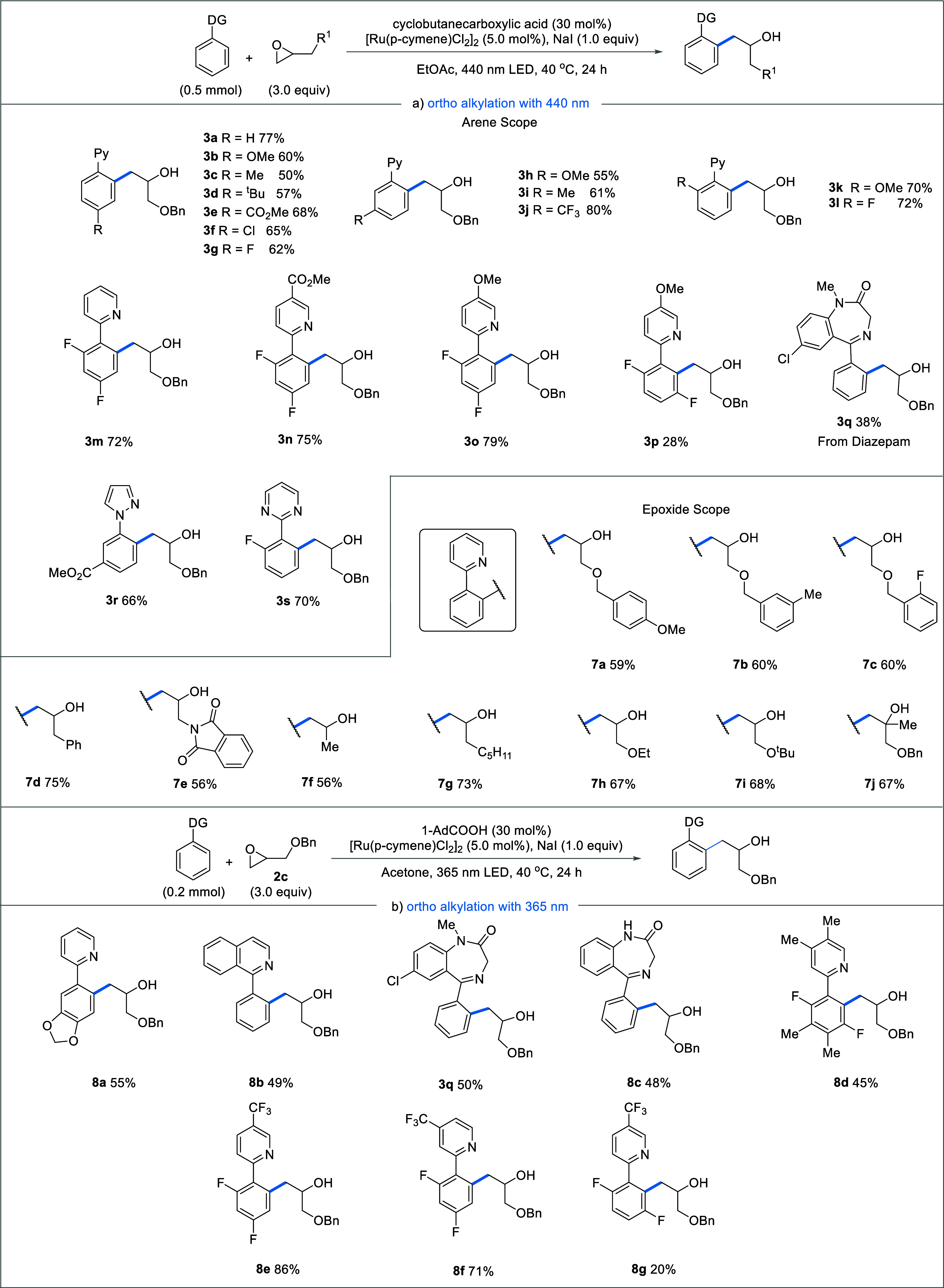
Reaction substrate scope. Yields reported are those of the isolated alkylated product.

With a comprehensive understanding of the reaction mechanism, a full scope of the light-induced Ru(II) catalyzed *ortho*-alkylation of directing group containing arenes was explored. From the outset, it was found that a diverse range of functional groups were well tolerated in the reaction (Figure 6a). Within the aryl unit of the 2-phenylpyridines, substitution at the *para* position with electron-donating groups OMe, Me, and tBu (**3b–3d**) resulted in 60, 50, and 57% yields, respectively. Substitution with electron-withdrawing groups at the *para* position also yielded good recovery of the *ortho* alkylated products **3e–3g**. Similarly, both electron-donating and electron-withdrawing substitution at the *meta* position within the aryl unit were well tolerated. However, we observed higher yields (80%) with the electron-withdrawing CF_3_ group at *meta* position **3j** than the electron-donating groups **3h** and **3i** with the respective yields of 55 and 61%. *ortho*-Substitution with OMe (**3k**) and F (**3l**) was also compatible, yielding 70 and 72% of the corresponding products, respectively. A double substitution within the aryl ring yielded 72% of the alkylated product **3m**, demonstrating that the reaction can tolerate multiple substitutions. Moreover, electron-withdrawing **3n** and electron-donating **3o** functionality directly attached to the pyridine ring gave good yields of the corresponding products. However, F-substitution next to the reactive C–H bond resulted in considerably lower yields (**3p**). Varying the directing group to diazepam **3q**, pyrazole **3r**, and pyrimidine **3s** showed good tolerance yielding 38, 66, and 70%, respectively.

Variations within the epoxide coupling partner were also explored. Aryls with both electron-donating (**7a**) and electron-withdrawing (**7c**) functional groups in the para position were tolerated with yields of 59 and 60%, respectively. A phthalimide substituent (**7e**) also produced the desired product in a yield of 56%. Finally, the reaction led to good yields with a variety of alkyl-substituted epoxides, with a methyl (**7f**) and an octyl (**7g**) substituted epoxide leading to the corresponding products in a yield of 56 and 73%, respectively. At several instances during our substrate scope, we observed arene substrates that either did not work at all or were poor yielding when the reaction was carried out under irradiation of 440 nm light. We reasoned that this lower reactivity could arise from variations in the energy for the excitation of the corresponding BCRC. Indeed, after further screening, we found that irradiation at 365 nm worked well for these previously sluggish or unreactive substrates (see SI for full screening). The contrasting results were obtained for the formation of products **8a** and **8b**, which performed poorly under 440 nm irradiation, (ca. 10 and <2%, respectively), but when subjected to 365 nm irradiation, product was formed in 55 and 49% yields, respectively (Figure 6b). These results further implicate the existence of an excited state BCRC as a key step in the mechanism. We also noticed an increase in the yield of the diazepam substrate (**3q**) to 50% in comparison with the previous conditions. Highly substituted products such as **8d** as well as electron-withdrawing functional groups **8e** and **8f** also were achievable under these conditions. Attempts at extending this protocol to include oxazoline, pyrimidine, or imine directing groups failed, likely due to poor overlap of the irradiation wavelength with the BCRC absorption (see SI). Additionally, employing the oxetane analogue of epoxide **2c** furnished no product.

## Conclusions

In conclusion, we have isolated and characterized a bis-cyclometalated ruthenium complex (BCRC) and studied its photoelectrochemical properties. Our studies demonstrate that BCRCs can undergo photoexcitation and that these excited states can be harnessed to carry out chemically useful reaction steps in a catalytic process. This exceptional reactivity allowed us to develop a mild Ru(II) photocatalyzed *ortho*-alkylation of arenes with epoxides, which has previously eluded ruthenium catalysis. This discovery has the potential to lead to a wide array of new applications for dual-function ruthenium catalysis. We are currently working on a wider application of the BCRC within organic synthesis, which will be reported in due course.

## Supporting Information Available

Experimental procedures and characterization data for all compounds (PDF)
