# Supplementary material for: Bis-Cycloruthenated Complexes in Visible Light-Induced C–H Alkylation with Epoxides
Source: J Am Chem Soc. 2025 Feb 4;147(6):5035–42. doi: 10.1021/jacs.4c14835 (PMC11826993; doi:10.1021/jacs.4c14835)
Supplement: Supplementary file 1 — ja4c14835_si_001.pdf [file ja4c14835_si_001.pdf]

Bis-Cycloruthenated Complexes in Visible Light Induced C-H Alkylation with Epoxides

Kurt Bentley[a], Mishra Deepak Hareram[a], Gang-Wei Wang[a,b], Alexander A. V. Millman[a], Ignacio Perez-Ortega[a], Luke M. Nichols[a], Cassandre C. Bories[a], Lauren E. Walker[a], Adam W. Woodward[a], Alexander P. Golovanov[a], Louise S. Natrajan[a] and Igor Larrosa[a,*]

[a]Department of Chemistry, School of Natural Science, University of Manchester, Oxford Road, Manchester M13 9PL, United Kingdom

[b]State Key Laboratory of Applied Organic Chemistry & College of Chemistry and Chemical Engineering, Lanzhou University, Lanzhou, 730000, China

Contents

Experimental Section 2

1.0. General Information 2

2.0. Reaction Optimisation 4

2.1. 440 nm Wavelength Reaction Optimisation 4

2.2. 365 nm Wavelength Reaction Optimisation 11

3.0. Enantiopure Epoxide 13

4.0. Mechanistic Studies 15

4.1. On/Off Experiment 15

4.2. Stoichiometric Reactions 16

4.2.1. Mono-cyclometallated Ruthenium Reaction 16

4.2.2. Bis-cyclometallated Ruthenium Reaction 20

4.2.3. Addition and irradiation of Alkyl Iodide 2d 25

4.3. Electrochemical Studies 28

4.3.1. Electrochemical Analysis of BCRC 28

4.3.2. Electrochemical Analysis of Mono-Cyclometallated Species 30

4.3.3. Electrochemical Analysis of Reaction Reagents 31

4.4. Absorption Spectroscopy Analyses 32

4.5. Emission Spectra 36

4.5.1. Stern-Volmer Quenching 42

4.6. Evaluation of the Excited State Potential 45

4.6.1. Evaluation of the Excited State Potential of Complex 6a 45

4.6.2. Evaluation of the Excited State Potential of Complex 9a 46

4.7. Lifetime Studies 47

4.8. Quantum Yield Determination 52

4.9. Unsuccessful Substrates 57

4.10. Static Vs Dynamic Quenching 59

4.11. Mechanistic Investigation into the Inhibitory Effect of Hydroxyl 60

4.12. Computational Studies 64

5.0. X-Ray Crystallographic Data 78

6.0 Procedures 84

6.1. General Procedures 84

6.2. Specific Procedures 87

7.0.Preparation of Ruthenium Complexes 88

7.1.Preparation of Mono-cyclometallated Ruthenium Complexes 88

7.2. Preparation of Bis-cyclometallated Ruthenium Complexes 91

8.0. Experimental Section 95

8.1. Synthesis of 2-phenylpyridines 95

8.2. Ruthenium-catalysed Alkylation of Arenes with Epoxides 100

9.0. Spectra 147

9.1. Mono-Cyclometallated Ruthenium Complexes 148

9.2. Bis-Cyclometallated Ruthenium Complexes 160

9.3. Phenylpyridine Derivatives 170

9.4. Alkyl Iodide 184

9.5. Alkylated Products 199

10.0. References 295

Experimental Section

1.0. General Information

All of the alkylation reactions were set up using a schlenk line with oven-dried crimpcap microwave vials (10 mL). The reactions were then capped (using PK100 20MM BUTYL SEPTA) and backfilled with nitrogen 3 times. All starting materials were purchased from Acros (Fisher), Aldrich (Merck), Alpha Aesar (Fisher) and Fluorochem and were used without further purification, unless otherwise stated. All solvents and liquid reagents were degassed with 3 freeze-pump-thaw cycles. Purification by flash column chromatography was carried out on thermo scientific silica gel, particle size 35-75 µm, using the stated solvent system. High resolution mass spectra were performed by the School of Chemistry Mass Spectrometry Service (University of Manchester) employing a Thermo Finnigan MAT95XP spectrometer. IR spectra were recorded using a Thermo Scientific Nicolet iS5 FTIR machine, relevant bands are quoted in cm^{-1}. ^{1}H NMR, ^{19}F NMR and ^{13}C NMR spectra were recorded at 400, 500 and

700 MHz on Bruker instruments. ^{1}H NMR are referenced to the residual solvent peak at 7.26 ppm ($CDCl_3$), 1.94 ppm (CD_3CN) or 7.15 ppm (C_6D_6). Ppm values are quoted to 2 decimal places, with coupling constants (J) to the nearest 0.1 Hz. ^{13}C NMR spectra were recorded at 101, 126 or 176 MHz and quoted in ppm to 1 decimal place with coupling constants (J) to the nearest 0.1 Hz. The spectra were referenced to the residual solvent peak at 77.2 ppm ($CDCl_3$), 1.3 ppm (CD_3CN) 128.1 ppm (C_6D_6). ^{19}F NMR spectra recorded at 471 or 376 MHz in $CDCl_3$ and quoted in ppm to 1 decimal place with coupling constants (J) to the nearest 0.1 Hz. Cyclic voltammetry (CV) experiments were conducted using a PalmSens4 potentiostat, controlled using PSTrace 5.9 software. The working electrode was a GC disc (3 mm dia., BASi part number MF-2012), the counter electrode was a Pt-wire (BASi part number MW-4130) and a Ag/AgCl reference electrode was used (BASi part number – MF-2052). Reactions were performed using a PR160L 440 nm Kessil lamp, a Evoluchem LED 365 nm DX or a Hepatochem LED 525 – 530 nm lamp. All starting materials were used as received with the exception of 2-phenylpyridine which was purified by column chromatography prior to use.

General Experimental Information for NMR Torch Experiment

NMR experiments with *in-situ* sample irradiation inside the NMR spectrometer were performed using the NMRtorch approach[1] using custom-made etched heavy-walled 5 mm quartz tubes sealed with a plug made of 7 mm long polished 5 mm diameter quartz rod, attached to the tube end with a piece of FEP tubing, and with a layer of vacuum grease placed between the rim of the tube and the edge of the rod, to prevent oxygen ingress into the sample in the course of experiments. Sample was filled into the tubes and sealed in an argon filled glovebox. Samples were illuminated in a Brucker Ascend 700 MHz NMR spectrometer equipped with a cryoprobe and Avance Neo console. A series of 1D ^{1}H spectra were recorded at 60 s intervals, with the illumination switched on using TTL trigger. Sample temperature inside the NMR

magnet was controlled at 15 ºC by VT gas, and the additional sample heating due to illumination was estimated to be well under 1°C. The sample was illuminated using a lighthead housing a 460 nm LED array with nominal electrical power of 10 W. NMR spectra were analysed using Topspin and MestReNova.

2.0. Reaction Optimisation

2.1. 440 nm Wavelength Reaction Optimisation

Table 1. Screening of Solvents

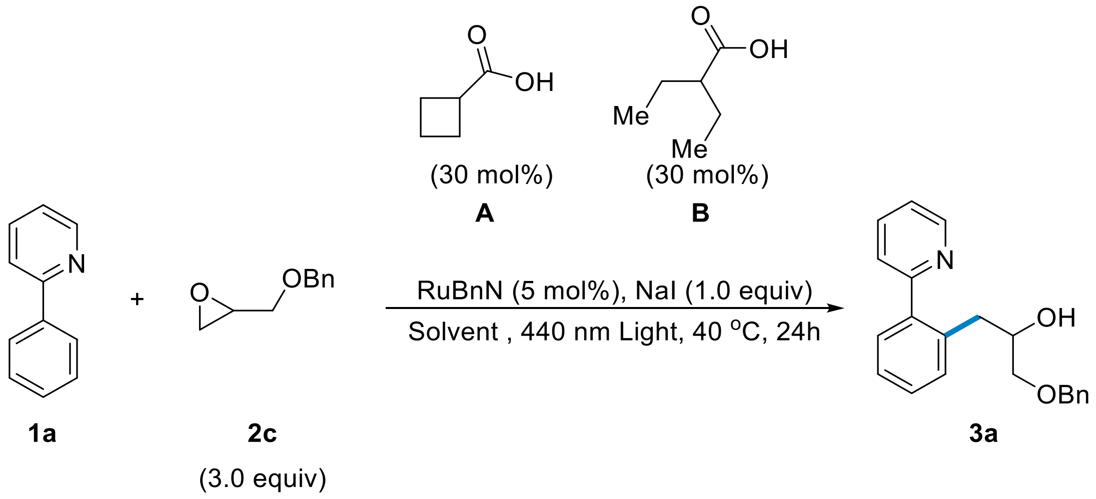

**Entry**	Solvent	Epoxide Equivalence	Acid	**3a** (%)[a]
1	THF	3.0	A	78
2	Acetone	3.0	A	52
3	HFIP	3.0	A	52
4	HFIP/AcOH (8:2)	3.0	A	7

**5**	**EtOAc**	**3.0**	**A**	**78**
6	Toluene	3.0	B	70
7	MeOH	2.0	B	18
8	Dioxane	2.0	B	37
9	DMF	2.0	B	-

[a]*Yields determined by ^{1}H NMR spectroscopy using 1,3,5 – trimethoxybenzene as an internal standard.*

Table 2. Base/Acid Screening

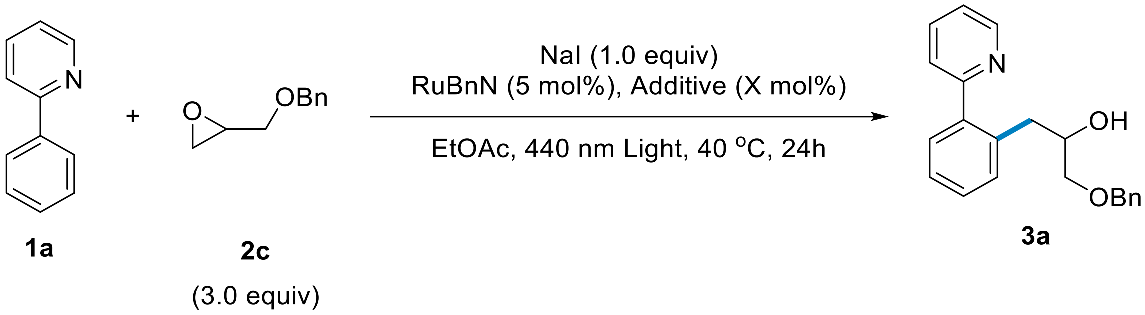

**Entry**	Additive	Additive Equivalence (mol%)	**3a** (%)[a]
1	$NaHCO_3$	30	24
2	$NaHCO_3$	50	59
3	$NaHCO_3$	100	57
4	KH_2PO_4	30	21
5	KOAc	100	62
**6**	**Cyclobutanecarboxylic acid**	**30**	**78**
7	Cyclobutanecarboxylic acid	50	51
8	Cyclobutanecarboxylic acid	100	52
9	2-ethylbutanoic acid	30	72
10	2-methyl-2-phenyl propanoic acid	30	37
11	Pivalic acid	30	29
12	1-Ad-COOH	30	24

[a]*Yields determined by ^1H NMR spectroscopy using 1,3,5 – trimethoxybenzene as an internal standard.*

Table 3. Epoxide Equivalence Screening

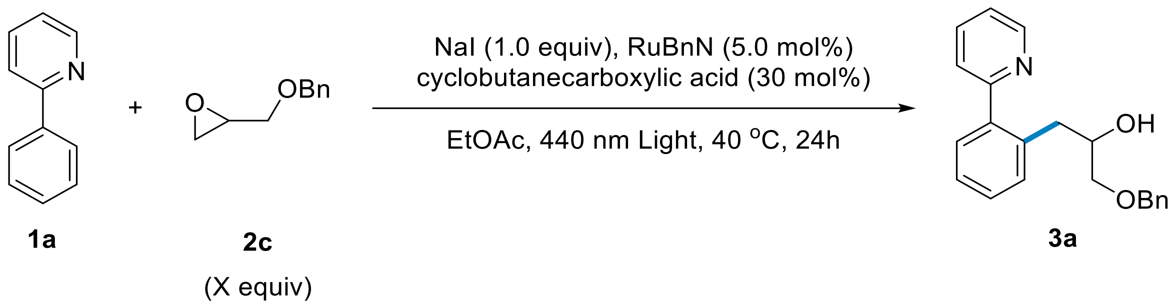

**Entry**	Epoxide Equivalence	**3a** (%)[a]
**1**	**3.0**	**78**
2	2.0	51
3	1.0	28

[a]Yields determined by ^{1}H NMR spectroscopy using 1,3,5 – trimethoxybenzene as an internal standard.

During our studies, we found that an excess of the epoxide is essential for high reactivity to occur. This is due to the side reactivity that occurs for the epoxide. We observe formation of a ketone which is consistent with elimination of the iodide. These alternative pathways mean a large excess of the epoxide is required.

Table 4. Catalyst Screening

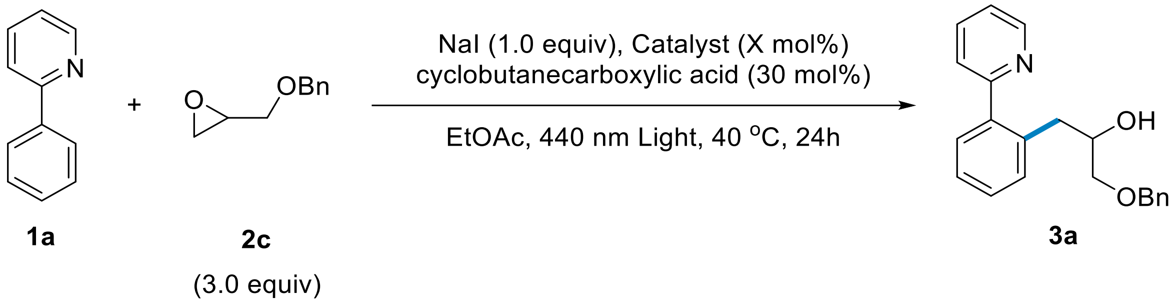

Entry	Catalyst	Solvent	Catalyst Loading	**3a** (%)[a]
1	RuBnN	EtOAc	5.0	78
2	RuBnN	EtOAc	10	66
3	RuBnN	EtOAc	15	62
4	RuBnN	EtOAc	20	66
5	[(*p*-cymene)$RuCl_2$]$_2$	EtOAc	2.5	81
**6**	**[(*p*-cymene)$RuCl_2$]$_2$**	EtOAc	**5.0**	**83**
7	[(benzene)$RuCl_2$]$_2$	EtOAc	2.5	23
8	[(benzene)$RuCl_2$]$_2$	EtOAc	5.0	32
9	$RuCl_2(PPh_3)_3$	EtOAc	5.0	nr
10	RuAqua	EtOAc	5.0	11
11	RuAqua	Acetone	5.0	61

[a]*Yields determined by ^{1}H NMR spectroscopy using 1,3,5 – trimethoxybenzene as an internal standard.*

Table 5. Wavelength Screening

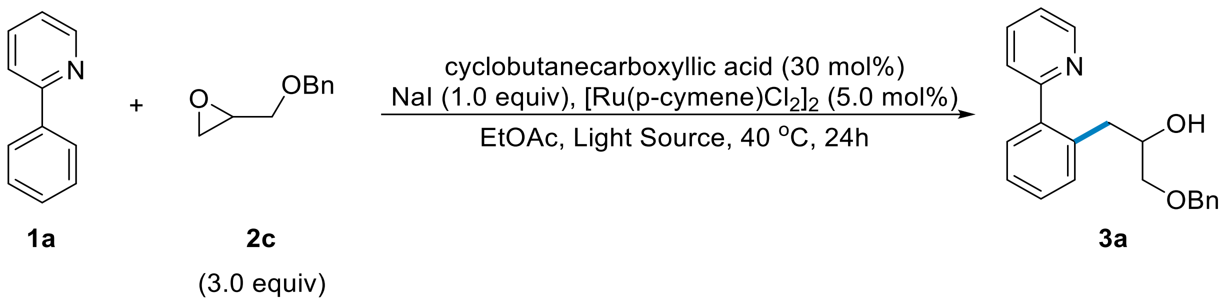

Entry	Conditions	**3a** (%)[a]
**1**	**440 nm**	**83**
2	427 nm	69
3	365 nm	68

[a]*Yields determined by ^{1}H NMR spectroscopy using 1,3,5 – trimethoxybenzene as an internal standard.*

Table 6. Halide Source Screening

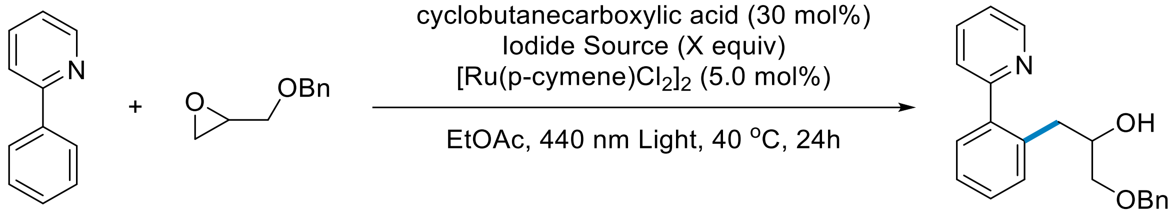

1a **2c** (3.0 equiv) **3a**

**Entry**	Halide Source	Halide Source Equivalence	**3a** (%)[a]
**1**	**NaI**	**1.0**	**83**
2	NaI	0.75	59
3	NaI	0.5	51
4	NaI	0.25	16
5	KI	1.0	52
6	*n*Bu_4NI	1.0	57
7	LiI	1.0	32
8	NaBr	1.0	-
9	KBr	1.0	-
10	NaCl	1.0	-

[a]*Yields determined by ^{1}H NMR spectroscopy using 1,3,5 – trimethoxybenzene as an internal standard.*

Table 7. Control Experiments

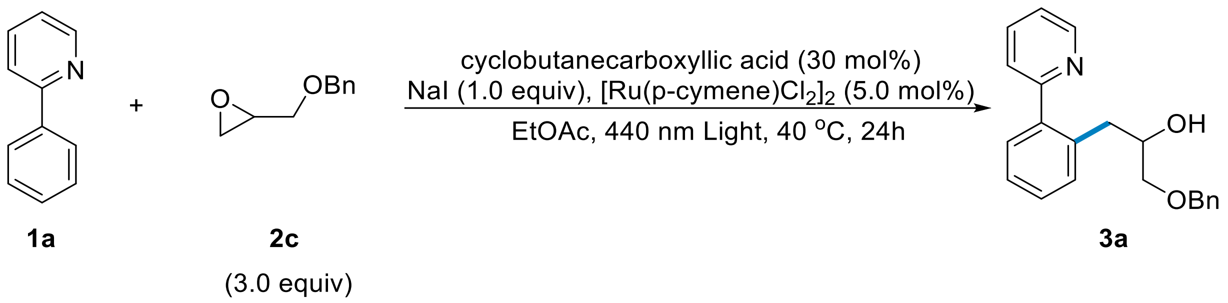

Entry	Change	**3a** (%)[a]	**1a** (%)[a]
1	No Catalyst	-	89
2	No NaI	-	78
3	No Acid	-	79
4	No light, 40 °C	-	75

[a]*Yields determined by ^{1}H NMR spectroscopy using 1,3,5 – trimethoxybenzene as an internal standard.*

2.2. 365 nm Wavelength Reaction Optimisation
Table 8. Solvent Screening

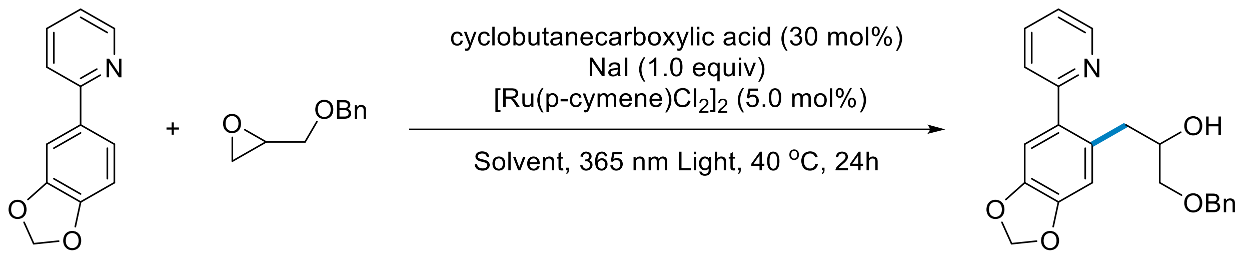

1u **2c** (3.0 equiv) **8a**

Entry	Solvent	**8a** (%)[a]
1	EtOAc	35
**2**	**Acetone**	**46**

[a]*Yields determined by ^{1}H NMR spectroscopy using 1,3,5 – trimethoxybenzene as an internal standard.*

Table 9. Additive Screening

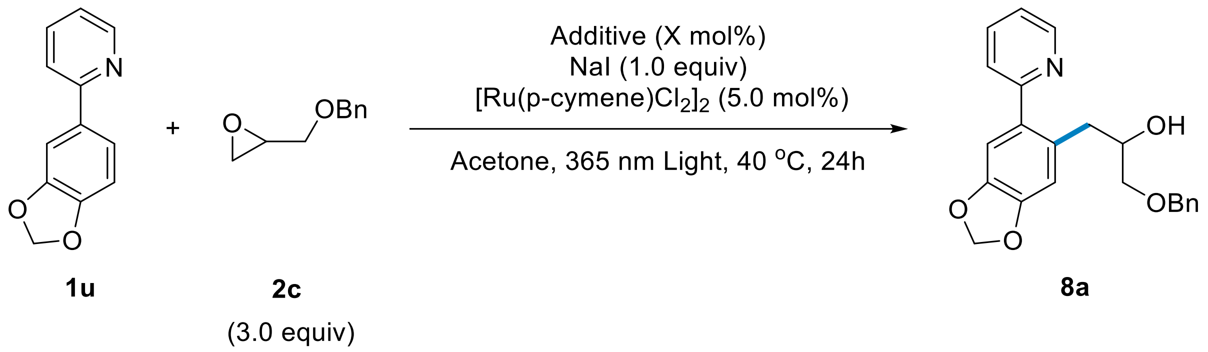

Entry	Additive	**8a** (%)[a]
1	PhCOOH (30 mol%)	60
2	CyclobutaneCOOH (1.0 equiv)	nr
3	K_2CO_3 (30 mol%)	nr
4	KOAc (30 mol%)	18
5	CyclobutaneCOOH (30 mol%) + K_2CO_3 (30 mol%)	50
**6**	**1-AdCOOH (30 mol%)**	**62**
7	2-AdCOOH (30 mol%)	23
8	*p*-OMe benzoic acid (30 mol%)	trace
9	*p*-CF_3 benzoic acid (30 mol%)	23
10	MesCOOH (30 mol%)	24

[a]*Yields determined by ^{1}H NMR spectroscopy using 1,3,5 – trimethoxybenzene as an internal standard.*

3.0. Enantiopure Epoxide

The standard reaction using 2-phenylpyridine (**1a**) was repeated using an enantiopure epoxide to confirm the retention of stereochemistry at the substituted epoxide carbon. Gratifyingly, complete retention of the stereochemistry occurs leading to the enantiopure product.

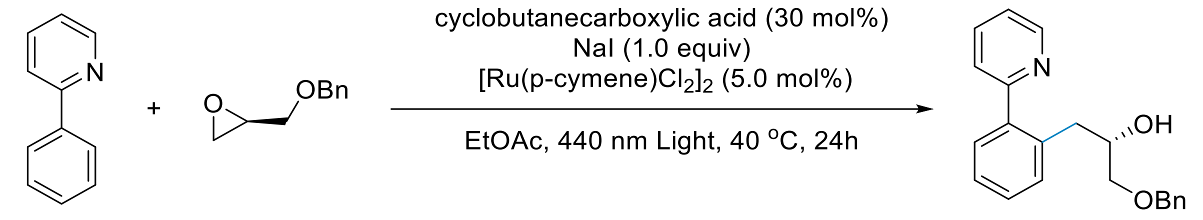

1a 0.2 mmol

2c' (3.0 equiv)

3a 74% Yield, 97% ee

*Figure 1. Enantiopure epoxide **2c'** under the standard conditions*

Racemic Product Trace (3a)

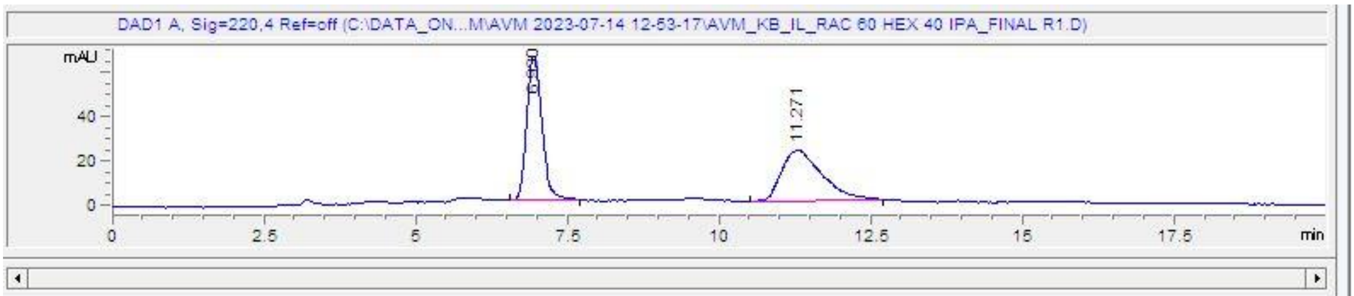

#	Time	Area	Height	Width	Area%	Symmetry
1	6.93	1111.8	65.3	0.2586	50.541	0.747
2	11.271	1088.1	23.1	0.6126	49.459	0.565

Conditions: 60% Hexane: 40% IPA_ Daical Chiracel OD-H column, 1 mL min flow rate

Enantiopure Product Trace (3a)

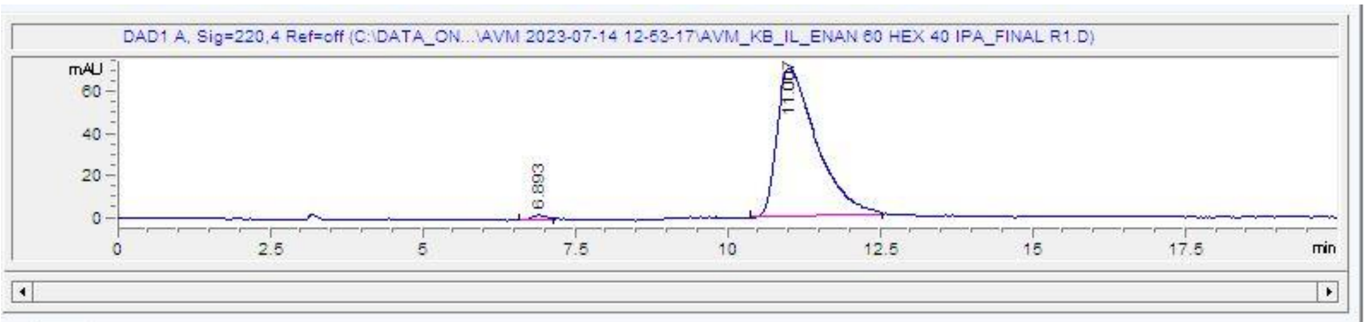

#	Time	Area	Height	Width	Area%	Symmetry
1	6.893	43.5	2.5	0.2159	1.339	1.023
2	11.007	3207.9	70.9	0.6135	98.661	0.456

Conditions: 60% Hexane: 40% IPA_ Daical Chiracel OD-H column, 1 mL min flow rate

Figure 2. HPLC traces for the enantiopure epoxide

4.0. Mechanistic Studies

4.1. On/Off Experiment

An on/off experiment was designed in order to elucidate the role of the light, this reaction was performed in an NMR tube and monitored over time. The fluorinated phenyl pyridine **1m** was chosen as an ideal substrate for this investigation. We found that product formation only occurred in the presence of light, indicating that the light has an essential role within the mechanistic cycle.

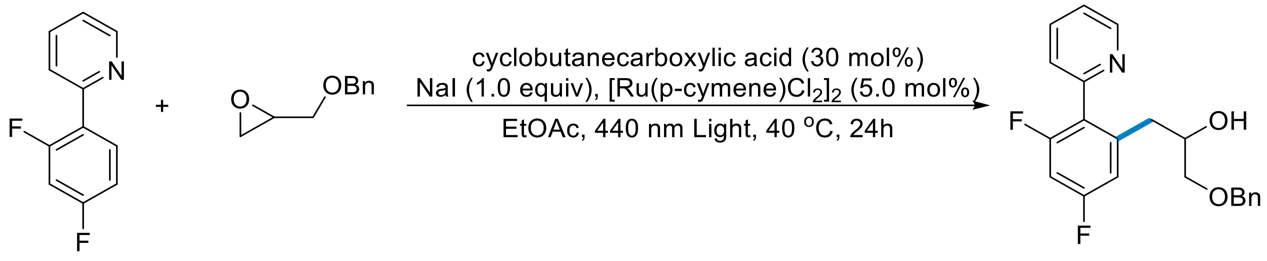

1m (0.5 mmol) **2c** (3.0 equiv) **3m**

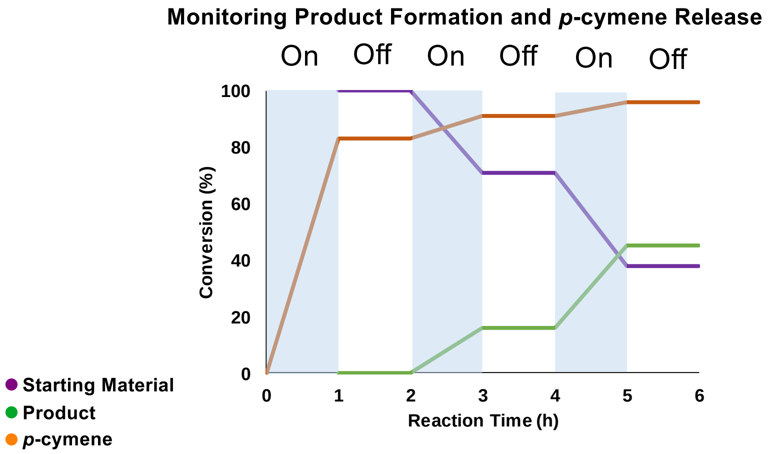

Figure 3. [a]Yields determined by ^{1}H NMR spectroscopy using 1,3,5 – trimethoxybenzene as an internal standard.

4.2. Stoichiometric Reactions

4.2.1. Mono-Cyclometallated Ruthenium Reactions

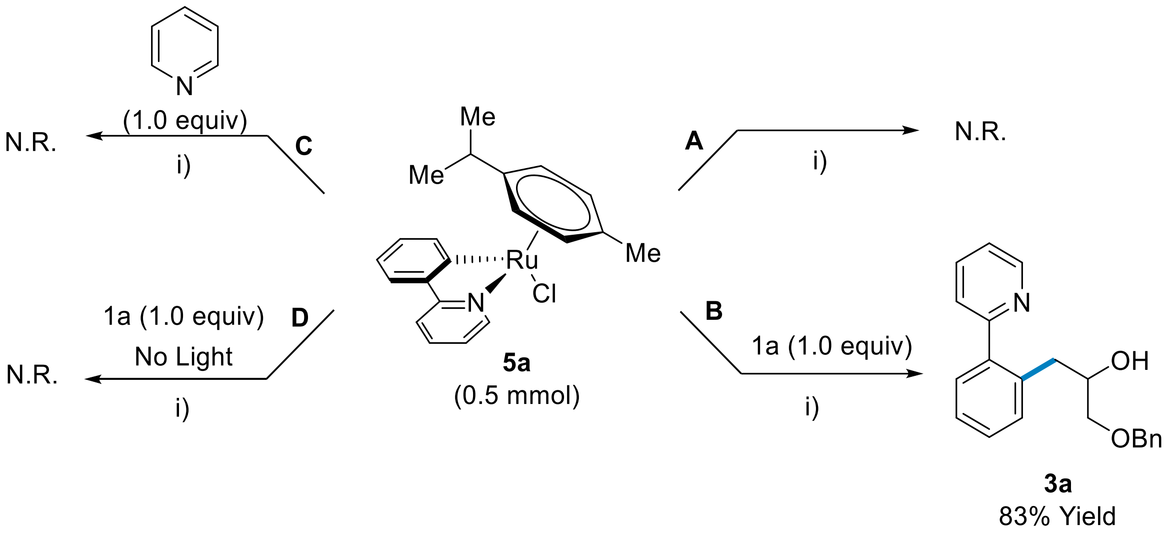

Figure 4a.i) 2c (3 equiv), cyclobutanecarboxylic acid (30 mol%), NaI (1.0 equiv) 440 nm light, 40 C 24 h [a]Yields determined by ^{1}H NMR spectroscopy using 1,3,5 – trimethoxybenzene as an internal standard.

Reaction of the mono-cyclometallated species **5a** in the absence of a second 2-phenylpyridine (**1a**) equivalent did not lead to any product formation (Figure 4a A). Upon addition of a second equivalent of **1a**, product formation occurred (Figure 4a B). Additionally, when an equivalent of pyridine was added, no product formation occurred (Figure 4a C). Finally, we confirmed that the mono-cyclometallated species does not form the product at 40 ^{o}C with an equivalent of 2PhPy (**1a**). These results together indicate that the bis-cyclometallated species is a key intermediate under these reaction conditions.

A cationic mono-cyclometallated ruthenium complex (**4a**) was also studied (Figure 4b). Irradiation of the complex under the reaction conditions led to no product formation (Figure 4b A). The addition of a second equivalent of **1a** without light irradiation led to no product formation (Figure 4b B). Finally, the addition of a second equivalent of **1a** as well as light irradiation generated the product in a yield of 89% (Figure 4b).

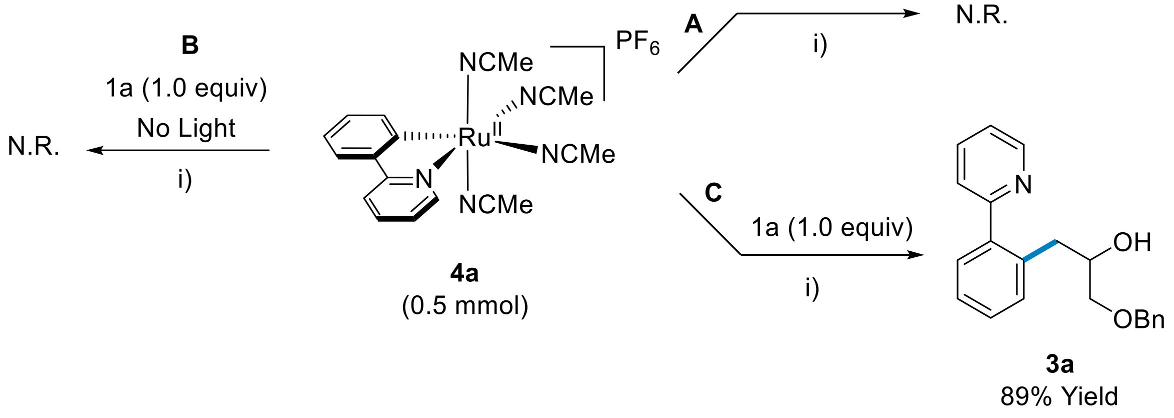

Figure 4b i) 2c (3 equiv), cyclobutanecarboxylic acid (30 mol%), NaI (1.0 equiv) 440 nm light, 40 C 24 h. [a]Yields determined by ^{1}H NMR spectroscopy using 1,3,5 – trimethoxybenzene as an internal standard.

In order to indicate that the mono species is not the key intermediate within these reactions, we performed a series of stoichiometric experiments (Figure 4c). We found complex **4b** unreactive in the presence of alkyl iodide as well as with an additional quantity of **1p** (Figure 4c i/ii) suggesting neither mono cyclometallation or arene coordination is sufficient for the reaction to occur. Addition of an ancillary quantity of a non-cyclometallating ligand (Figure 4c iii) alongside a base also showed no reactivity, indicating that the base's role is to facilitate the cycloruthenation rather than as a ligand.

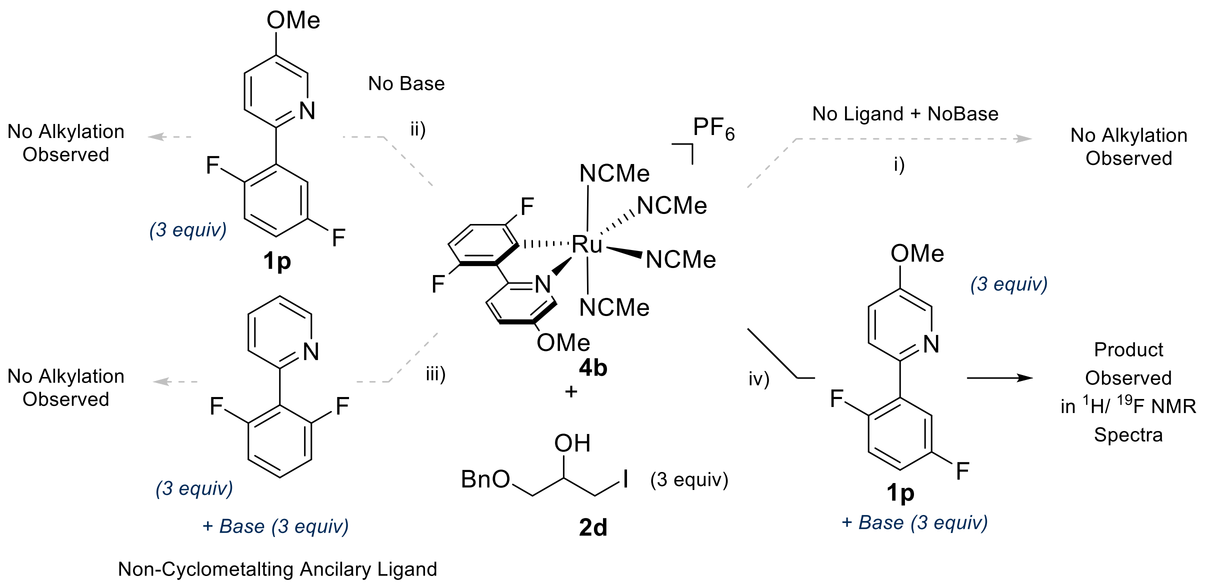

*Figure 4c. Stoichiometric Reactivity of **4b** with alkyl iodide **2d**. Reaction Conditions = 0.5 mmol scale, EtOAc [0.57 M], 40 °C, 440 nm Light, 2 h, base = sodium cyclobutane carboxylate*

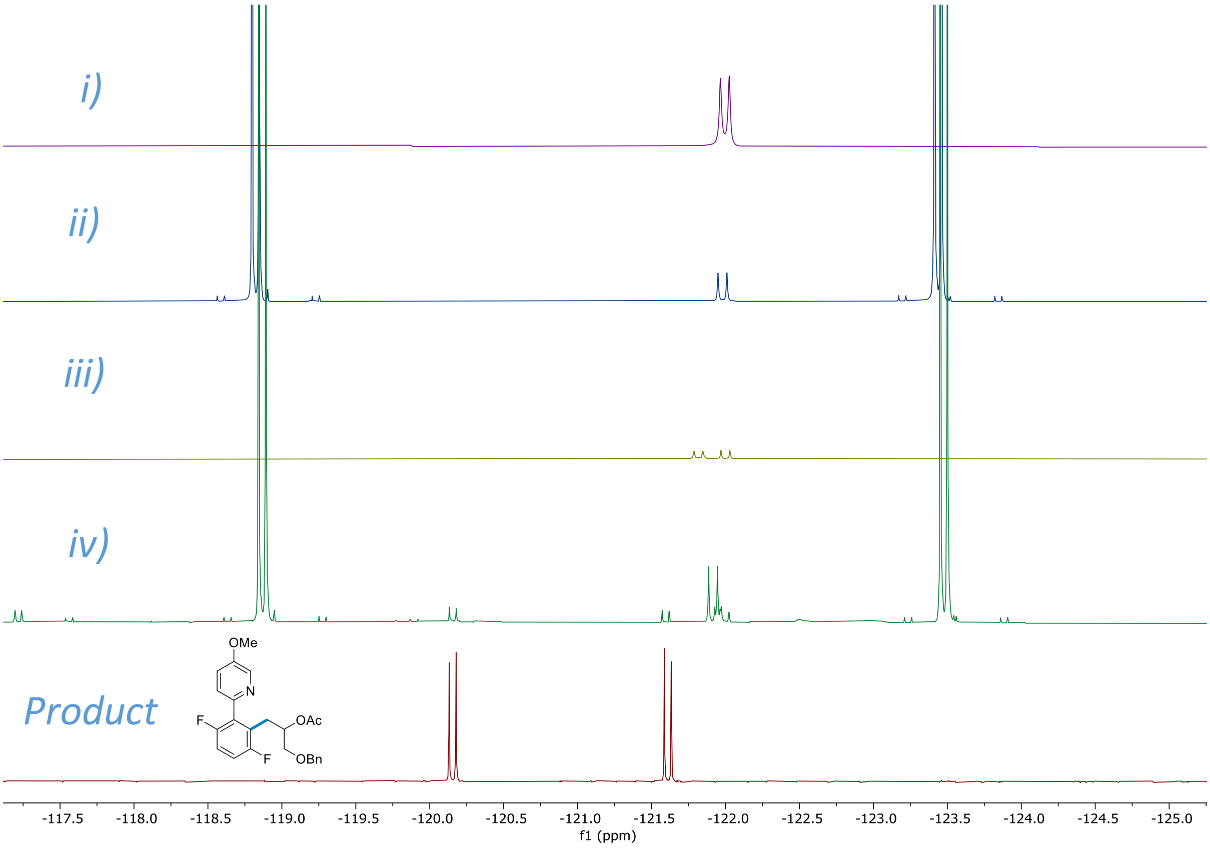

*Figure 4d. Crude $^{19}F^{2}$ (376 MHz, $CDCl_3$), NMR spectra of stoichiometric experimetns of monocyclometallated ruthenium complex **4b** in figure **4c.***

We attempted to add an external photocatalyst to our reaction conditions but did not observe any product formation. (figure 4e)

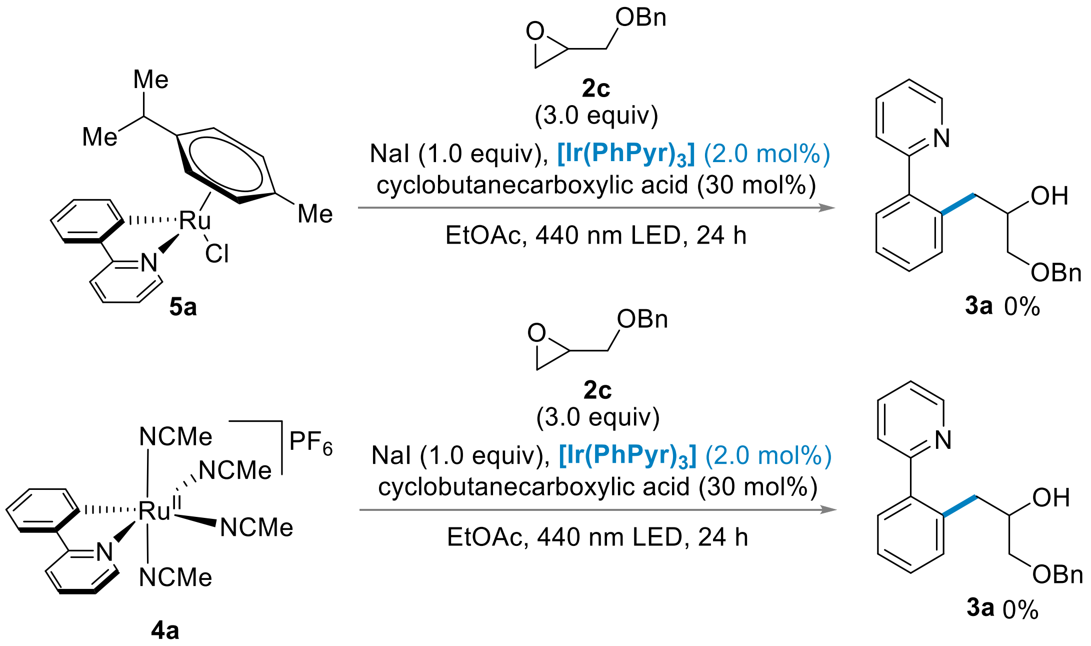

Figure 4e. Addition of an external photocatalyst to stoichiometric mono-cyclometallated reaction

We attempted a stoichiometric experiment using complex **4b** under the standard conditions. (figure 4f) This gave the product in a yield of 21%.

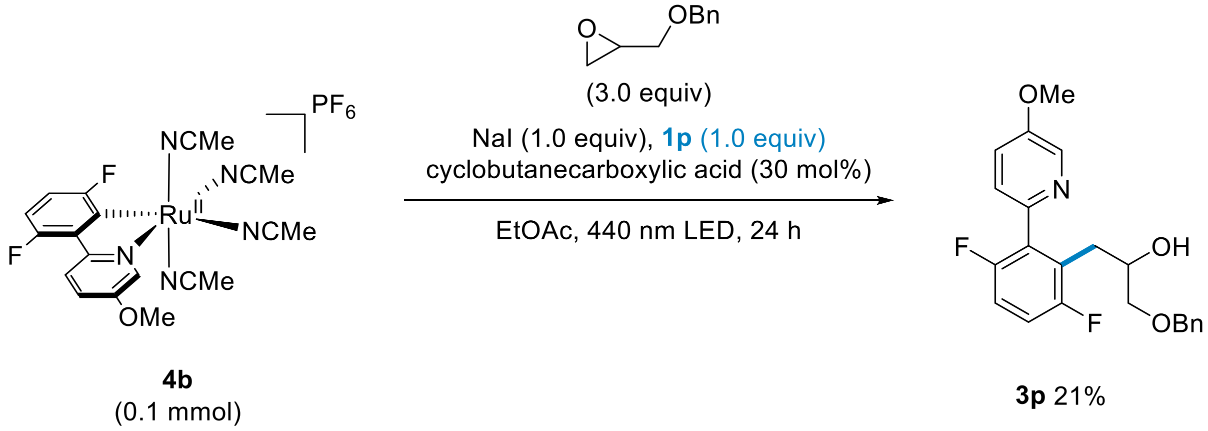

*Figure 4f. Addition of stoichiometric amounts of **4b** to the standard conditions*

4.2.2. Bis-Cyclometallated Ruthenium Reactions

The BCRC (**6a**) was reacted with both the alkyl iodide (**2d**) and the epoxide (**2c**). (Figure 5a) Although no product formation occurs for the unprotected alkyl iodide **2d**, protodemetallation of the complex **6a** does occur.

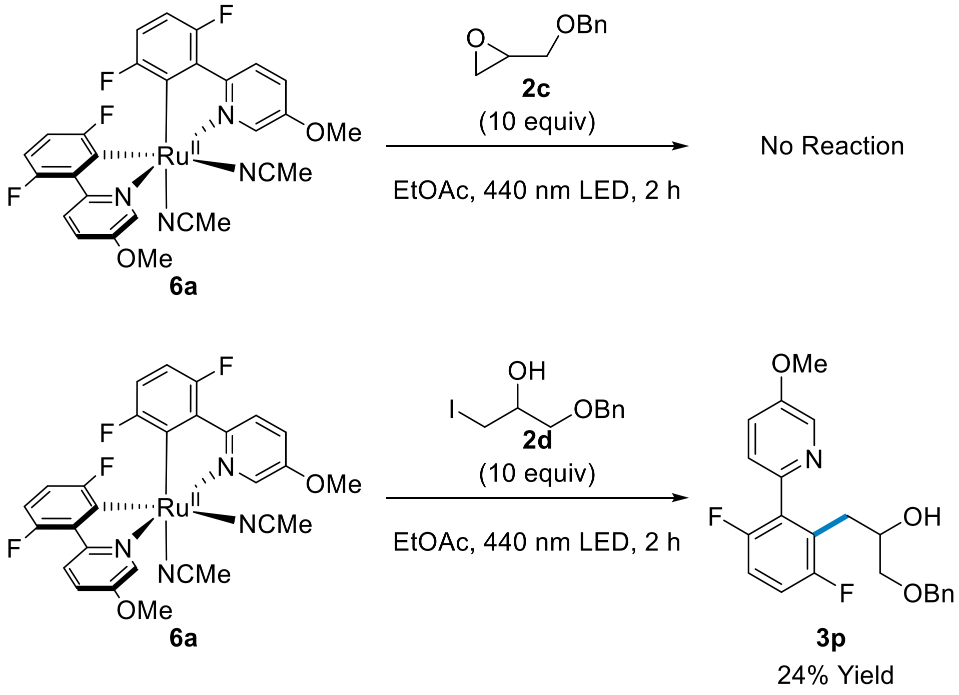

*Figure 5a. Stoichiometric addition of the alkyl iodide (**2d**) and epoxide (**2c**) to BCRC **6a***

To test whether we could push the stoichiometric reaction to proceed thermally, we heated the BCRC **6a** in the presence of the alkyl iodide **2d** at 80 °C and found that product formation occured. (Figure 5b)

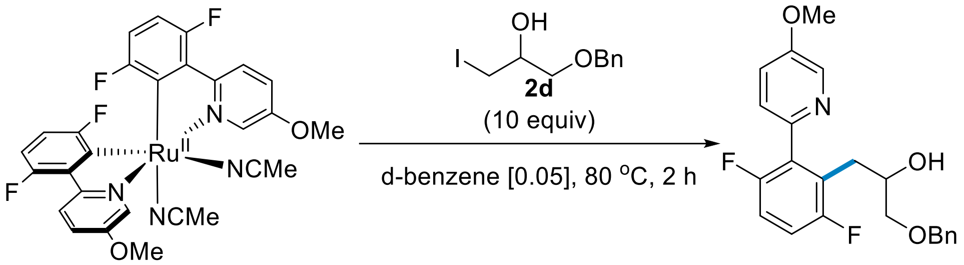

6a
(0.025 mmol)

3p
20% Yield

*Figure 5b. Stoichiometric addition of the alkyl iodide (**2d**) to BCRC **6a** under thermal conditions*

We attempted a stoichiometric experiment using complex **6a** under the standard conditions. This gave the product in a yield of 25%. (figure 5c)

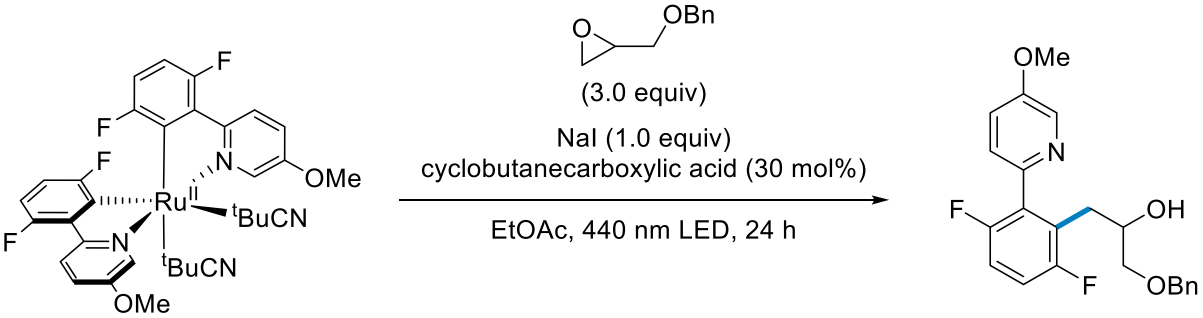

6a
(0.1 mmol)

3p 25%

*Figure 5c. Addition of stoichiometric amounts of **6a** to the standard conditions*

During these studies, we noticed that no reaction occurred initially between the alkyl iodide and the bis-cyclometallated species. We theorised that the oxygen coordination is essential to inhibiting the reaction occurring immediately and that either high temperatures or access to an excited state needs to be achieved in order to facilitate reactivity. To test this hypothesis, we added the alkyl iodide **2b** to the BCRC **6a**. (Figure 6)

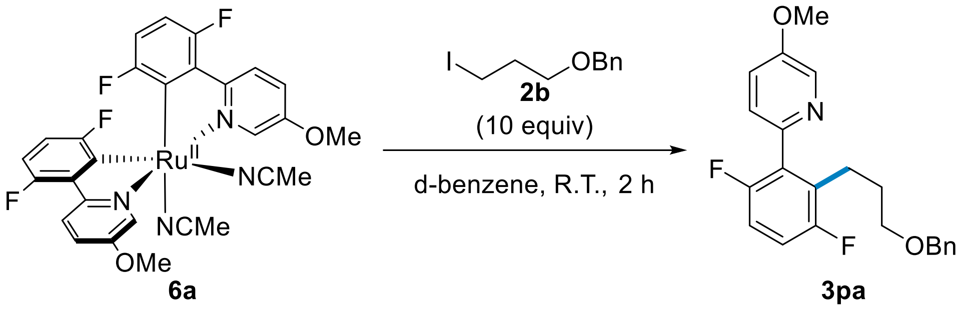

*Figure 6. Stoichiometric addition of the alkyl iodide (**2b**) to BCRC **6a***

Addition of the alkyl iodide **2b** to the BCRC **6a** led to instantaneous reactivity leading to the alkylated product **3pa**. This result indicates that oxygen coordination is inhibiting the reaction from occurring in the ground state.

Substrate **2d** would not be suitable for the Stern-Volmer studies since protodemetallation is also observed. Therefore, another alkyl iodide is needed in order to perform the Stern-Volmer quenching studies. So, experimentation was carried out using the protected alkyl iodide **2e**, which also did not react with **6a** in the ground state but did in the excited state. More importantly, this did not lead to protodemetallation in the ground state. (Figure 7)

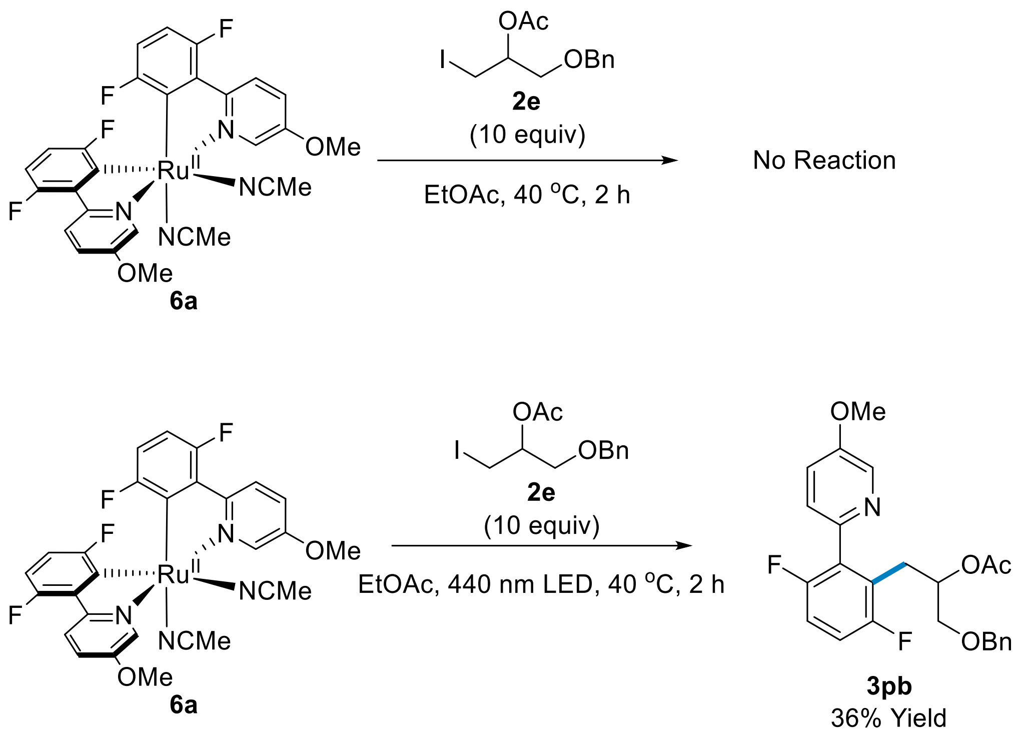

*Figure 7. Reactivity of BCRC **6a*** with alkyl iodide **2e**

Upon reacting the BCRC species **6a** with alkyl iodide species **2g**, which cannot eliminate to form the ketone, we observed a cyclisation product that would be formed through a hydrogen atom transfer (HAT) followed by cyclisation. This result indicates the formation of radicals within our reaction conditions. This cyclisation product is observed both thermally (40 °C) and photochemically (440 nm), however, consistent with our reactivity comparisons between thermal and photocatalyzed process, the light mediated process is significantly faster. (Figure 8)

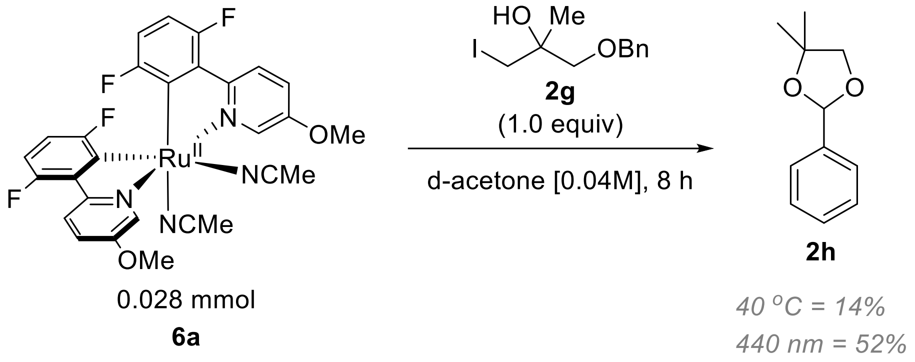

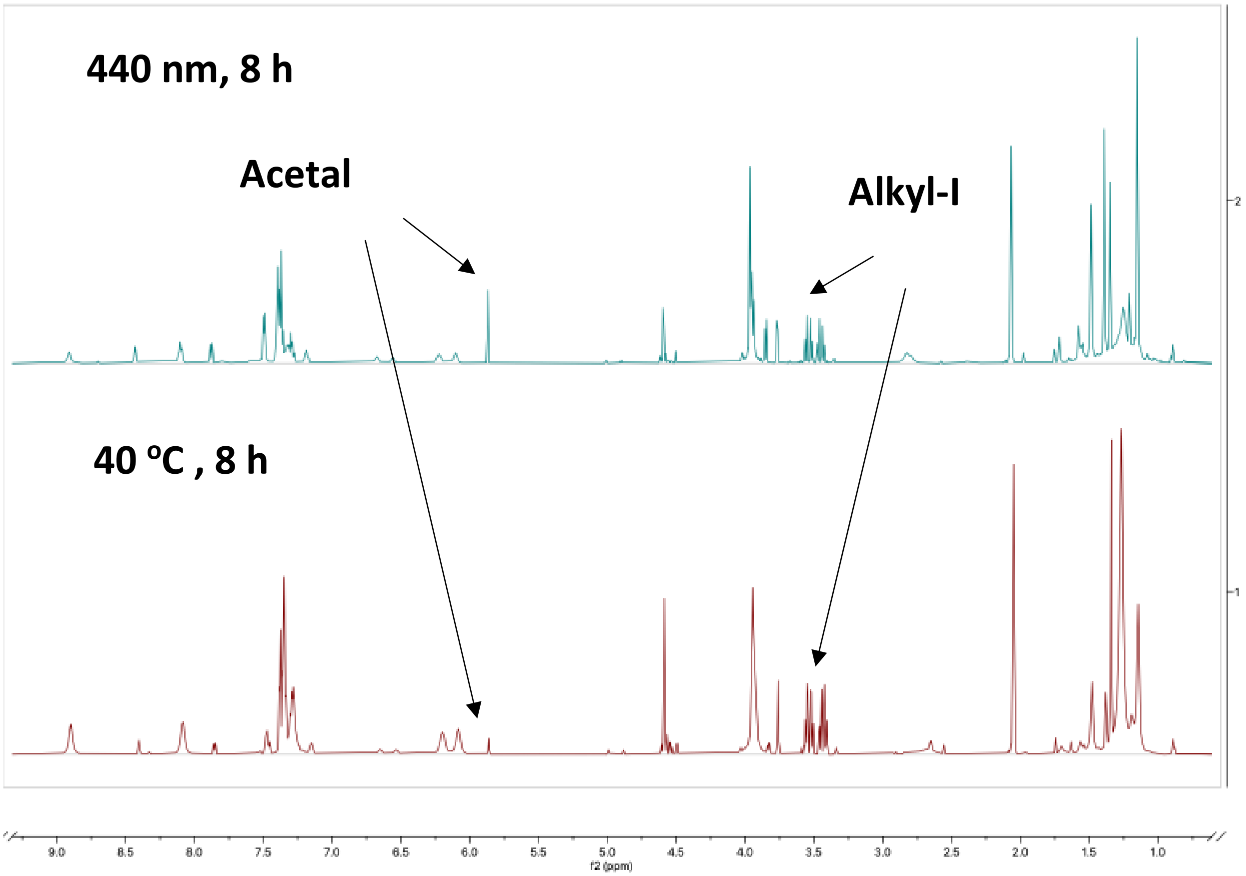

Figure 8. Reaction of BCRC **6a** with alkyl iodide **2g** forming the cyclised product **2h** thermally and photochemically

4.2.3. Addition and Irradiation of Alkyl Iodide 2d

In order to confirm the alkyl iodide **2d** as the active species within the reaction, a reaction was performed using 3.0 equivalence of the alkyl iodide. We found that product formation occurred to give the product **3a** in a yield of 31%. (Figure 9)

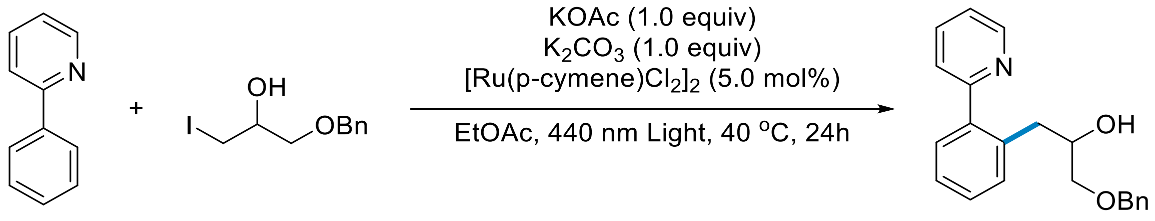

1a **2d** (3.0 equiv) **3a** 31% Yield

*Figure 9. Stoichiometric addition of the alkyl iodide (**2d**) to the standard conditions*

In order to analyse the effects of exposing the alkyl iodide **2d** to light irradiation, an NMR experiment was performed. The alkyl iodide **2d** was irradiated for 24 h at both 440 nm and 365 nm in EtOAc and the resulting ^1H NMR was recorded in $CDCl_3$. In both cases, the alkyl iodide (**2d**) remained unreacted.

Irradiation at 440 nm:

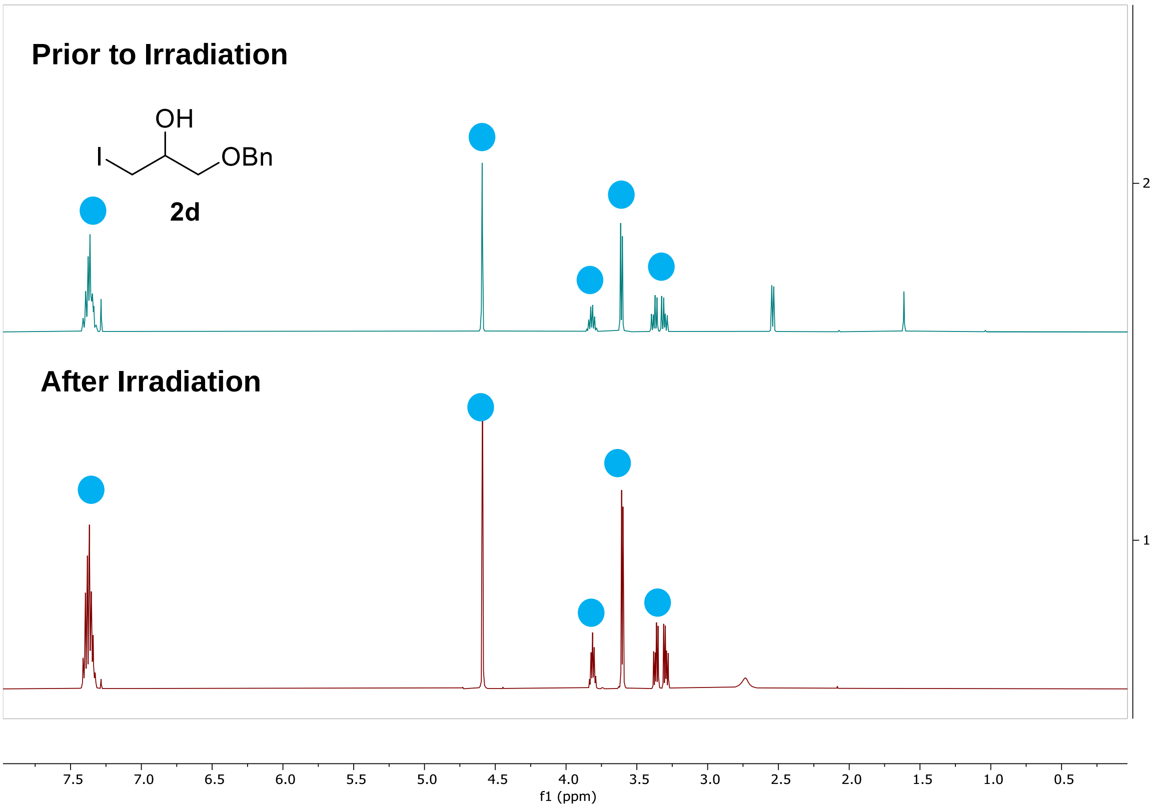

Irradiation at 365 nm:

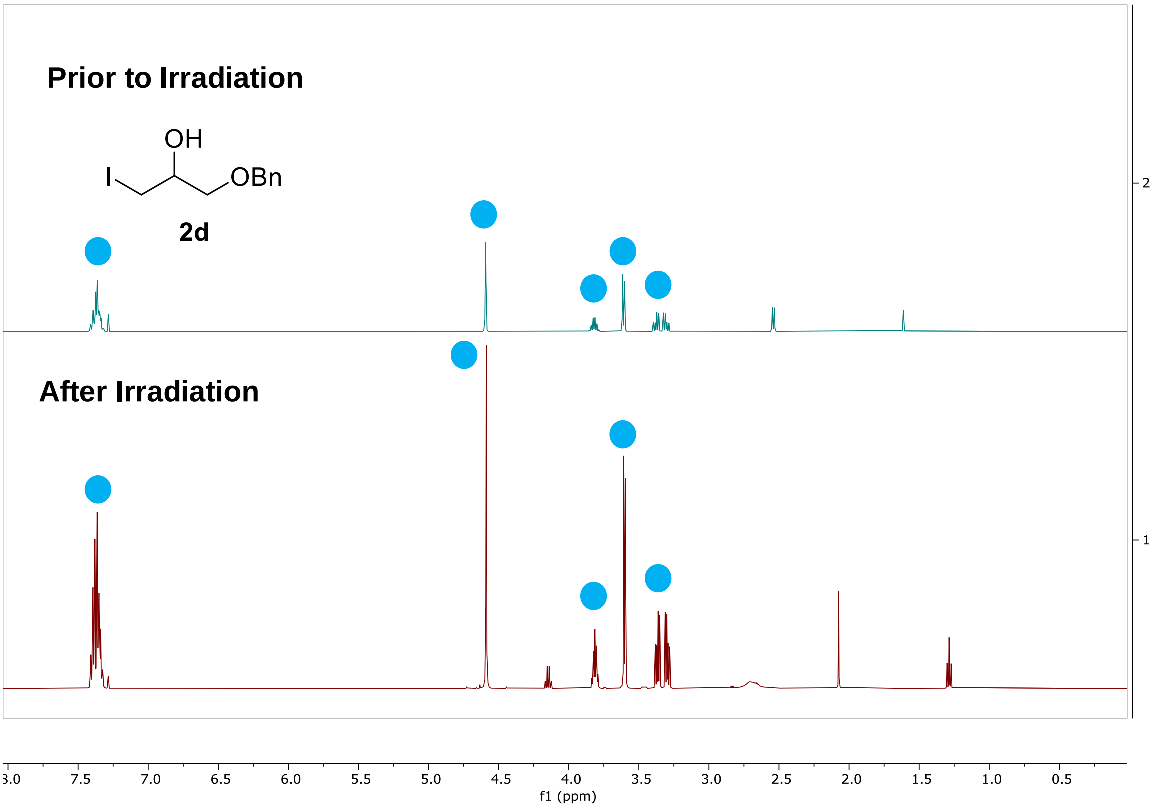

4.3. Electrochemical Studies

4.3.1. Electrochemical Analysis of BCRC

Cyclic Voltammetry (CV) experiments were conducted in a 10 mL glass vial fitted with a glassy carbon working electrode (3 mm dia., BASi), a Ag/AgCl reference electrode and a platinum wire counter electrode. The CV was performed in an argon filled glovebox. The parent data was referenced relative to the $Fc^{+/0}$ couple that was recorded.

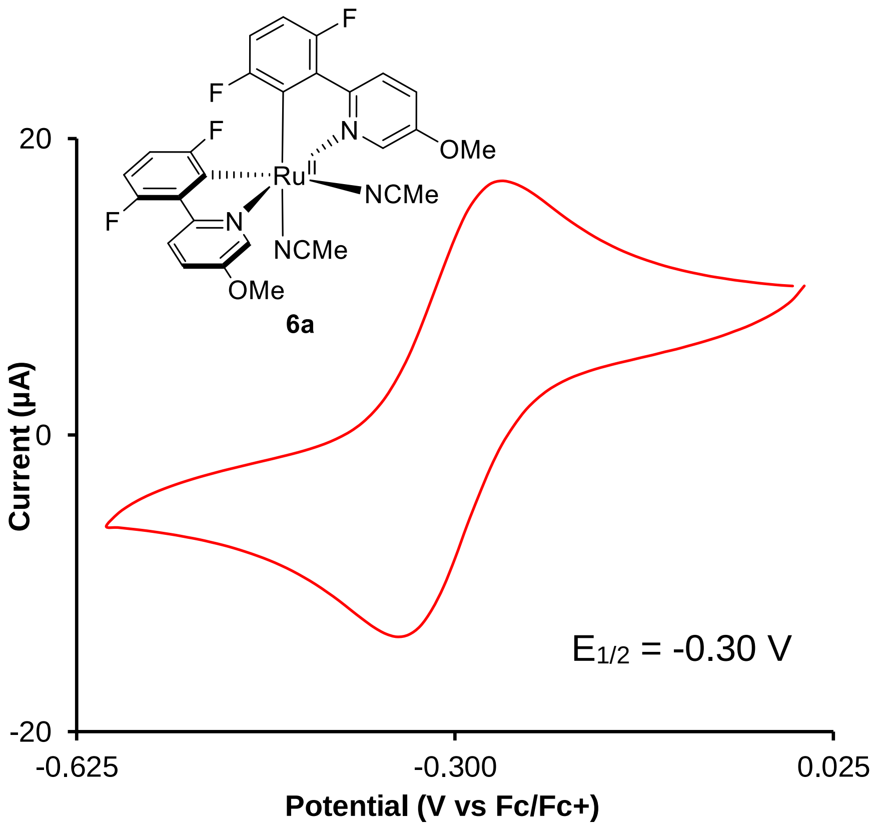

*Figure 10. Oxidative scan for the OMe bis-cyclometallated species (**6a**)*

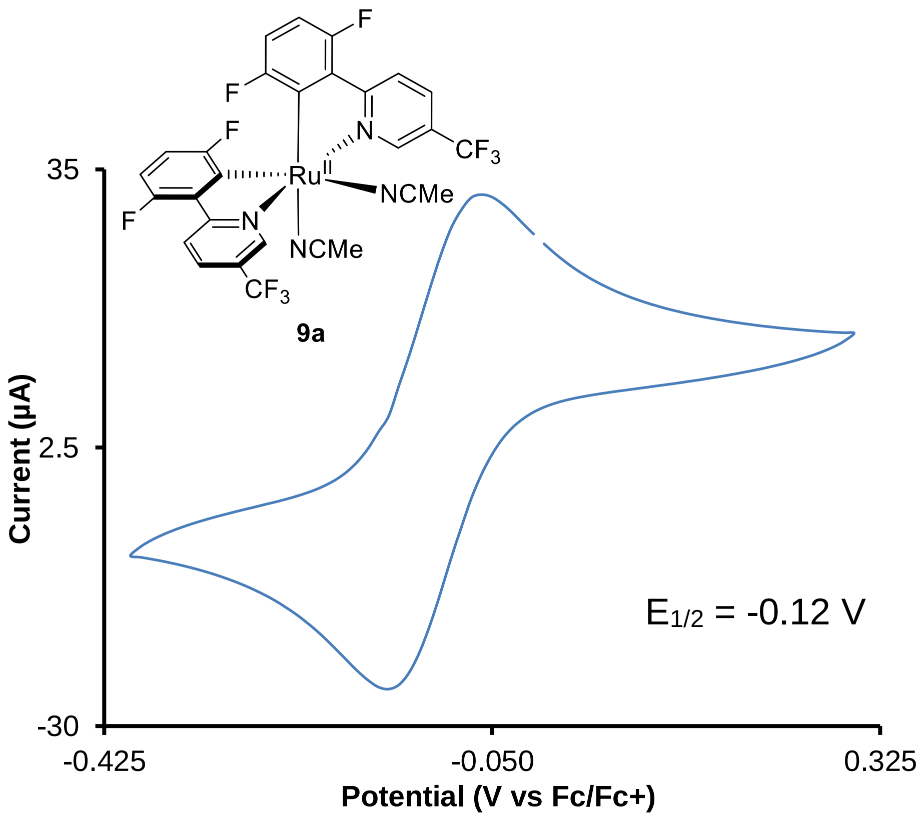

*Figure 11. Oxidative Scan of the CF_3 bis-cyclometallated species (**9a**)*

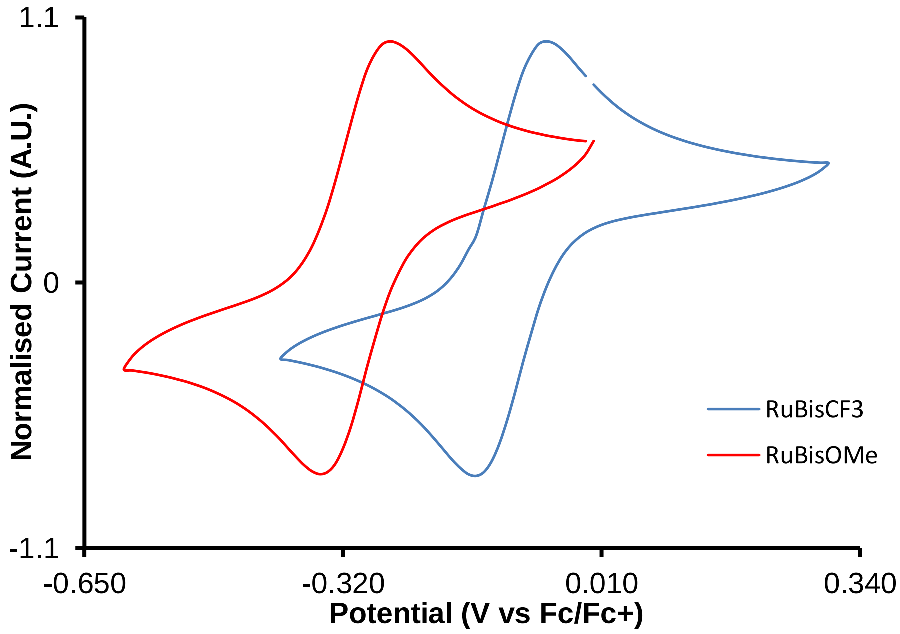

*Figure 12. Comparison of the reduction scans for both the BCRC Species (**6a** and **9a**)*

4.3.2. Electrochemical Analysis of Mono-Cyclometallated Species

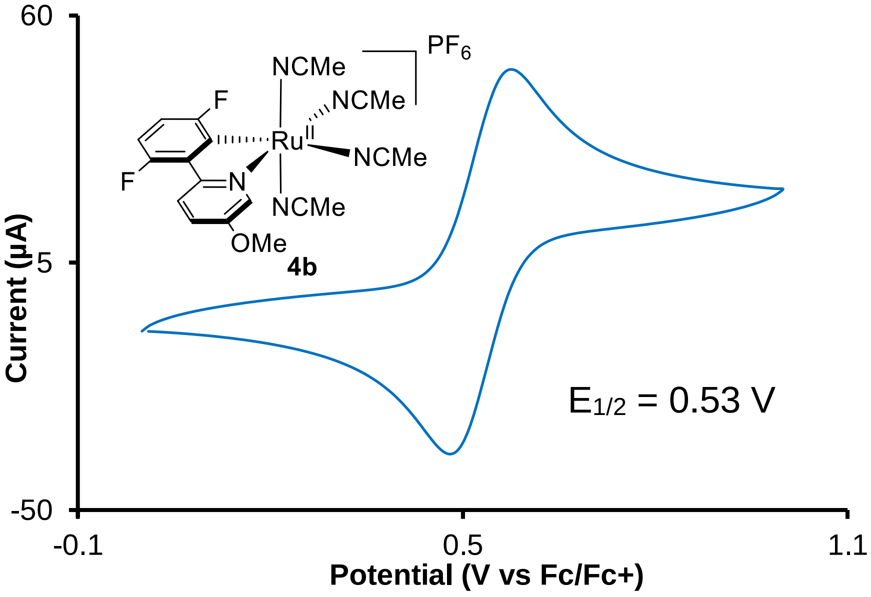

*Figure 13. Oxidative scan for the OMe Mono-Cyclometallated species (**4b**)*

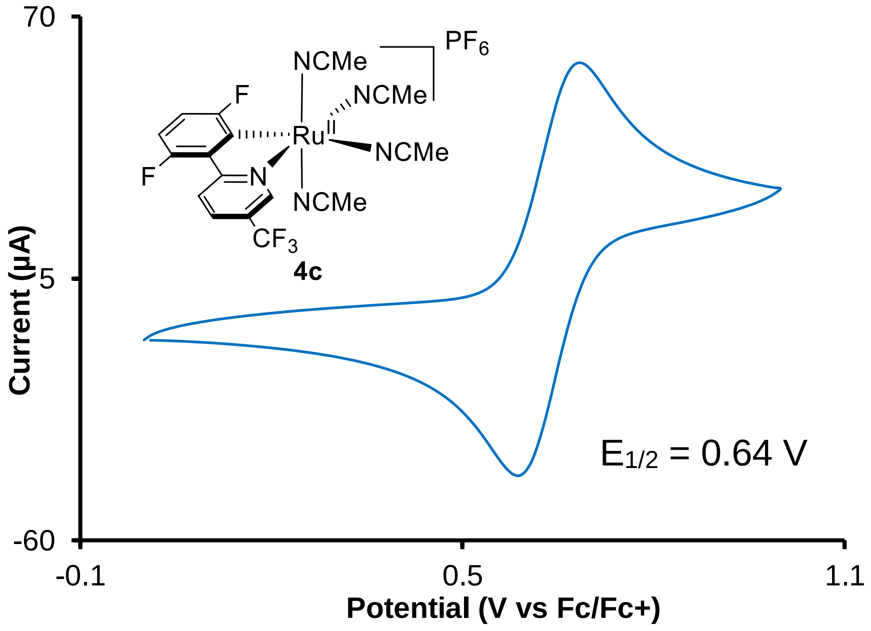

*Figure 14. Oxidative scan for the CF_3 Mono-Cyclometallated species (**4c**)*

4.3.3. Electrochemical Analysis of Reaction Reagents

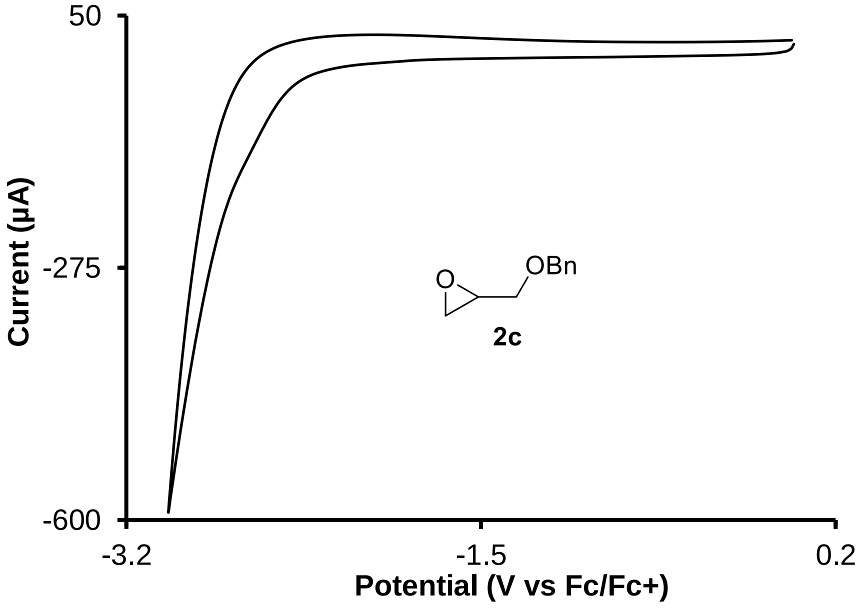

*Figure 15. Reductive Scan of the Epoxide (**2c**)*

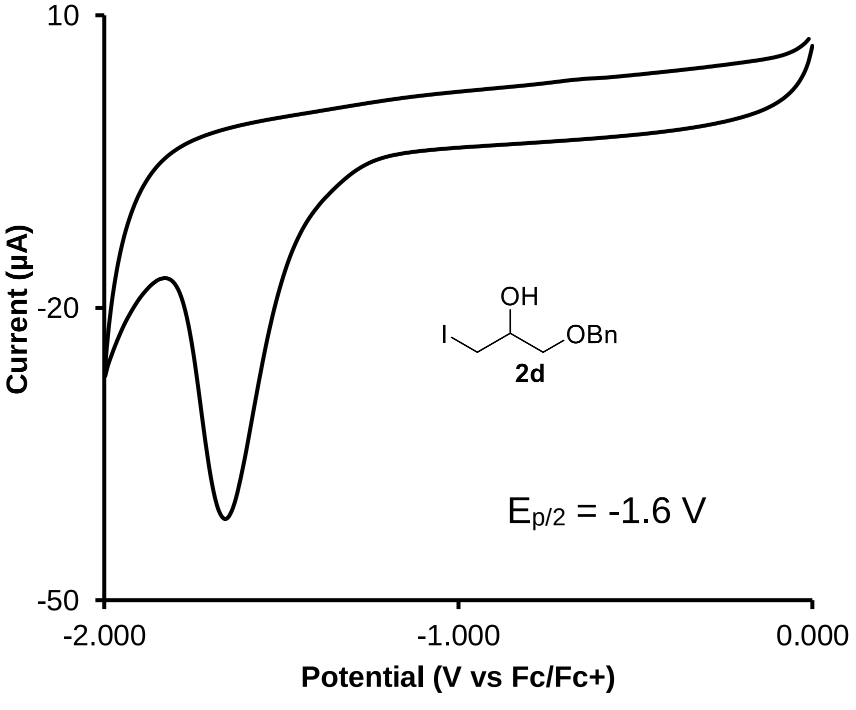

*Figure 16. Reductive Scan of the Alkyl halide (**2d**)*

4.4. Absorption Spectroscopy Analyses

Solutions at different concentrations of the BCRC were introduced into a 1 cm path length quarts' cuvette equipped with a Teflon® septum, under an argon atmosphere. All of the analysis were conducted using a UV-Vis cary60-TR0 spectrophotometer.

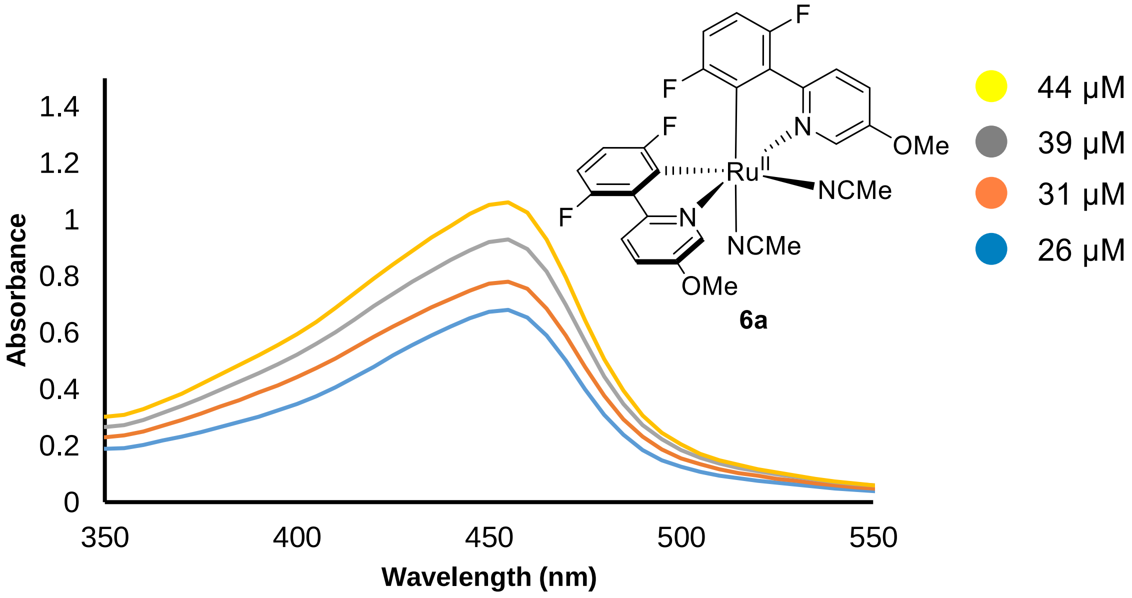

*Figure 17. UV-Vis absorption spectra for the BCRC **6a** recorded in MeCN.*

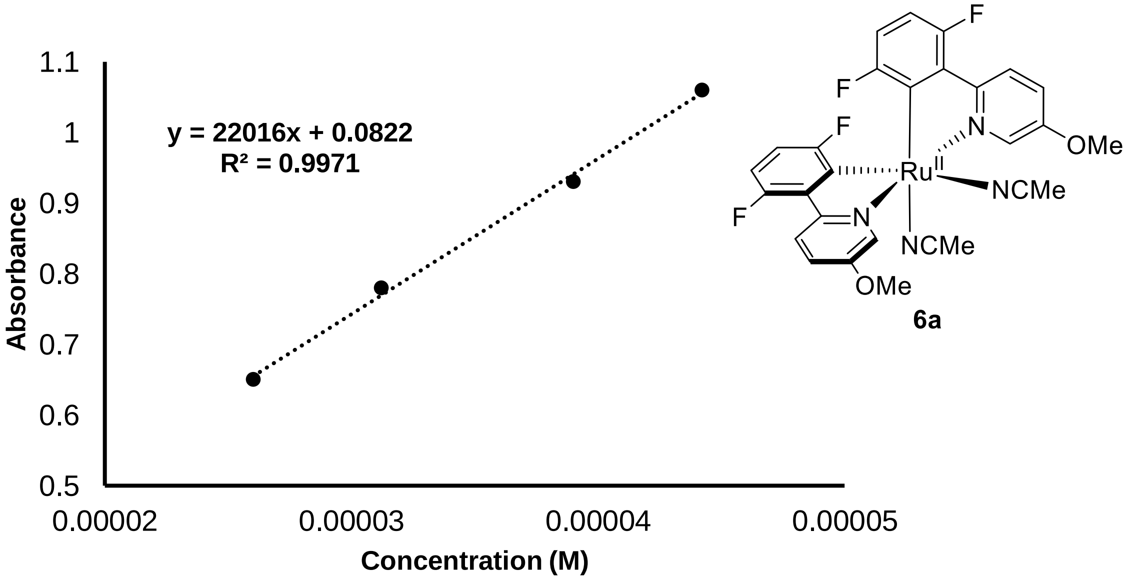

*Figure 18. Beer-Lambert plot for BCRC **6a***

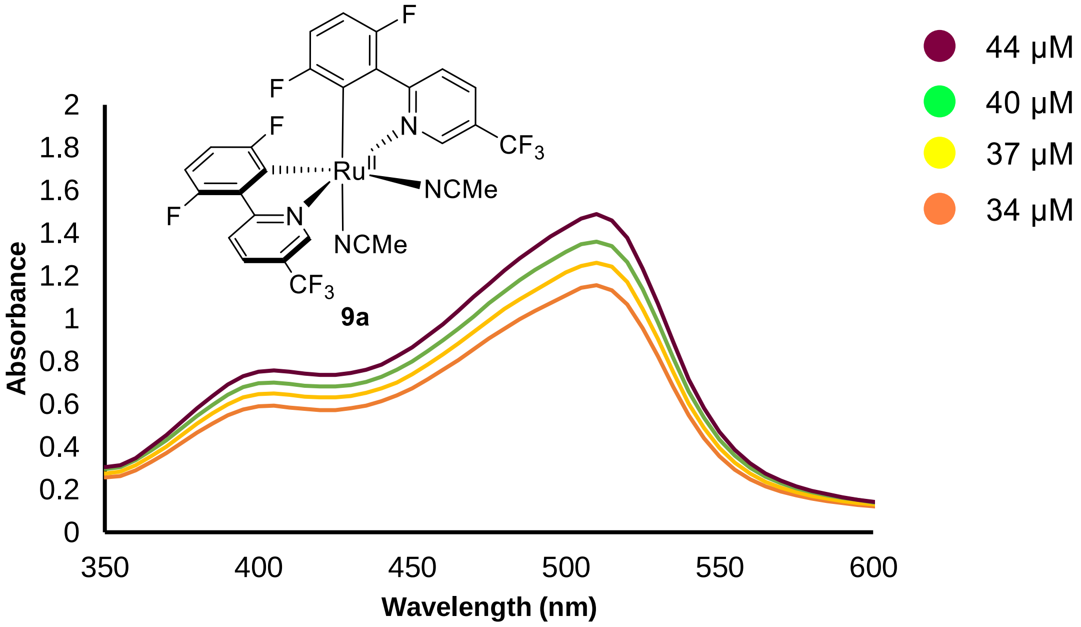

*Figure 19. UV-Vis absorption spectra for the BCRC **9a** recorded in MeCN.*

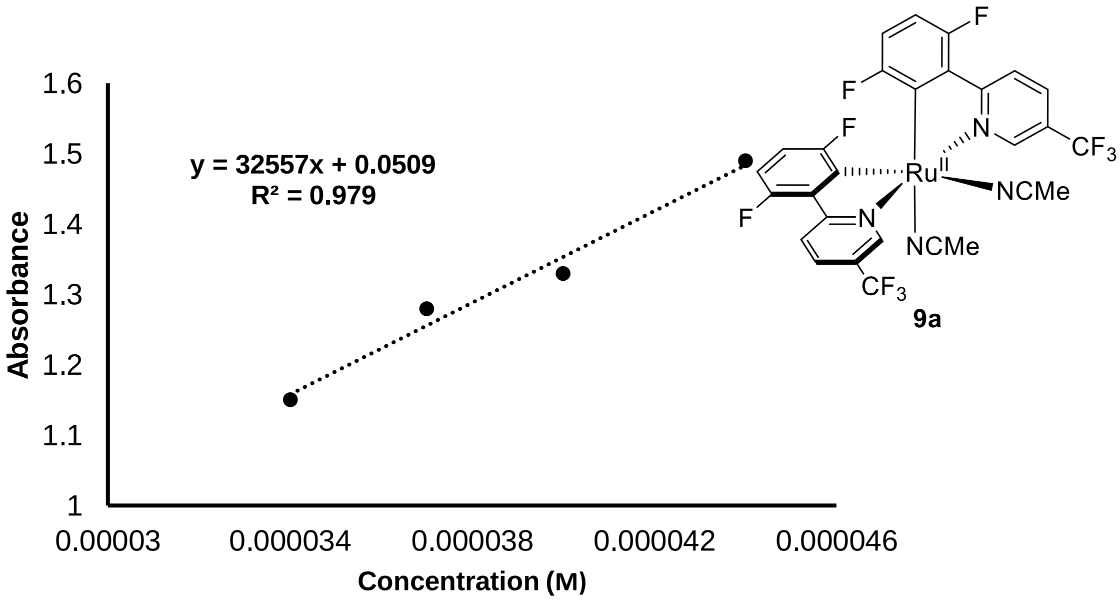

*Figure 20. Beer-Lambert plot for BCRC **9a***

When 2-phenylpyridine **1t**, which froms the BCRC **9a**, is irradiated under our reaction conditions at 440 nm, we do not observe any reactivity. As the UV-Vis absorption above shows, this BCRC species absorbs below 400 nm and above 500 nm. So, we attempted to irradiate this substrate at 365 nm and 525 nm, in order to attain a better overlap with the

absorption spectrum (Figure 19). Gratifyingly, we observe reactivity of 20% and 24% respectively at these wavelengths.

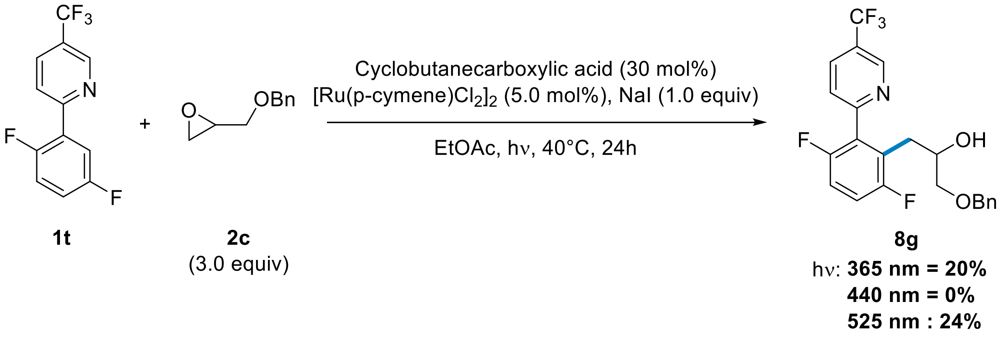

*Figure 21. Reactivity of substrate **1t** under our reaction conditions at 365nm, 440nm, 525nm.*

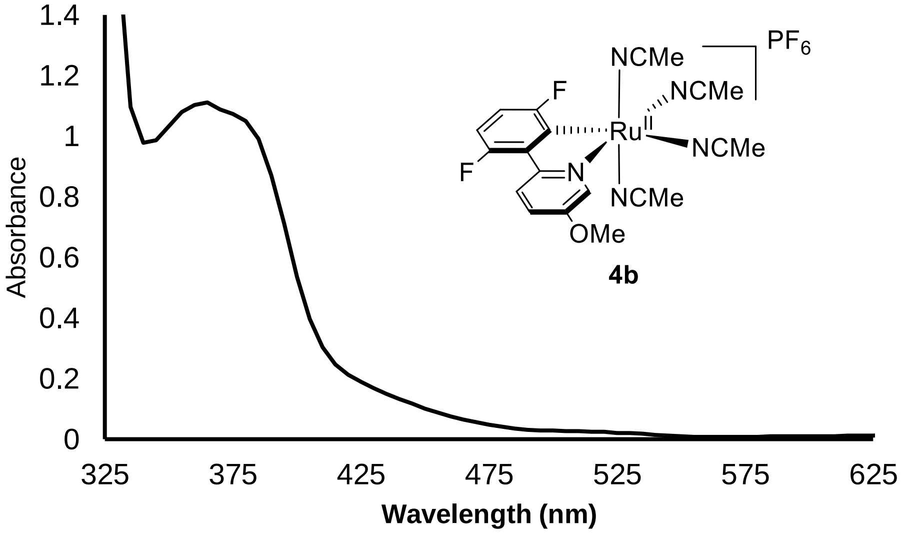

*Figure 22. UV-Vis absorption spectra for the OMe mono-cyclometallated species **4b** recorded in acetonitrile.*

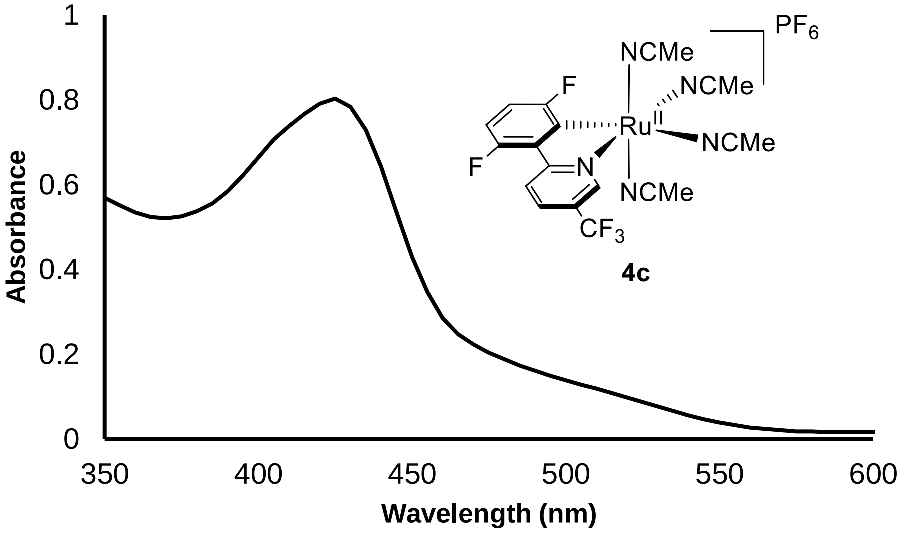

*Figure 23. UV-Vis absorption spectra for the CF_3 mono-cyclometallated species **4c** recorded in acetonitrile.*

4.5. Emission Spectra

Prior to measurements, the concentrations of the solutions were verified by UV-vis electronic absorption spectroscopy and were recorded on a Mettler Toledo UV5Bio spectrophotometer and were all below 0.5 absorption units to minimise inner filter effects and to maximise signal intensities. All emission and excitation data were recorded using an Edinburgh Instruments FLS-1000 photoluminescence spectrometer equipped with a 450 W steady state xenon lamp (with double 325 mm focal length excitation and emission monochromators in Czerny Turner configuration), interchangeable EPL pulsed diode lasers, a NKT SuperK Fianium FIU-6 fibre coupled supercontinuum laser, and a red-sensitive Hamamatsu PMT-900 detector. Room temperature samples were recorded using Youngs tap appended 1 cm path length quartz cuvettes and frozen solution samples using Youngs tap appended borosilicate NMR tubes inserted into an EPR finger dewar filled with liquid nitrogen. All spectra were corrected for the excitation and detector response to account for variations in the detector sensitivity across the visible spectrum and repeated at least three times to ensure reproducibility. All measurements were taken using appropriate long-pass filters to minimise scatter and second order effects and all emission spectra recorded over an excitation range of 300 – 700 nm to further rule out second order effects.

Emission Spectra of 6a

Anhydrous acetone, degassed by freeze pump thaw (4 cycles), was placed into a 10 x 10 mm light path quartz fluorescence cuvette equipped with a septum under an argon atmosphere. The concentration of BCRC in both cases was 52 μM The excitation wavelength was fixed at 460 nm while the emission was acquired from 550 nm to 900 nm. A solvent blank was subtracted from the measurement. An excitation spectrum was also recorded from 350 to 690 nm for the emission observed at 720 nm.

BCRC 6a Emission Spectra

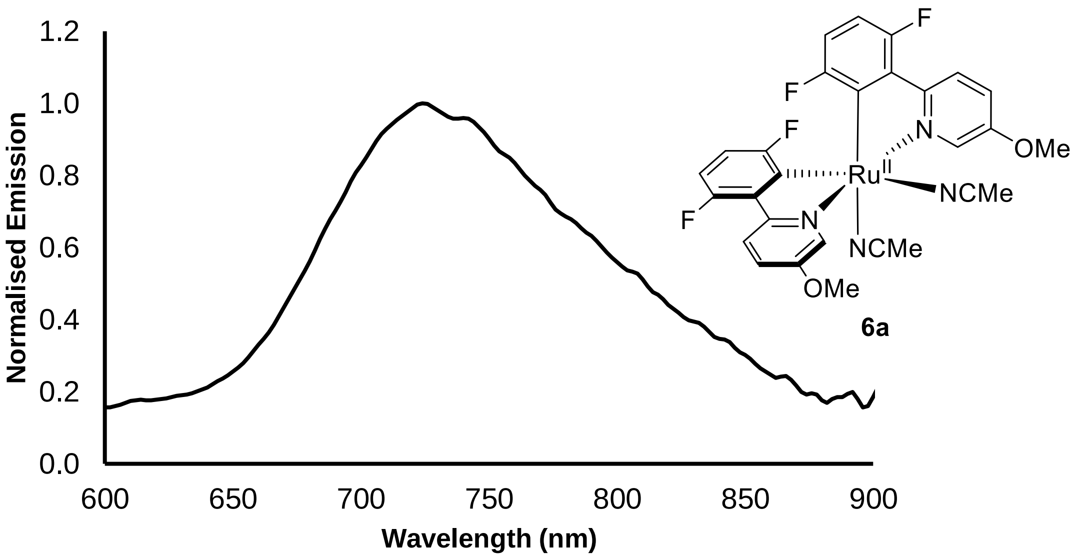

*Figure 24. Corrected emission spectra of BCRC **6a** at RT (excitation at 460 nm)*

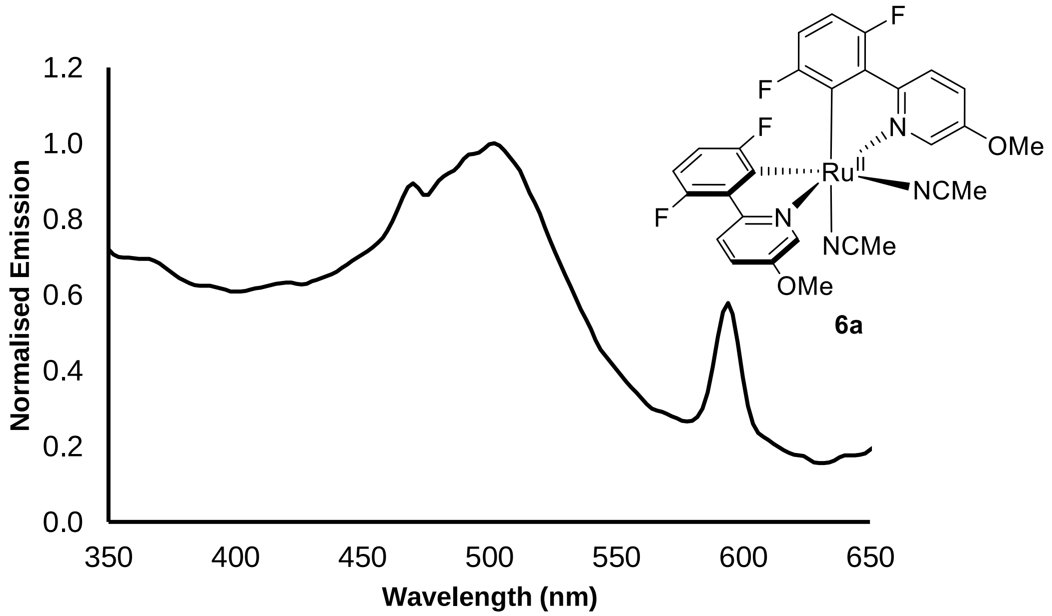

*Figure 25. Corrected excitation spectra of BCRC **6a** at RT (emission at 720 nm). *The feature at ca.595 nm is due to scattering effects from the sample.*

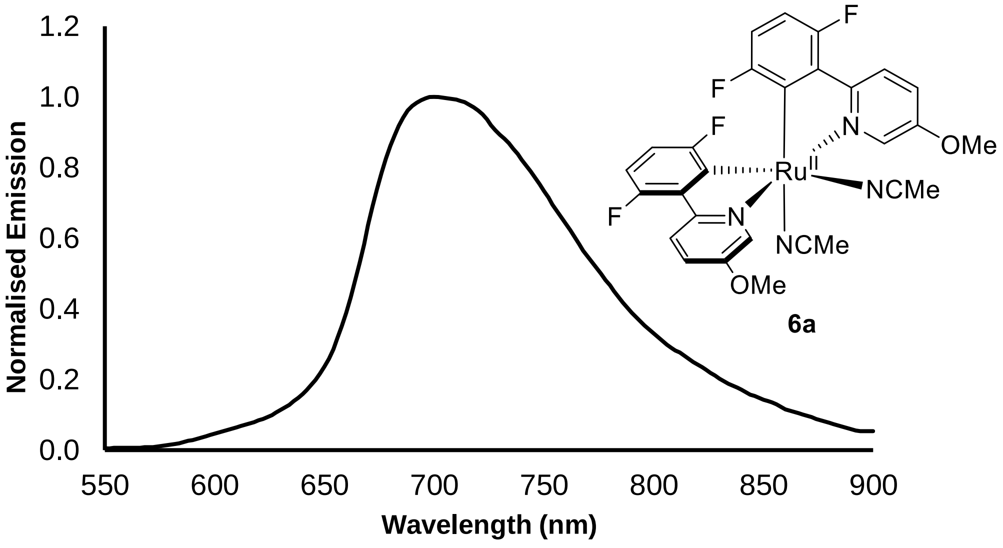

*Figure 26. Corrected emission spectra of BCRC **6a** at 77 K (excitation at 460 nm).*

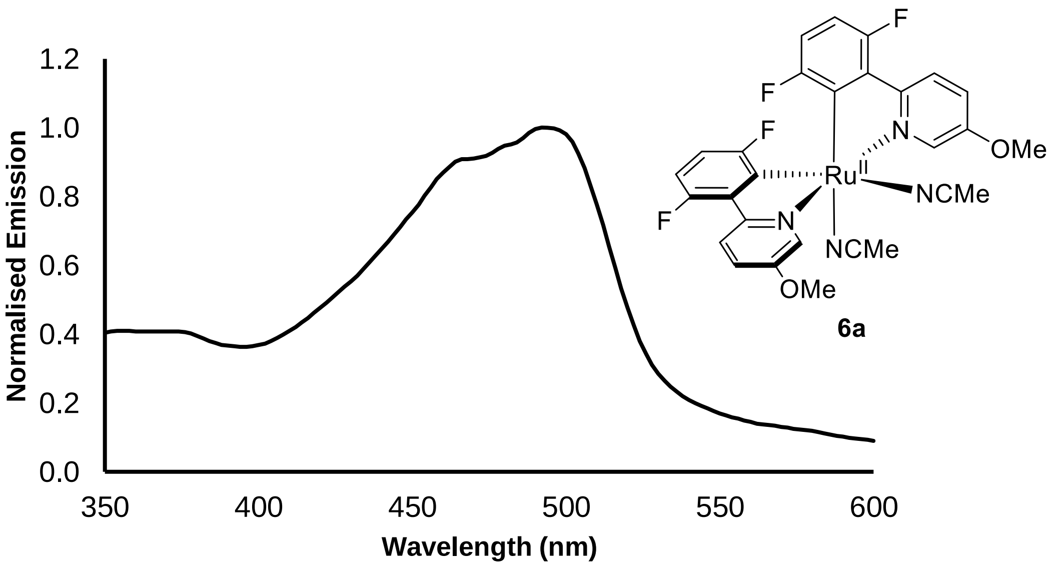

*Figure 27. Corrected excitation spectra of BCRC **6a** at 77 K (emission at 720 nm).*

Emission Spectra of 9a

Anhydrous acetone, degassed by freeze pump thaw (4 cycles), was placed into a 10 x 10 mm light path quartz fluorescence cuvette equipped with a septum under an argon atmosphere. The concentration of BCRC in both cases was 52 µM The excitation wavelength was fixed at 520 nm while the emission was acquired from 650 nm to 900 nm. A solvent blank was subtracted from the measurement. An excitation spectrum was also recorded from 350 to 700 nm for the emission observed at 878 nm.

BCRC 9a Emission Spectra

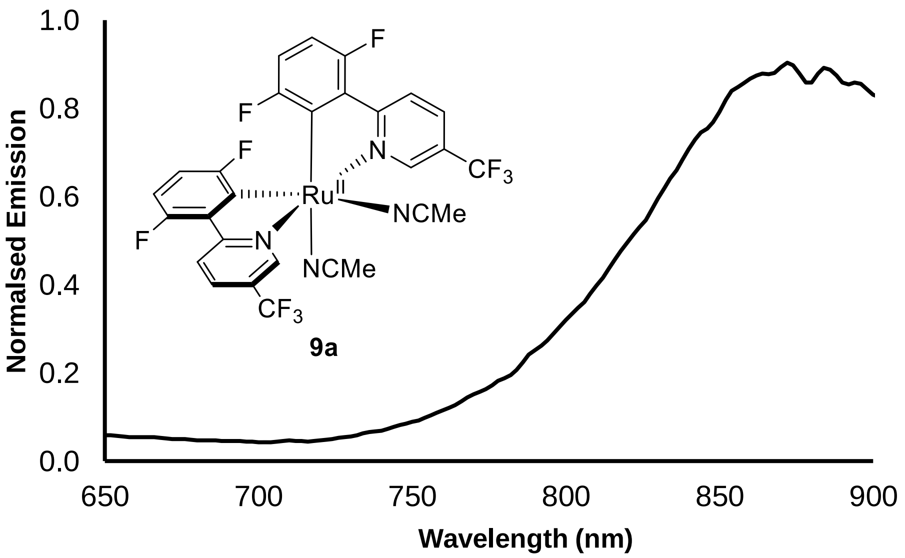

*Figure 28. Corrected emission spectrum of BCRC **9a** at RT (excitation at 520 nm)*

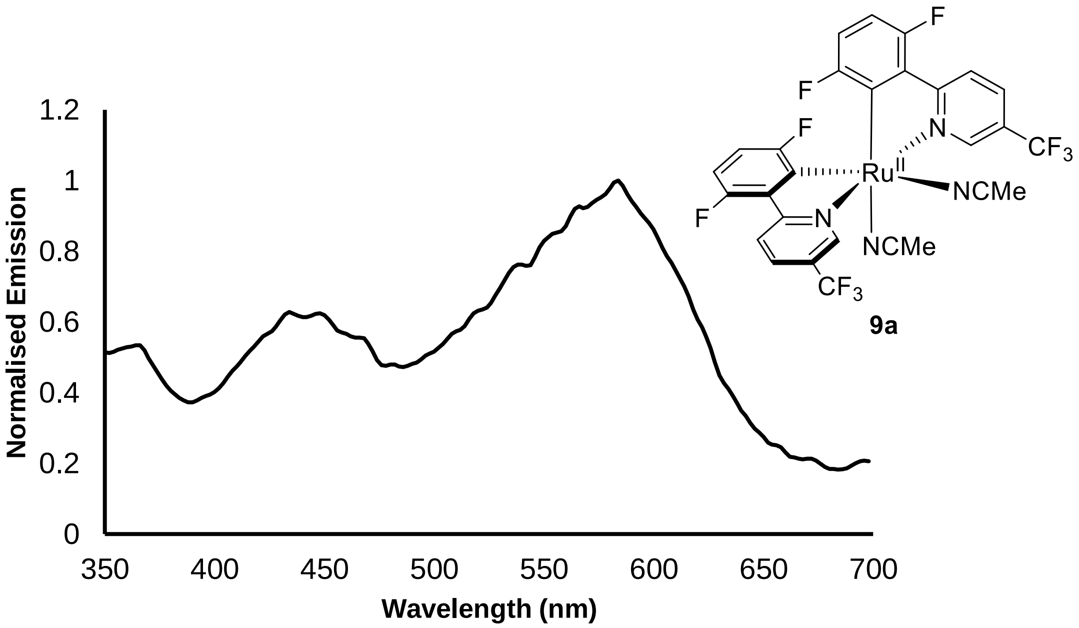

*Figure 29. Corrected excitation spectrum of BCRC **9a** at RT (emission at 878 nm)*

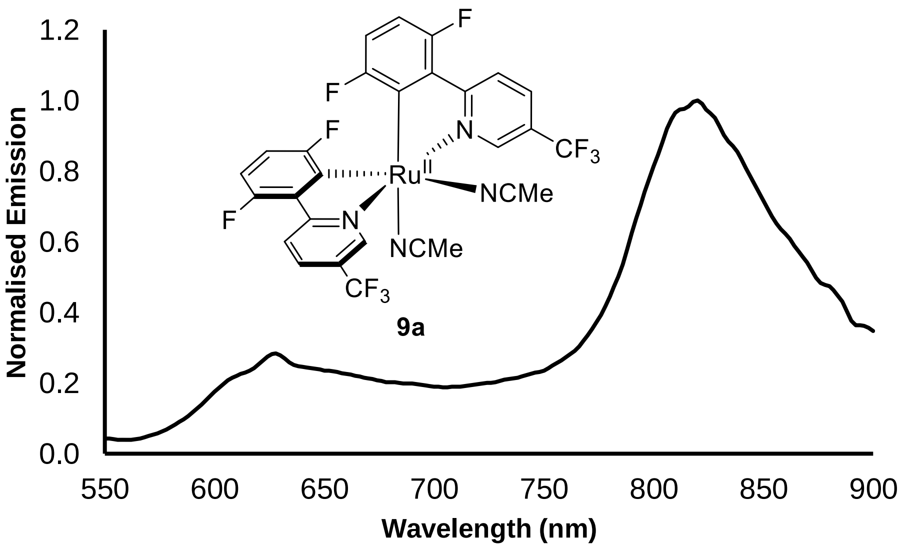

*Figure 30. Corrected emission spectrum of BCRC **9a** at 77 K (excitation at 530 nm)*

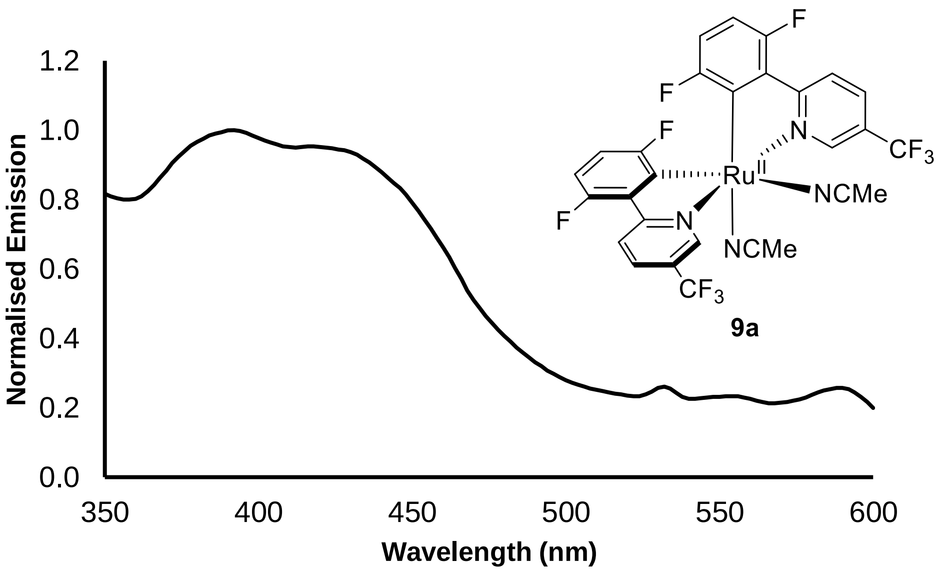

*Figure 31. Corrected excitation spectrum of BCRC **9a** at 77 K (emission at 630 nm)*

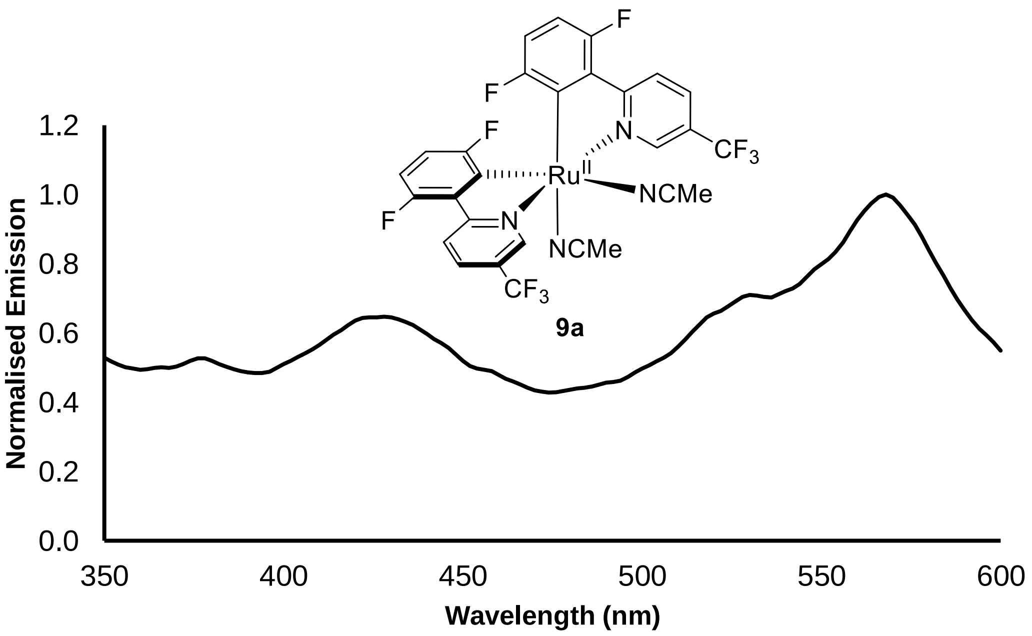

*Figure 32. Corrected excitation spectrum of BCRC **9a** at 77 K (emission at 820 nm)*

4.5.1 Stern-Volmer Quenching

Stern-Volmer Quenching of BCRC 6a

Anhydrous acetone, degassed by freeze pump thaw (4 cycles), was placed into a 10 x 10 mm light path quartz fluorescence cuvette equipped with a septum under an argon atmosphere**.** The concentration of BCRC was 52 µM. The excitation wavelength was fixed at 460 nm while the emission was acquired from 600 nm to 900 nm. A solvent blank was subtracted from the measurement. Separately, a solution of **2e** was prepared with a concentration of 15 mM. The addition of **2e** was repeated five times. After each addition, both an absorption and emission spectrum were recorded. No change in the absorption spectrum was observed.

Alongside this, another sample of BCRC was prepared with the same concentration as above. To this BCRC solution was added the same amount of acetone in the experiment above. An absorption and emission spectrum were recorded for each addition and this value was taken as I_0.

The results shown in figure 33 indicate that alkyl iodide **2e**, quenches the excited state of the BCRC and its emission. (Figure 33)

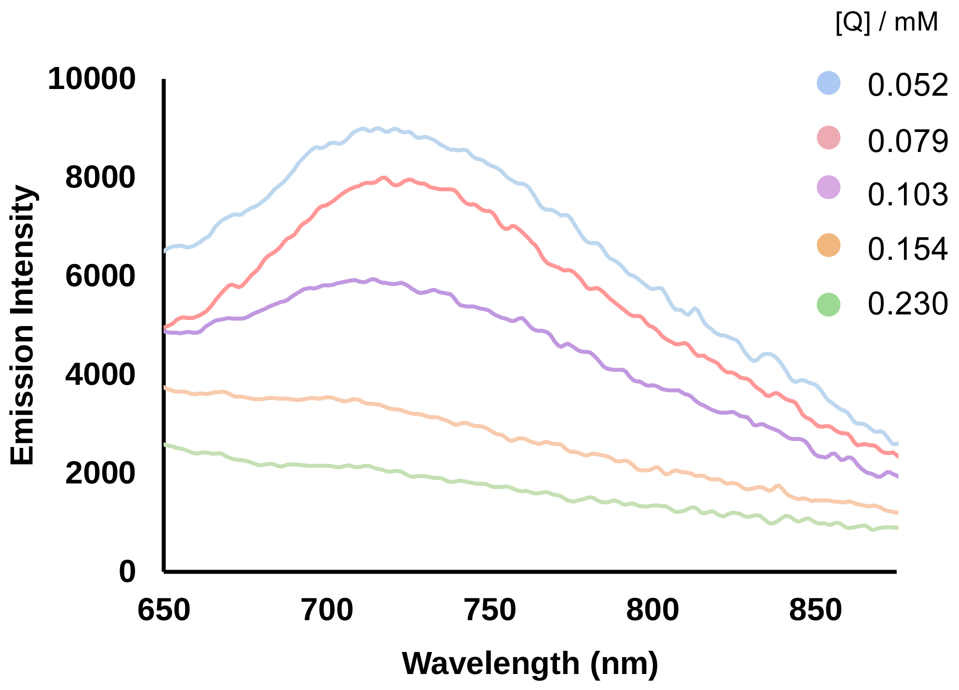

*Figure 33. Stern-Volmer Quenching of BCRC **6a***

The Stern-Volmer plot below shows a linear correlation between an increasing amount of **2e** and the ratio of I_0/I. Based on the below equation, it is possible to calculate the Stern-Volmer quenching constant. (Figure 34)

$$I_0/I = 1 + K_{SV}[Q]$$

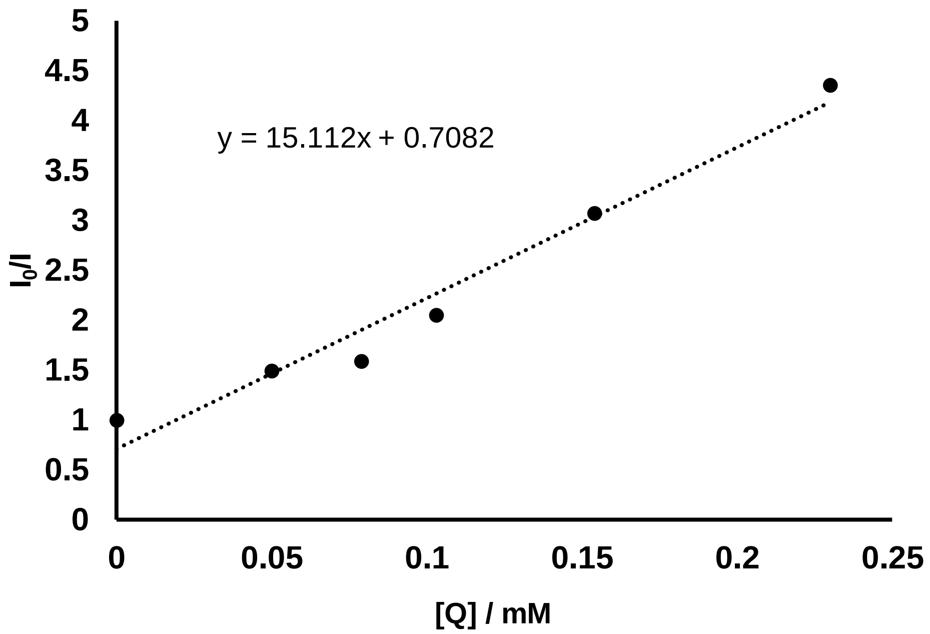

*Figure 34. Stern-Volmer Plot of BCRC **6a***

We calculated a Stern-Volmer quenching constant of 15 mM^{-1}. (Figure 34)

Below is the change in emission recorded due to dilution of the sample without quencher (Figure 35).

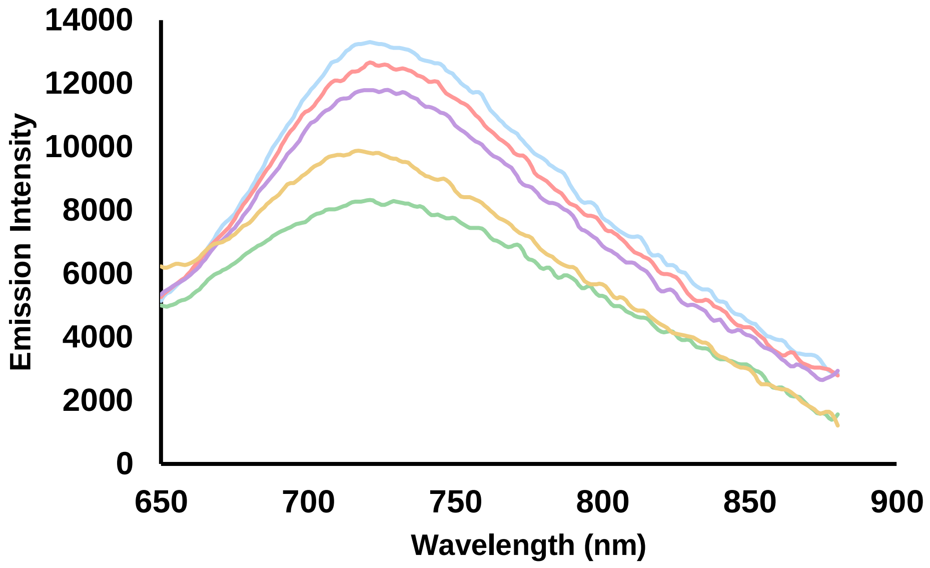

Figure 35. Dilution of the sample during the Stern-Volmer experiment without the quencher

From this value, we can calculate an estimate for the quenching fraction. It should be noted that this value uses information from room temperature and 77K measurements and therefore should only be taken as an estimate. We can calculate a bimolecular quenching constant, k_q using Eq.1…

$$K_{SV} = k_q \tau_0 \text{ [Eq. 1]}$$

Which gives a k_q value of 1.1 x 10^9 M^{-1}.

We can then estimate a value for the quenching fraction using Eq. 2 and the value of the lifetime recorded in section 4.7…

$$Q = \frac{kq(Q)}{\tau 0 + \mathrm{kq(Q)}} = \frac{1.1x10^{9}(0.052)}{10x10^{-6} + 1.1x10^{9}(0.052)} = 1$$

4.6. Evaluation of the Excited State Potential

Using the information gathered from the cyclic voltammetry (CV) studies (Section 4.3) and from the emission spectra (Section 4.5) of the BCRC complexes, we could estimate the redox potential relative to their excited state via the following equation 2:

$$E(\frac{\boldsymbol{BCRC}(II)*}{\boldsymbol{BCRC}(II)}) = E(\frac{\boldsymbol{BCRC}(III)}{\boldsymbol{BCRC}(\boldsymbol{II})}) - E_{0\text{-}0}(\frac{\boldsymbol{BCRC}(II)*}{\boldsymbol{BCRC}(II)}) \; [Eq.\ 2]$$

4.6.1. Evaluation of the Excited State Potential of Complex 6a

Since the electrochemical oxidation of **6a** was reversible (Figure 10), the reversible peak potential *E*p was used for *E*(Ru(III)/Ru(II)). E_{0-0}(Ru(II)*/Ru(II)) was estimated spectroscopically using the 10% rule, where the value for E_{0-0} is taken at 10% of the emission intensity. This method of estimating the excited state potential is consistent with previously reported methods. [3] For **6a**, the E_p, corresponding to the *E*(Ru(III)/Ru(II)) is -0.30 V (Figure 10). While 10 % of the emission intensity corresponds to 648 nm and a $E_{0\text{-}0}$(Ru(II)*/Ru(II)) value of 1.91 V.

$$E(\frac{Ru(II)*}{Ru(III)}) = -0.30 - 1.91 = -2.21 \text{ V (Vs Fc/Fc+)}$$

4.6.2. Evaluation of the Excited State Potential of Complex 9a

Since the electrochemical oxidation of **9a** was reversible (Figure 11), the reversible peak potential E_p was used for E(Ru(III)/Ru(II)). Therefore, E_{0-0}(Ru(II)*/Ru(II)) was estimated spectroscopically using the 10% rule, where the value for E_{0-0} is taken at 10% of the emission intensity. For **9a**, the E_p, corresponding to the E(Ru(III)/Ru(II)) is -0.12 V (Figure 11). While 10 % of the emission intensity corresponds to 769 nm, corresponding to an E_{0-0}(Ru(II)*/Ru(II)) of 1.61 V.

$$E\left(\frac{Ru(II)*}{Ru(III)}\right) = -0.12 - 1.61 = -1.73 \text{ V (Vs Fc/Fc+)}$$

4.7. Lifetime Studies

Lifetime data were recorded following excitation with a 60 W microsecond flashlamp, a EPL 405 nm diode laser or a NKT SuperK Fianium FIU-6 supercontinuum laser using multi-channel scaling (MCS, μs lifetimes) or time correlated single photon counting (TCSPC, ns lifetimes). Lifetimes were obtained by either tail fitting or reconvolution fitting using the instrumental response function (TCPSC) on the data obtained, and the quality of fit judged by minimization of reduced χ-squared and residuals squared. In cases where the microsecond flash lamp was used, care was taken to account for the instrument response function and lifetimes were fitted after this had decayed to negligible intensity by tail fitting (approximately after 25 μs).

Anhydrous acetonitrile, degassed by freeze pump thaw (4 cycles), was placed into a J-Young NMR tube under an argon atmosphere**.** The concentration of BCRC was 52 μM. A solvent blank was subtracted from the measurement. Lifetime experiments were performed at both room temperature and 77 K for BCRC **9a** and at 77 K only for BCRC **6a** as the emission signal at room temperature was either too weak and or short-lived to be measured.

Table 10. **Photophysical data for the complexes recorded in MeCN**

Complex	Temperature	λ_{exc} (nm)	λ_{em} (nm)	τ_1 (ns), (%)	τ_2 (ns), (%)	χ^2
**BCRC 6a**[a]	77 K	360	540	13,563 (80)	67,523 (20)	1.1
**BCRC 6a**[a]	77 K	430	612	10,060	-	1.5
**BCRC 9a**[b]	289 K	520	775	3.1	-	1.2
**BCRC 9a**[c]	77 K	405	785	531	-	1.1

[a] Recorded using a 60 W microsecond pulsed flashlamp operating at 100 Hz, [b] recorded using a NKT supercontinuum laser operating at 26 MHz, [b] recorded using the EPL-405 nm pulsed diode laser operating at 200 kHz.

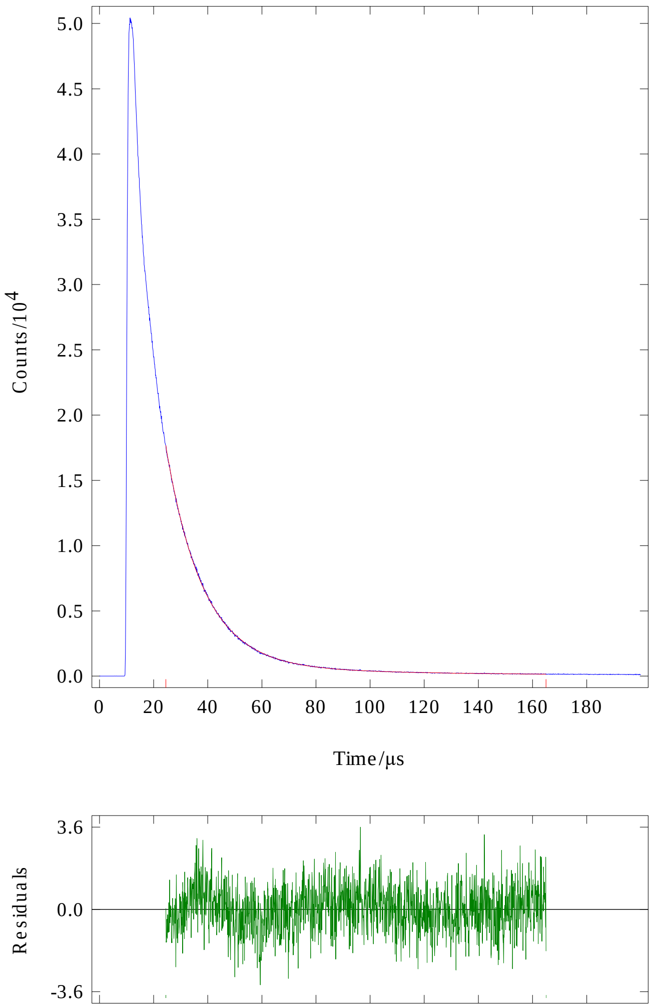

*Figure 36. Fitted kinetic trace of BCRC **6a** at 77K in MeCN (excitation at 360 nm, emission at 540 nm)*

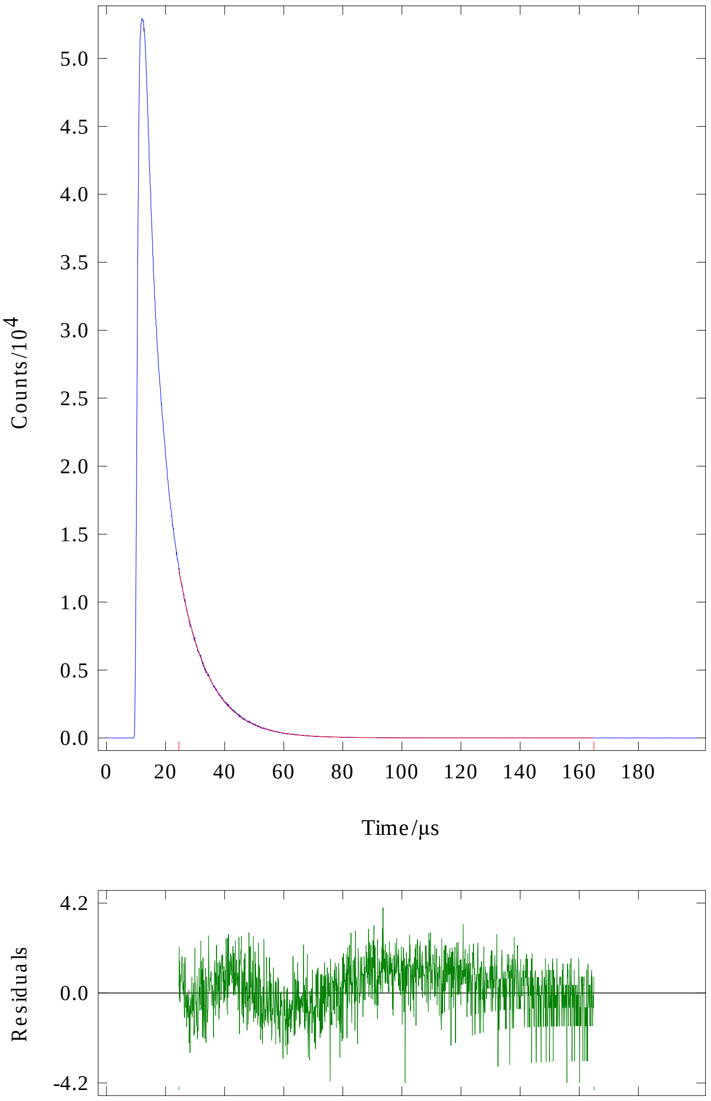

*Figure 37. Fitted kinetic trace of BCRC **6a** at 77K in MeCN (excitation at 430 nm, emission at 612 nm)*

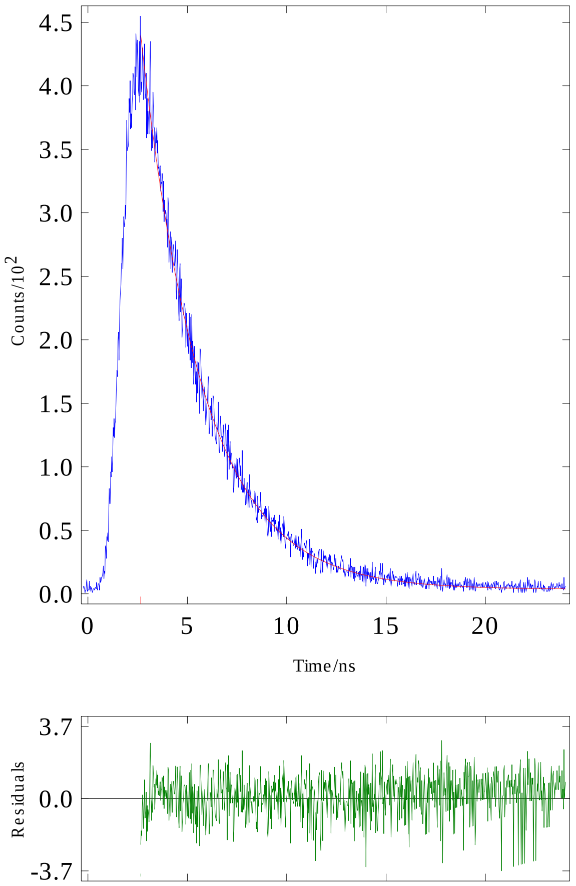

Figure 38. Fitted kinetic trace of BCRC ***9a*** *at 298 K in MeCN (excitation at 520 nm, emission at 785 nm)*

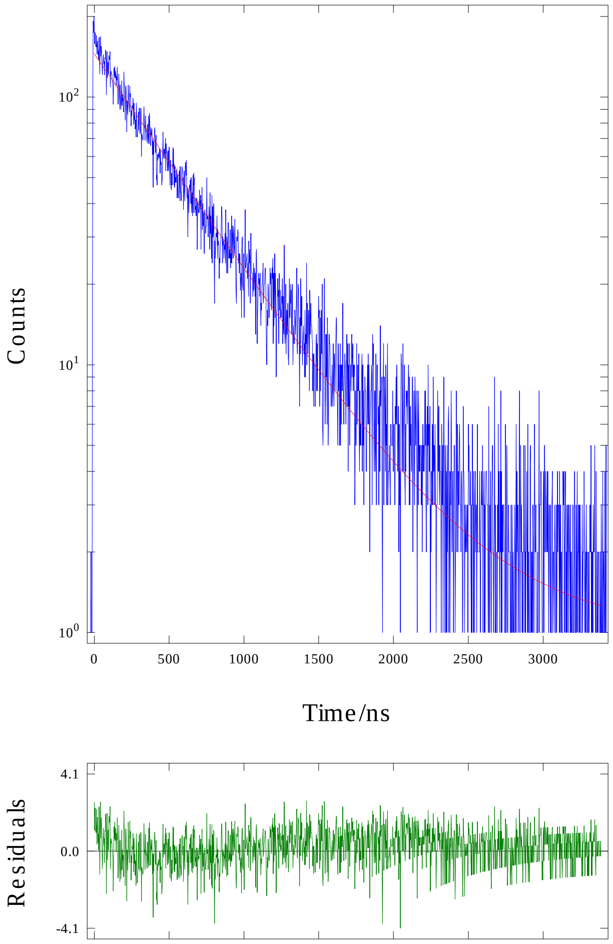

*Figure 39. Fitted kinetic trace of BCRC **9a** at 77 K in MeCN (excitation at 405 nm, emission at 775 nm)*

4.8. Quantum Yield Determination

Determination of the photon flux at 440 nm: The photon flux of the 440 nm Blue LED used in these studies was determined as previously reported.[4] A 0.15 M solution of potassium ferrioxalate was prepared by dissolving 2.21 g of potassium ferrioxalate hydrate in 30 mL H_2SO_4 (0.05 M). A buffered solution of phenanthroline was prepared by dissolving 50 mg of phenanthroline and 11.25 g sodium acetate in 50 mL H_2SO_4 (0.5 M). Next, a microwave vial was completely covered in blue tape except for a small 1 cm^2 hole near the base. The prepared tube was charged with 5 mL of the ferrioxalate solution and irradiated for 10 s. This solution was treated with 0.875 mL phenanthroline solution and stirred for 1 h in the dark. The absorbance of the solution was measured at 510 nm. A non-irradiated sample was also prepared, and the absorbance measured at 510 nm. Finally, the conversion of the ferrioxalate was calculated:

$$Mol\ Fe^{2+} = V\left(\frac{\Delta A}{l\ x\ \varepsilon}\right) = 0.005875\ x\ \left(\frac{0.285-0.11}{1\ x\ 11100}\right) = 9.26x10^{-8}\ mol$$

Where V is the total volume of the solution after addition of phenanthroline, *ΔA* is the difference in absorbance at 510 nm between the irradiated and non-irradiated solution, l is the path length and ε is the molar absorptivity.

The photon flux was calculated as follows:

$$Photon\ Flux = \frac{mol\ Fe2+}{\phi\ x\ t\ x\ f} = \frac{9.26x10-8}{1.01\ x\ 10\ x\ 0.44} = 2.08x10^{--8}\ cm^{--2}s^{--1}$$

Where ϕ is the quantum yield for the ferrioxalate actinometer, t is the time and f is the fraction of light absorbed at 440 nm.

Determination of the quantum yield: In order to calculate the f value for the subsequent BCRC complex **6a** that forms, a UV-Vis was recorded using the same concentration of **BCRC** found in the reaction mixture. The absorption recorded was much greater than 3, indicating an f value of ~1.

$$f = 1 - 10^{-A} = 1 - 10^{-3} = 0.999$$

The standard reaction was performed in a microwave vial wrapped completely in blue tape except for a small hole near the base with an area of 1 cm^2. The sample was irradiated and the yield of the reaction was recorded over the course of 15 hours. (Figure 40)

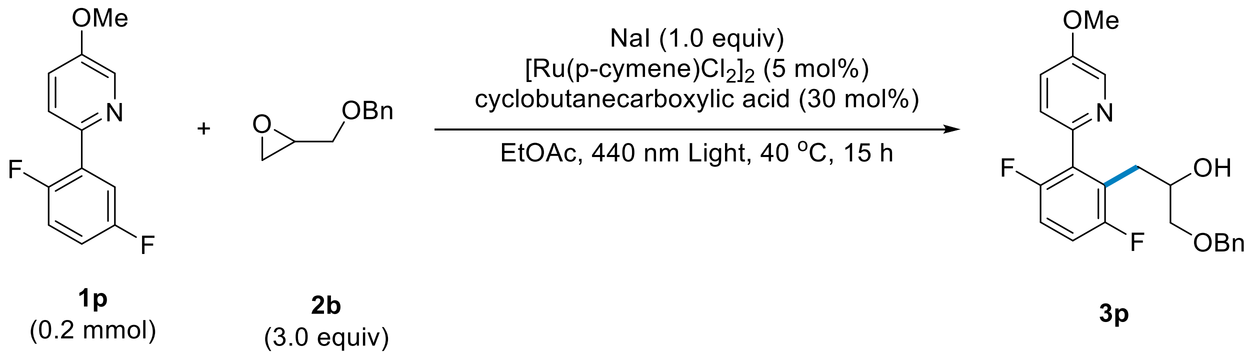

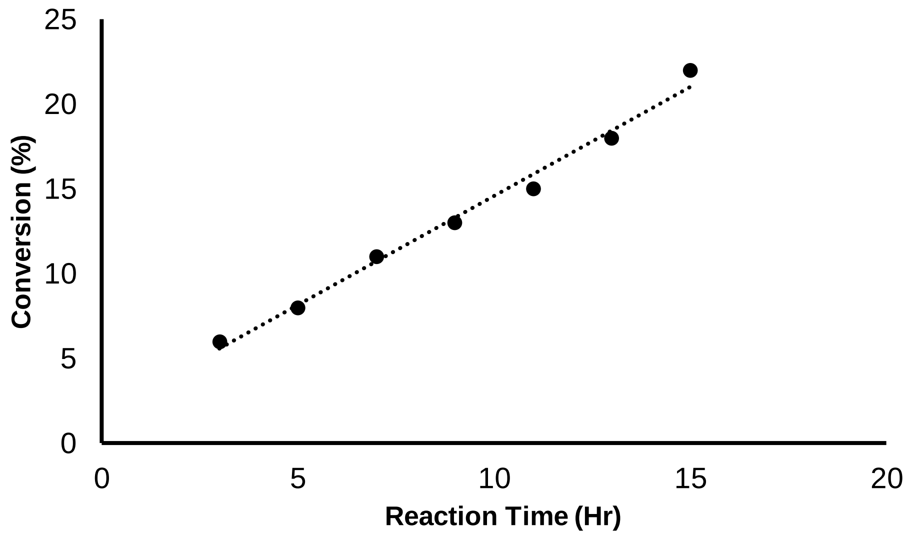

Figure 40. Product yield over the course of the first 15 hours. Reaction yields were recorded via ^{1}H NMR spectroscopy using trimethoxybenzene as an internal standard. Each point corresponds to a seperate reaction.

The quantum yield was determined using the following equation:

$$\Phi = \frac{mol\ P}{flux\ x\ t\ x\ f}$$

Which gave a consistent value of 0.04 for the quantum yield of this transformation. (Figure 41)

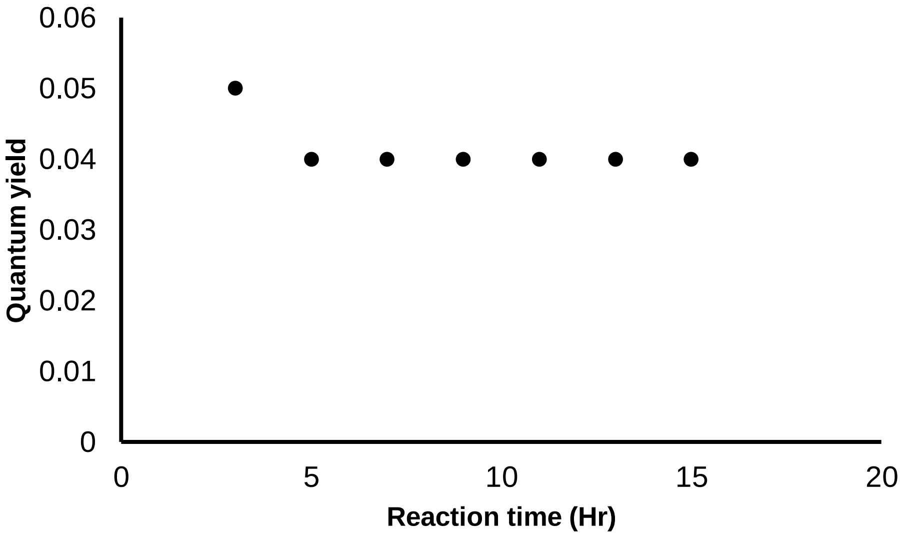

Figure 41. Quantum yield over the course of the first 15 hours.

Additionally, we have recorded the quantum yield of substrate **3o** which has the fluorine para to the pyridine and gave a yield of 79% for the product. The standard reaction was performed in a microwave vial wrapped completely in blue tape except for a small hole near the base with an area of 1 cm^2. The sample was irradiated and the yield of the reaction was recorded over the course of 24 hours.

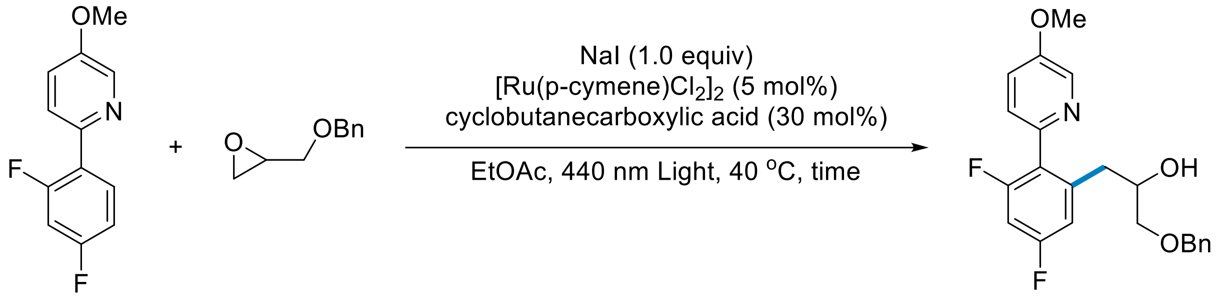

1o (0.2 mmol) **2b** (3.0 equiv) **3o**

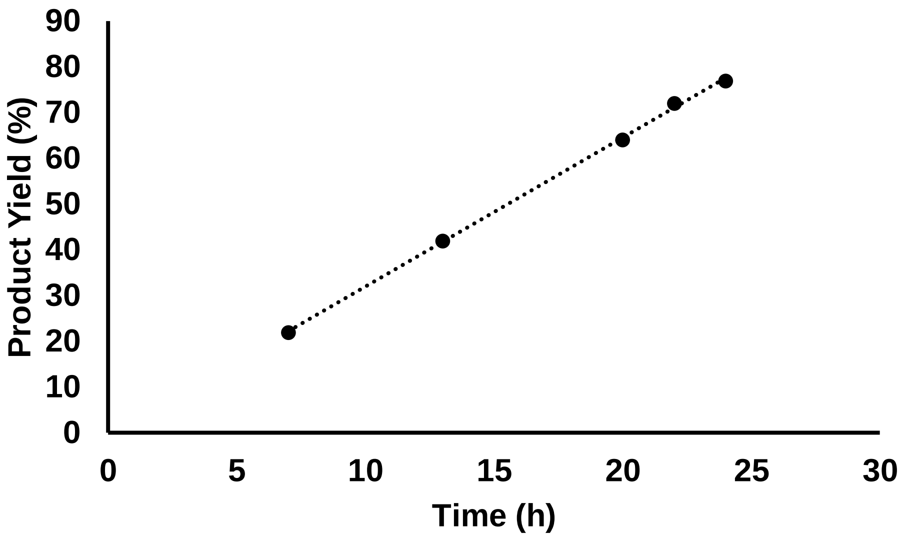

Figure 42. Product yield over the course of the first 24 hours. Reaction yields were recorded via ^{1}H NMR spectroscopy using trimethoxybenzene as an internal standard. Each point corresponds to a separate reaction.

The quantum yield was determined using the following equation:

$$\Phi = \frac{mol\ P}{flux\ x\ t\ x\ f}$$

Which gave an average value of 0.86 for the quantum yield of this transformation. (Figure 43)

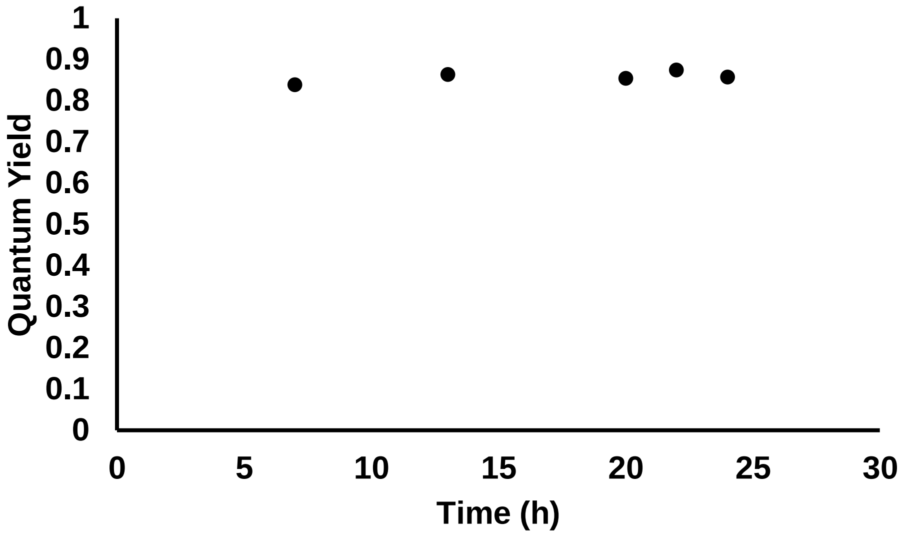

Figure 43. Quantum yield over the course of the first 24 hours for ***3o***

4.9. Unsuccessful Substrates

During our studies, multiple substrates were not suitable for our reaction conditions and did not furnish product. (Figure 44)

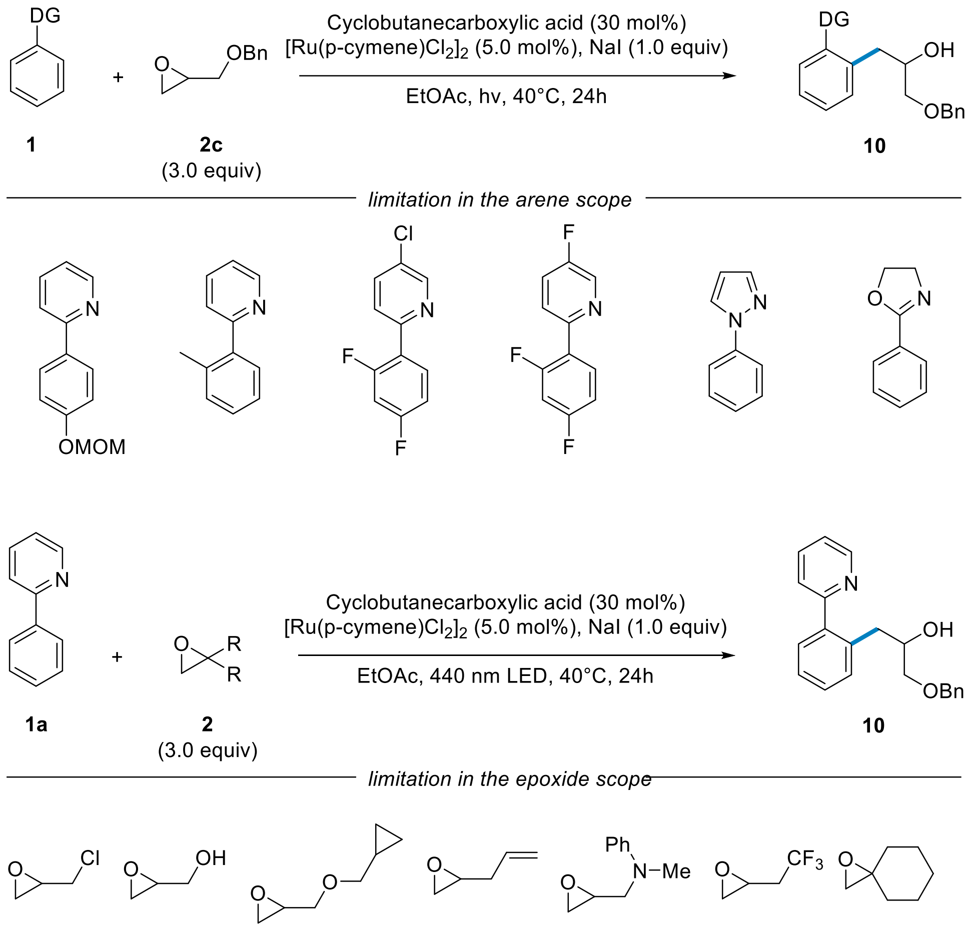

Figure 44. Unsuccessful substrate scope

Attempts were made to extend this protocol to oxetanes, however we were unable to form product from the oxetane analogue of epoxide **2c**. (Figure 45)

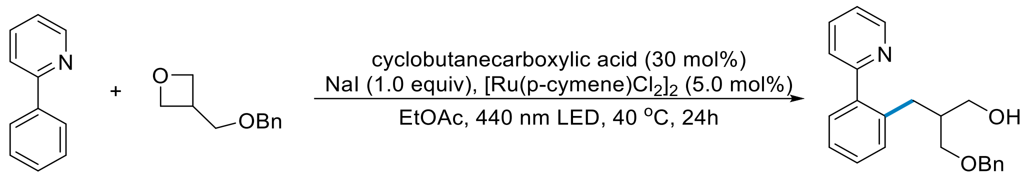

1a **2i** (3.0 equiv) 0%

Figure 45. Attempt at extending the protocol to oxetanes

We also attempted to use phenyloxirane, but got only trace amounts of ortho product. We were unable to separate this from the meta product. (Figure 46)

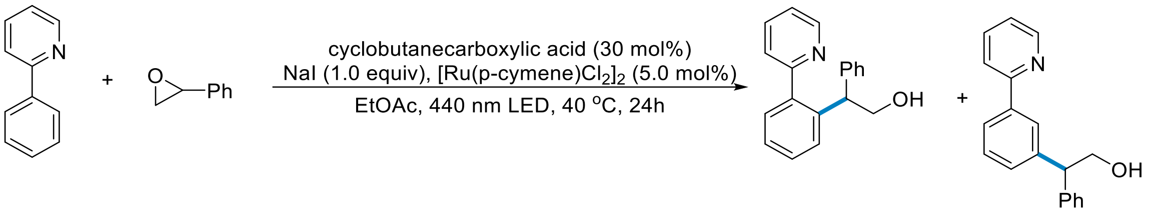

1a **2j** (3.0 equiv) **7ka** 4% **7kb** 34%

Figure 46. Attempt at extending the protocol to phenyloxirane

4.10. Static Vs Dynamic Quenching

To determine whether a complex is formed as a result of mixing a BCRC species with an increasing amount of the alkyl halide **2e**, a UV experiment was performed. Shown below are the absorption spectra of the BCRC species **6a** after the addition of the alkyl iodide **2e**. No change is observed to the absorption spectra, therefore, no new complex is formed as a result of mixing these species together. (Figure 47)

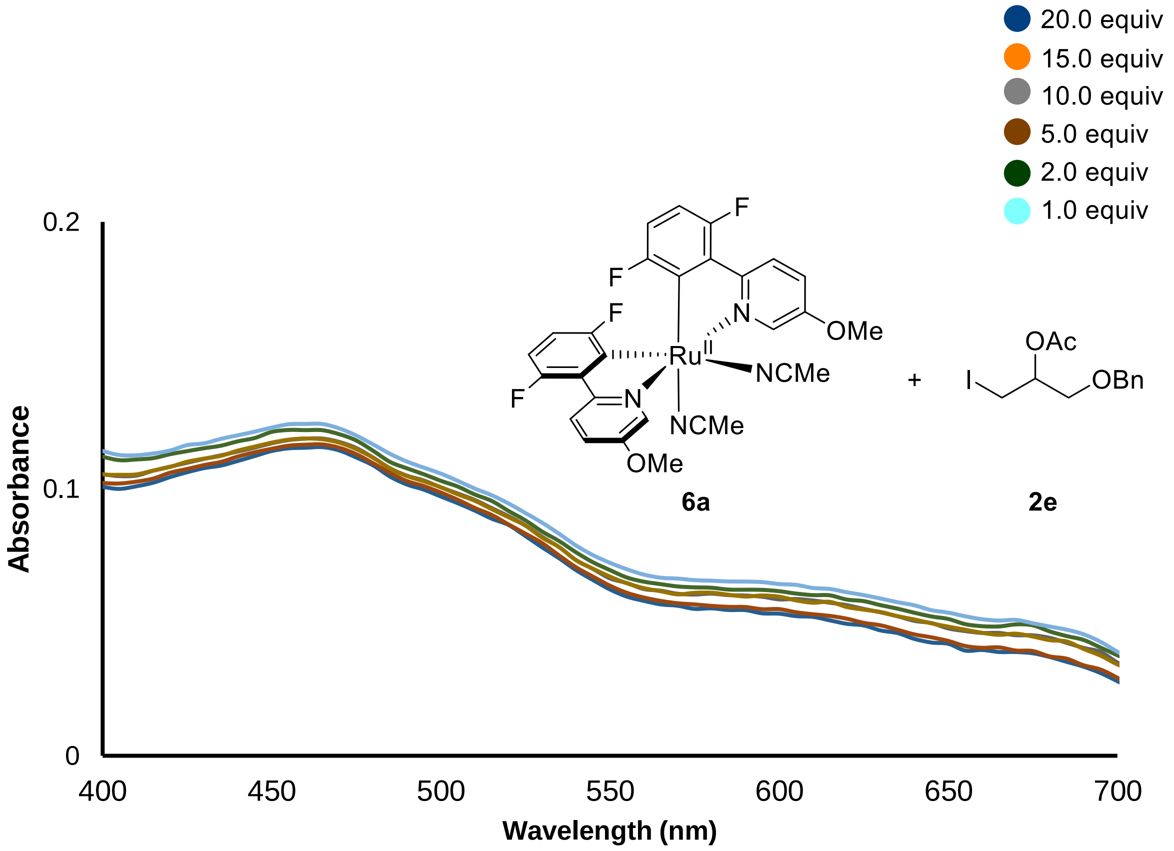

*Figure 47. UV – absorption spectra of the BCRC **6a** and the alkyl iodide **2e** in benzene*

4.11. Mechanistic Investigation into Inhibitory Effect of the Hydroxyl

Previous experimentation has shown epoxide opening (eg to alkyl iodide **2d**) must occur before the reaction can proceed. Furthermore, a BCRC (biscyclometallated ruthenium complex, eg **6a**) has been shown to be the on-cycle active catalytic species. When we monitored the stochiometric reaction of BCRC **6a** with alkyl iodide **2d** (Scheme 48a) by ^{1}H NMR (Scheme 48b) we observed consumption of both **6a** and **2d**. However, instead of forming the product of alkylation, we observed the formation of deiodinated ketone **2k**, protodemetallated arylpyridine (**1p**) and a monocyclometallated ruthenium complex that we tentatively assigned as the the iodide bound **Ru-I**. Remarkably, over 80% of the alkyl iodide was converted to ketone in just over two hours at room temperature. Observation of the same ketone under the catalytic reaction conditions can be attributed to this unproductive background H-I elimination reaction occurring alongside the productive photochemical pathway (*vide infra*).

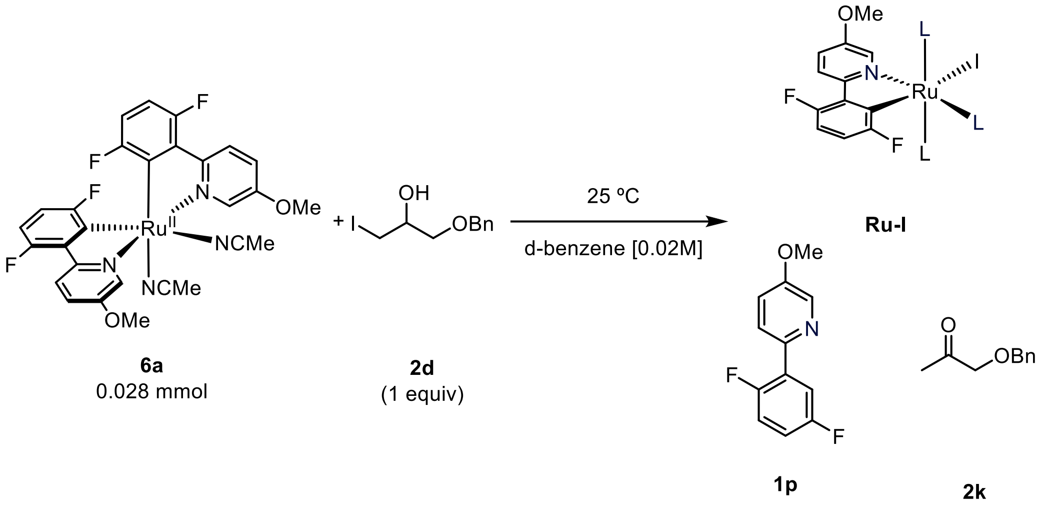

Figure 48a. Stoichiometric reaction between alkyl iodide and BCRC

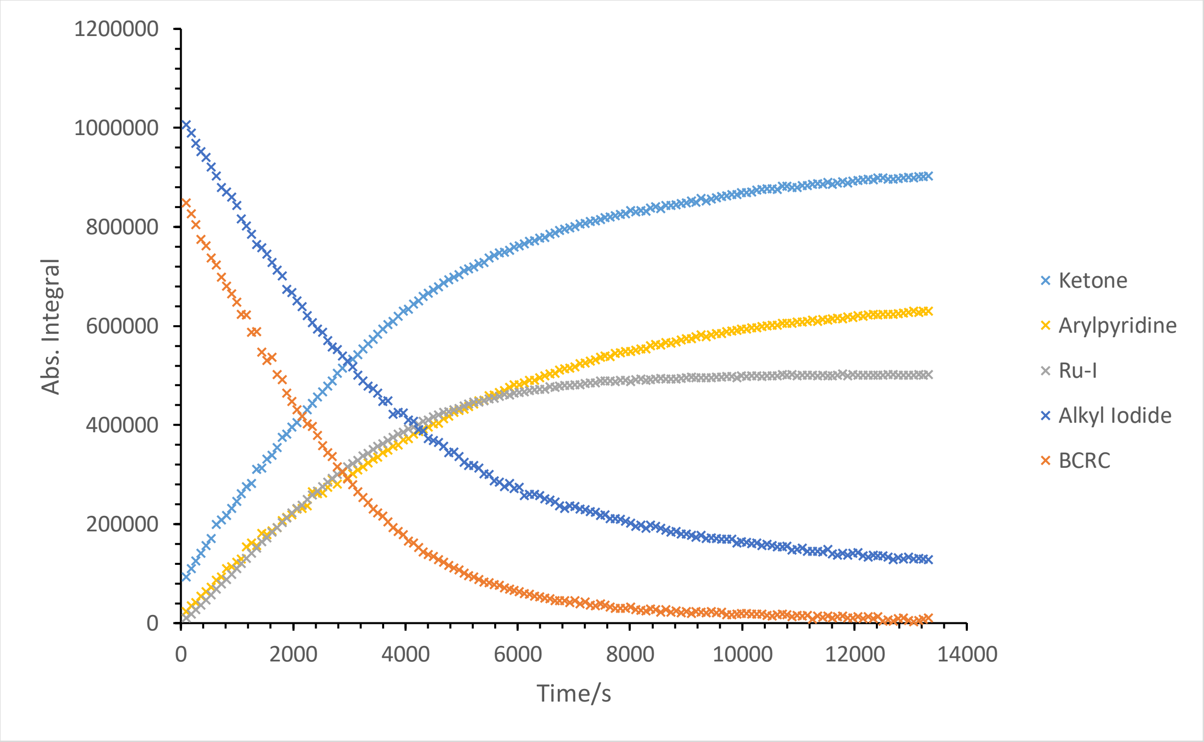

Figure 48b. *Time course of the reaction shown in figure 48a.*

When then studied the same reaction under photochemical conditions using the NMR Torch developed by Dr. Alexander Golovanov which allowed for *in-situ* NMR tracking of the photochemical reaction.[1] This set up allows us to control the exact temperature of the reaction in the spectrometer, which we set at 15 ºC to slow the rate of the undesired thermal elimination reaction. The first 44 min of the reaction was observed in the dark to establish a baseline rate before starting illumination. The time course shows that only the undesired side elimination reaction proceeds before illumination, indicating the inaccessibility of the single electron transfer (SET) under thermal conditions. The 460 nm light was switched on at 44 min and maintained for the remainder of the observation. Under illumination, rapid consumption of the BCRC **6a** combined with concurrent product formation is observed, while the rate of the background elimination remains largely unaffected (Scheme 48d). These results clearly demonstrate that the formal oxidative addition of **2d** on **6a** is induced by light irradiation.

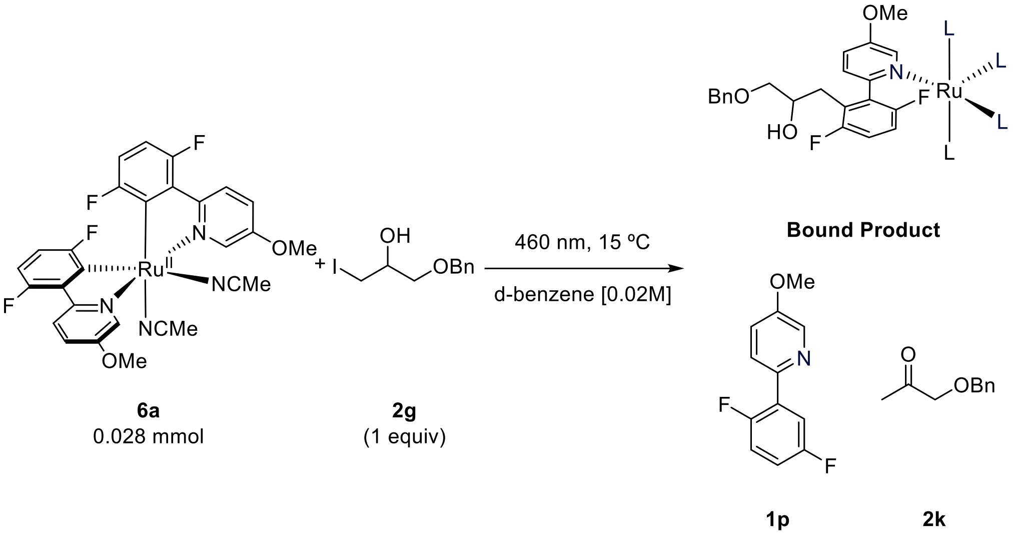

Figure 48c. Stoichiometric reaction of alkyl iodide and BCRC under in-situ illumination conditions.

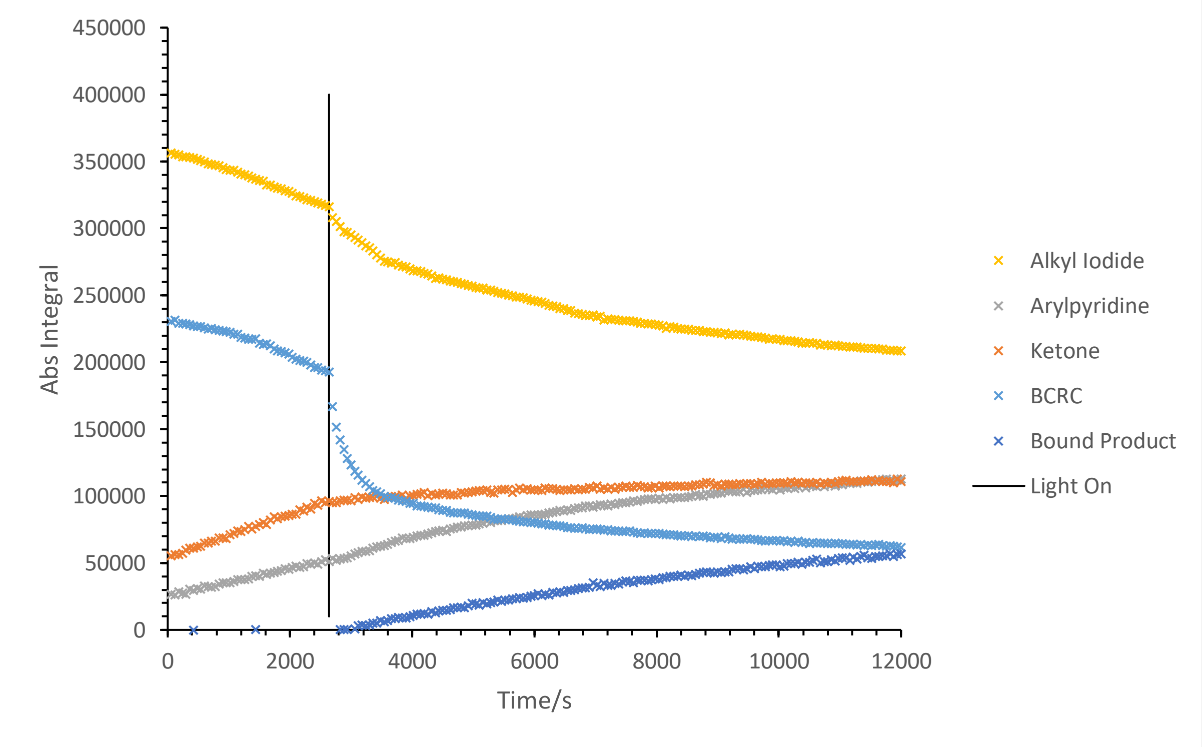

Figure 48d. *Time course of the reaction shown in figure 48c.*

Considering these results, we propose the following rational for the requirement of light in this reaction. Under thermal conditions, elimination pathway **A** is significantly more accessible, resulting in a rapid elimination with the Ru–C bond acting as a base in a concerted 6-

membered transition state (Scheme 48e). When compared with simple alkyl iodides, the presence of a hydroxyl group lowers the overall energy of this pathway due to the formation of a ketone with increased thermodynamic stability when compared with an alkene (BDE = 146 kJ mol $^{-1}$ C=C vs 192 kJ mol $^{-1}$ C=O). Conversely, under photochemical conditions, pathway **B** then becomes operative. As SET occurs from an excited state BCRC, the productive pathway becomes accessible and outcompetes the thermal elimination to furnish the desired product.

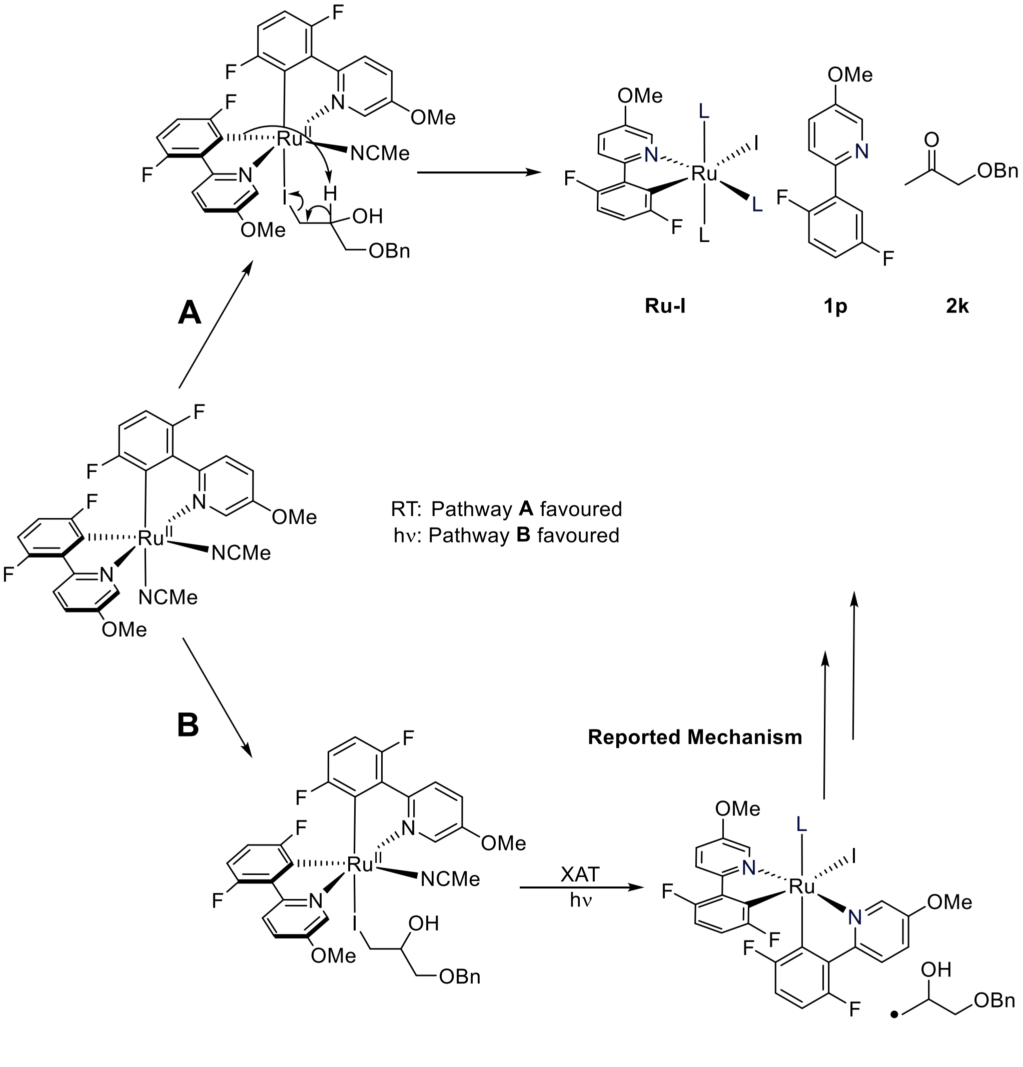

Figure 48e Proposed mechanistic switch between reaction under dark and illuminated conditions

4.12. Computational Studies

Molecular geometries were optimised with the Gaussian16 software suite,[6] using the single crystal structures as an initial input. These calculations were run using the B3LYP method,[7, 8] with the 6-311G(d,p) Pople basis set[9] describing lighter elements while the ruthenium utilised the ECP28MWB pseudopotential[10] to define the inner 28 electrons and the associated ECP28MWB basis set for the valence electrons.[10, 11] These results were verified with subsequent frequency calculations to confirm a global minimum energy had been reached. Ethyl acetate solvent environments were approximated through PCM models. Time-dependent calculations were run on these structures, with the method adjusted to M06-L[12] to give the best agreement with experimental data; spectra were convoluted from the log file in GaussView6. Theoretical excited state absorptions were obtained with similar time-dependent calculations using an optimised triplet structure as the initial state.[13]

The optimised structure of BCRC **6a** shows good agreement with the single crystal data (Table 11), with the angles surrounding the metal less than 1% different, and the mean bond lengths for identical ligands less than 1.25%. The results of the TD-DFT calculations performed with the M06L method and incorporating an approximation of ethyl acetate as solvent show good agreement with the experimentally obtained spectrum (Figure 49). The experimental MLCT band at 500 nm appears narrower in the calculated spectrum possibly due to underrepresentation of the *d-d* transitions also in this region.

Table 11: Comparison of XRD structures to optimised geometries.

Bond angle /°	XRD	DFT
C(py)-Ru-C(py′)	90.09	90.12
N(py)-Ru-N(py`)	177.15	176.84
N(sol)-Ru-N(sol`)	89.34	89.95

Bond distance /Å	XRD	DFT
Ru-C(py)	2.036	2.056
	2.025	2.056
mean	2.030	2.056
Ru-N(py)	2.061	2.088
	2.066	2.088
mean	2.063	2.088
Ru-N(sol)	2.111	2.102
	2.101	2.102
mean	2.106	2.102

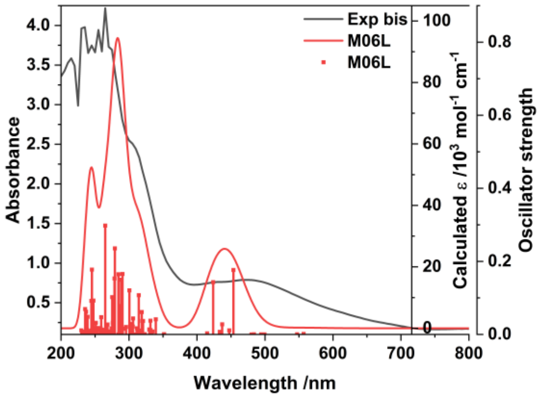

Figure 49. Calculated absorption spectra (M06L SCRF=ethyl acetate, half-width half-height = 0.15 eV) with individual transition bars overlaying the experimental.

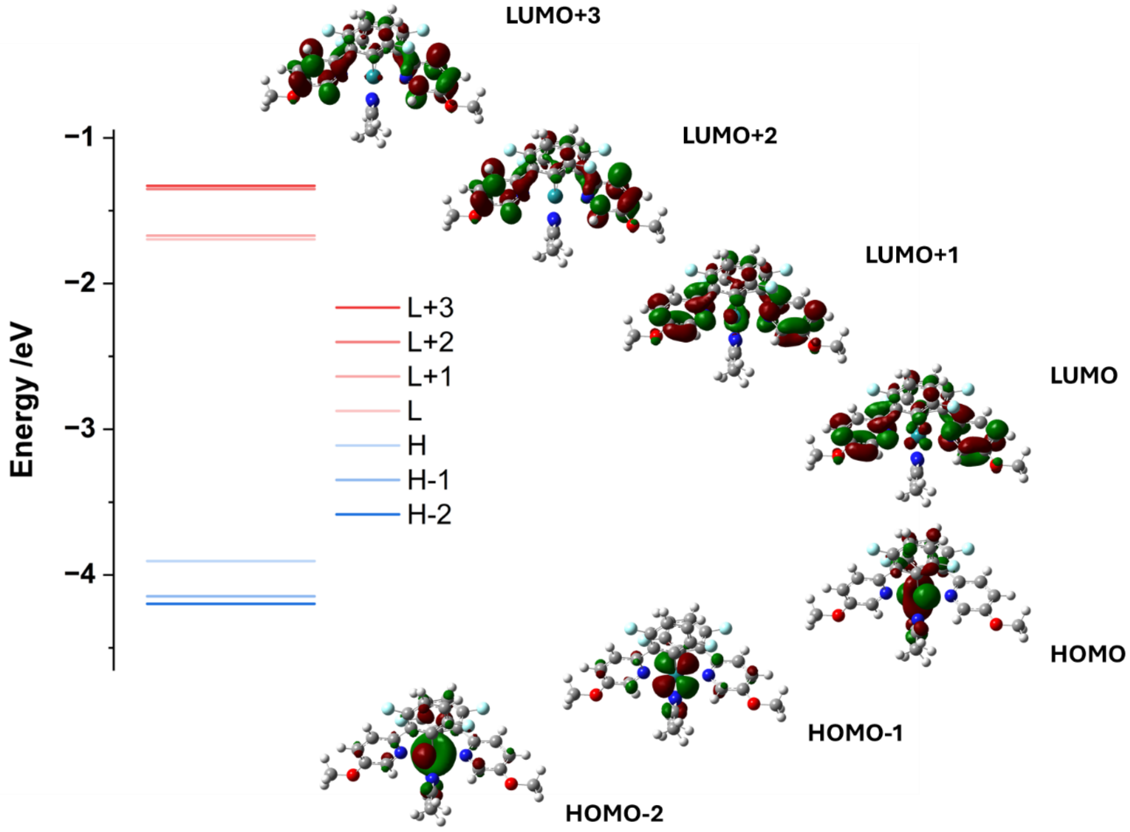

*Figure 50: Frontier molecular orbitals overlaid on the optimised structure of BCRC **6a** (isovalue = 0.035) and a plot of their relative energies (H = HOMO, highest occupied molecular orbital, L = LUMO, lowest unoccupied molecular orbital).*

HOMO = -3.90502 eV

LUMO = -1.69699 eV

Δ = 2.20803 eV

Table 12: Calculated TD-DFT transitions for BCRC 6a (M06L, SCRF = EtAc, nstates = 100), the molecular orbital transitions involved, and their respective contributions.

Wavelength /nm	Oscillator strength	Transition	Contribution
556.81	0.0016	H -> L	1.00
547.19	0.0005	H -> L+1	1.00
499.19	0.0002	H-2 -> L	0.40
		H-1 -> L+1	0.60
494.27	0.0002	H-2 -> L+1	0.28
		H-1 -> L	0.68
		H -> L+3	0.03
483.78	0.0006	H-2 -> L	0.06
		H-1 -> L+1	0.03
		H -> L+2	0.90
480.15	0	H-2 -> L+1	0.15
		H -> L+3	0.84
453.51	0.1766	H-2 -> L	0.41
		H-2 -> L+2	0.02
		H-1 -> L+1	0.28
		H-1 -> L+3	0.20
		H -> L+2	0.08
447.47	0.0111	H-2 -> L+1	0.21
		H-1 -> L	0.09
		H-1 -> L+2	0.64
		H -> L+3	0.05
437.00	0.0278	H-2 -> L	0.02
		H-2 -> L+2	0.52
		H-1 -> L+3	0.44
433.42	0.0072	H-2 -> L+1	0.10
		H-2 -> L+3	0.67
		H-1 -> L	0.06
		H-1 -> L+2	0.14
		H -> L+3	0.03
423.69	0.143	H-2 -> L	0.09
		H-2 -> L+2	0.45
		H-1 -> L+1	0.07
		H-1 -> L+3	0.35
414.86	0.003	H-2 -> L+1	0.23
		H-2 -> L+3	0.32
		H-1 -> L	0.14
		H-1 -> L+2	0.20
		H -> L+3	0.04
351.68	0.0007	H-4 -> L+1	0.42

		H-3 -> L	0.57
351.28	0.0001	H-4 -> L	0.47
		H-3 -> L+1	0.52
339.33	0.0414	H-4 -> L+1	0.03
		H -> L+4	0.71
		H -> L+6	0.21
334.40	0.0081	H-5 -> L	0.47
		H -> L+5	0.40
		H -> L+7	0.09
332.79	0.0053	H-5 -> L	0.39
		H-4 -> L	0.03
		H -> L+5	0.16
		H -> L+7	0.35
331.74	0.0119	H-5 -> L+1	0.90
		H-4 -> L+1	0.03
331.40	0.0377	H-4 -> L+1	0.02
		H-2 -> L+4	0.03
		H-2 -> L+6	0.02
		H -> L+4	0.14
		H -> L+6	0.73
329.85	0.0137	H-5 -> L	0.04
		H-4 -> L	0.05
		H-3 -> L+1	0.02
		H-2 -> L+5	0.03
		H -> L+5	0.28
		H -> L+7	0.52
		H -> L+8	0.02
323.17	0.0007	H-1 -> L+4	0.93
		H -> L+8	0.02
320.62	0.037	H-4 -> L+1	0.10
		H-4 -> L+3	0.22
		H-3 -> L	0.07
		H-3 -> L+2	0.48
		H-2 -> L+4	0.09
320.02	0.0001	H-4 -> L+2	0.42
		H-3 -> L+3	0.53
319.46	0.037	H-4 -> L+1	0.10
		H-4 -> L+3	0.16
		H-3 -> L	0.08
		H-3 -> L+2	0.09
		H-2 -> L+4	0.32
		H-1 -> L+5	0.20
318.70	0.0614	H-6 -> L+1	0.03
		H-5 -> L	0.02
		H-4 -> L	0.31
		H-4 -> L+2	0.06

		H-3 -> L+1	0.31
		H-2 -> L+5	0.15
		H -> L+5	0.02
318.17	0.0106	H-4 -> L+1	0.04
		H-3 -> L	0.03
		H-3 -> L+2	0.02
		H-2 -> L+4	0.07
		H-1 -> L+5	0.79
316.31	0.0032	H-2 -> L+5	0.02
		H-1 -> L+4	0.03
		H-1 -> L+6	0.03
		H -> L+8	0.82
		H -> L+11	0.02
314.33	0.1074	H-6 -> L	0.03
		H-4 -> L+1	0.14
		H-4 -> L+3	0.08
		H-3 -> L	0.11
		H-2 -> L+4	0.36
		H-2 -> L+6	0.10
		H-1 -> L+7	0.02
		H -> L+4	0.04
		H -> L+9	0.03
312.31	0.0075	H-1 -> L+7	0.96
311.74	0.0168	H-2 -> L+5	0.47
		H-2 -> L+7	0.07
		H-1 -> L+6	0.32
		H -> L+10	0.06
		H -> L+11	0.02
307.48	0	H-2 -> L+7	0.85
		H-1 -> L+6	0.08
		H -> L+11	0.03
305.96	0.0438	H-6 -> L+1	0.03
		H-5 -> L+2	0.18
		H-2 -> L+5	0.22
		H-2 -> L+7	0.02
		H-2 -> L+8	0.12
		H-1 -> L+6	0.21
		H -> L+5	0.03
		H -> L+8	0.04
		H -> L+10	0.06
		H -> L+11	0.04
305.62	0.0216	H-5 -> L+3	0.13
		H-2 -> L+4	0.04
		H-2 -> L+6	0.56
		H-1 -> L+8	0.10
		H -> L+9	0.12

303.78	0.0291	H-5 -> L+2	0.65
		H-4 -> L+2	0.05
		H-2 -> L+5	0.02
		H-2 -> L+8	0.06
		H-1 -> L+6	0.04
		H -> L+11	0.03
303.72	0.0189	H-5 -> L+3	0.54
		H-2 -> L+6	0.11
		H-1 -> L+8	0.17
		H -> L+9	0.13
300.52	0.1206	H-5 -> L+3	0.20
		H-4 -> L+3	0.10
		H-3 -> L+2	0.03
		H-1 -> L+8	0.12
		H -> L+9	0.48
295.62	0.0203	H-5 -> L+2	0.02
		H-4 -> L+2	0.07
		H-3 -> L+3	0.04
		H-2 -> L+8	0.21
		H-2 -> L+10	0.03
		H-2 -> L+11	0.02
		H-1 -> L+9	0.02
		H -> L+10	0.35
		H -> L+11	0.15
290.90	0.0158	H-4 -> L+2	0.08
		H-3 -> L+3	0.09
		H-2 -> L+8	0.23
		H-2 -> L+10	0.14
		H-2 -> L+11	0.07
		H-1 -> L+9	0.05
		H -> L+11	0.26
290.35	0.1656	H-6 -> L	0.07
		H-4 -> L+1	0.03
		H-4 -> L+3	0.13
		H-3 -> L	0.03
		H-3 -> L+2	0.11
		H-2 -> L+6	0.02
		H-2 -> L+9	0.21
		H-1 -> L+8	0.09
		H-1 -> L+10	0.12
		H -> L+9	0.08
		H -> L+12	0.04
288.34	0.048	H-6 -> L+1	0.02
		H-4 -> L+2	0.03
		H-3 -> L+3	0.03
		H-2 -> L+8	0.10

		H-1 -> L+9	0.57
		H -> L+10	0.06
		H -> L+11	0.10
288.28	0.1498	H-6 -> L+2	0.02
		H-4 -> L+3	0.09
		H-3 -> L+2	0.07
		H-2 -> L+9	0.05
		H-1 -> L+8	0.07
		H-1 -> L+10	0.05
		H-1 -> L+11	0.06
		H -> L+12	0.49
287.30	0.0056	H-6 -> L	0.58
		H-5 -> L+3	0.02
		H-1 -> L+10	0.15
		H-1 -> L+11	0.14
286.92	0.0302	H-6 -> L	0.09
		H-4 -> L+3	0.02
		H-3 -> L+2	0.03
		H-2 -> L+9	0.02
		H-1 -> L+8	0.03
		H-1 -> L+10	0.13
		H-1 -> L+11	0.21
		H -> L+12	0.40
286.83	0.0776	H-6 -> L+3	0.03
		H-4 -> L+2	0.11
		H-3 -> L+1	0.02
		H-3 -> L+3	0.11
		H-2 -> L+10	0.11
		H-1 -> L+9	0.35
		H -> L+10	0.03
		H -> L+11	0.11
284.43	0.1648	H-6 -> L	0.06
		H-2 -> L+6	0.04
		H-2 -> L+9	0.17
		H-1 -> L+8	0.19
		H-1 -> L+10	0.31
		H-1 -> L+11	0.04
		H -> L+4	0.03
284.37	0.0002	H-6 -> L+1	0.77
		H-5 -> L+2	0.03
		H-3 -> L+3	0.03
		H-2 -> L+8	0.02
		H-2 -> L+10	0.03
279.23	0.2356	H-6 -> L+1	0.03
		H-6 -> L+3	0.03
		H-4 -> L+2	0.04

		H-3 -> L+1	0.02
		H-3 -> L+3	0.04
		H-2 -> L+5	0.02
		H-2 -> L+8	0.02
		H-2 -> L+10	0.37
		H-2 -> L+11	0.06
		H-1 -> L+6	0.07
		H-1 -> L+12	0.03
		H -> L+11	0.14
278.60	0.1533	H-6 -> L+2	0.03
		H-4 -> L+3	0.05
		H-3 -> L+2	0.05
		H-2 -> L+9	0.37
		H-1 -> L+10	0.12
		H-1 -> L+11	0.10
		H -> L+9	0.04
		H -> L+13	0.10
276.06	0.0321	H-6 -> L+2	0.02
		H-1 -> L+10	0.03
		H-1 -> L+11	0.08
		H -> L+13	0.79
275.29	0.1022	H-6 -> L	0.04
		H-2 -> L+6	0.08
		H-2 -> L+9	0.14
		H-1 -> L+8	0.13
		H-1 -> L+11	0.28
		H -> L+9	0.06
		H -> L+12	0.03
		H -> L+13	0.06
		H -> L+15	0.04
		H -> L+17	0.03
274.30	0.0166	H-2 -> L+11	0.02
		H -> L+14	0.89
273.66	0.0062	H-2 -> L+10	0.06
		H-2 -> L+11	0.59
		H-1 -> L+12	0.20
		H -> L+11	0.04
		H -> L+14	0.03
271.07	0.0059	H-2 -> L+10	0.06
		H-2 -> L+11	0.14
		H-1 -> L+12	0.74
		H-1 -> L+17	0.02
269.64	0.0083	H -> L+15	0.93
268.42	0.0273	H-6 -> L+2	0.02
		H-2 -> L+12	0.90
265.00	0.298	H-6 -> L+2	0.79

		H-4 -> L+3	0.04
		H-3 -> L+2	0.02
264.90	0.0049	H-6 -> L+3	0.74
		H-4 -> L+2	0.03
		H-2 -> L+10	0.04
		H -> L+16	0.03
263.10	0.0008	H-6 -> L+3	0.02
		H-1 -> L+13	0.26
		H -> L+16	0.67
262.82	0.0055	H-7 -> L+1	0.03
		H-2 -> L+11	0.02
		H-1 -> L+13	0.63
		H -> L+16	0.26
261.33	0.0122	H-7 -> L	0.04
		H-2 -> L+13	0.11
		H-1 -> L+14	0.68
		H -> L+17	0.15
259.62	0.0133	H-7 -> L	0.22
		H-2 -> L+13	0.70
		H -> L+17	0.03
259.13	0.0473	H-6 -> L+3	0.03
		H-2 -> L+8	0.02
		H-2 -> L+10	0.02
		H-2 -> L+14	0.72
		H-1 -> L+6	0.03
		H -> L+10	0.04
258.18	0.0172	H-8 -> L+1	0.29
		H-7 -> L	0.41
		H-2 -> L+13	0.09
		H-1 -> L+14	0.12
		H -> L+17	0.05
257.63	0.0002	H-8 -> L	0.33
		H-7 -> L+1	0.63
256.88	0.0132	H-7 -> L	0.15
		H-2 -> L+15	0.13
		H-2 -> L+17	0.05
		H-1 -> L+14	0.05
		H -> L+17	0.54
256.10	0	H-2 -> L+14	0.02
		H-1 -> L+15	0.91
		H-1 -> L+17	0.04
253.19	0.0176	H-10 -> L+1	0.02
		H-9 -> L	0.24
		H-8 -> L	0.42
		H-7 -> L+1	0.20
253.13	0.0002	H-2 -> L+15	0.83

		H-2 -> L+17	0.06
		H -> L+17	0.05
251.58	0.0024	H-5 -> L+5	0.02
		H-4 -> L+5	0.20
		H-3 -> L+4	0.72
251.18	0.0318	H-5 -> L+4	0.03
		H-4 -> L+4	0.61
		H-3 -> L+5	0.33
251.06	0.016	H-9 -> L+1	0.04
		H-8 -> L+1	0.24
		H-7 -> L	0.03
		H-2 -> L+17	0.03
		H-1 -> L+14	0.02
		H-1 -> L+16	0.56
250.49	0.0149	H-9 -> L+1	0.17
		H-8 -> L+1	0.27
		H-7 -> L	0.06
		H-1 -> L+14	0.02
		H-1 -> L+16	0.39
248.45	0.0166	H-9 -> L	0.06
		H-3 -> L+5	0.04
		H-2 -> L+16	0.83
247.60	0.0928	H-9 -> L	0.07
		H-5 -> L+4	0.03
		H-4 -> L+6	0.51
		H-3 -> L+5	0.02
		H-2 -> L+8	0.03
		H-2 -> L+14	0.07
		H-1 -> L+6	0.03
		H -> L+10	0.06
247.05	0.0052	H-3 -> L+5	0.05
		H-3 -> L+7	0.91
246.93	0.0037	H-4 -> L+5	0.02
		H-4 -> L+7	0.16
		H-3 -> L+6	0.81
246.63	0.0008	H-4 -> L+5	0.07
		H-4 -> L+7	0.71
		H-3 -> L+6	0.19
246.59	0.0022	H-10 -> L+1	0.15
		H-9 -> L	0.41
		H-8 -> L	0.07
		H-7 -> L+1	0.04
		H-4 -> L+4	0.02
		H-3 -> L+5	0.08
		H-2 -> L+16	0.09
		H-1 -> L+17	0.04

246.09	0.0112	H-10 -> L	0.26
		H-9 -> L+1	0.55
		H-8 -> L+1	0.07
		H-7 -> L	0.02
		H-4 -> L+5	0.03
245.42	0.1776	H-10 -> L+1	0.04
		H-9 -> L	0.06
		H-5 -> L+4	0.03
		H-4 -> L+4	0.08
		H-4 -> L+6	0.39
		H-3 -> L+5	0.15
		H-2 -> L+8	0.02
		H-2 -> L+14	0.04
		H-1 -> L+6	0.02
		H -> L+10	0.03
244.55	0.0919	H-10 -> L	0.07
		H-4 -> L+5	0.54
		H-4 -> L+7	0.12
		H-3 -> L+4	0.13
244.03	0.011	H-10 -> L+1	0.10
		H-4 -> L+4	0.13
		H-4 -> L+6	0.07
		H-4 -> L+9	0.05
		H-3 -> L+5	0.21
		H-3 -> L+7	0.05
		H-3 -> L+8	0.02
		H-2 -> L+8	0.03
		H-2 -> L+10	0.03
		H-1 -> L+6	0.04
		H -> L+10	0.09
241.40	0.0075	H-8 -> L+3	0.08
		H-7 -> L+2	0.90
240.60	0	H-8 -> L+2	0.21
		H-7 -> L+3	0.74
		H-1 -> L+17	0.04
239.52	0.0011	H-8 -> L+3	0.03
		H-5 -> L+5	0.03
		H-5 -> L+7	0.13
		H-2 -> L+17	0.63
		H -> L+17	0.05
239.44	0.0018	H-7 -> L+3	0.05
		H-5 -> L+6	0.20
		H-2 -> L+16	0.02
		H-1 -> L+17	0.60
239.00	0.0485	H-10 -> L+1	0.04
		H-5 -> L+4	0.50

		H-5 -> L+6	0.26
		H-5 -> L+9	0.04
		H-1 -> L+17	0.02
238.99	0.0037	H-8 -> L+3	0.02
		H-5 -> L+5	0.08
		H-5 -> L+7	0.73
		H-2 -> L+17	0.12
238.57	0.0024	H-8 -> L+2	0.04
		H-5 -> L+4	0.12
		H-5 -> L+6	0.32
		H-3 -> L+8	0.32
		H-1 -> L+17	0.07
238.06	0.001	H-4 -> L+8	0.99
237.93	0.0131	H-9 -> L+2	0.05
		H-8 -> L+2	0.64
		H-7 -> L+3	0.15
		H-5 -> L+4	0.03
		H-3 -> L+8	0.05
		H-1 -> L+17	0.02
237.74	0.0221	H-5 -> L+4	0.03
		H-5 -> L+6	0.18
		H-3 -> L+8	0.57
		H-1 -> L+17	0.05
237.26	0.0316	H-10 -> L	0.12
		H-9 -> L+1	0.02
		H-9 -> L+3	0.03
		H-8 -> L+3	0.57
		H-7 -> L+2	0.03
		H-5 -> L+5	0.08
		H-5 -> L+7	0.04
236.75	0.0621	H-10 -> L	0.14
		H-9 -> L+1	0.04
		H-8 -> L+3	0.21
		H-5 -> L+5	0.32
		H-5 -> L+7	0.05
		H-4 -> L+10	0.04
		H-2 -> L+17	0.04
235.60	0.0026	H-10 -> L+1	0.51
		H-9 -> L	0.06
		H-8 -> L	0.04
		H-5 -> L+4	0.05
		H-4 -> L+9	0.08
		H-3 -> L+10	0.11
235.47	0.0695	H-10 -> L	0.24
		H-9 -> L+1	0.06
		H-5 -> L+5	0.28

		H-5 -> L+7	0.03
		H-5 -> L+10	0.02
		H-4 -> L+10	0.03
		H-3 -> L+9	0.21
231.89	0.0001	H-10 -> L+3	0.05
		H-9 -> L+2	0.83
		H-8 -> L+2	0.02
		H-3 -> L+10	0.04
231.27	0.0076	H-10 -> L+2	0.07
		H-9 -> L+3	0.84
		H-8 -> L+3	0.02
		H-3 -> L+9	0.02
230.91	0.0049	H-5 -> L+8	0.99
229.88	0.0113	H-5 -> L+5	0.03
		H-5 -> L+10	0.02
		H-4 -> L+10	0.46
		H-3 -> L+9	0.45

5.0. X-Ray Crystallographic Data

Data Collection: Crystals suitable for diffraction were isolated by slow diffusion of diethyl ether into acetonitrile for compounds **4b** and **4c**. Compounds **6a** and **9a** were crystallised similarly, using hexane and benzene as the solvents. Single crystal X-Ray diffraction data for compound **9a** was collected at 100 K on a Rigaku XtaLab AFC-11 four circle goniometer equipped with a Hypix6000HE detector and Oxford cryosystem. Data was collected with a dual source Rigaku FR-X rotating anode using Cu – Kα (λ= 1.54184 Å) radiation. Single crystal X-Ray diffraction data for compounds **4b**, **6a** and **4c** was collected at 100 K on an Agilent Supernova 4-circle diffractometer equipped with an Agilent Eos detector using Mo – Kα (λ= 0.71073 Å) radiation. Data was collected using the CrysAlisPro program.

Crystal structure determination and refinements: Data processing and reduction was performed with CrysAlisPro. Empirical absorption correction was applied using spherical harmonics, implemented with the SCALE3 ABSPACK algorithm. The crystal structure was solved and refined using the SHELX suite of programmes in Olex2.[14, 15] All non – hydrogen atoms were refined anisotropically. Hydrogen atom positions were calculated and assigned fixed isotropic displacement parameters. Some PF6 ions were disordered and modelled over two positions.

Crystallographic data has been deposited with the CCDC with the following deposition numbers:

4b: 2290546

4c: 2290565

6a: 2290545

9a: 2290531

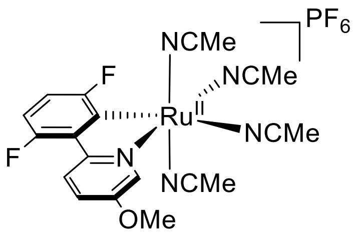

=

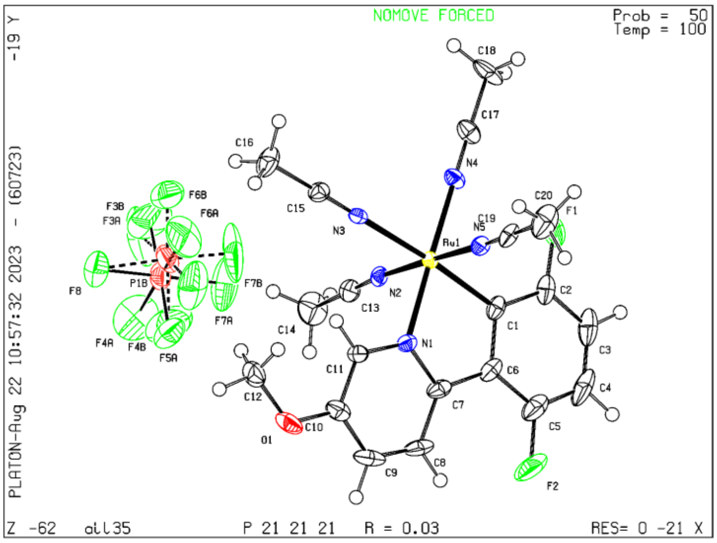

4b

CCDC - 2290546

Table 13. Crystal data and structure refinement for 4b

Empirical formula	$C_{20}H_{20}F_8N_5OPRu$
Formula weight	630.45
Temperature/K	99.9(2)
Crystal system	orthorhombic
Space group	$P2_12_12_1$
a/Å	12.7423(3)
b/Å	13.1528(3)
c/Å	14.7676(4)
α/°	90
β/°	90
γ/°	90
Volume/Å^3	2475.00(11)
Z	4
ρ_{calc}g/cm^3	1.692
μ/mm^{-1}	0.781
F(000)	1256.0
Crystal size/mm^3	0.537 × 0.351 × 0.131
Radiation	Mo Kα (λ = 0.71073)
2Θ range for data collection/°	6.782 to 58.168
Index ranges	-15 ≤ h ≤ 15, -16 ≤ k ≤ 17, -20 ≤ l ≤ 18
Reflections collected	28950
Independent reflections	5775 [R_{int} = 0.0435, R_{sigma} = 0.0407]
Data/restraints/parameters	5775/93/385
Goodness-of-fit on F^2	1.040
Final R indexes [I>=2σ (I)]	R_1 = 0.0310, wR_2 = 0.0644
Final R indexes [all data]	R_1 = 0.0340, wR_2 = 0.0662
Largest diff. peak/hole / e Å^{-3}	1.06/-0.55

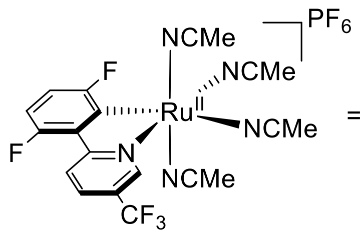

4c

CCDC - 2290565

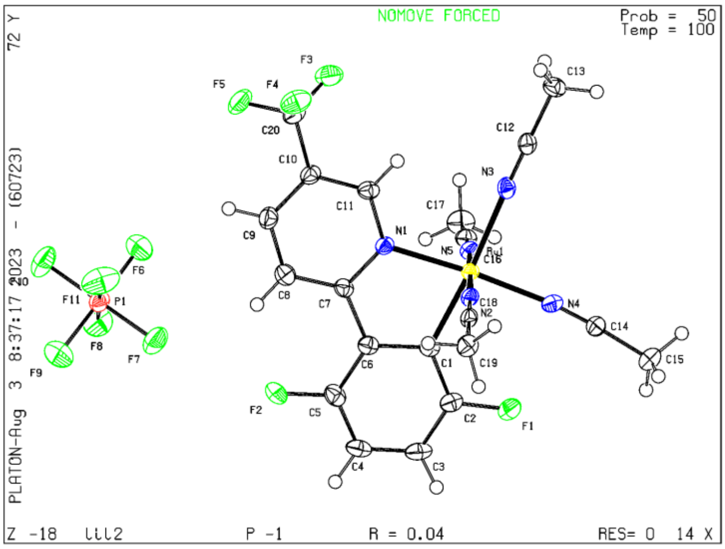

Table 14. Crystal data and structure refinement for 4c

Empirical formula	$C_{20}H_{17}F_{11}N_5PRu$
Formula weight	668.42
Temperature/K	100.0(2)
Crystal system	triclinic
Space group	P-1
a/Å	8.5617(4)
b/Å	11.4349(5)
c/Å	13.6362(4)
α/°	88.715(3)
β/°	78.542(4)
γ/°	69.269(4)
Volume/Å^3	1222.02(9)
Z	2
ρ_{calc}g/cm^3	1.817
μ/mm^{-1}	6.797
F(000)	660
Crystal size/mm^3	0.19 × 0.14 × 0.12
Radiation	Cu Kα (λ = 1.54184)
2Θ range for data collection/°	6.622 to 152.19
Index ranges	-10 ≤ h ≤ 8, -14 ≤ k ≤ 14, -17 ≤ l ≤ 16
Reflections collected	9558
Independent reflections	4739 [R_{int} = 0.0673, R_{sigma} = 0.0549]
Data/restraints/parameters	4739/0/347
Goodness-of-fit on F^2	1.032
Final R indexes [I>=2σ (I)]	R_1 = 0.0428, wR_2 = 0.1156
Final R indexes [all data]	R_1 = 0.0435, wR_2 = 0.1164
Largest diff. peak/hole / e Å^{-3}	1.29/-1.70

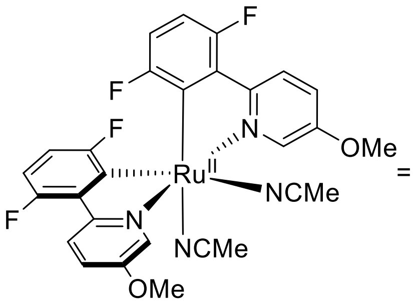

6a

CCDC - 2290545

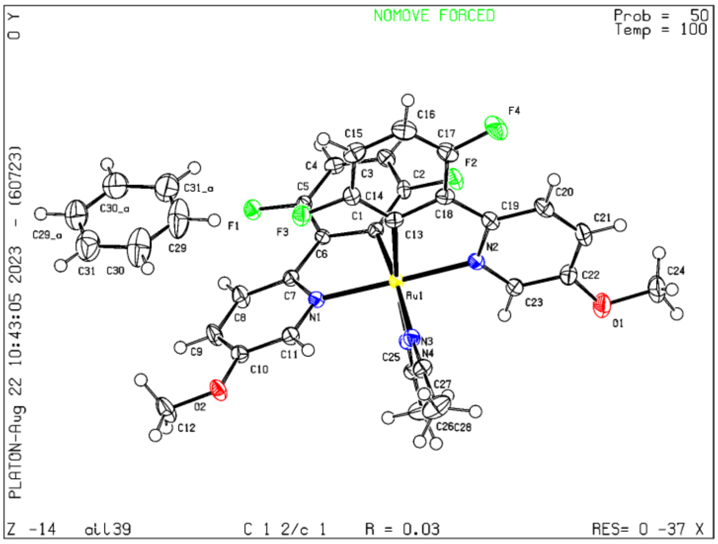

Table 15. Crystal data and structure refinement for 6a

Empirical formula	$C_{31}H_{25}F_4N_4O_2Ru$
Formula weight	662.62
Temperature/K	99.8(3)
Crystal system	monoclinic
Space group	C2/c
a/Å	22.0235(6)
b/Å	16.2117(4)
c/Å	16.2520(4)
α/°	90
β/°	108.440(3)
γ/°	90
Volume/Å^3	5504.7(3)
Z	8
ρ_{calc}g/cm^3	1.599
μ/mm^{-1}	0.634
F(000)	2680.0
Crystal size/mm^3	0.457 × 0.076 × 0.059
Radiation	Mo Kα (λ = 0.71073)
2Θ range for data collection/°	6.994 to 58.08
Index ranges	-15 ≤ h ≤ 29, -18 ≤ k ≤ 20, -21 ≤ l ≤ 22
Reflections collected	11954
Independent reflections	6232 [R_{int} = 0.0226, R_{sigma} = 0.0417]
Data/restraints/parameters	6232/0/383
Goodness-of-fit on F^2	1.063
Final R indexes [I>=2σ (I)]	R_1 = 0.0333, wR_2 = 0.0680
Final R indexes [all data]	R_1 = 0.0459, wR_2 = 0.0735
Largest diff. peak/hole / e Å^{-3}	0.45/-0.38

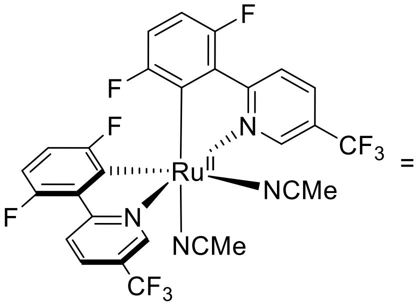

9a

CCDC - 2290531

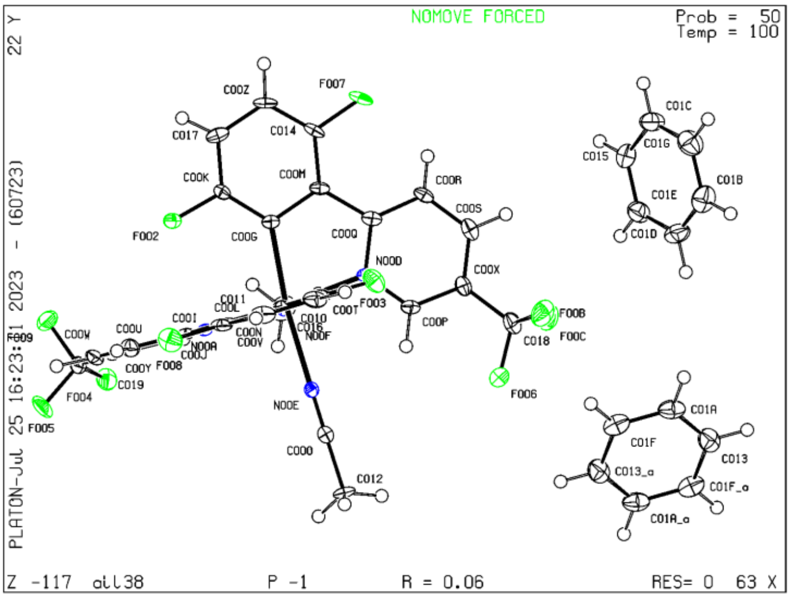

Table 16. Crystal data and structure refinement for 9a

Empirical formula	$C_{37}H_{25}F_{10}N_4Ru$
Formula weight	816.68
Temperature/K	99.8(4)
Crystal system	triclinic
Space group	P-1
a/Å	8.8646(6)
b/Å	12.2846(9)
c/Å	16.9698(11)
α/°	110.762(6)
β/°	93.267(6)
γ/°	105.811(6)
Volume/Å^3	1638.2(2)
Z	2
ρ_{calc}g/cm^3	1.656
μ/mm^{-1}	0.571
F(000)	818
Crystal size/mm^3	0.12 × 0.06 × 0.05
Radiation	Mo Kα (λ = 0.71073)
2Θ range for data collection/°	6.9 to 58.18
Index ranges	-12 ≤ h ≤ 11, -12 ≤ k ≤ 16, -22 ≤ l ≤ 22
Reflections collected	11359
Independent reflections	7298 [R_{int} = 0.0521, R_{sigma} = 0.1234]
Data/restraints/parameters	7298/0/471
Goodness-of-fit on F^2	1.032

Final R indexes [I>=2σ (I)]	R_1 = 0.0582, wR_2 = 0.0713
Final R indexes [all data]	R_1 = 0.0980, wR_2 = 0.0831
Largest diff. peak/hole / e $Å^{-3}$	0.72/-0.77

6.0 Procedures

6.1. General Procedures

General Procedure A: Synthesis of Mono-cyclometallated Ruthenium Complexes

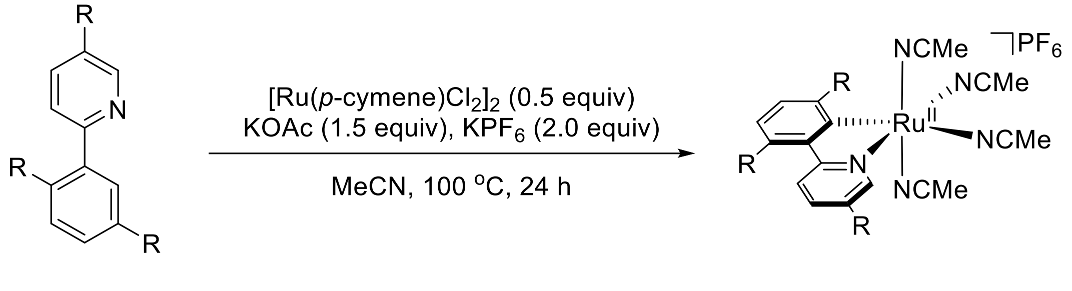

An oven dried 100 mL Ace pressure tube equipped with a stirring bar was transferred to a glove box, then $[RuCl_2(p\text{-cymene})]_2$ (490 mg, 0.80 mmol, 0.50 equiv), KOAc (236 mg, 2.40 mmol, 1.50 equiv), KPF_6 (589 mg, 3.20 mmol, 2.00 equiv), phenylpyridine (1.6 mmol, 1.0 equiv) and MeCN (10 mL, 0.16 M) were added. The tube was sealed, transferred out of the box, placed in an oil bath at 100 °C and stirred for 24 h. Upon completion, the reaction crude was loaded in an aluminium oxide (Al_2O_3, neutral) column conditioned with CH_2Cl_2 and quickly eluted with CH_3CN/CH_2Cl_2 (1:1) using N_2 in replacement of compressed air collecting the yellow/orange band. The solution was concentrated under reduced pressure and then quickly precipitated with Et_2O affording the desired complex **4a** (876 mg, 90 %) as a yellow/orange solid. The complex has to be promptly transferred to a glove box as it decomposes turning green if exposed to air. The complexes are subjected to quantitative 1 H NMR after their synthesis. They are generally in the region of 99% pure by this measure. If they are of lower purity, then the complex should be dissolved in MeCN inside the glovebox and filtered through a small plug of alumina. Then it should be concentrated under vacuum and crashed out with Et2O/Pentane (1:1). Data is in accordance with those previously reported.[16]

General Procedure B: Synthesis of 2-Phenylpyridines

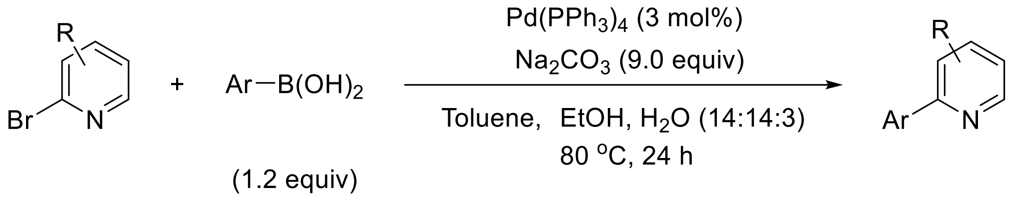

An oven dried round bottomed flask equipped with a stirrer bar was charged with 2-halopyridine (10 mmol, 1.0 equiv), aryl boronic acid (12 mmol, 1.2 equiv), $Pd(PPh_3)_4$ (347 mg, 0.30 mmol, 3.00 mol%) and anhydrous Na_2CO_3 (9.5 g, 90 mmol, 9.0 equiv). The flask was then purged with nitrogen for 10 minutes. Degassed toluene (35 mL), water (35 mL) and EtOH (7.5 mL) was added and the resulting mixture was stirred at 80 °C for 24 h under nitrogen. The resulting mixture was washed with a saturated NH_4Cl solution (100 mL) and dried over $MgSO_4$. Purification by flash column chromatography gave the pure product.

General Procedure C: Ruthenium-Catalyzed Ortho Alkylation of Arenes using Epoxides with 440 nm Light

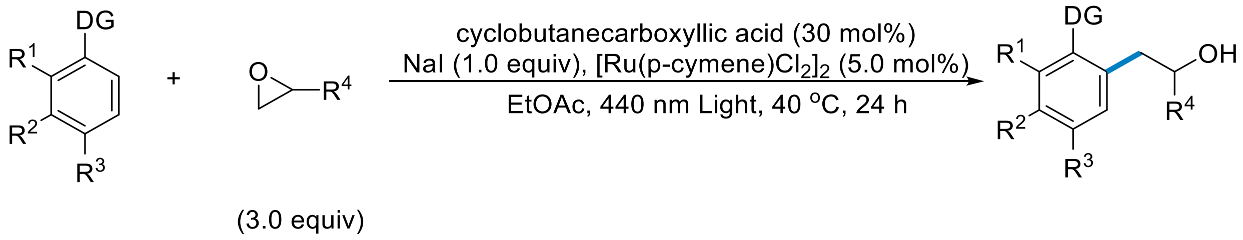

An oven dried 10 mL microwave vial equipped with a stirrer bar was charged with sodium iodide (75 mg, 0.5 mmol, 1.0 equiv), $[(p\text{-cymene})RuCl_2]_2$ (15 mg, 0.025 mmol, 5.0 mol%) and purged with nitrogen for 10 minutes, the arene (0.5 mmol, 1.0 equiv), epoxide (1.5 mmol, 3.0 equiv) and ethyl acetate (0.875 mL) were added followed by cyclobutanecarboxylic acid (15 µL, 0.15 mmol, 30 mol%). The vial was stirred for 24 h under a 440 nm LED. Purification by solid loaded flash column chromatography gave the pure product.

General Procedure D: Ruthenium-Catalyzed Ortho Alkylation of Arenes using Epoxides with 365 nm Light

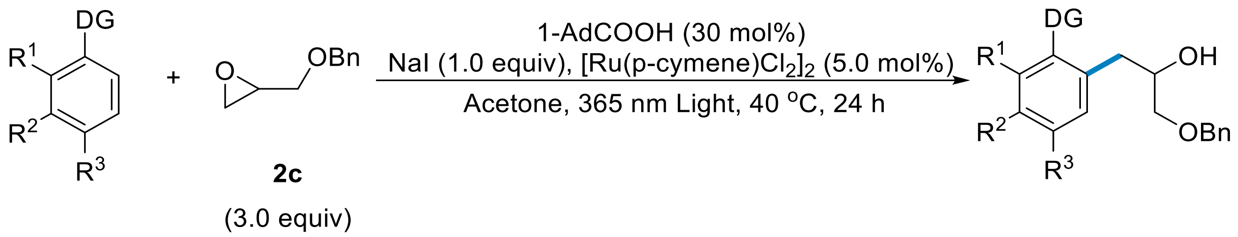

An oven dried 10 mL microwave vial equipped with a stirrer bar was charged with sodium iodide (75 mg, 0.5 mmol, 1.0 equiv), [(*p*-cymene)$RuCl_2]_2$ (15 mg, 0.025 mmol, 5.0 mol%) and purged with nitrogen for 10 minutes, the arene (0.5 mmol, 1.0 equiv), **2c** (0.23 mL, 1.50 mmol, 3.0 equiv) and acetone (0.875 mL) were added followed by adamantylcarboxylic acid (15 µL, 0.15 mmol, 30 mol%). The vial was stirred for 24 h under a 365 nm LED. Purification by solid loaded flash column chromatography gave the pure product.

6.2. Specific Procedures

NMR experiment monitoring the formation of the cyclised product

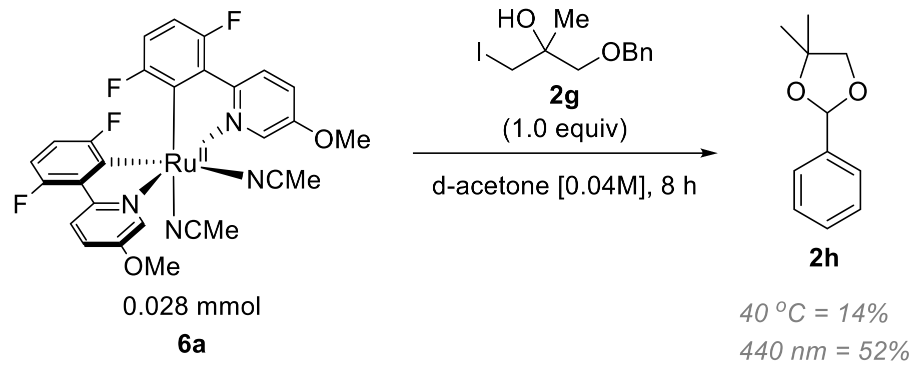

0.028 mmol

6a

2g

2h

In an argon filled glove box a stock solution of alkyl iodide **2g** (34.2 mg, 0.112 mmol) was prepared in d-Acetone (2 mL). Two oven dried j-young NMR tubes were each charged with BCRC **6a** (19.8 mg, 0.028 mmol) followed by the stock solution of alkyl iodide **2g** (0.5 mL). To each tube was then added d-Acetone (0.2 mL) for a final concentration of 0.04M. One tube was irradiated at 440 nm for 8 h, while the other was heated to 45 °C for 8 h. Each reaction was subjected to qNMR analysis. Conversion was calculated by the ratio of the ^{1}H signals at 5.85 (**2h**) and 3.50 (**2g**)

7.0. Preparation of Ruthenium Complexes

7.1. Preparation of Mono-Cyclometallated Ruthenium Complexes

Mono-cyclometallated complex (4a)

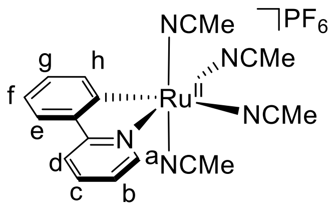

4a

The title compound was synthesised as outlined in general procedure **A** using 2-phenylpyridine (78 µl, 0.5 mmol). The complex was isolated as an orange solid (220 mg, 0.29 mmol, 58%).

^{1}H NMR (400 MHz, CD_3CN) δ 8.91 (1H, d, *J* = 5.70, **H**[a]), 7.97 (1H, dd, *J* = 7.5, 1.3 Hz, **H**[d]), 7.89–7.86 (1H, m, **H**[h]), 7.77–7.70 (2H, m, Ar**H**), 7.18–7.06 (2H, m, Ar**H**), 6.95 (1H, td, *J* = 7.4, 1.3 Hz, **H**[f]), 2.51 (3H, s, C**H**$_3$), 2.00 (6H, s, C**H**$_3$), 1.96 (3H, s, C**H**$_3$).

^{13}C NMR (126 MHz, CD_3CN) δ 185.2 (C), 169.2 (C), 153.4 (CH), 147.8 (C), 139.2 (CH), 137.1 (CH), 128.5 (CH), 124.2 (CH), 122.1 (CH), 121.6 (CH), 118.7 (CH), 24.4 (CH_3), 4.4 (CH_3), 3.8 (2 x CH_3).

^{19}F NMR (376 MHz, CD_3CN) δ 71.9 (d, *J* = 25.8 Hz), 73.8 (d, *J* = 25.8 Hz).

Mono-cyclometallated complex (4b)

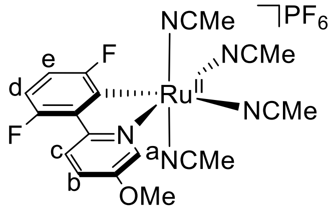

4b

The title compound was synthesised as outlined in general procedure **A** using **1p** (110 mg, 0.50 mmol). The complex was isolated as a yellow solid (658 mg, 0.31 mmol, 61%)

^{1}**H NMR** (400 MHz, CD_3CN) δ 8.70 (1H, d, *J* = 2.9 Hz, **H^a**), 8.15 (1H, dd, *J* = 9.1, 2.7 Hz, **H^b**), 7.42 (1H, dd, *J* = 9.1, 2.7 Hz, **H^c**), 6.75–6.70 (1H, m, **H^d**), 6.66–6.60 (1H, m, **H^e**), 3.94 (3H, s, C**H$_3$**), 2.43 (3H, s, C**H$_3$**), 2.06 (6H, s, 2 x C**H$_3$**), 1.96 (3H, s, C**H$_3$**).

^{13}**C NMR** (126 MHz, CD_3CN) δ 169.9 (C, d, *J* = 225.6 Hz), 165.3 (C, dd, *J* = 48.6, 3.0 Hz), 159.2 (C, d, *J* = 7.8 Hz), 157.2 (C, dd, *J* = 248.3, 3.0 Hz), 155.0 (C, d, *J* = 2.0 Hz), 141.6 (CH), 136.5 (C, dd, *J* = 18.8, 5.0 Hz), 124.4 (C), 123.9 (C), 123.7 (C), 122.9 (CH), 122.0 (CH, d, *J* = 3.0 Hz), 114.6 (CH, dd, *J* = 32.7, 10.0 Hz), 110.0 (CH, dd, *J* = 26.3, 9.7 Hz), 56.8 (OCH_3), 3.87 (CH_3).

^{19}**F NMR** (400 MHz, CD_3CN) δ -72.8 (d, *J* = 706.9 Hz), -104.9 (dq, *J* = 23.7, 3.4 Hz), -123.5 (dd, *J* = 23.0, 11.2 Hz).

HRMS (ESI^+): Mass calculated for $C_{20}H_{20}ON_5F_2Ru$ [M+] = 486.0679. Mass found = 486.0674.

m.p. 136–139 °C (Decomposition) (Et_2O)

Mono-cyclometallated complex (4c)

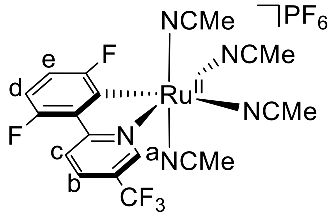

4c

The title compound was synthesised as outlined in general procedure **A** using **1t** (130 mg, 0.50 mmol). The complex was isolated as a yellow solid (821 mg, 0.36 mmol, 72%)

^{1}**H NMR** (400 MHz, CD_3CN) δ 9.24 (1H, s, **H^a**), 8.37 (1H, dd, *J* = 8.7, 3.2 Hz, **H^b**), 8.08–8.03 (1H, m, **H^c**), 6.88–6.83 (1H, m, **H^d**), 6.73–6.67 (1H, m, **H^e**), 2.45 (3H, s, C**H$_3$**), 2.05 (6H, s, C**H$_3$**), 1.96 (3H, s, C**H$_3$**).

^{13}**C NMR** (126 MHz, CD_3CN) δ 170.5 (C, d, *J* = 72.4 Hz), 170.4 (C, dd, *J* = 48.7, 3.2 Hz), 169.6 (C, d, *J* = 163.2 Hz), 159.2 (C, dd, *J* = 252.0, 3.9, Hz), 150.4 (CH, q, *J* = 4.6 Hz), 135.6 (C, dd, *J* = 18.5, 4.8 Hz), 134.5 (CH), 124.9 (C), 124.7 (C, dd, *J* = 34.1, 2.2 Hz), 124.1 (C, q, *J* = 269.4

Hz), 123.4 (C), 123.2 (CH, d, J = 4.0 Hz), 123.1 (C), 122.8 (C), 116.9 (CH, dd, J = 33.9, 10.1 Hz), 110.2 (CH, dd, J = 17.1, 8.3 Hz), 3.98 (CH_3), 3.90 (CH_3).

^{19}F NMR (471 MHz, CD_3CN) δ -62.9 (s), -72.8 (d, J = 713.3 Hz), -104.3 (dt, J = 23.2, 7.0 Hz), -119.6 (dd, J = 23.2, 11.3 Hz).

HRMS (ESI^+): Mass calculated for $C_{20}H_{17}N_5F_5Ru$ [M+] = 524.0448. Mass found = 524.0442.

m.p. 151–154 °C (Decomposition) (Et_2O)

An oven dried 100 mL Ace pressure tube equipped with a stirrer bar was transferred to an argon filled glove box, then [Ru(*p*-cymene)Cl_2]$_2$ (306 mg, 0.50 mmol, 0.50 equiv), KOAc (196 mg, 2.00 mmol, 1.80 equiv), 2-phenylpyridine (155 mg, 1.10 mmol, 1.00 equiv) and MeOH (4 mL) were added. The tube was sealed, transferred out of the box, placed in an oil bath at 45 ^oC for 16 h. Upon completion, the reaction crude was loaded onto an aluminium oxide (Al_2O_3, neutral) column conditioned with CH_2Cl_2 and quickly eluted with CH_3CN using N_2 instead of compressed air collecting the yellow band. The solution was concentrated under reduced pressure and quickly precipitated with Et_2O quickly to afford the desired complex **5a** (403 mg, 93%) as an orange solid. The complex must then be transferred to a glovebox as it decomposes turning green when exposed to air. The complexes are subjected to quantitative 1 H NMR after their synthesis. If they are of lower purity, then the complex should be dissolved in MeCN inside the glovebox and filtered through a small plug of alumina. Then it should be concentrated under vacuum and crashed out with Et_2O/Pentane (1:1).Data is in accordance with those previously reported.[17] Giving the mono-cyclometallated ruthenium complex **5a** as an orange solid (383 mg, 0.87 mmol, 87%).

p-cymene bound Mono-cyclometallated complex (5a)

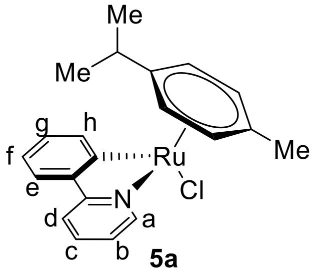

5a

IR v_{max} (neat/cm^{-1}): 3031, 1577, 1452, 1410, 1267, 1121, 1008, 763.

^{1}H NMR (500 MHz, CD_3CN) δ 9.30 (1H, d, J = 5.71 Hz, **H^a**), 8.15 (1H, d, J = 7.6 Hz, **H^d**), 7.85–7.73 (2H, m, Ar**H**), 7.67 (1H, dd, J = 7.8, 1.4 Hz, **H^h**), 7.18–7.09 (2H, m, Ar**H**), 7.00 (1H, td, J = 7.4, 1.2 Hz, **H^f**), 5.67–5.62 (2H, m, Ar**H**), 5.30 (1H, d, J = 5.98 Hz, Ar**H**), 5.01 (1H, d, J = 5.01 Hz), 2.38–2.26 (1H, m, C**H**(CH_3)$_2$), 1.98 (3H, s, C**H**$_3$), 0.91 (3H, d, J = 6.9 Hz, C**H**$_3$), 0.79 (3H, d, J = 6.9 Hz, C**H**$_3$).

^{13}C NMR (126 MHz, CD_3CN) δ 182.9 (C), 165.9 (C), 156.3 (CH), 144.9 (C), 140.9 (CH), 138.0 (CH), 129.6 (CH), 124.5 (CH), 123.0 (CH), 122.6 (CH), 119.5 (CH), 102.4 (C), 100.6 (C), 92.2 (CH), 90.5 (CH), 85.4 (CH), 82.5 (CH), 31.6 (CH), 22.6 (CH_3), 21.8 (CH_3), 18.9 (CH_3).

m.p. 150 – 154 °C (Decomposition) (Et_2O)

7.2. Preparation of Bis-Cyclometallated Ruthenium Complexes

An oven dried microwave vial equipped with a stirrer bar was transferred to a glove box, then the mono-cyclometallated complex **4b** (135 mg, 0.20 mmol, 1.0 equiv), the 2-phenylpyridine (**1p**) (49.0 mg, 0.22 mmol, 1.1 equiv), K_2CO_3 (83 mg, 0.6 mmol, 3.0 equiv), KOAc (10 mg, 0.1 mmol, 0.5 equiv), dry acetone (3.8 mL) were added. The mixture was then heated to 70 ºC and stirred for 16 h. Both solutions were drawn up into one 24 mL syringe with a long needle attached. The residual material on the vial was washed with acetone and drawn into the same syringe. The solution was then filtered with 2 x PTFE filters. The resultant solution was evaporated to dryness. The resultant solid was then dissolved in 2 mL of benzene. Excess hexane was slowly added whilst stirring vigorously. The resultant precipitate was filtered and washed with hexane. The BCRC **6a** was afforded as a dark red solid (60 mg, 0.1 mmol, 48%).

Bis-cycloruthenеated complex (6a)

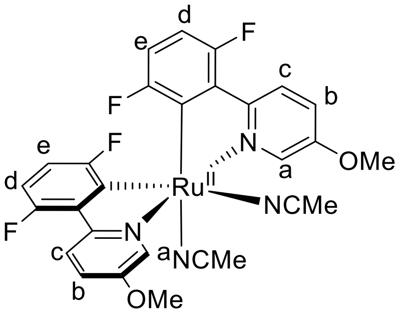

6a

^{1}H NMR (500 MHz, C_6D_6) δ 9.43 (2H, d, *J* = 2.8 Hz, **H^{a}**), 8.54 (2H, dd, *J* = 9.0, 3.0 Hz, **H^{c}**), 6.76 (2H, dd, *J* = 9.0, 3.0 Hz, **H^{b}**), 6.50–6.38 (4H, m, Ar**H$^{d/e}$**), 3.30 (6H, s, OC**H$_3$**), 0.36 (6H, s, C**H$_3$**).

^{13}C NMR (126 MHz, C_6D_6) δ 174.2 (2 x C, dd, *J* = 51.3, 2.6 Hz), 169.2 (2 x C, d, *J* = 225.4 Hz), 159.6 (2 x C, d, *J* = 8.0 Hz), 157.6 (2 x C, d, 248.5 Hz), 153.2 (2 x C, d, *J* = 3.1 Hz), 140.5 (2 x CH), 135.5 (2 x C, *J* = 20.3, 4.0 Hz), 127.0 (2 x C), 112.2 (2 x CH, d, *J* = 22.2 Hz), 116.8 (2 x CH), 112.9 (2 x CH, dd, *J* = 38.4, 15.3 Hz), 106.8 (2 x CH, dd, *J* = 26.0, 10.7 Hz), 55.0 (2 x CH_3), 27.8 (2 x CH_3).

^{19}F^{2} NMR (471 MHz, C_6D_6) δ -105.9 (dt, *J* = 23.9, 5.4 Hz), -123.8 (dd, *J* = 22.1, 11.7 Hz).

HRMS (ESI$^+$): Mass calculated for C28H22N4F4O2Ru [M+] = 624.0722. Mass found = 624.0717.

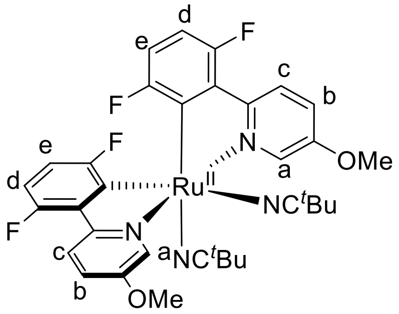

6ab

Inside an argon filled glove box, four oven dried microwave vials were each charged with [Ru(H2O)(tBuCN)5][BF4]2 (142 mg, 0.20 mmol, 1.00 equiv), KOAc (9.80 mg, 0.10 mmol, 0.50

equiv.), K2CO3 (83.0 mg, 0.60 mmol, 3.00 equiv.), arylpyridine 1p (93.0 mg, 0.42 mmol, 2.10 equiv.) and acetone (4 mL, 0.05 M). The mixture was heated at 70 ºC for 16 h with vigorous stirring. All four reaction mixtures were drawn into the same syringe (20 mL) and passed through a PTFE syringe filter (0.2 μm, 17 mm). Note: it was necessary to use multiple filters to pass the entire reaction mixture due to clogging. The filtrate was then evaporated to dryness. The resulting residue was redissolved in benzene (18 mL) and passed through a PTFE filter (0.2 μm, 17 mm). The filtrate was then concentrated to approximately 2mL. With vigorous stirring (1000 rpm), excess hexane was slowly added. The resulting precipitate was isolated by filtration and washed with hexane to give BCRC **6ab** (320 mg, 56 %) as a bright red/orange solid.

^{1}H NMR (700 MHz, C_6D_6) δ 9.37 (2H, d, *J* = 2.9 Hz, **H**^{a}), 8.48 (2H, dd, *J* = 9.1, 2.7 Hz, **H**^{c}), 6.79 (2H, dd, *J* = 9.0, 2.9 Hz, **H**^{b}), 6.42 (2H, ddd, *J* = 11.9, 8.4, 3.7 Hz, **H**^{d}), 6.37 (2H, ddd, *J* = 8.4, 6.7, 3.8 Hz, **H**^{e}), 3.32 (6H, s, OC**H**$_3$), 0.58 (18H, s, CC**H**$_3$).

^{13}C NMR (126 MHz, C_6D_6) δ 173.9 (d, *J* = 51.1 Hz, C), 168.8 (d, *J* = 225.3 Hz, C), 159.3 (d, *J* = 7.5 Hz, C), 157.3 (d, *J* = 246.9 Hz, C), 152.9 (d, *J* = 2.0 Hz, C), 140.0 (CH), 135.1 (dd, *J* = 20.4, 3.8 Hz, C), 126.7 (C), 121.8 (d, *J* = 21.7 Hz, CH), 116.5 (CH), 112.5 (dd, *J* = 34.4, 9.3 Hz, CH), 106.3 (dd, *J* = 27.0, 8.6 Hz, CH), 54.7 (CH$_3$), 27.5 (CH$_3$).

^{19}F NMR (376 MHz, C_6D_6) δ -105.9 (ddd, *J* = 23.4, 6.7, 4.0 Hz), -123.7 – -124.0 (m)

HRMS (ESI$^+$): Mass calculated for $C_{24}H_{16}F_4N_2O_2Ru$ [M-2^{t}BuCN] = 542.0192. Mass found = 542.0188

An oven dried microwave vial equipped with a stirrer bar was transferred to a glove box, then the mono-cyclometallated complex **4c** (135 mg, 0.2 mmol), 2-phenylpyridine (**1t**) (49 mg, 0.22 mmol), K_2CO_3 (83 mg, 0.6 mmol), KOAc (10 mg, 0.1 mmol), dry benzene (3.8 mL) and NMP

(0.1 mL) were added. The mixture was then heated to 80 °C and stirred for 16 h. The solution was drawn up into one 24 mL syringe with a long needle attached. The residual material on the vial was washed with benzene and drawn into the same syringe. The solution was then filtered with 2 x PTFE filters. The resultant solution was evaporated till 2 mL of the mixture remained. Excess hexane was slowly added whilst stirring vigorously. The resultant precipitate was filtered and washed with hexane. The BCRC **9a** was afforded as an orange solid (92 mg, 0.13 mmol, 66%).

Bis-cyclorutheneated complex (9a)

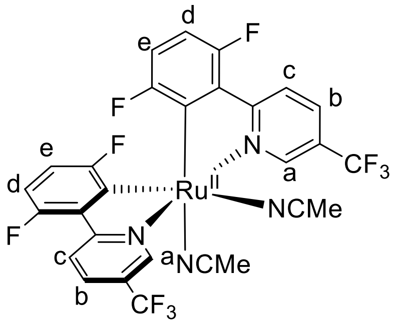

9a

^{1}H NMR (500 MHz, C_6D_6) δ 9.85 (2H, s, **H^{a}**), 8.40 (2H, dd, *J* = 8.7, 3.4 Hz, **H^{b}**), 7.32 (2H, d, *J* = 8.0 Hz, **H^{c}**), 6.37–6.31 (4H, m, Ar**H$^{d/e}$**), 0.32 (6H, s, C**H**$_3$).

^{13}C NMR (176 MHz, C_6D_6) δ 176.5 (C, d, *J* = 49.9 Hz), 169.3 (C, d, *J* = 7.9 Hz), 168.8 (C, d, *J* = 227.0 Hz), 159.0 (C, dd, *J* = 251.6, 2.1 Hz), 147.7 (CH, d, *J* = 5.2 Hz), 134.2 (C, d, *J* = 19.7 Hz), 129.3 (CH), 124.4 (C, q, *J* = 271.2 Hz), 122.4 (C, q, *J* = 32.1 Hz,), 121.8 (CH, d, *J* = 25.3 Hz), 118.8 (C), 115.0 (CH, dd, *J* = 33.9, 10.7 Hz), 107.0 (CH, *J* = 26.3, 8.7 Hz), 48.0 (C), 29.9 (C), 28.8 (2 x CH_3).

^{19}F NMR (471 MHz, C_6D_6) δ -61.7 (s), -105.6 (d, *J* = 20.7 Hz), -119.5 (s).

HRMS (ESI^{+}): Mass calculated for $C_{28}H_{16}N_4F_{10}Ru$ [M+] = 700.0259. Mass found = 700.0258.

8.0.Experimental Section

8.1.Synthesis of 2-Phenylpyridines

2-(4-fluorophenyl)pyridine (1g)

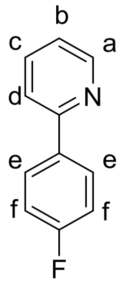

1g

The title compound was synthesised as outlined in general procedure **B** using (4-fluorophenylboronic acid (1.68 g, 12.0 mmol). Purification by flash column chromatography (20% EtOAc/Hexane) afforded **1g** as a colourless amorphous solid (865 mg, 5.0 mmol, 50%).

R$_f$ = 0.47 (1:5 EtOAc:Hexane)

m.p. 46 – 49 °C (hexane)

^{1}H NMR (400 MHz, $CDCl_3$) δ 8.65 (1H, d, *J* = 4.7 Hz, **H^a**), 7.98–7.93 (2H, m, **H^e**), 7.73–7.67 (1H, m, **H^c**), 7.65–7.62 (1H, m, **H^d**), 7.22–7.18 (1H, m, **H^b**), 7.16–7.11 (2H, m, **H^f**).

^{13}C NMR (126 MHz, $CDCl_3$) δ 163.7 (d, *J* = 248.4 Hz, C-F), 156.6 (C), 149.8 (CH), 137.0 (CH), 135.7 (d, *J* = 3.2 Hz, (C)), 128.8 (d, *J* = 8.3 Hz, CH), 122.2 (CH), 120.4 (CH), 115.8 (d, *J* = 21.6 Hz, CH).
^{19}F^2 NMR (400 MHz, $CDCl_3$) δ −113.01 (s).

Data was in accordance with those previously described within the literature.[18]

4-(pyridin-2-yl)benzoate (1e)

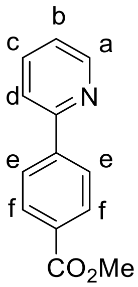

1e

The title compound was synthesised as outlined in general procedure **B** using (4-(methoxycarbonyl)phenyl)boronic acid (6.5 g, 36 mmol). Purification by flash column chromatography (20% EtOAc/Hexane) afforded **1e** as a yellow amorphous solid (1.2 g, 5.6 mmol, 56%).

m.p. 104 – 106 °C (hexane)

^{1}H NMR (400MHz, $CDCl_3$) δ 8.74–8.71 (1H, m, **H^{a}**), 8.16–8.12 (2H, m, **H^{f}**), 8.08–8.05(2H, m, **H^{e}**), 7.80–7.77 (2H, m, Ar**H**), 7.31–7.25 (1H, m, **H^{b}**), 3.94 (3H, s, C**H$_3$**).

^{13}C NMR (101 MHz, $CDCl_3$) δ 167.0 (C), 156.4 (C), 150.0 (CH), 143.7 (C), 137.0 (CH), 130.5 (C), 130.2 (2 x CH), 127.0 (2 x CH), 123.0 (CH), 121.1 (CH), 52.3 (CH_3).

Data was in accordance with those previously described within the literature.[19]

2-(2-methoxyphenyl)pyridine (1k)

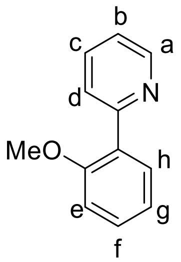

1k

The title compound was synthesised as outlined in general procedure **B** using (2-methoxyphenyl)boronic acid (4.9 g, 36 mmol). Purification by flash column chromatography (20% EtOAc/Hexane) afforded **1k** as an orange oil (2.9 g, 5.0 mmol, 50%).

^{1}H NMR (400MHz, $CDCl_3$) δ 8.72–8.69 (1H, m, **H^a**), 7.81 (1H, dt, J = 7.9, 1.1 Hz, **H^d**), 7.78 (1H, dd, J = 7.5, 1.8 Hz, **H^h**), 7.68 (1H, td, J = 7.9, 1.9 Hz, **H^c**), 7.40–7.34 (1H, m, **H^b**), 7.21–7.17 (1H, m, **H^f**), 7.08 (1H, td, J = 7.5, 1.1 Hz, **H^g**), 7.00 (1H, d, J = 8.3 Hz, **H^e**), 3.75 (3H, s, C**H**$_3$).

^{13}C NMR (101 MHz, $CDCl_3$) δ 156.8 (C), 156.1 (C), 149.3 (CH), 135.5 (CH), 131.1 (CH), 129.9 (CH), 129.1 (C), 125.1 (CH), 121.6 (CH), 120.9 (CH), 111.2 (CH), 55.5 (CH_3).

Data was in accordance with those previously described within the literature.[20]

2-(2,5-difluorophenyl)-5-methoxypyridine (1p)

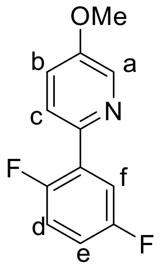

1p

The title compound was synthesised as outlined in general procedure **B** using (2,5 -difluorophenyl)boronic acid (6.2 g, 36 mmol) and 2-bromo-5-methoxypyridine (1.9 g, 10 mmol). Purification by flash column chromatography (5% EtOAc/Hexane) afforded **1p** as a white solid (6.0 g, 7.6 mmol, 76 %).

R*f* = 0.42 (1:9 EtOAc:Hexane)

m.p. = 61-64 °C (hexane)

^{1}H NMR (400 MHz, $CDCl_3$) δ 8.42 (1H, d, *J* = 3.1 Hz, **H^{a}**), 7.78 (1H, dd, *J* = 8.8, 1.9 Hz, **H^{c}**), 7.75–7.69 (1H, m, **H^{f}**), 7.29–7.25 (1H, m, **H^{f}**), 7.13–7.06 (1H, m, **H^{e}**), 7.03–6.96 (1H, m, **H^{d}**), 3.91 (3H, s, OC**H_3**).

^{13}C NMR (101 MHz, $CDCl_3$) δ 159.1 (dd, *J* = 241.8, 2.2 Hz, C), 156.4 (dd, *J* = 244.5, 2.4 Hz, C), 155.3 (d, *J* = 1.0 Hz, C), 144.6 (dd, *J* = 3.0, 1.9 Hz, C), 137.8, 128.5 (dd, *J* = 14.1, 8.0 Hz, CH), 124.8 (d, *J* = 10.8 Hz, CH), 120.8 (CH), 117.4 (dd, *J* = 26.4, 8.6 Hz, CH), 116.8 (dd, *J* = 25.4, 3.7 Hz), 116.1 (dd, *J* = 24.4, 9.0 Hz, CH), 55.8 (CH_3).

^{19}F^{2} NMR (376 MHz, $CDCl_3$) δ -118.9 (d, *J* = 18.2 Hz), -123.4 (d, *J* = 18.0 Hz).

HRMS (ESI^{+}): Mass calculated for $C_{12}H_9F_2NO$ [M+H] = 222.0652. Mass found = 222.0743.

2-(2,5-difluorophenyl)-5-methoxypyridine (1t)

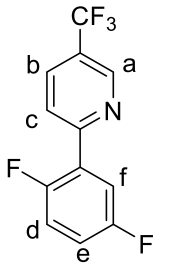

1t

The title compound was synthesised as outlined in general procedure **B** using (2,5 - difluorophenyl)boronic acid (6.2 g, 36 mmol) and 2-bromo-5-trifluoromethylpyridine (2.2 g ,10 mmol). Purification by flash column chromatography (5% EtOAc/Hexane) afforded **1t** as a white solid (2.0 g, 7.6 mmol, 76 %).

$\mathbf{R}_f$ = 0.39 (1:9 EtOAc:Hexane)

m.p. = 65-68 ^{o}C (hexane)

^{1}H NMR (400MHz, $CDCl_3$) δ 9.00–8.96 (1H, m, **H^{a}**), 8.03–7.96 (2H, m, Ar**H**), 7.86–7.79 (1H, m, **H^{c}**), 7.19–7.09 (2H, m, Ar**H**).

^{13}C NMR (101 MHz, $CDCl_3$) δ 159.0 (C, dd, *J* = 285.0, 2.9 Hz), 157.0 (C, dd, *J* = 288.0, 2.9 Hz), 155.5 (C), 146.7 (CH, q, *J* = 4.1 Hz), 134.0 (CH, q, *J* = 3.7 Hz), 127.4 (C, dd, *J* = 13.3, 7.8 Hz), 125.7 (C, q, *J* = 33.7 Hz), 124.1 (CH, d, *J* = 11.9 Hz), 123.7 (C, q, *J* = 272.9 Hz), 118.1 (CH, dd, *J* = 43.1, 9.0 Hz), 117.9 (CH, dd, *J* = 44.5, 8.3 Hz), 117.4 (CH, dd, *J* = 25.5, 3.0 Hz).

^{19}F^{2} NMR (376 MHz, $CDCl_3$) δ -62.4 (s), -117.9 (d, *J* = 18.1 Hz), -122.2 (d, *J* = 18.1 Hz).

HRMS (ESI^{+}): Mass calculated for $C_{12}H_9F_2NO$ [M+H] = 260.0224. Mass found = 260.0501.

8.2. Ruthenium-catalysed Alkylation of Arenes with Epoxides

1-(benzyloxy)-3-(2-(pyridin-2-yl)phenyl)propan-2-ol **(3a)**

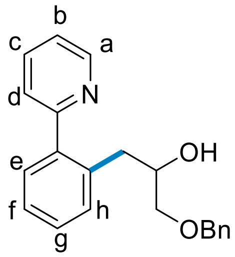

3a

The title compound was synthesised as outlined in general procedure **C** using 2-phenylpyridine (71 µl, 0.5 mmol) and **2c** (0.23 mL, 1.5 mmol). Purification by flash column chromatography (40% EtOAc/Hexane) afforded **3a** as a light brown oil (126 mg, 0.39 mmol, 77%).

R*f* = 0.54 (2:3 EtOAc:Hexane)

^{1}H NMR (400MHz, $CDCl_3$) δ 8.59 (1H, d, *J* = 4.4 Hz, **H**^{a}), 7.84 (1H, td, *J* = 7.9, 1.8 Hz, **H**^{c}), 7.55 (1H, d, *J* = 7.9 Hz, **H**^{d}), 7.43–7.25 (10H, m, Ar**H**), 4.63 (2H, m, PhC**H**$_2$), 4.18–4.07 (1H, m, C**H**OH), 3.65 (1H, dd, *J* = 9.4, 4.8 Hz, C**H**$_a$H$_b$OBn), 3.51 (1H, dd, *J* = 9.4, 6.6Hz, CH$_a$**H**$_b$OBn), 2.95 (1H, dd, *J* = 13.7, 4.0 Hz, CC**H**$_a$H$_b$), 2.89 (1H, dd, *J* = 13.7, 8.9 Hz, CCH$_a$**H**$_b$).

^{13}C NMR (101 MHz, $CDCl_3$) δ 158.8 (C), 147.3 (CH), 139.7 (C), 138.7 (C), 137.9 (CH), 137.7 (C), 131.4 (CH), 130.1 (CH), 129.2 (CH), 128.4 (2 x CH), 127.8 (2 x CH), 127.6 (CH), 126.6 (CH), 124.9 (CH), 122.2 (CH), 75.1 (CH_2), 73.5 (CH_2), 71.9 (CH), 36.9 (CH_2).

HRMS (ESI^+): Mass calculated for $C_{21}H_{21}NO_2$ [M+Na] = 342.1470. Mass found = 342.1453.

IR ν_{max} (neat/cm^{-1}): 3321 (O-H), 1737, 1447, 1301, 1233, 1043, 938, 847.

***1-(benzyloxy)-3-(5-methoxy-2-(pyridin-2-yl)phenyl)propan-2-ol* (3b)**

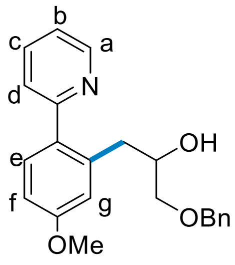

3b

The title compound was synthesised as outlined in general procedure **C** using 2-(*p*-methoxyphenyl)pyridine (93 mg, 0.5 mmol) and **2c** (0.23 mL, 1.5 mmol). Purification by flash column chromatography (40% EtOAc/Hexane) afforded **3b** as a brown oil (90 mg, 0.3 mmol, 60 %).

R_f = 0.48 (2:3 EtOAc:Hexane)

^{1}H NMR (400 MHz, $CDCl_3$) δ 8.57 (1H, d, *J* = 4.8 Hz, **H^a**), 7.86–7.79 (1H, m, **H^c**), 7.53 (1H, d, *J* = 8.9 Hz, **H^e**), 7.43–7.24 (7H, m, Ar**H**), 6.97 (1H, d, *J* = 2.7 Hz, **H^g**), 6.89 (1H, dd, *J* = 8.9, 2.7 Hz, **H^f**), 4.66 (2H, s, PhC$\mathbf{H_2}$), 4.21–4.11 (1H, m, C**H**OH), 3.86 (3H, s, C$\mathbf{H_3}$), 3.71 (1H, dd, *J* = 9.4, 4.8 Hz, C$\mathbf{H_a}$$H_b$OBn), 3.55 (1H, dd, *J* = 9.4, 6.7 Hz, C$H_a$$\mathbf{H_b}$OBn), 2.99–2.89 (2H, m, C$\mathbf{CH_2}$CH).

^{13}C NMR (126 MHz, $CDCl_3$) δ 160.2 (C), 158.6 (C), 147.2 (CH), 139.5 (C), 138.7 (C), 137.7 (CH), 132.6 (C), 131.5 (CH), 128.4 (2 x CH), 127.7 (2 x CH), 127.6 (CH), 124.6 (CH), 121.7 (CH), 116.1 (CH), 112.6 (CH), 75.3 (CH_2), 73.5 (CH_2), 71.9 (CH), 55.4 (CH_3), 37.2 (CH_2).

HRMS (ESI$^+$): Mass calculated for $C_{22}H_{23}NO_3$ [M+Na] = 372.1678. Mass found = 372.1561.

IR ν_{max} (neat/cm^{-1}): 3152 (O-H), 2853, 1592, 1506, 1320, 1074, 820, 697.

***1-(benzyloxy)-3-(5-methyl-2-(pyridin-2-yl)phenyl)propan-2-ol* (3c)**

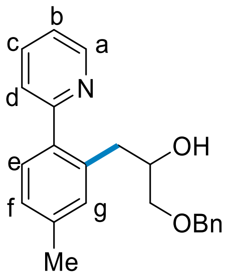

3c

The title compound was synthesised as outlined in general procedure **C** using 2-(*p*-tolyl)pyridine (85 mg, 0.5 mmol) and **2c** (0.23 mL, 1.5 mmol). Purification by flash column chromatography (40% EtOAc/Hexane) afforded **3c** as a brown oil (83 mg, 0.25 mmol, 50 %).

R*f* = 0.57 (2:3 EtOAc:Hexane)

^{1}H NMR (500 MHz, $CDCl_3$) δ 8.56 (1H, d, *J* = 4.8 Hz, **H^a**), 7.81 (1H, td, *J* = 7.7, 1.3 Hz, **H^c**), 7.79–7.65 (1H, br s, O**H**), 7.53 (1H, d, *J* = 7.7, **H^d**), 7.40–7.33 (4H, m, Ar**H**), 7.32–7.25 (3H, m, Ar**H**), 7.24–7.23 (1H, s, **H^g**), 7.13 (1H, d, *J* = 7.7 Hz, **H^f**), 4.67–4.60 (2H, m, PhC**H**$_2$), 4.16–4.09 (1H, m, C**H**OH), 3.67 (1H, dd, *J* = 9.4, 4.8 Hz, C**H**$_a$H$_b$OBn), 3.52 (1H, dd, *J* = 9.4, 6.6 Hz, CH$_a$**H**$_b$OBn), 2.94–2.83 (2H, m, CC**H**$_2$CH), 2.39 (3H, s, C**H**$_3$).

^{13}C NMR (126 MHz, $CDCl_3$) δ 158.9 (C), 147.3 (CH), 139.0 (C), 138.8 (C), 137.7 (CH), 137.6 (C), 137.0 (C), 132.0 (CH), 130.1 (CH), 128.4 (2 x CH), 127.8 (2 x CH), 127.6 (CH), 127.4 (CH), 124.7 (CH), 121.9 (CH), 75.3 (CH_2), 73.5 (CH_2), 72.0 (CH), 36.8 (CH_2), 21.3 (CH_3).

HRMS (ESI$^+$): Mass calculated $C_{22}H_{23}NO_2$ [M+Na] = 356.1729. Mass found = 356.1608.

IR ν_{max} (neat/cm^{-1}): 3152 (O-H), 2853, 1734, 1591, 1453, 1285, 1045, 734.

***1-(benzyloxy)-3-(5-(tert-butyl)-2-(pyridin-2-yl)phenyl)propan-2-ol* (3d)**

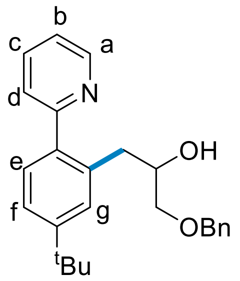

3d

The title compound was synthesised as outlined in general procedure **C** using 2-(4-*tert*-butylphenyl)pyridine (106 mg, 0.50 mmol) and **2c** (0.23 mL, 1.5 mmol). Purification by flash column chromatography (40% EtOAc/Hexane) afforded **3d** as a brown oil (107 mg, 0.29 mmol, 57 %).

R_f = 0.60 (2:3 EtOAc:Hexane)

^{1}H NMR (500 MHz, $CDCl_3$) δ 8.57 (1H, d, *J* = 5.1 Hz, **H^a**), 7.82 (1H, td, *J* = 7.7, 1.8 Hz, **H^c**), 7.81–7.75 (1H, br s, O**H**) 7.79–7.76 (1H, m, **H^f**), 7.55 (1H, d, *J* = 7.7, **H^d**), 7.44–7.24 (8H, m, Ar**H**), 4.64 (2H, s, PhC**H$_2$**), 4.18–4.09 (1H, m, C**H**OH), 3.67 (1H, dd, *J* = 9.4, 4.9 Hz, C**H$_a$**H$_b$OBn), 3.52 (1H, dd, *J* = 9.4, 6.7 Hz, CH$_a$**H$_b$**OBn), 2.94 (2H, m, CC**H**$_2$), 1.36 (9H, s, C**H**$_3$).

^{13}C NMR (126 MHz, $CDCl_3$) δ 158.9 (C), 152.1 (C), 147.3 (C-H), 138.8 (C), 137.8 (C-H), 137.2 (C), 137.1 (C), 129.9 (C-H), 128.5 (2 x C-H), 128.3 (C-H), 127.8 (2 x C-H), 127.6 (C-H), 124.7 (C-H), 123.7 (C-H), 122.0 (C-H), 75.4 (CH_2), 73.8 (CH_2), 72.0 (C-H), 37.2 (CH_2), 34.9 (C), 31.5 (CH_3).

HRMS (ESI$^+$): Mass calculated for $C_{25}H_{29}NO_2$ [M+H] = 376.2198. Mass found = 376.2277.

IR ν_{max} (neat/cm^{-1}): 3167 (O-H), 3048, 1514, 1453, 1362, 1140, 1104, 954, 841.

methyl *3-(3-(benzyloxy)-2-hydroxypropyl)-4-(pyridin-2-yl)benzoate* (3e)

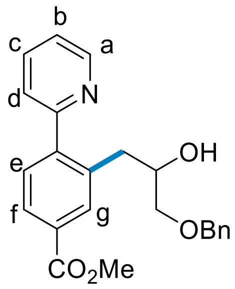

3e

The title compound was synthesised as outlined in general procedure **C** using methyl 4-(pyridin-2-yl)benzoate (107 mg, 0.50 mmol) and **2c** (0.23 mL, 1.5 mmol). Purification by flash column chromatography (40% EtOAc/Hexane) afforded **3e** as a red oil (99 mg, 0.27 mmol, 53 %).

R*f* = 0.42 (2:3 EtOAc:Hexane)

^{1}H NMR (400 MHz, $CDCl_3$) δ 8.50 (1H, m, **H^a**), 8.15–8.10 (1H, m, **H^g**), 7.98 (1H, dd, *J* = 8.0, 1.8 Hz, **H^f**), 7.86 (1H, td, *J* = 7.8, 1.8 Hz, **H^c**), 7.56 (1H, d, *J* = 7.8 Hz, **H^d**), 7.47 (1H, d, *J* = 8.0 Hz, **H^e**), 7.40–7.27 (6H, m, Ar**H**), 4.62 (2H, s, C**H**$_2$Ph), 4.18–4.10 (1H, m, C**H**OH), 3.93 (3H, s, C**H**$_3$), 3.66 (1H, dd, *J* = 9.4, 4.8 Hz, C**H**$_a$H$_b$OBn), 3.51 (1H, dd, *J* = 9.4, 6.4 Hz, CH$_a$**H**$_b$OBn), 3.02–2.90 (2H, m, CC**H**$_2$).

^{13}C NMR (101 MHz, $CDCl_3$) δ 166.8 (C), 157.8 (C), 147.6 (CH), 143.9 (C), 138.6 (C), 138.1 (C), 137.9 (CH), 132.5 (CH), 130.5 (C), 130.2 (CH), 128.4 (2 x CH), 127.7 (2 x CH), 127.6 (CH), 127.5 (CH), 124.9 (CH), 122.7 (CH), 74.8 (CH_2), 73.4 (CH_2), 71.7 (CH), 52.3 (CH_3), 37.0 (CH_2).

HRMS (ESI$^+$): Mass calculated for $C_{23}H_{23}NO_4$ [M+Na] = 400.1627. Mass found = 400.1505.

IR ν_{max} (neat/cm^{-1}): 3193 (O-H), 2931, 1717 (C=O), 1632, 1429, 1289, 1025, 762.

***1-(benzyloxy)-3-(5-chloro-2-(pyridin-2-yl)phenyl)propan-2-ol* (3f)**

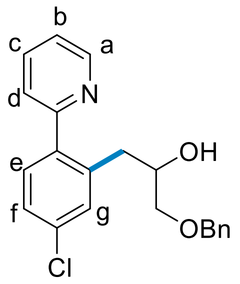

3f

The title compound was synthesised as outlined in general procedure **C** using 2-(4-chloro-butylphenyl)pyridine (95 mg, 0.5 mmol) and **2c** (0.23 mL, 1.5 mmol). Purification by flash column chromatography (1% MeOH/DCM) afforded **3f** as a brown oil (124 mg, 0.35 mmol, 70 %).

R*f* = 0.53 (2:3 EtOAc:Hexane)

^{1}H NMR (400 MHz, $CDCl_3$) δ 8.57 (1H, m, **H**^{a}), 7.83 (1H, td, *J* = 7.8, 1.8 Hz, **H**^{c}), 7.51 (1H, d, *J* = 7.8 Hz, **H**^{d}), 7.42–7.27 (9H, m, Ar**H**), 4.61 (2H, s, C**H**$_2$Ph), 4.10 (1H, m, C**H**OH), 3.64 (1H, dd, *J* = 9.4, 4.7 Hz, C**H**^{a}H^{b}OBn), 3.50 (1H, dd, *J* = 9.4, 6.6 Hz, CH^a**H**^{b}OBn), 2.94–2.85 (2H, m, CC**H**$_2$).

^{13}C NMR (126 MHz, $CDCl_3$) δ 157.8 (C), 147.6 (CH), 139.8 (C), 138.6 (C), 138.2 (C), 138.0 (CH), 135.0 (C), 131.4 (CH), 131.2 (CH), 128.5 (2 x CH), 127.8 (2 x CH), 127.7 (CH), 126.8 (CH), 124.7 (CH), 122.5 (CH), 74.9 (CH_2), 73.5 (CH_2), 71.7 (CH), 36.8 (CH_2).

HRMS (ESI^+): Mass calculated for $C_{21}H_{20}ClNO_2$ [M+Na] = 376.1183. Mass found = 376.1070.

IR ν_{max} (neat/cm^{-1}): 3164 (O-H), 3028, 1562, 1429, 1363, 1120, 1028, 825, 765, 697.

***1-(benzyloxy)-3-(5-fluoro-2-(pyridin-2-yl)phenyl)propan-2-ol* (3g)**

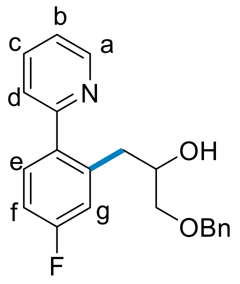

3g

The title compound was synthesised as outlined in general procedure **C** using 2-(4-fluorophenyl)pyridine (87 mg, 0.5 mmol) and **2c** (0.23 mL, 1.5 mmol). Purification by flash column chromatography (40% EtOAc/Hexane) afforded **3g** as a brown oil (212 mg, 0.32 mmol, 63 %).

R_f = 0.60 (2:3 EtOAc:Hexane)

^{1}H NMR (400 MHz, $CDCl_3$) δ 8.57 (1H, d, *J* = 4.8 Hz, **H^a**), 7.83 (1H, td, *J* = 7.7, 1.8 Hz, **H^c**), 7.57–7.48 (1H, m, **H^d**), 7.39–7.27 (7H, m, Ar**H**), 7.11 (1H, m, **H^f**), 7.01 (1H, td, *J* = 7.7, 2.7 Hz, **H^b**), 4.62 (2H, s, C**H**$_2$), 4.15–4.05 (1H, m, C**H**OH), 3.64 (1H, dd, *J* = 9.4, 4.8 Hz, C**H**$_a$H$_b$OBn), 3.50 (1H, dd, *J* = 9.4, 6.7 Hz, CH$_a$**H**$_b$OBn), 2.96–2.84 (2H, m, CC**H**$_2$).

^{13}C NMR (126 MHz, $CDCl_3$) δ 163.1 (C, d, *J* = 248.1 Hz), 158.0 (C), 147.5 (CH), 140.5 (C, d, *J* = 8.5 Hz), 138.6 (C), 137.9 (CH), 136.0 (C, d, *J* = 3.0 Hz), 131.8 (CH, d, *J* = 8.5 Hz), 128.5 (2 x CH), 127.8 (2 x CH), 127.7 (CH), 124.7 (CH), 122.2 (CH), 117.7 (CH, d, *J* = 21.3 Hz), 113.6 (CH, d, *J* = 21.3 Hz), 74.8 (CH_2), 73.5 (CH_2), 71.6 (CH), 37.0 (CH_2).

^{19}F^2 NMR (471 MHz, $CDCl_3$) δ −113.25 (m).

HRMS (ESI$^+$): Mass calculated for $C_{21}H_{20}FNO_2$ [M+Na] = 360.1478. Mass found = 360.1362.

IR v_{max} (neat/cm^{-1}): 3155 (O-H), 3030, 1608, 1470, 1274, 1027, 880, 729.

***1-(benzyloxy)-3-(4-methoxy-2-(pyridin-2-yl)phenyl)propan-2-ol* (3h)**

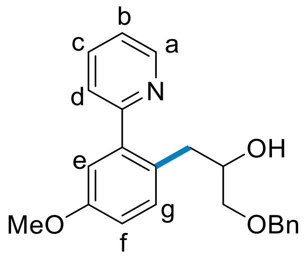

3h

The title compound was synthesised as outlined in general procedure **C** using 2-(3-methoxyphenyl)pyridine (93 mg, 0.5 mmol) and **2c** (0.23 mL, 1.5 mmol). Purification by flash column chromatography (40% EtOAc/Hexane) afforded **3h** as a brown oil (100 mg, 0.31 mmol, 61 %).

R*f* = 0.36 (2:3 EtOAc:Hexane)

^{1}H NMR (400 MHz, $CDCl_3$) δ 8.60 (1H, d, *J* = 5.0 Hz, **H^{a}**), 7.74 (1H, td, *J* = 7.8, 1.8 Hz, **H^{c}**), 7.52–7.48 (1H, m, **H^{d}**), 7.37–7.22 (7H, m, Ar**H**), 7.00 (1H, d, *J* = 7.7 Hz, **H^{f}**), 6.91 (1H, br s, O**H**), 6.86 (1H, d, *J* = 7.7 Hz, **H^{g}**), 4.57 (2H, s, PhC**H$_2$**), 4.07–3.99 (1H, m, C**H**OH), 3.73 (3H, s, C**H$_3$**), 3.56 (1H, dd, *J* = 9.4, 5.0 Hz, C**H$_a$**H$_b$OBn), 3.43 (1H, dd, *J* = 9.4, 6.3 Hz, CH$_a$**H$_b$**OBn), 2.78 (1H, dd, *J* = 13.6, 3.9 Hz, CC**H$_a$**H$_b$), 2.54 (1H, dd, *J* = 13.6, 9.3 Hz, CCH$_a$**H$_b$**).

^{13}C NMR (101 MHz, $CDCl_3$) δ 156.9 (C), 155.1 (C), 147.8 (C-H), 139.4 (C), 138.6 (C), 136.1 (C-H), 129.6 (C-H), 129.3 (C), 128.3 (2 x C-H), 127.7 (2 x C-H), 127.6 (C-H), 127.5 (C-H), 123.0 (C-H), 122.0 (C-H), 109.0 (C-H), 75.0 (CH_2), 73.3 (CH_2), 71.4 (C-H), 55.7 (CH_3), 36.8 (CH_2).

HRMS (ESI^+): Mass calculated for $C_{22}H_{23}NO_3$ [M+H] = 350.1678. Mass found = 350.1756.

IR ν_{max} (neat/cm^{-1}): 3126 (O-H), 3126, 1610, 1576, 1482, 1404, 1221, 1068, 1002, 943.

***1-(benzyloxy)-3-(4-methyl-2-(pyridin-2-yl)phenyl)propan-2-ol* (3i)**

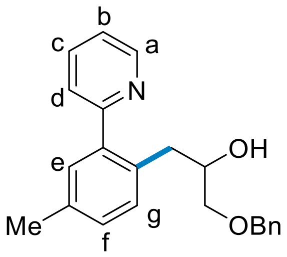

3i

The title compound was synthesised as outlined in general procedure **C** using 2-(3-methylphenyl)pyridine (85 mg, 0.5 mmol) and **2c** (0.23 mL, 1.5 mmol). Purification by flash column chromatography (40% EtOAc/Hexane) afforded **3i** as a brown oil (102 mg, 0.31 mmol, 61 %).

R_f = 0.63 (2:3 EtOAc:Hexane)

^{1}H NMR (400 MHz, $CDCl_3$) δ 8.58–8.55 (1H, m, **H^a**), 7.82 (1H, td, *J* = 7.7, 1.8 Hz, **H^c**), 7.53 (1H, d, *J* = 7.7 Hz, **H^d**), 7.46–7.42 (1H, m, **H^f**), 7.39–7.27 (6H, m, Ar**H**), 7.22–7.18 (2H, m, Ar**H**), 4.65–4.57 (2H, m, C**H_2**Ph), 4.13–4.01 (1H, m, C**H**OH), 3.63 (1H, dd, *J* = 9.4, 4.9 Hz, C**H^a**H^bOBn), 3.49 (1H, dd, *J* = 9.4, 6.6 Hz, CH^a**H^b**OBn), 2.91 (1H, dd, *J* = 13.7, 3.9 Hz, CC**H^a**H^b), 2.83 (1H, dd, *J* = 13.7, 8.9 Hz, CCH^a**H^b**), 2.38 (3H, s, C**H_3**).

^{13}C NMR (126 MHz, $CDCl_3$) δ 159.1 (C), 147.4 (CH), 139.7 (C), 138.8 (C), 137.7 (CH), 136.0 (C), 134.6 (C), 131.3 (CH), 130.8 (CH), 129.9 (CH), 128.4 (2 x CH), 127.8 (2 x CH), 127.6 (CH), 124.8 (CH), 122.1 (CH), 75.1 (CH_2), 73.5 (CH_2), 71.9 (CH), 36.4 (CH_2), 21.1 (CH_3).

HRMS (ESI^+): Mass calculated for $C_{22}H_{23}NO_2$ [M+H] = 333.1729. Mass found = 334.1812.

IR ν_{max} (neat/cm^{-1}): 3171 (O-H), 3028, 1593, 1496, 1363, 1267, 1206, 1118, 1004, 861.

***1-(benzyloxy)-3-(2-(pyridin-2-yl)-4-(trifluoromethyl)phenyl)propan-2-ol* (3j)**

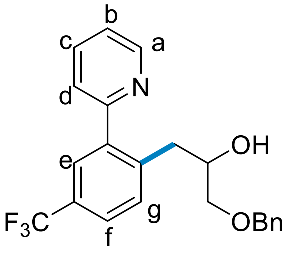

3j

The title compound was synthesised as outlined in general procedure **C** using 2-((3-trifluoromethyl)phenyl)pyridine (111 mg, 0.50 mmol) and **2c** (0.23 mL, 1.5 mmol). Purification by flash column chromatography (40% EtOAc/Hexane) afforded **3j** as a yellow oil (154 mg, 0.4 mmol, 80 %).

R_f = 0.63 (2:3 EtOAc:Hexane)

^{1}H NMR (400 MHz, $CDCl_3$) δ 8.56 (1H, d, *J* = 4.8 Hz, **H^a**), 7.86–7.78 (1H, m, **H^c**), 7.64–7.57 (2H, m, Ar**H**), 7.50 (2H, m, Ar**H**), 7.36–7.23 (6H, m, Ar**H**), 4.59 (2H, s, C**H**$_2$Ph), 4.14–4.06 (1H, m, C**H**OH), 3.62 (1H, dd, *J* = 9.4, 4.7 Hz, C**H**$_a$H$_b$OBn), 3.48 (1H, dd, *J* = 9.4, 6.6 Hz, CH$_a$**H**$_b$OBn), 3.01–2.89 (2H, m, CC**H**$_2$).

^{13}C NMR (126 MHz, $CDCl_3$) δ 157.4 (C), 147.7 (CH), 142.0 (C), 140.3 (C), 138.5 (C), 138.1 (CH), 131.8 (CH), 128.7 (C, q, *J* = 32.6 Hz), 128.4 (2 x CH), 127.8 (2 x CH), 127.6 (CH), 126.8 (CH, q, *J* = 4.2 Hz), 125.5 (CH, q, *J* = 4.2 Hz), 124.8 (CH), 124.2 (C, q, *J* = 272.4 Hz), 122.8 (CH), 74.8 (CH_2), 73.4 (CH_2), 71.6 (CH), 36.8 (CH_2).

^{19}F^2 NMR (471 MHz, $CDCl_3$) δ − 62.37 (s).

HRMS (ESI$^+$): Mass calculated for $C_{22}H_{20}F_3NO_2$ [M+Na] = 410.1446. Mass found = 410.1329

IR v_{max} (neat/cm^{-1}): 3131 (O-H), 2859, 1735, 1496, 1308, 1096, 1003, 732.

***1-(benzyloxy)-3-(3-methoxy-2-(pyridin-2-yl)phenyl)propan-2-ol* (3k)**

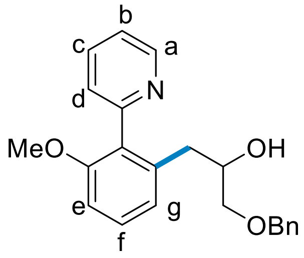

3k

The title compound was synthesised as outlined in general procedure **C** using 2-(2-methoxyphenyl)pyridine (93 mg, 0.50 mmol) and **2c** (0.23 mL, 1.5 mmol). Purification by flash column chromatography (40% EtOAc/Hexane) afforded **3k** as a grey amorphous solid (98 mg, 0.28 mmol, 56 %).

R_f =0.30 (2:3 EtOAc:Hexane)

m.p. 144 °C (decomposition) (Et_2O)

^{1}H NMR (400 MHz, $CDCl_3$) δ 8.55–8.52 (1H, m, **H^a**), 7.70–7.64 (1H, m, **H^c**), 7.44 (1H, d, *J* = 7.9 Hz, **H^d**), 7.30–7.15 (7H, m, Ar**H**), 6.94 (1H, d, *J* = 7.7, **H^e**), 6.79 (1H, d, *J* = 8.1 Hz, **H^g**), 4.51 (2H, s, C**H**$_2$Ph), 4.02–3.93 (1H, m, C**H**OH), 3.65 (3H, m, C**H**$_3$), 3.50 (1H, dd, *J* = 9.4, 5.0 Hz, OC**H**$_a$CH$_b$), 3.37 (1H, dd, *J* = 9.4, 6.3 Hz, OCH$_a$C**H**$_b$), 2.72 (1H, dd, *J* = 13.6, 3.9 Hz, CC**H**$_a$H$_b$), 2.48 (1H, m, CCH$_a$**H**$_b$).

^{13}C NMR (101 MHz, $CDCl_3$) δ 156.9 (C), 155.1 (C), 147.8 (CH), 139.4 (C), 138.6 (C), 136.2 (CH), 129.7 (CH), 129.3 (C), 128.4 (2 x CH), 127.7 (2 x CH), 127.6 (CH), 127.5 (CH), 123.0 (CH), 122.1 (CH), 109.0 (CH), 75.0 (CH_2), 73.4 (CH_2), 71.4 (CH), 55.7 (CH_3), 36.8 (CH_2).

HRMS (ESI$^+$): Mass calculated for $C_{22}H_{23}NO_3$ [M+Na] = 372.1678. Mass found = 372.1558.

IR ν_{max} (neat/cm^{-1}): 3089 (O-H), 1598, 1454, 1403, 1306, 1052, 1035, 801, 736, 646.

***1-(benzyloxy)-3-(3-fluoro-2-(pyridin-2-yl)phenyl)propan-2-ol* (3l)**

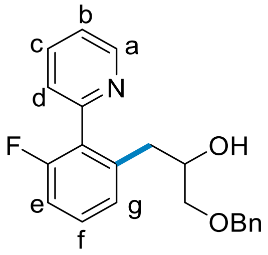

3l

The title compound was synthesised as outlined in general procedure **C** using 2-(2-fluorophenyl)pyridine (87 mg, 0.50 mmol) and **2c** (0.23 mL, 1.5 mmol). Purification by flash column chromatography (40% EtOAc/Hexane) afforded **3l** as a brown oil (118 mg, 0.35 mmol, 70 %).

R_f =0.39 (2:3 EtOAc:Hexane)

^{1}H NMR (500 MHz, $CDCl_3$) δ 8.64–8.62 (1H, m, **H^a**), 7.82 (1H, td, *J* = 7.7, 1.8 Hz, **H^c**), 7.57–7.53 (1H, m, **H^d**), 7.37–7.27 (7H, m, Ar**H**), 7.18 (1H, d, *J* = 7.6 Hz, **H^e**), 7.07–7.02 (1H, m, **H^f**), 4.58 (2H, s, C**H**$_2$Ph), 4.08–4.02 (1H, m, C**H**OH), 3.59 (1H, dd, *J* = 9.4, 4.8 Hz, C**H**$_a$H$_b$O), 3.46 (1H, dd, *J* = 9.4, 6.4 Hz, CH$_a$**H**$_b$O), 2.88 (1H, dd, *J* = 13.7, 3.8 Hz, CC**H**$_a$H$_b$), 2.70 (1H, dd, *J* = 13.7, 9.3 Hz, CCH$_a$**H**$_b$).

^{13}C NMR (101 MHz, $CDCl_3$) δ 171.3 (C), 160.2 (C, d, *J* = 246.1 Hz), 152.4 (C), 148.2 (CH), 140.5 (C, d, *J* = 2.4 Hz), 138.6 (C), 137.0 (CH), 130.0 (CH, d, *J* = 9.5 Hz), 128.4 (2 x CH), 127.7 (2 x CH), 127.6 (CH), 127.2 (CH, d, *J* = 3.9 Hz), 126.5 (CH, d, *J* = 3.9 Hz), 122.8 (CH), 113.6 (CH, d, *J* = 23.0 Hz), 74.8 (CH_2), 73.4 (CH_2), 71.5 (CH), 36.5 (CH_2).

^{19}F^2 NMR (376 MHz, $CDCl_3$) δ −116.67 (m).

HRMS (ESI$^+$): Mass calculated for $C_{21}H_{20}FNO_2$ [M+Na] = 360.1478. Mass found = 360.1360.

IR ν_{max} (neat/cm^{-1}): 3155 (O-H), 2859, 1734, 1614, 1495, 1300 (C-O), 1154, 802, 735, 697.

***1-(benzyloxy)-3-(3,5-difluoro-2-(pyridin-2-yl)phenyl)propan-2-ol* (3m)**

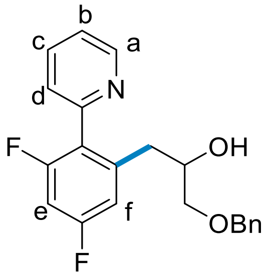

3m

The title compound was synthesised as outlined in general procedure **C** using 2-(2,4-difluorophenyl)pyridine (96 mg, 0.50 mmol) and **2c** (0.23 mL, 1.5 mmol). Purification by flash column chromatography (40% EtOAc/Hexane) afforded **3m** as a brown oil (128 mg, 0.36 mmol, 72%).

R_f = 0.45 (2:3 EtOAc:Hexane)

^{1}H NMR (400 MHz, $CDCl_3$) δ 8.64–8.59 (1H, m, **H^a**), 7.82 (1H, td, *J* = 7.8, 1.8 Hz, **H^c**), 7.54–7.49 (1H, m, **H^d**), 7.37–7.27 (6H, m, Ar**H**), 7.21–7.05 (1H, m, O**H**), 6.96–6.90 (1H, m, **H^e**), 6.84–6.77 (1H, m, **H^f**), 4.58 (2H, s, C**H**$_2$Ph), 4.07–3.99 (1H, m, C**H**OH), 3.59 (1H, dd, *J* = 9.4, 4.7 Hz, C**H^a**H^{b}O), 3.45 (1H, dd, *J* = 9.4, 6.5 Hz, CH^a**H^b**O), 2.84 (1H, dd, *J* = 13.6, 3.9 Hz, CC**H^a**H^b), 2.73 (1H, dd, *J* = 13.6, 9.3 Hz, CCH^a**H^b**).

^{13}C NMR (126 MHz, $CDCl_3$) δ 162.5 (C, dd, *J* = 279.3, 13.7 Hz), 160.6 (C, dd, *J* = 279.3, 13.7 Hz), 151.6 (C), 148.3 (CH), 142.5 (C, dd, *J* = 9.0, 3.2 Hz), 138.5 (C), 137.2 (CH), 128.5 (2 x CH), 127.8 (2 x CH), 127.7 (CH), 127.2 (CH, d, *J* = 4.1 Hz), 124.2 (C, dd, *J* = 3.2, 14.1 Hz), 122.9 (CH), 113.4 (CH, dd, *J* = 20.9, 3.2 Hz), 102.3 (CH, t, *J* = 26.2 Hz), 74.6 (CH_2), 73.5 (CH_2), 71.3 (CH), 36.8 (CH_2).

^{19}F^2 NMR (376 MHz, $CDCl_3$) δ -109.45 (q, *J* = 8.4 Hz), -112.4 (t, *J* = 7.6 Hz).

HRMS (ESI$^+$): Mass calculated for [M+Na] = 378.1384. Mass found = 378.1279.

IR v_{max} (neat/cm^{-1}): 3410 (O-H), 3058, 2860, 2227, 1720, 1559, 1267, 1098, 1028, 778.

methyl 6-(2-(3-(benzyloxy)-2-hydroxypropyl)-4,6-difluorophenyl)nicotinate **(3n)**

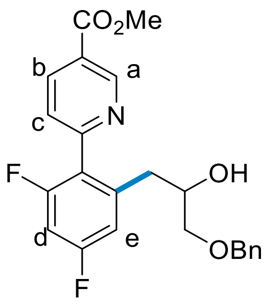

3n

The title compound was synthesised as outlined in general procedure **C** using methyl 6-(2,4-difluorophenyl)nicotinate (125 mg, 0.50 mmol) and **2c** (0.23 mL, 1.5 mmol). Purification by flash column chromatography (40% EtOAc/Hexane) afforded **3n** as a brown oil (163 mg, 0.4 mmol, 79 %).

R*f* = 0.58 (2:3 EtOAc:Hexane)

^{1}H NMR (400 MHz, $CDCl_3$) δ 9.25–9.22 (1H, m, **H^a**), 8.41 (1H, dd, *J* = 8.2, 2.2 Hz, **H^b**), 7.63–7.58 (1H, m, **H^c**), 7.37–7.27 (5H, m, Ar**H**), 6.99–6.93 (1H, m, **H^e**), 6.86–6.78 (1H, m, **H^e**), 6.18 (1H, d, *J* = 5.5 Hz, O**H**), 4.57 (2H, s, C**H$_2$**Ph), 4.08–4.00 (1H, m, C**H**OH), 3.98 (3H, s, C**H$_3$**), 3.57 (1H, dd, *J* = 9.4, 4.8 Hz, C**H^a**H^{b}O), 3.45 (1H, dd, *J* = 9.5, 6.2 Hz, CH^a**H^b**O), 2.84 (1H, dd, *J* = 13.7, 4.0 Hz, CC**H^a**H^b), 2.72 (1H, dd, *J* = 13.7, 9.3 Hz, CCH^a**H^b**).

^{13}C NMR (101 MHz, $CDCl_3$) δ 165.3 (C), 163.0 (C, dd, *J* = 241.8, 13.2 Hz), 160.4 (C, dd, *J* = 250.7, 12.5 Hz), 155.8 (C), 149.6 (CH), 142.6 (C, dd, *J* = 9.3, 3.2 Hz), 138.4 (C), 138.1 (CH), 128.5 (2 x CH), 127.8 (3 x CH), 126.8 (CH, d, *J* = 4.4 Hz), 125.2 (C), 123.4 (C), 113.7 (CH, dd, *J* = 21.3, 3.5 Hz), 102.5 (CH, t, *J* = 26.2 Hz), 74.5 (CH_2), 73.6 (CH_2), 71.4 (CH), 52.7 (CH_3), 36.8 (CH_2).

$^{19}F^2$ NMR (376 MHz, $CDCl_3$) δ -108.2 (q, *J* = 8.4 Hz), -111.9 (t, *J* = 9.3 Hz).

HRMS (ESI^+): Mass calculated for [M+Na] = 436.1439. Mass found = 436.1333.

IR ν_{max} (neat/cm^{-1}): 3233 (O-H), 3031, 2859, 2106, 1726 (C=O), 1469, 1193, 1036, 910, 798.

***1-(benzyloxy)-3-(3,5-difluoro-2-(5-methoxypyridin-2-yl)phenyl)propan-2-ol* (3o)**

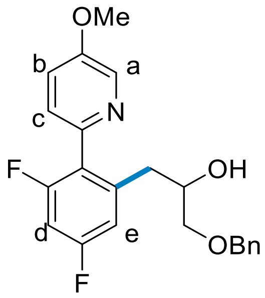

3o

The title compound was synthesised as outlined in general procedure **C** using 2-(2,4-difluorophenyl)-5-methoxypyridine (111 mg, 0.50 mmol) and **2c** (0.23 mL, 1.5 mmol). Purification by flash column chromatography (40% EtOAc/Hexane) afforded **3o** as a brown oil (144 mg, 0.38 mmol, 75 %).

$\mathbf{R}_f$ = 0.45 (2:3 EtOAc:Hexane)

^{1}H NMR (400 MHz, $CDCl_3$) δ 8.31 (1H, d, *J* = 2.9 Hz, **H^a**), 7.44 (1H, dd, *J* = 8.7, 3.5 Hz, **H^b**), 7.37–7.27 (6H, m, Ar**H**), 6.94–6.88 (1H, m, **H^e**), 6.82–6.74 (1H, m, **H^d**), 4.59 (2H, s, C**H**$_2$Ph), 4.07–4.00 (1H, m, C**H**OH), 3.88 (3H, s, C**H**$_3$), 3.59 (1H, dd, *J* = 9.4, 4.7 Hz, C**H^a**H^{b}O), 3.45 (1H, dd, *J* = 9.4, 6.4 Hz, CH^a**H^b**O), 2.83 (1H, dd, *J* = 13.6, 4.0 Hz, CC**H^a**H^b), 2.73 (1H, dd, *J* = 13.6, 9.2 Hz, CCH^a**H^b**).

^{13}C NMR (126 MHz, $CDCl_3$) δ 162.3 (C, dd, *J* = 246.0, 13.3 Hz), 160.5 (C, dd, *J* = 246.0, 13.3 Hz), 155.2 (C), 143.7 (C), 142.4 (C, dd, *J* = 9.1, 3.4 Hz), 138.5 (C), 135.5 (CH), 128.5 (2 x CH), 127.7 (2 x CH), 127.7 (CH) 127.4 (CH, d, *J* = 4.0 Hz), 123.9 (C, dd, *J* = 14.2, 3.8 Hz), 122.0 (CH), 113.3 (CH, dd, *J* = 20.9, 3.5 Hz), 102.9 (CH, t, *J* = 26.5 Hz), 74.7 (CH_2), 73.5 (CH_2), 71.4 (CH), 55.6 (CH_3), 36.6 (CH_2).

^{19}F^2 NMR (376 MHz, $CDCl_3$) δ -110.2 (q, *J* = 8.4 Hz), -112.4 (t, *J* = 8.8 Hz).

HRMS (ESI$^+$): Mass calculated for [M+Na] = 408.1489. Mass found = 408.1381.

IR ν_{max} (neat/cm^{-1}): 3211 (O-H), 3031, 2859, 2325, 1495, 1320, 1028, 909, 736, 611.

***1-(benzyloxy)-3-(3,6-difluoro-2-(5-methoxypyridin-2-yl)phenyl)propan-2-ol* (3p)**

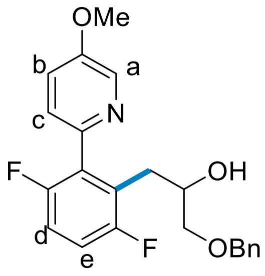

3p

The title compound was synthesised as outlined in general procedure **C** using 2-(2,5-difluorophenyl)-5-methoxypyridine (44 mg, 0.50 mmol) and **2c** (0.23 mL, 1.5 mmol). Purification by flash column chromatography (40% EtOAc/Hexane) afforded **3p** as a brown oil (54 mg, 0.14 mmol, 28 %).

R_f = 0.51 (2:3 EtOAc:Hexane)

^{1}H NMR (500 MHz, $CDCl_3$) δ 8.26 (1H, d, *J* = 2.8 Hz, **H^a**), 7.43 (1H, dd, *J* = 8.7, 3.6 Hz, **H**^{b}), 7.32–7.18 (5H, m, Ar**H**), 7.05–6.99 (1H, m, Ar**H**), 6.99–6.93 (1H, m, Ar**H**), 6.83 (1H, d, *J* = 6.2 Hz, **H^c**), 4.54 (2H, s, C**H**$_2$Ph), 4.13–4.04 (1H, m, C**H**OH), 3.82 (3H, s, C**H**$_3$), 3.54 (1H, dd, *J* = 9.6, 4.6 Hz, C**H^a**H^{b}OBn), 3.47 (1H, dd, *J* = 9.6, 4.6 Hz, CH^a**H^b**OBn), 2.97 (1H, d, *J* = 13.7 Hz, CC**H^a**H^b), 2.57 (1H, m, CCH^a**H^b**).

^{13}C NMR (101 MHz, $CDCl_3$) δ 158.0 (C, dd, *J* = 242.1, 2.9 Hz), 156.1 (C, dd, *J* = 242.1, 3.9 Hz), 155.3 (C), 143.5 (C, d, *J* = 2.3 Hz), 138.7 (C), 135.6 (CH), 129.0 (C, dd, *J* = 15.8, 5.1 Hz), 128.4 (2 x CH), 127.6 (2 x CH), 127.6 (C, dd, J = 18.2, 2.4 Hz), 127.5 (2 x CH), 122.0 (CH), 116.1 (CH, dd, *J* = 25.6, 9.2 Hz), 114.5 (CH, dd, *J* = 25.6, 9.2 Hz), 75.5 (CH_2), 73.4 (CH_2), 69.6 (CH), 55.8 (CH_3), 31.0 (CH_2).

^{19}F^2 NMR (376 MHz, $CDCl_3$) δ -120.2 (dd, *J* = 13.3, 6.3 Hz), -121.6 (dd, *J* = 13.3, 4.7 Hz).

HRMS (ESI$^+$): Mass calculated for [M+Na] = 408.1384. Mass found = 408.1490

IR ν_{max} (neat/cm^{-1}): 3246 (O-H), 2917, 1732, 1605, 1241, 1006, 785, 721

5-(2-(3-(benzyloxy)-2-hydroxypropyl)phenyl)-7-chloro-1-methyl-1,3-dihydro-2H-benzo[e][1,4]diazepin-2-one (3q)

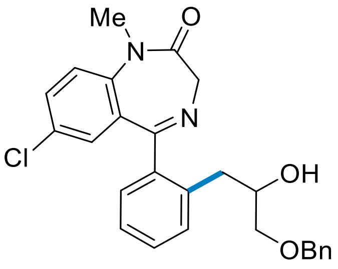

3q

The title compound was synthesised as outlined in general procedure **C** using diazapam (57 mg, 0.20 mmol) and **2c** (0.23 mL, 1.5 mmol). Purification by flash column chromatography (40% EtOAc/Hexane) afforded **3q** as a brown oil (34 mg, 0.08 mmol 38%).

The title compound was synthesised as outlined in general procedure **D** using diazapam (57 mg, 0.20 mmol) and **2c** (0.23 mL, 1.5 mmol). Purification by flash column chromatography (40% EtOAc/Hexane) afforded **3q** as a brown oil (45 mg, 0.1 mmol, 50%).

R*f* = 0.18 (2:3 EtOAc:Hexane)

^{1}H NMR (500 MHz, $CDCl_3$) δ 7.53–7.47 (1H, m, **H^{b}**), 7.45–7.40 (2H, m, **H^{c}**), 7.39–7.12 (5H, m, Ar**H**), 7.22–7.09 (2H, m, Ar**H**), 7.04 (1H, d, *J* = 2.5 Hz, Ar**H**), 6.95 (1H, d, J = 7.7 Hz, Ar**H**), 4.87–4.75 (1H, dd, *J* = 26.5, 11.0 Hz, C(O)C**H^{a}**H^{b}), 4.66–4.52 (2H, m, C**H**$_2$Ph), 4.03–3.94 (1H, m, C**H**OH), 3.76 (1H, dd, *J* = 32.1, 11.0 Hz, C(O)CH^{a}**H^{b}**), 3.62 (1H, dd, *J* = 9.5, 4.4 Hz, C**H^{a}**H^{b}O), 3.51 (1H, dd, *J* = 9.5, 5.8 Hz, CH^{a}**H^{b}**O), 3.41 (3H, s, C**H**$_3$), 3.06–2.83 (2H, m, CC**H$_2$**).

^{13}C NMR (126 MHz, $CDCl_3$) δ 170.3 (C), 169.3 (C), 142.6 (C), 139.6 (C), 138.7 (C), 138.1 (C), 132.1 (CH), 131.5 (C), 131.4 (CH), 131.2 (CH), 130.5 (CH), 130.3 (CH), 129.7 (C), 128.5 (2 x CH), 127.8 (2 x CH), 127.7 (CH), 126.1 (CH), 122.6 (CH), 75.5 (CH_2), 73.7 (CH_2), 72.7 (CH), 56.0 (CH_2), 37.4 (CH_2), 35.2 (CH_3).

HRMS (ESI^+): Mass calculated for [$M+H^+$] = 449.1554. Mass found = 449.1596.

IR ν_{max} (neat/cm^{-1}):3208 (O-H), 2984, 1737, 1447, 1233, 1043, 786, 634.

2-(2-(3-(benzyloxy)propyl)-3,6-difluorophenyl)-5-methoxypyridine (3pa)

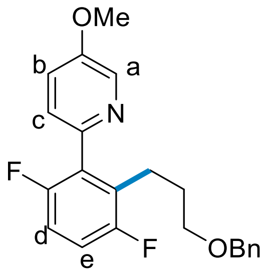

3pa

An oven dried 10 mL microwave vial equipped with a stirrer bar was charged with sodium iodide (30 mg, 0.2 mmol, 1.0 equiv), [(*p*-cymene)$RuCl_2$]$_2$ (6.0 mg, 0.01 mmol, 5.0 mol%), 2-(2,5-difluorophenyl)-5-(methoxy)pyridine (44 mg, 0.20 mmol, 1.0 equiv), K_2CO_3 (23 mg, 0.2 mmol, 1.0 equiv) and KOAc (10 mg, 0.1 mmol, 0.5 equiv) and purged with nitrogen for 10 minutes. **2c** (92 µL, 0.60 mmol) and ethyl acetate (0.35 mL) were added followed by cyclobutanecarboxylic acid (5.0 µL, 0.06 mmol, 30 mol%). The vial was stirred for 24 h under a 440 nm LED. Purification by solid flash column chromatography (10% EtOAc/Hexane) afforded **3pa** as a colourless oil (9.0 mg, 0.22 mmol, 44 %).

R_f = 0.23 (1:9 EtOAc/Hexnae)

^{1}H NMR (400 MHz, $CDCl_3$) δ 8.40 (1H, d, *J* = 2.8 Hz, **H^a**), 7.39–7.23 (7H, m, Ar**H**), 7.07–7.00 (1H, m, **H^d**), 6.99–6.93 (1H, m, **H^e**), 4.40 (2H, s, C**H**$_2$Ph), 3.87 (3H, s, C**H**$_3$), 3.39 (2H, t, *J* = 6.4 Hz, CC**H**$_2$), 2.71–2.66 (2H, m, C**H**$_2$O), 1.82–1.74 (2H, m, CH_2C**H**$_2$$CH_2$).

^{13}C NMR (126 MHz, $CDCl_3$) δ 157.6 (C, d, *J* = 241.1 Hz), 156.2 (C, d, *J* = 246.0 Hz), 155.0 (C), 145.2 (C, d, *J* = 2.1 Hz), 138.7 (C), 137.3 (CH), 130.3 (C, dd, *J* = 19.0, 2.8 Hz), 128.4 (2 x CH), 127.5 (2 x CH), 127.5 (CH), 125.9 (CH, d, *J* = 2.6 Hz), 120.8 (CH), 115.8 (CH, dd, *J* = 26.3, 9.1 Hz), 113.8 (CH, dd, *J* = 26.3, 9.1 Hz), 72.6 (CH_2), 69.9 (CH_2), 55.7 (CH_2), 29.9 (CH_2), 23.1 (CH_3).

^{19}F NMR (400MHz, $CDCl_3$) δ -121.9 (dd, *J* = 17.4, 4.3 Hz), -122.3 (dd, *J* = 17.6, 4.8 Hz).

HRMS (ESI$^+$): Mass calculated for [M+Na] = 392.1540. Mass found = 392.1433.

IR v_{max} (neat/cm^{-1}): 2896, 1642 (C=C), 1325, 1159, 965, 745, 623, 598.

methyl *4-(3-(benzyloxy)-2-hydroxypropyl)-3-(1H-pyrazol-1-yl)benzoate* (3r)

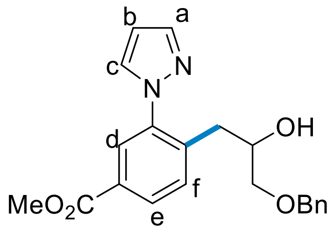

3r

The title compound was synthesised as outlined in general procedure **C** using methyl 3-pyrazolbenzoate (85 mg, 0.50 mmol) and **2c** (0.23 mL, 1.5 mmol). Purification by flash column chromatography (40% EtOAc/Hexane) afforded **3r** as a yellow oil (120 mg, 0.33 mmol, 66 %).

$\mathbf{R}_{f}$ = 0.63 (2:3 EtOAc:Hexane)

^{1}H NMR (400 MHz, $CDCl_3$) δ 7.96–7.91 (1H, m, **H^a**), 7.88–7.84 (1H, m, **H^d**), 7.65–7.61 (2H, m, Ar**H**), 7.40 (1H, d, *J* = 8.0 Hz, **H^f**), 7.30–7.18 (5H, m, Ar**H**), 6.42–6.38 (1H, m, **H^b**), 4.52 (H, br s, OH), 4.50 (2H, s, PhC**H**$_2$), 4.01–3.91 (1H, m, C**H**OH), 3.83 (3H, s, C**H**$_3$), 3.48 (1H, dd, *J* = 9.5, 5.2 Hz, C**H**$_a$H$_b$OBn), 3.37 (1H, dd, *J* = 9.5, 6.1 Hz, CH$_a$**H**$_b$OBn), 2.84 (1H, dd, *J* = 13.6, 3.9 Hz, CC**H**$_a$H$_b$), 2.72 (1H, dd, *J* = 13.6, 8.9 Hz, CCH$_a$**H**$_b$).

^{13}C NMR (101 MHz, $CDCl_3$) δ 165.9 (C), 140.4 (C), 139.9 (C), 139.8 (C), 138.2 (C), 132.0 (CH), 131.1 (CH), 129.3 (CH), 129.3 (CH), 128.4 (2 x CH), 127.7 (2 x CH), 127.6 (CH), 126.5 (CH), 107.3 (CH), 74.3 (CH_2), 73.3 (CH_2), 70.9 (CH), 52.3 (CH_3), 35.2 (CH_2).

HRMS (ESI$^+$): Mass calculated for $C_{21}H_{22}N_2O_4$ [M+Na] = 389.1580. Mass found = 389.1456.

IR ν_{max} (neat/cm^{-1}): 3272 (O-H), 2859, 1719, 1614, 1452, 1288, 1112, 761.

***1-(benzyloxy)-3-(3-fluoro-2-(pyrimidin-2-yl)phenyl)propan-2-ol* (3s)**

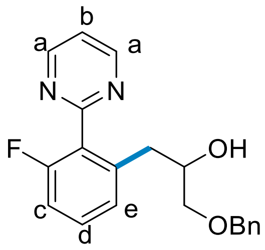

3s

The title compound was synthesised as outlined in general procedure **C** using 2-(2-fluorophenyl)pyrimidine (87 mg, 0.50 mmol) and **2c** (0.23 mL, 1.5 mmol). Purification by flash column chromatography (40% EtOAc/Hexane) afforded **3s** as a brown oil (125 mg, 0.35 mmol, 70 %).

R*f* = 0.27 (2:3 EtOAc:Hexane)

^{1}H NMR (400 MHz, $CDCl_3$) δ 8.77 (2H, d, *J* = 5.0 Hz, **H^a**), 7.36–7.19 (7H, m, Ar**H**), 7.11 (1H, d, *J* = 7.7 Hz, **H^c**), 7.05–6.98 (1H, m, **H^d**), 5.35 (1H, d, *J* = 5.4 Hz, O**H**), 4.49 (2H, s, C**H**$_2$), 4.01–3.92 (1H, m, C**H**OH), 3.45 (1H, dd, *J* = 9.4, 4.9 Hz, C**H**$_a$H$_b$O), 3.40 (1H, dd, *J* = 9.4, 5.5 Hz, CH$_a$**H**$_b$O), 2.81 (1H, dd, *J* = 13.9, 4.4 Hz, CC**H**$_a$H$_b$), 2.67 (1H, dd, *J* = 13.9, 9.0 Hz, CCH$_a$**H**$_b$).

^{13}C NMR (101 MHz, $CDCl_3$) δ 162.9 (C), 160.7 (C, d, *J* = 248.1 Hz), 157.0 (2 x CH), 139.8 (C, d, *J* = 2.5 Hz), 138.4 (C), 130.8 (CH, d, *J* = 11.1 Hz), 128.5 (2 x CH), 127.8 (2 x CH), 127.7 (CH), 127.4 (C, d, *J* = 11.1 Hz), 126.2 (CH, d, *J* = 2.5 Hz), 119.6 (CH), 114.1 (CH, d, *J* = 21.9 Hz), 74.3 (CH_2), 73.4 (CH_2), 71.6 (CH), 36.6 (CH_2).

^{19}F^2 NMR (471 MHz, $CDCl_3$) δ −116.24 (dd, *J* = 11.0, 6.5 Hz).

HRMS (ESI$^+$): Mass calculated for $C_{20}H_{19}FN_2O_2$ [M+Na] = 361.1431. Mass found = 361.1310.

IR ν_{max} (neat/cm^{-1}): 3236 (O-H), 2859, 1734, 1558, 1363, 1309 (C-O), 1050, 817, 797, 697.

1-(benzyloxy)-3-(3,5-difluoro-2-(5-(trifluoromethyl)pyridin-2-yl)phenyl)propan-2-ol (8g)

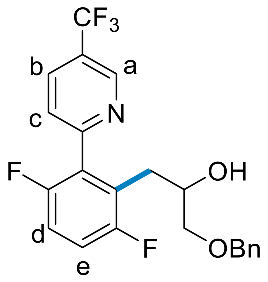

3t

An oven dried 10 mL microwave vial equipped with a stirrer bar was charged with sodium iodide (30 mg, 0.20 mmol, 1.0 equiv), [(*p*-cymene)$RuCl_2$]$_2$ (5.0 mg, 0.01 mmol, 5.0 mol%) and purged with nitrogen for 10 minutes, phenylpyridine **1t** (130 mg, 0.20 mmol, 1.00 equiv), **2c** (93 µL, 0.6 mmol, 3.0 equiv) and ethyl acetate (0.875 mL) were added followed by cyclobutanecarboxylic acid (5.0 µL, 0.06 mmol, 30 mol%). The vial was stirred for 24 h under a 525 - 530 nm LED. Purification by flash column chromatography (10% EtOAc/Hexane) afforded **8g** as a brown oil (21 mg, 0.05 mmol, 24 %).

The title compound was synthesised as outlined in general procedure **D** using phenylpyridine **1t** (130 mg, 0.20 mmol, 1.00 equiv) and **2c** (93 µL, 0.6 mmol). Purification by flash column chromatography (10% EtOAc/Hexane) afforded **8g** as a brown oil (17 mg, 0.04 mmol, 20 %).

$\mathbf{R_f}$ = 0.42 (2:3 EtOAc/Hexane)

^{1}H NMR (400 MHz, $CDCl_3$) δ 8.86 (1H, s, **H**^{a}), 8.02 (1H, d, *J* = 8.5 Hz, **H**^{b}), 7.68–7.61 (1H, m, **H**^{c}), 7.32–7.19 (5H, m, Ar**H**), 7.14–7.07 (1H, m, **H**^{d}), 7.05–6.98 (1H, m, **H**^{e}), 4.52 (2H, s, C**H**$_2$Ph), 4.13–4.05 (1H, m, C**H**OH), 3.54–3.46 (2H, m, OC**H**$_2$C), 2.96 (1H, dd, *J* = 13.8, 3.0 Hz, CC**H**^{a}H^{b}C), 2.62–2.53 (1H, m, CCH^a**H**^{b}C).

^{13}C NMR (126 MHz, $CDCl_3$) δ 157.0 (C, dd, *J* = 482.2, 3.8 Hz), 156.9 (C, t, *J* = 3.3 Hz), 155.1 (C), 145.3 (CH, q, *J* = 4.4 Hz), 138.3 (C), 134.3 (CH, q, *J* = 3.4 Hz), 128.4 (2 x CH), 128.0 (C, dd, *J* = 15.1, 7.3 Hz), 127.7 (CH), 127.6 (C), 127.6 (2 x CH), 127.2 (CH, d, *J* = 4.5 Hz), 126.0

(C, q, *J* = 34.5 Hz), 123.2 (C, q, *J* = 274.2 Hz), 117.4 (CH, dd, *J* = 24.8, 8.4 Hz), 114.9 (CH, dd, *J* = 25.1, 9.4 Hz), 75.0 (CH_2), 73.4 (CH_2), 69.6 (CH), 30.6 (CH_2).

$^{19}F^2$ NMR (376 MHz, $CDCl_3$) δ -62.4 (s), -119.4 (dd, *J* = 15.9, 7.8 Hz), -121.2 (dd, 13.6, 4.7 Hz).

HRMS (ESI^+): Mass calculated for [M+Na] = 446.1258. Mass found = 446.1168.

IR ν_{max} (neat/cm^{-1}): 3354 (O-H), 2891, 1598, 1428, 1187, 845, 542, 498.

***1-(4-methoxyphenethoxy)-3-(2-(pyridin-2-yl)phenyl)propan-2-ol* (7a)**

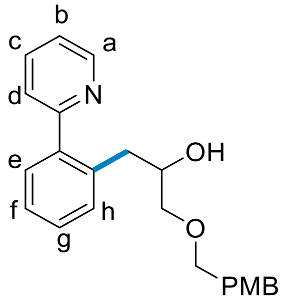

7a

The title compound was synthesised as outlined in general procedure **C** using 2-phenylpyridine (75 µL, 0.5 mmol) and 2-((4-methoxyphenethoxy)methyl)oxirane (291 mg, 1.50 mmol). Purification by flash column chromatography (40% EtOAc/Hexane) afforded **7a** as a brown oil (100 mg, 0.31 mmol, 61 %).

R_f = 0.5 (2:3 EtOAc:Hexane)

^1H NMR (400 MHz, $CDCl_3$) δ 8.60–8.56 (1H, m, **H^a**), 7.83 (1H, td, *J* = 7.7, 1.8 Hz, **H^c**), 7.56–7.51 (1H, m, **H^d**), 7.41–7.27 (7H, m, Ar**H**), 6.91–6.86 (2H, m, Ar**H**), 4.59–4.51 (2H, m, C**H_2**Ph), 4.14–4.06 (1H, m, C**H**OH), 3.81 (3H, s, OC**H_3**), 3.61 (1H, dd, *J* = 9.4, 4.9 Hz, C**H^a**H^bO), 3.47 (1H, dd, *J* = 9.4, 6.7 Hz, CH^a**H^b**O), 2.94 (1H, dd, *J* = 13.7, 3.9 Hz, CC**H^a**H^b), 2.86 (1H, dd, *J* = 13.7, 9.0 Hz, CCH^a**H^b**),

^{13}C NMR (101 MHz, $CDCl_3$) δ 159.3 (C), 159.0 (C), 147.4 (CH), 139.8 (C), 137.8 (CH), 137.7 (C), 131.4 (CH), 130.8 (C), 130.1 (CH), 129.4 (2 x CH), 129.1 (CH), 126.5 (CH), 124.8 (CH), 122.2 (CH), 113.8 (2 x CH), 74.8 (CH_2), 73.1 (CH_2), 71.9 (CH), 55.4 (CH_3), 36.9 (CH_2).

HRMS (ESI^+): Mass calculated for $C_{23}H_{25}NO_3$ [M+Na] = 372.1678. Mass found = 372.1562

IR ν_{max} (neat/cm^{-1}): 3165 (O-H), 2855, 1611, 1511, 1442, 1245, 1173, 1091, 942, 889.

***1-((3-methylbenzyl)oxy)-3-(2-(pyridin-2-yl)phenyl)propan-2-ol* (7b)**

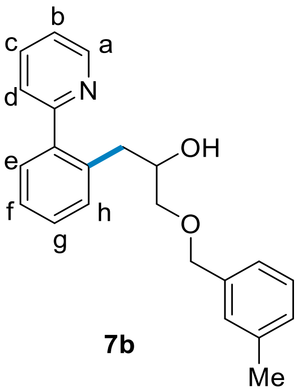

7b

The title compound was synthesised as outlined in general procedure **C** using 2-phenylpyridine (75 µL, 0.5 mmol) 2-(((3-methylbenzyl)oxy)methyl)oxirane (267 mg, 1.50 mmol). Purification by flash column chromatography (40% EtOAc/Hexane) afforded **7b** as a brown oil (100 mg, 0.3 mmol, 60 %).

R*f* = 0.65 (2:3 EtOAc:Hexane)

^{1}H NMR (500 MHz, $CDCl_3$) δ 8.61–8.56 (1H, m, **H^a**), 7.83 (1H, td, *J* = 7.7, 1.8 Hz, **H^c**), 7.54 (1H, d, *J* = 7.7 Hz, **H^d**), 7.42–7.37 (3H, m, Ar**H**), 7.34–7.07 (6H, m, Ar**H**), 4.58 (2H, m, C**H$_2$**Ar), 4.16–4.06 (1H, m, C**H**OH), 3.64 (1H, dd, *J* = 9.4, 4.9 Hz, C**H^a**H^{b}O), 3.50 (1H, dd, *J* = 9.4, 6.6 Hz, CH^a**H^b**O), 2.95 (1H, dd, *J* = 13.6, 3.9 Hz, CC**H^a**H^b), 2.88 (1H, dd, *J* = 13.6, 8.9 Hz, CCH^a**H^b**), 2.35 (3H, s, C**H$_3$**).

^{13}C NMR (126 MHz, $CDCl_3$) δ 159.0 (C), 147. (CH), 139.8 (C), 138.7 (C), 138.1 (C), 137.8 (CH), 137.8 (C), 131.4 (CH), 130.2 (CH), 129.1 (CH), 128.6 (CH), 128.4 (CH), 128.4 (CH), 126.6 (CH), 124.9 (CH), 124.8 (CH), 122.2 (CH), 75.1 (CH_2), 73.6 (CH_2), 71.9 (CH), 36.9 (CH_2), 21.6 (CH_3).

HRMS (ESI^+): Mass calculated for $C_{22}H_{23}NO_2$ [M+Na] = 356.1729. Mass found = 356.1622.

IR ν_{max} (neat/cm^{-1}): 3462 (O-H), 2989, 2186, 1767, 1561, 1426, 1308, 1190, 974, 766.

***1-((2-fluorobenzyl)oxy)-3-(2-(pyridin-2-yl)phenyl)propan-2-ol* (7c)**

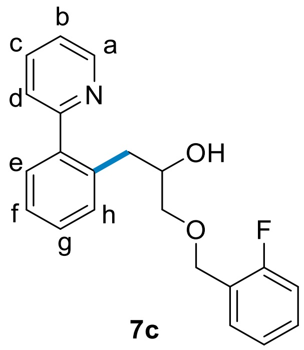

7c

The title compound was synthesised as outlined in general procedure **C** using 2-phenylpyridine (75 μL, 0.5 mmol) and 2-(((2-fluorobenzyl)oxy)methyl)oxirane (273 mg, 1.50 mmol). Purification by flash column chromatography (40% EtOAc/Hexane) afforded **7c** as a brown oil (101 mg, 0.3 mmol, 60 %).

R_f = 0.53 (2:3 EtOAc:Hexane)

^{1}H NMR (500 MHz, $CDCl_3$) δ 8.59–8.56 (1H, m, **H^a**), 7.84 (1H, td, *J* = 7.7, 1.8 Hz, **H^c**), 7.77–7.61 (1H, m, O**H**), 7.61–7.57 (1H, m, O**H**), 7.56–7.53 (1H, m, Ar**H**), 7.41–7.28 (7H, m, Ar**H**), 7.05–7.00 (2H, m, Ar**H**), 4.62–4.53 (2H, m, C**H**$_2$Ar), 4.14–4.08 (1H, m, C**H**OH), 3.62 (1H, dd, *J* = 9.4, 4.9 Hz, C**H^a**H^{b}O), 3.50 (1H, dd, *J* = 9.4, 6.5 Hz, CH^a**H^b**O), 2.96–2.84 (2H, m, CC**H**$_2$).

^{13}C NMR (101 MHz, $CDCl_3$) δ 162.3 (C, d, *J* = 244.6 Hz), 158.9 (C), 147.4 (CH), 139.8 (C), 137.8 (CH), 137.6 (C), 134.5 (C, d, *J* = 3.7 Hz), 131.3 (CH), 130.2 (CH), 129.5 (CH), 129.5 (CH), 129.1 (CH), 126.6 (CH), 124.8 (CH), 122.2 (CH),115.4 (CH), 115.1 (CH, d, *J* = 21.4 Hz), 75.1 (CH_2), 72.7 (CH_2), 71.9 (CH), 36.9 (CH_2).

^{19}F^2 NMR (376 MHz, $CDCl_3$) δ -115.1 (s).

HRMS (ESI$^+$): Mass calculated for $C_{21}H_{20}FNO_2$ [M+H] = 338.1478. Mass found = 338.1565.

IR ν_{max} (neat/cm^{-1}): 3164 (O-H), 3062, 1508, 1442, 1219, 1155, 1035, 823, 748, 630.

***1-phenyl-3-(2-(pyridin-2-yl)phenyl)propan-2-ol* (7d)**

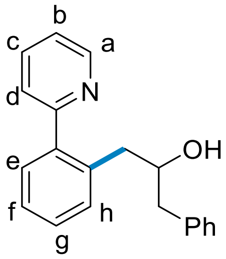

7d

The title compound was synthesised as outlined in general procedure **C** using 2-phenylpyridine (75 µL, 0.5 mmol) and 2-benzyloxirane (201 mg, 1.50 mmol). Purification by flash column chromatography (40% EtOAc/Hexane) afforded **7d** as a brown oil (145 mg, 0.38 mmol, 75 %).

R*f* = 0.65 (2:3 EtOAc:Hexane)

^{1}H NMR (500 MHz, $CDCl_3$) δ 8.50–8.47 (1H, m, **H^a**), 7.81 (1H, td, *J* = 7.7, 1.8 Hz, **H^c**), 7.52–7.48 (1H, m, **H^d**), 7.43–7.34 (3H, m, Ar**H**), 7.32–7.27 (6H, m, Ar**H**), 7.23–7.17 (1H, m, Ar**H**), 4.22–4.15 (1H, m, C**H**OH), 3.05 (1H, dd, *J* = 13.3, 5.4 Hz, C**H^a**H^{b}Ph), 2.85 (1H, dd, *J* = 13.3, 7.0 Hz, CH^a**H^b**Ph), 2.77–2.70 (2H, m, CC**H**$_2$).

^{13}C NMR (126 MHz, $CDCl_3$) δ 158.9 (C), 147.4 (CH), 139.7 (C), 139.3 (C), 138.4 (C), 137.7 (CH), 131.1 (CH), 130.1 (CH), 129.8 (2 x CH), 129.1 (CH), 128.4 (2 x CH), 126.5 (CH), 126.1 (CH), 124.7 (CH), 122.1 (CH), 74.3 (CH), 45.6 (CH_2), 38.9 (CH_2).

HRMS (ESI$^+$): Mass calculated for $C_{20}H_{19}NO$ [M+Na] = 312.1467. Mass found = 312.1351.

IR ν_{max} (neat/cm^{-1}): 3188 (O-H), 3060, 2917, 1592, 1561, 1427, 1044, 911, 797, 686.

2-(2-hydroxy-3-(2-(pyridin-2-yl)phenyl)propyl)isoindoline-1,3-dione **(7e)**

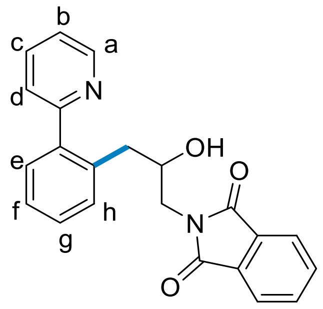

7e

The title compound was synthesised as outlined in general procedure **C** using 2-phenylpyridine (75 µL, 0.5 mmol) and 2-(oxiran-2-ylmethyl)isoindoline-1,3-dione (305 mg, 1.50 mmol). Purification by flash column chromatography (40% EtOAc/Hexane) afforded **7e** as a grey amorphous solid (100 mg, 0.28 mmol, 56 %).

R*f* = 0.5 (2:3 EtOAc:Hexane)

m.p. 139 °C

^{1}H NMR (500 MHz, $CDCl_3$) δ 8.66–8.62 (1H, m, **H^a**), 7.89–7.80 (3H, m, Ar**H**), 7.74–7.65 (2H, m, Ar**H**), 7.54 (1H, d, *J* = 7.8 Hz, **H^d**), 7.42–7.36 (3H, m, Ar**H**), 7.35–7.28 (2H, m, Ar**H**), 4.33 (1H, m, C**H**OH), 3.91 (1H, dd, *J* = 13.5, 7.5 Hz, C**H^a**H^{b}N), 3.80 (1H, dd, *J* = 13.5, 5.5 Hz, CH^a**H^b**N), 2.90–2.80 (2H, m, C**CH$_2$**).

^{13}C NMR (101 MHz, $CDCl_3$) δ 168.8 (2 x C), 158.8 (C), 147.5 (CH), 139.8 (C), 137.9 (CH), 137.2 (C), 134.0 (2 x CH), 132.4 (2 x C), 131.2 (CH), 130.2 (CH), 129.3 (CH), 126.7 (CH), 124.8 (CH), 123.4 (2 x CH), 122.3 (CH), 70.5 (CH), 45.4 (CH_2), 37.9 (CH_2).

HRMS (ESI$^+$): Mass calculated for $C_{22}H_{18}N_2O_3$ [M+Na] = 381.1317. Mass found = 381.1210.

IR ν_{max} (neat/cm^{-1}): 3190 (O-H), 2971, 1719, 1562, 1473, 1362, 1253, 1154, 1000, 849.

***1-(2-(pyridin-2-yl)phenyl)propan-2-ol* (7f)**

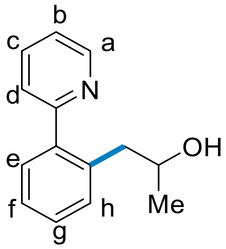

7f

The title compound was synthesised as outlined in general procedure **C** using 2-phenylpyridine (75 µL, 0.5 mmol) and 2-methyloxirane (87 mg, 1.5 mmol). Purification by flash column chromatography (40% EtOAc/Hexane) afforded **7f** as a yellow oil (60.0 mg, 0.28 mmol, 56 %).

R*f* = 0.46 (2:3 EtOAc:Hexane)

^{1}H NMR (500 MHz, $CDCl_3$) δ 8.63 (1H, m, **H^{a}**), 7.84 (1H, td, *J* = 7.8, 1.8 Hz, **H^{c}**), 7.54 (1H, d, *J* = 7.8 Hz, **H^{d}**), 7.43–7.37 (3H, m, Ar**H**), 7.34–7.29 (2H, m, Ar**H**), 7.22 (1H, m, O**H**), 4.16–4.06 (1H, m, C**H**OH), 2.82 (1H, dd, *J* = 13.6, 3.8 Hz, CC**H^{a}**H^{b}), 2.75 (1H, dd, *J* = 13.6, 9.0 Hz, CCH^{a}**H^{b}**), 1.33 (3H, d, *J* = 6.2 Hz, C**H**$_3$).

^{13}C NMR (126 MHz, $CDCl_3$) δ 159.1 (C), 147.5 (CH), 139.8 (C), 138.3 (C), 137.8 (CH), 131.4 (CH), 130.2 (CH), 129.1 (CH), 126.5 (CH), 124.8 (CH), 122.2 (CH), 69.3 (CH_2), 41.9 (CH), 25.2 (CH_3).

HRMS (ESI^+): Mass calculated for $C_{14}H_{15}NO$ [M+Na] = 236.1154. Mass found = 236.1045.

IR ν_{max} (neat/cm^{-1}): 3434 (O-H), 2975, 2870, 1709, 1602, 1458, 1325, 1187, 1111, 646.

***1-(2-(pyridin-2-yl)phenyl)octan-2-ol* (7g)**

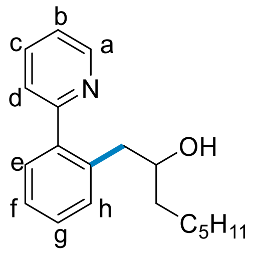

7g

The title compound was synthesised as outlined in general procedure **C** using 2-phenylpyridine (75 μL, 0.5 mmol) and 1,2-epoxyoctane (0.23 ml, 1.5 mmol). Purification by flash column chromatography (40% EtOAc/Hexane) afforded **7g** as a light brown oil (78 mg, 0.28 mmol, 55%).

$\mathbf{R_f}$ = 0.63 (2:3 EtOAc:Hexane)

^{1}H NMR (400 MHz, $CDCl_3$) δ 8.58 (1H, d, *J* = 4.9 Hz, $\mathbf{H^a}$), 7.78 (1H, td, *J* = 7.9, 1.8 Hz $\mathbf{H^c}$), 7.49 (1H, d, *J* = 7.9 Hz, $\mathbf{H^d}$), 7.40-7.32 (3H, m, Ar**H**), 7.30–7.23 (2H, m, Ar**H**), 7.18–7.13 (1H, m, O**H**) 3.92–3.83 (1H, m, C**H**OH), 2.79 (1H, dd, *J* = 13.6, 3.9 Hz, $CC\mathbf{H_a}H_b$), 2.72 (1H, dd, *J* = 13.6, 9.0 Hz, $CCH_a\mathbf{H_b}$),1.65–1.22 (10H, m, $(C\mathbf{H_2})_5$), 0.91-0.82 (3H, m, $-C\mathbf{H_3}$).

^{13}C NMR (101 MHz, $CDCl_3$) δ 159.0 (C), 147.3 (CH), 139.6 (C), 138.4 (CH), 137.6 (CH), 131.2 (CH), 130.0 (CH), 128.9 (CH), 126.3 (CH), 124.7 (CH), 122.0 (CH), 73.1 (C), 39.8 (CH_2), 39.1 (CH_2), 32.0 (CH_2), 29.6 (CH_2), 25.8 (CH_2), 22.7 (CH_2), 14.2 (CH_3).

HRMS (ESI^+): Mass calculated for $C_{19}H_{25}NO$ [M+Na] = 306.1936. Mass found = 306.1817.

IR ν_{max} (neat/cm^{-1}): 3223 (O-H), 2924, 2853, 1591, 1441, 1297, 1059, 748.

***1-ethoxy-3-(2-(pyridin-2-yl)phenyl)propan-2-ol* (7h)**

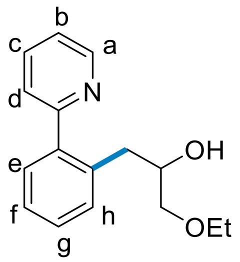

7h

The title compound was synthesised as outlined in general procedure **C** using 2-phenylpyridine (75 µL, 0.5 mmol) and 2-(ethoxymethyl)oxirane (153 mg, 1.50 mmol). Purification by flash column chromatography (40% EtOAc/Hexane) afforded **7h** as a brown oil (86 mg, 0.34 mmol, 67 %).

$\mathbf{R}_f$ = 0.76 (2:3 EtOAc:Hexane)

^{1}H NMR (400 MHz, $CDCl_3$) δ 8.61 (1H, m, $\mathbf{H^a}$), 7.83 (1H, td, *J* = 7.7, 1.8 Hz, $\mathbf{H^c}$), 7.54 (1H, d, *J* = 7.7 Hz, $\mathbf{H^d}$), 7.47–7.28 (5H, m, Ar**H**), 4.13–4.02 (1H, m, C**H**OH), 3.63–3.56 (2H, m, O$C\mathbf{H_2}CH_3$), 3.59–3.54 (1H, m, $C\mathbf{H^a}H^bO$), 3.43 (1H, dd, *J* = 9.5, 6.5 Hz, $CH^a\mathbf{H^b}O$), 2.92 (1H, dd, *J* = 13.6, 3.9 Hz, $CC\mathbf{H^a}H^b$), 2.84 (1H, dd, *J* = 13.6, 8.9 Hz, $CCH^a\mathbf{H^b}$), 1.23 (3H, t, *J* = 7.0 Hz, $C\mathbf{H_3}$).

^{13}C NMR (101 MHz, $CDCl_3$) δ 159.0 (C), 147.4 (CH), 139.9 (C), 137.8 (C), 137.8 (CH), 131.4 (CH), 130.1 (CH), 129.1 (CH), 126.5 (CH), 124.8 (CH), 122.2 (CH), 75.5 (CH_2), 71.8 (CH), 66.8 (CH_2), 36.9 (CH_2), 15.4 (CH_3).

HRMS (ESI^+): Mass calculated for $C_{16}H_{19}NO_2$ [M+Na] = 280.1416. Mass found = 280.1308.

IR ν_{max} (neat/cm^{-1}): 3354 (O-H), 2859, 1718, 1647, 1375, 1276, 1155, 1006, 764, 631.

***1-(tert-butoxy)-3-(2-(pyridin-2-yl)phenyl)propan-2-ol* (7i)**

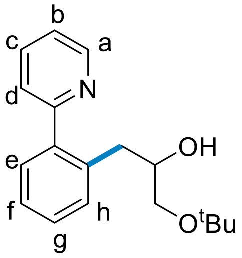

7i

The title compound was synthesised as outlined in general procedure **C** using 2-phenylpyridine (75 μL, 0.5 mmol) and 2-(tert-butoxymethyl)oxirane (195 mg,1.50 mmol). Purification by flash column chromatography (40% EtOAc/Hexane) afforded **7i** as a brown oil (97 mg, 0.34 mmol, 68 %).

R_f = 0.58 (2:3 EtOAc:Hexane)

^{1}H NMR (500 MHz, $CDCl_3$) δ 8.63–8.60 (1H, m, **H^a**), 7.83 (1H, td, *J* = 7.7, 1.8 Hz, **H^c**), 7.54 (1H, d, *J* = 7.7 Hz, **H^d**), 7.47–7.43 (1H, m, **H^h**), 7.42–7.28 (4H, m, Ar**H**), 4.00–3.92 (1H, m, C**H**OH), 3.52 (1H, dd, *J* = 8.8, 4.9 Hz, C**H^a**H^{b}O), 3.28 (1H, dd, *J* = 8.9, 7.2 Hz, CH^a**H^b**O), 2.97 (1H, dd, *J* = 13.6, 3.8 Hz, CC**H^a**H^b), 2.80 (1H, dd, *J* = 13.6, 9.1 Hz, CCH^a**H^b**), 1.21 (9H, s, C**H$_3$**).

^{13}C NMR (101 MHz, $CDCl_3$) δ 159.2 (C), 147.5 (CH), 139.9 (C), 138.1 (C), 137.7 (CH), 131.4 (CH), 130.1 (CH), 129.0 (CH), 126.4 (CH), 124.8 (CH), 122.1 (CH), 72.9 (C), 72.6 (CH), 66.5 (CH_2), 37.0 (CH_2), 27.8 (3 x CH_3).

HRMS (ESI$^+$): Mass calculated for $C_{18}H_{23}NO_2$ [M+Na] = 308.1621. Mass found = 308.1729.

IR ν_{max} (neat/cm^{-1}): 3245 (O-H), 1718, 1582, 1442, 1350, 1276, 1155, 1089, 906, 647.

1-(benzyloxy)-3-(6-(pyridin-2-yl)benzodioxol-5-yl)propan-2-ol (8a)

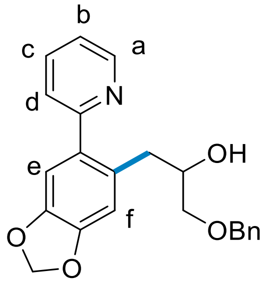

8a

The title compound was synthesised as outlined in general procedure **D** using 2-(benzodioxol-5-yl)pyridine (40 mg, 0.2 mmol) and **2c** (92 µL, 0.6 mmol). Purification by flash column chromatography (40% EtOAc/Hexane) afforded **8a** as a brown oil (40 mg, 0.11 mmol, 55 %).

R*f* = 0.5 (2:3 EtOAc:Hexane)

^{1}H NMR (400 MHz, $CDCl_3$) δ 8.54 (1H, d, *J* = 4.5 Hz, **H^{a}**), 7.81 (1H, td, *J* = 7.8, 1.8 Hz, **H^{c}**), 7.53–7.49 (1H, m, **H^{d}**), 7.38–7.24 (6H, m, Ar**H**), 6.93 (1H, d, *J* = 8.0 Hz, Ar**H**), 6.79 (1H, d *J* = 8.0 Hz, Ar**H**), 6.05–6.01 (2H, m, OC**H**), 4.67–4.59 (2H, m, C**H$_2$**Ph), 4.27–4.20 (1H, m, C**H**OH), 3.65 (1H, dd, *J* = 9.6, 4.8 Hz, OC**H^{a}**H^{b}), 3.55 (1H, dd, *J* = 9.6, 5.7 Hz, OCH^{a}**H^{b}**), 3.01 (1H, dd, *J* = 13.7, 3.5 Hz, CC**H^{a}**H^{b}), 2.80 (1H, dd, *J* = 13.7, 10.3 Hz, CCH^{a}**H^{b}**).

^{13}C NMR (126 MHz, $CDCl_3$) δ 157.9 (C), 147.9 (C), 147.2 (CH), 138.9 (CH), 138.2 (C), 128.5 (C), 128.4 (2 x CH), 127.8 (C), 127.8 (2 x CH), 127.5 (CH), 124.8 (CH), 124.1 (CH), 122.0 (CH), 120.1 (C), 106.8 (CH), 101.5 (CH_2), 75.7 (CH_2), 73.5 (CH_2), 69.6 (CH), 31.2 (CH_2).

HRMS (ESI^+): Mass calculated for [M+Na] =386.1471. Mass found = 386.1363.

IR ν_{max} (neat/cm^{-1}): 3178 (O-H), 2858, 1590, 1451, 1252, 1046, 783, 697.

1-(benzyloxy)-3-(2-(isoquinolin-1-yl)phenyl)propan-2-ol (8b)

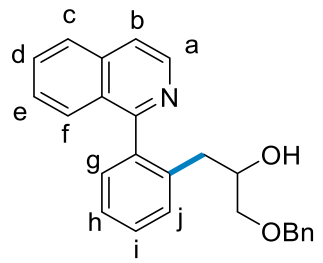

8b

The title compound was synthesised as outlined in general procedure **D** using 1-phenylisoquinoline (47 mg, 0.2 mmol) and **2c** (92 μL, 0.6 mmol). Purification by flash column chromatography (40% EtOAc/Hexane) afforded **8b** as a brown oil (36 mg, 0.1 mmol, 49 %).

R*f* = 0.48 (2:3 EtOAc:Hexane)

^{1}H NMR (400 MHz, $CDCl_3$) δ 8.49 (1H, d, *J* = 5.8 Hz, **H^a**), 7.91–7.85 (2H, m, Ar**H**), 7.75–7.63 2H, m, Ar**H**), 7.53–7.43 (2H, m, Ar**H**), 7.40–7.19 (8H, m, Ar**H**), 4.51 (2H, s, C**H$_2$**Ph), 4.06–3.98 (1H, m, C**H**OH), 3.55 (1H, dd, *J* = 9.5, 4.7 Hz, OC**H^a**H^b), 3.37 (1H, dd, *J* = 9.5, 6.1 Hz, OCH^a**H^b**), 2.89 (1H, dd, *J* = 13.7, 3.5 Hz, CC**H^a**H^b), 2.53 (1H, dd, *J* = 13.7, 10.6 Hz, CCH^a**H^b**).

^{13}C NMR (126 MHz, $CDCl_3$) δ 159.9 (C), 139.0 (CH), 138.7 (CH), 137.5 (CH), 131.9 (C), 131.1 (CH), 131.1 (C), 131.0 (CH), 128.4 (2 x CH), 128.3 (CH), 128.0 (CH), 127.9 (C), 127.8 (CH), 127.8 (C), 127.7 (2 x CH), 127.6 (CH), 127.2 (CH), 126.1 (C), 125.7 (CH), 75.5 (CH_2), 73.4 (CH), 72.4 (CH_2), 37.5 (CH_2).

HRMS (ESI$^+$): Mass calculated for [M+H] = 370.1728. Mass found = 370.1802.

IR ν_{max} (neat/cm^{-1}): 3451 (O-H), 2914, 2850, 1735, 1455, 1174, 1101, 733.

5-(2-(3-(benzyloxy)-2-hydroxypropyl)phenyl)-1,3-dihydro-2H-benzo[e][1,4]diazepin-2-one (8c)

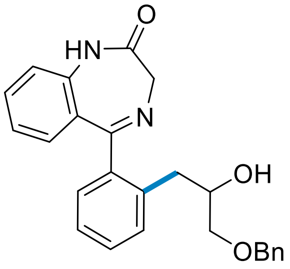

8c

The title compound was synthesised as outlined in general procedure **D** using 5-phenyl-1,3-dihydrobenzodiazepin-2-one (47 mg, 0.2 mmol) and **2c** (92 µL, 0.6 mmol). Purification by flash column chromatography (50% EtOAc/Hexane) afforded **8c** as a brown oil (37 mg, 0.1 mmol, 48 %).

R*f* = 0.11 (2:3 EtOAc:Hexane)

^{1}H NMR (400 MHz, $CDCl_3$) δ 9.35–9.19 (1H, br s, N**H**), 7.53–7.46 (1H, m, Ar**H**), 7.40–7.25 (7H, m, Ar**H**), 7.21–7.12 (2H, m, Ar**H**), 7.08 (2H, d, *J* = 4.3 Hz, Ar**H**), 6.95 (1H, d, *J* = 7.7 Hz, Ar**H**), 4.60 (2H, s, C**H**$_2$Ph), 4.51 (1H, d, *J* = 11.3 Hz, NC**H^a**H^b), 4.08–3.98 (2H, m, NCH^a**H^b** + C**H**OH), 3.58 (1H, dd, *J* = 9.5, 4.7 Hz, OC**H^a**H^b), 3.49 (1H, dd, *J* = 9.5, 6.0 Hz, OCH^a**H^b**), 3.04–2.90 (2H, m, CC**H$_2$**).

^{13}C NMR (101 MHz, $CDCl_3$) δ 172.4 (C), 170.9 (C), 138.9 (C), 138.7 (C), 138.6 (C), 138.5 (CH), 132.6 (C), 131.7 (CH), 131.4 (CH), 131.1 (CH), 130.1 (CH), 128.4 (2 x CH), 127.8 (2 x CH), 127.7 (CH), 126.0 (CH), 123.7 (CH), 121.2 (CH), 74.5 (CH_2), 73.5 (CH_2), 71.7 (CH), 55.2 (CH_2), 37.0 (CH_2).

HRMS (ESI$^+$): Mass calculated for [M+Na] = 423.1787. Mass found = 423.1679.

IR ν_{max} (neat/cm^{-1}): 3107 (O-H), 2981, 1638, 1425, 1351, 1178, 754, 624.

1-(benzyloxy)-3-(2-(4,5-dimethylpyridin-2-yl)-3,6-difluoro-4,5-dimethylphenyl)propan-2-ol (8d)

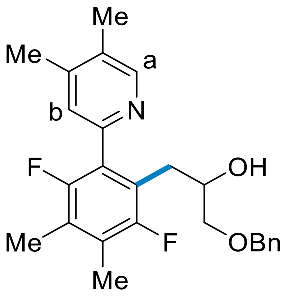

8d

The title compound was synthesised as outlined in general procedure **D** using 2-(2,5-difluoro-3,4-dimethylphenyl)-4,5-dimethylpyridine (49 mg, 0.2 mmol) and **2c** (92 µL, 0.6 mmol). Purification by flash column chromatography (40% EtOAc/Hexane) afforded **8d** as a brown oil (37 mg, 0.09 mmol, 45 %).

R_f = 0.46 (2:3 EtOAc:Hexane)

^{1}H NMR (500 MHz, $CDCl_3$) δ 8.32 (1H, s, NC**H**), 7.35–7.29 (5H, m, Ar**H**), 7.29–7.24 (2H, m, Ar**H**), 4.60 (2H, s, C**H**$_2$Ph), 4.18–4.09 (1H, m, C**H**OH), 3.61 (1H, dd, *J* = 9.7, 4.6 Hz, OC**H**^{a}H^b), 3.52 (1H, dd, *J* = 9.7, 5.4 Hz, OCH^a**H**^{b}), 3.00 (1H, d, *J* = 13.7 Hz, CC**H**^{a}H^b), 2.56–2.48 (1H, m, CCH^a**H**^{b}), 2.34 (3H, s, C**H**$_3$), 2.27 (3H, s, C**H**$_3$), 2.25–2.23 (3H, s, C**H**$_3$), 2.22–2.20 (3H, s, C**H**$_3$).

^{13}C NMR (101 MHz, $CDCl_3$) δ 156.1 (C, d, *J* = 240.6 Hz), 154.2 (C, d, *J* = 240.6 Hz), 149.6 (C), 148.0 (CH), 147.1 (C), 138.9 (CH), 131.8 (C), 128.4 (2 x CH), 127.9 (C, d, *J* = 5.4 Hz), 127.7 (2 x CH), 127.5 (CH), 125.6 (2 x C), 123.6 (C, dd, *J* = 20.0, 2.8 Hz), 123.4 (C, dd, *J* = 18.7, 4.9 Hz), 75.7 (CH_2), 73.4 (CH_2), 69.6 (CH), 30.7 (CH_2), 19.6 (CH_3), 16.4 (CH_3), 11.6 (CH_3, dd, *J* = 5.8, 2.2 Hz), 11.35 (CH_3, dd, *J* = 5.8, 2.2 Hz).

^{19}F NMR (376 MHz, $CDCl_3$) δ -123.6 (d, *J* = 18.7), -124.7 (d, *J* = 17.0 Hz).

HRMS (ESI^+): Mass calculated for [M+H] =412.2088. Mass found =412.2083.

IR v_{max} (neat/cm^{-1}): 3321 (O-H), 2908, 1743, 1604, 1452, 1087, 796, 699.

1-(benzyloxy)-3-(3,6-difluoro-2-(5-(trifluoromethyl)pyridin-2-yl)phenyl)propan-2-ol (8e)

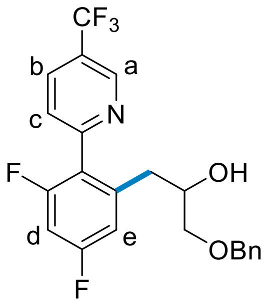

8e

The title compound was synthesised as outlined in general procedure **D** using 2-(2,4-difluorophenyl)-5-(trifluoromethyl)pyridine (52 mg, 0.2 mmol) and **2c** (92 µL, 0.6 mmol). Purification by flash column chromatography (40% EtOAc/Hexane) afforded **8e** as a brown oil (73 mg, 0.17 mmol, 86 %).

R_f = 0.45 (2:3 EtOAc:Hexane)

^{1}H NMR (400 MHz, $CDCl_3$) δ 8.93–8.90 (1H, m, **H^a**), 8.06 (1H, dd, *J* = 8.3, 2.3 Hz, **H^b**), 7.66 (1H, dd, *J* = 8.3, 3.4 Hz, **H^c**), 7.36–7.26 (5H, m, Ar**H**), 7.00–6.94 (1H, m, **H^e**), 6.84 (1H, ddd, *J* = 10.6, 8.4, 2.5 Hz, **H^e**), 4.57 (2H, s, C**H$_2$**Ph), 4.07–3.98 (1H, m, C**H**OH), 3.55 (1H, dd, *J* = 9.5, 4.9 Hz, OC**H^a**H^{b}C), 3.46 (1H, dd, *J* = 9.5, 5.9 Hz, OCH^a**H^b**C), 2.83 (1H, dd, *J* = 13.8, 4.0 Hz, CC**H^a**H^{b}C), 2.73 (1H, dd, *J* = 13.8, 9.2 Hz, CCH^a**H^b**C), 2.37 (1H, br s, O**H**).

^{13}C NMR (126 MHz, $CDCl_3$) δ 163.1 (C, dd, *J* = 252.0, 13.7 Hz), 160.8 (C, dd, *J* = 252.0, 12.8 Hz), 153.4 (C), 149.6 (CH), 142.5 (C, dd, *J* = 9.1, 3.7 Hz), 139.5 (C, dd, *J* = 34.8, 4.2 Hz), 138.3 (C), 128.5 (2 x CH), 127.9 (2 x CH), 127.9 (CH), 123.0 (C), 122.9 (CH), 122.6 (C, q, *J* = 270.0 Hz), 118.7 (CH), 113.7 (CH, dd, *J* = 21.3, 3.6 Hz), 102.6 (CH, t, *J* = 27.3 Hz), 74.3 (CH_2), 73.6 (CH_2), 71.3 (CH), 36.6 (CH_2).

^{19}F^2 NMR (471 MHz, $CDCl_3$) δ -62.4 (s), -107.7 (q, *J* = 9.0 Hz), -112.0 (t, *J* = 9.7 Hz).

HRMS (ESI$^+$): Mass calculated for [M+Na] = 446.1258. Mass found = 446.1273.

IR ν_{max} (neat/cm^{-1}): 3367 (O-H), 2934, 1623, 1401, 1120, 993, 845, 698.

1-(benzyloxy)-3-(3,6-difluoro-2-(4-(trifluoromethyl)pyridin-2-yl)phenyl)propan-2-ol (8f)

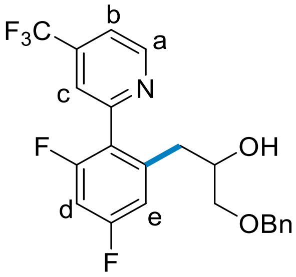

8f

The title compound was synthesised as outlined in general procedure **D** using 2-(2,4-difluorophenyl)-4-(trifluoromethyl)pyridine (52 mg, 0.2 mmol) and **2c** (92 µL, 0.6 mmol). Purification by flash column chromatography (40% EtOAc/Hexane) afforded **8f** as a brown oil (60 mg, 0.14 mmol, 71 %).

R_f = 0.46 (2:3 EtOAc:Hexane)

^{1}H NMR (400 MHz, $CDCl_3$) δ 8.77 (1H, d, *J* = 5.2 Hz, **H^a**), 7.76–7.72 (1H, m, **H^c**), 7.52 (1H, d, *J* = 4.7 Hz, **H^b**), 7.37–7.28 (5H, m, Ar**H**), 6.99–6.95 (1H, m, Ar**H**), 6.87–6.81 (1H, m, Ar**H**), 4.56 (2H, s, C**H**$_2$Ph), 4.07–3.99 (1H, m, C**H**OH), 3.55 (1H, dd, *J* = 9.4, 4.6 Hz, OC**H^a**H^{b}C), 3.47 (1H, dd, *J* = 9.4, 5.9 Hz, OCH^a**H^b**C), 2.82 (1H, dd, *J* = 13.8, 4.0 Hz, CC**H^a**H^{b}C), 2.72 (1H, dd, *J* = 13.8, 9.3 Hz, CCH^a**H^b**C).

^{13}C NMR (126 MHz, $CDCl_3$) δ 162.7 (C, dd, *J* = 334.0, 14.8 Hz), 160.9 (C, dd, *J* = 332.7, 12.5 Hz), 153.4 (C), 149.6 (CH), 142.5 (C, dd, *J* = 9.2, 2.9 Hz), 139.5 (C, dd, *J* = 34.8, 4.0 Hz), 138.3 (C), 128.5 (2 x CH), 127.9 (2 x CH), 127.9 (CH), 123.1 (C, dt, *J* = 16.3, 5.0 Hz), 123.0 (CH), 122.6 (C, q, *J* = 272.4 Hz), 118.7 (CH), 113.8 (CH, dd, *J* = 20.9, 3.4 Hz), 102.6 (CH, t, *J* = 27.0 Hz), 74.3 (CH_2), 73.6 (CH_2), 71.4 (CH), 36.7 (CH_2).

^{19}F^2 NMR (376 MHz, $CDCl_3$) δ -64.8 (s), -107.9 (q, *J* = 8.8 Hz), -112.2 (td, *J* = 9.8, 3.1 Hz).

HRMS (ESI$^+$): Mass calculated for [M+Na] = 446.1258. Mass found = 446.1273.

IR ν_{max} (neat/cm^{-1}): 3214 (O-H), 2981, 1623, 1334, 1121, 951, 895, 697.

1-(benzyloxy)-3-(3,6-difluoro-2-(5-methoxypyridin-2-yl)phenyl)propan-2-yl acetate (3pb)

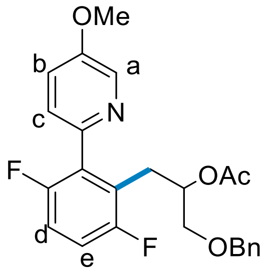

3pb

The title compound was synthesised by charging a 10 mL oven dried microwave vial equipped with a stirrer bar with **1p** (44 mg, 0.2 mmol), the protected alkyl iodide **2e** (67 mg, 0.2 mmol), [Ru(*p*-cymene)Cl_2]$_2$ (8.00 mg, 0.01 mmol), K_2CO_3 (83 mg, 0.6 mmol) and acetone (0.35 mL) and irradiated with 440 nm light for 24 h. The product was isolated by column chromatography (20% EtOAc/Hexane) to give a yellow oil (22 mg, 0.06 mmol, 32%).

R_f = 0.41 (1:4 EtOAc/Hexane)

^{1}H NMR (500 MHz, $CDCl_3$) δ 8.37 (1H, d, *J* = 2.9 Hz, H^a), 7.36–7.27 (5H, m, Ar**H**), 7.23–7.19 (2H, m, Ar**H**), 7.06–6.96 (2H, m, Ar**H**), 5.16–5.09 (1H, m, C**H**OAc), 4.43 (1H, d, *J* = 12.1 Hz, OC**H**^{a}H^{b}Ph), 4.37 (1H, d, *J* = 12.1 Hz, OCH^a**H**^{b}Ph), 3.87 (3H, s, C**H**$_3$), 3.37 (2H, d, *J* = 5.0 Hz, C**H**$_2$OBn), 3.04 (2H, dd, *J* = 6.8, 2.0 Hz, CC**H**$_2$), 1.92 (3H, s, C**H**$_3$).

^{13}C NMR (101 MHz, $CDCl_3$) δ 170.6 (C), 157.5 (C), 155.1 (2 x C), 144.8 (C, d, *J* = 2.1 Hz), 138.2 (C), 137.4 (CH), 128.5 (2 x CH), 127.7 (CH), 127.6 (2 x CH), 126.5 (CH, d, *J* = 2.5 Hz), 125.8 (C), 125.6 (C), 120.8 (CH), 115.8 (CH, dd, *J* = 24.9, 8.4 Hz), 115.0 (CH, dd, *J* = 26.0, 9.1 Hz), 73.0 (CH_2), 72.0 (CH_2), 71.0 (CH_2), 55.8 (CH), 27.5 (CH_3), 21.2 (CH_3).

^{19}F^2 NMR (471 MHz, $CDCl_3$) δ -120.2 (d, *J* = 17.4 Hz), -121.0 (d, *J* = 17.4 Hz).

HRMS (ESI^+): Mass calculated for [M+H] = 428.1595. Mass found = 428.1668.

IR ν_{max} (neat/cm^{-1}): 3014, 2894, 1876 (C=O), 1546, 1387, 1256, 1023, 746.

2-(2-(3-(benzyloxy)propyl)phenyl)pyridine (3aa)

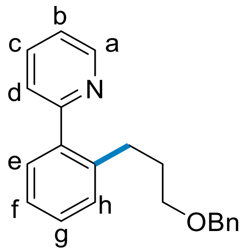

3aa

This product was formed following a procedure from the literature.[21] A microwave vial was charged with RuBnN (8.0 mg, 0.02 mmol, 10 mol%), K_3PO_4 (127 mg, 0.60 mmol, 3.00 equiv), potassium phenyl phosphonate (14 mg, 0.06 mmol, 0.30 equiv), **1a** (31 µl, 0.2 mmol, 1.0 equiv) and the alkyl iodide **2b** (55 mg, 0.2 mmol, 1.0 equiv) followed by NMP (1.0 mL). The vials were then capped and transferred outside the argon filled glovebox and stirred at 25 ^{o}C for 24 h. The product was purified by column chromatography (20% EtOAc/Hexane) to give **3aa** as a colourless oil (24 mg, 0.08 mmol, 40%).

R*f* = 0.46 (1:4 EtOAc/Hexane)

^{1}H NMR (400 MHz, $CDCl_3$) δ 8.69–8.65 (1H, m, **H^{a}**), 7.71 (1H, td, *J* = 7.8, 1.8 Hz, **H^{c}**), 7.38 (1H, dt, *J* = 7.8, 1.1 Hz, **H^{d}**), 7.36–7.25 (10H, m, Ar**H**), 7.23 (1H, ddd, *J* = 7.6, 4.8, 1.2 Hz, **H^{b}**), 4.40 (2H, s, PhC**H**$_2$), 3.37 (2H, t, *J* = 6.2 Hz, OC**H**$_2$), 2.8 (2H, t, *J* = 8.1 Hz, CC**H**$_2$), 1.83–1.76 (2H, m, CH_2C**H**$_2$).

^{13}C NMR (101 MHz, $CDCl_3$) δ 160.3 (C), 149.3 (CH), 140.6 (C), 140.0 (C), 138.8 (C), 136.3 (CH), 130.0 (2 x CH), 128.5 (CH), 128.4 (2 x CH), 127.7 (2 x CH), 127.6 (CH), 126.1 (CH), 124.2 (CH), 121.8 (CH), 73.0 (CH_2), 69.8 (CH_2), 31.2 (CH_2), 29.7 (CH_2).

HRMS (ESI^{+}): Mass calculated for [M+H] = 304.1623. Mass found = 304.1722.

IR ν_{max} (neat/cm^{-1}): 2985, 1737, 1372, 1233 (C-O), 1043, 918, 786, 634.

1-(benzyloxy)-3-iodopropan-2-ol (2d)

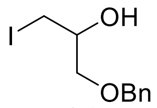

2d

The title compound was synthesised as described within the literature.[22] A round bottomed flask was charged with NaI (300 mg, 2.00 mmol, 1.00 equiv) and NaOAc (164 mg, 2.00 mmol, 1.00 equiv). To this was added AcOH (10 mL) and then **2c** (0.3 mL, 2.0 mmol, 1.0 equiv). Purification by column chromatography (10% EtOAc/Hexane) afforded **2d** as a colourless oil. (256 mg, 79%).

R_f =0.32 (1:4 EtOAc/Hexane)

^{1}H NMR (400 MHz, $CDCl_3$) δ 7.32–7.19 (5H, m, Ar**H**), 4.47 (2H, s, PhC**H**$_2$), 3.73–3.65 (1H, m, C**H**OH), 3.48 (2H, appt d, J = 5.1 Hz, C**H**$_2$I), 3.25 (1H, dd, J = 10.2, 5.5 Hz, C**H**$_a$H$_b$OBn), 3.17 (1H, dd, J = 10.2, 5.9 Hz, CH$_a$**H**$_b$OBn), 2.78 (1H, d, J = 5.0 Hz, O**H**).

^{13}C NMR (101 MHz, $CDCl_3$) δ 137.6 (C), 128.5 (2 x CH), 128.0 (CH), 127.9 (2 x CH), 73.5 (CH_2), 72.7 (CH_2), 69.9 (CH), 9.5 (CH_2).

2-(3-(pyridin-2-yl)phenyl)cyclohexan-1-ol (7l)

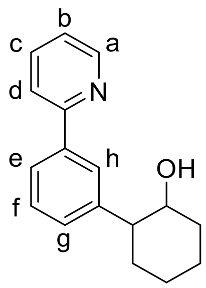

7l

The title compound was synthesised as outlined in general procedure **C** using cyclohexene oxide (59 mg, 0.60 mmol). Purification by flash column chromatography (40% EtOAc/Toluene) afforded **7l** as a brown oil (7 mg, 5 %). A mixture of ortho products were also produced from this reaction (~20%) but we were unable to separate these.

R_f = 0.12 (2:8 EtOAc/Hexane)

^{1}H NMR (400 MHz, $CDCl_3$) δ 8.69 (1H, d, *J* = 4.9 Hz, **H^a**), 7.91 (1H, t, *J* = 1.7 Hz, **H^h**), 7.83 (1H, dt, *J* = 7.9, 1.2 Hz, **H^d**), 7.78–7.71 (2H, m, Ar**H**), 7.45 (1H, t, *J* = 7.4 Hz, **H^f**), 7.32 (1H, dt, *J* = 7.5, 1.4 Hz, **H^g**), 7.25–7.21 (1H, m, **H^c**), 3.82–3.73 (1H, m, CC**H**), 2.60–2.50 (1H, m, C**H**OH), 2.18–2.10 (1H, m, C**H^a**H^b), 1.97–1.84 (2H, m, C**H**$_2$), 1.82–1.74 (1H, m, CH^a**H^b**), 1.50 –1.31 (4H, m, C**H**$_2$).

^{13}C NMR (126 MHz, $CDCl_3$) δ 157.6 (C), 149.8 (CH), 144.1 (C), 140.0 (C), 136.9 (CH), 129.3 (CH), 128.7 (CH), 126.7 (CH), 125.6 (CH), 122.3 (CH), 120.9 (CH), 74.5 (CH), 53.6 (CH), 34.7 (CH_2), 33.6 (CH_2), 26.2 (CH_2), 25.2 (CH_2).

HRMS (ESI^+): Mass calculated for [M+H] = 254.1467. Mass found = 254.1552.

IR ν_{max} (neat/cm^{-1}): 3302 (O-H), 2981, 1452, 1123, 987, 869, 756, 658.

1-(benzyloxy)-2-methyl-3-(2-(pyridin-2-yl)phenyl)propan-2-ol (7m)

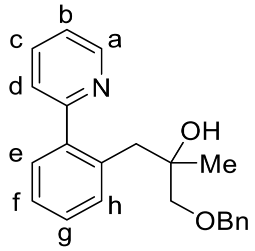

7m

The title compound was synthesised as outlined in general procedure **C** using 2-((benzyloxy)methyl)-2-methyloxirane (106 mg, 0.60 mmol). Purification by flash column chromatography (20% EtOAc/Hexane) afforded **7m** as a brown oil (45 mg, 67 %).

R*f* = 0.19 (2:8 EtOAc/Hexane)

^{1}H NMR (400 MHz, $CDCl_3$) δ 8.54 (1H, d, *J* = 5.1 Hz, **H^{a}**), 8.34 (1H, br s, O**H**), 7.82 (1H, td, *J* = 7.7, 1.9 Hz, **H^{b}**), 7.50 (1H, d, *J* = 8.2 Hz, **H^{d}**), 7.42–7.24 (10H, m, Ar**H**), 4.62 (2H, s, C**H$_2$**Ph), 3.50 (1H, d, *J* = 8.6 Hz, CC**H^{a}**H^{b}), 3.36 (1H, d, *J* = 8.3 Hz, CCH^{a}**H^{b}**), 3.12 (1H, d, *J* = 13.8 Hz, C**H^{a}**H^{b}OBn), 2.73 (1H, d, *J* = 13.8 Hz, CH^{a}**H^{b}**OBn), 1.36 (3H, s, C**H$_3$**).

^{13}C NMR (126 MHz, $CDCl_3$) δ 158.98 (C), 147.1 (CH), 140.2 (C), 138.9 (C), 137.8 (CH), 136.6 (C), 132.8 (CH), 130.3 (CH), 128.5 (CH), 128.4 (2 x CH), 127.8 (2 x CH), 127.6 (CH), 126.6 (CH), 125.2 (CH), 122.2 (CH), 78.0 (CH_2), 73.5 (CH_2), 40.8 (CH_2), 26.4 (CH_3).

HRMS (ESI^+): Mass calculated for [M+H] = 334.1729. Mass found = 334.1818.

IR ν_{max} (neat/cm^{-1}): 3289 (O-H), 2984, 1635, 1230, 1158, 952, 765, 685.

1-(benzyloxy)-3-iodopropan-2-yl acetate (2e)

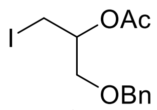

2e

An oven dried round bottomed flask was charged with alkyl iodide **2d** (101 mg, 0.35 mmol, 1.00 equiv), AcCl (39 mg, 0.51 mmol, 1.4 equiv), Et_3N (51 mg, 0.51 mmol, 1.4 equiv) and DMAP (10 mg, 0.17 mmol, 0.5 equiv). DCM (1 mL) was added and the reaction stirred at room temperature for 48 h. Purification by column chromatography (10% EtOAc/Hexane) gave the title compound as a colourless oil (80 mg, 0.24 mmol, 69%)

R_f = 0.35 (1:9 EtOAc/Hexane)

^{1}H NMR (400 MHz, $CDCl_3$) δ 7.34–7.24 (5H, m, Ar**H**), 4.88 (1H, *J* = 5.3 Hz, C**H**OAc), 4.56–4.74 (2H, m, C**H$_2$**Ph), 3.65 (1H, dd, *J* = 10.4, 5.0 Hz, OC**H$_a$**H$_b$C), 3.55 (1H, dd, *J* = 10.4, 5.0 Hz, OCH$_a$**H$_b$**C), 3.40 (1H, dd, *J* = 10.4, 5.6 Hz, CC**H$_a$**H$_b$C), 3.31 (1H, dd, *J* = 10.4, 5.6 Hz), 2.06 (3H, s, C**H$_3$**).

^{13}C NMR (101 MHz, $CDCl_3$) δ 170.1 (C), 137.7 (C), 128.5 (2 x CH), 127.9 (CH), 127.8 (2 x CH), 73.5 (CH_2), 71.4 (CH_2), 70.2 (CH), 21.1 (CH_2), 4.1 (CH_3).

4,4-dimethyl-2-phenyl-1,3-dioxolane (2h)

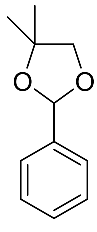

2h

In an argon filled glove box, an oven dried microwave vial was charged with BCRC **6a** (56.6 mg, 0.08 mmol, 1.00 equiv), alkyl iodide **2g** (49.0 mg, 0.16 mmol, 2.00 equiv) and acetone (2 mL, 0.04M). The vial was capped, transferred out of the glove box and irradiated at 440 nm for 16 h. The solvent was removed under vacuum and the residue purified by column chromatography (10% Et_2O in pentane) to give acetal **2h** (13 mg, 0.08 mmol, 97%) as a colourless oil.

Data in accordance with those previously reported.[23]

^{1}H NMR (700 MHz, $CDCl_3$) δ 7.51 – 7.47 (2H, m, Ar**H**), 7.40 – 7.34 (3H, m, Ar**H**), 5.91 (1H, s, **H**^{a}), 3.87 (1H, d, *J* = 7.8 Hz, OC**H**^{a}H^b), 3.78 (1H, d, *J* = 7.8 Hz, OCH^a**H**^{b}), 1.43 (3H, s, C**H**$_3$), 1.42 (3H, s, C**H**$_3$).

^{13}C NMR (176 MHz, $CDCl_3$) δ 138.4 (C), 129.3 (C), 128.5 (CH), 126.7 (CH), 103.45 (C), 79.4 (CH), 76.8 (CH), 27.0 (CH_3), 25.3 (CH_3).

HRMS (APCI+): Mass calculated for C11H15O2 [M+H] = 179.1067. Mass found = 179.1068

IR Vmax (Neat/cm-1): 2926 (C-H), 1720, 1460, 1090, 757, 641

1-(benzyloxy)propan-2-one (2k)

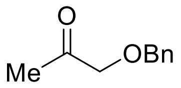

2k

The title compound was synthesised as a side product of the reaction outlined in general procedure **B** using 2-phenylpyridine (71 µl, 0.5 mmol) and **2c** (0.23 mL, 1.5 mmol). Purification by flash column chromatography (10% EtOAc/Hexane) afforded **2k** as a colourless oil (8 mg, 0.045 mmol , 9%).

Data is in accordance with those previously reported.[24]

R_f =0.39 (1:4 EtOAc/Hexane)

^{1}H NMR (400 MHz, $CDCl_3$) δ 7.29–7.17 (5H, m Ar**H**), 4.49 (2H, s, PhC**H$_2$**), 3.95 (2H, s, C(O)C**H$_2$**), 2.04 (3H, s, C**H$_3$**).

^{13}C NMR (126 MHz, $CDCl_3$) δ 206.4 (C), 137.1 (C), 128.4 (2 x CH), 127.8 (CH), 127.7 (2 x CH), 75.1 (CH_2), 73.1 (CH_2), 26.2 (CH_3).

Synthesis of (((2-Methylallyl)oxy)methyl)benzene

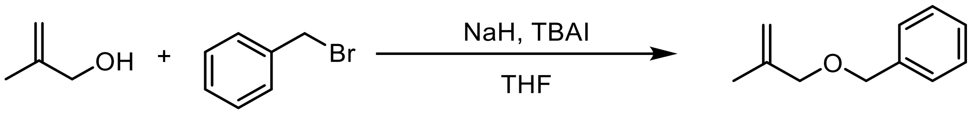

Neat 2-methylprop-2-en-1-ol (5.83 mL, 69.3 mmol, 1.00 equiv) was added dropwise to a suspension of sodium hydride (2.03 g, 76.2 mmol, 1.10 equiv, 90 % in mineral oil) in THF (40 mL) at 0 ºC. After 1 h, benzyl bromide (8.24 mL, 69.3 mmol, 1.00 equiv) was added dropwise, followed by tetrabutylammonium iodide (691 mg, 1.87 mmol, 0.027 equiv). The reaction was stirred at 0 ºC for 30 min, after which stirring was continued at room temperature for 4 h. The reaction was quenched with NH_4Cl (20 mL, sat. aq.) and extracted with Et_2O (3 x 50 mL). The combined organics were washed with water (30 mL), brine (30 mL) and dried ($MgSO_4$). The solvent was removed under vacuum to give (((2-Methylallyl)oxy)methyl)benzene (9.67 g, 56.0 mmol, 86%) as a colourless liquid which was used without further purification.

^{1}H NMR (400 MHz, $CDCl_3$) δ 7.41 – 7.27 (5H, m, ArH), 5.03 (1H, m, CC**H**^{a}H^b), 4.98 – 4.92 (1H, m, CCH^a**H**^{b}), 4.52 (2H, s, OC**H**$_2$C), 3.96 (2H, s, CH_3CC**H$_2$**), 1.79 (3H, m, C**H**$_3$).

^{13}C NMR (101 MHz, $CDCl_3$) δ 142.3 (C), 138.6 (C), 128.5 (CH), 127.8 (CH), 127.6 (C), 112.4 (C), 74.2 (CH_2), 71.9 (CH_2), 19.7 (CH_3).

IR V_{max} (neat/cm^{-1}): 2853 (C-H), 1656 (C=C), 1452, 1094, 898, 734, 695

GCMS (EI): Mass calculated for $C_{11}H_{14}O$ [M^+] = 162.1. Mass found = 162.2.

Synthesis of 2-((Benzyloxy)methyl)-2-methyloxirane (2f)

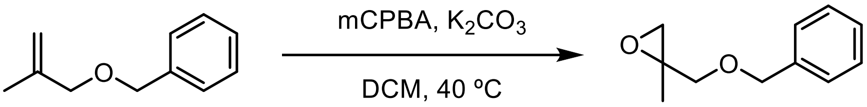

2f

To a solution of (((2-Methylallyl)oxy)methyl)benzene (3.00 g, 18.5 mmol, 1.00 equiv) in DCM (150 mL) was added K_2CO_3 (6.64 g, 48.1 mmol, 2.60 equiv) followed by mCPBA (6.64 g, 27.7 mmol, 1.50 equiv, 72% in H_2O). The mixture was heated at 40 ºC for 6 h with vigorous stirring. After cooling to room temperature sodium sulphite (100 mL, 10% aq. sln) was added and the mixture was stirred for 30 min. The phases were separated and the organics washed with sodium sulphite (50 mL, 10% aq. sln.), water (50 mL), brine (50 mL) and dried ($MgSO_4$). The solvent was removed under vacuum and the residue purified by column chromatography (10 % EtOAc in hexane) to give 2-((benzyloxy)methyl)-2-methyloxirane (2.34 g, 13.0 mmol, 71%) as a colourless liquid.

^{1}H NMR (400 MHz, $CO(CD_3)_2$) δ 7.43 – 7.21 (m, 5H, Ar**H**), 4.60 – 4.49 (m, 2H, OC**H₂**Ph), 3.58 (d, *J* = 10.9 Hz, 1H, OCC**H**^{a}H^b), 3.39 (d, *J* = 10.9 Hz, 1H, OCCH^a**H**^{b}), 2.65 (d, *J* = 5.0 Hz, 1H, OC**H**^{a}H^b), 2.56 (d, *J* = 5.0 Hz, 1H, OCH^a**H**^{b}), 1.33 (s, 3H, C**H**$_3$).

^{13}C NMR (101 MHz, $CO(CD_3)_2$) δ 139.6 (C), 129.1 (CH), 128.3 (CH), 128.2 (C), 74.6 (CH_2), 73.5 (CH_2), 56.2 (C), 51.4 (CH_2), 18.7 (CH_3).

GCMS (EI): Mass calculated for $C_{11}H_{14}O_2$ [M^+] = 178.1. Mass found = 178.1.

IR V_{max} (neat/cm^{-1}): 2860 (C-H), 1496, 1088, 736, 697

Synthesis of 1-(Benzyloxy)-3-iodo-2-methylpropan-2-ol (2g)

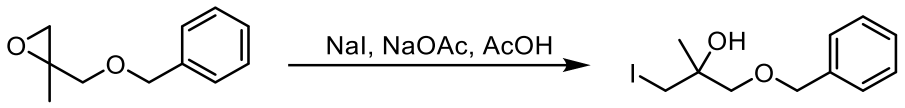

2f **2g**

To a solution of 2-((Benzyloxy)methyl)-2-methyloxirane (340 mg, 1.91 mmol, 1.00 equiv) in acetic acid (15 mL) was added sodium acetate (313 mg, 3.82 mmol, 2.00 equiv) followed by sodium iodide (573 mg, 3.82 mmol, 2.00 equiv). The mixture was stirred at RT for 6 h, after which the volatiles were removed under vacuum. The residue was redissolved in Et_2O (100 mL) and water (100 mL). The phases were separated and the organics washed with $NaHCO_3$ (3 x 100 mL, sat. aq.), sodium thiosulfate (50 mL, 10% aq. sln.), brine (50 mL) and dried ($MgSO_4$). The solvent was removed under vacuum and the residue purified by column chromatography (20% EtOAc in hexane) to give 1-(Benzyloxy)-3-iodo-2-methylpropan-2-ol (433 mg, 1.41 mmol, 74%) as a yellow oil.

^{1}H NMR (400 MHz, $CDCl_3$) δ 7.32 – 7.14 (m, 5H, Ar**H**), 4.49 (s, 2H, C**H_2**Ph), 3.50 (d, *J* = 9.1 Hz, 1H, OCC**H^a**H^bO), 3.35 (d, *J* = 9.1 Hz, 1H, OCCH^a**H^b**O), 3.28 (m, 2H, OCCH_2I), 2.46 (s, 1H, **OH**), 1.26 (s, 3H, C**H_3**).

^{13}C NMR (101 MHz, $CDCl_3$) δ 137.8 (C), 128.6 (CH), 128.0 (C), 127.8 (CH), 75.1 (CH_2), 73.6 (C), 71.0 (CH_2), 23.9 (CH_3), 16.6 (CH_2).

GCMS (EI): Mass calculated for $C_{11}H_{15}O_2I$ [M^+] = 306.1. Mass found = 306.1.

IR *V_{max}* (neat/cm^{-1}): 3432 (OH), 2858 (C-H), 1453, 1093, 735, 696

9.0. Spectra

9.1. Mono-Cyclometallated Ruthenium Species

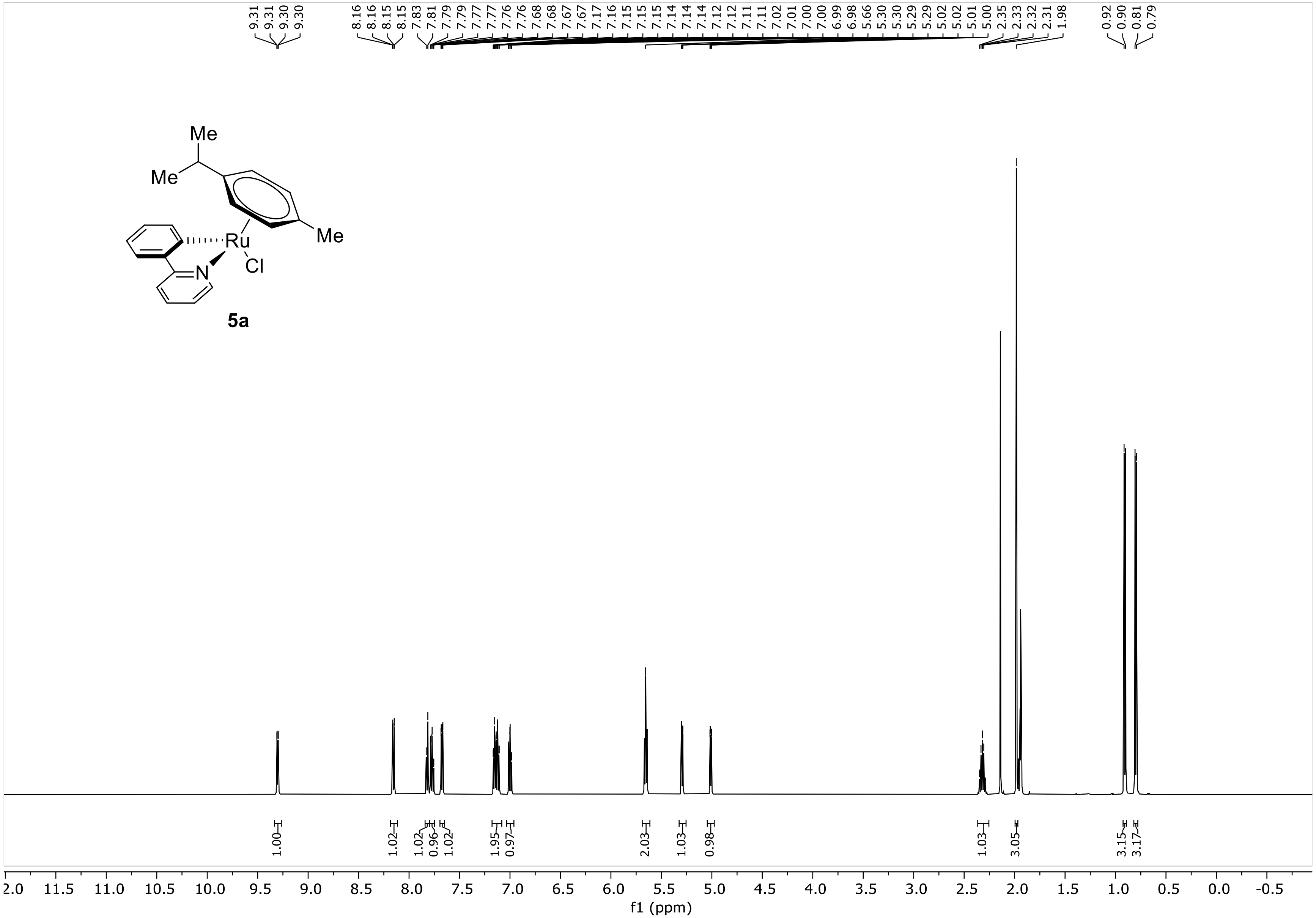

^{1}H NMR spectra (500 MHz, CD_3CN) of *p*-cymene bound mono-cyclometallated ruthenium species **5a**

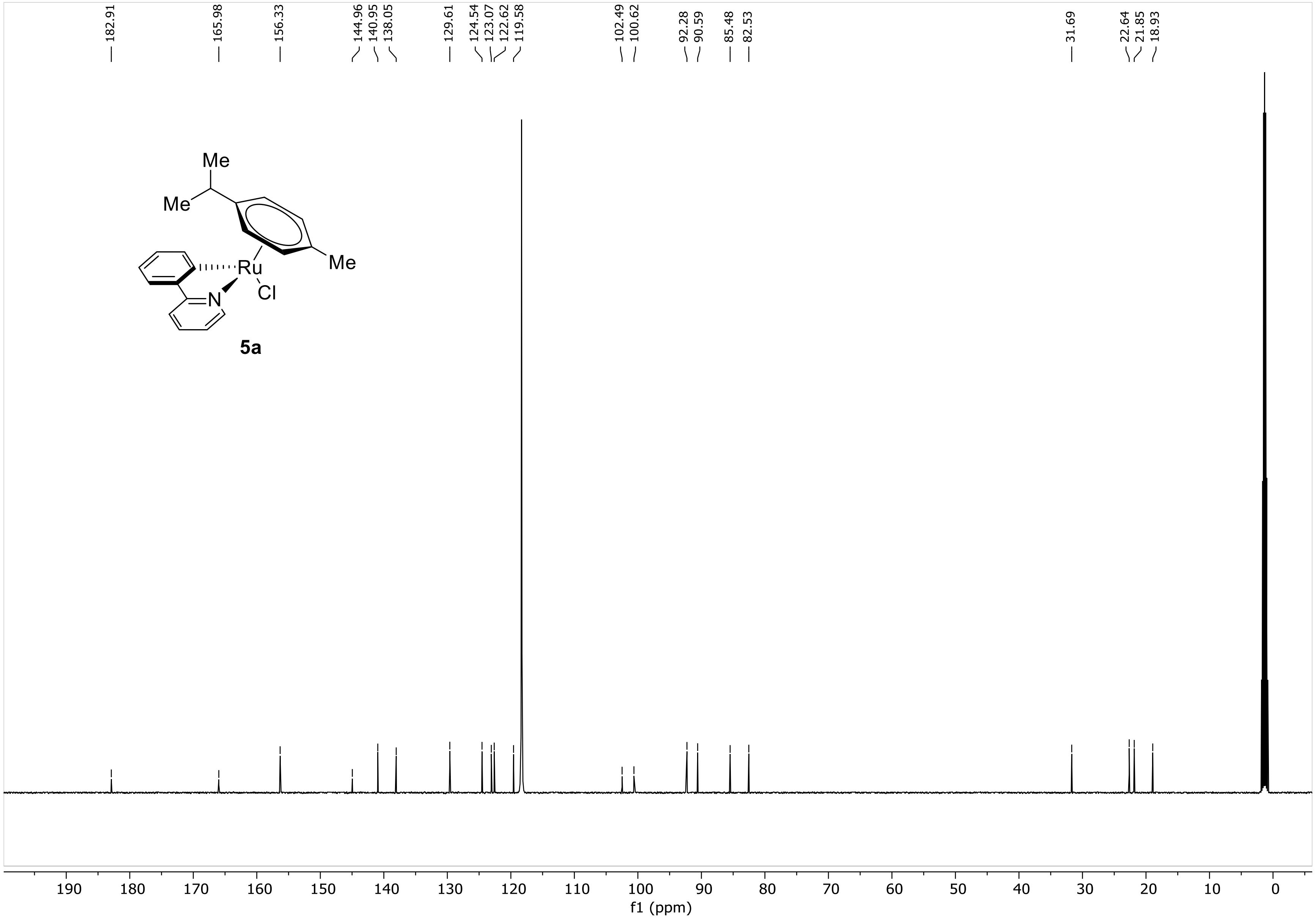

^{13}C NMR spectra (126 MHz, CD_3CN) of *p*-cymene bound mono-cyclometallated ruthenium species **5a**

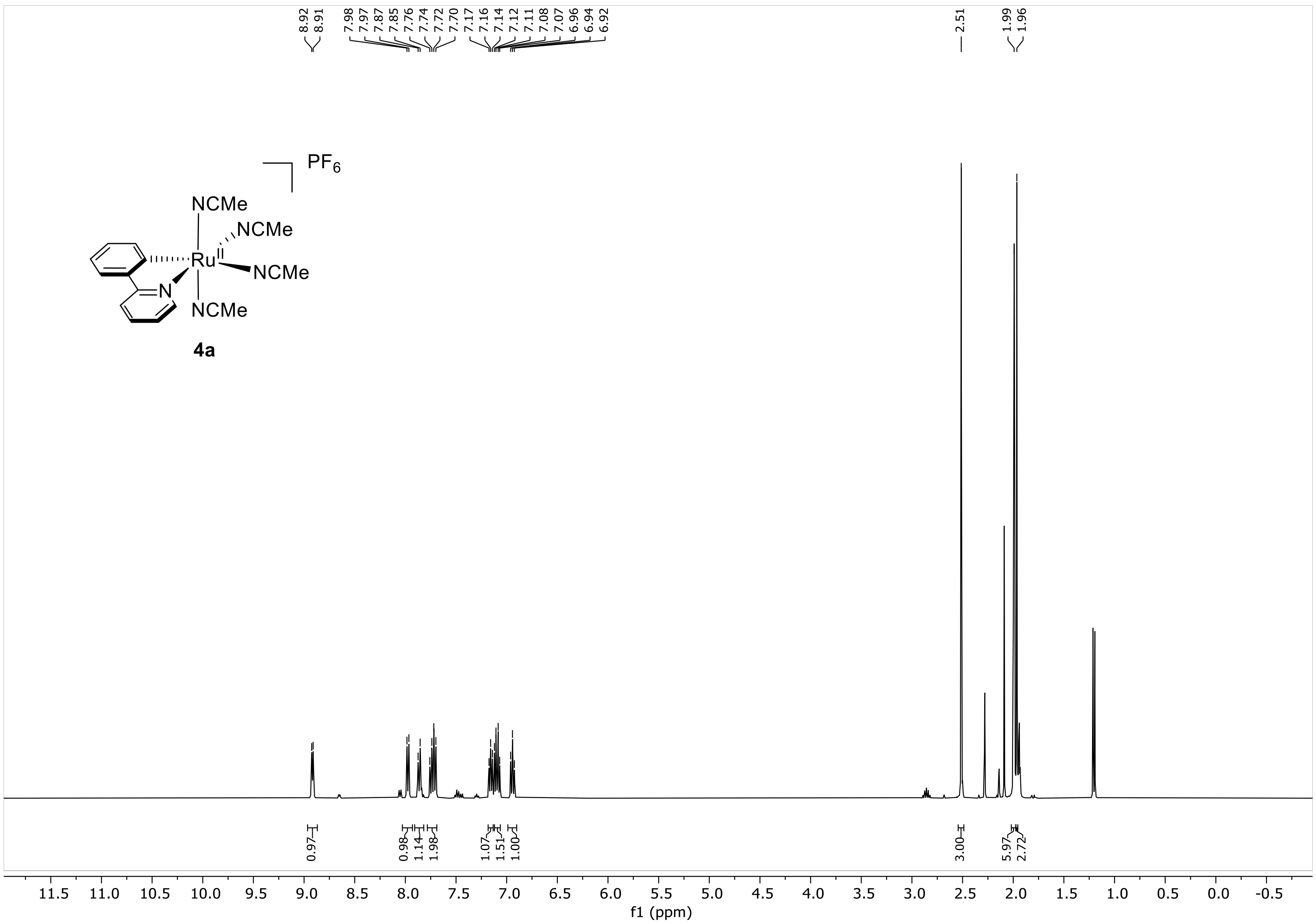

^{1}H NMR spectra (400 MHz, CD_3CN) of cationic mono-cyclometallated ruthenium species **4a**

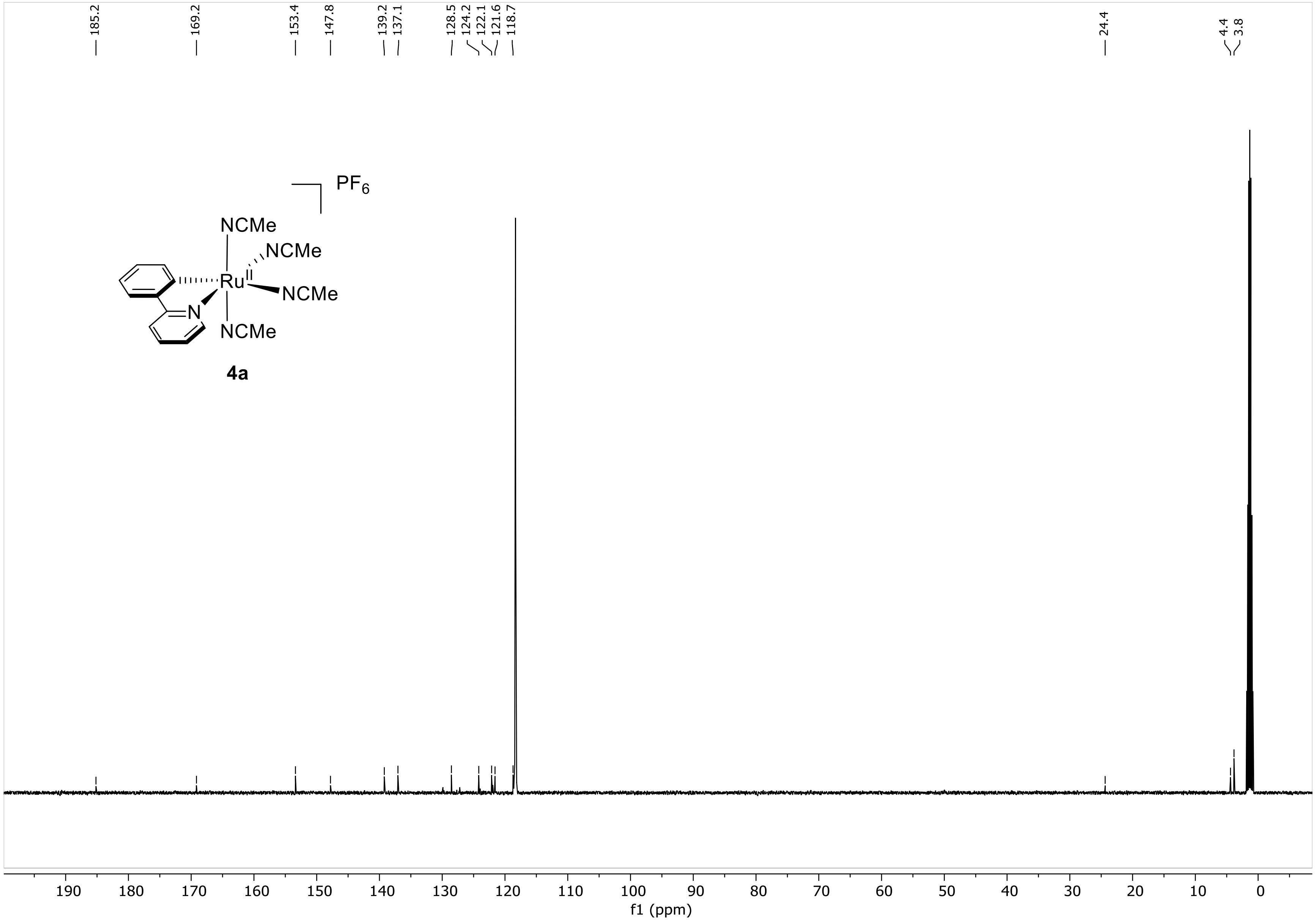

^{13}C NMR spectra (126 MHz, CD_3CN) of cationic mono-cyclometallated ruthenium species **4a**

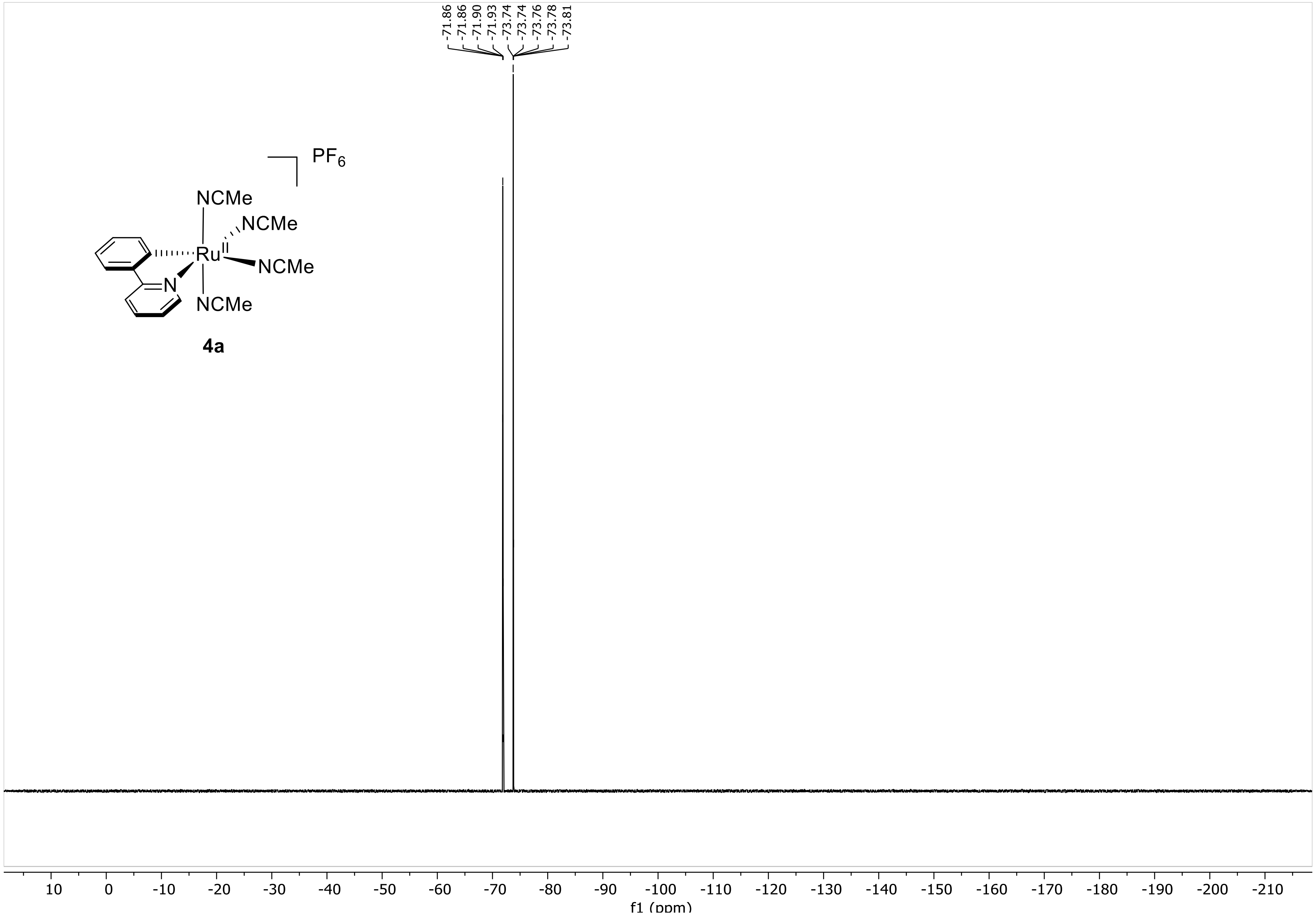

^{19}F NMR spectra (376 MHz, CD_3CN) of cationic mono-cyclometallated ruthenium species **4a**

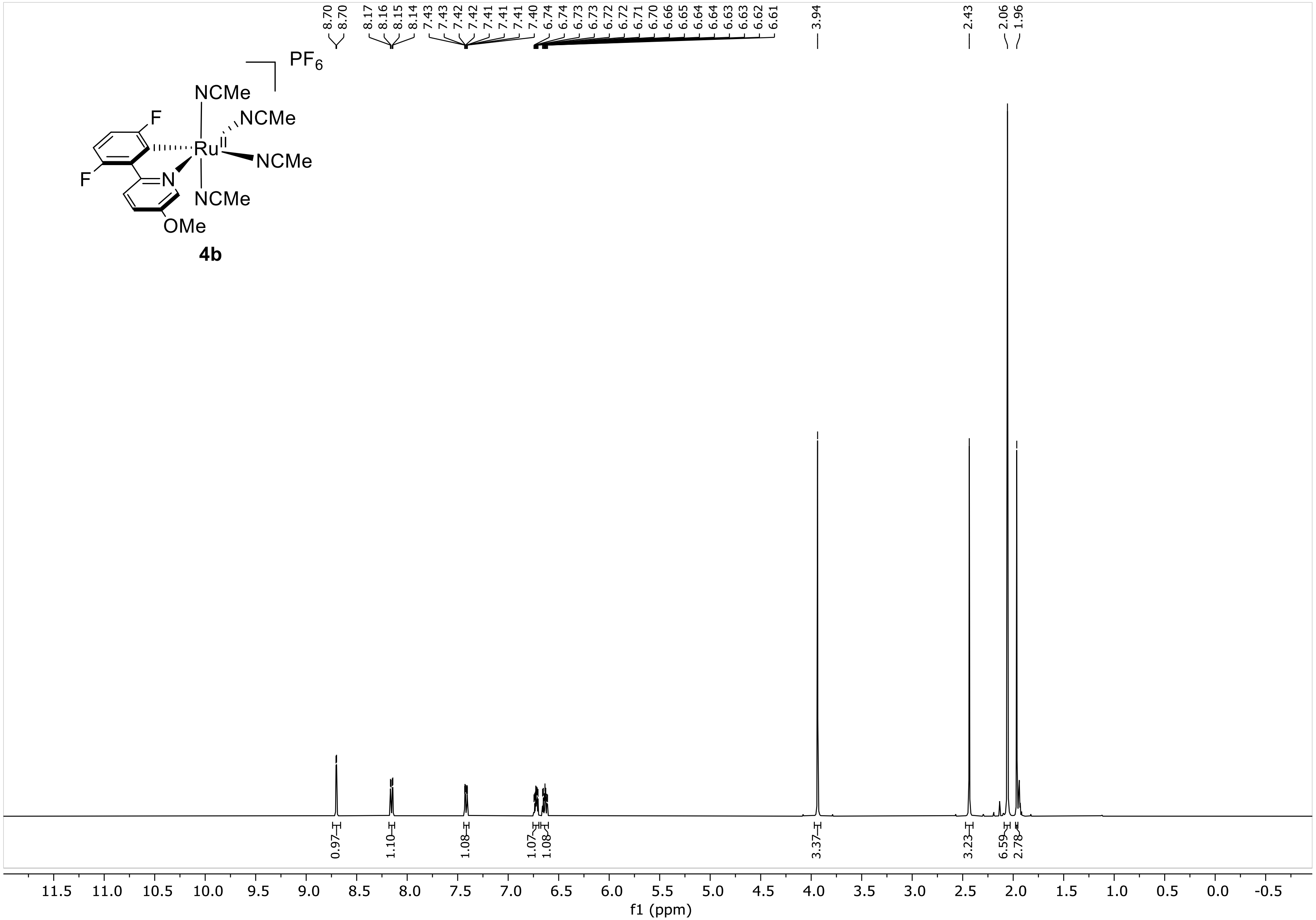

^{1}H NMR spectra (500 MHz, CD_3CN) of cationic mono-cyclometallated ruthenium species **4b**

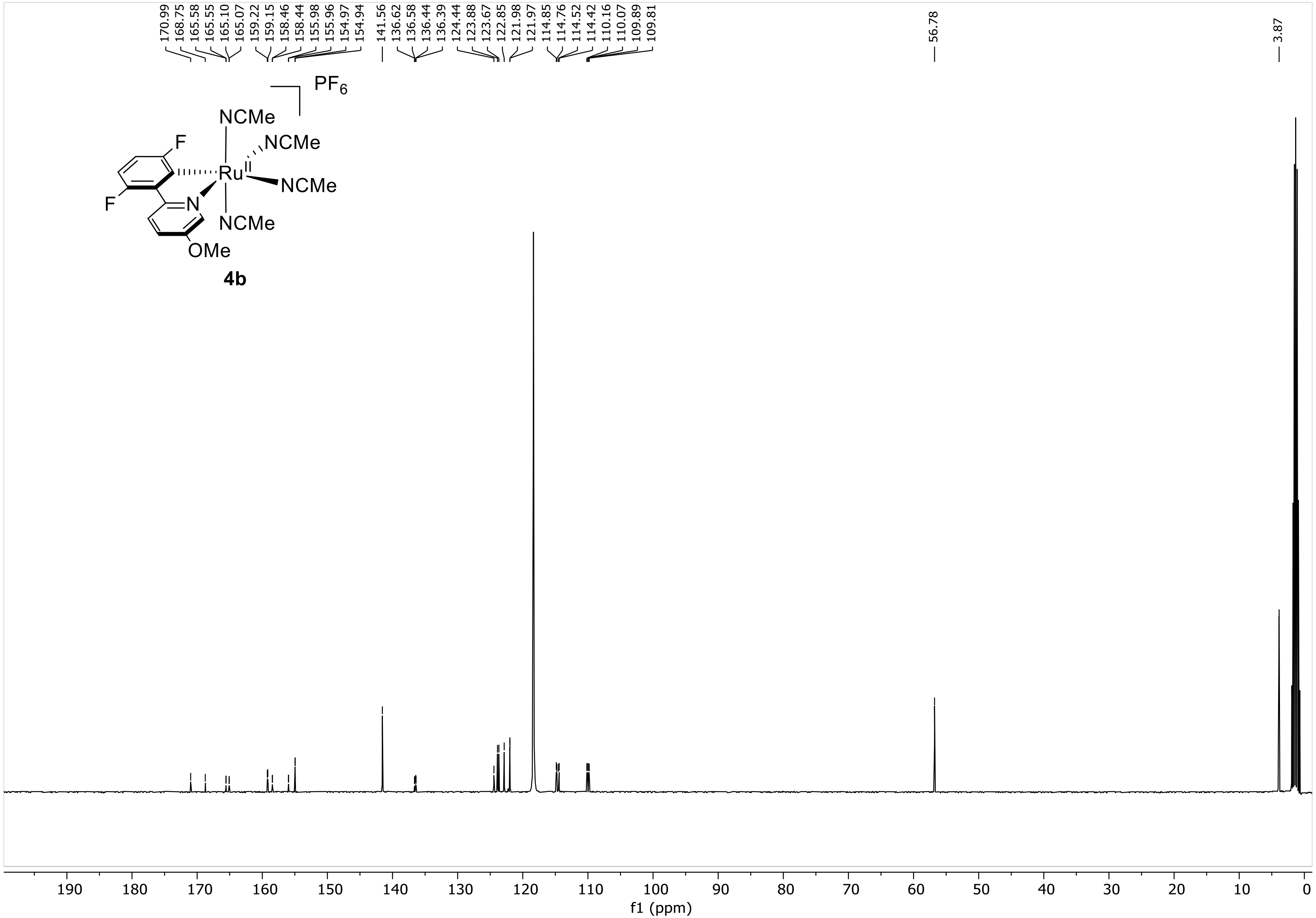

^{13}C NMR spectra (101 MHz, CD_3CN) of cationic mono-cyclometallated ruthenium species **4b**

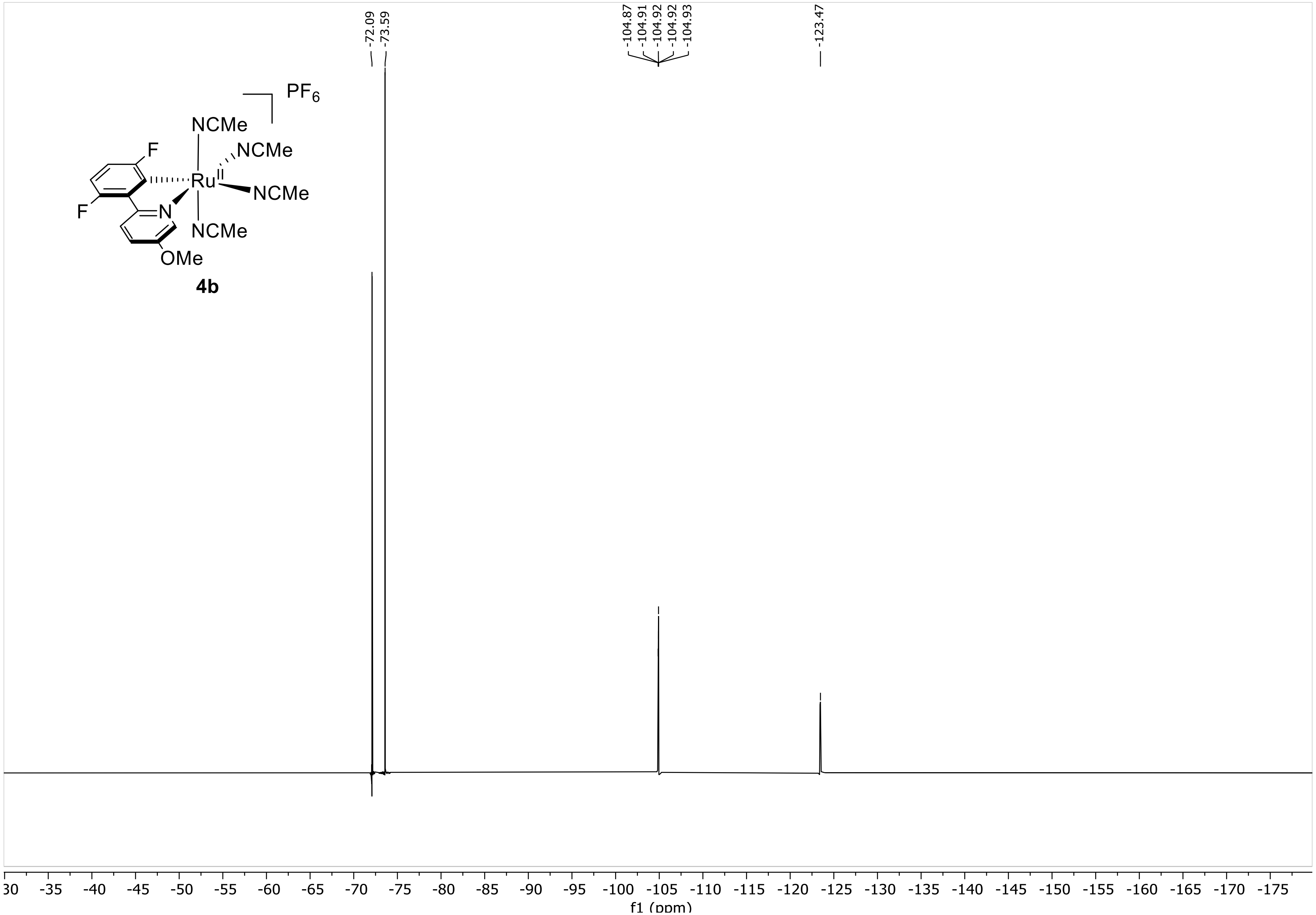

^{19}F NMR spectra (471 MHz, CD_3CN) of cationic mono-cyclometallated ruthenium species **4b**

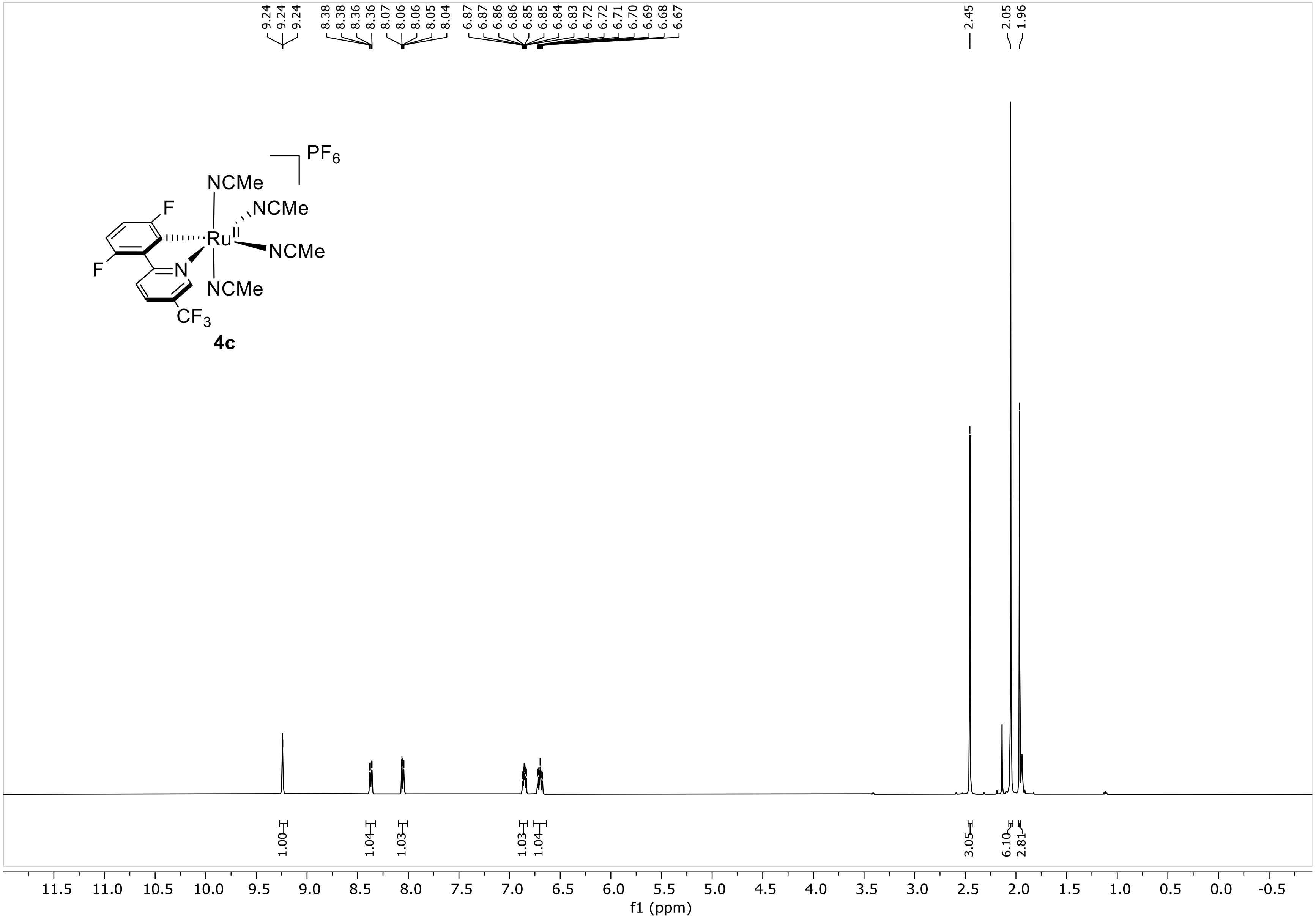

^{1}H NMR spectra (500 MHz, CD_3CN) of cationic mono-cyclometallated ruthenium species **4c**

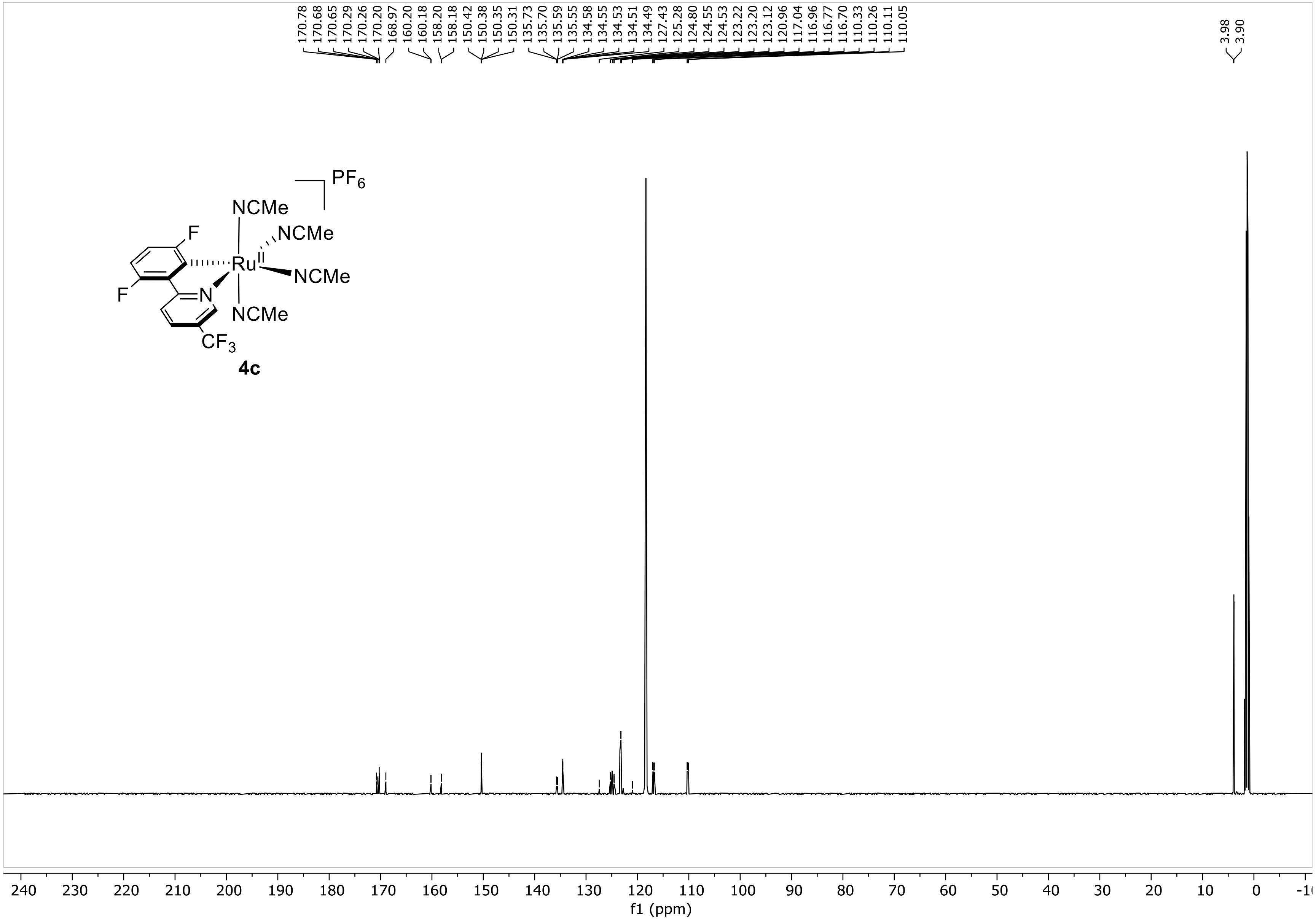

^{13}C NMR spectra (126 MHz, CD_3CN) of cationic mono-cyclometallated ruthenium species **4c**

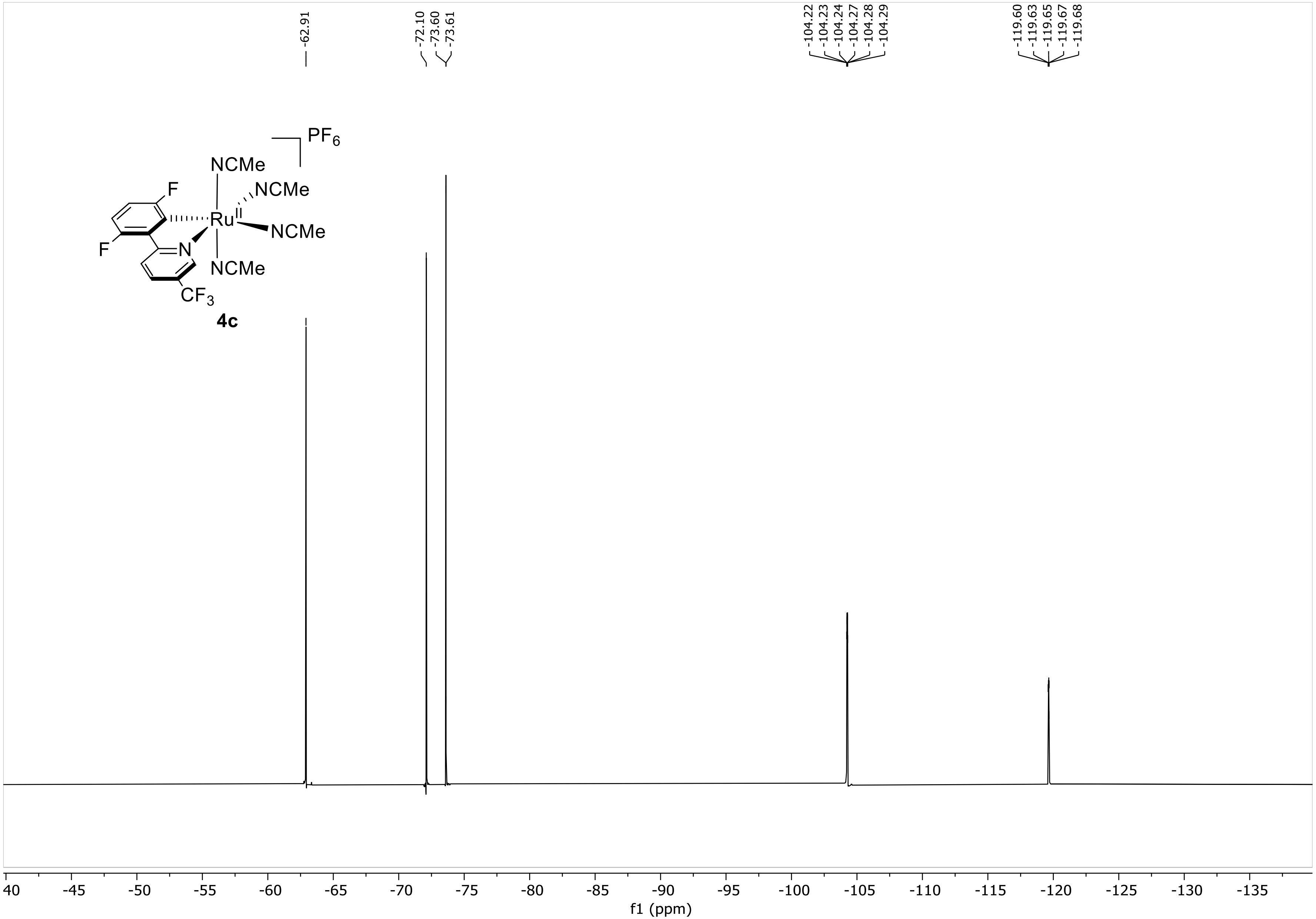

^{19}F NMR spectra (471 MHz, CD_3CN) of cationic mono-cyclometallated ruthenium species **4c**

9.2. Bis-Cyclometallated Ruthenium Species

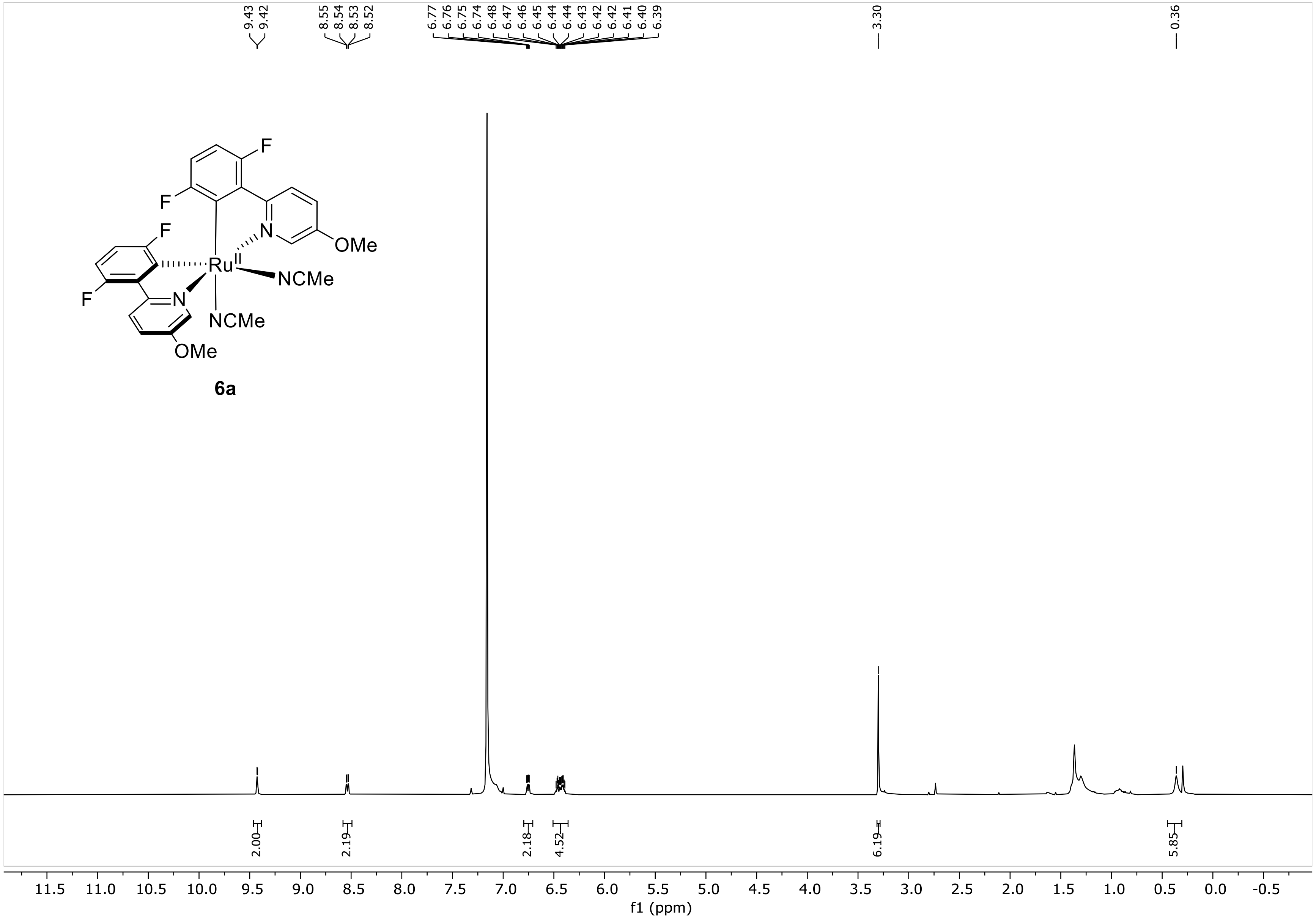

^{1}H NMR spectra (500 MHz, C_6D_6) of **6a**

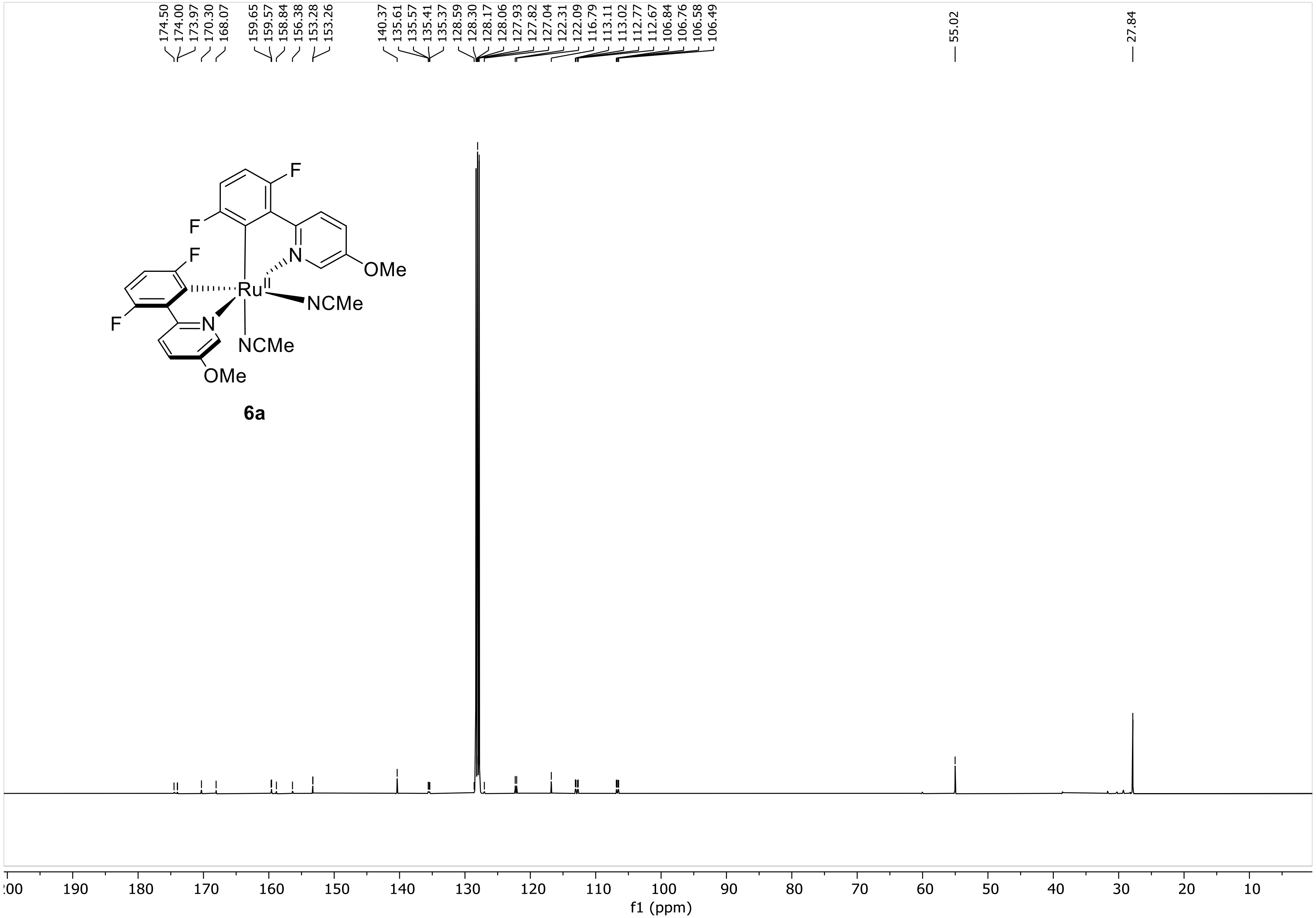

^{13}C NMR spectra (126 MHz, C_6D_6) of **6a**

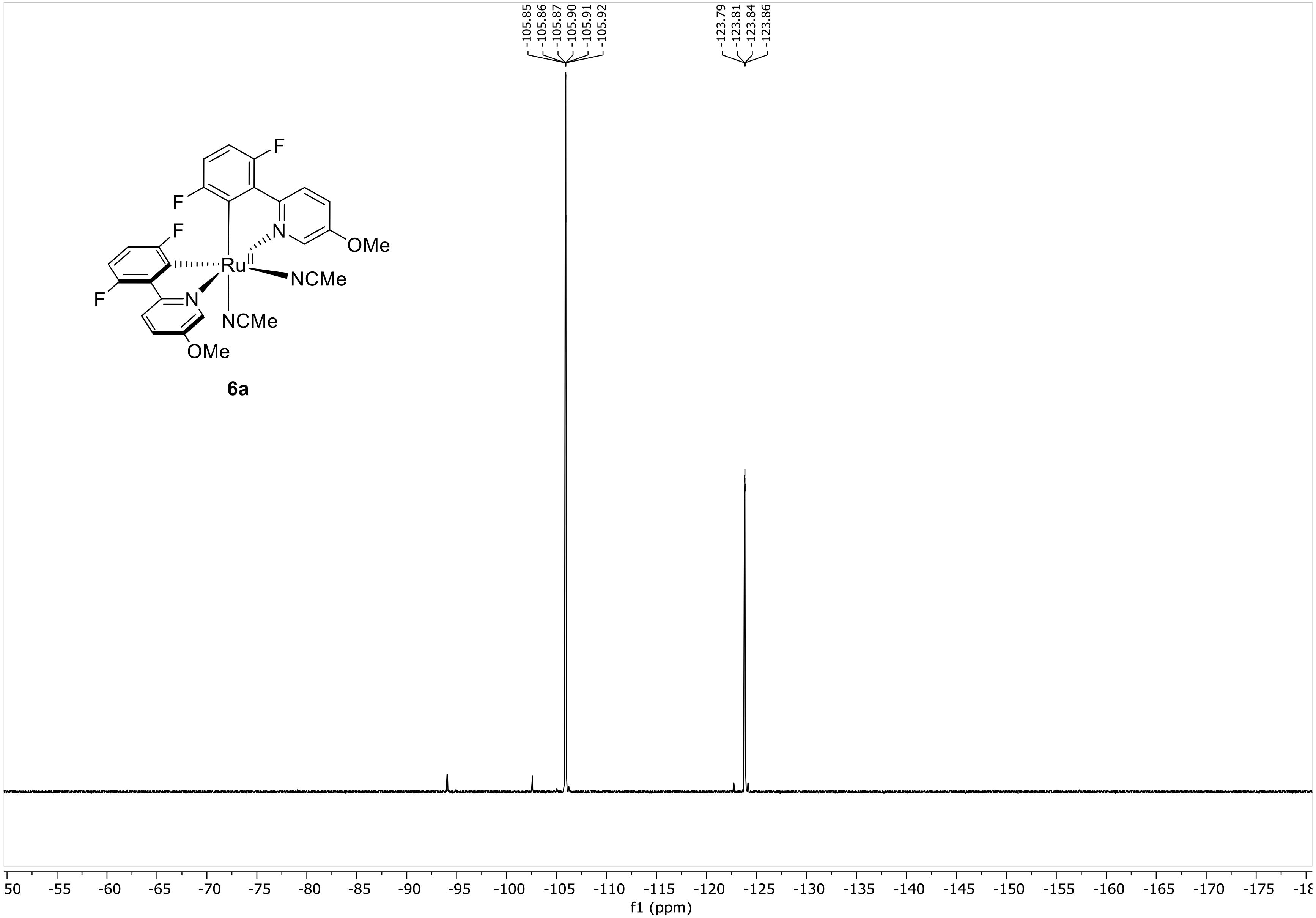

^{19}F NMR spectra (471 MHz, C_6D_6) of **6a**

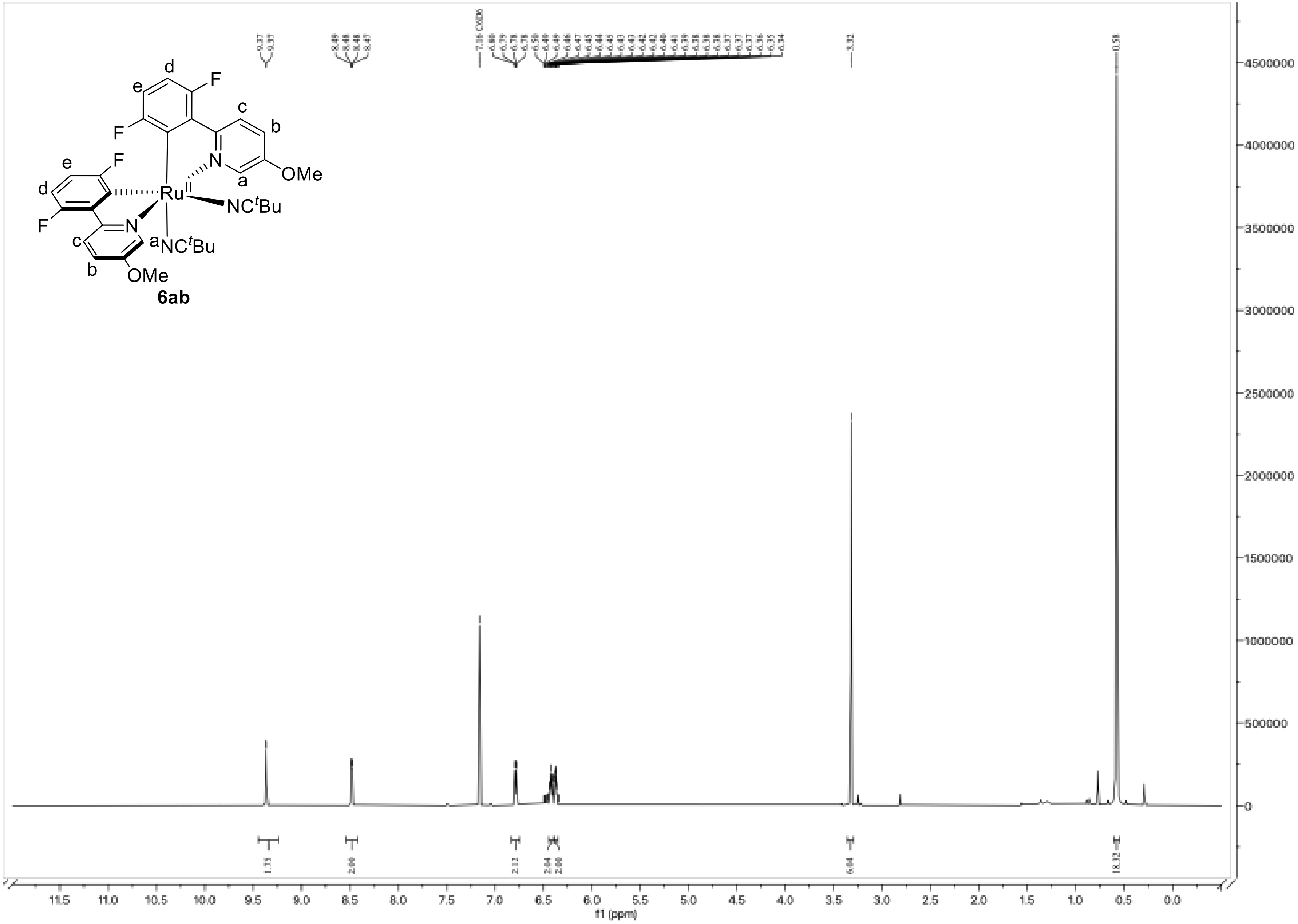

^{1}H NMR spectra (700 MHz, C_6D_6) of **6ab**

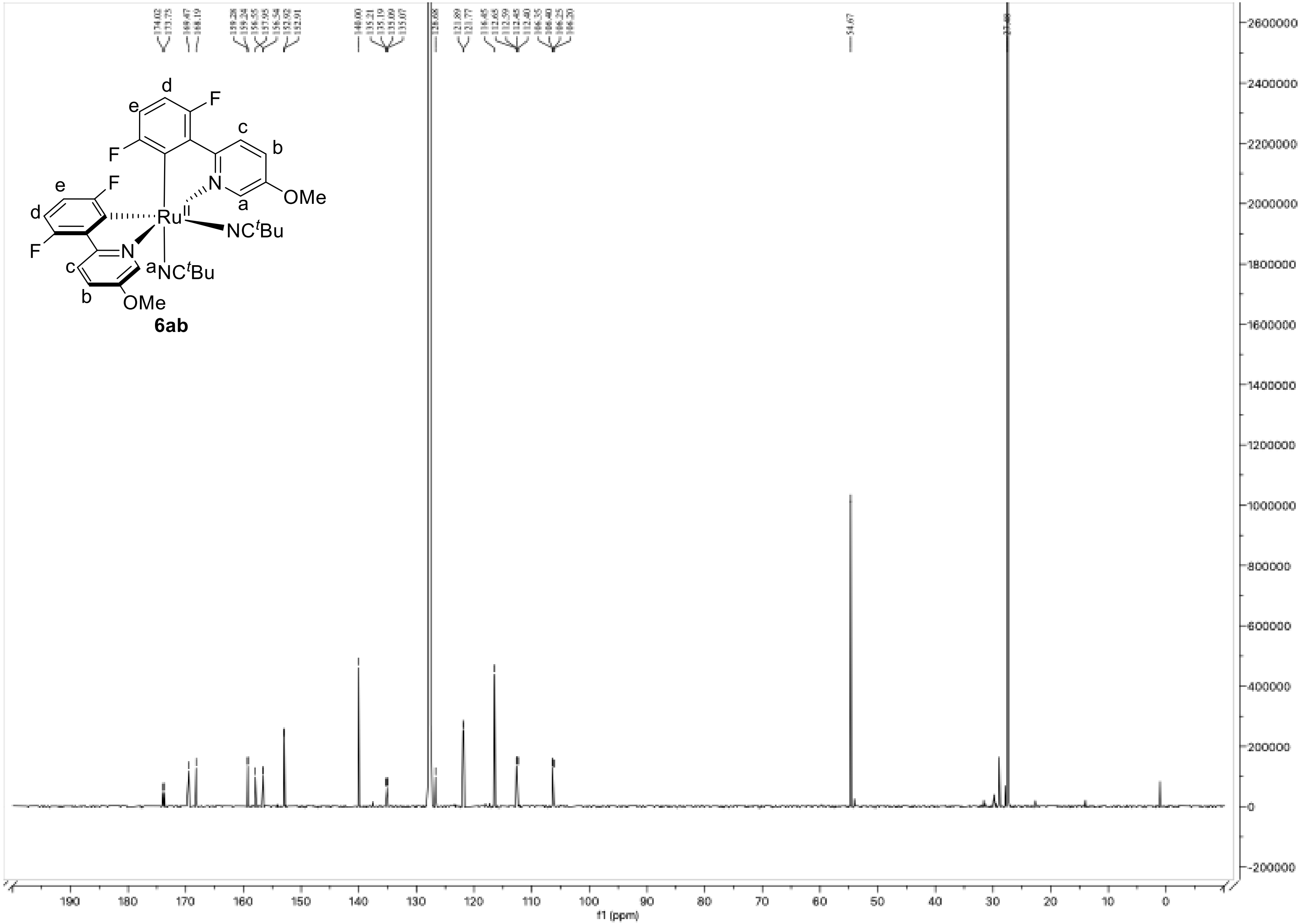

^{13}C NMR spectra (176 MHz, C_6D_6) of **6ab**

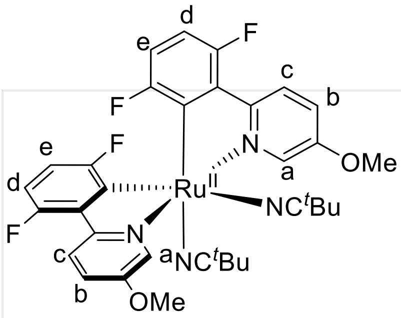

6ab

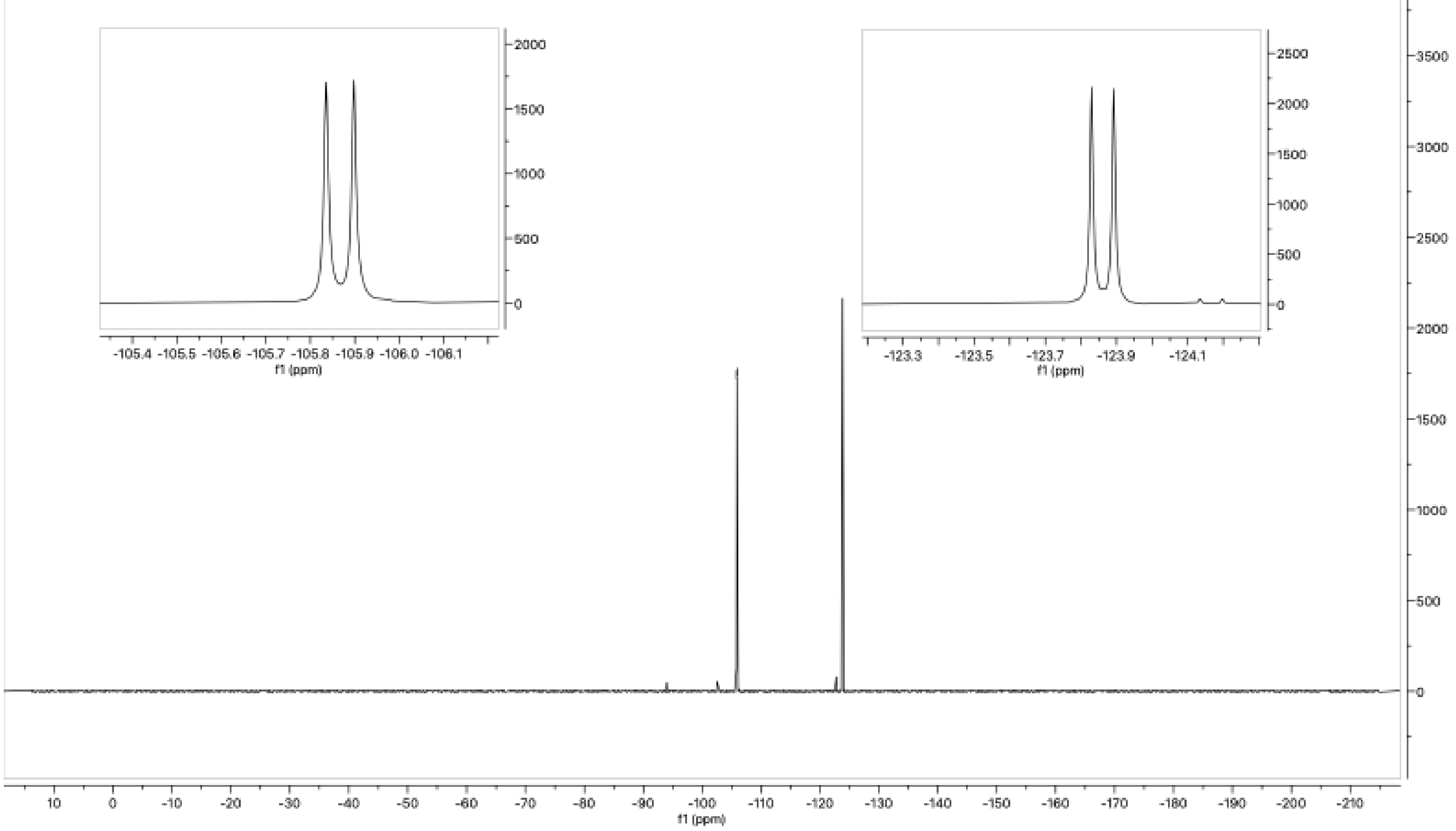

^{19}F NMR Spectra (376 MHz, C_6D_6) of **6ab**

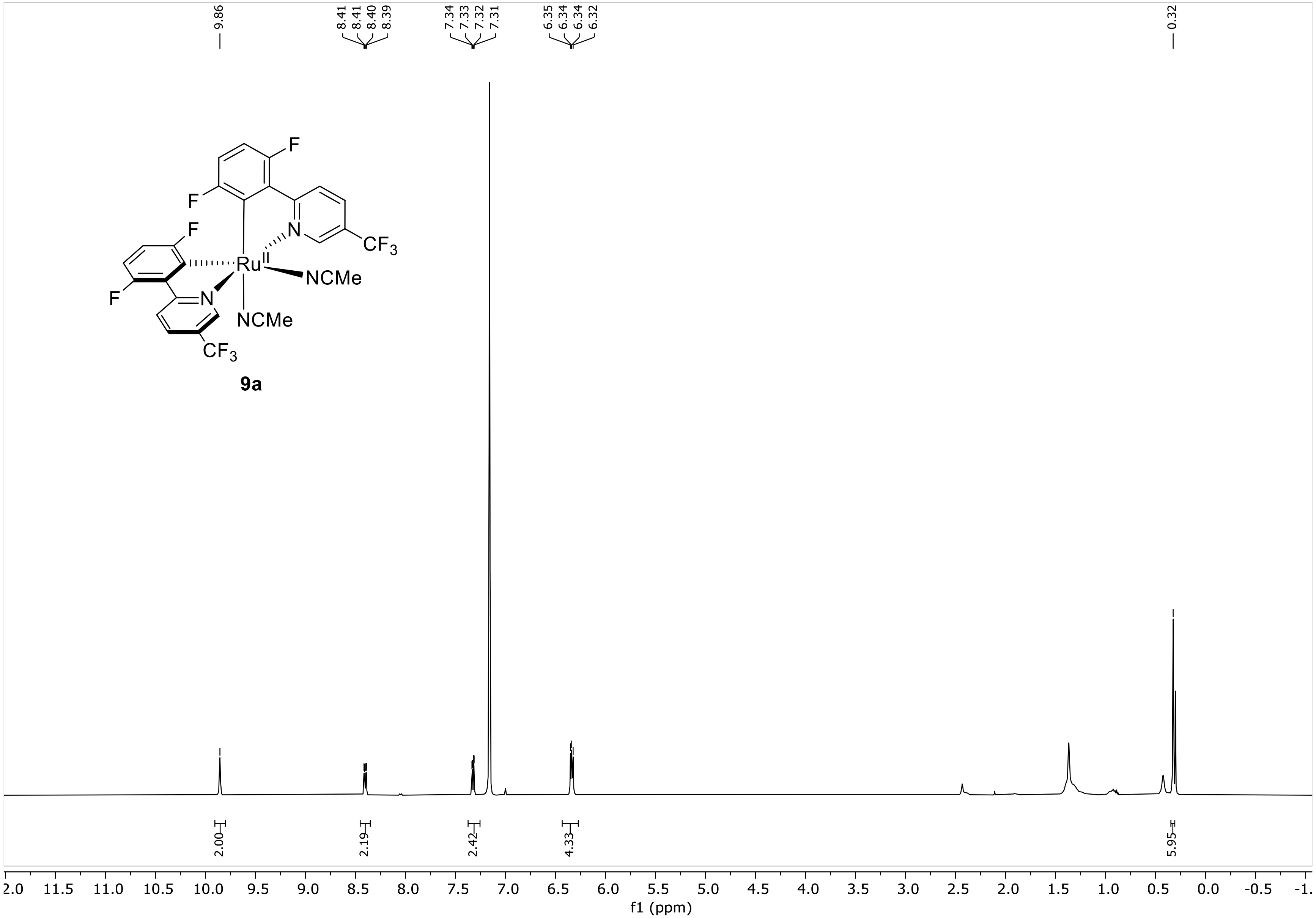

^{1}H NMR spectra (500 MHz, C_6D_6) of **9a**

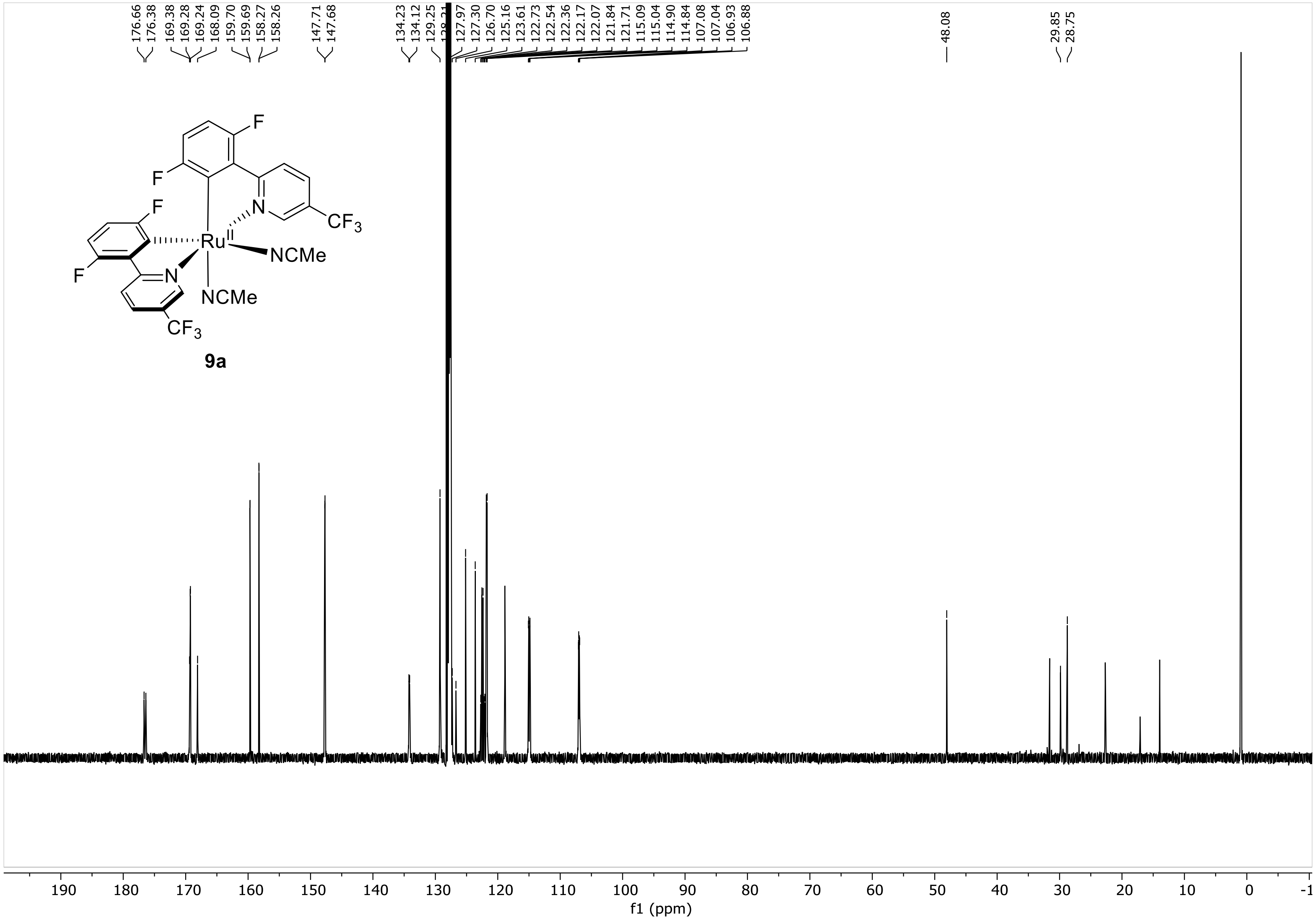

^{13}C NMR spectra (176 MHz, C_6D_6) of **9a**

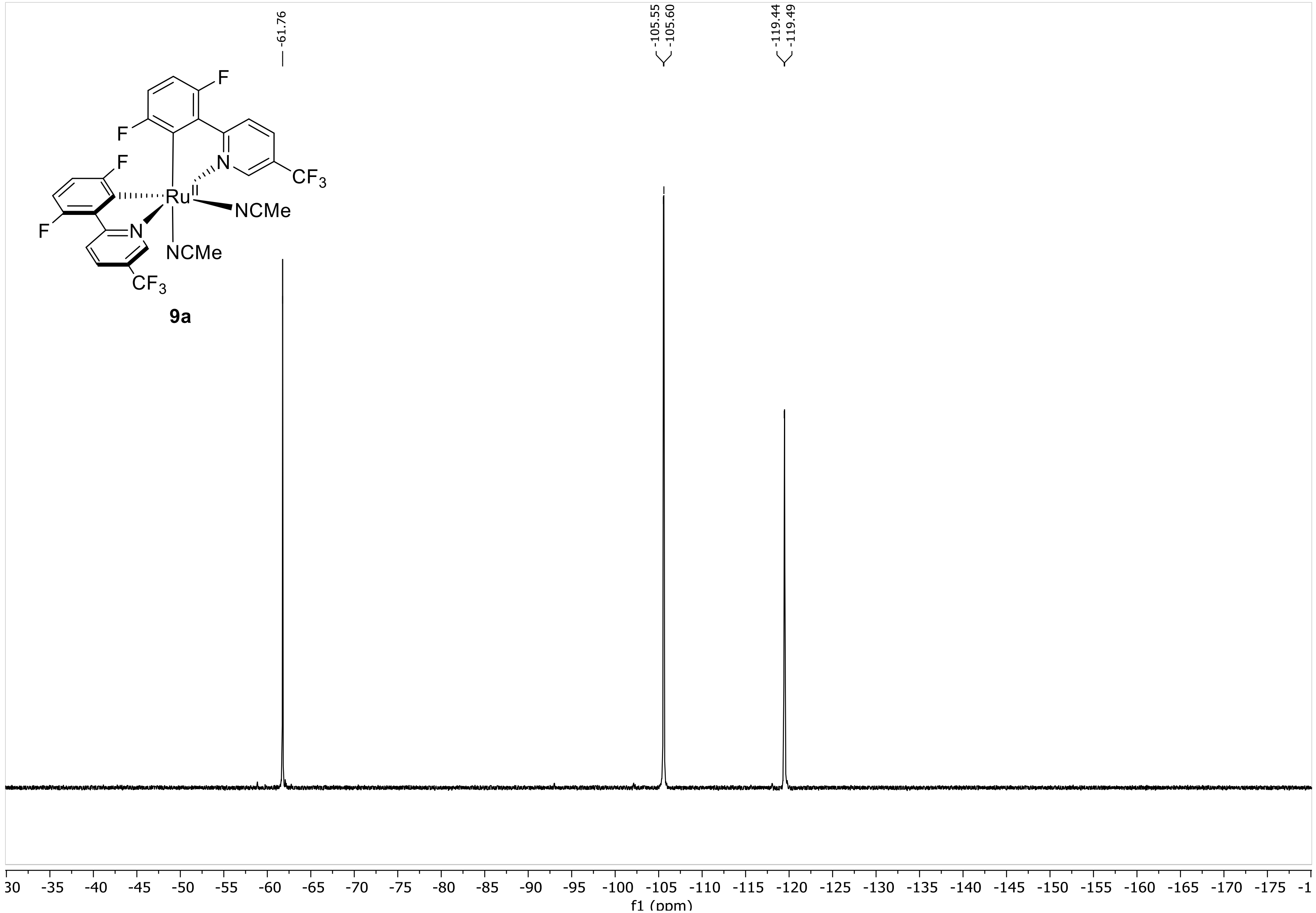

^{19}F NMR spectra (471 MHz, C_6D_6) of **9a**

9.3. Phenylpyridine Derivatives

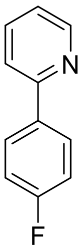

1g

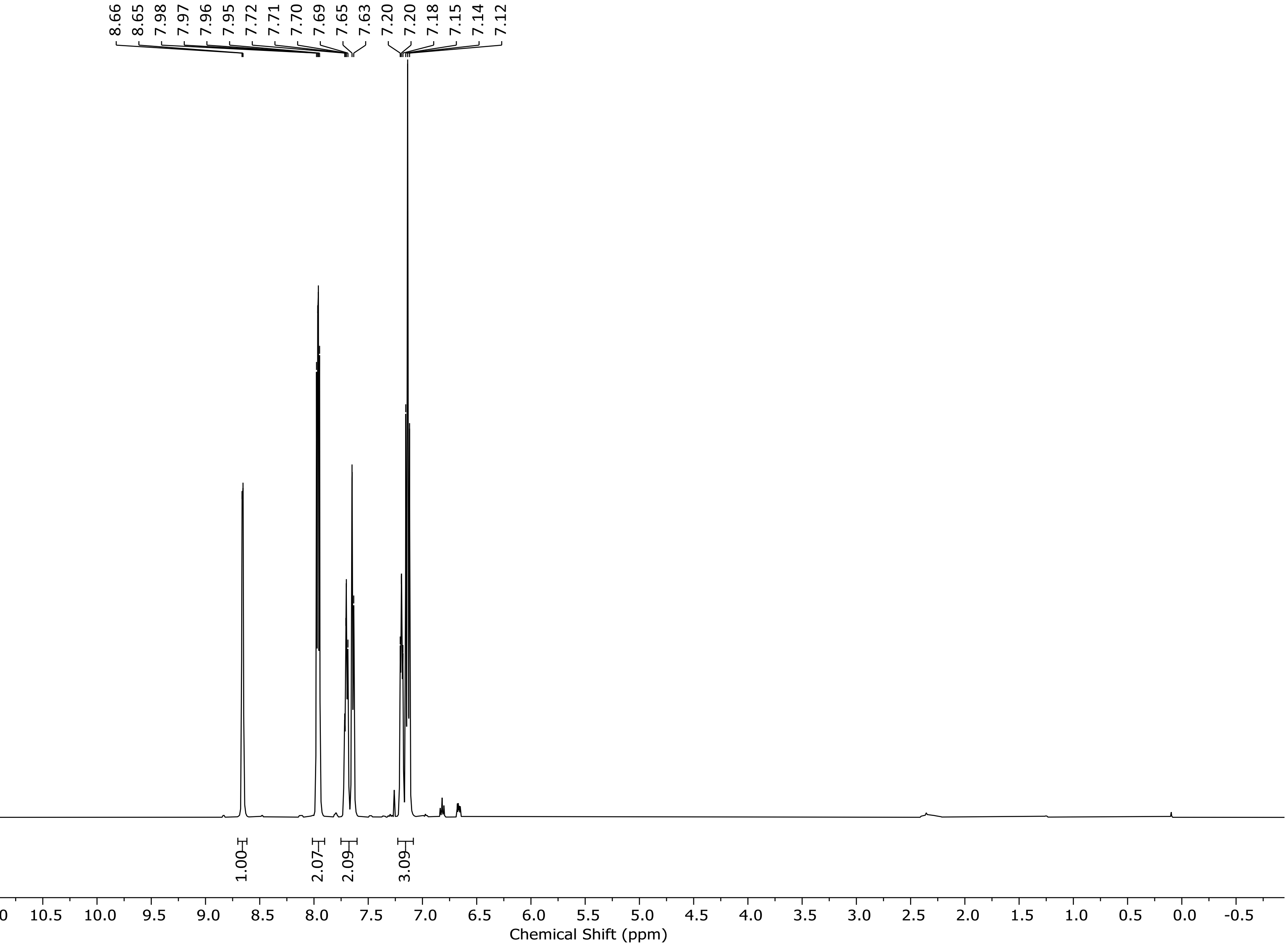

^{1}H NMR spectra (500 MHz, $CDCl_3$) of 2-(4-fluorophenyl)pyridine (**1g**)

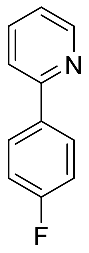

1g

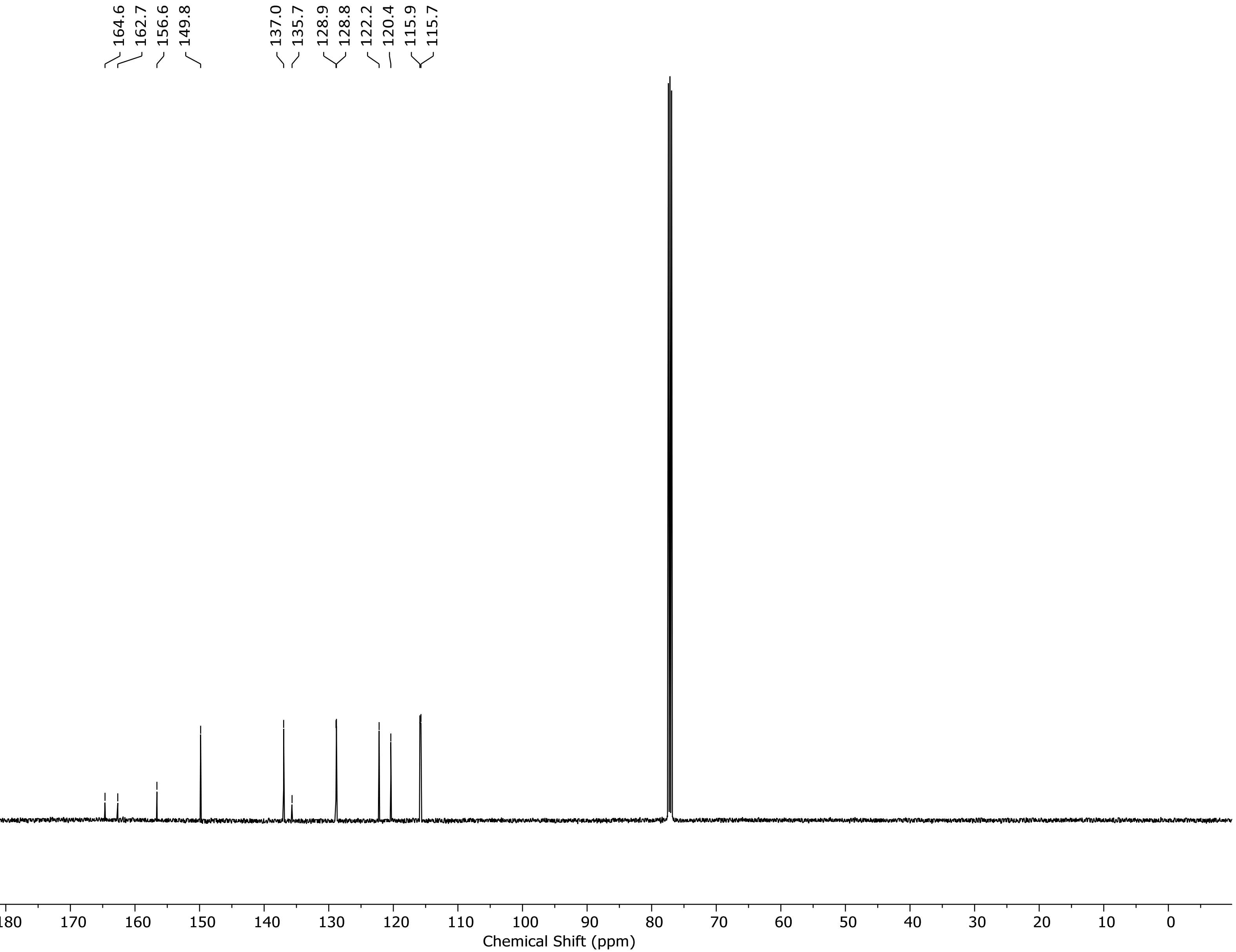

^{13}C NMR spectra (126 MHz, $CDCl_3$) of 2-(4-fluorophenyl)pyridine (**1g**)

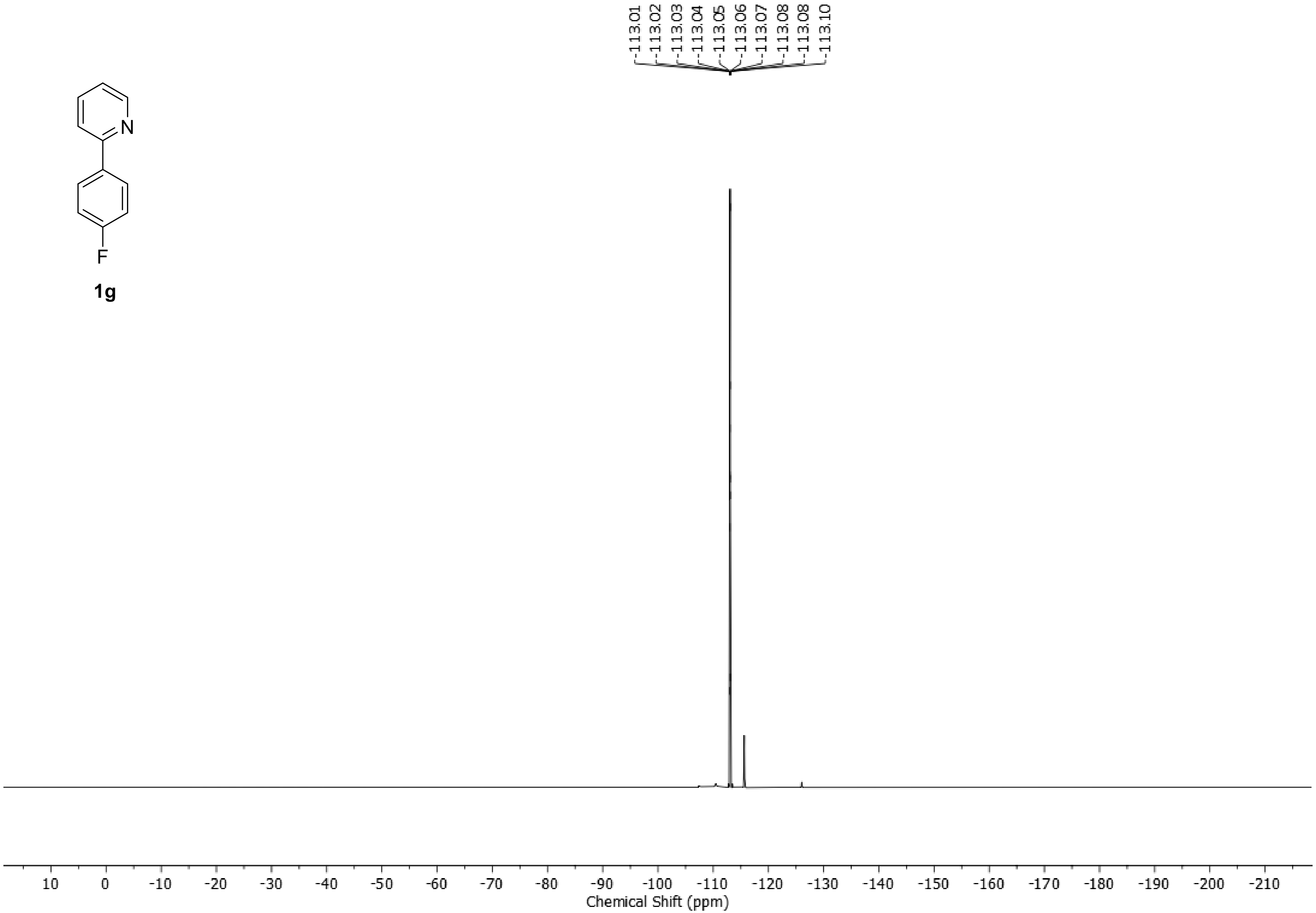

^{19}F NMR spectra (471 MHz, $CDCl_3$) of 2-(4-fluorophenyl)pyridine (**1g**)

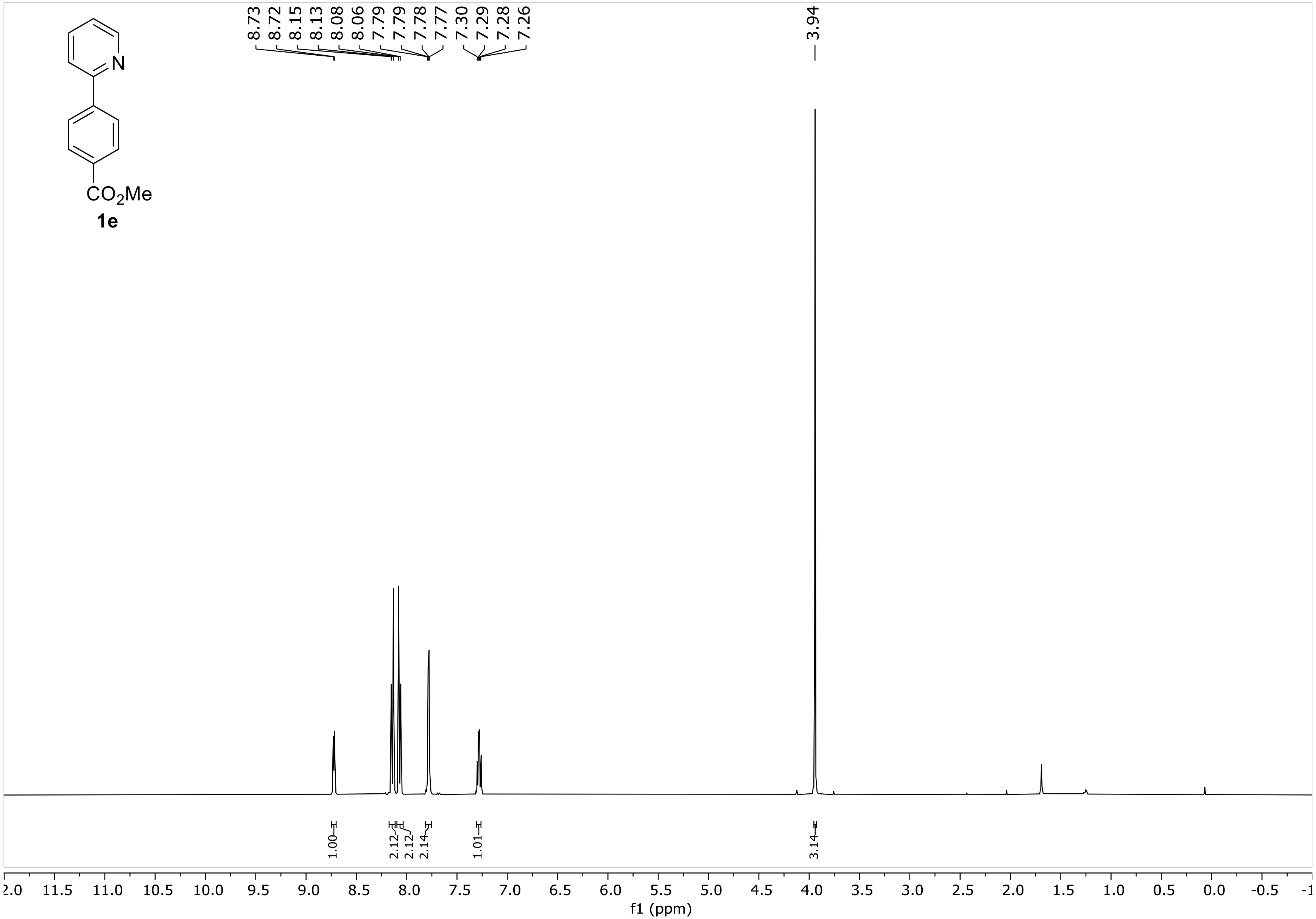

^{1}H NMR spectra (400 MHz, $CDCl_3$) of methyl 4-(pyridin-2-yl)benzoate (**1e**)

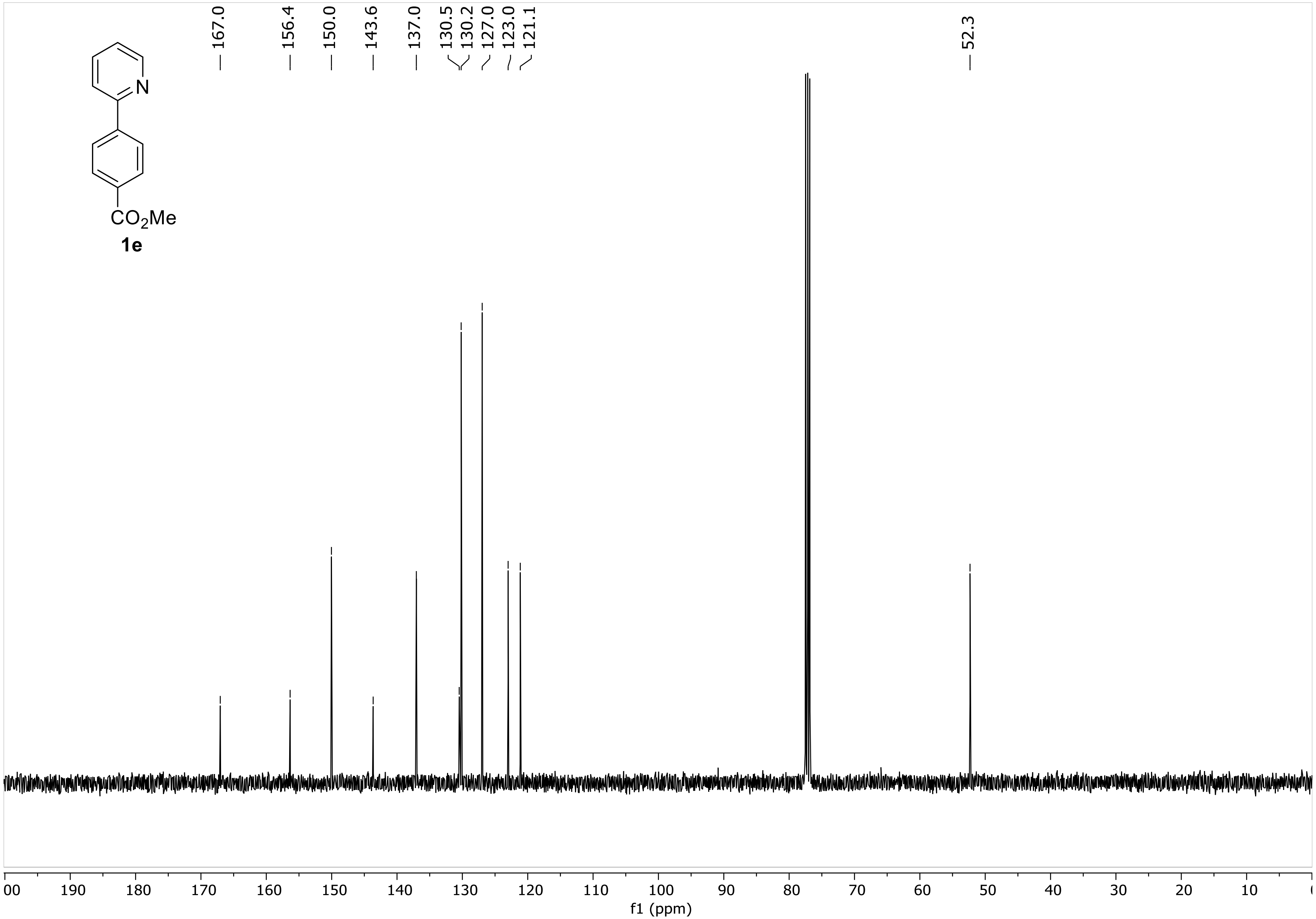

^{13}C NMR spectra (101 MHz, $CDCl_3$) of methyl 4-(pyridin-2-yl)benzoate (**1e**)

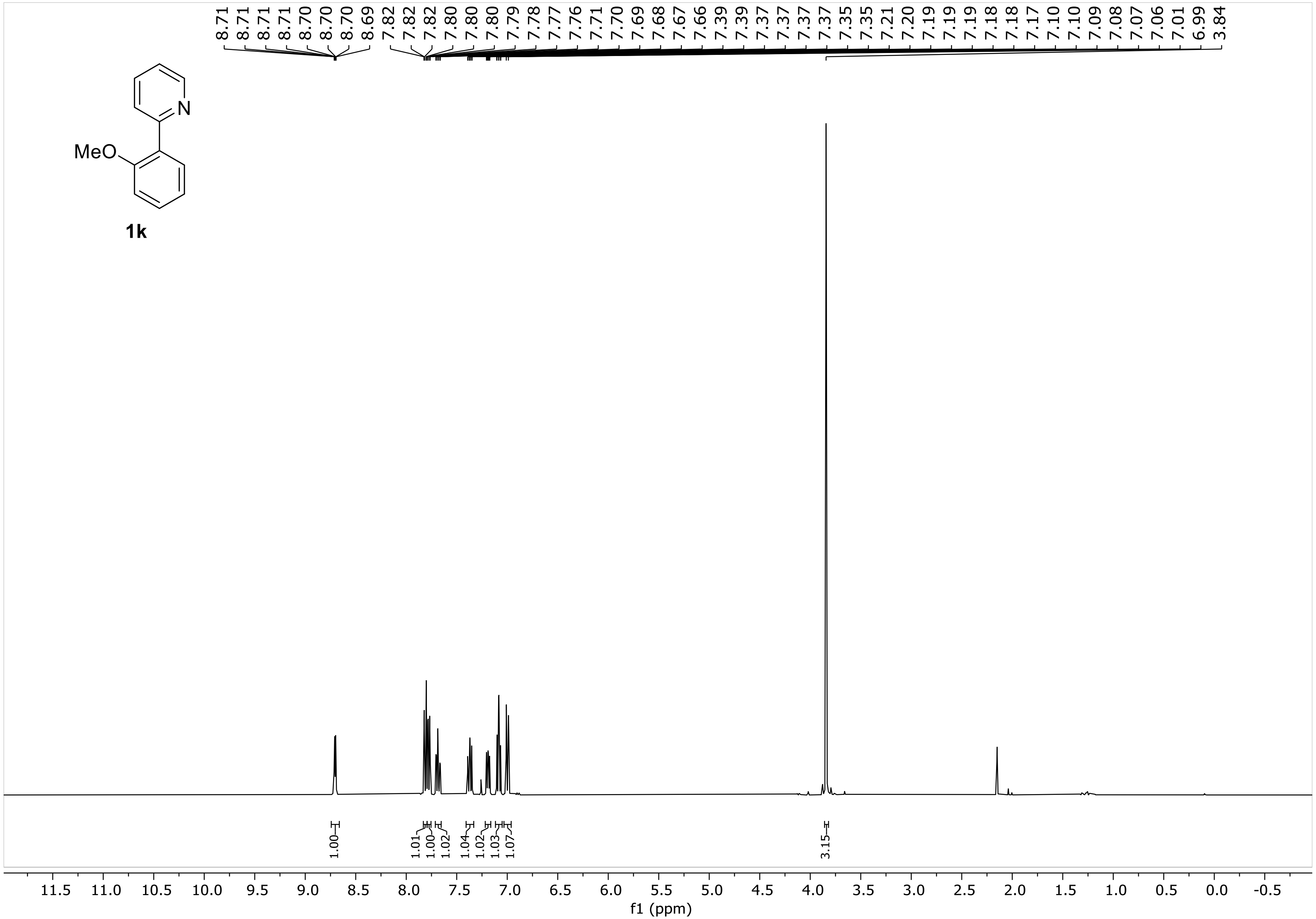

^{1}H NMR spectra (400 MHz, $CDCl_3$) of 2-(2-methoxyphenyl)pyridine (**1k**)

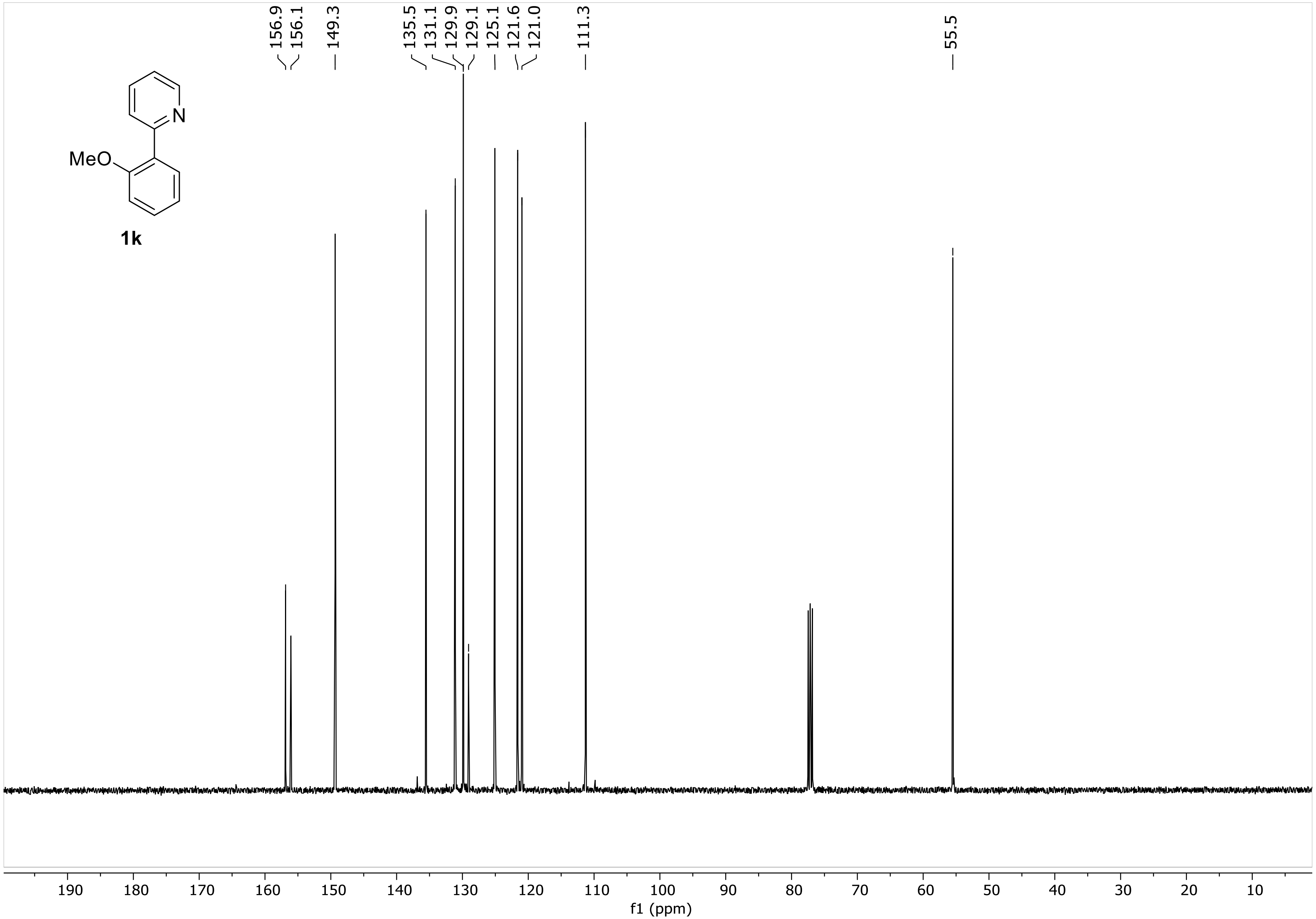

^{13}C NMR spectra (101 MHz, $CDCl_3$) of 2-(2-methoxyphenyl)pyridine (**1k**)

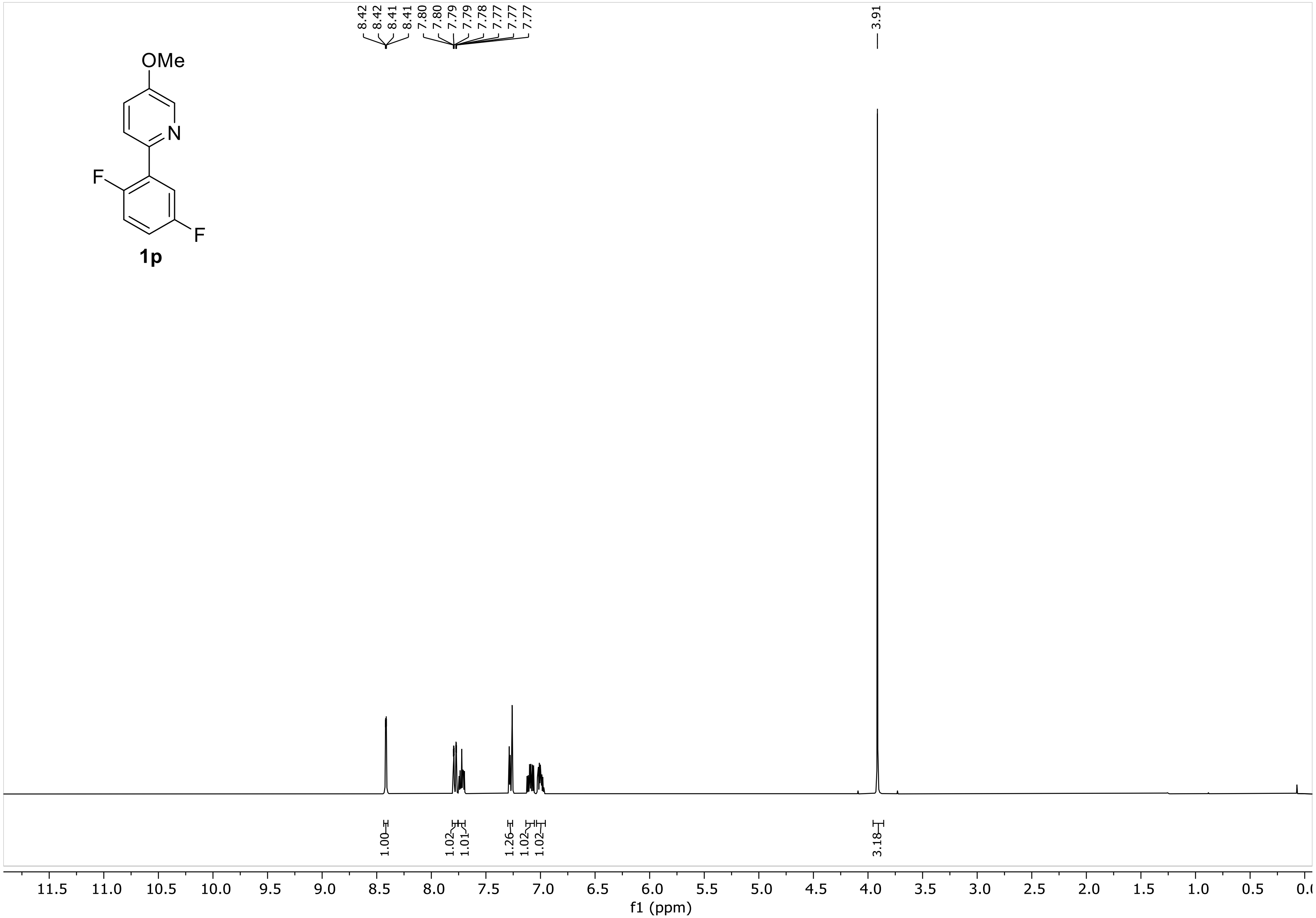

^{1}H NMR spectra (400 MHz, $CDCl_3$) of 2-(2,5-difluorophenyl)-5-methoxypyridine (**1p**)

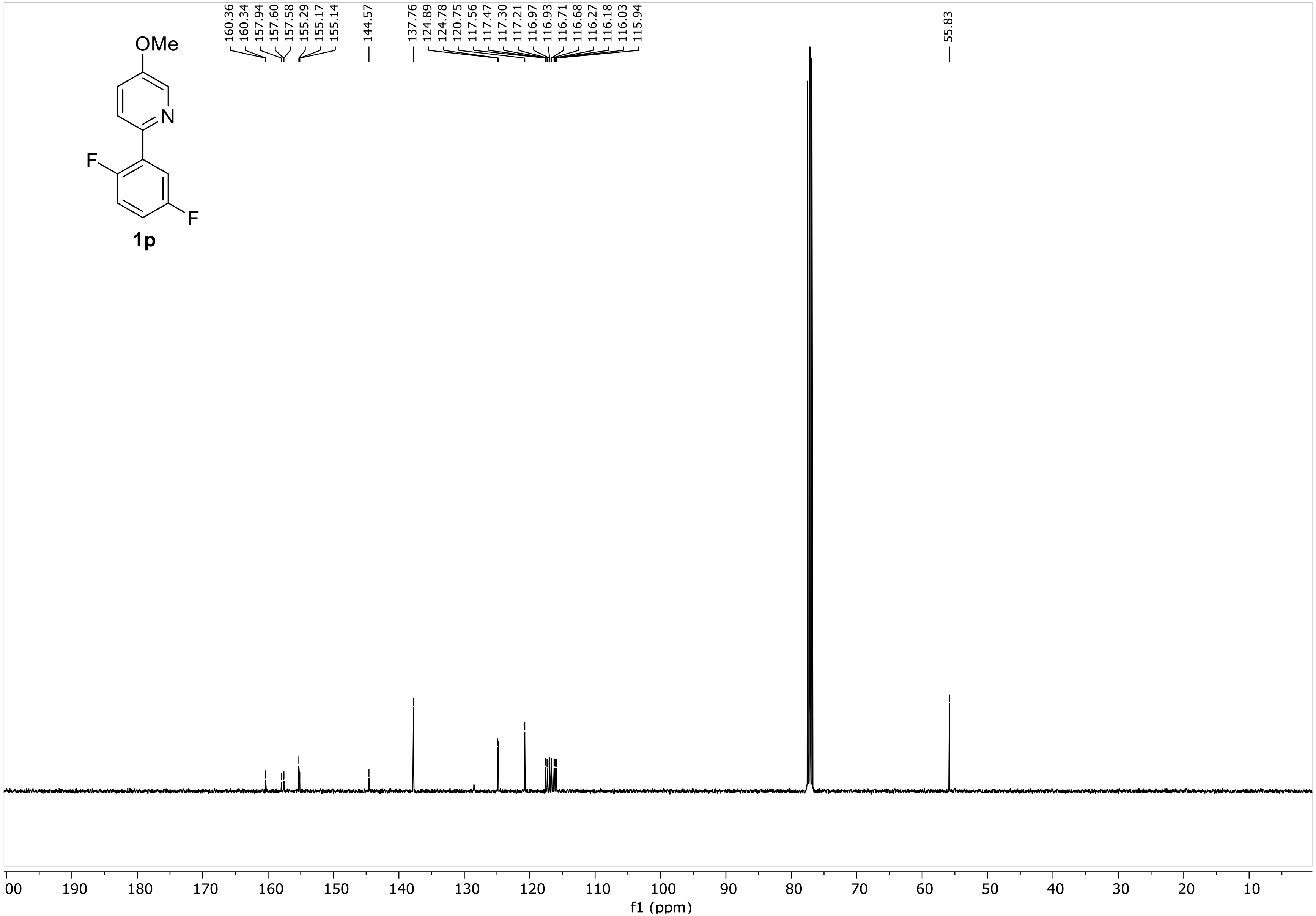

^{13}C NMR spectra (101 MHz, $CDCl_3$) of 2-(2,5-difluorophenyl)-5-methoxypyridine (**1p**)

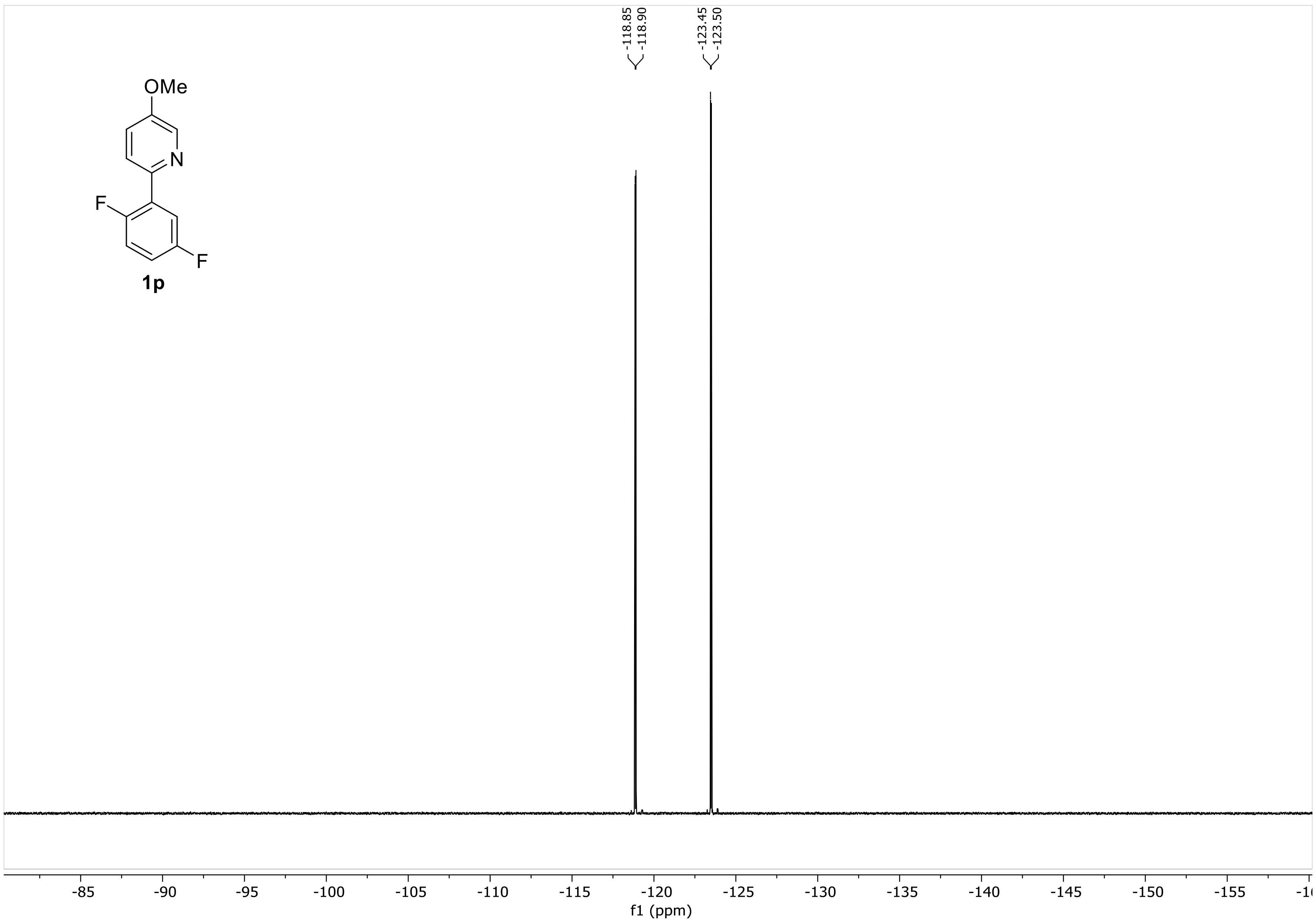

^{19}F NMR spectra (376 MHz, $CDCl_3$) of 2-(2,5-difluorophenyl)-5-methoxypyridine (**1p**)

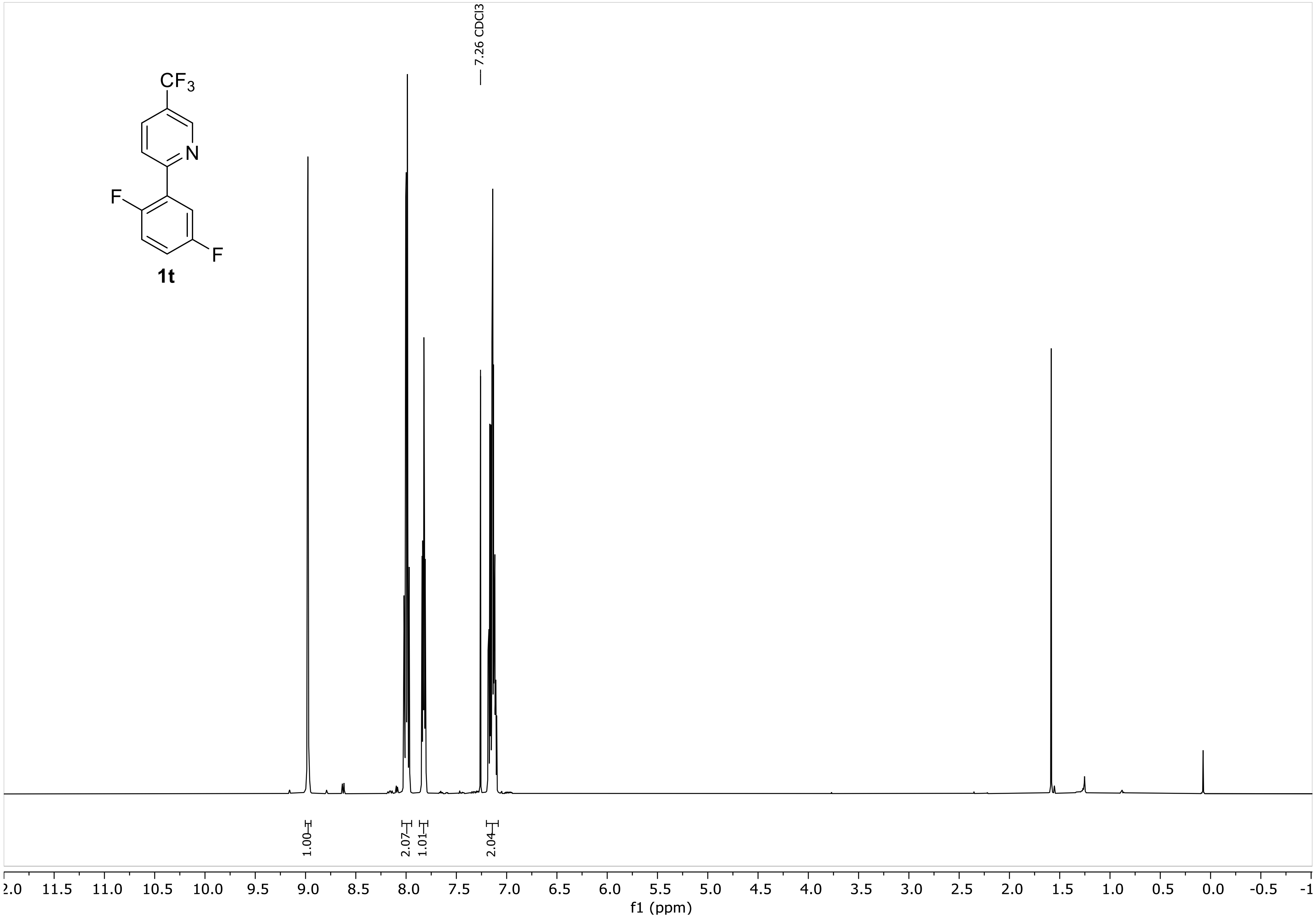

^{1}H NMR spectra (400 MHz, $CDCl_3$) of 2-(2,5-difluorophenyl)-5-trifluoropyridine (**1t**)

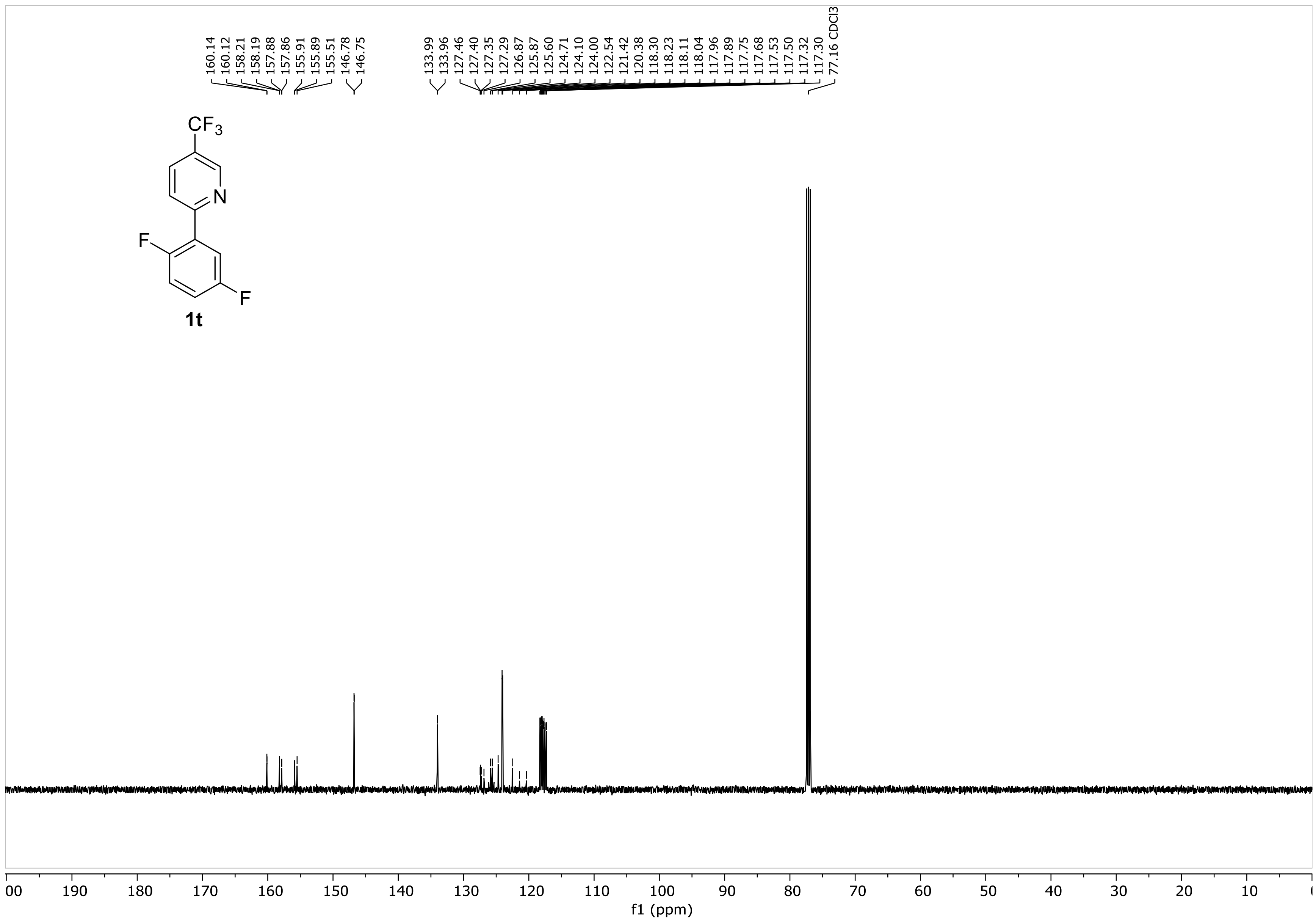

^{13}C NMR spectra (101 MHz, $CDCl_3$) of 2-(2,5-difluorophenyl)-5-trifluoropyridine (**1t**)

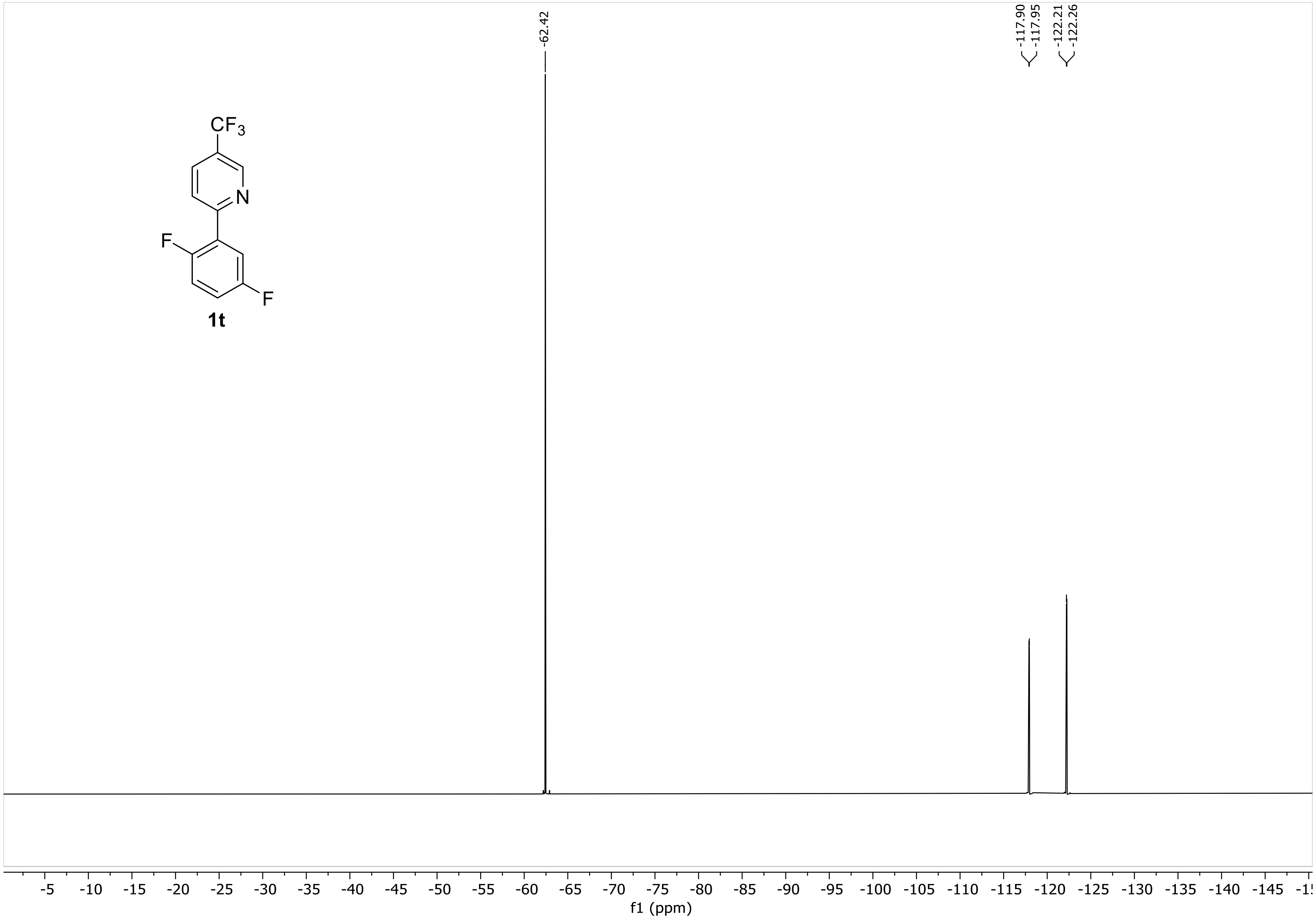

^{19}F NMR spectra (376 MHz, $CDCl_3$) of 2-(2,5-difluorophenyl)-5-trifluoropyridine (**1t**)

9.4. Alkyl Iodide Species

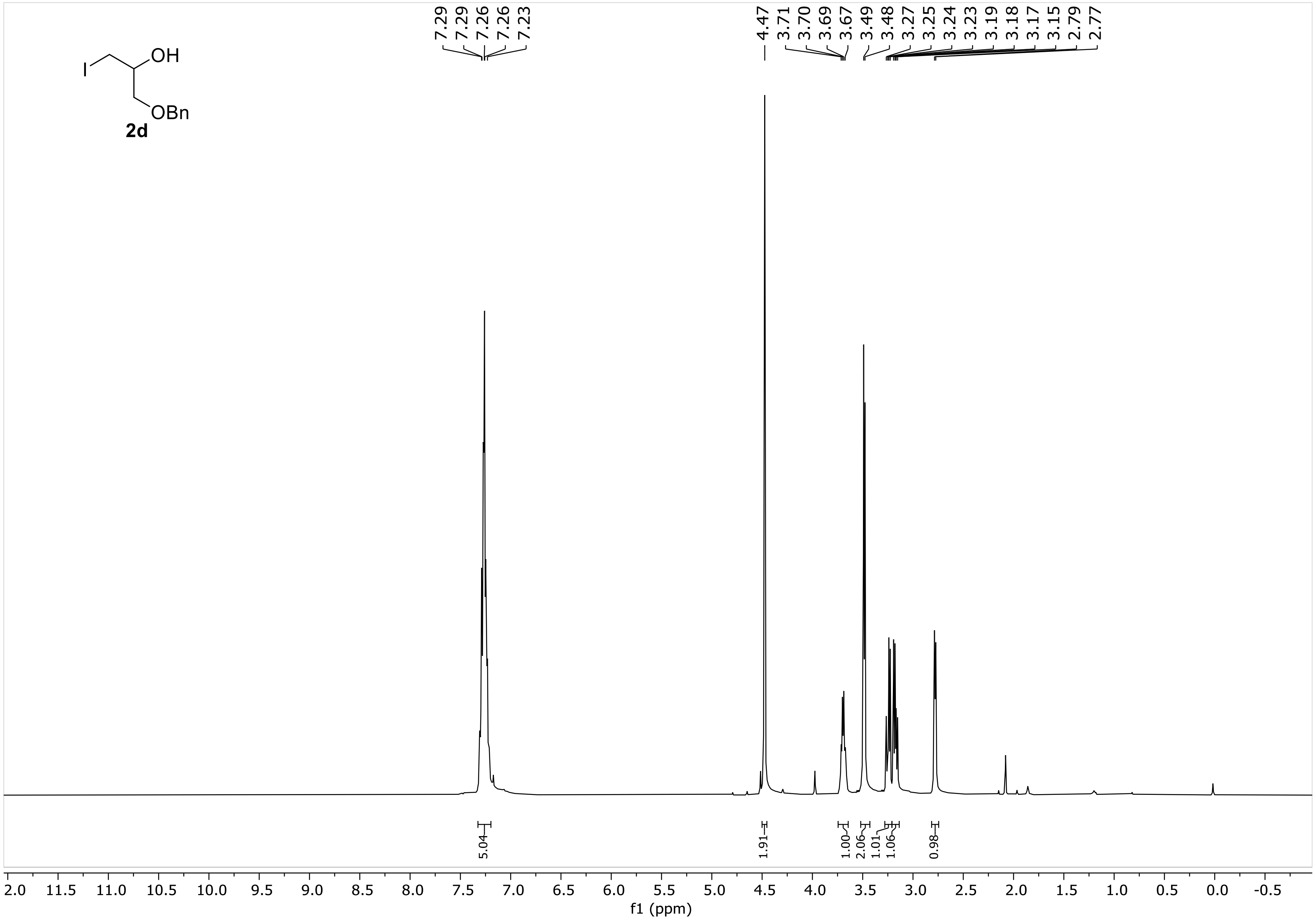

^{1}H NMR spectra (400 MHz, $CDCl_3$) of 1-(benzyloxy)-3-iodopropan-2-ol (**2d**)

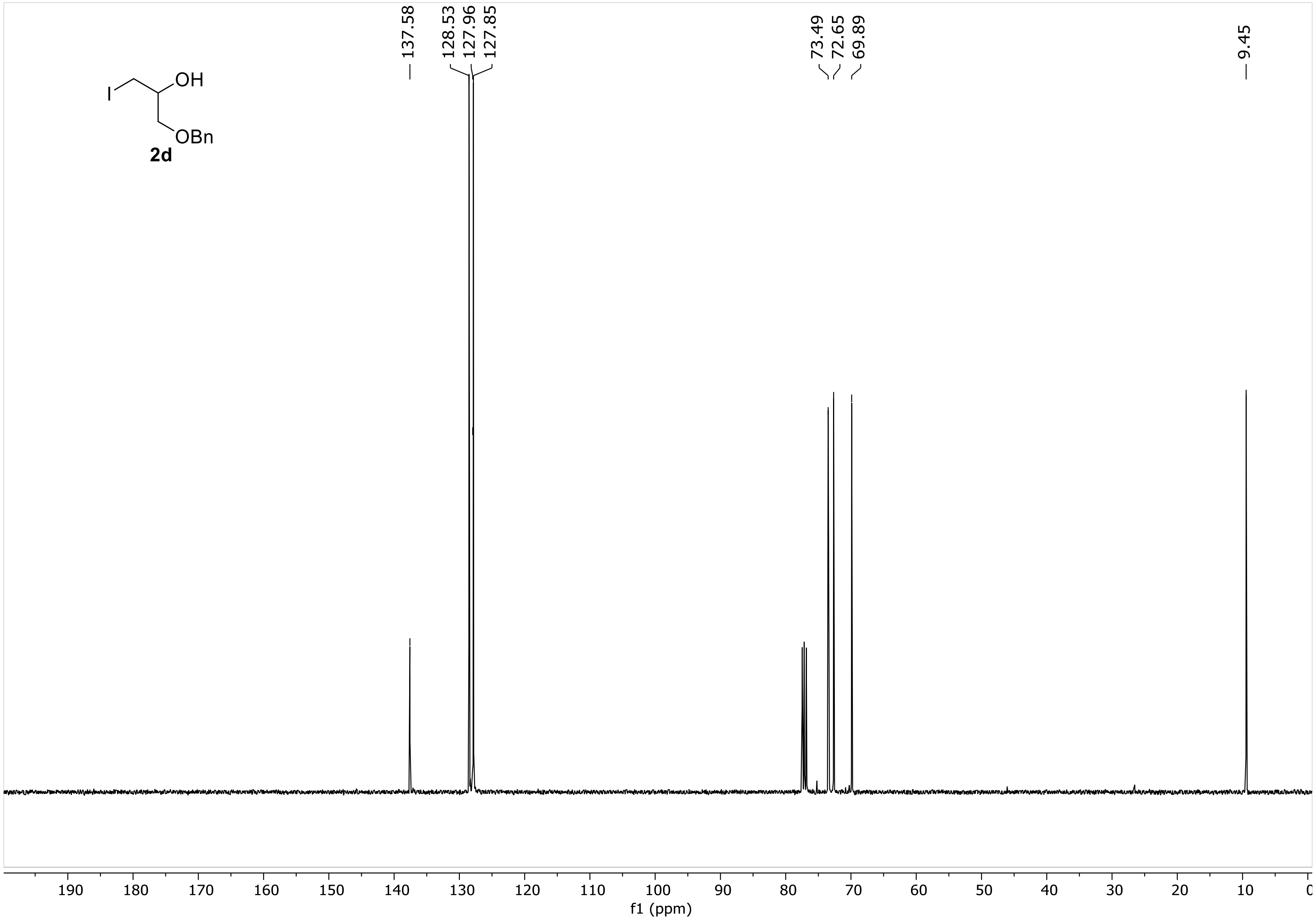

^{13}C NMR spectra (101 MHz, $CDCl_3$) of 1-(benzyloxy)-3-iodopropan-2-ol (**2d**)

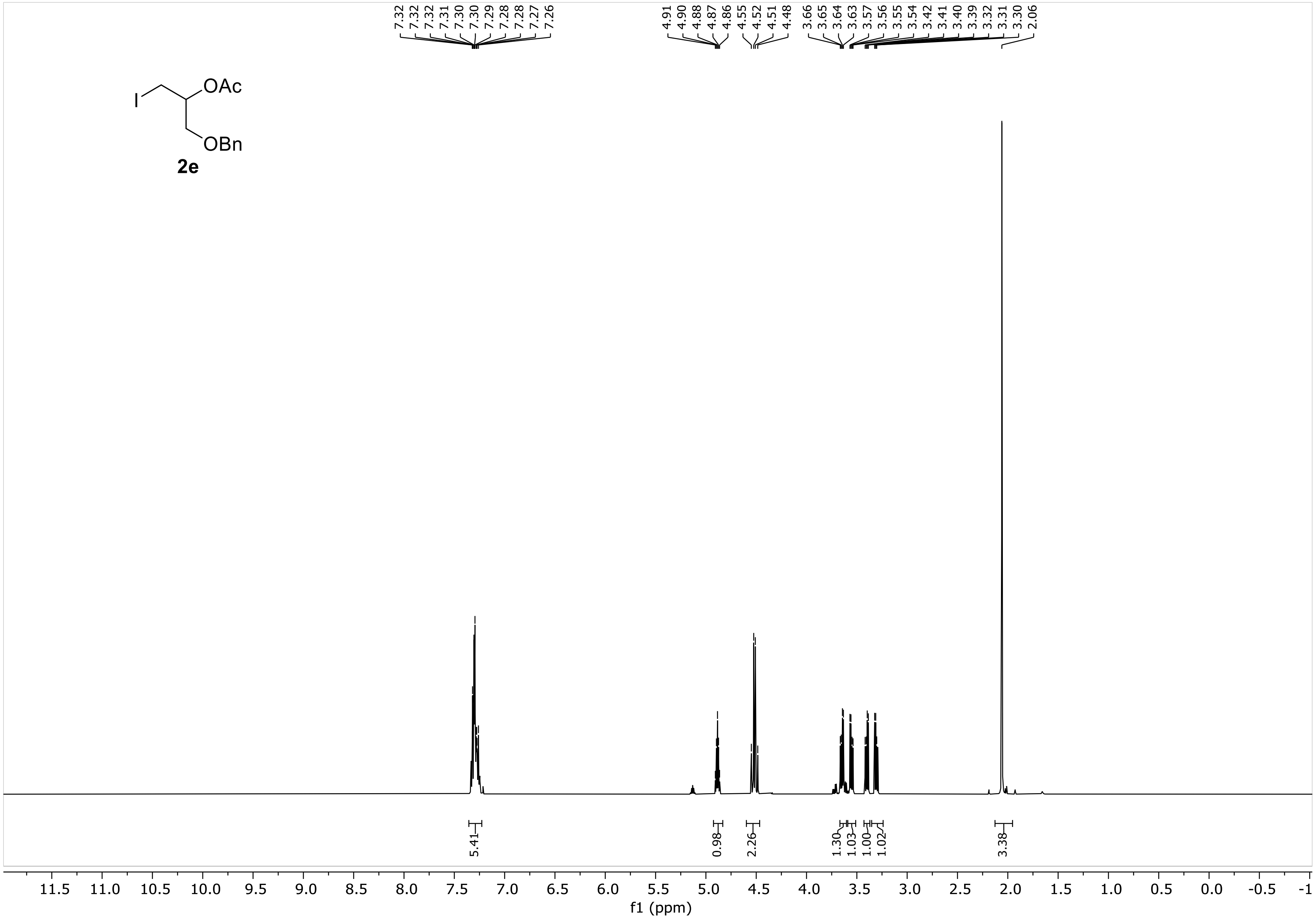

^{1}H NMR spectra (400 MHz, $CDCl_3$) of 1-(benzyloxy)-3-iodopropan-2-yl acetate (**2e**)

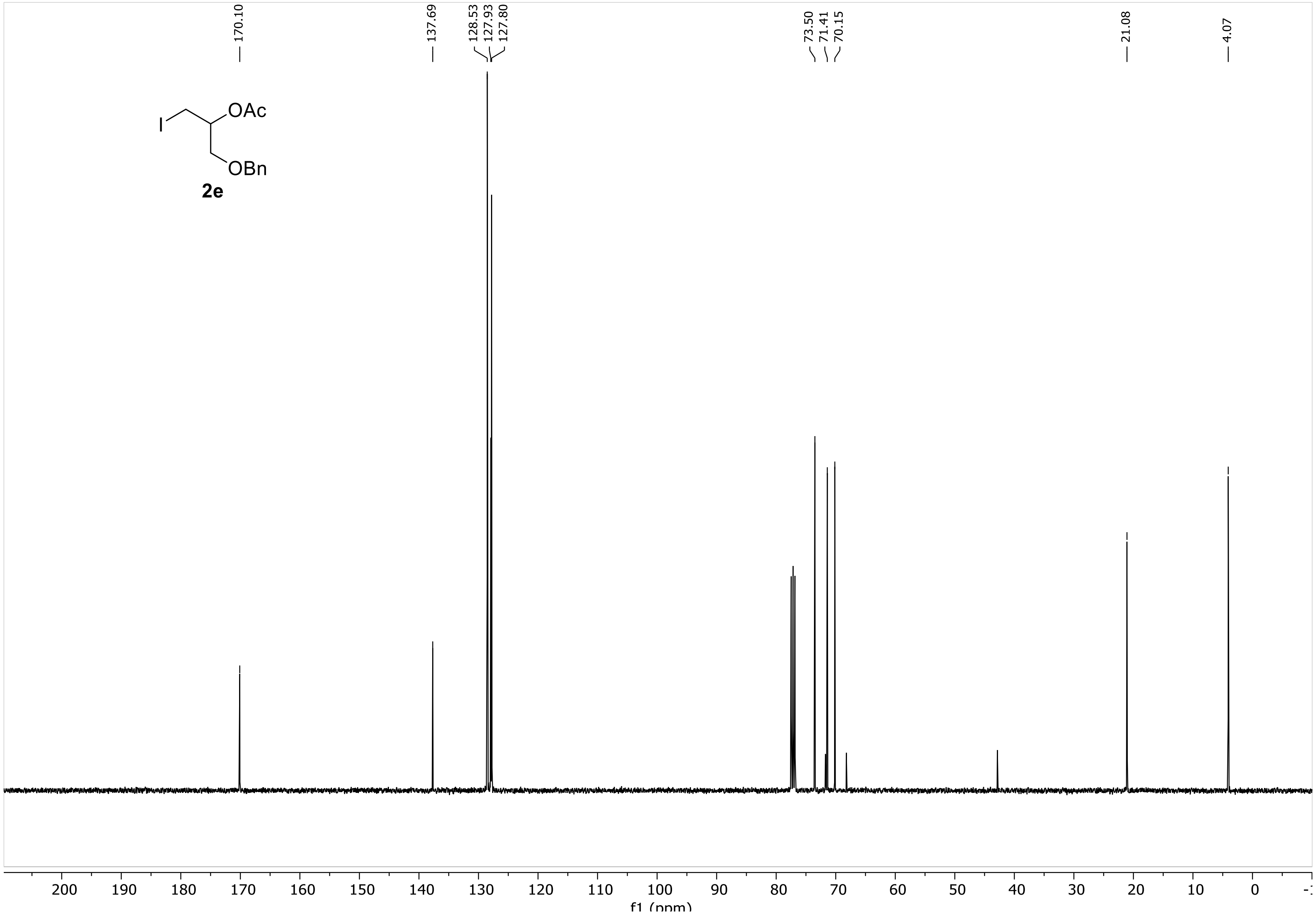

^{13}C NMR spectra (400 MHz, CDCl$_3$) of 1-(benzyloxy)-3-iodopropan-2-yl acetate (**2e**)

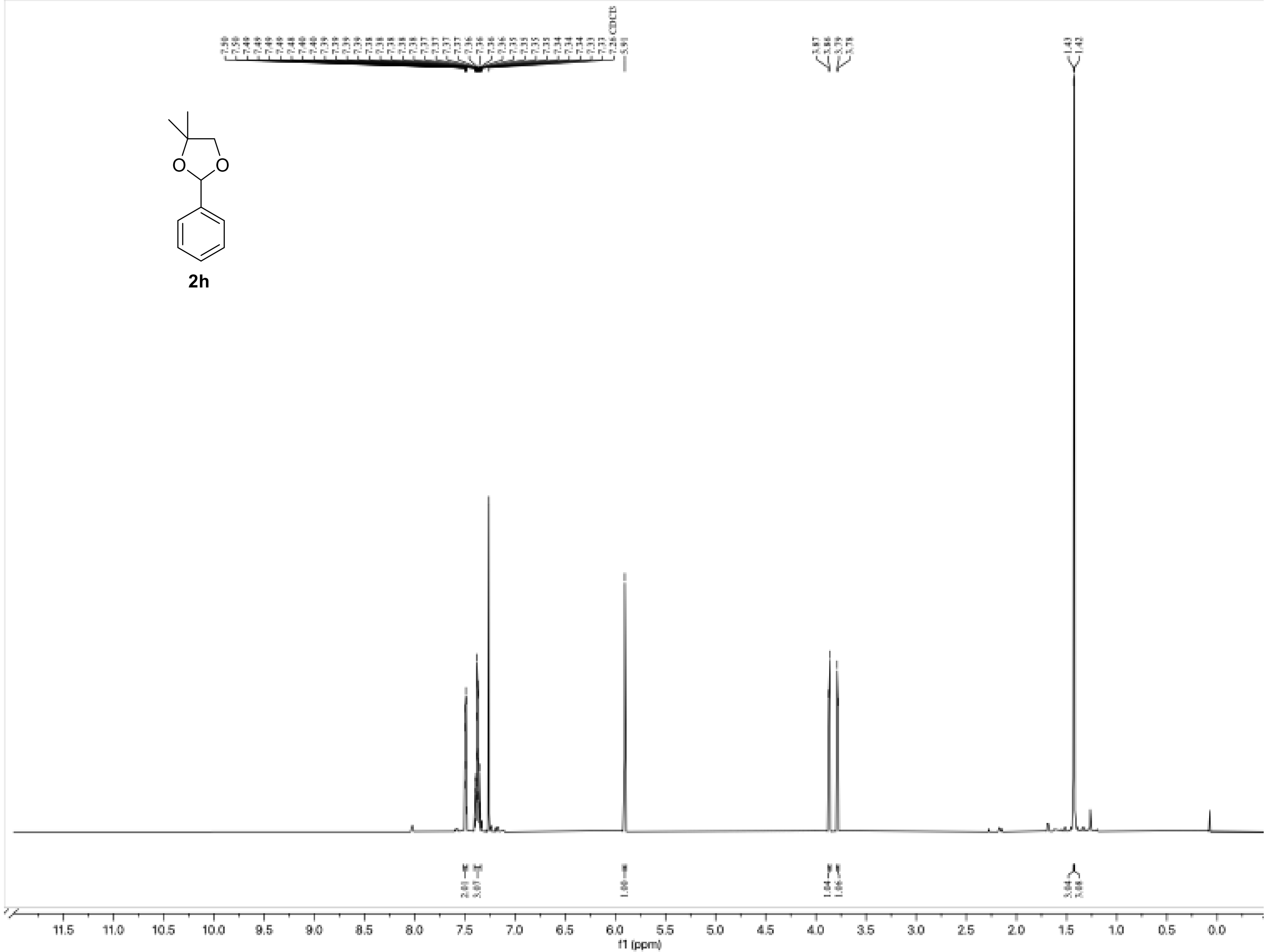

^{1}H NMR spectra (700 MHz, $CDCl_3$) of 4,4-dimethyl-2-phenyl-1,3-dioxolane (**2h**)

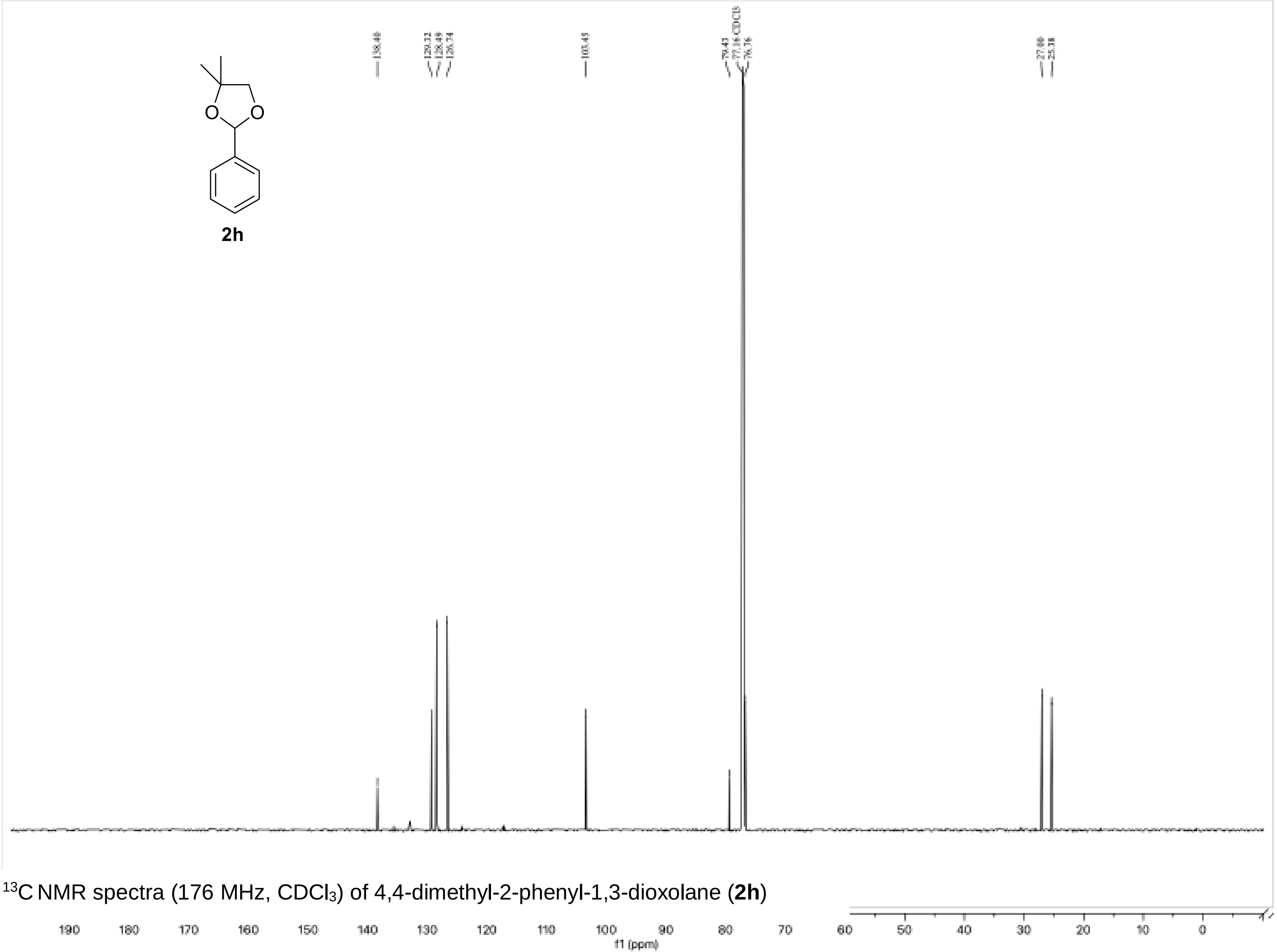

^{13}C NMR spectra (176 MHz, $CDCl_3$) of 4,4-dimethyl-2-phenyl-1,3-dioxolane (**2h**)

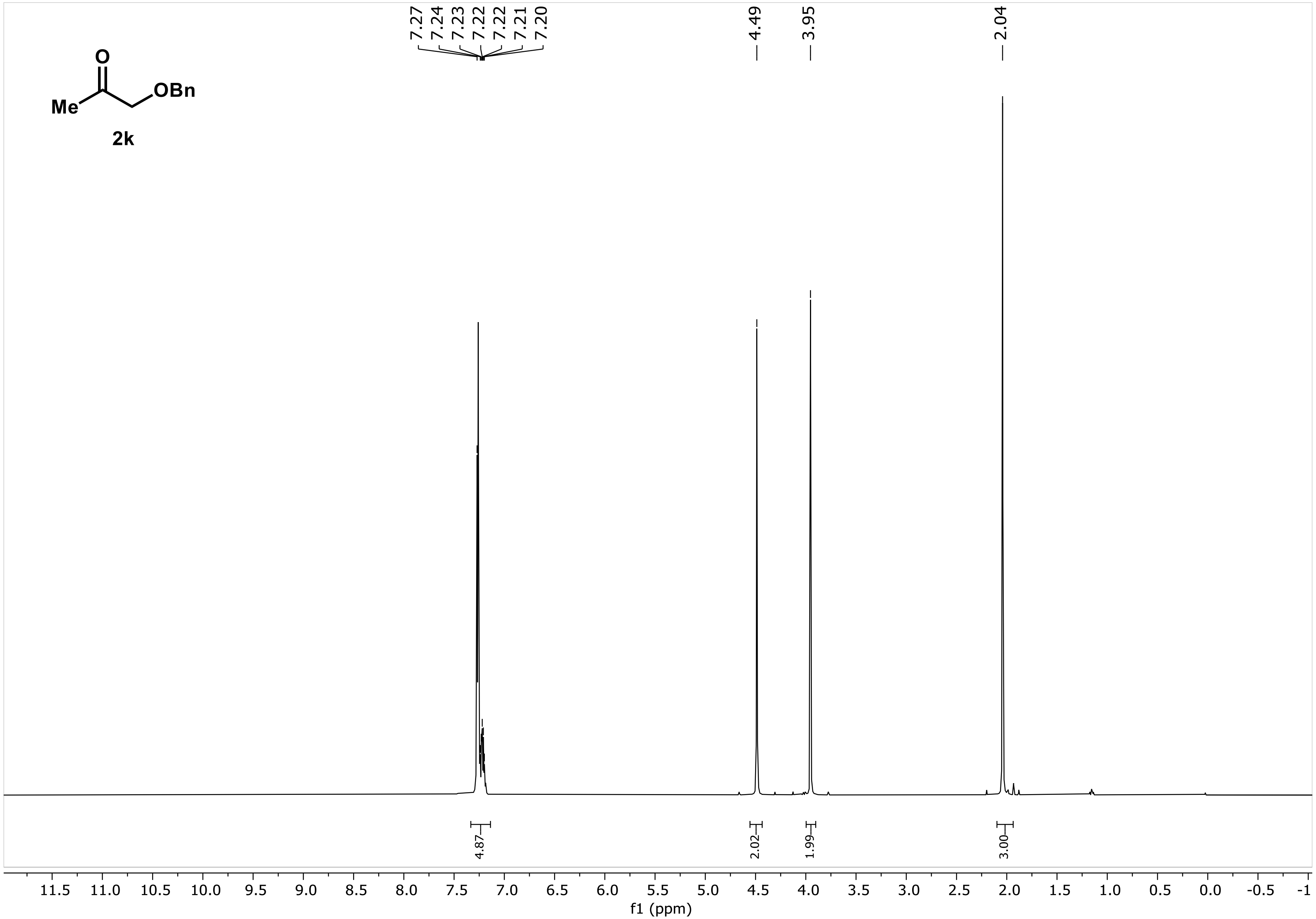

^{1}H NMR spectra (400 MHz, $CDCl_3$) of 1-(benzyloxy)propan-2-one (**2k**)

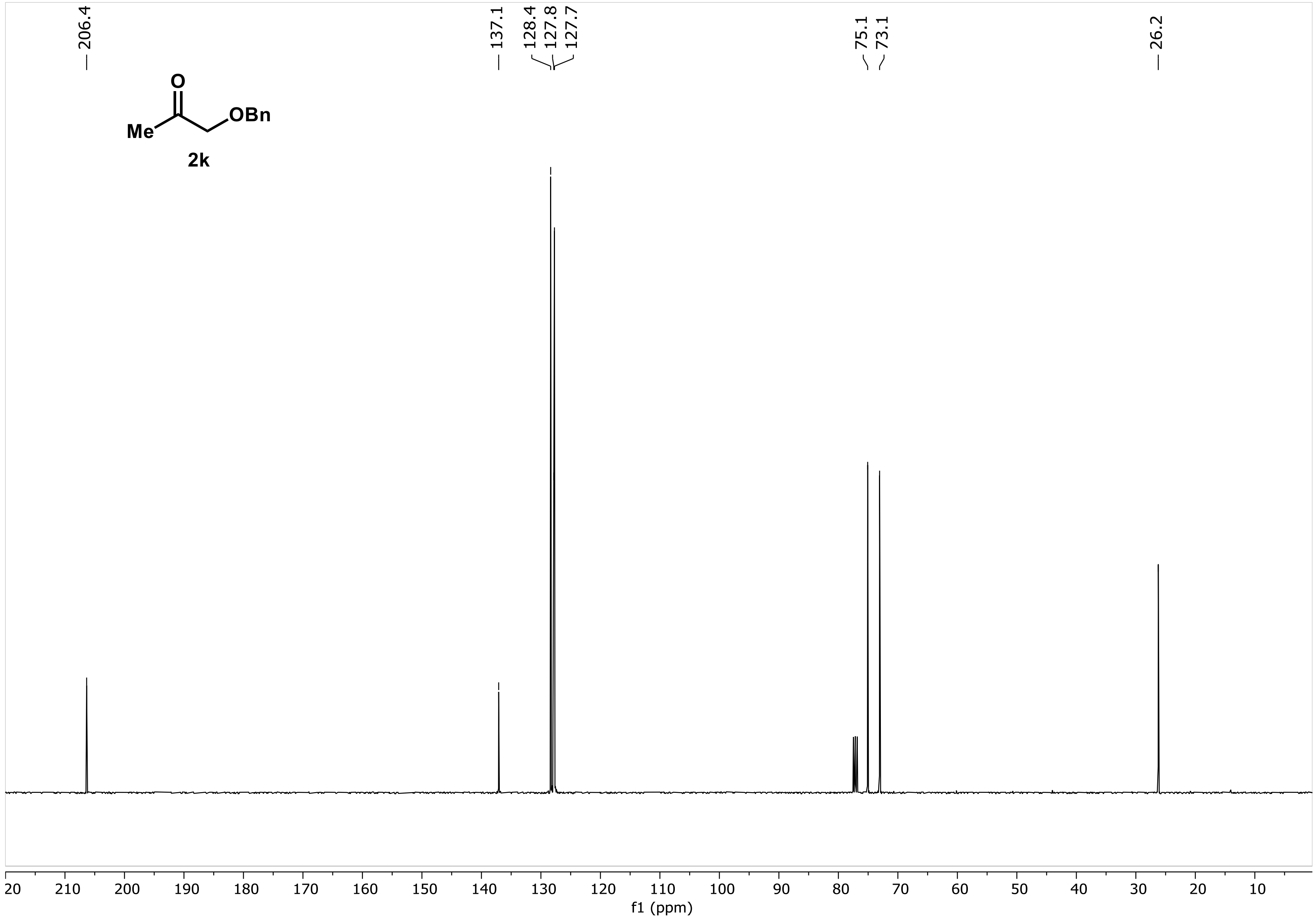

^{13}C NMR spectra (101 MHz, $CDCl_3$) of 1-(benzyloxy)propan-2-one (**2k**)

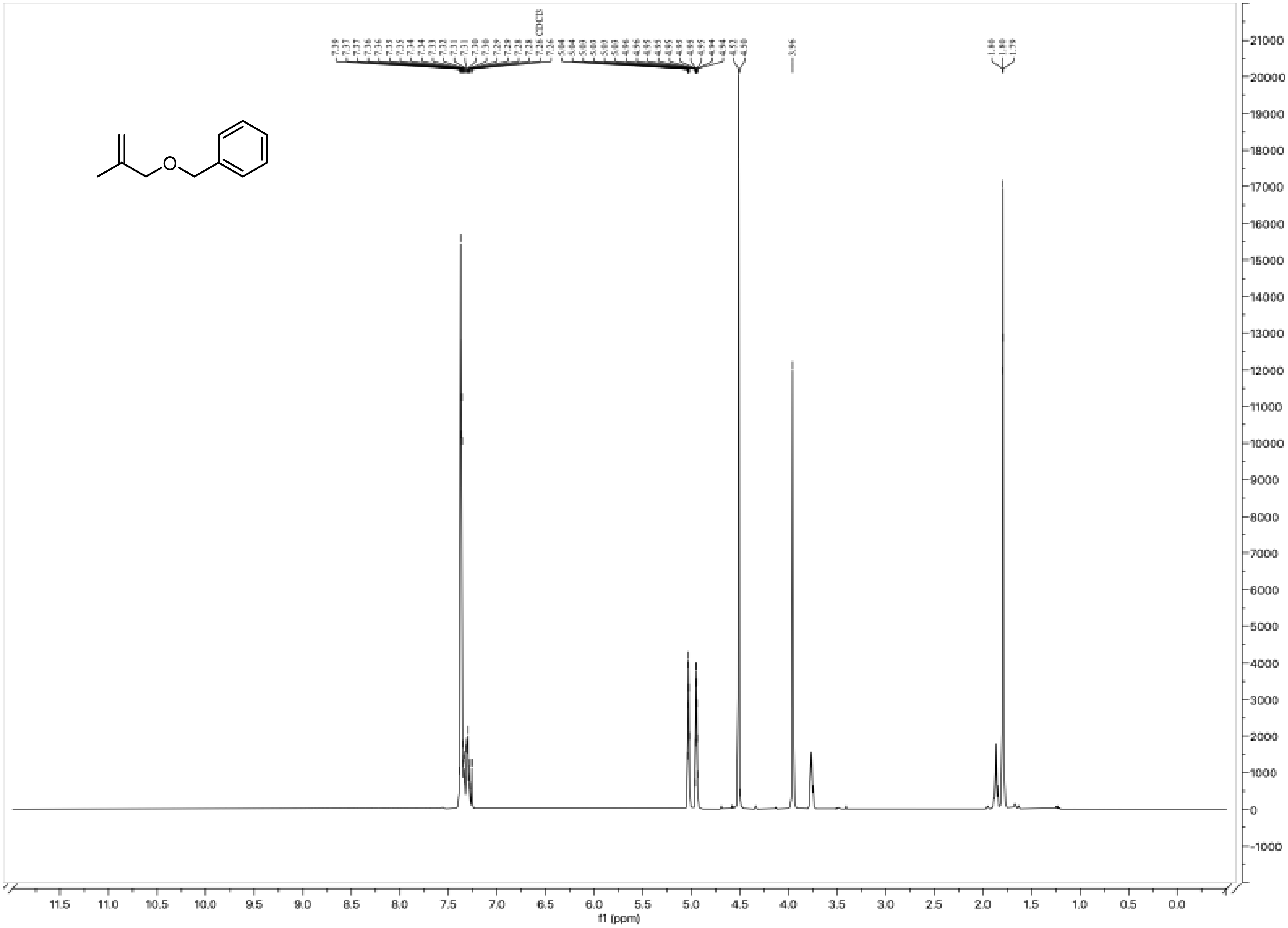

^{1}H NMR spectra (400 MHz, $CDCl_3$) of (((2-Methylallyl)oxy)methyl)benzene

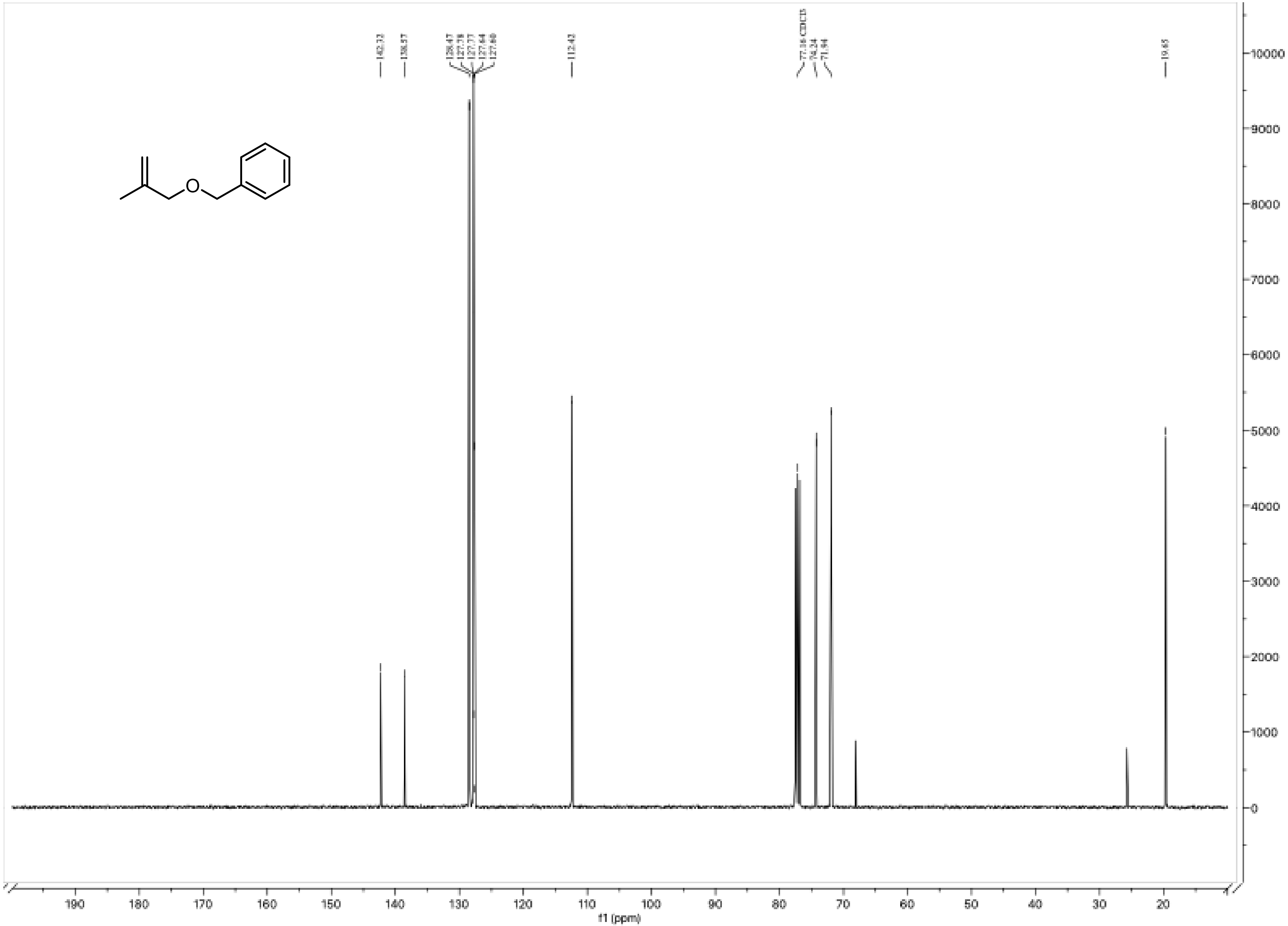

^{13}C NMR spectra (101 MHz, $CDCl_3$) of (((2-Methylallyl)oxy)methyl)benzene

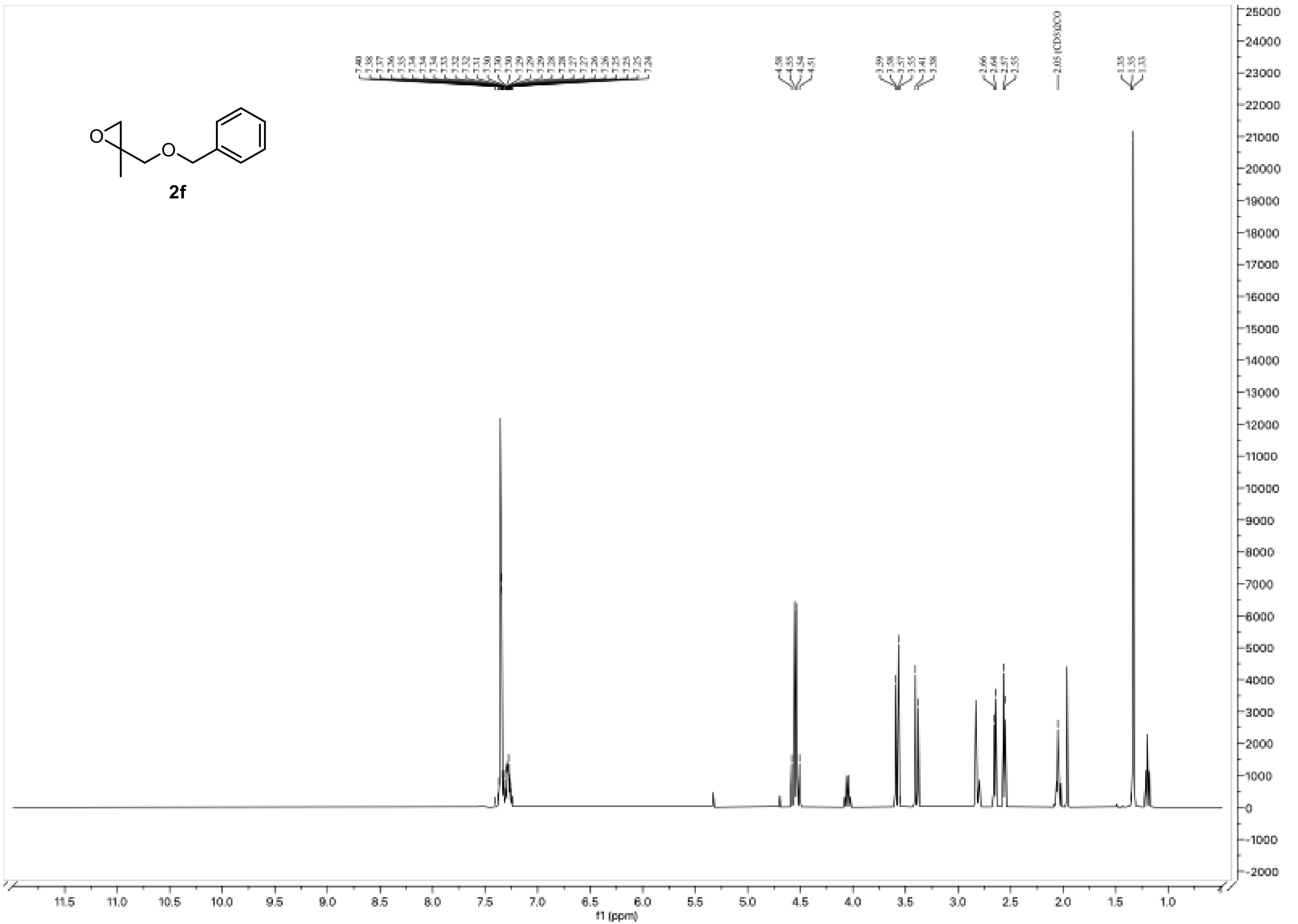

^{1}H NMR spectra (400 MHz, $CO(CD_3)$) of 2-((Benzyloxy)methyl)-2-methyloxirane (**2f**)

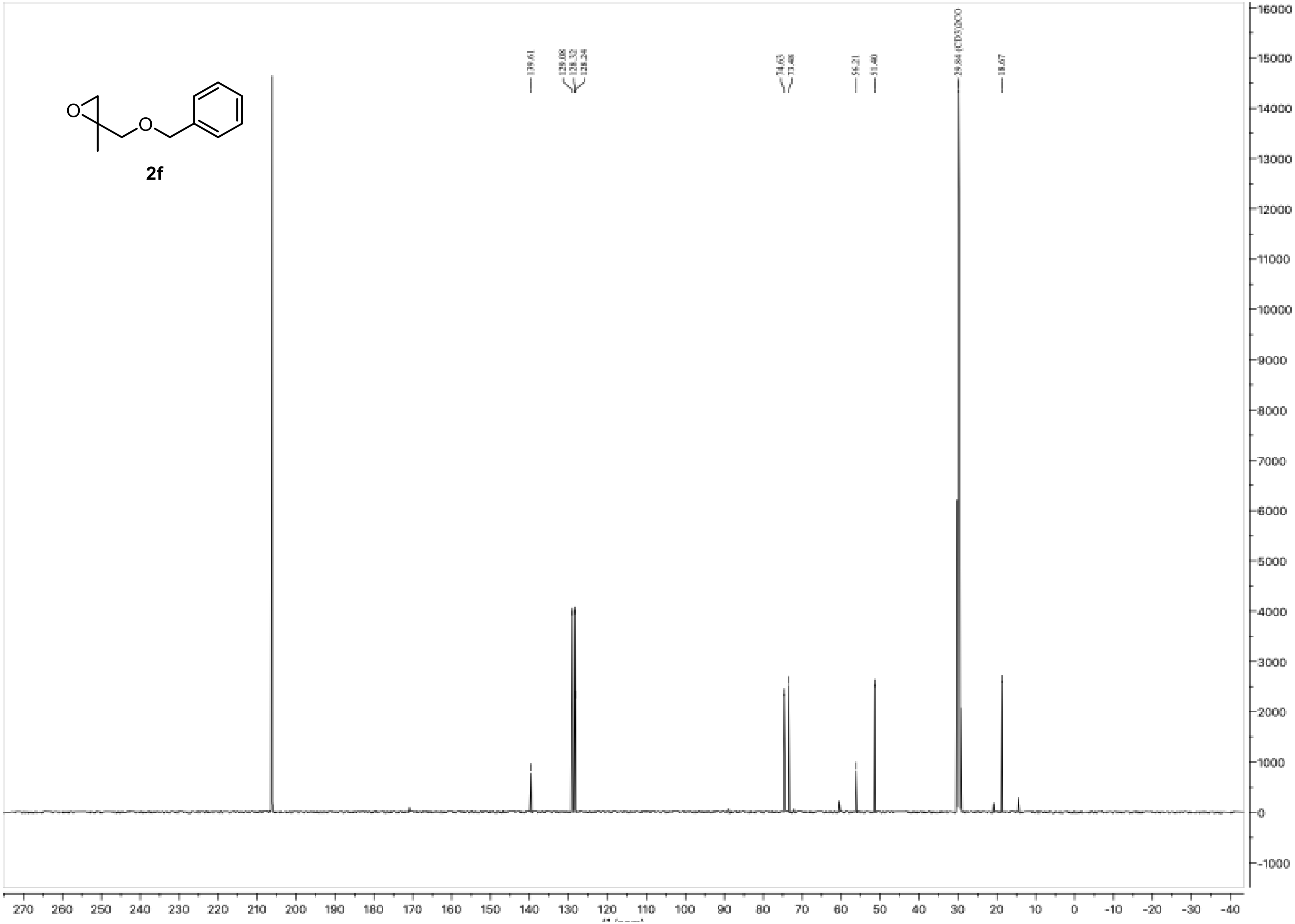

^{13}C NMR spectra (101 MHz, $CO(CD_3)_2$) of 2-((Benzyloxy)methyl)-2-methyloxirane (**2f**)

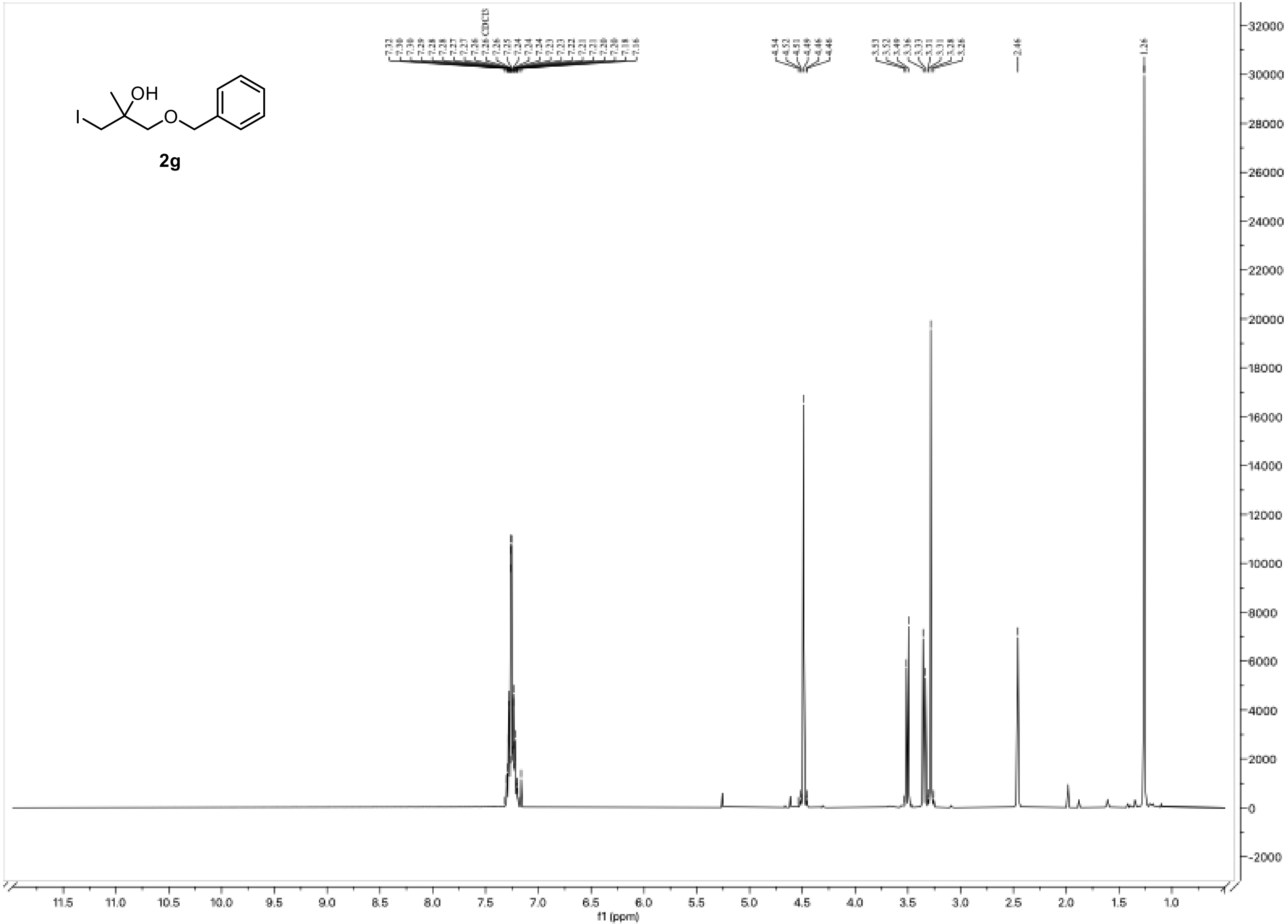

^{1}H NMR spectra (400 MHz, $CDCl_3$) of 1-(Benzyloxy)-3-iodo-2-methylpropan-2-ol (**2g**)

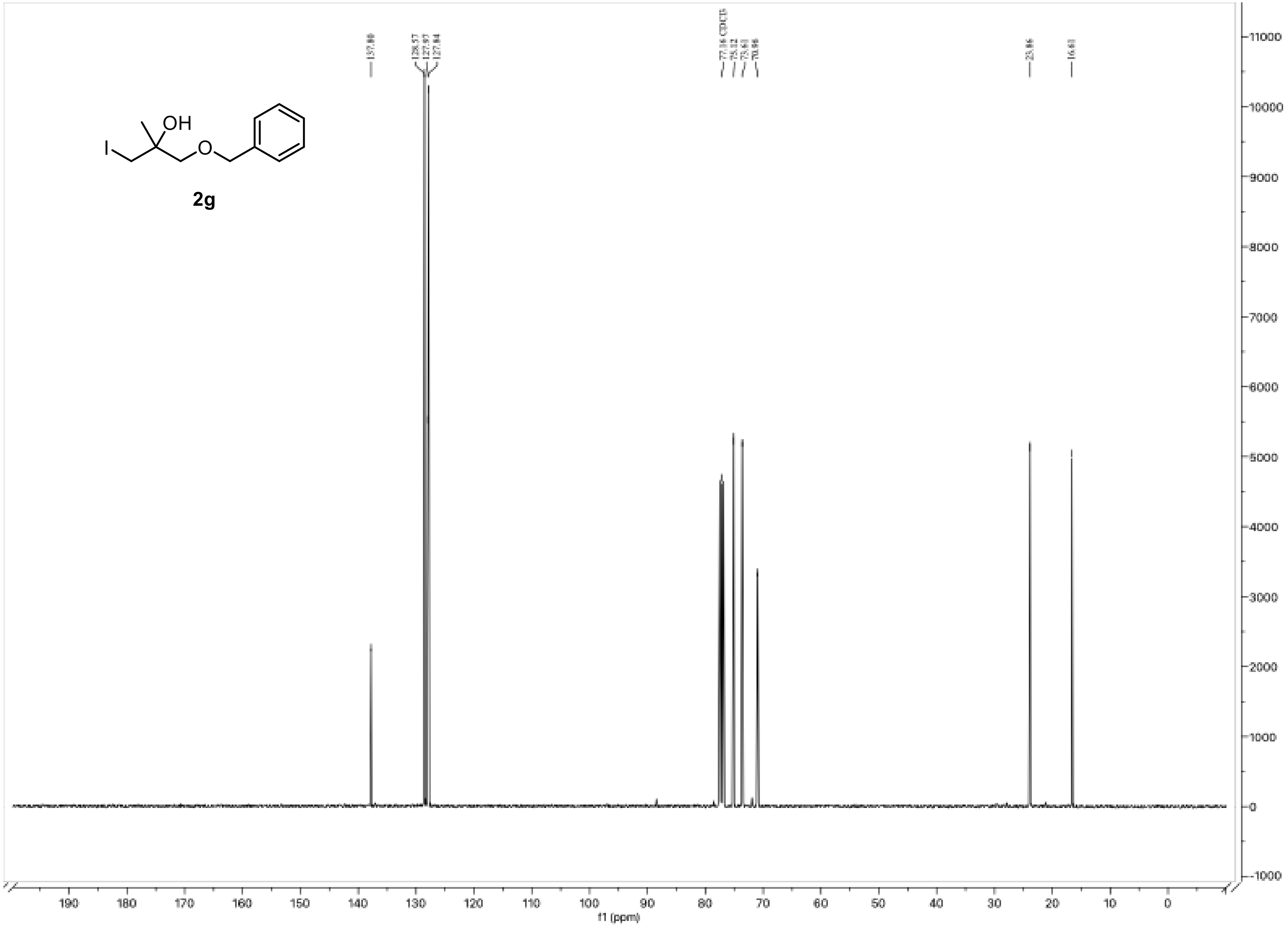

^{13}C NMR spectra (101 MHz, $CDCl_3$) of 1-(Benzyloxy)-3-iodo-2-methylpropan-2-ol (**2g**)

9.5. Alkylated Products

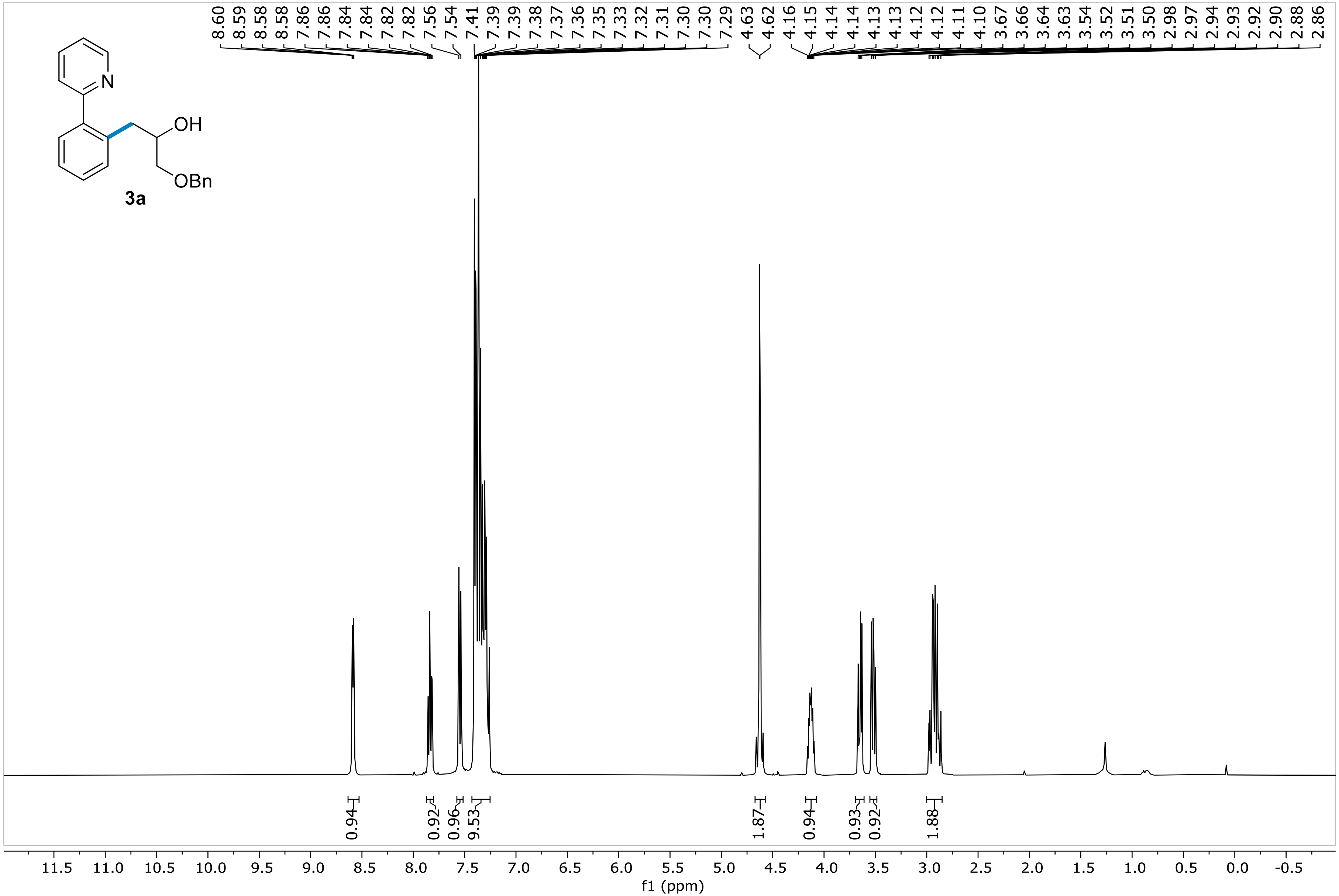

^{1}H NMR spectra (400 MHz, $CDCl_3$) of 1-(benzyloxy)-3-(2-(pyridin-2-yl)phenyl)propan-2-ol (**3a**)

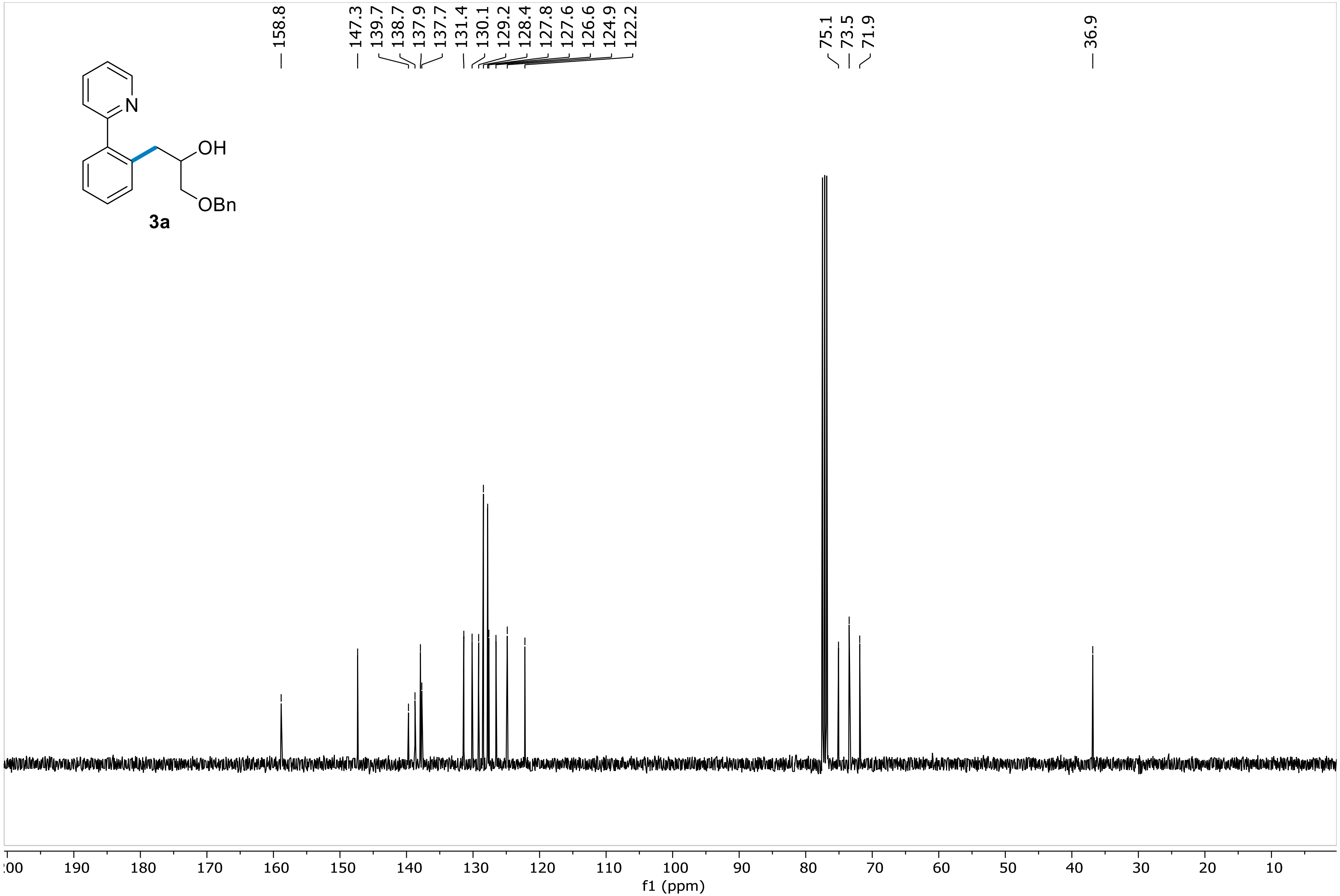

^{13}C NMR spectra (101 MHz, $CDCl_3$) of 1-(benzyloxy)-3-(2-(pyridin-2-yl)phenyl)propan-2-ol (**3a**)

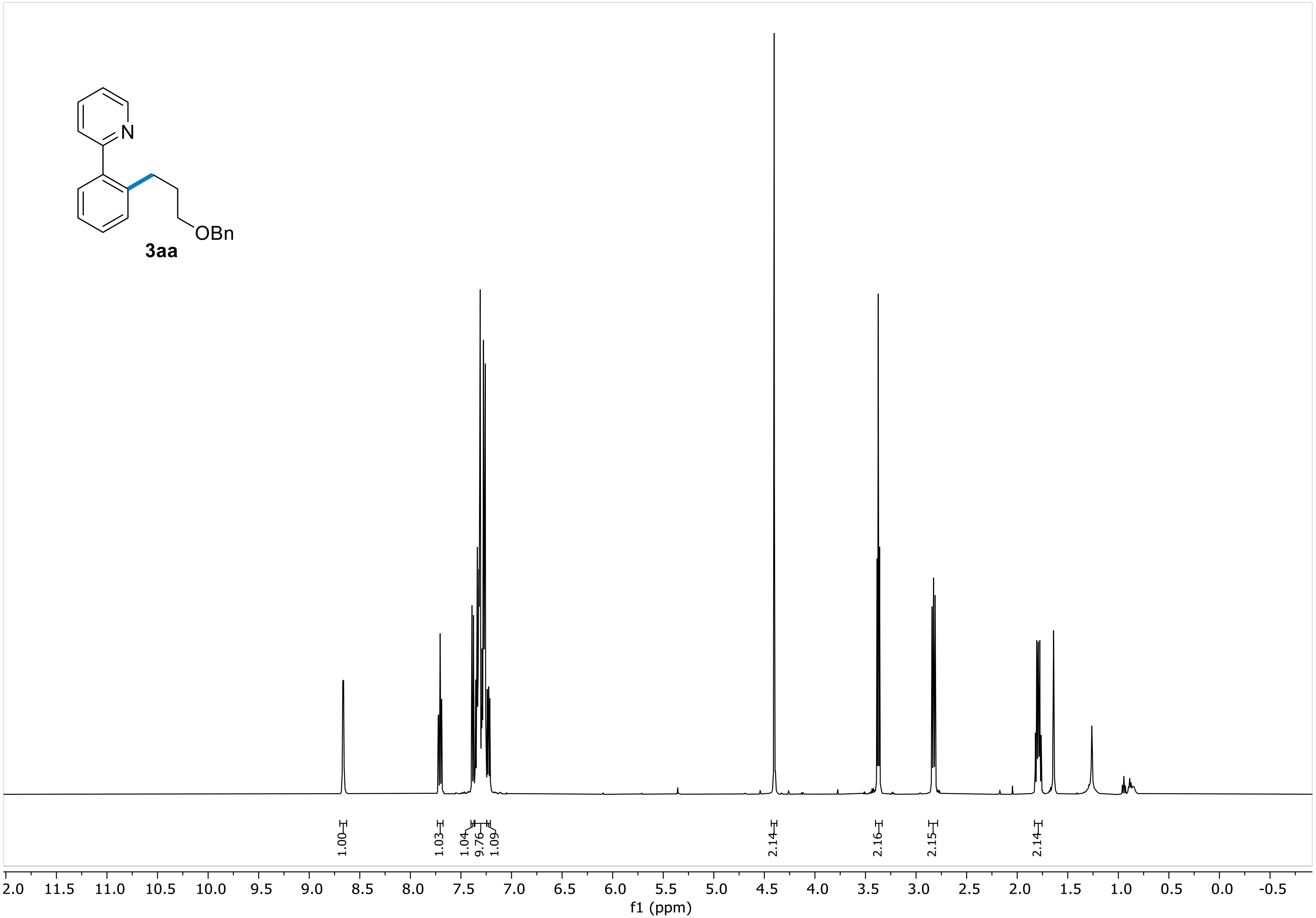

^{1}H NMR spectra (400 MHz, $CDCl_3$) of 1-(benzyloxy)-3-(2-(pyridin-2-yl)phenyl)propane (**3aa**)

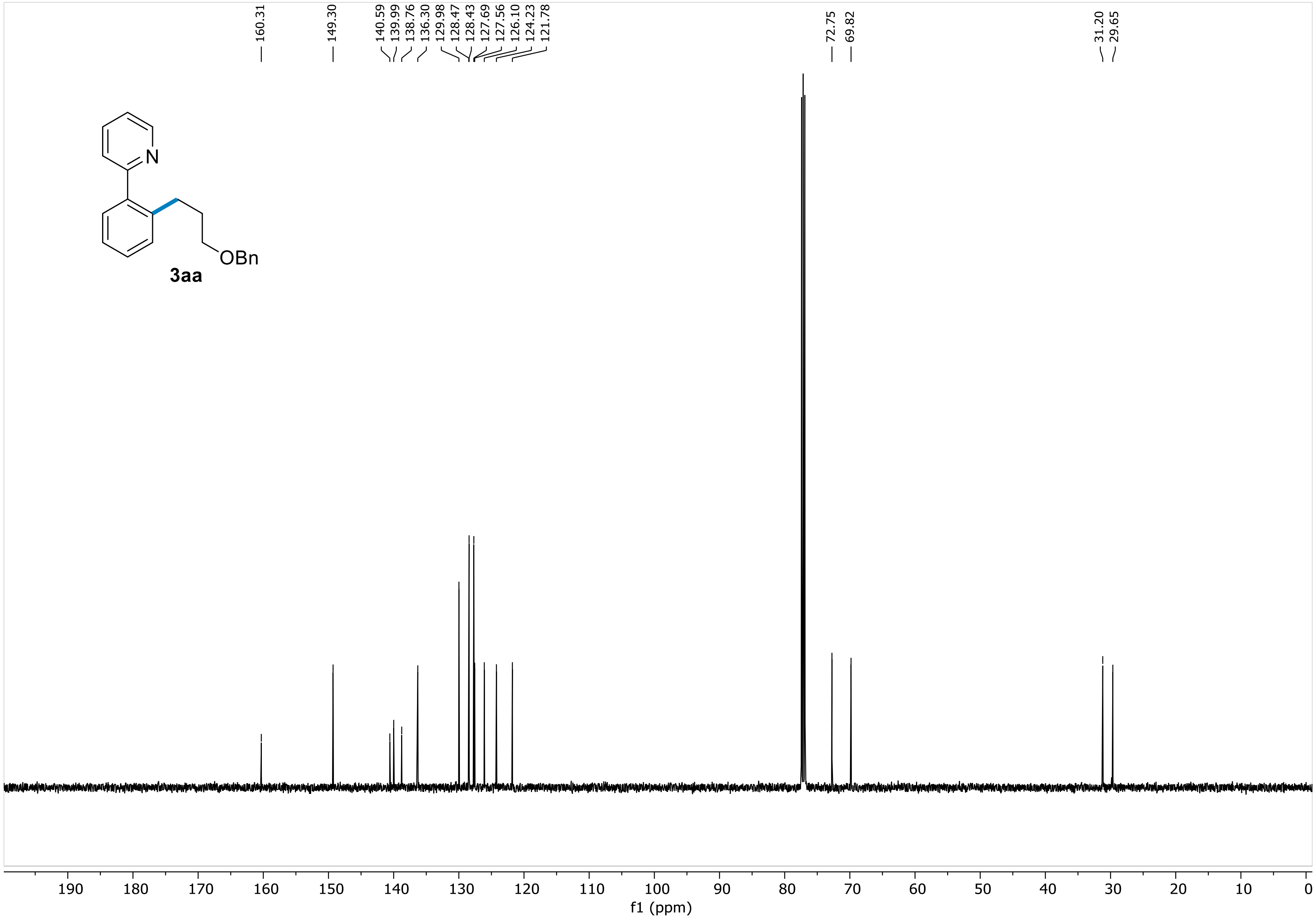

^{13}C NMR spectra (101 MHz, $CDCl_3$) of 1-(benzyloxy)-3-(2-(pyridin-2-yl)phenyl)propane (**3aa**)

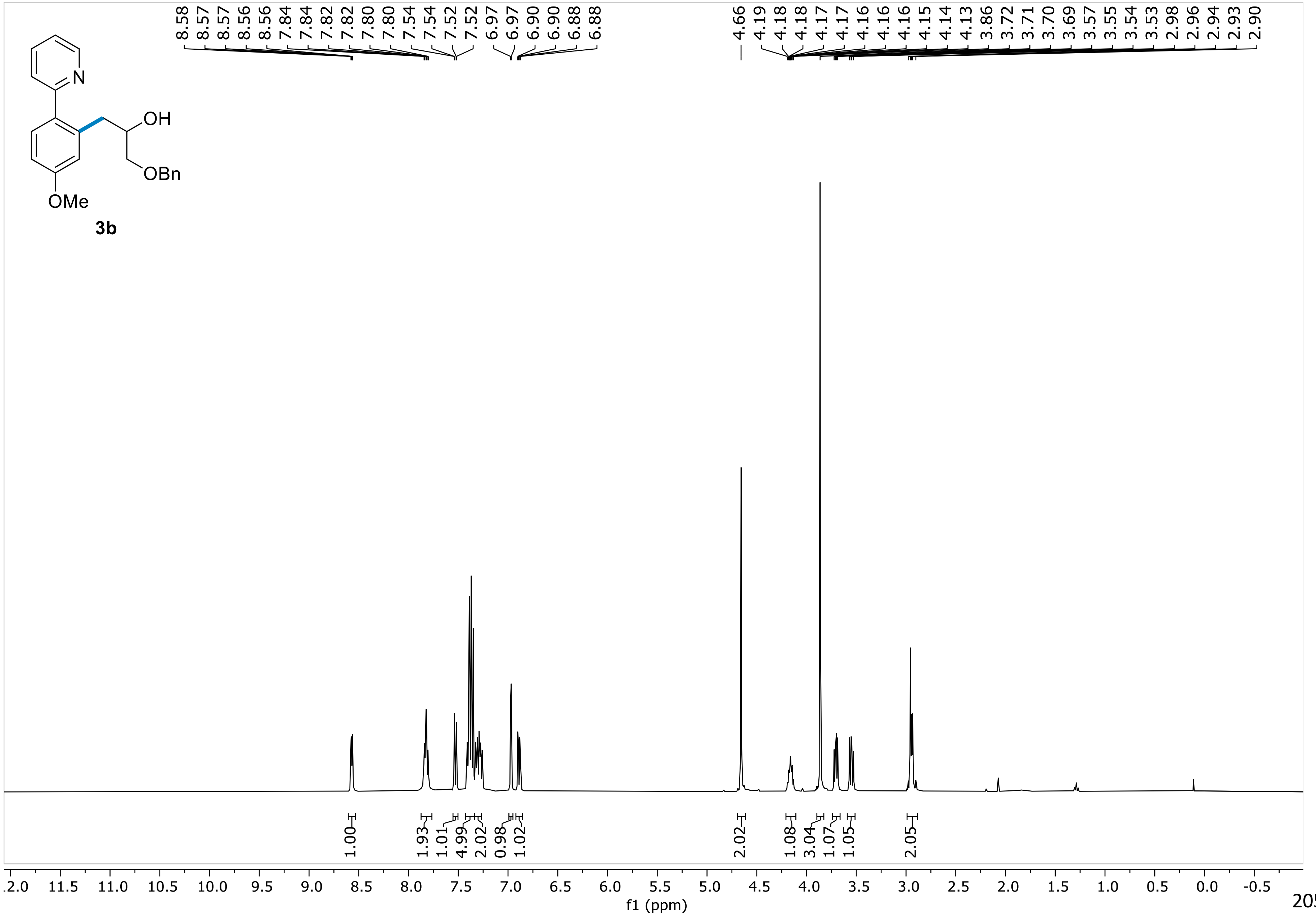

^{1}H NMR spectra (400 MHz, CDCl$_3$) of 1-(benzyloxy)-3-(5-methoxy-2-(pyridin-2-yl)phenyl)propan-2-ol (**3b**)

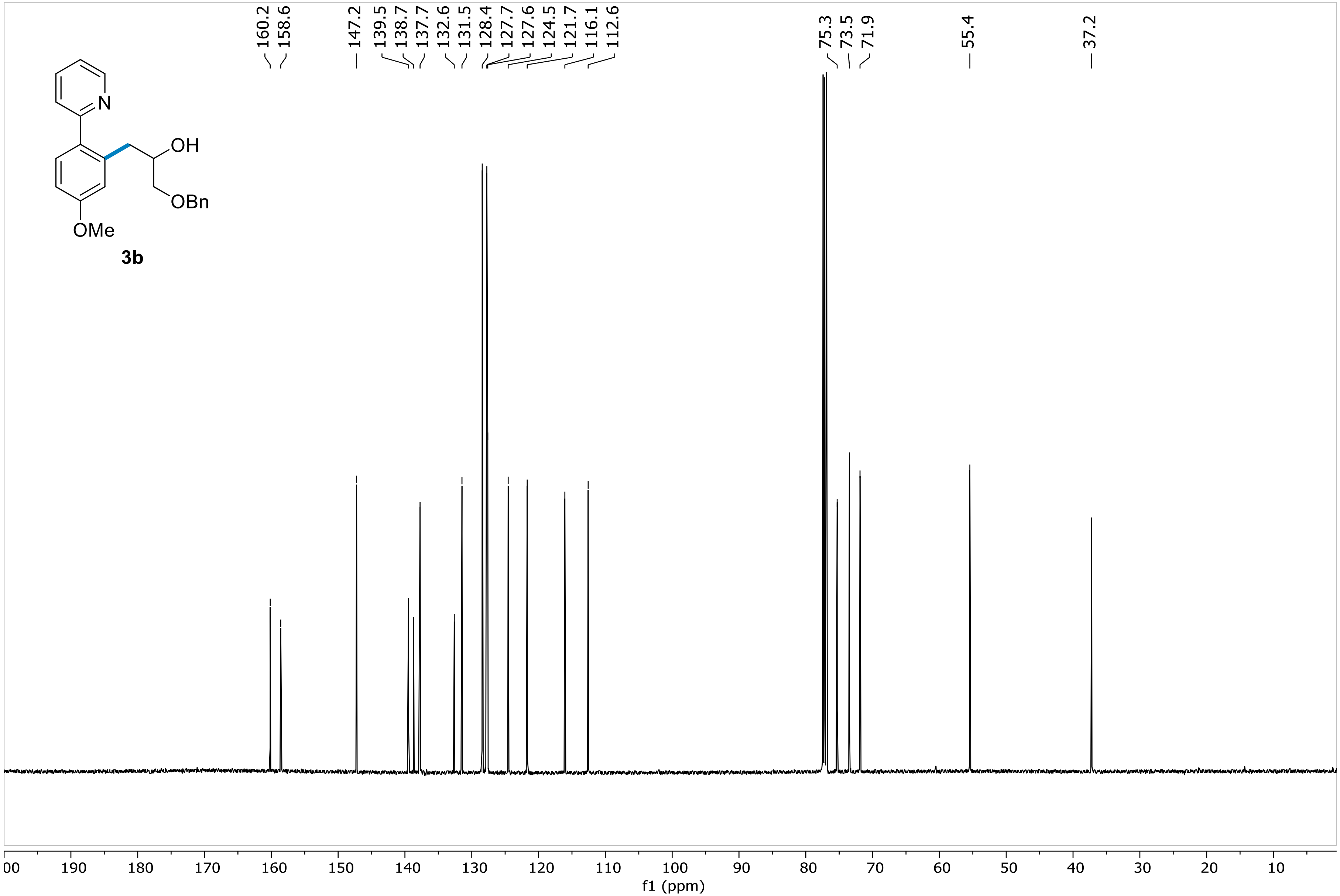

^{13}C NMR spectra (126 MHz, $CDCl_3$) of 1-(benzyloxy)-3-(5-methoxy-2-(pyridin-2-yl)phenyl)propan-2-ol (**3b**)

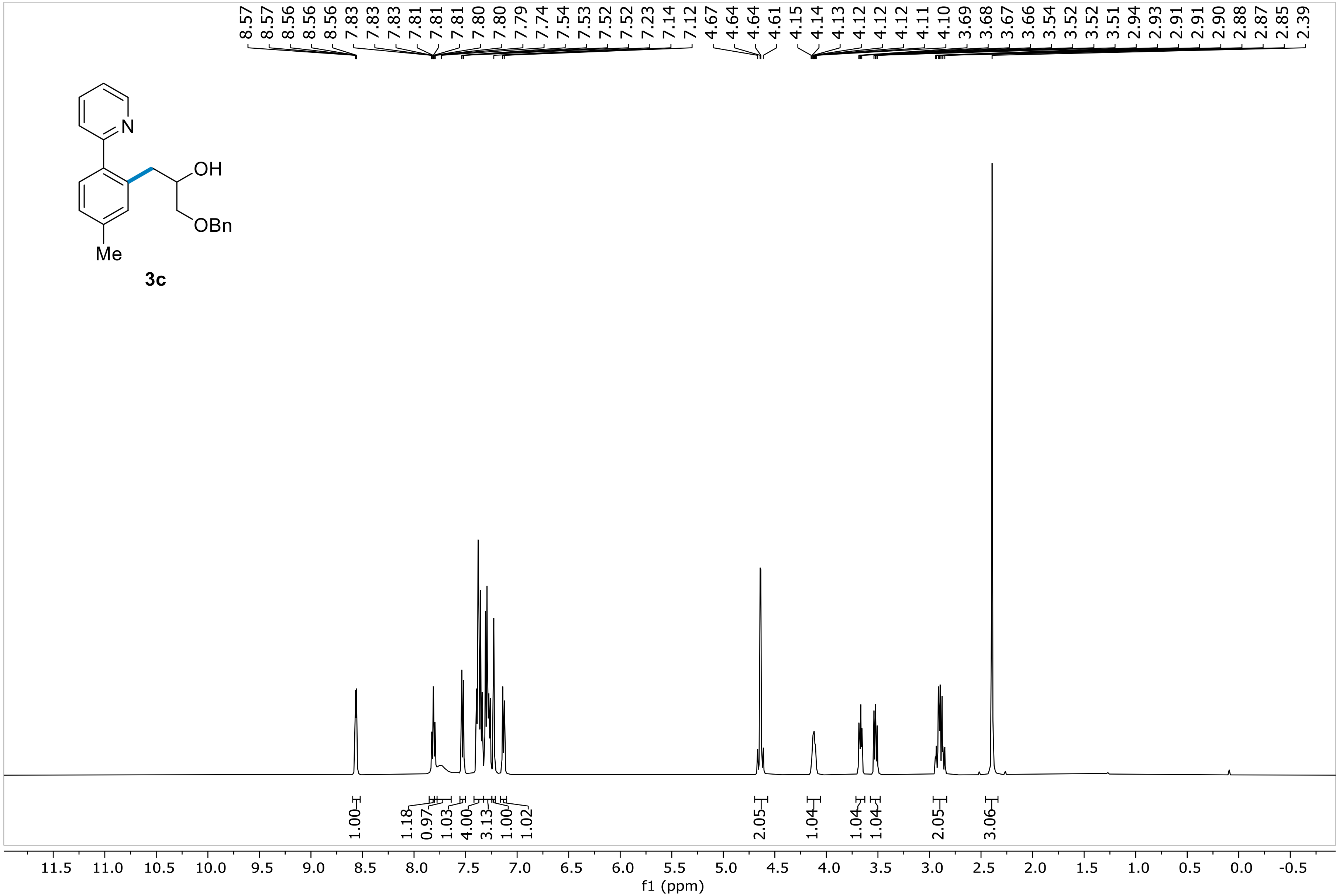

^{1}H NMR spectra (500 MHz, $CDCl_3$) of 1-(benzyloxy)-3-(5-methyl-2-(pyridin-2-yl)phenyl)propan-2-ol (**3c**)

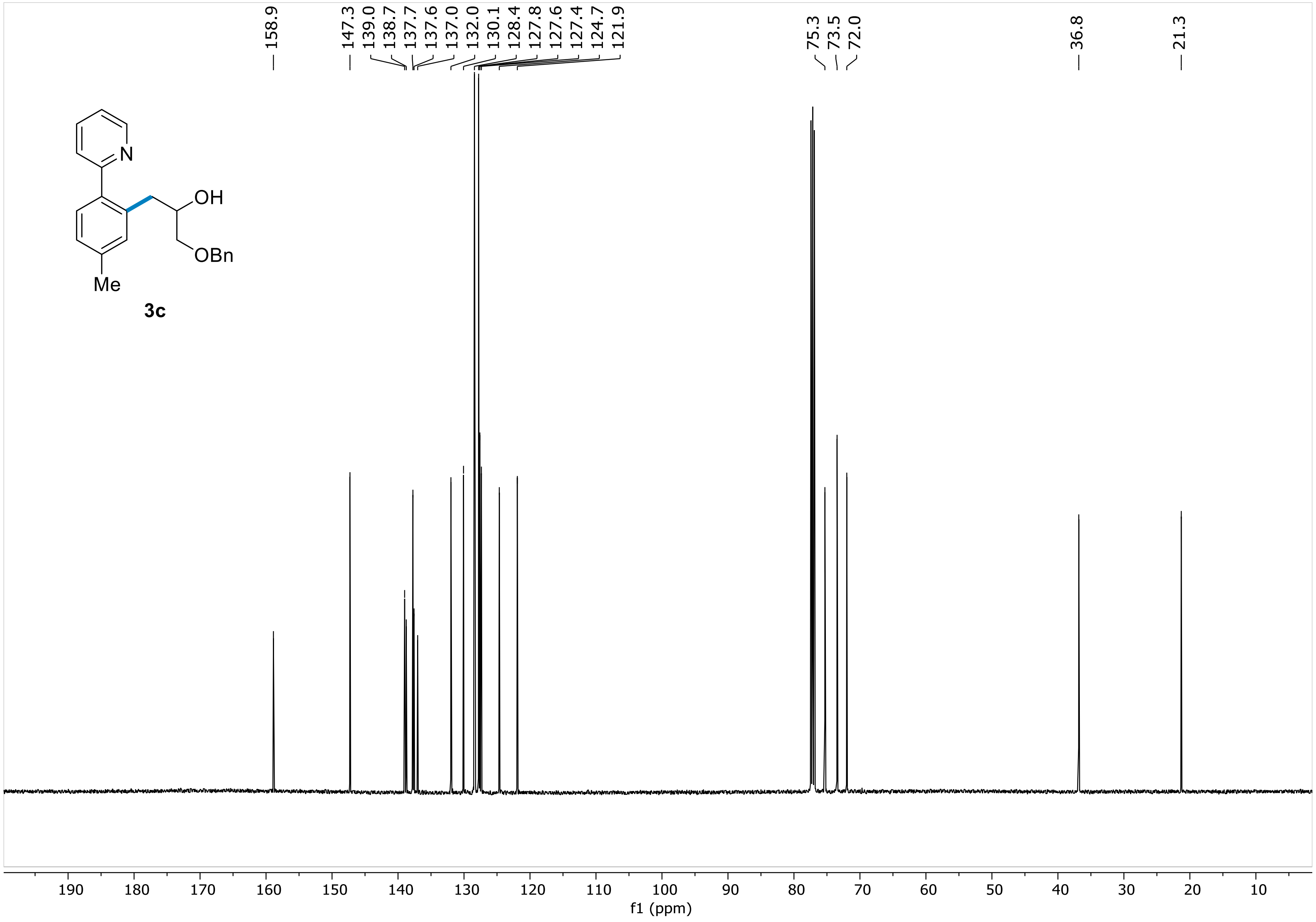

^{13}C NMR spectra (126 MHz, $CDCl_3$) of 1-(benzyloxy)-3-(5-methyl-2-(pyridin-2-yl)phenyl)propan-2-ol (**3c**)

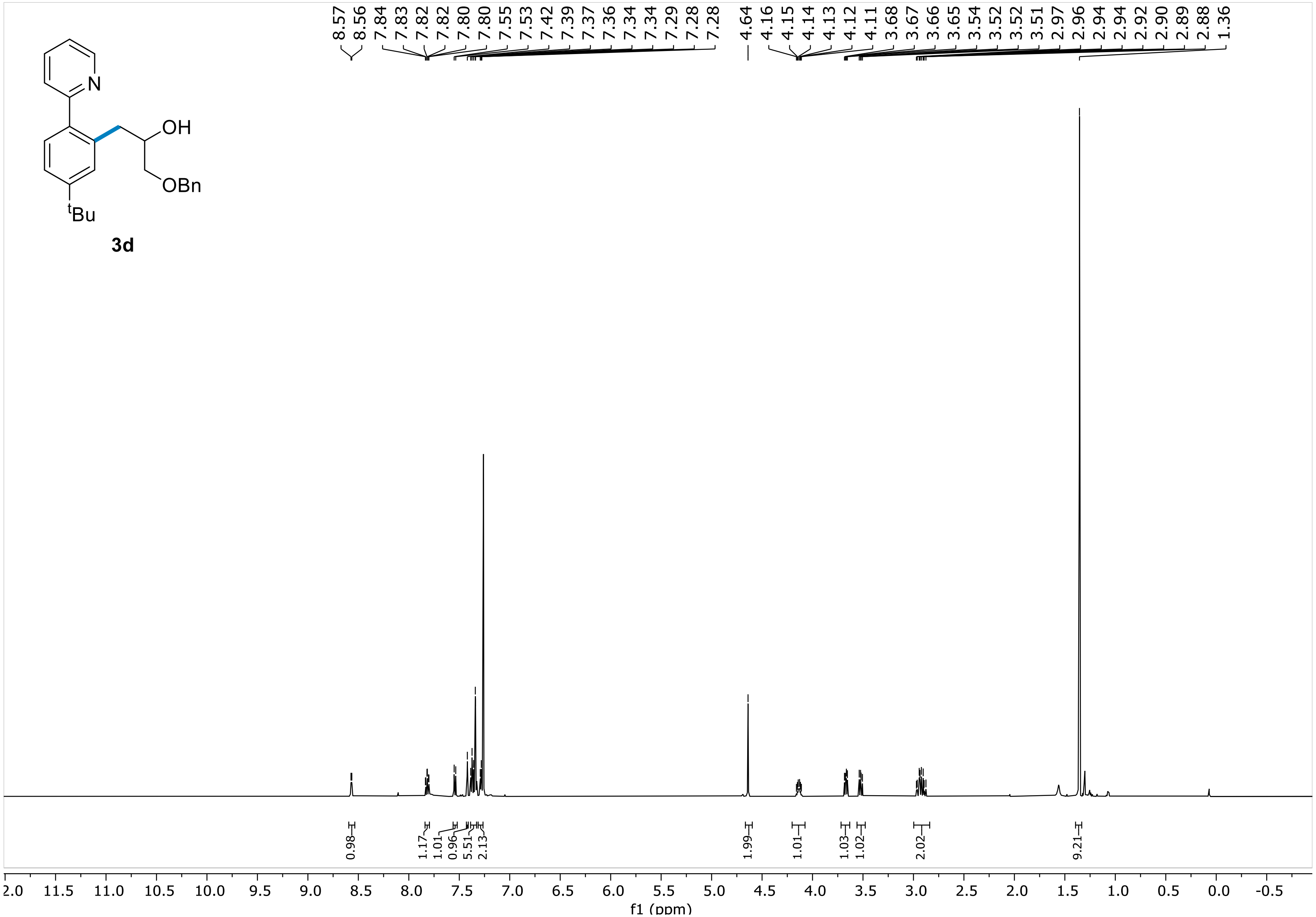

^{1}H NMR spectra (500 MHz, $CDCl_3$) of 1-(benzyloxy)-3-(5-(tert-butyl)-2-(pyridin-2-yl)phenyl)propan-2-ol (**3d**)

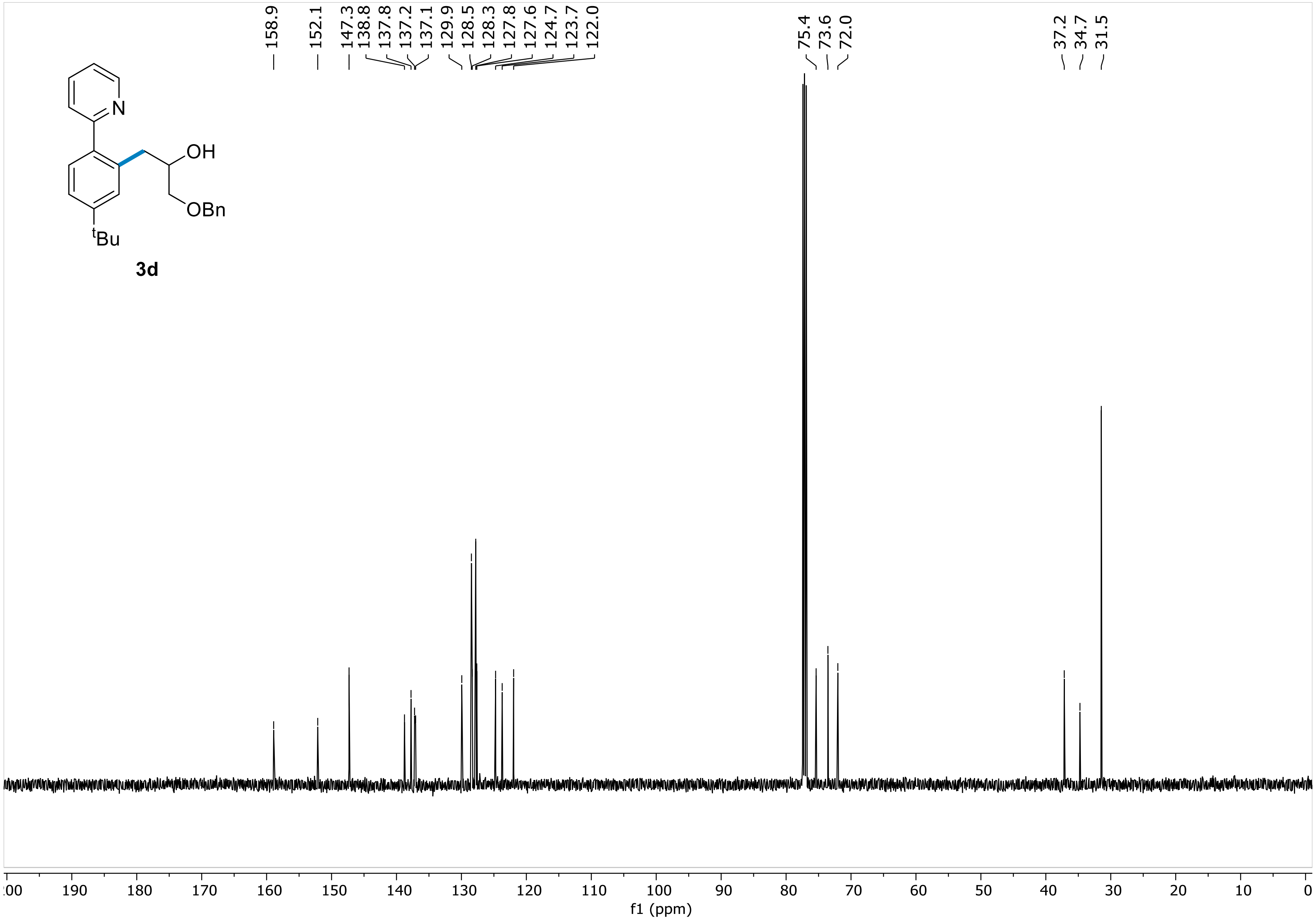

^{13}C NMR spectra (126 MHz, $CDCl_3$) of 1-(benzyloxy)-3-(5-(tert-butyl)-2-(pyridin-2-yl)phenyl)propan-2-ol (**3d**)

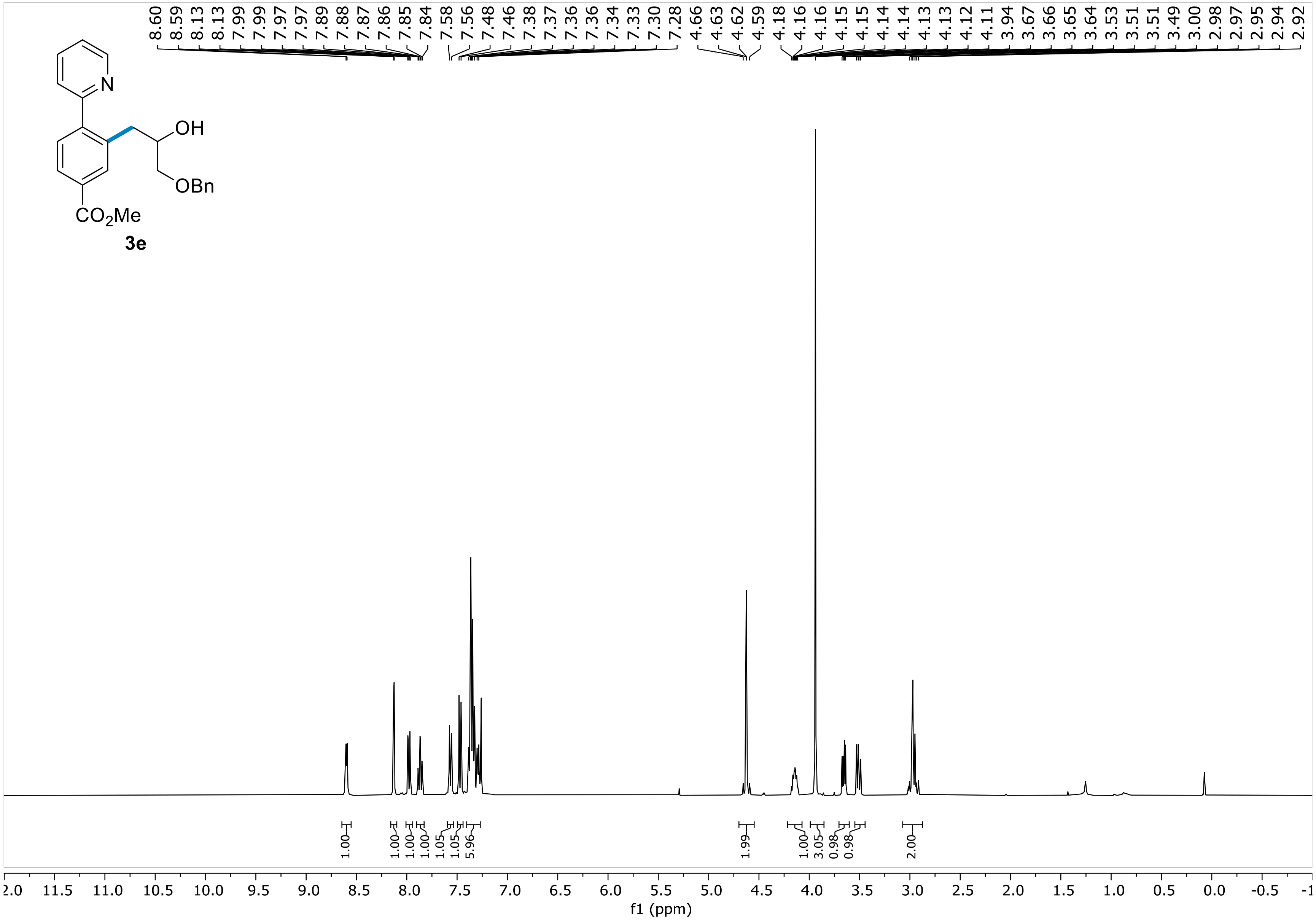

^{1}H NMR spectra (400 MHz, $CDCl_3$) of methyl 3-(3-(benzyloxy)-2-hydroxypropyl)-4-(pyridin-2-yl)benzoate (**3e**)

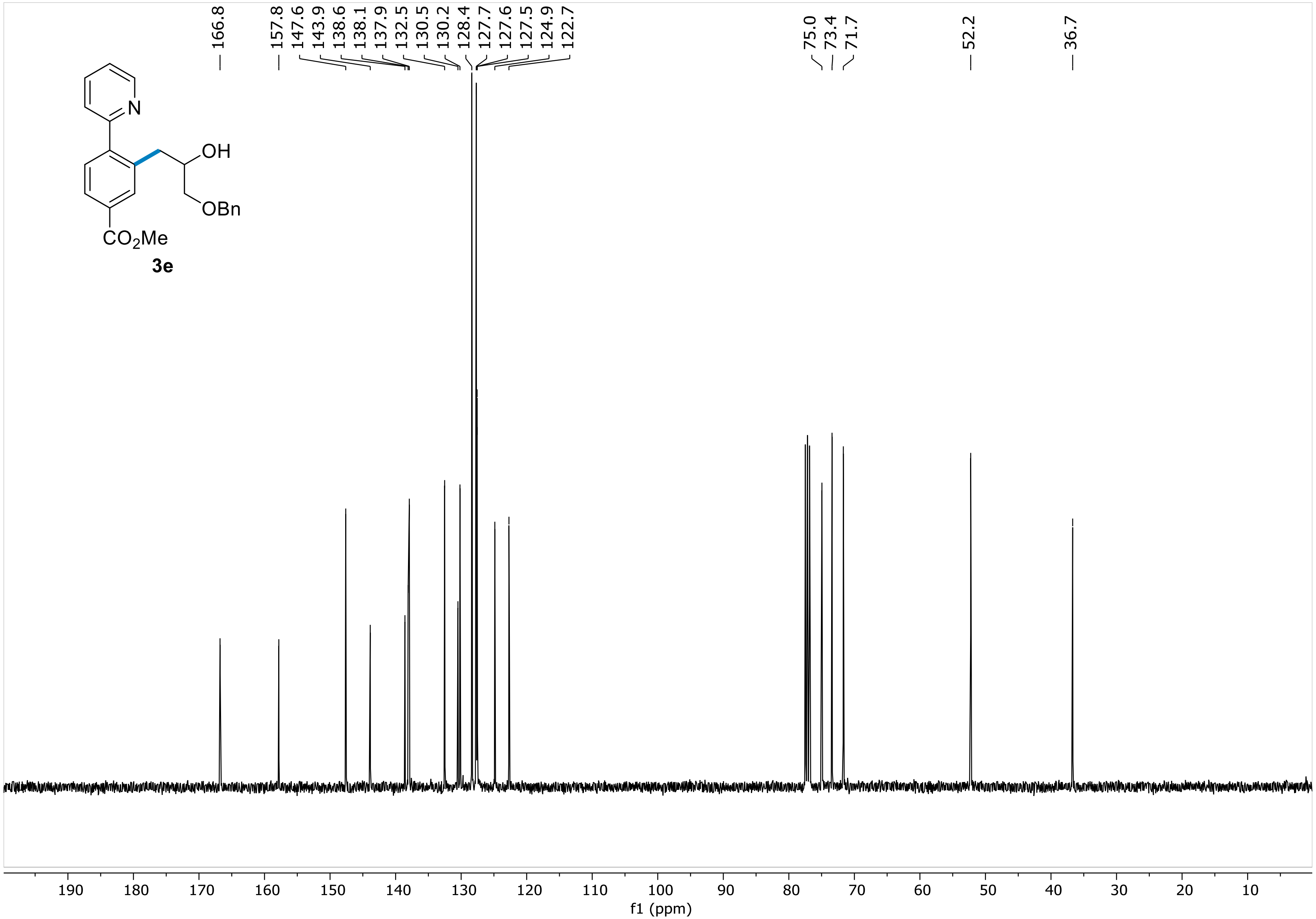

^{13}C NMR spectra (101 MHz, $CDCl_3$) of methyl 3-(3-(benzyloxy)-2-hydroxypropyl)-4-(pyridin-2-yl)benzoate (**3e**)

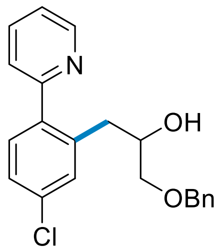

3f

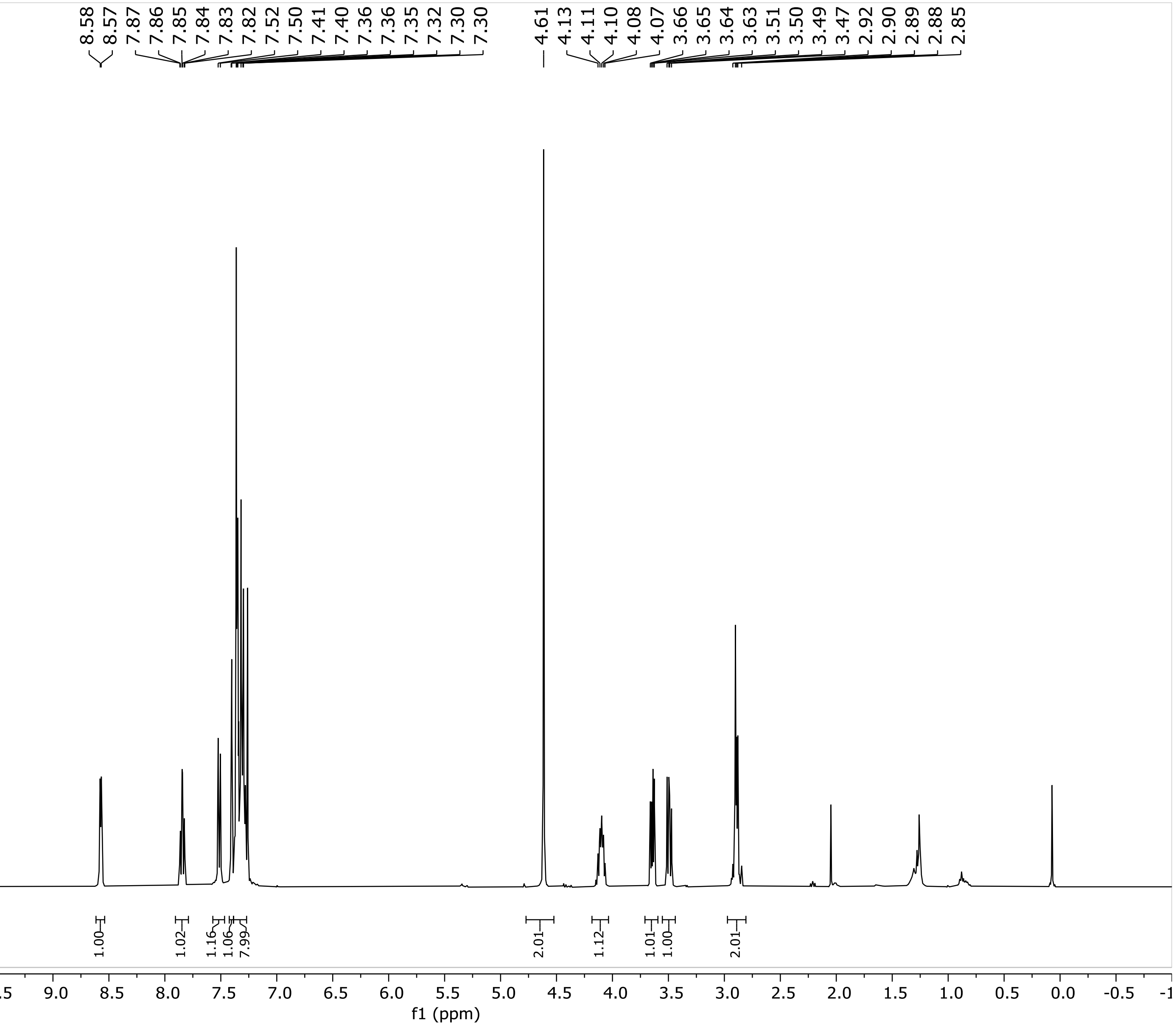

^{1}H NMR spectra (400 MHz, $CDCl_3$) of 1-(benzyloxy)-3-(5-chloro-2-(pyridin-2-yl)phenyl)propan-2-ol (**3f**)

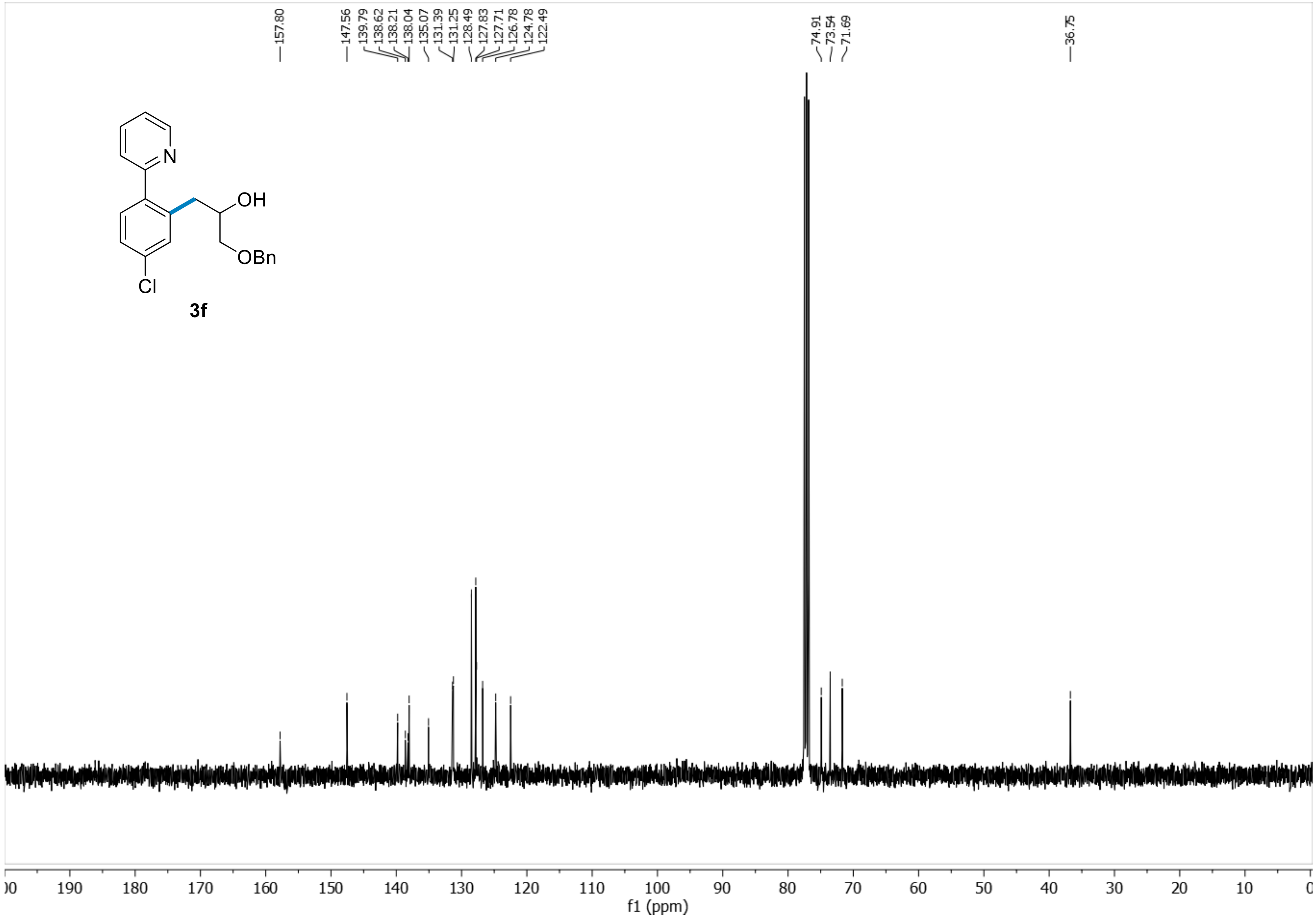

^{13}C NMR spectra (126 MHz, $CDCl_3$) of 1-(benzyloxy)-3-(5-chloro-2-(pyridin-2-yl)phenyl)propan-2-ol (**3f**)

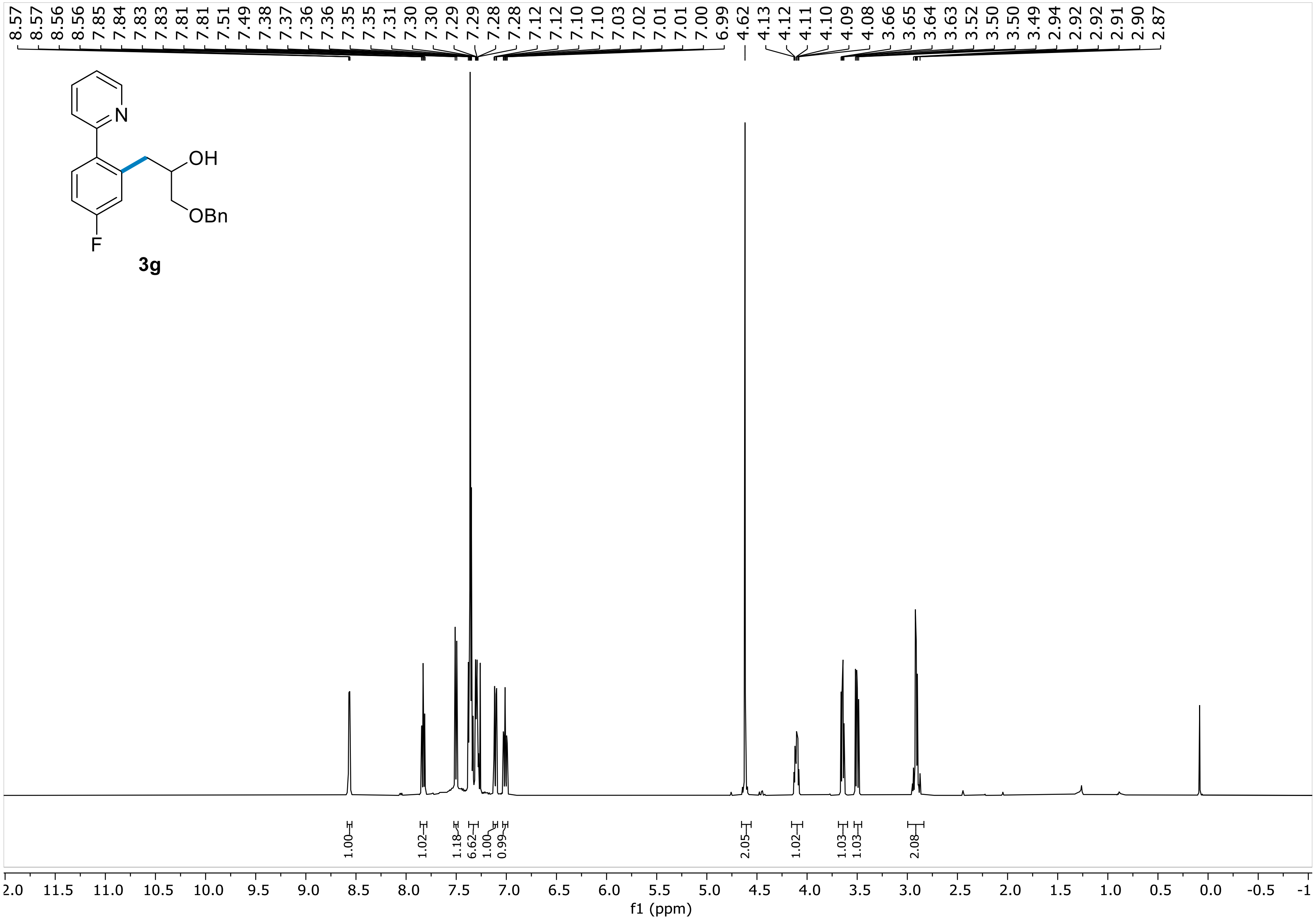

^{1}H NMR spectra (500 MHz, $CDCl_3$) of 1-(benzyloxy)-3-(5-fluoro-2-(pyridin-2-yl)phenyl)propan-2-ol (**3g**)

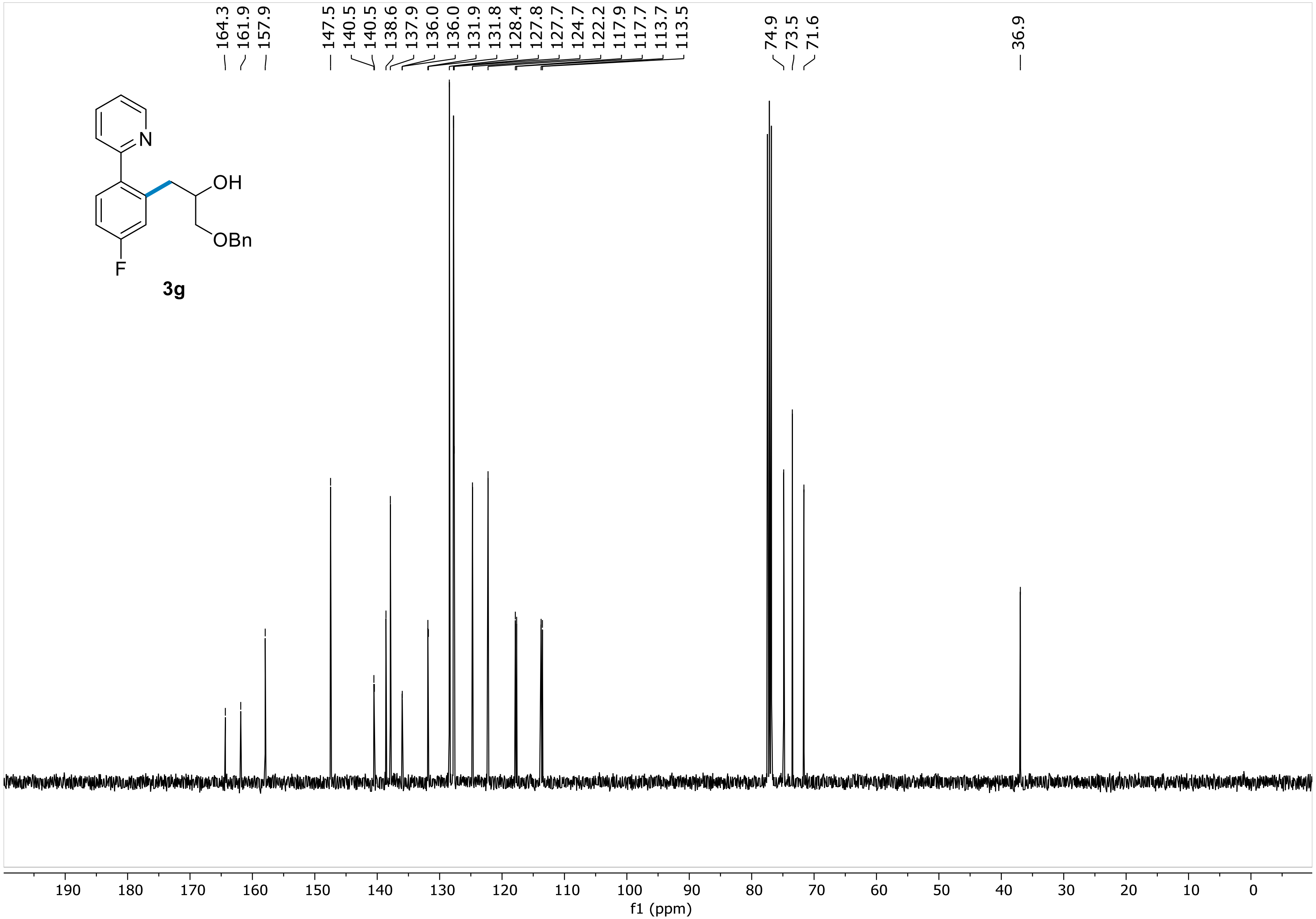

^{13}C NMR spectra (101 MHz, $CDCl_3$) of 1-(benzyloxy)-3-(5-fluoro-2-(pyridin-2-yl)phenyl)propan-2-ol (**3g**)

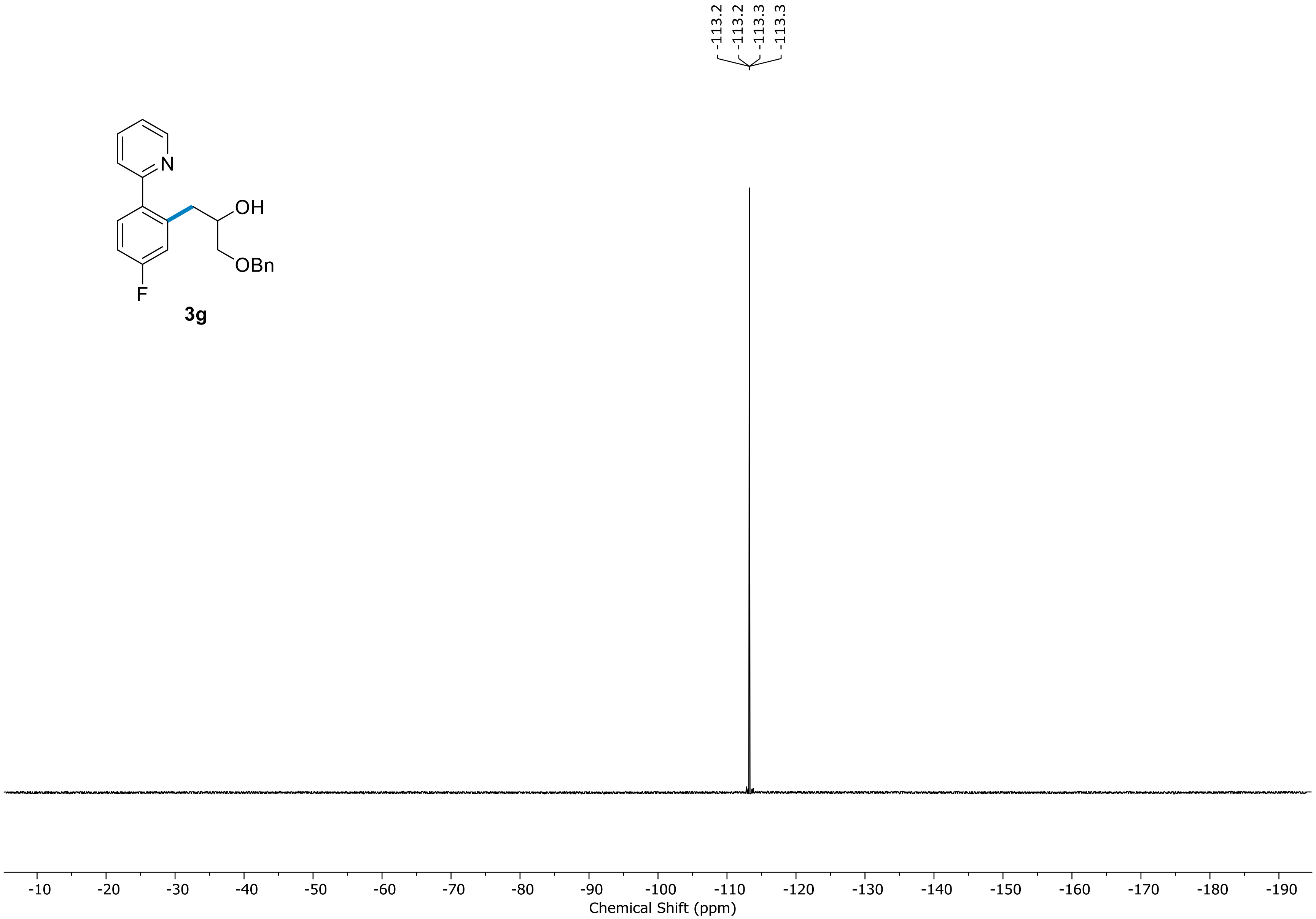

^{19}F NMR spectra (471 MHz, $CDCl_3$) of 1-(benzyloxy)-3-(5-fluoro-2-(pyridin-2-yl)phenyl)propan-2-ol (**3g**)

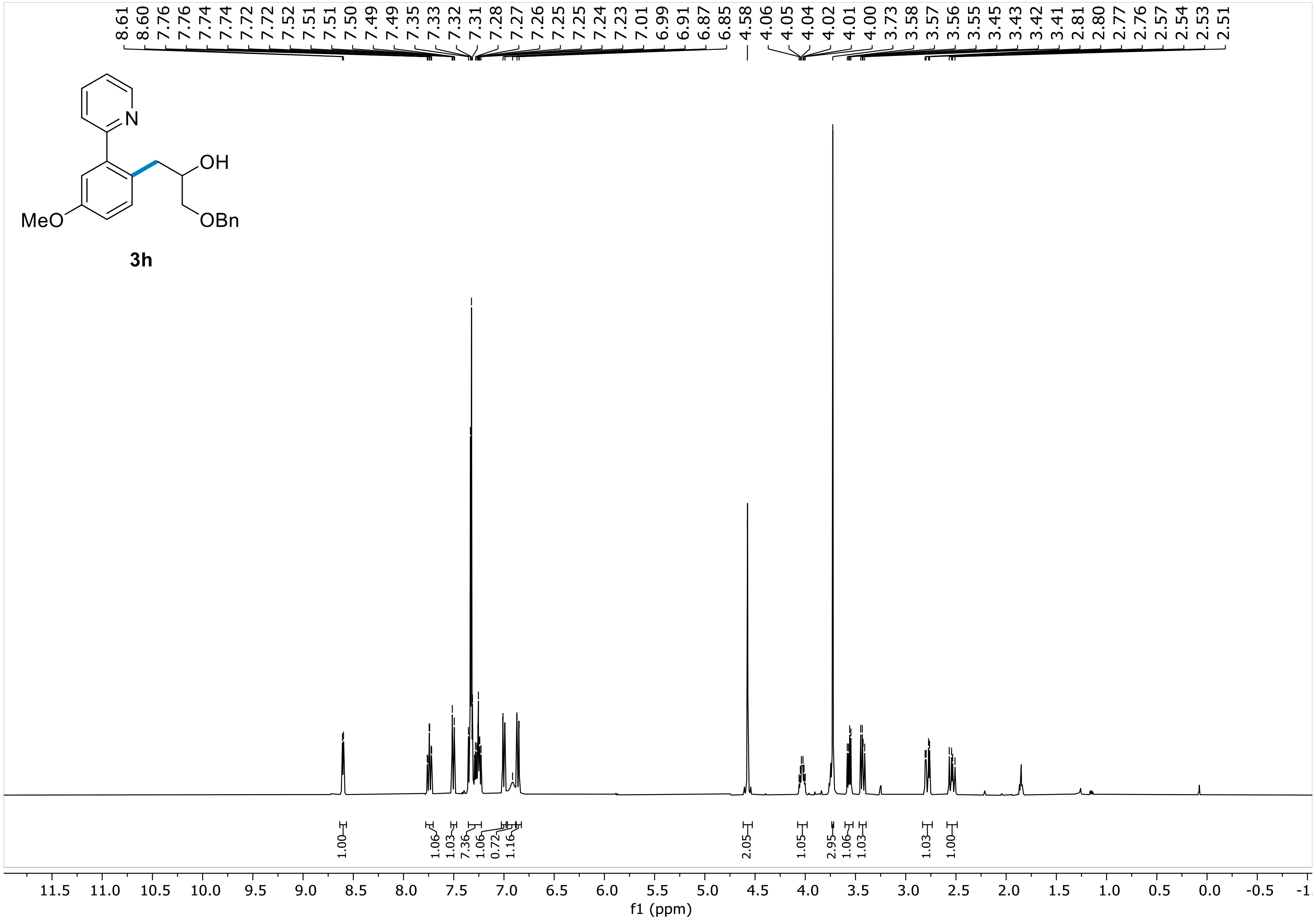

^{1}H NMR spectra (400 MHz, $CDCl_3$) of 1-(benzyloxy)-3-(4-methoxy-2-(pyridin-2-yl)phenyl)propan-2-ol (**3h**)

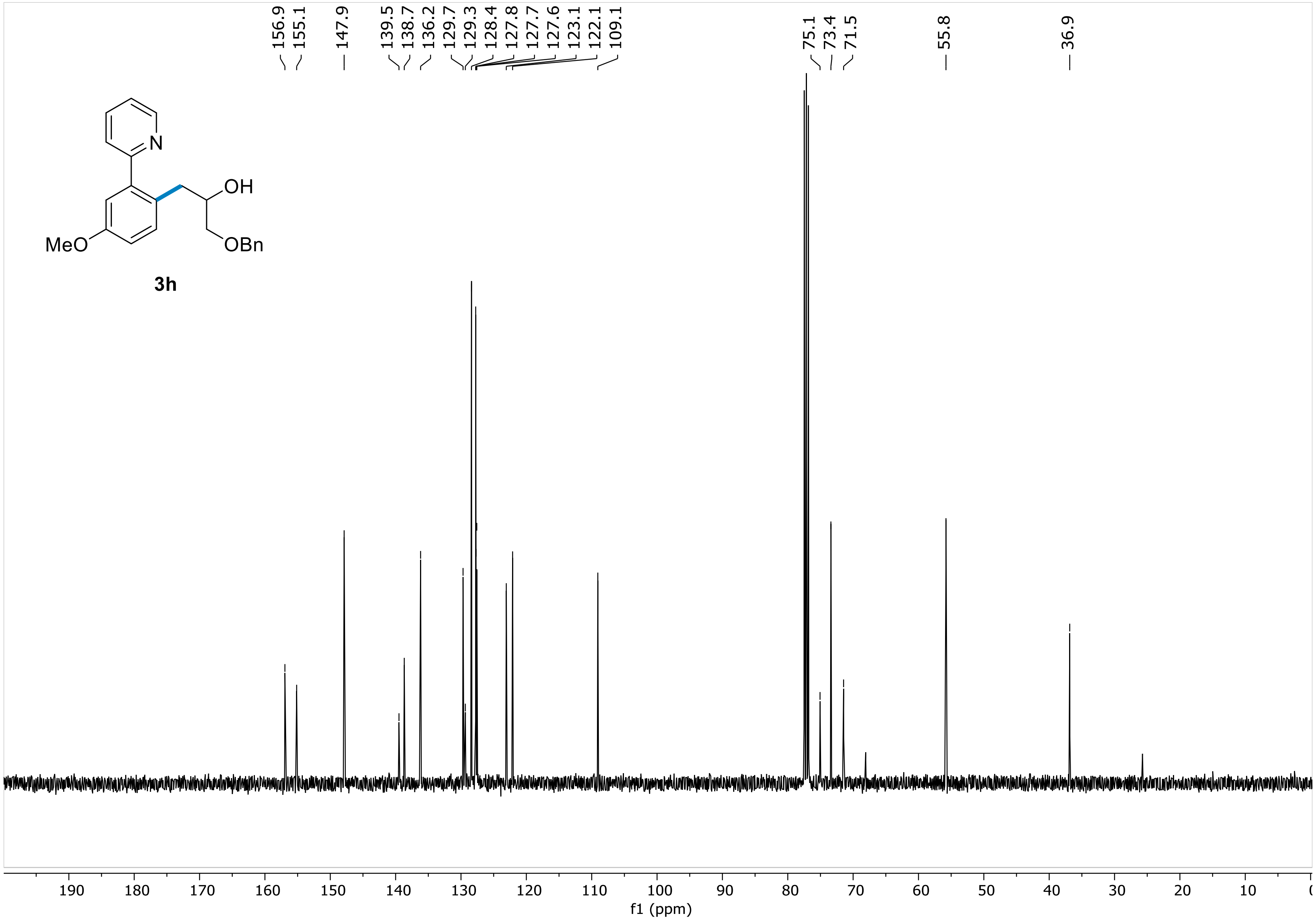

^{13}C NMR spectra (101 MHz, $CDCl_3$) of 1-(benzyloxy)-3-(4-methoxy-2-(pyridin-2-yl)phenyl)propan-2-ol (**3h**)

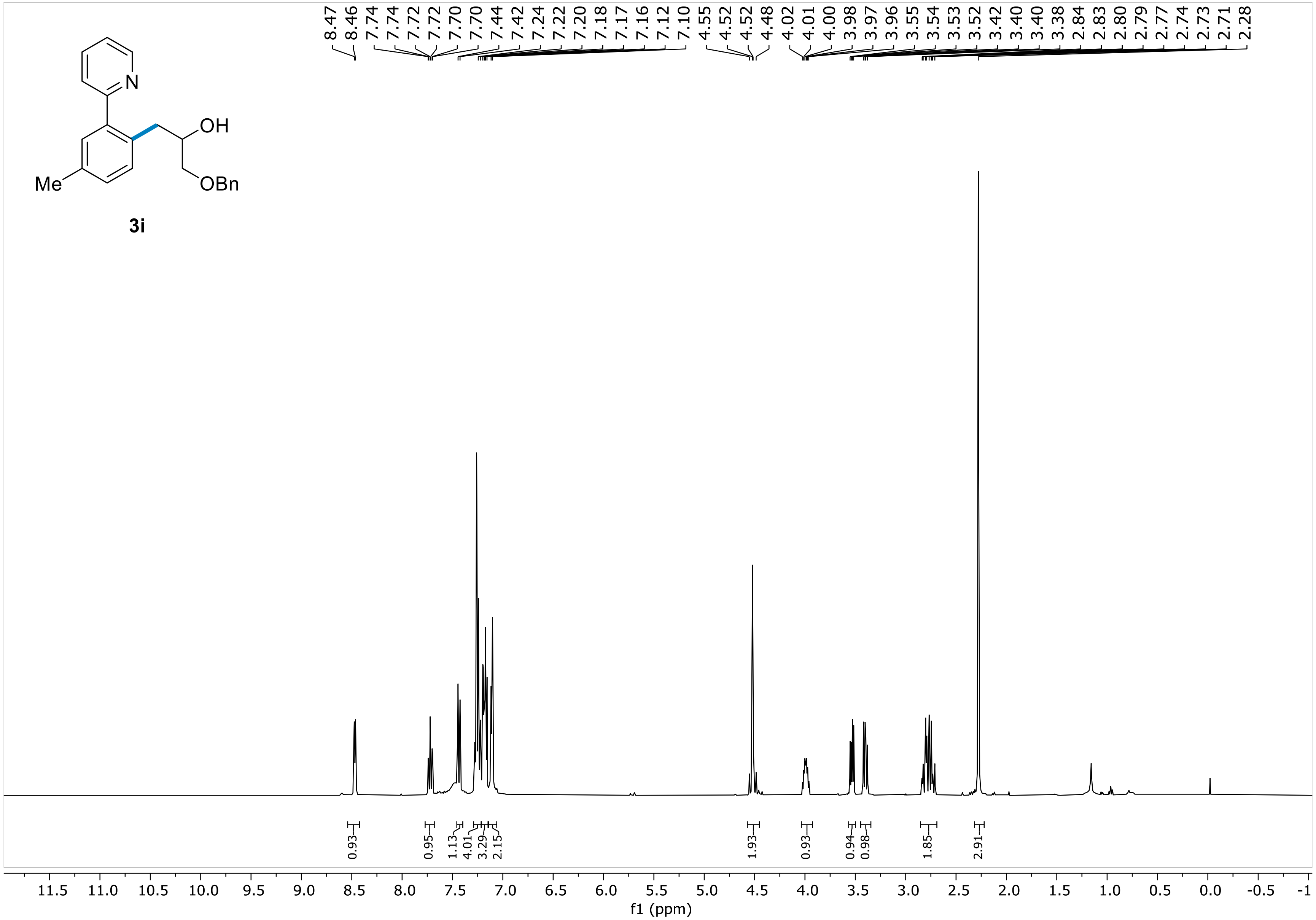

^{1}H NMR spectra (400 MHz, $CDCl_3$) of 1-(benzyloxy)-3-(4-methyl-2-(pyridin-2-yl)phenyl)propan-2-ol (**3i**)

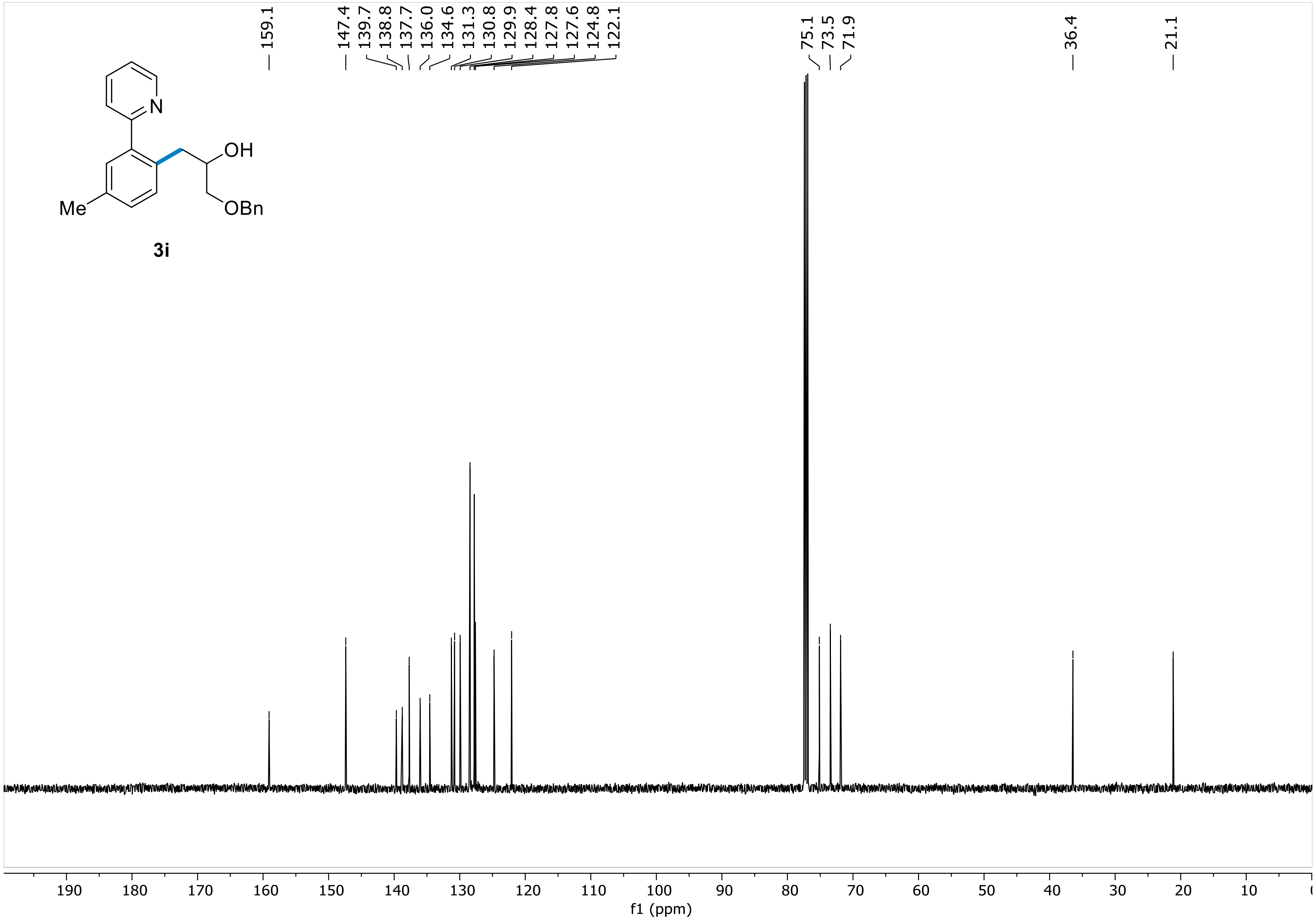

^{13}C NMR spectra (126 MHz, $CDCl_3$) of 1-(benzyloxy)-3-(4-methyl-2-(pyridin-2-yl)phenyl)propan-2-ol (**3i**)

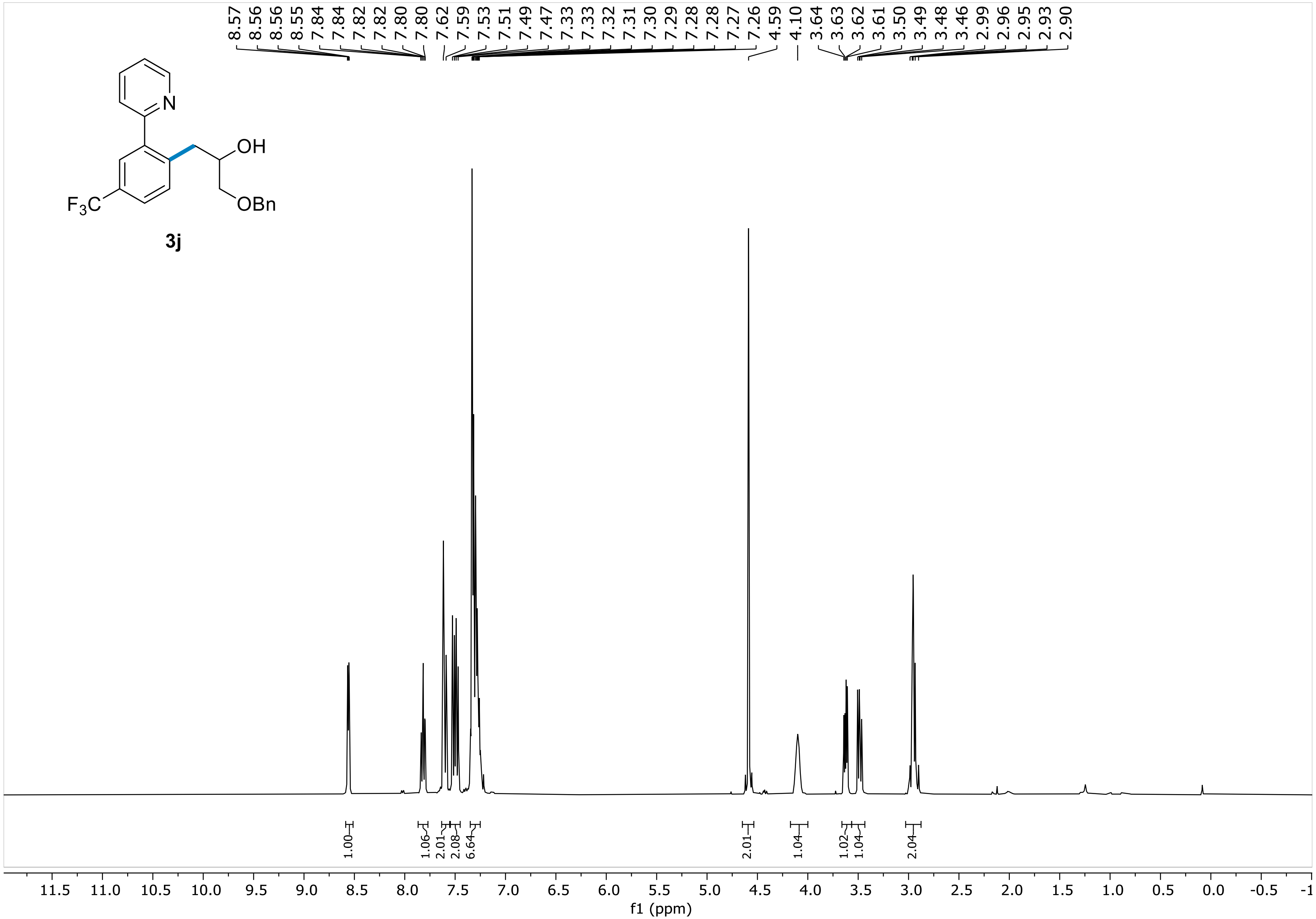

^{1}H NMR spectra (400 MHz, $CDCl_3$) of 1-(benzyloxy)-3-(2-(pyridin-2-yl)-4-(trifluoromethyl)phenyl)propan-2-ol (**3j**)

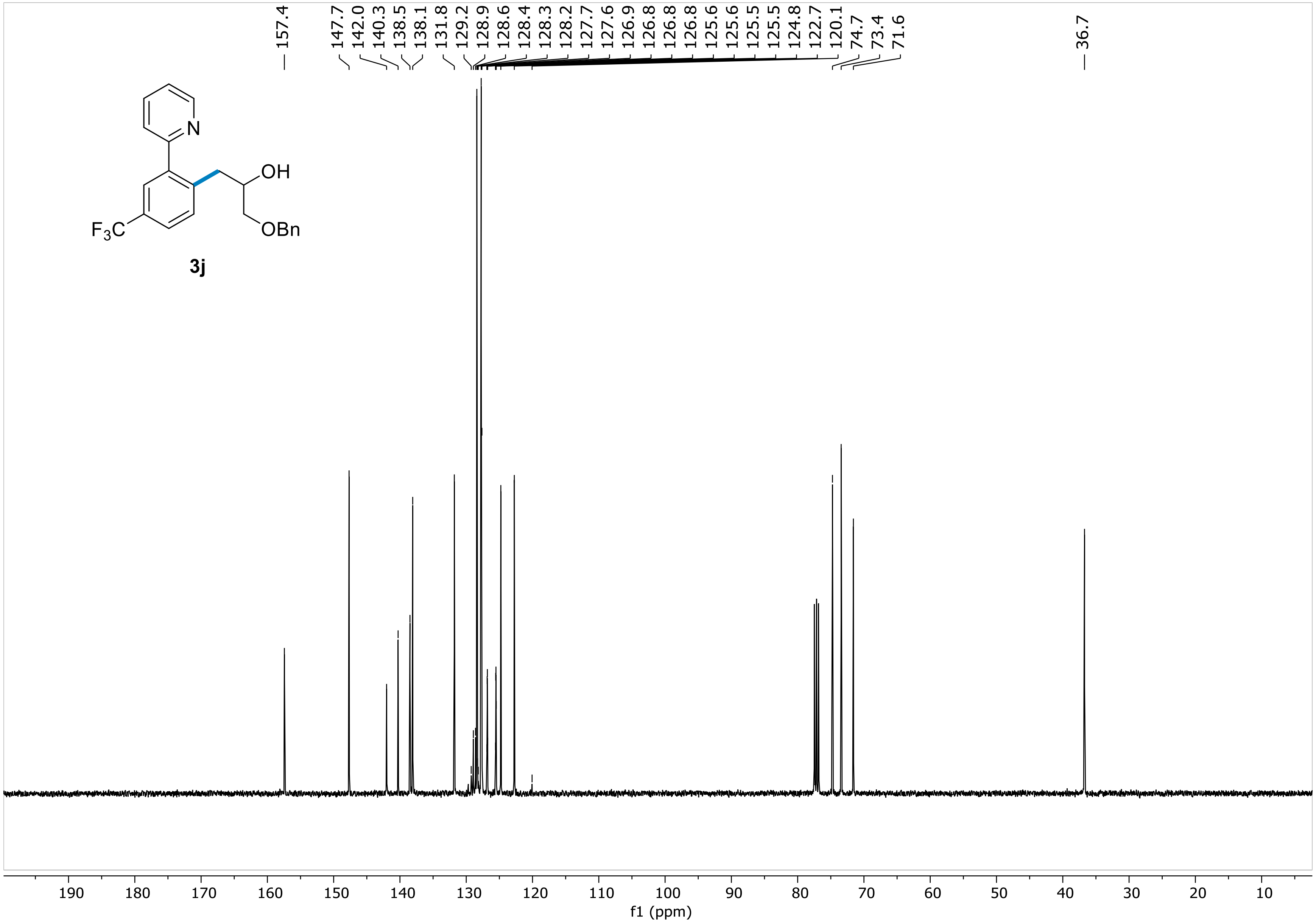

^{13}C NMR spectra (101 MHz, $CDCl_3$) of 1-(benzyloxy)-3-(2-(pyridin-2-yl)-4-(trifluoromethyl)phenyl)propan-2-ol (**3j**)

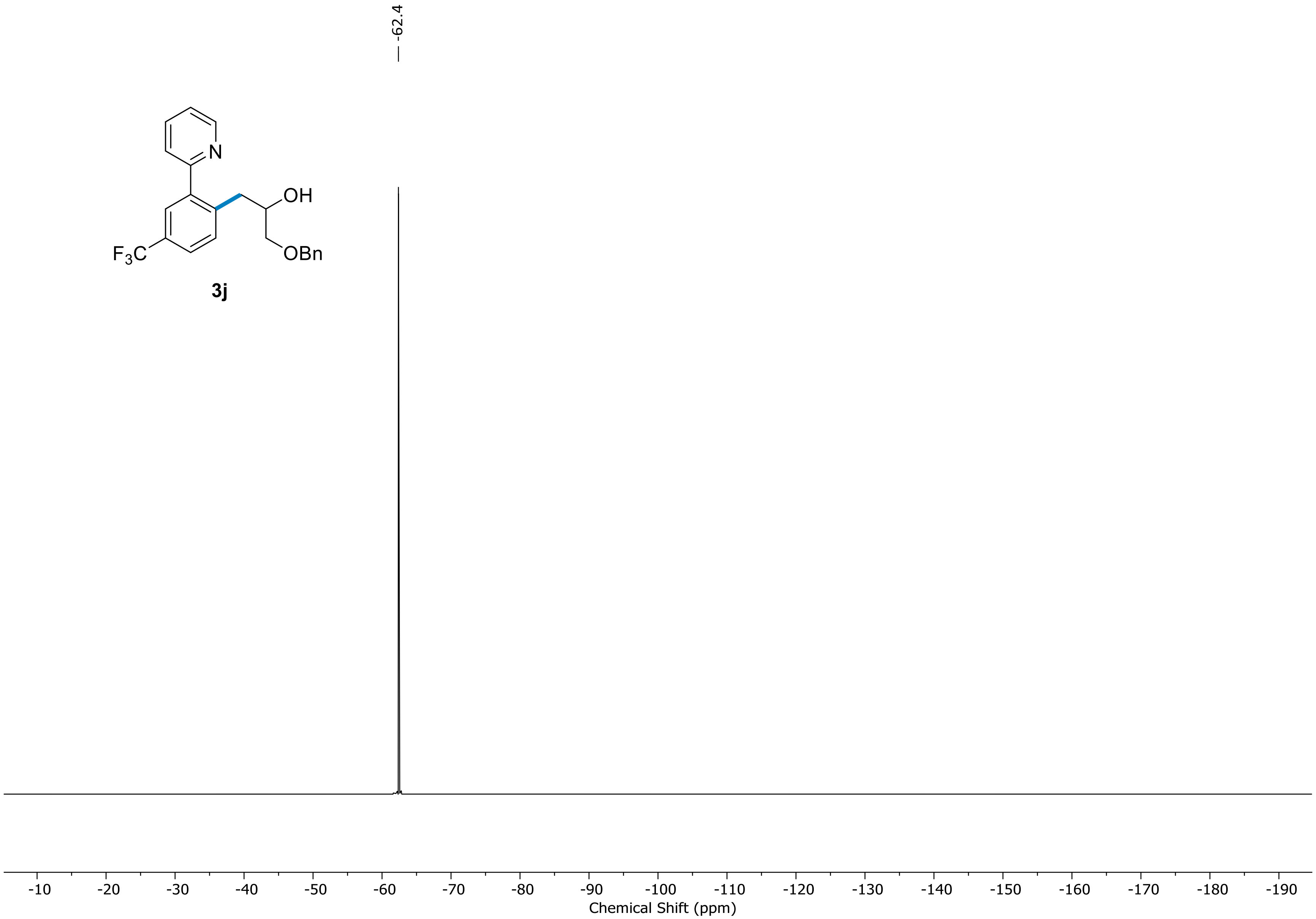

^{19}F NMR spectra (471 MHz, $CDCl_3$) of 1-(benzyloxy)-3-(2-(pyridin-2-yl)-4-(trifluoromethyl)phenyl)propan-2-ol (**3j**)

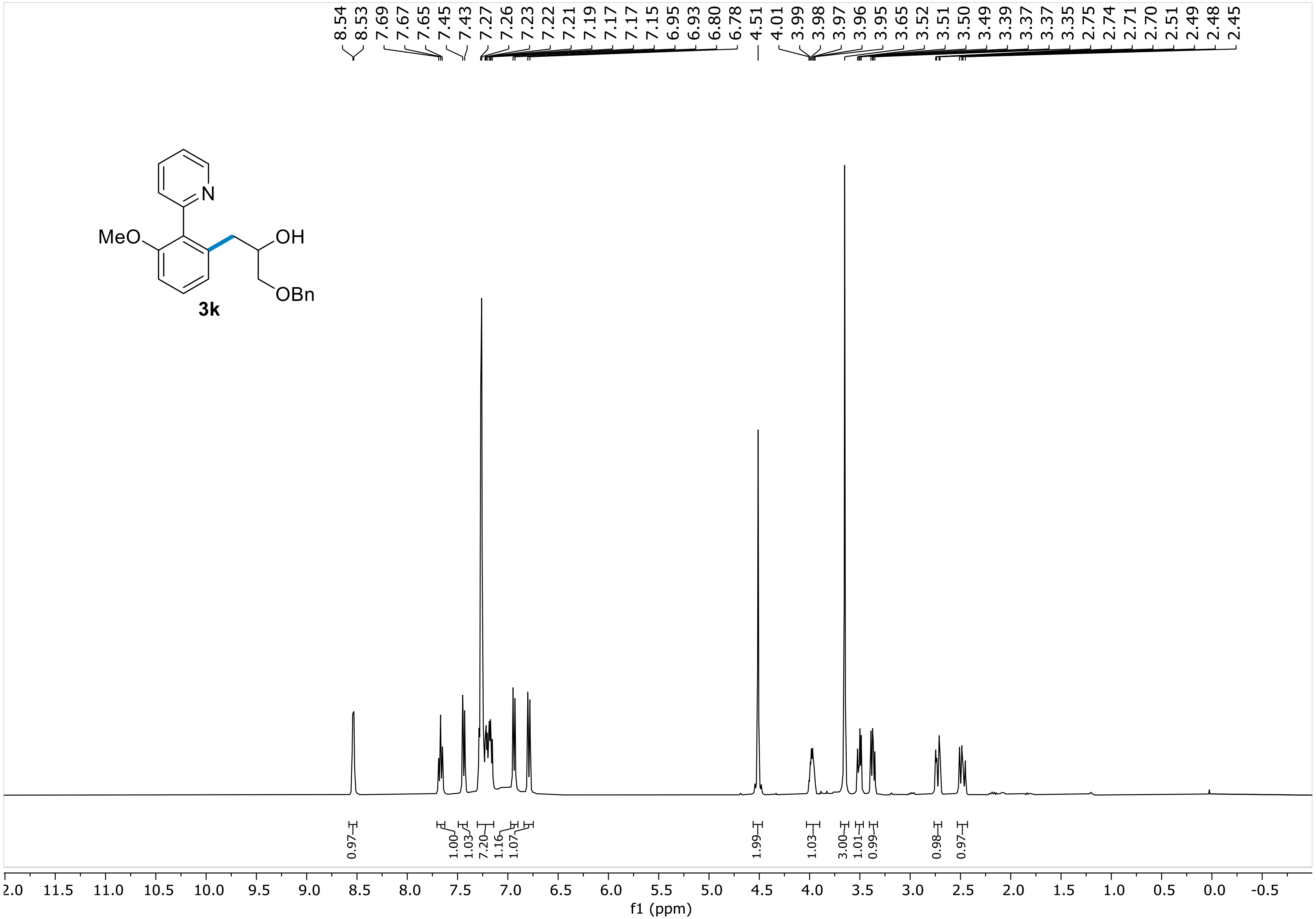

^{1}H NMR spectra (400 MHz, $CDCl_3$) of 1-(benzyloxy)-3-(3-methoxy-2-(pyridin-2-yl)phenyl)propan-2-ol (**3k**)

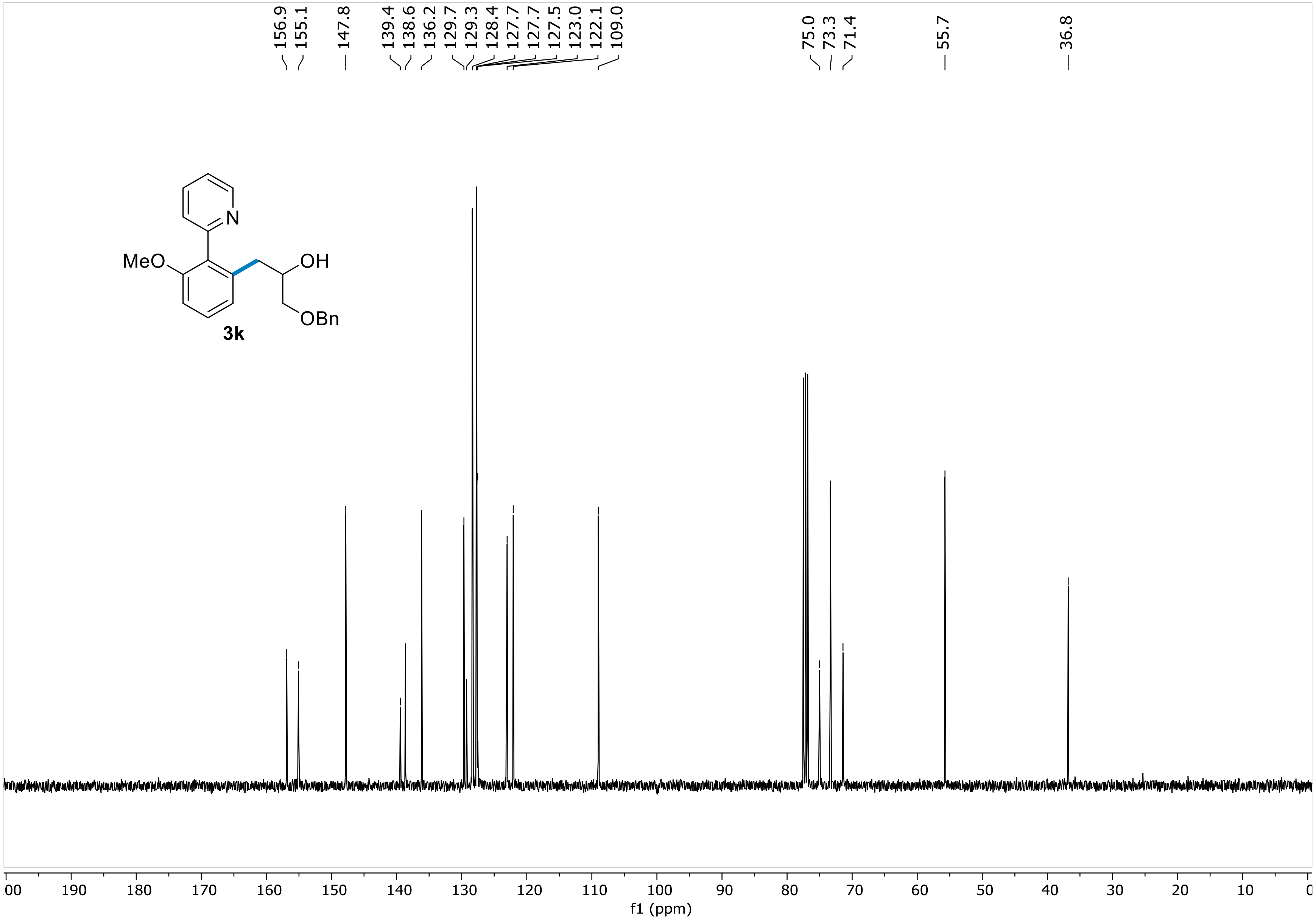

^{13}C NMR spectra (101 MHz, $CDCl_3$) of 1-(benzyloxy)-3-(3-methoxy-2-(pyridin-2-yl)phenyl)propan-2-ol (**3k**)

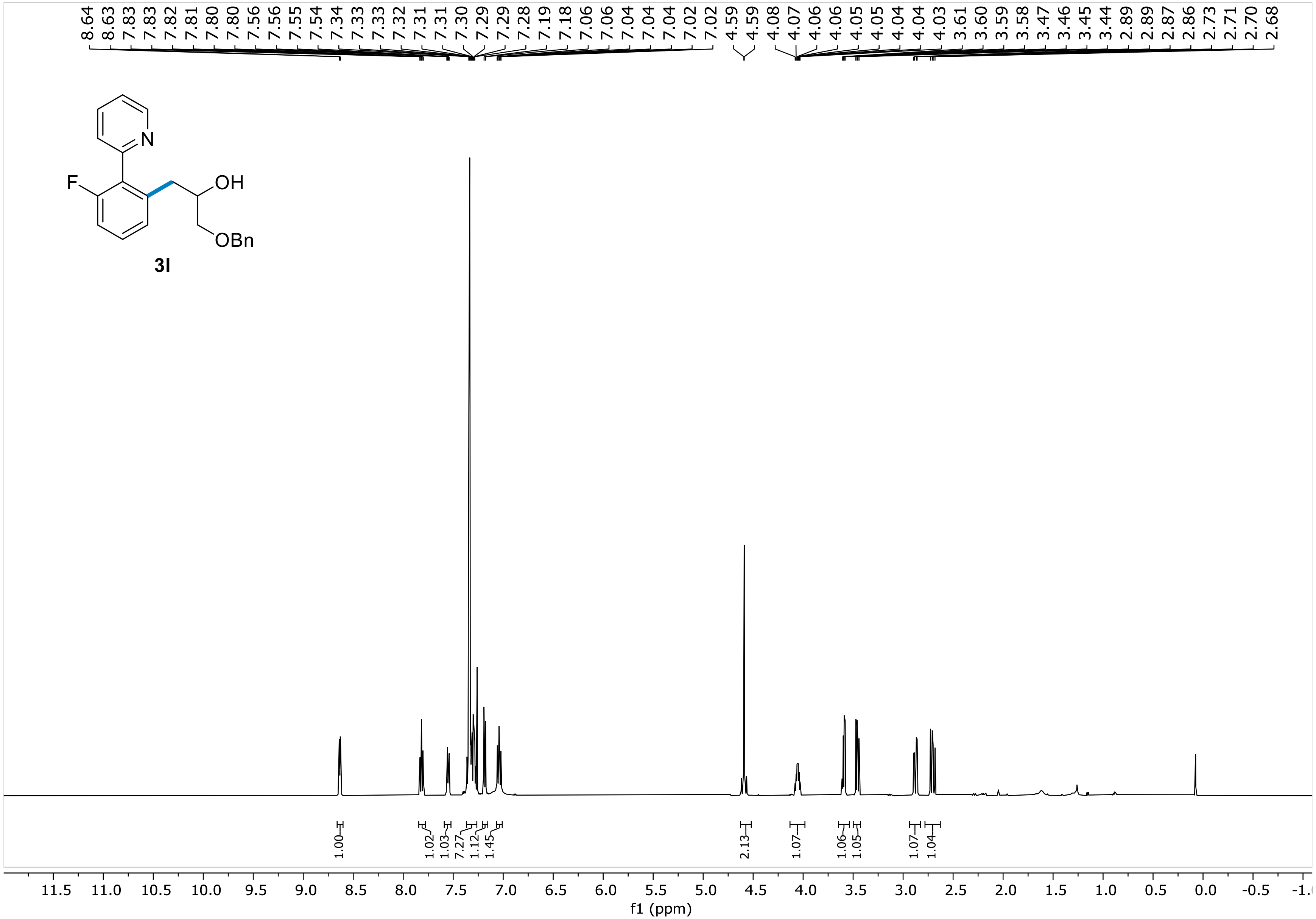

^{1}H NMR spectra (500 MHz, $CDCl_3$) of 1-(benzyloxy)-3-(3-fluoro-2-(pyridin-2-yl)phenyl)propan-2-ol (**3l**)

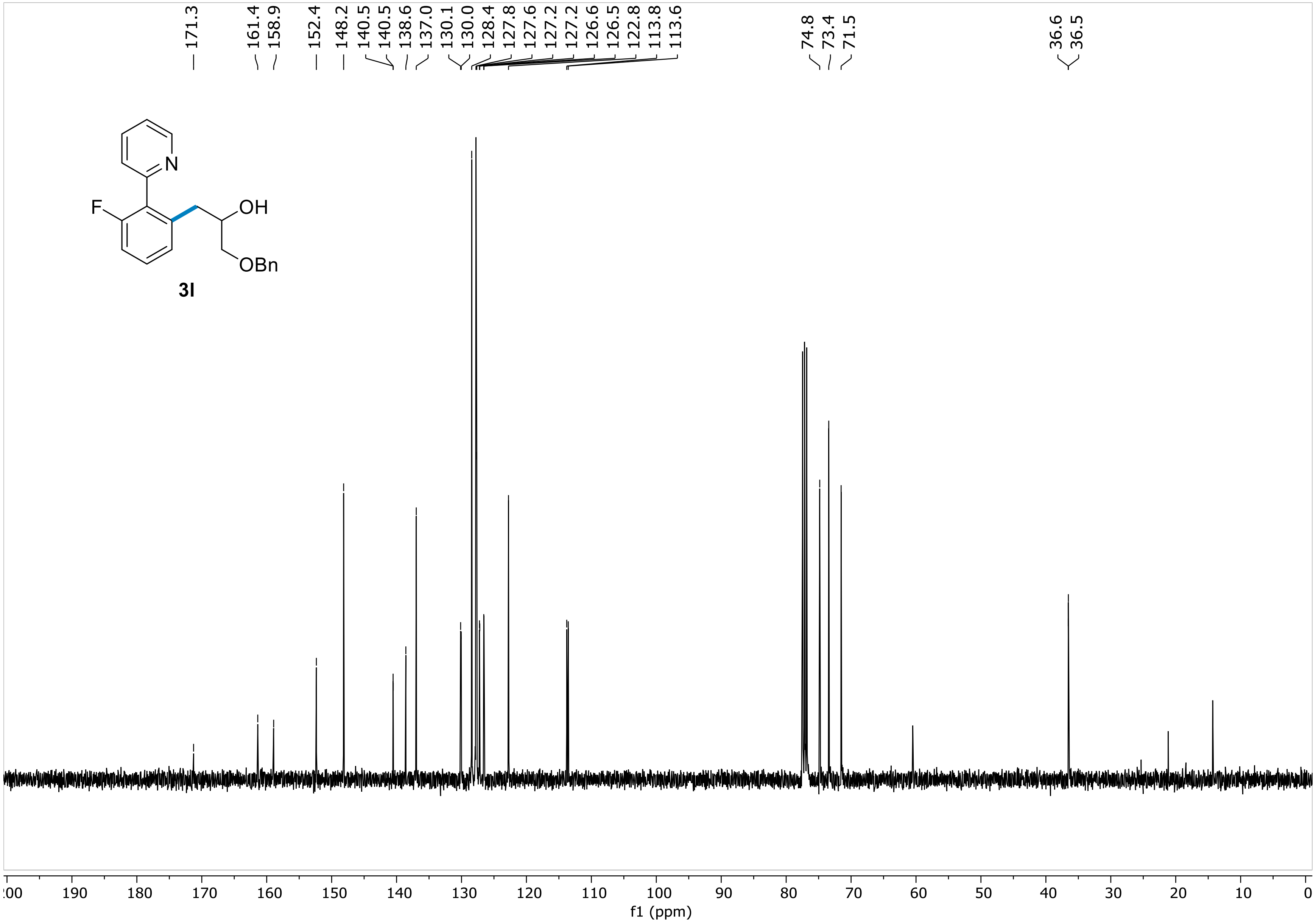

^{13}C NMR spectra (101 MHz, $CDCl_3$) of 1-(benzyloxy)-3-(3-fluoro-2-(pyridin-2-yl)phenyl)propan-2-ol (**3l**)

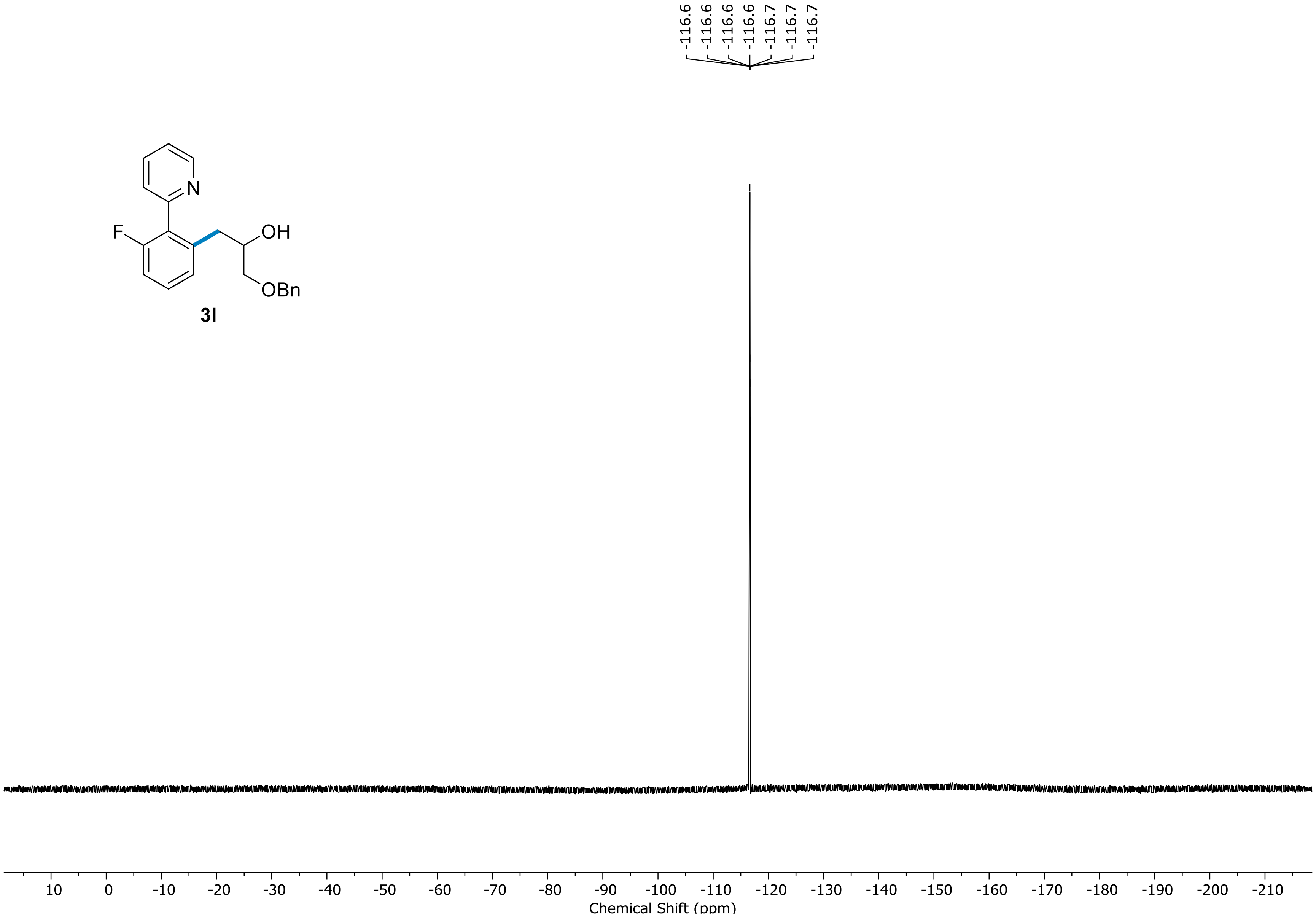

^{19}F NMR spectra (376 MHz, $CDCl_3$) of 1-(benzyloxy)-3-(3-fluoro-2-(pyridin-2-yl)phenyl)propan-2-ol (**3l**)

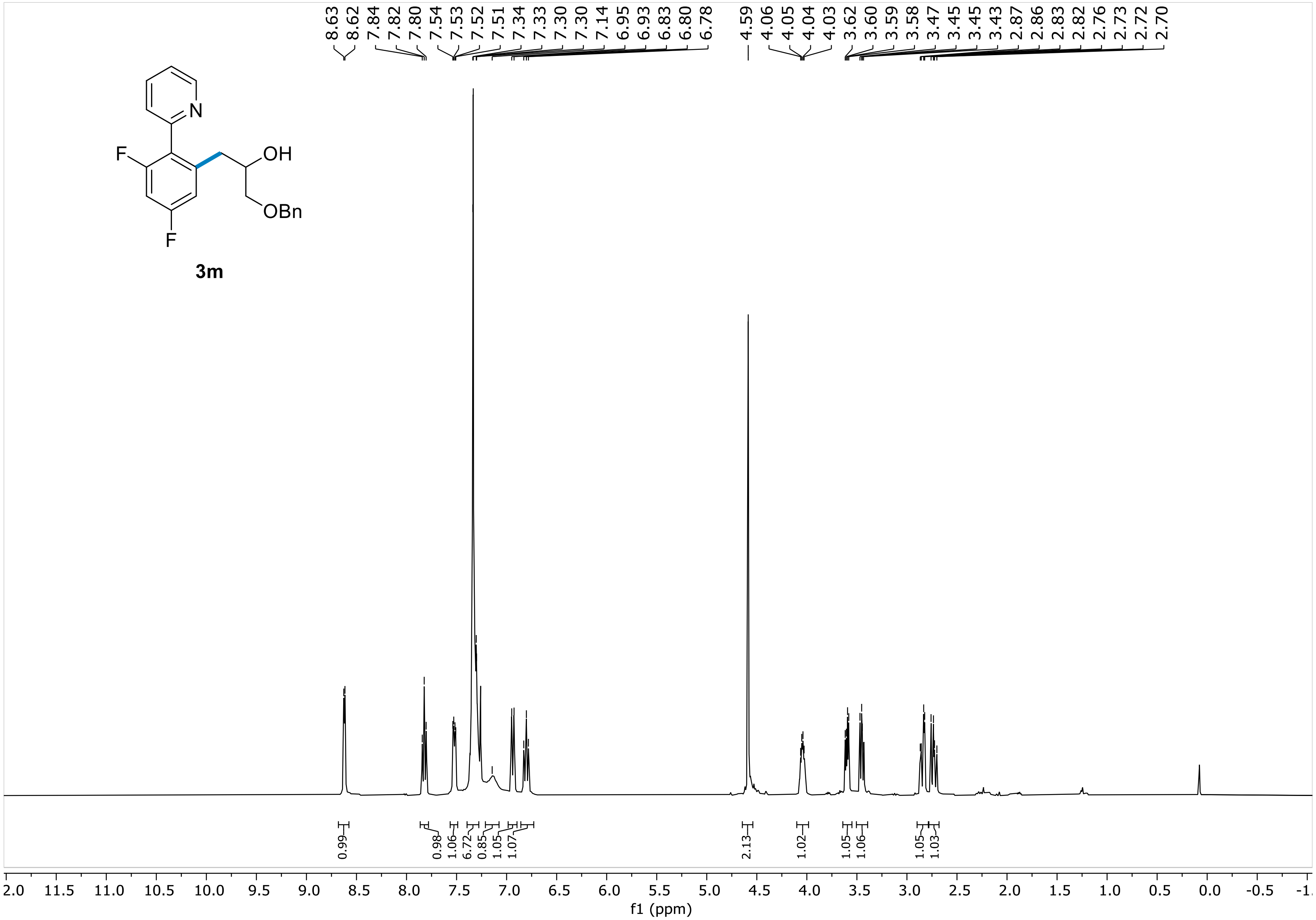

^{1}H NMR spectra (400 MHz, $CDCl_3$) of 1-(benzyloxy)-3-(3,5-difluoro-2-(pyridin-2-yl)phenyl)propan-2-ol (**3m**)

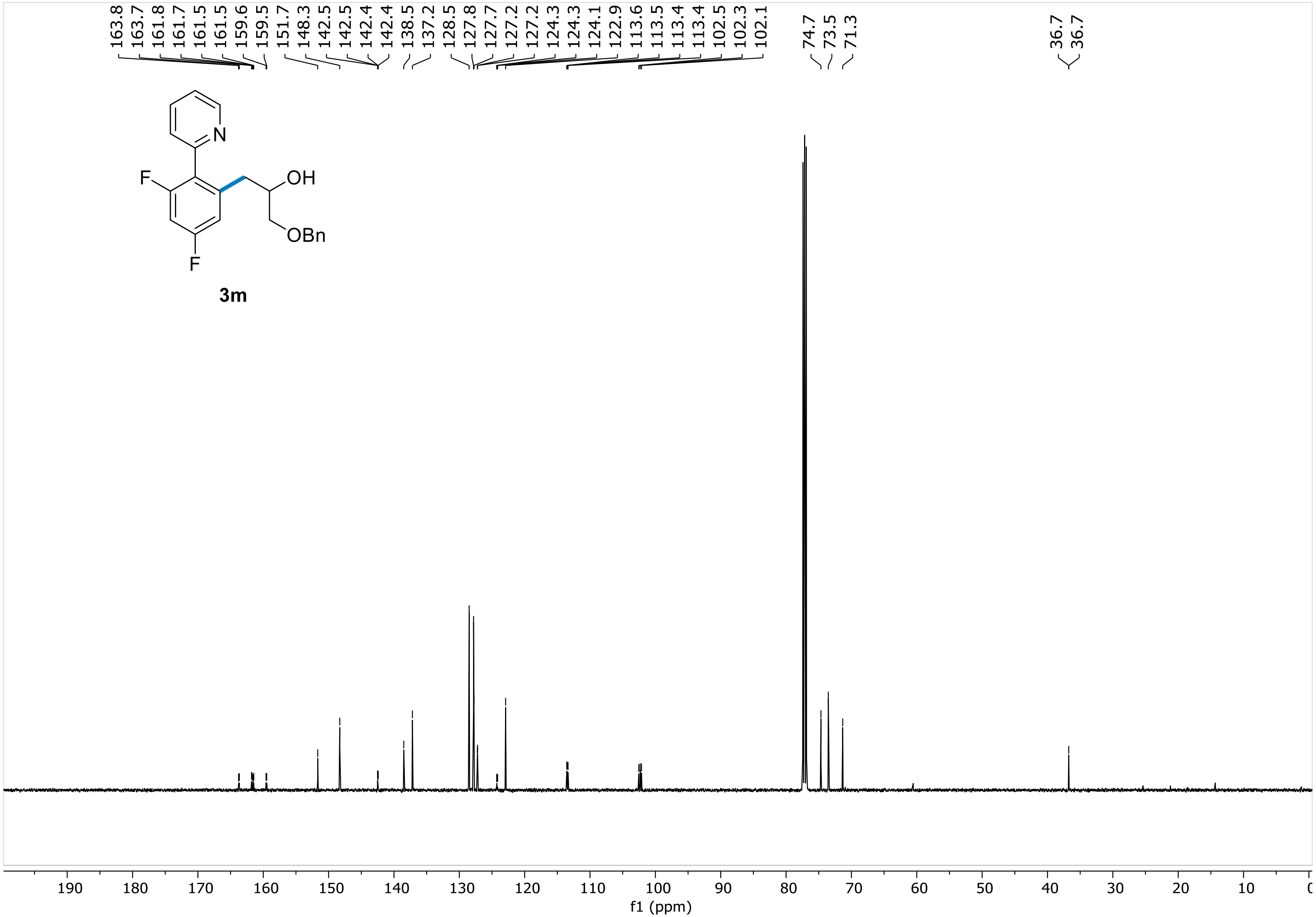

^{13}C NMR spectra (126 MHz, $CDCl_3$) of 1-(benzyloxy)-3-(3,5-difluoro-2-(pyridin-2-yl)phenyl)propan-2-ol (**3m**)

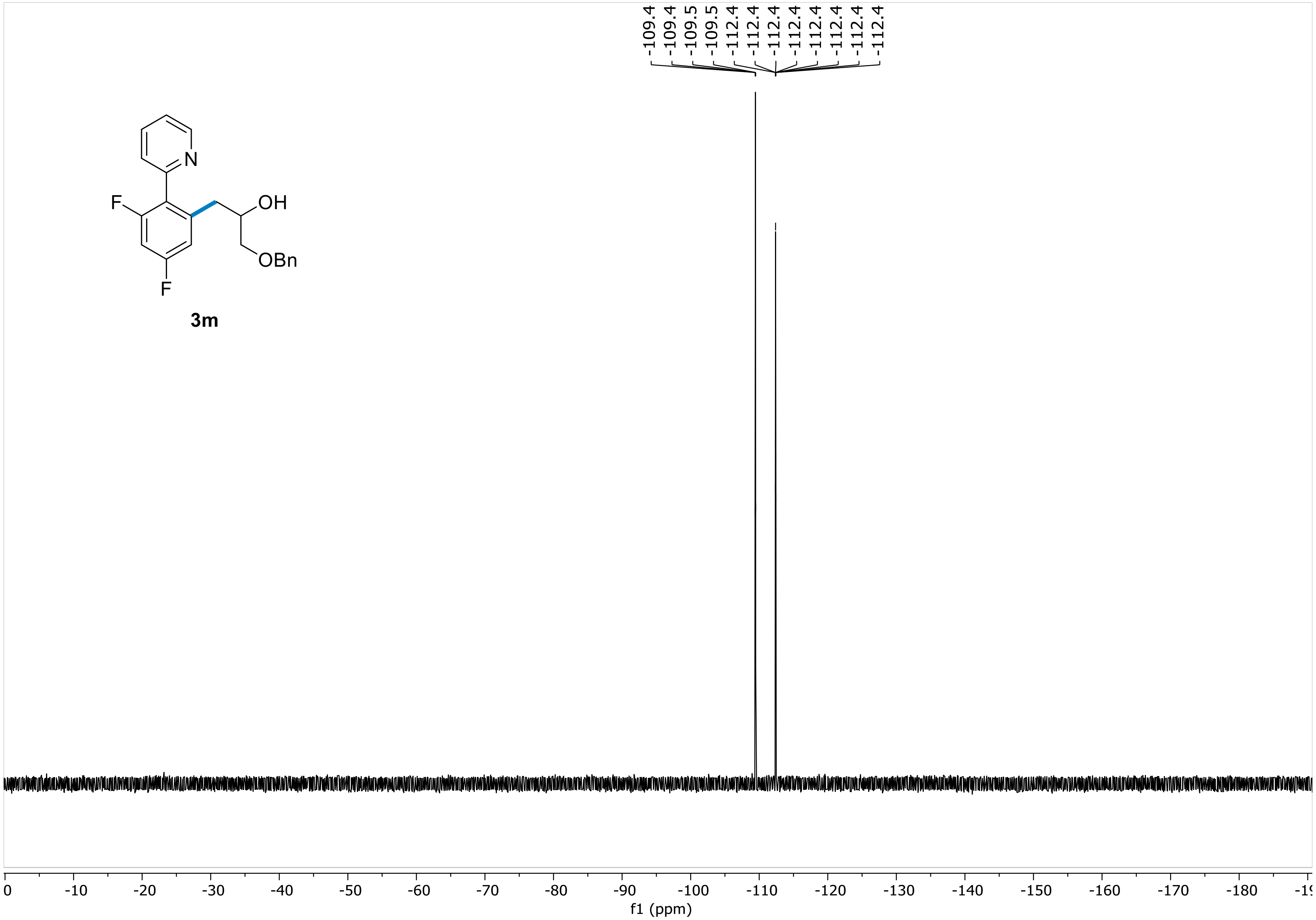

^{19}F NMR spectra (376 MHz, $CDCl_3$) of 1-(benzyloxy)-3-(3,5-difluoro-2-(pyridin-2-yl)phenyl)propan-2-ol (**3m**)

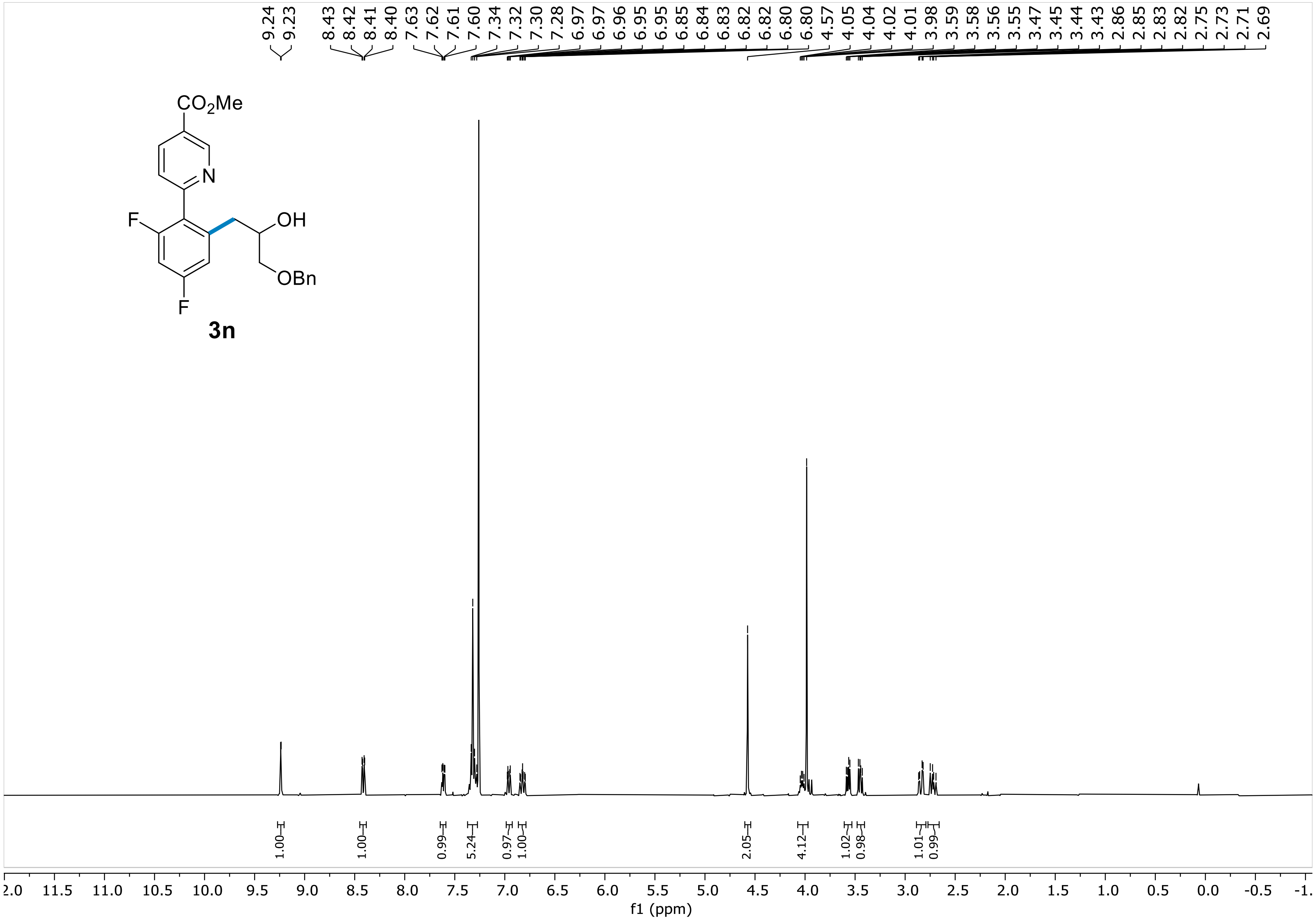

^{1}H NMR spectra (400 MHz, $CDCl_3$) of methyl 6-(2-(3-(benzyloxy)-2-hydroxypropyl)-4,6-difluorophenyl)nicotinate (**3n**)

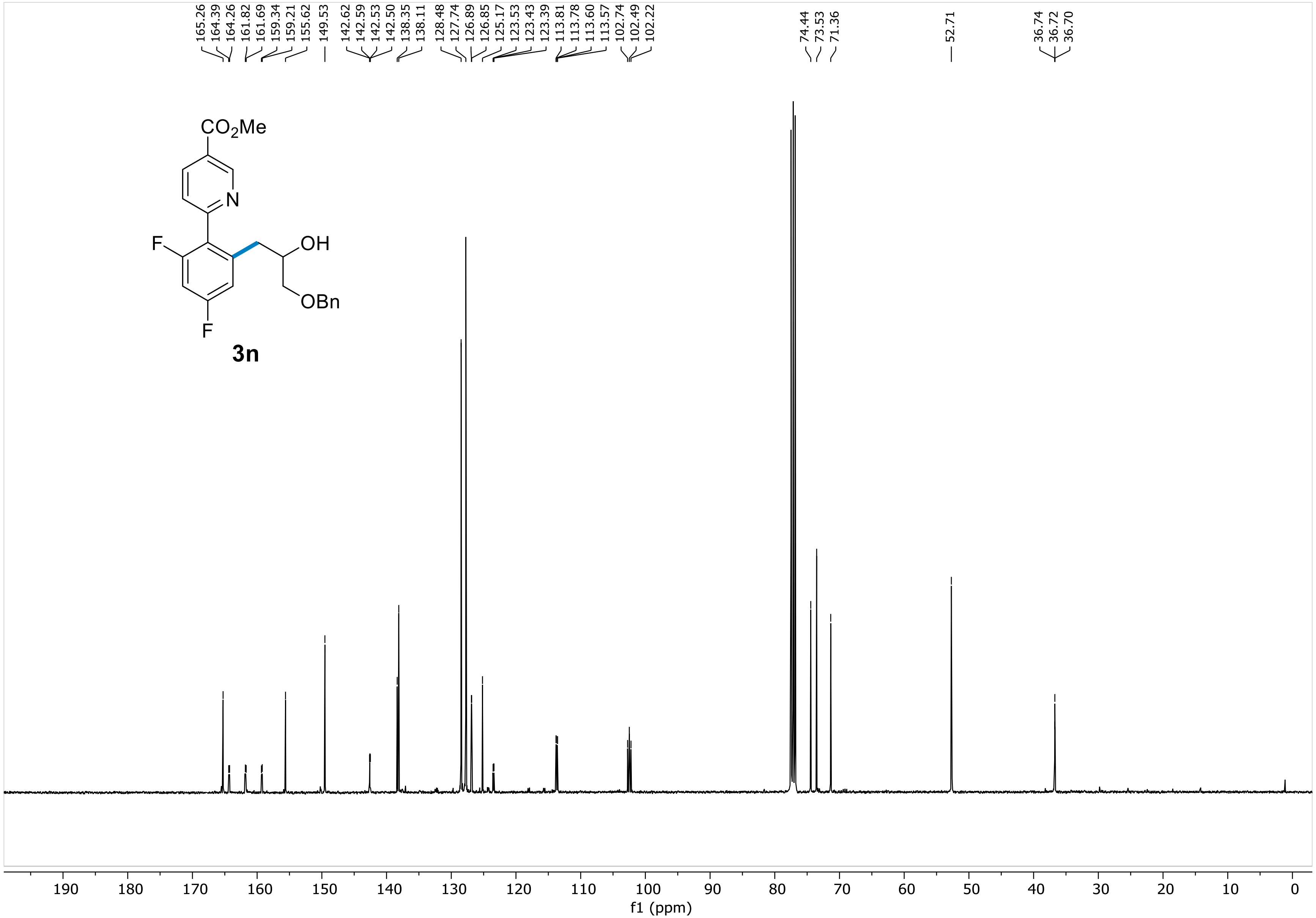

^{13}C NMR spectra (101 MHz, $CDCl_3$) of methyl 6-(2-(3-(benzyloxy)-2-hydroxypropyl)-4,6-difluorophenyl)nicotinate (**3n**)

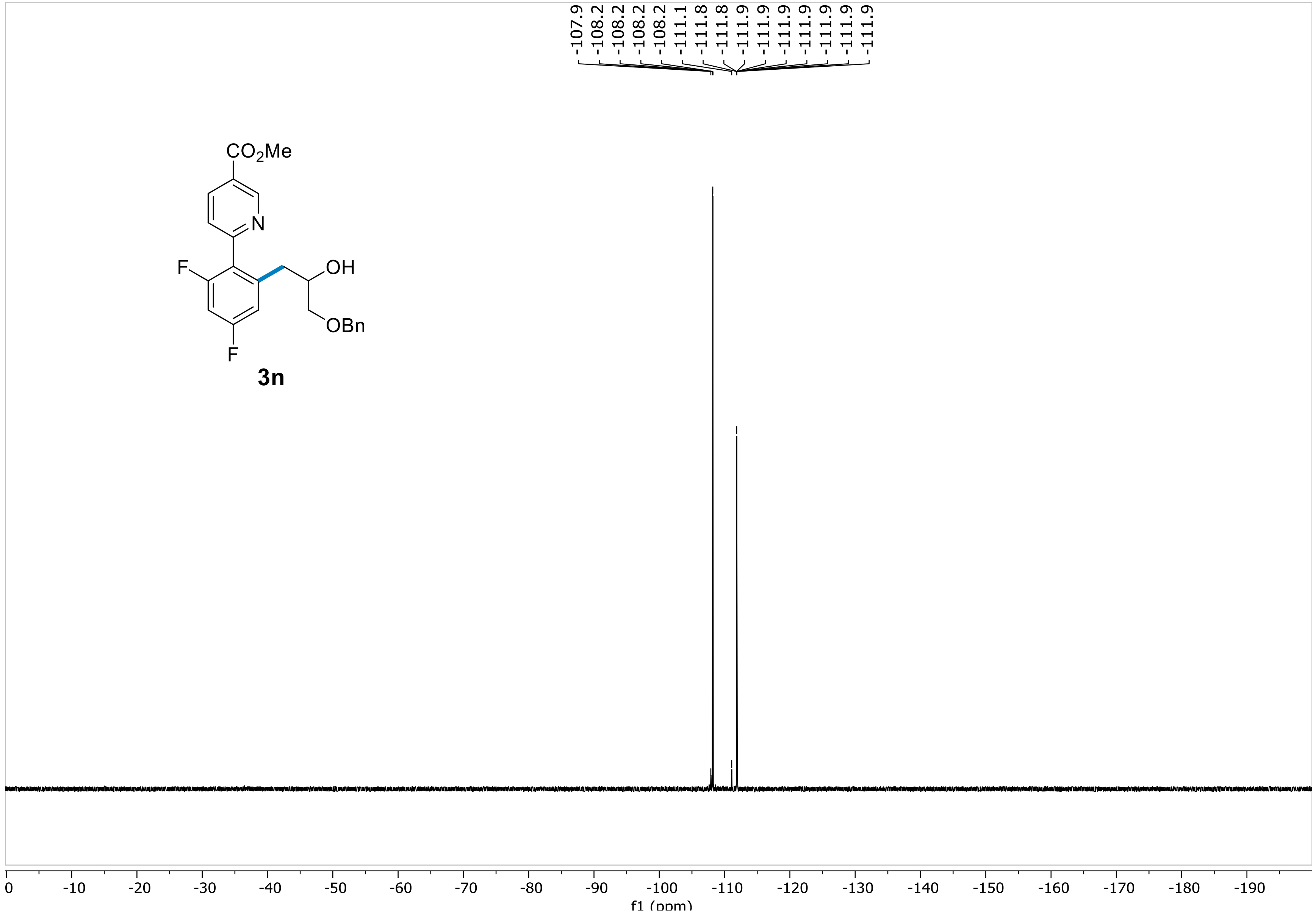

^{19}F NMR spectra (376 MHz, $CDCl_3$) of methyl 6-(2-(3-(benzyloxy)-2-hydroxypropyl)-4,6-difluorophenyl)nicotinate (**3n**)

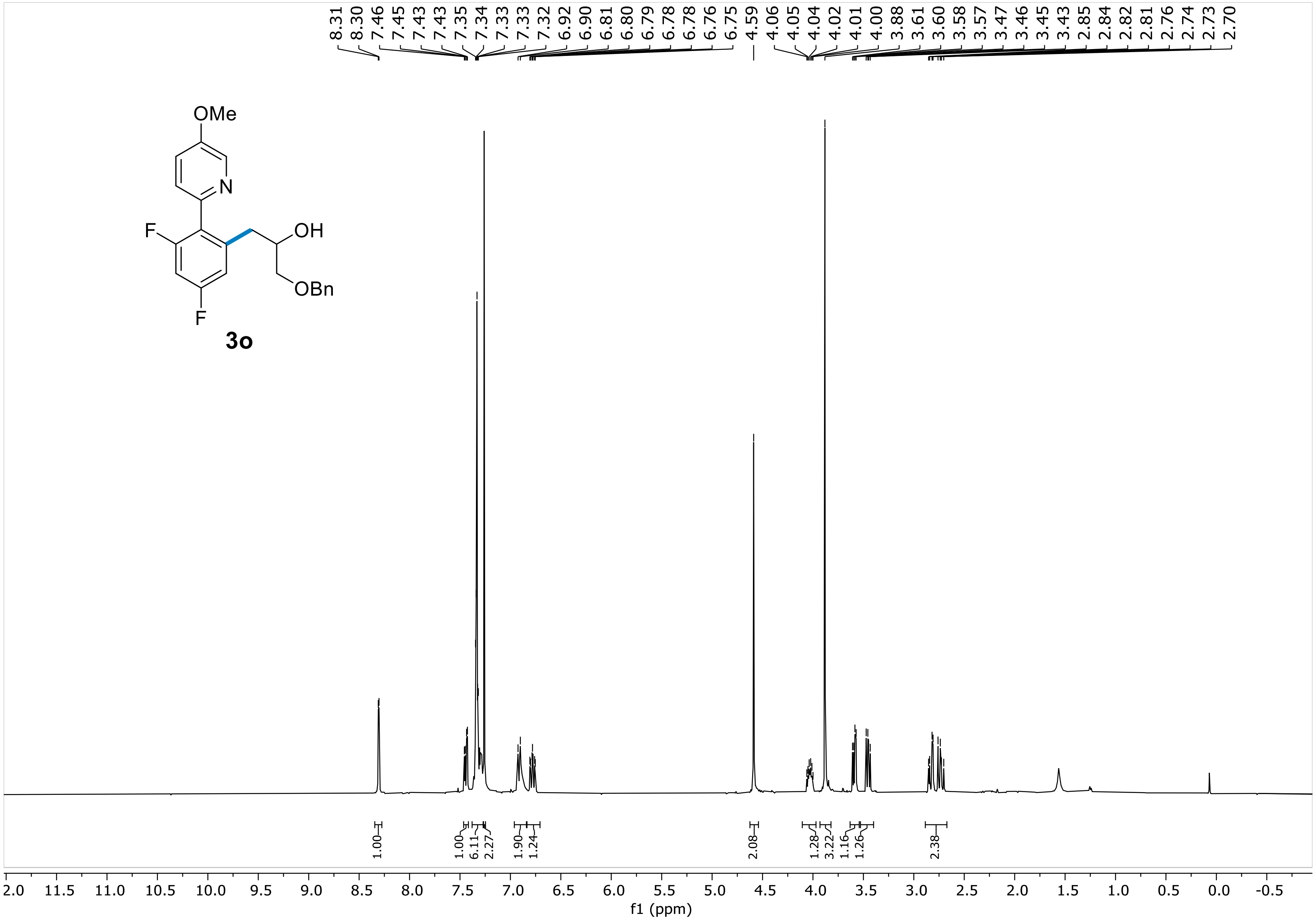

^{1}H NMR spectra (400 MHz, $CDCl_3$) of 1-(benzyloxy)-3-(3,5-difluoro-2-(5-methoxypyridin-2-yl)phenyl)propan-2-ol (**3o**)

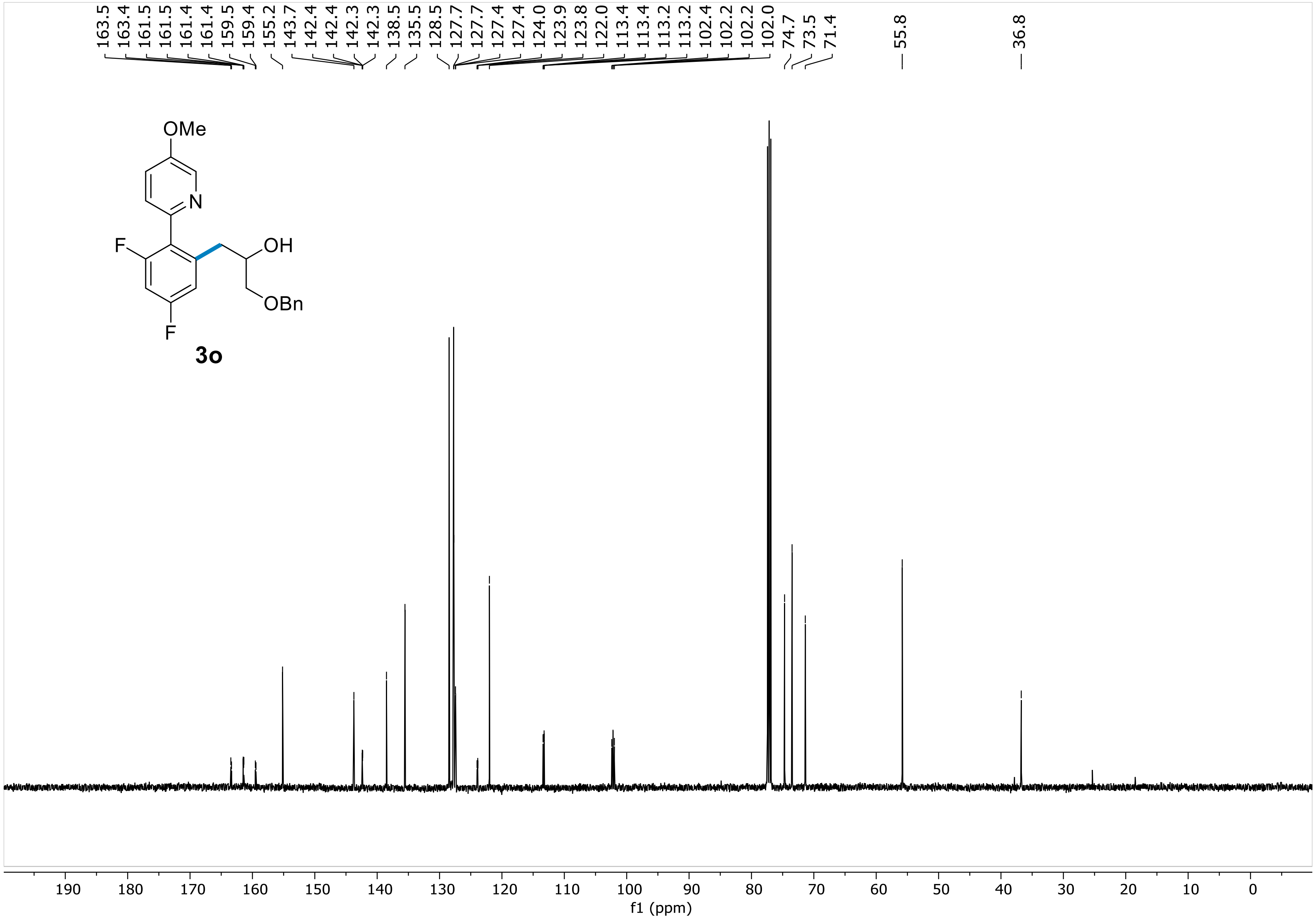

^{13}C NMR spectra (126 MHz, $CDCl_3$) of 1-(benzyloxy)-3-(3,5-difluoro-2-(5-methoxypyridin-2-yl)phenyl)propan-2-ol (**3o**)

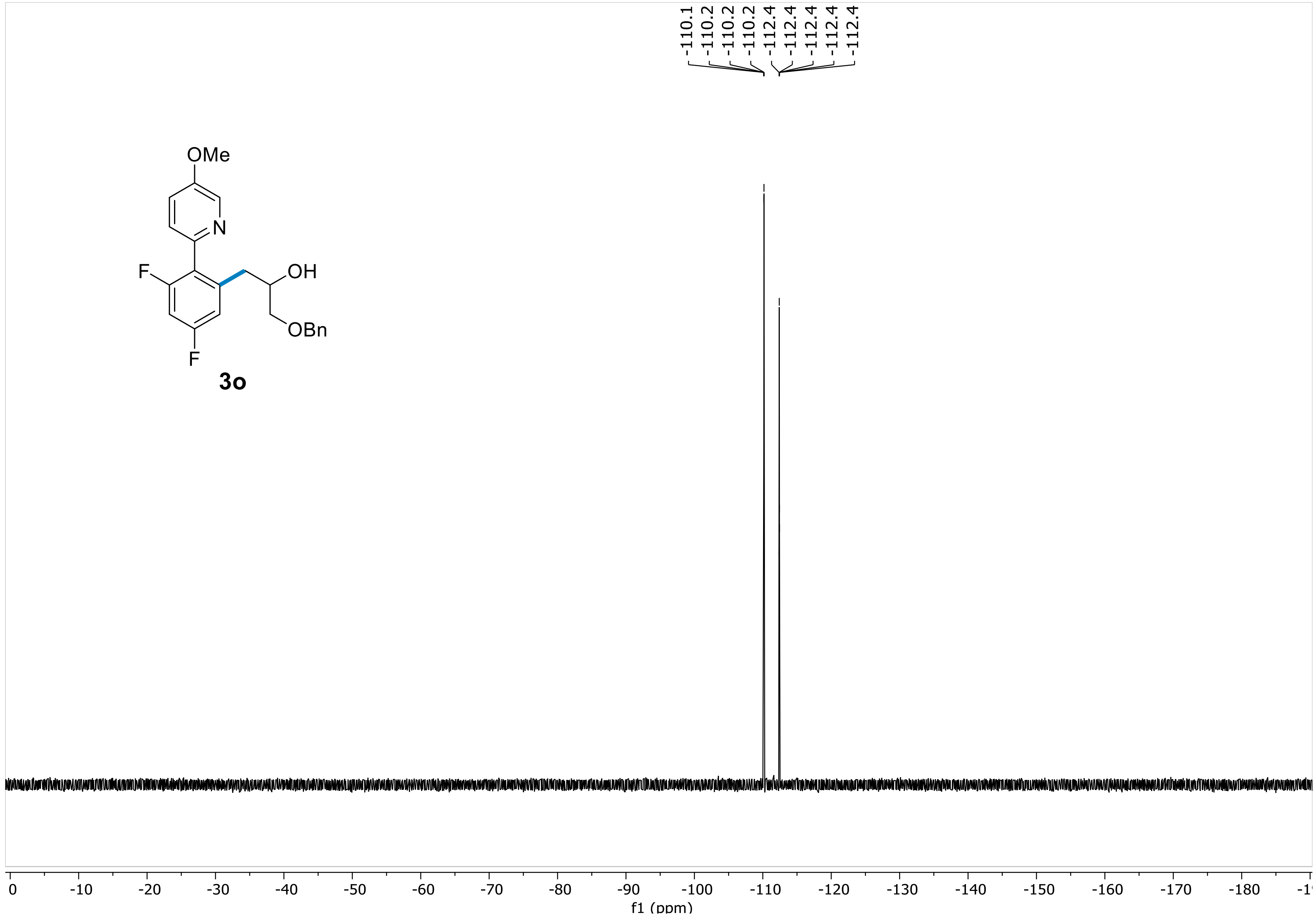

^{19}F NMR spectra (376 MHz, $CDCl_3$) of 1-(benzyloxy)-3-(3,5-difluoro-2-(5-methoxypyridin-2-yl)phenyl)propan-2-ol (**3o**)

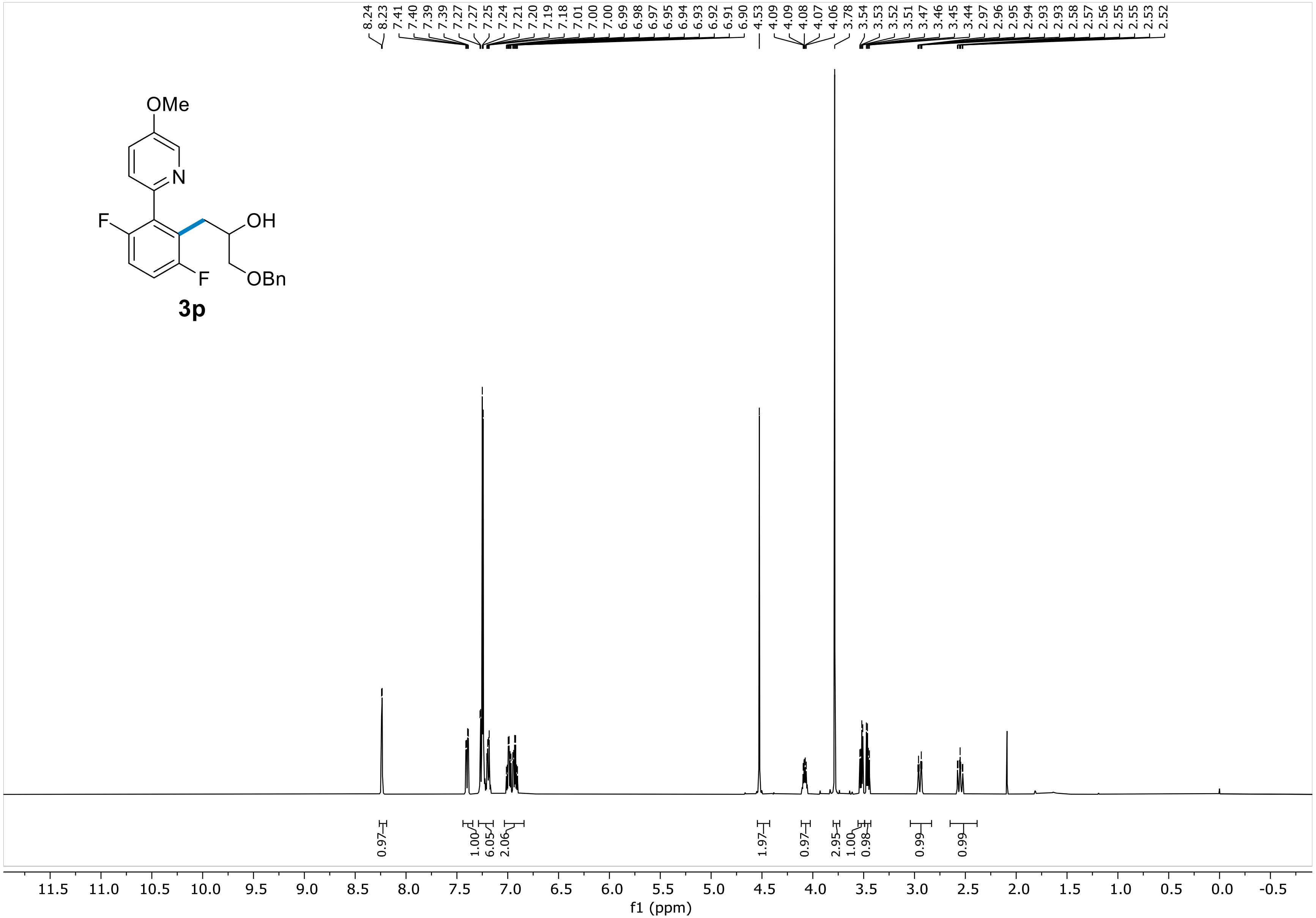

^{1}H NMR spectra (500 MHz, $CDCl_3$) of 1-(benzyloxy)-3-(3,6-difluoro-2-(5-methoxypyridin-2-yl)phenyl)propan-2-ol (**3p**)

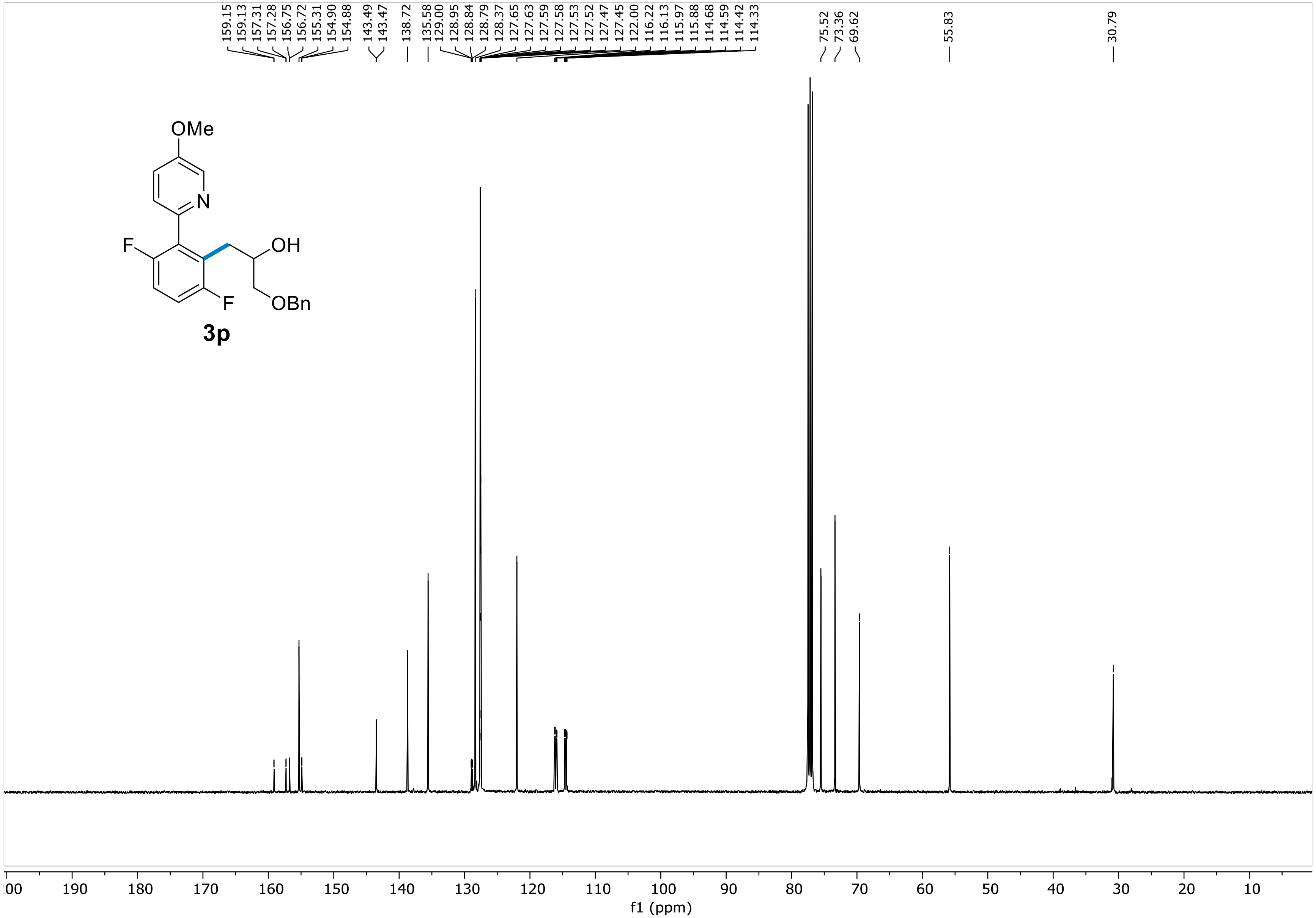

^{13}C NMR spectra (101 MHz, $CDCl_3$) of 1-(benzyloxy)-3-(3,6-difluoro-2-(5-methoxypyridin-2-yl)phenyl)propan-2-ol (**3p**)

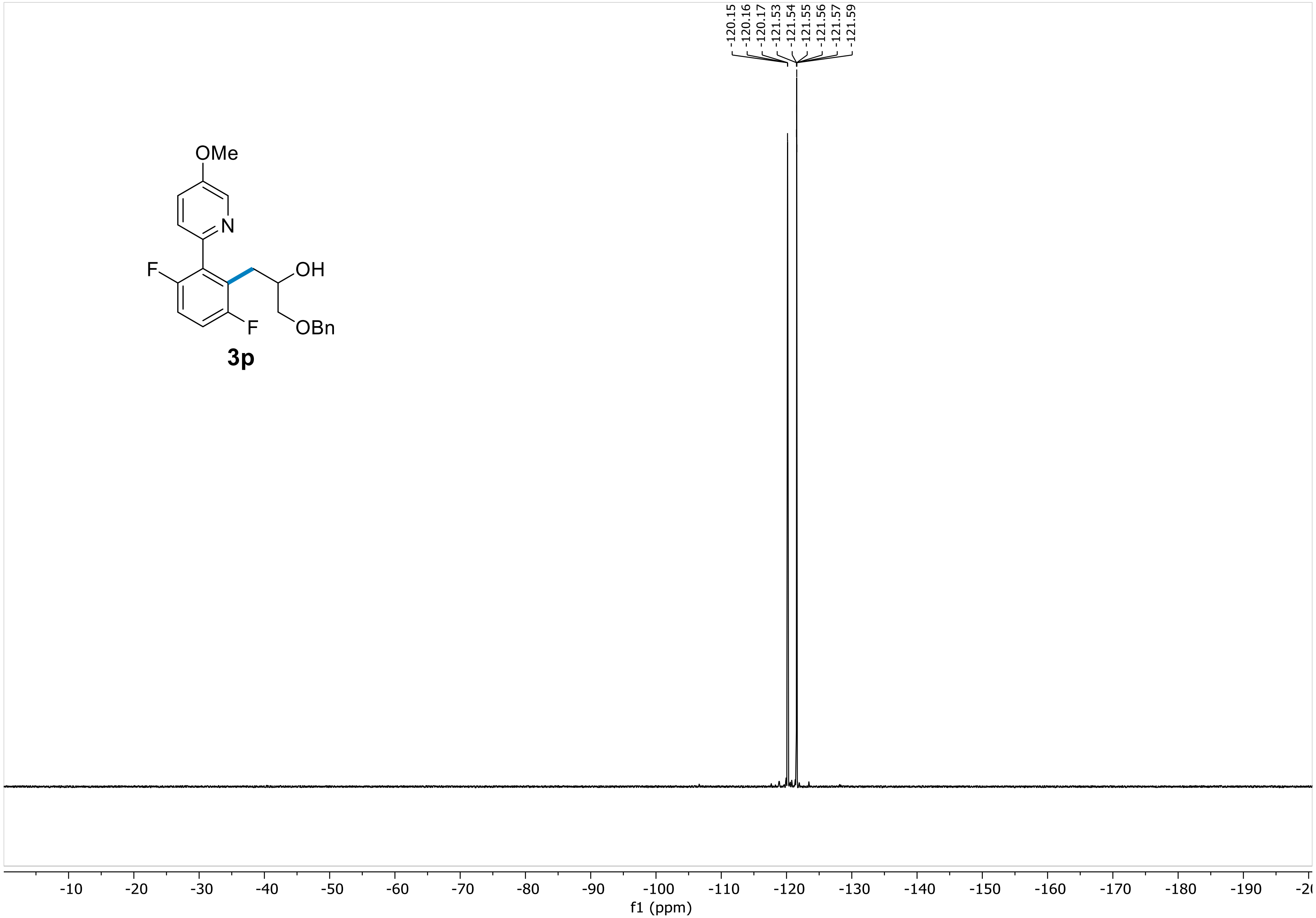

^{19}F NMR spectra (376 MHz, $CDCl_3$) of 1-(benzyloxy)-3-(3,6-difluoro-2-(5-methoxypyridin-2-yl)phenyl)propan-2-ol (**3p**)

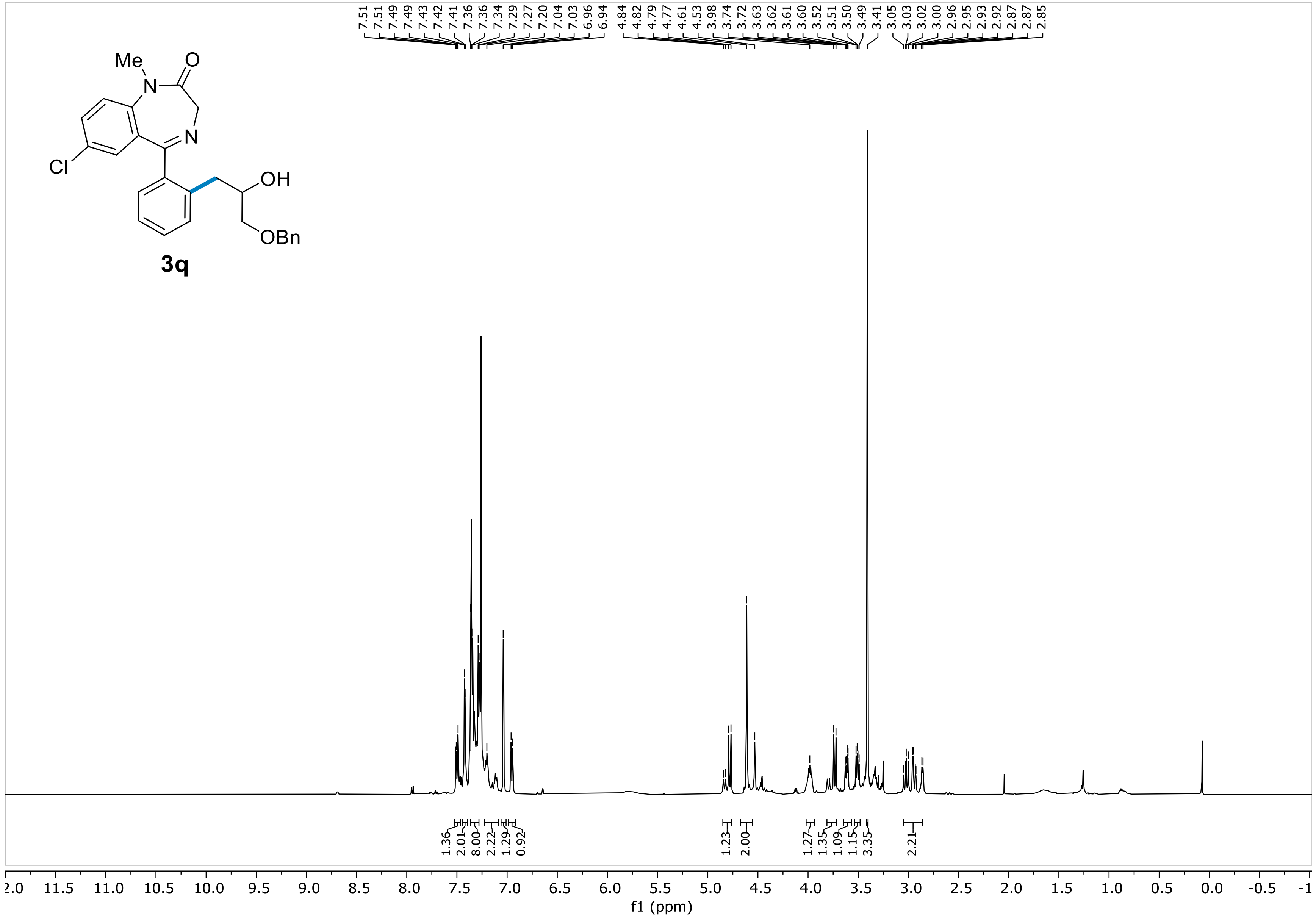

^{1}H NMR spectra (500 MHz, $CDCl_3$) of 5-(2-(3-(benzyloxy)-2-hydroxypropyl)phenyl)-7-chloro-1-methyl-1,3-dihydro-2H-benzo[e][1,4]diazepin-2-one (**3q**)

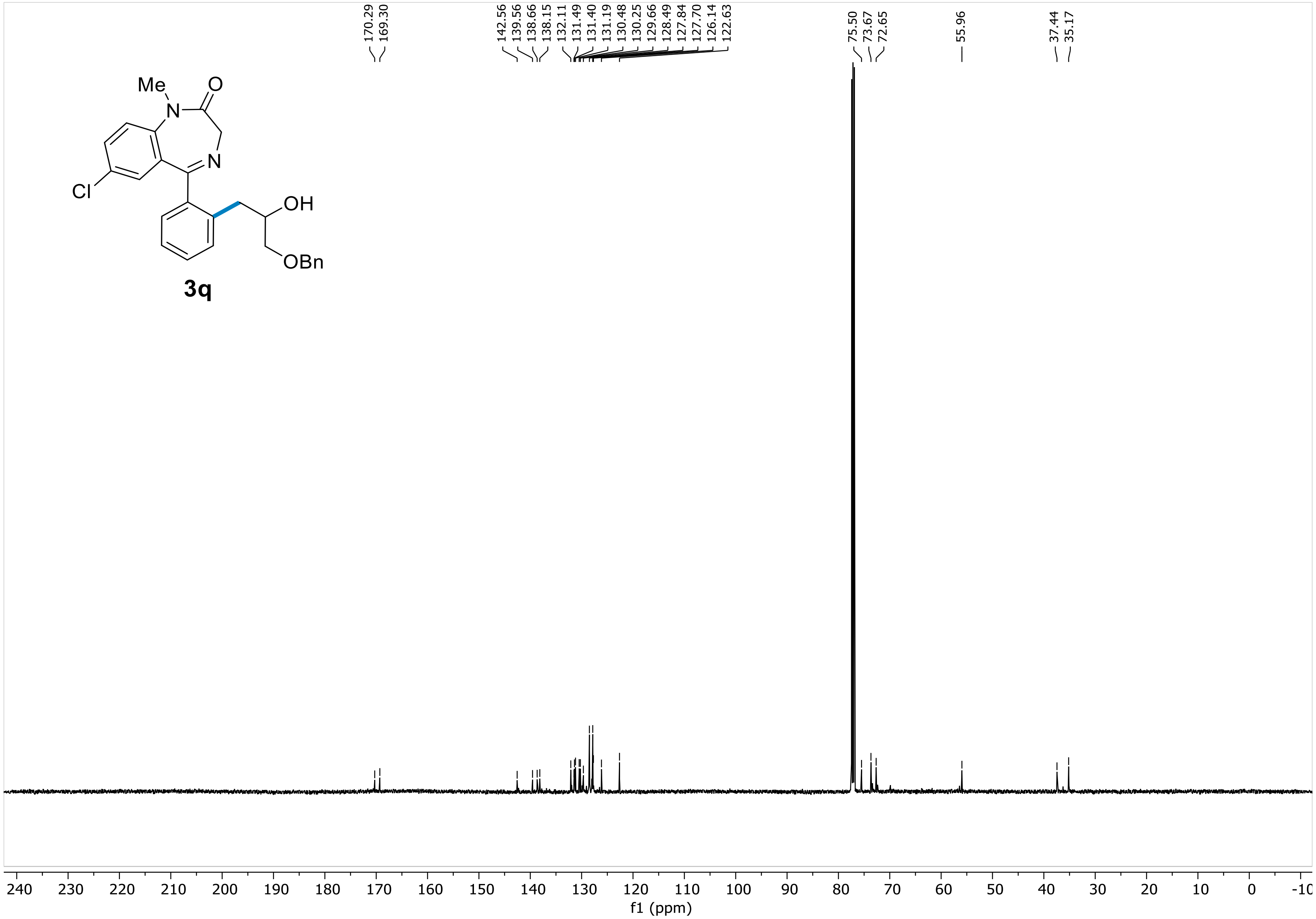

^{13}C NMR spectra (126 MHz, $CDCl_3$) of 5-(2-(3-(benzyloxy)-2-hydroxypropyl)phenyl)-7-chloro-1-methyl-1,3-dihydro-2H-benzo[e][1,4]diazepin-2-one (**3q**)

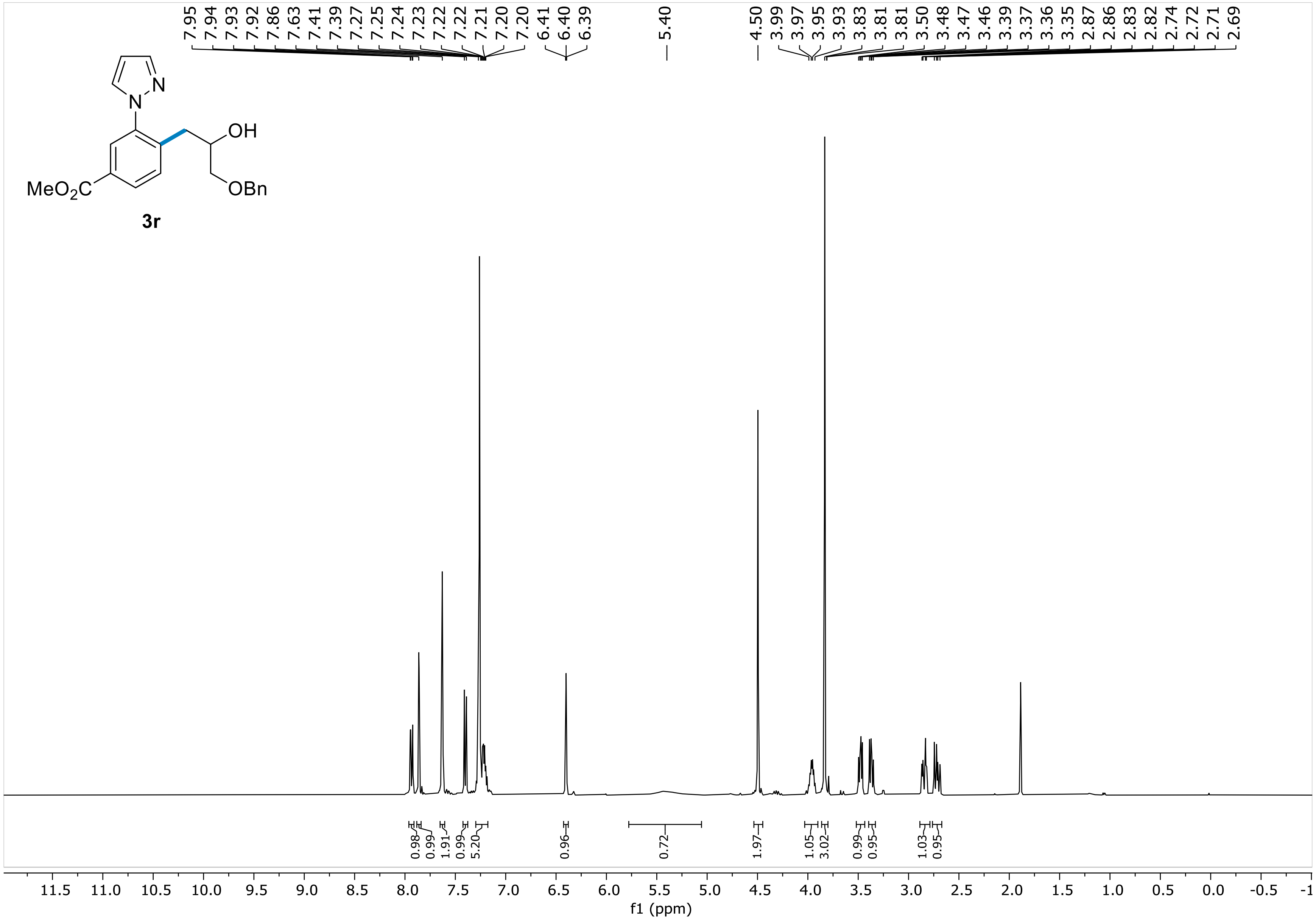

^{1}H NMR spectra (400 MHz, $CDCl_3$) of methyl 4-(3-(benzyloxy)-2-hydroxypropyl)-3-(1H-pyrazol-1-yl)benzoate (**3r**)

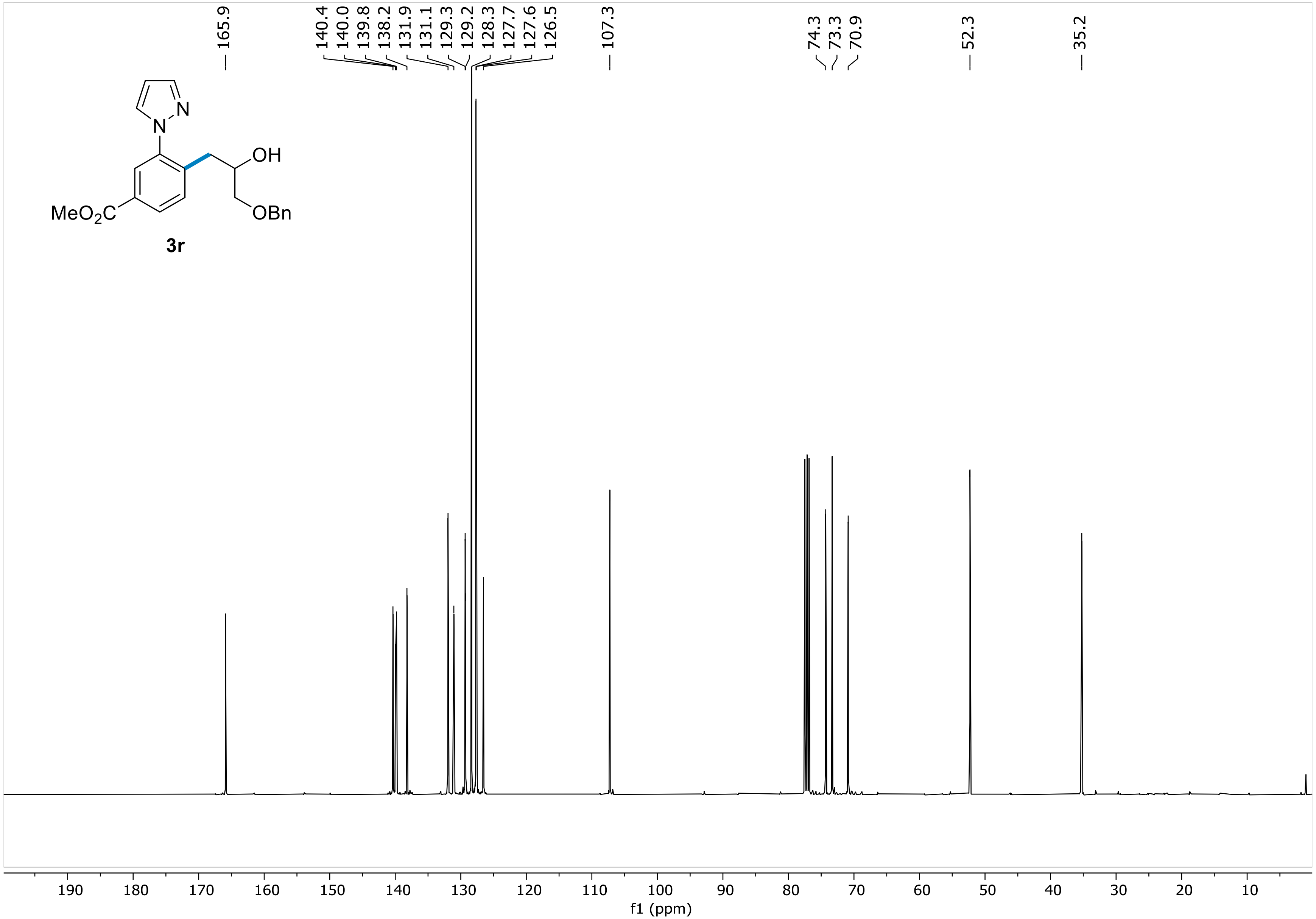

^{13}C NMR spectra (101 MHz, $CDCl_3$) of methyl 4-(3-(benzyloxy)-2-hydroxypropyl)-3-(1H-pyrazol-1-yl)benzoate (**3r**)

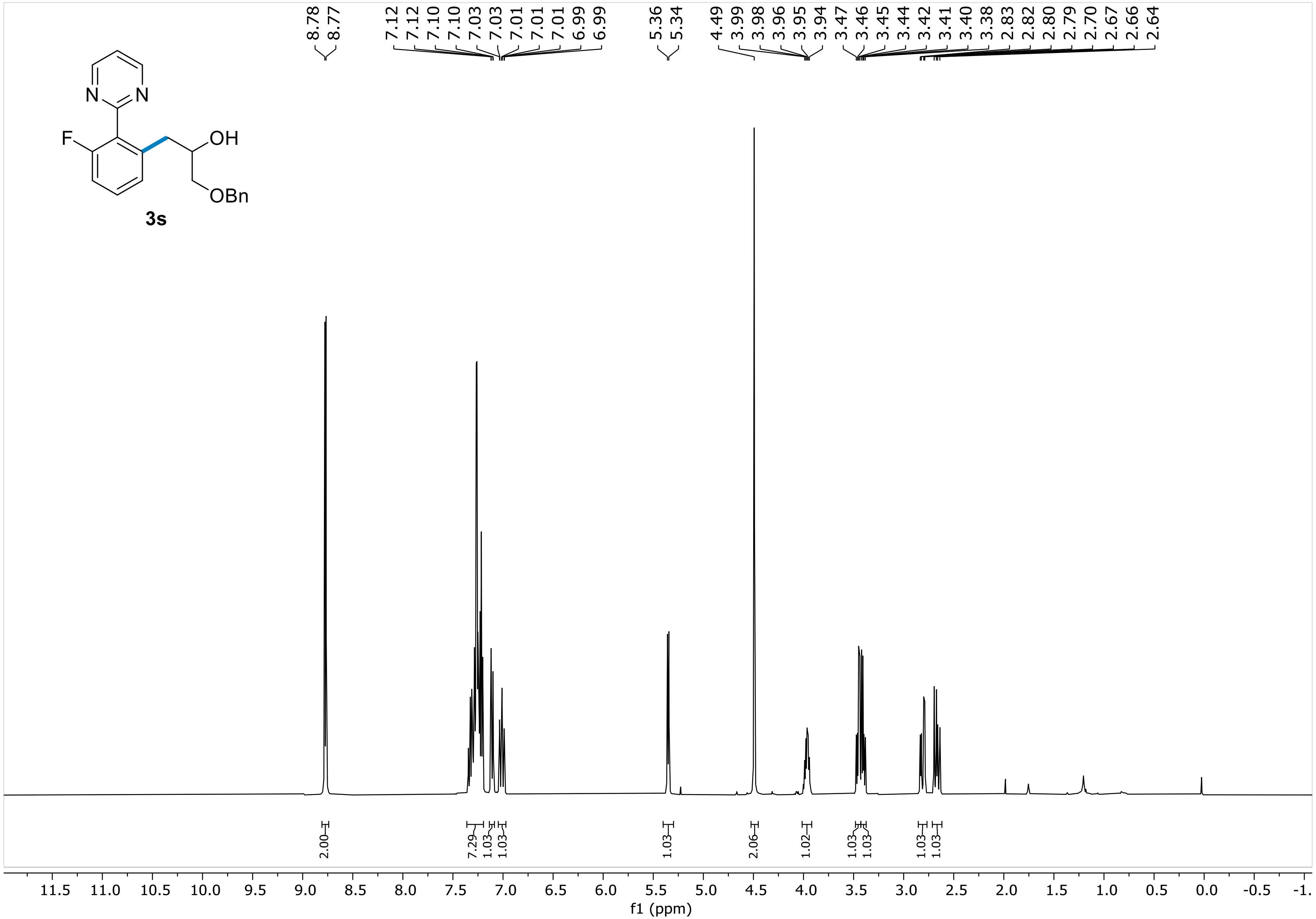

^{1}H NMR spectra (400 MHz, $CDCl_3$) of 1-(benzyloxy)-3-(3-fluoro-2-(pyrimidin-2-yl)phenyl)propan-2-ol (**3s**)

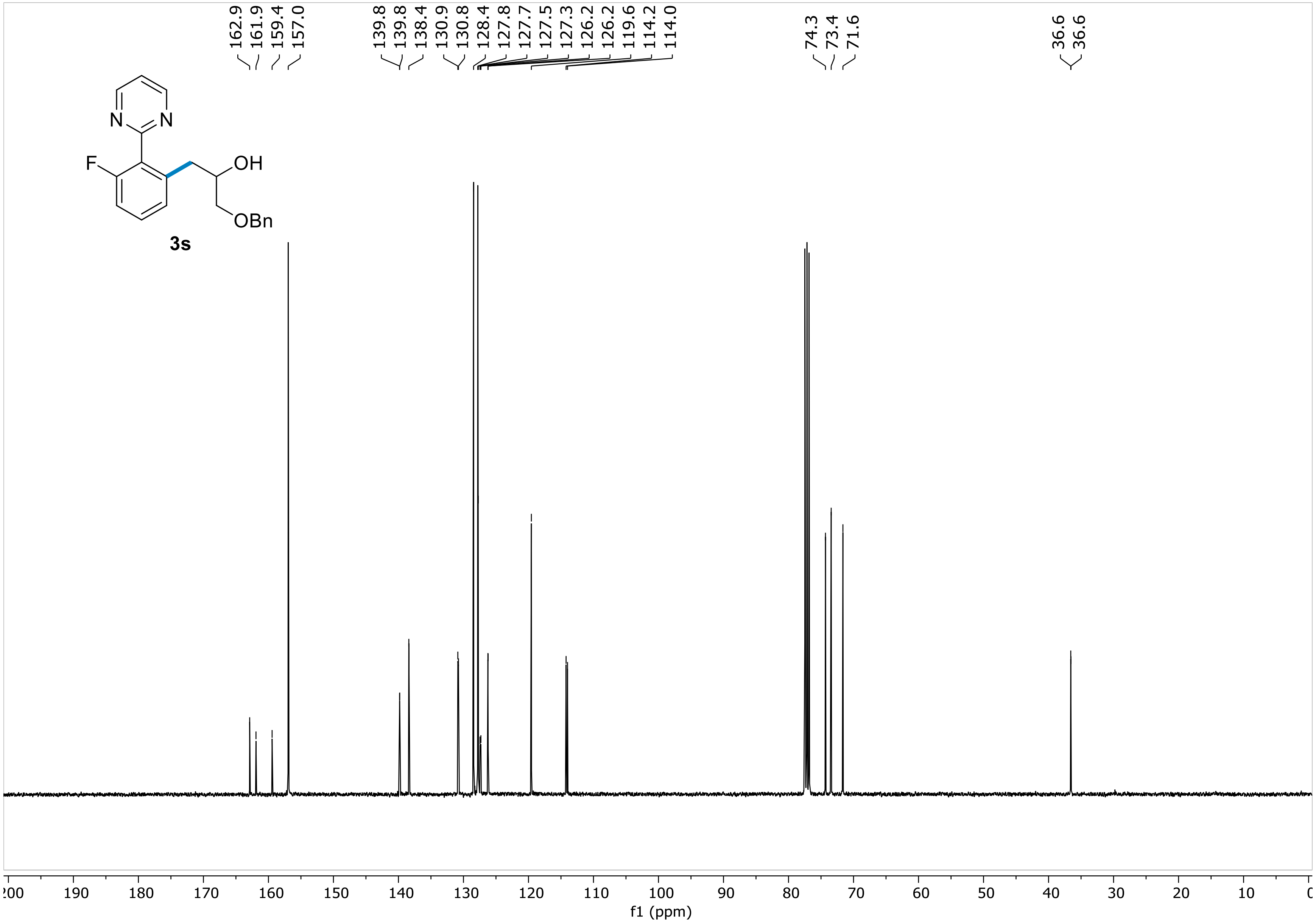

^{13}C NMR spectra (101 MHz, $CDCl_3$) of 1-(benzyloxy)-3-(3-fluoro-2-(pyrimidin-2-yl)phenyl)propan-2-ol (**3s**)

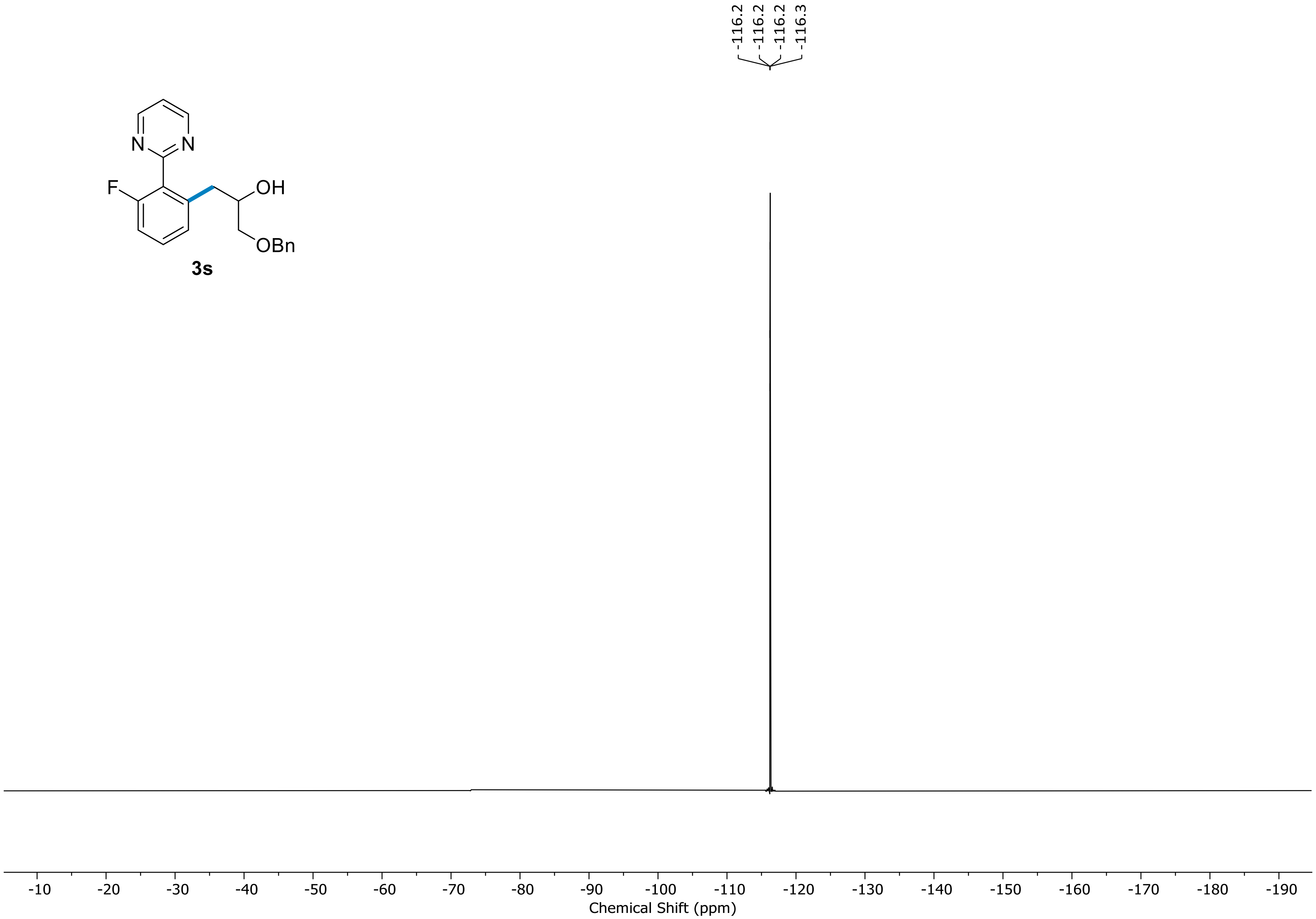

^{19}F NMR spectra (471 MHz, $CDCl_3$) of 1-(benzyloxy)-3-(3-fluoro-2-(pyrimidin-2-yl)phenyl)propan-2-ol (**3s**)

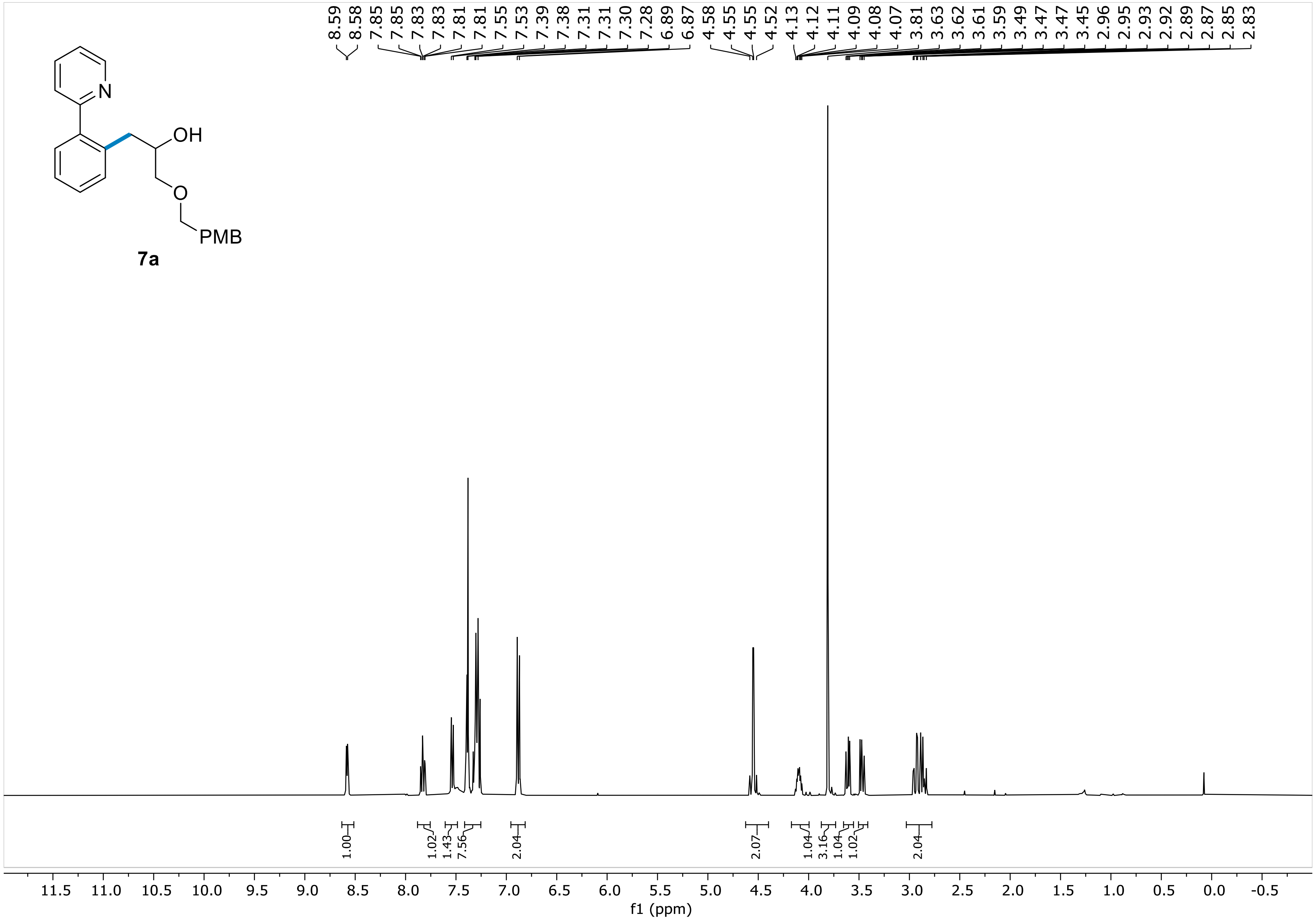

^{1}H NMR spectra (400 MHz, $CDCl_3$) of 1-(4-methoxyphenethoxy)-3-(2-(pyridin-2-yl)phenyl)propan-2-ol (**7a**)

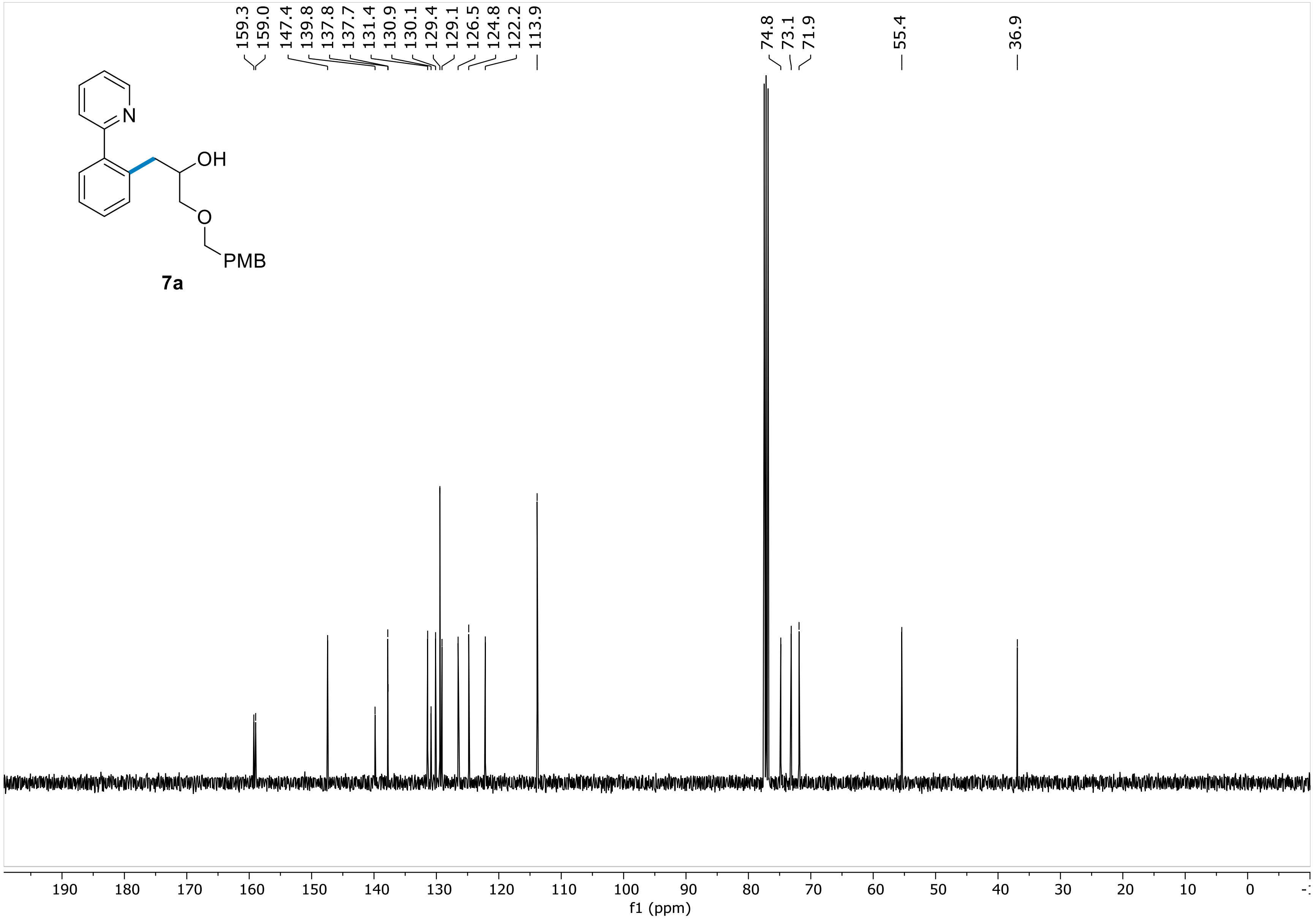

^{13}C NMR spectra (101 MHz, $CDCl_3$) of 1-(4-methoxyphenethoxy)-3-(2-(pyridin-2-yl)phenyl)propan-2-ol (**7a**)

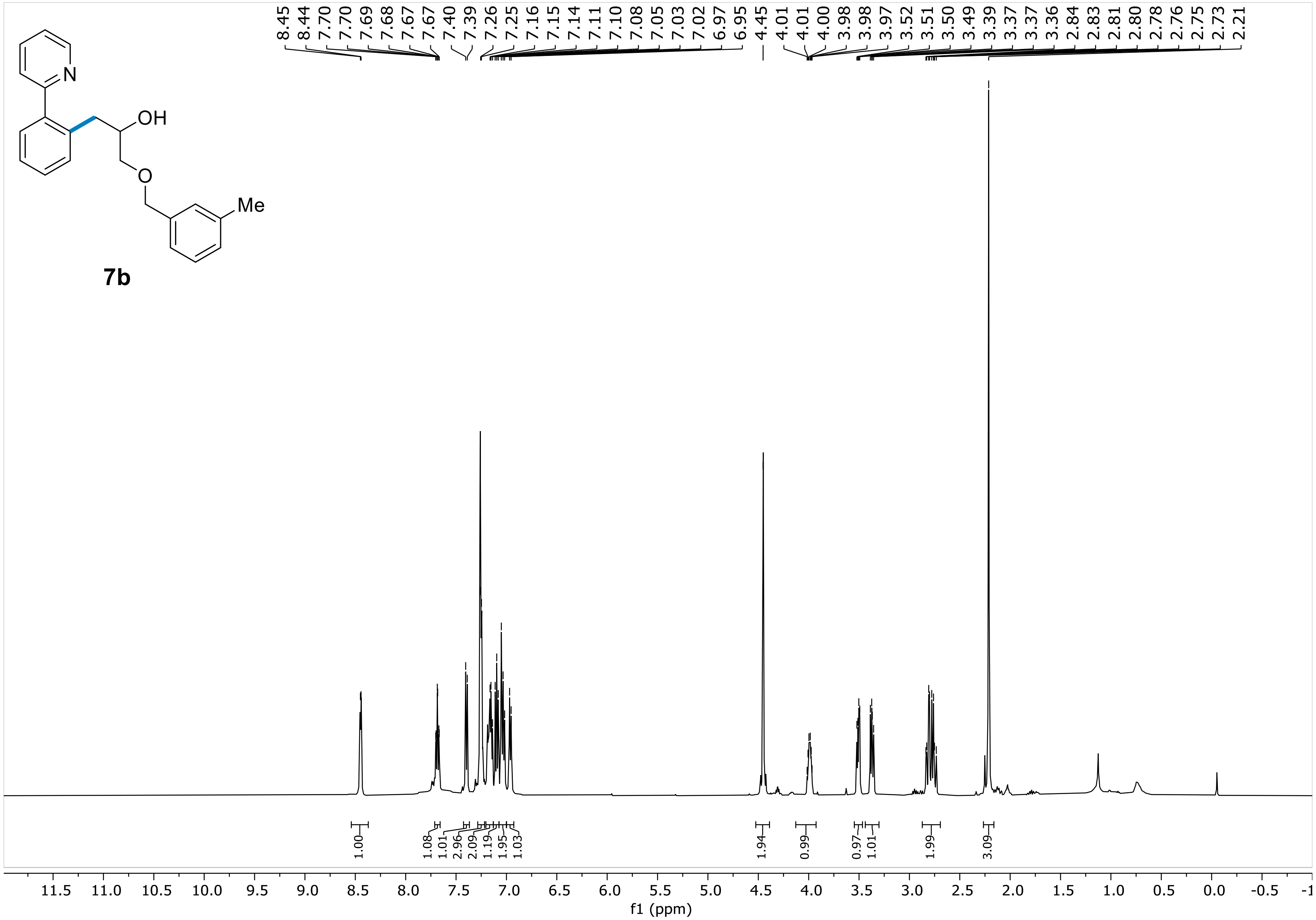

^{1}H NMR spectra (500 MHz, $CDCl_3$) of 1-((3-methylbenzyl)oxy)-3-(2-(pyridin-2-yl)phenyl)propan-2-ol (**7b**)

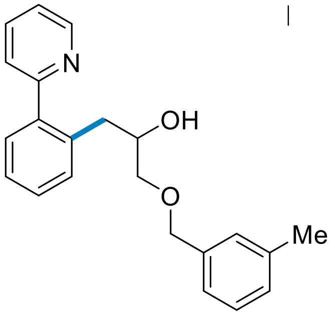

7b

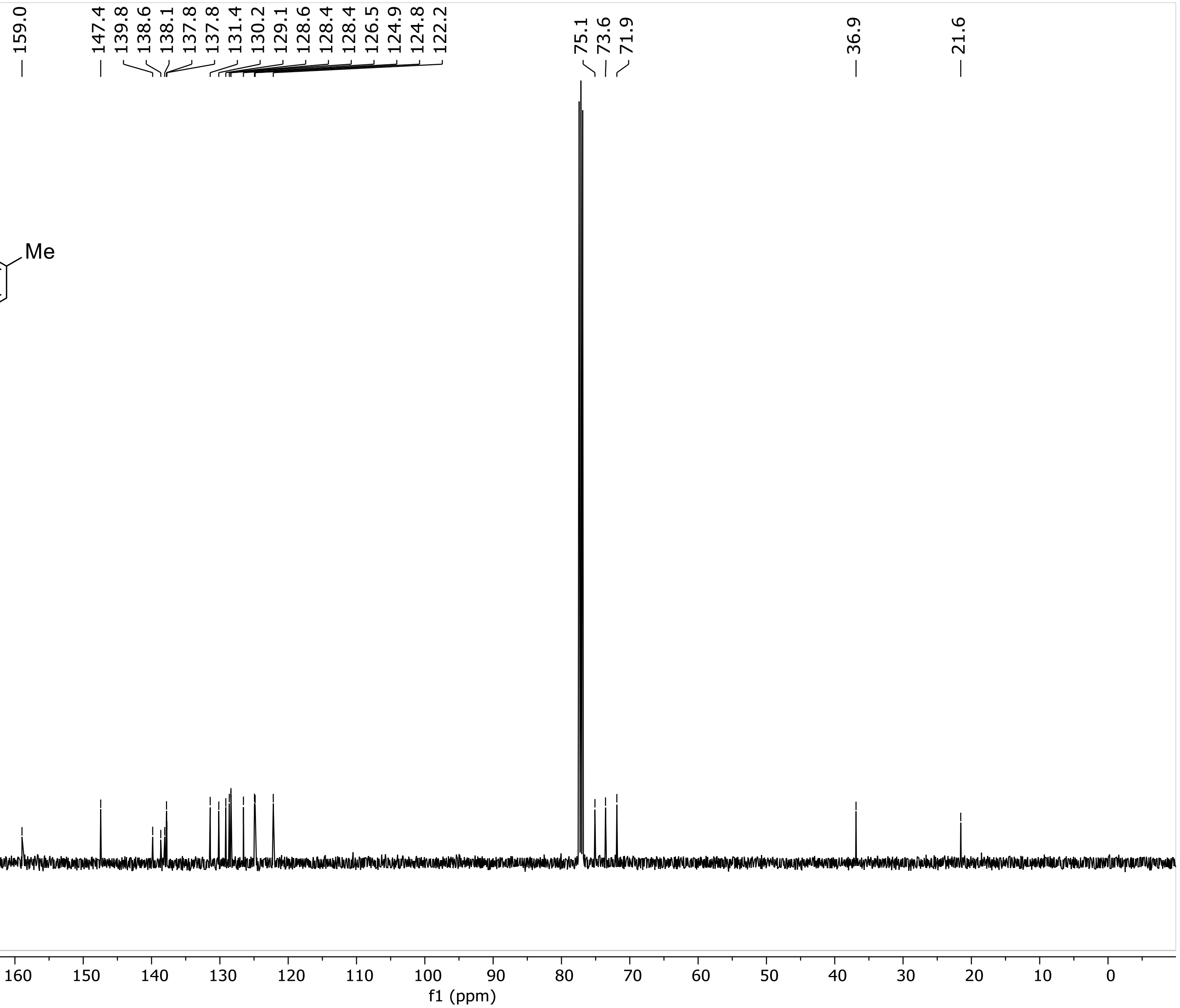

^{13}C NMR spectra (126 MHz, $CDCl_3$) of 1-((3-methylbenzyl)oxy)-3-(2-(pyridin-2-yl)phenyl)propan-2-ol (**7b**)

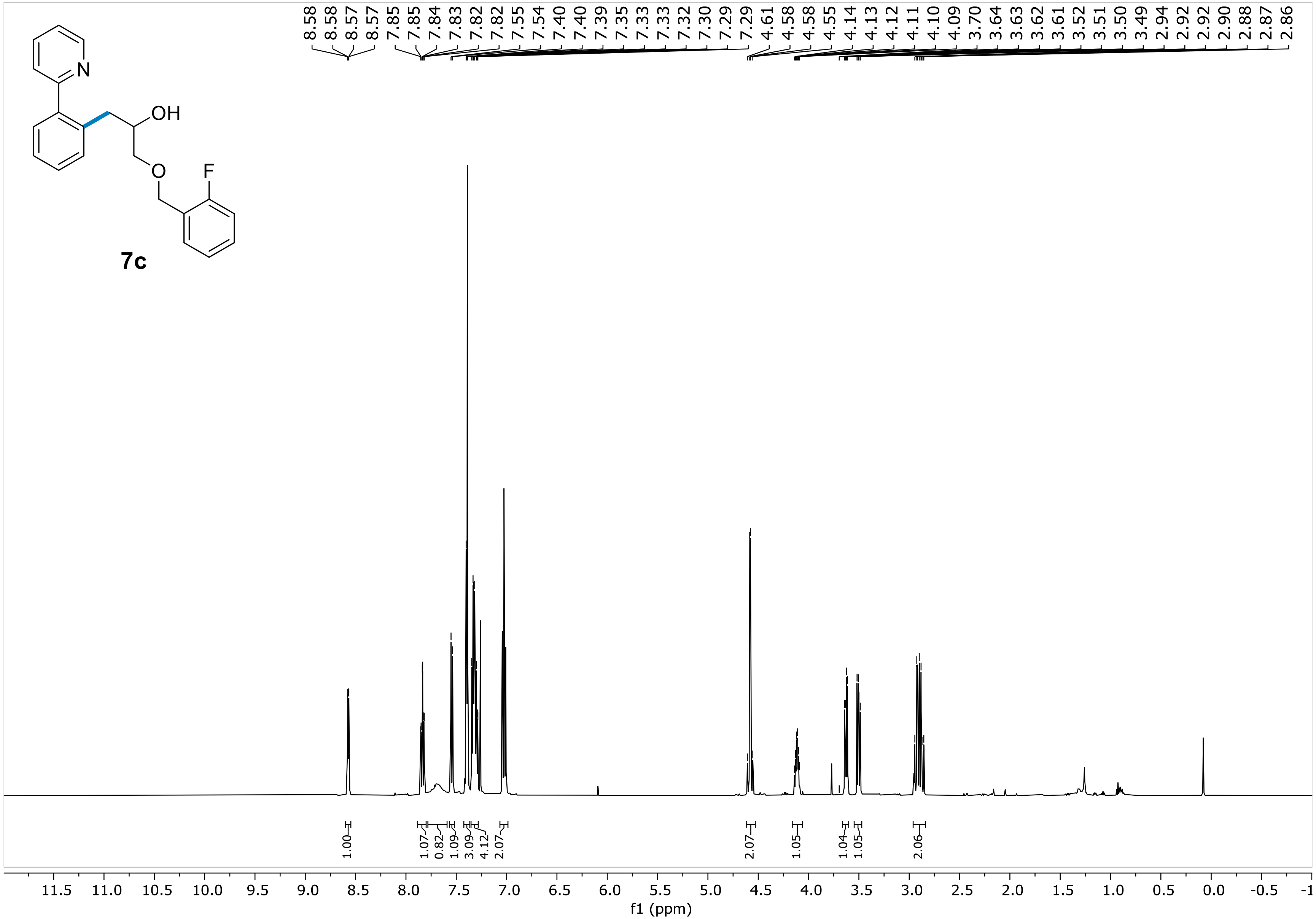

^{1}H NMR spectra (500 MHz, $CDCl_3$) of 1-((2-fluorobenzyl)oxy)-3-(2-(pyridin-2-yl)phenyl)propan-2-ol (**7c**)

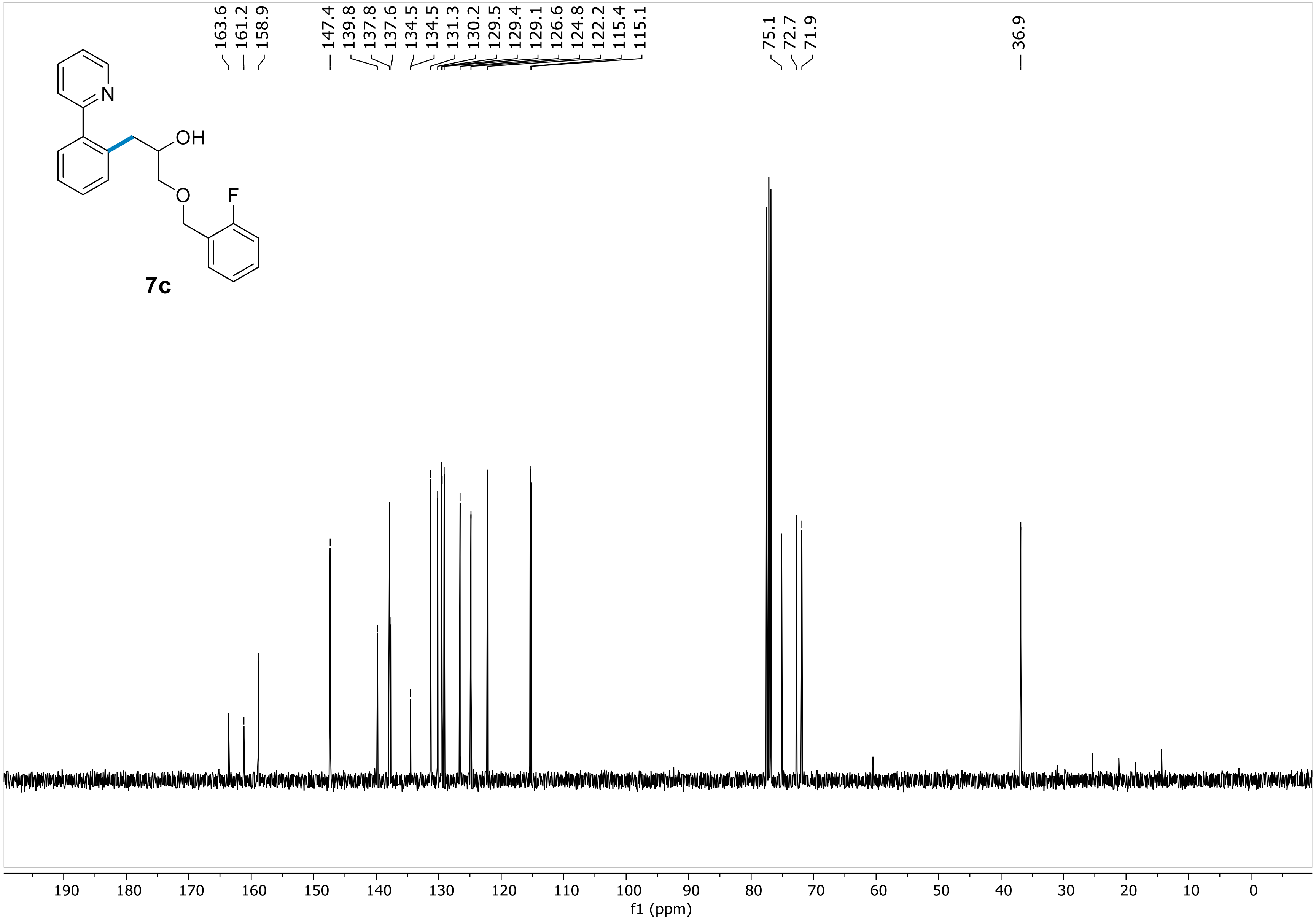

^{13}C NMR spectra (101 MHz, $CDCl_3$) of 1-((2-fluorobenzyl)oxy)-3-(2-(pyridin-2-yl)phenyl)propan-2-ol (**7c**)

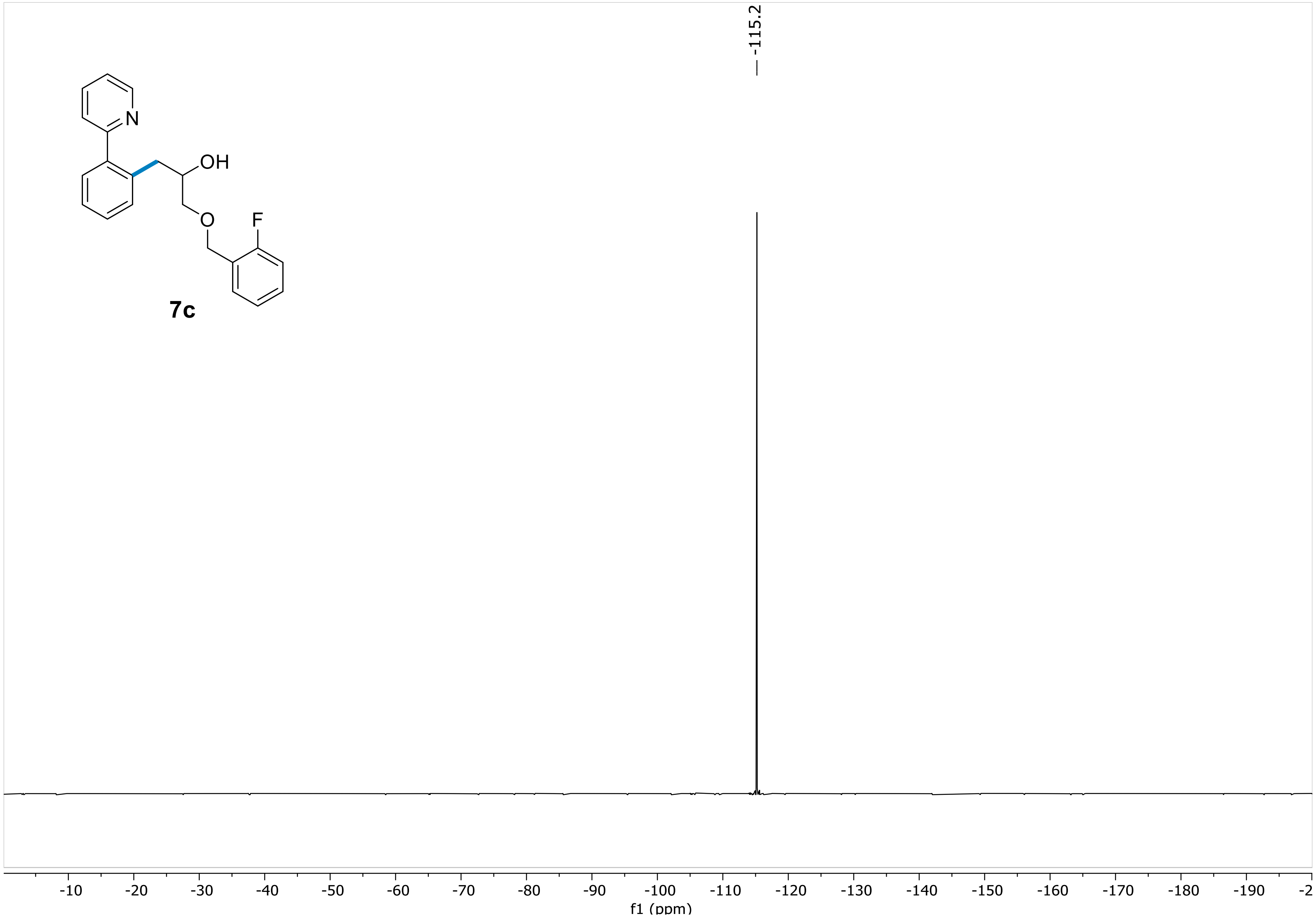

19F NMR spectra (376 MHz, $CDCl_3$) of 1-((2-fluorobenzyl)oxy)-3-(2-(pyridin-2-yl)phenyl)propan-2-ol (**7c**)

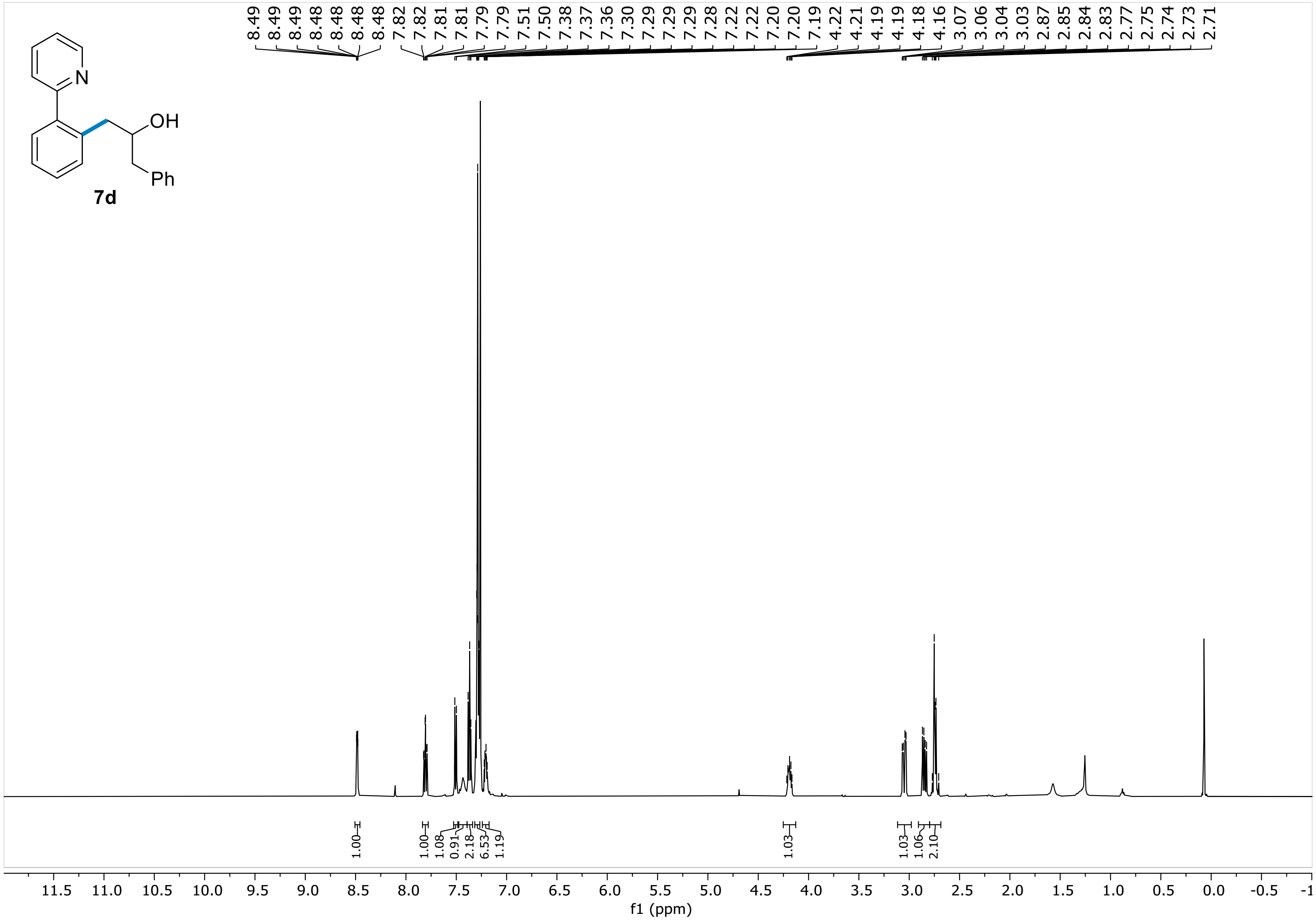

^{1}H NMR spectra (500 MHz, $CDCl_3$) of 1-phenyl-3-(2-(pyridin-2-yl)phenyl)propan-2-ol (**7d**)

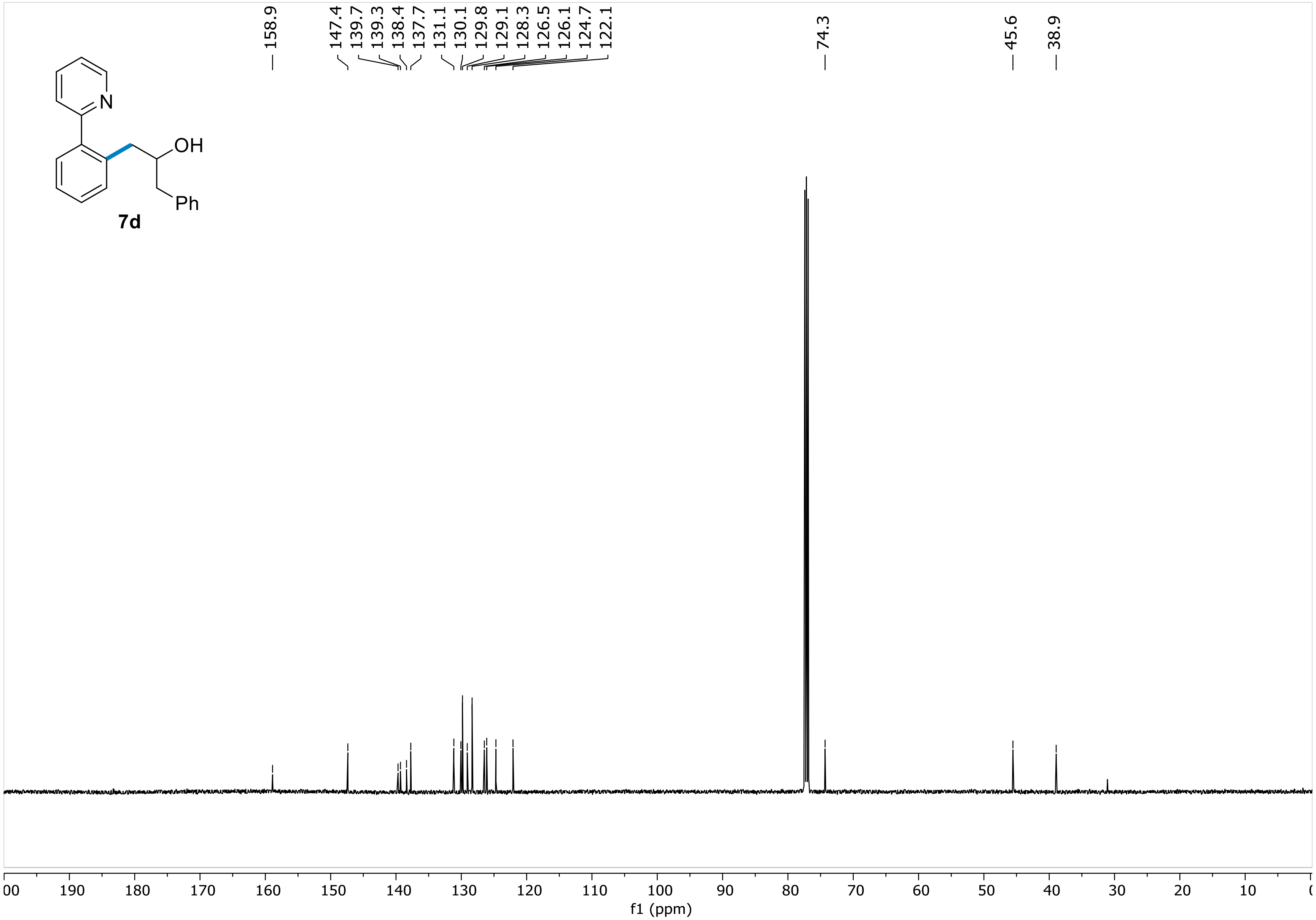

^{13}C NMR spectra (126 MHz, $CDCl_3$) of 1-phenyl-3-(2-(pyridin-2-yl)phenyl)propan-2-ol (**7d**)

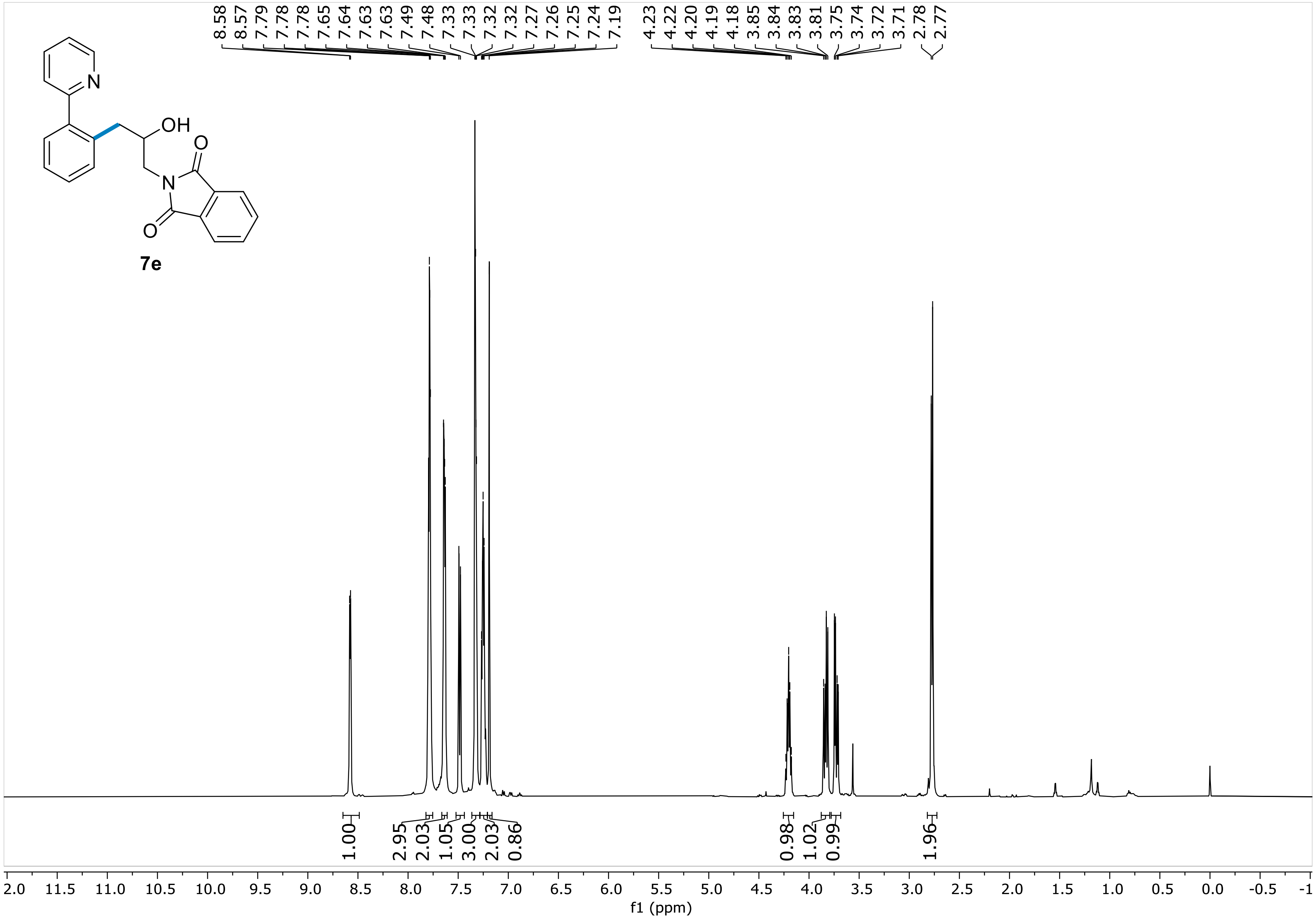

^{1}H NMR spectra (500 MHz, $CDCl_3$) of 2-(2-hydroxy-3-(2-(pyridin-2-yl)phenyl)propyl)isoindoline-1,3-dione (**7e**)

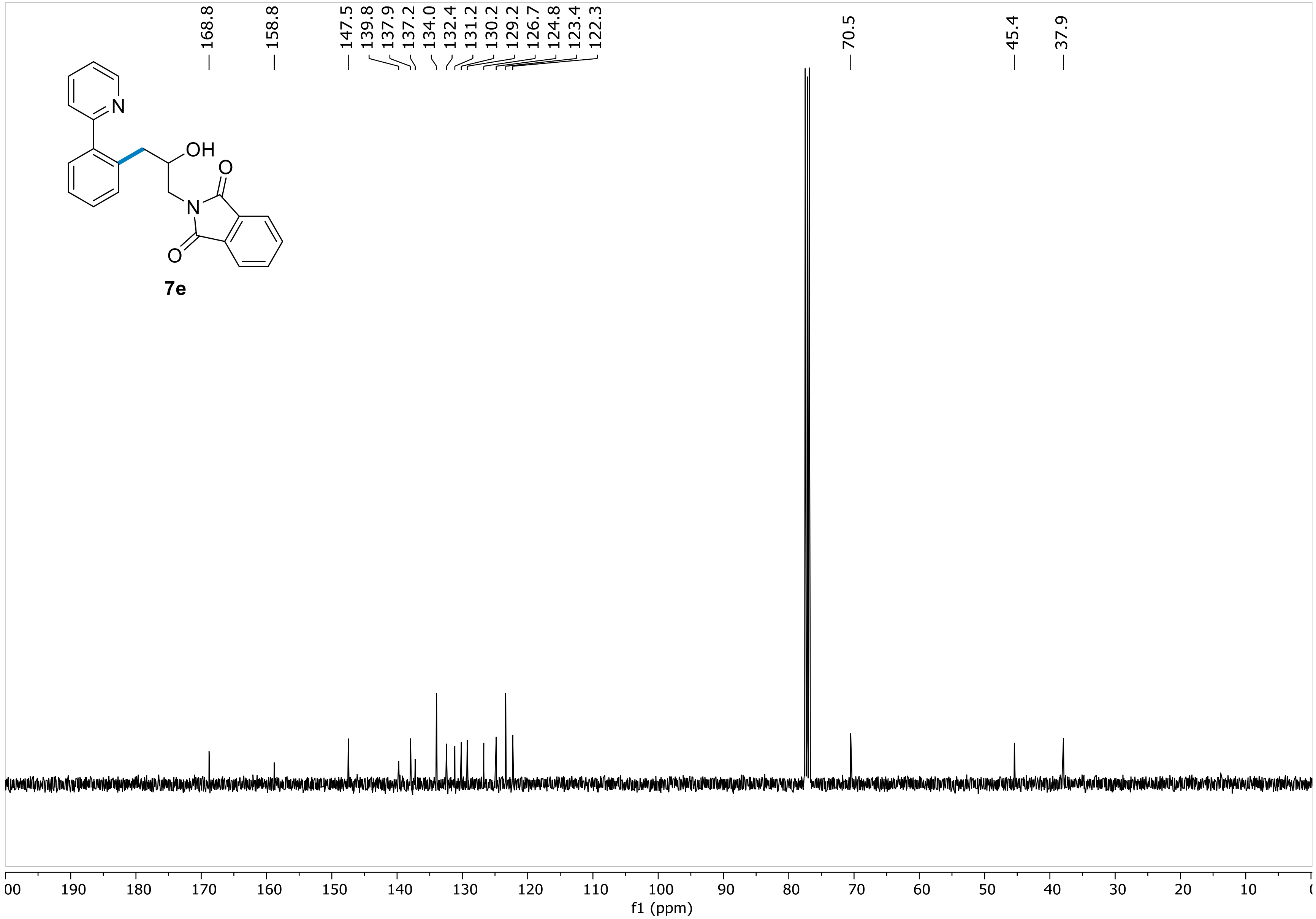

^{13}C NMR spectra (101 MHz, $CDCl_3$) of 2-(2-hydroxy-3-(2-(pyridin-2-yl)phenyl)propyl)isoindoline-1,3-dione (**7e**)

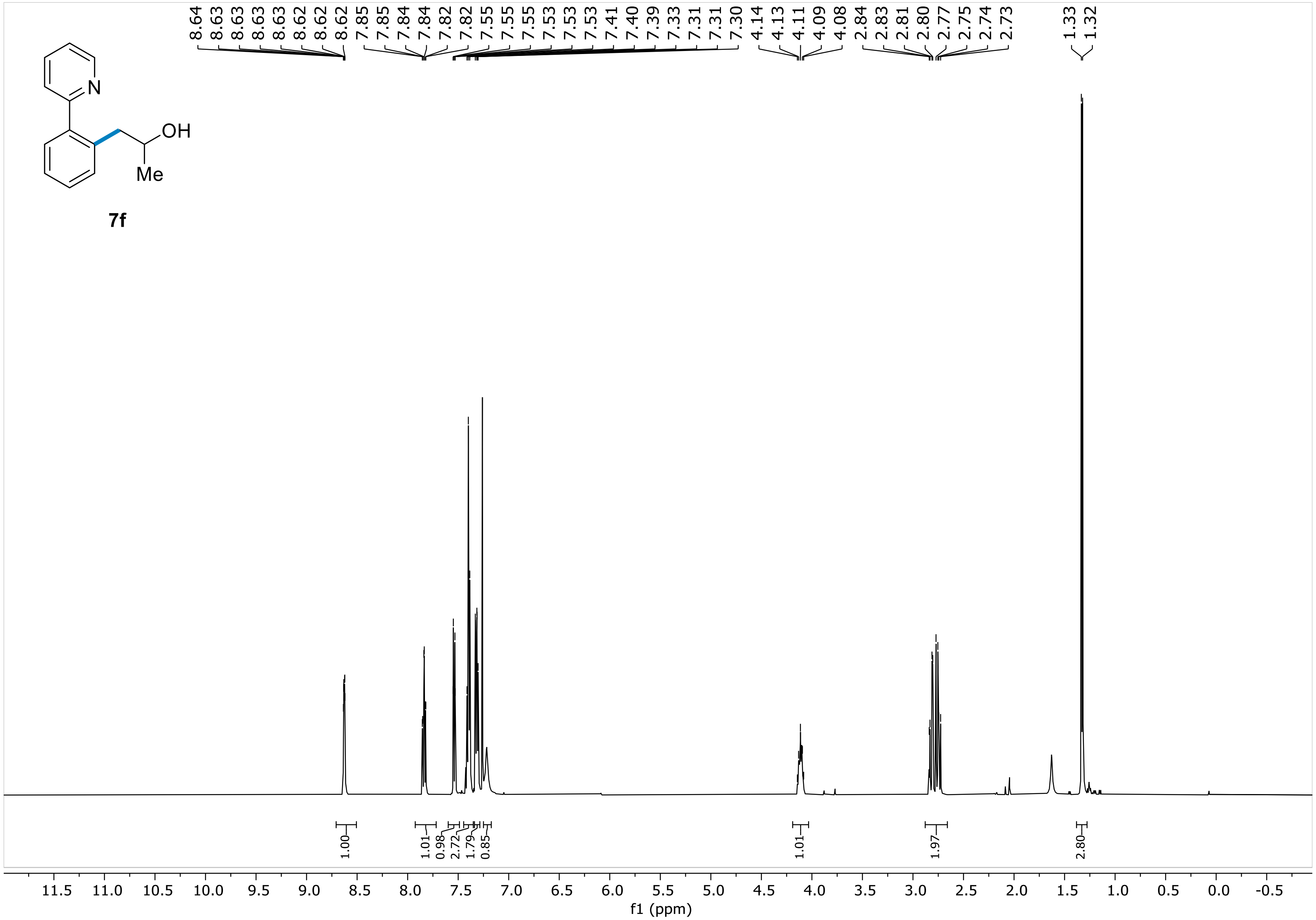

^{1}H NMR spectra (500 MHz, $CDCl_3$) of 1-(2-(pyridin-2-yl)phenyl)propan-2-ol (**7f**)

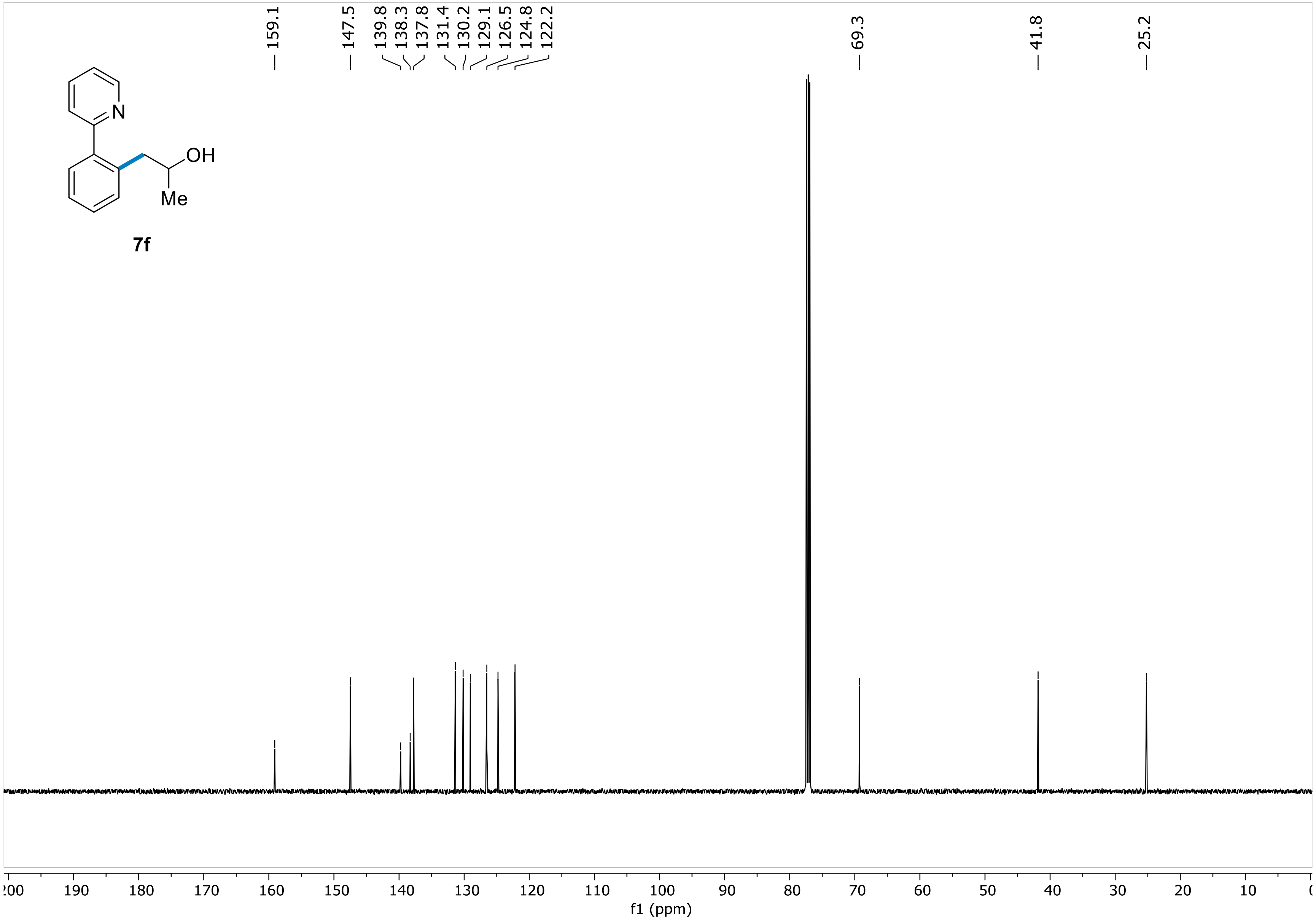

^{13}C NMR spectra (126 MHz, $CDCl_3$) of 1-(2-(pyridin-2-yl)phenyl)propan-2-ol (**7f**)

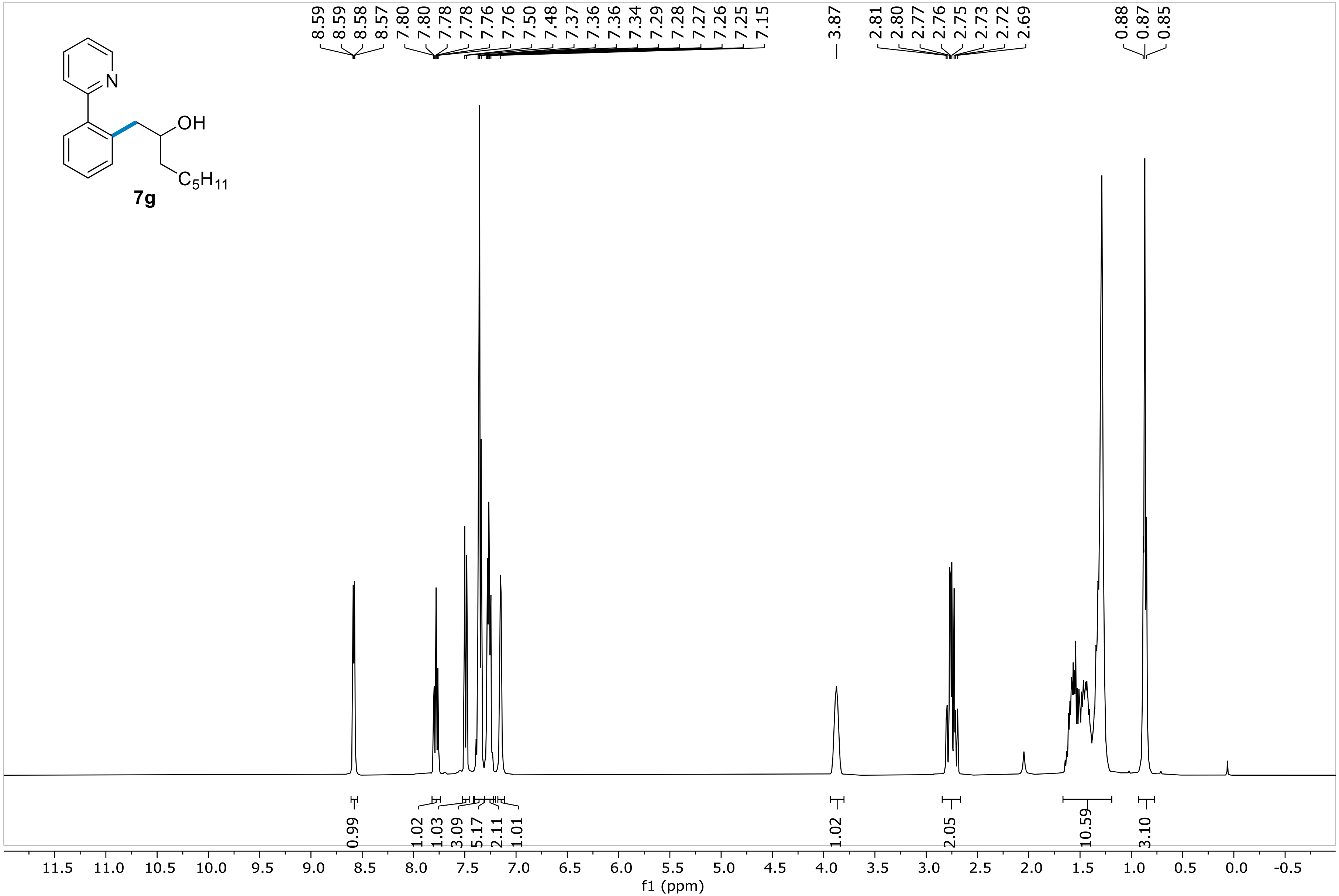

^{1}H NMR spectra (400 MHz, $CDCl_3$) of 1-(2-(pyridin-2-yl)phenyl)octan-2-ol (**7g**)

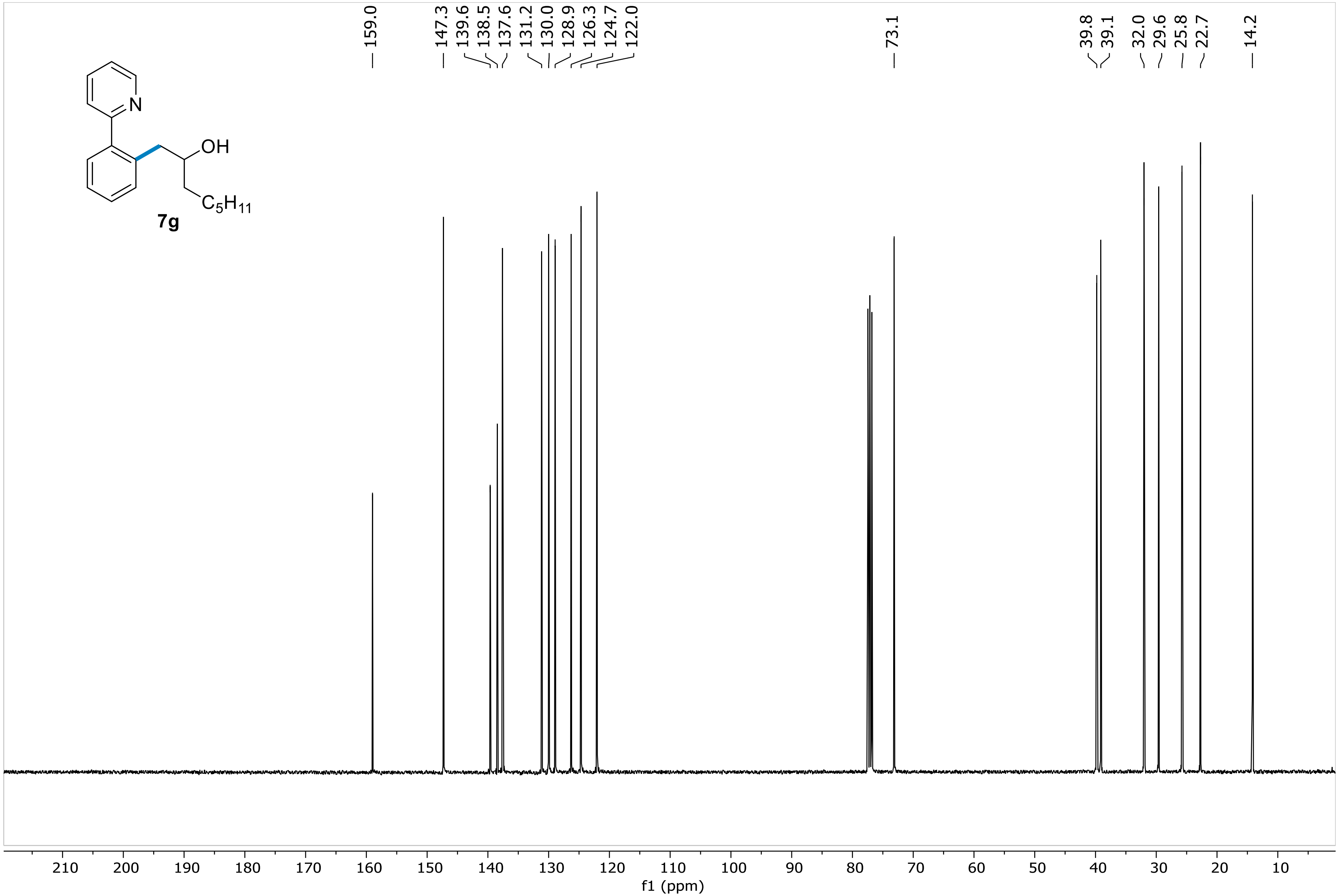

^{13}C NMR spectra (101 MHz, $CDCl_3$) of 1-(2-(pyridin-2-yl)phenyl)octan-2-ol (**7g**)

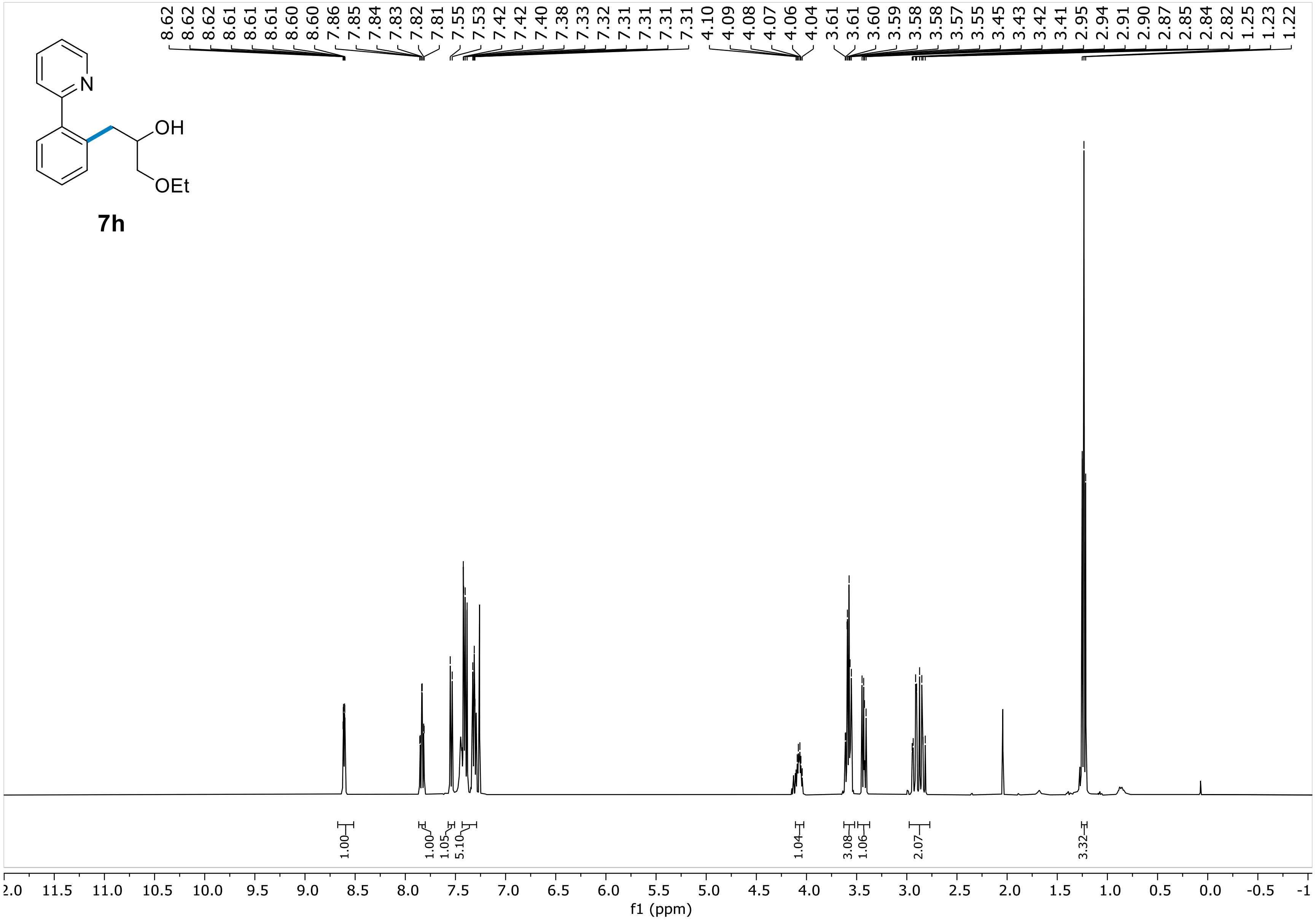

^{1}H NMR spectra (400 MHz, $CDCl_3$) of 1-ethoxy-3-(2-(pyridin-2-yl)phenyl)propan-2-ol (**7h**)

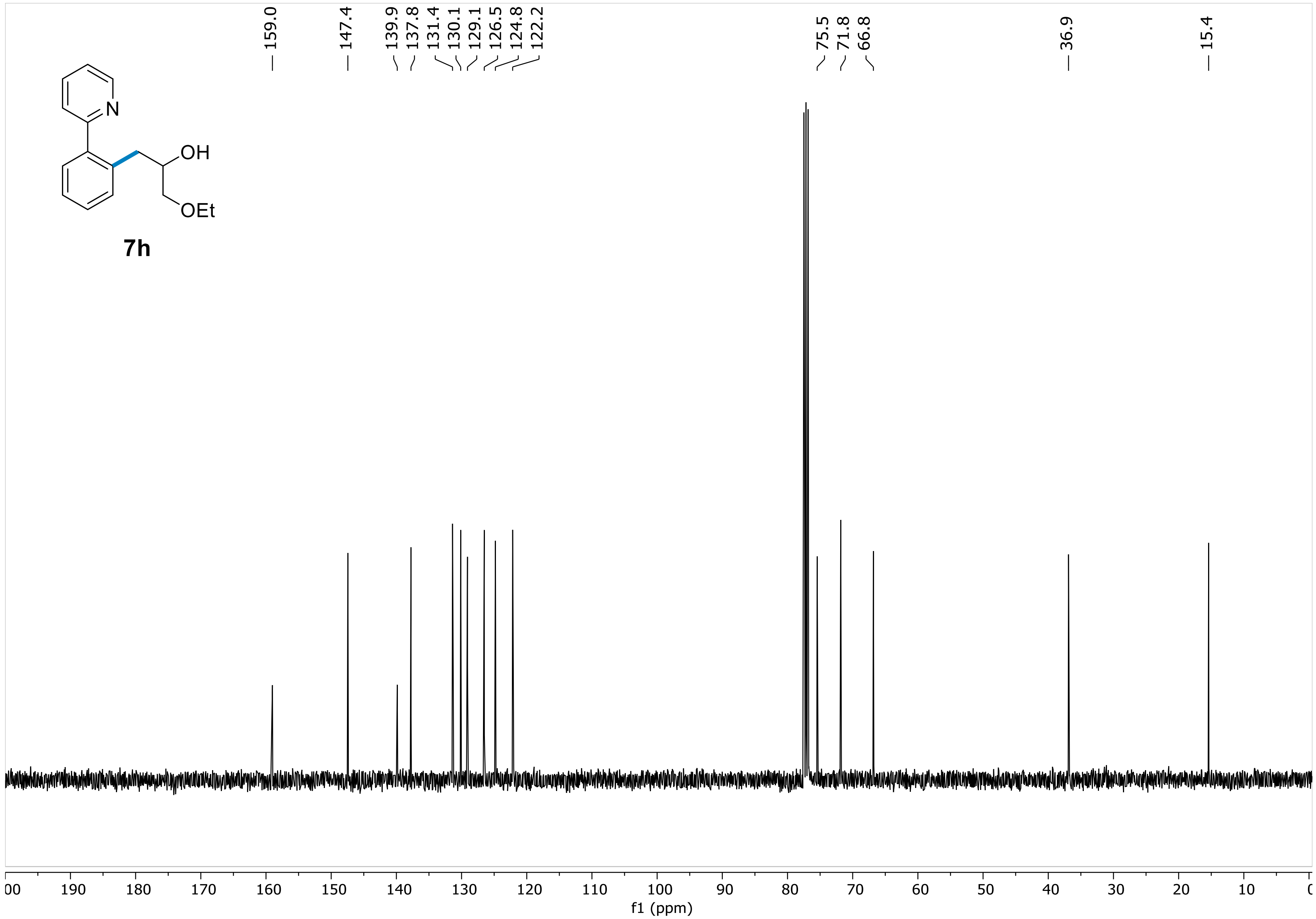

^{13}C NMR spectra (101 MHz, $CDCl_3$) of 1-ethoxy-3-(2-(pyridin-2-yl)phenyl)propan-2-ol (**7h**)

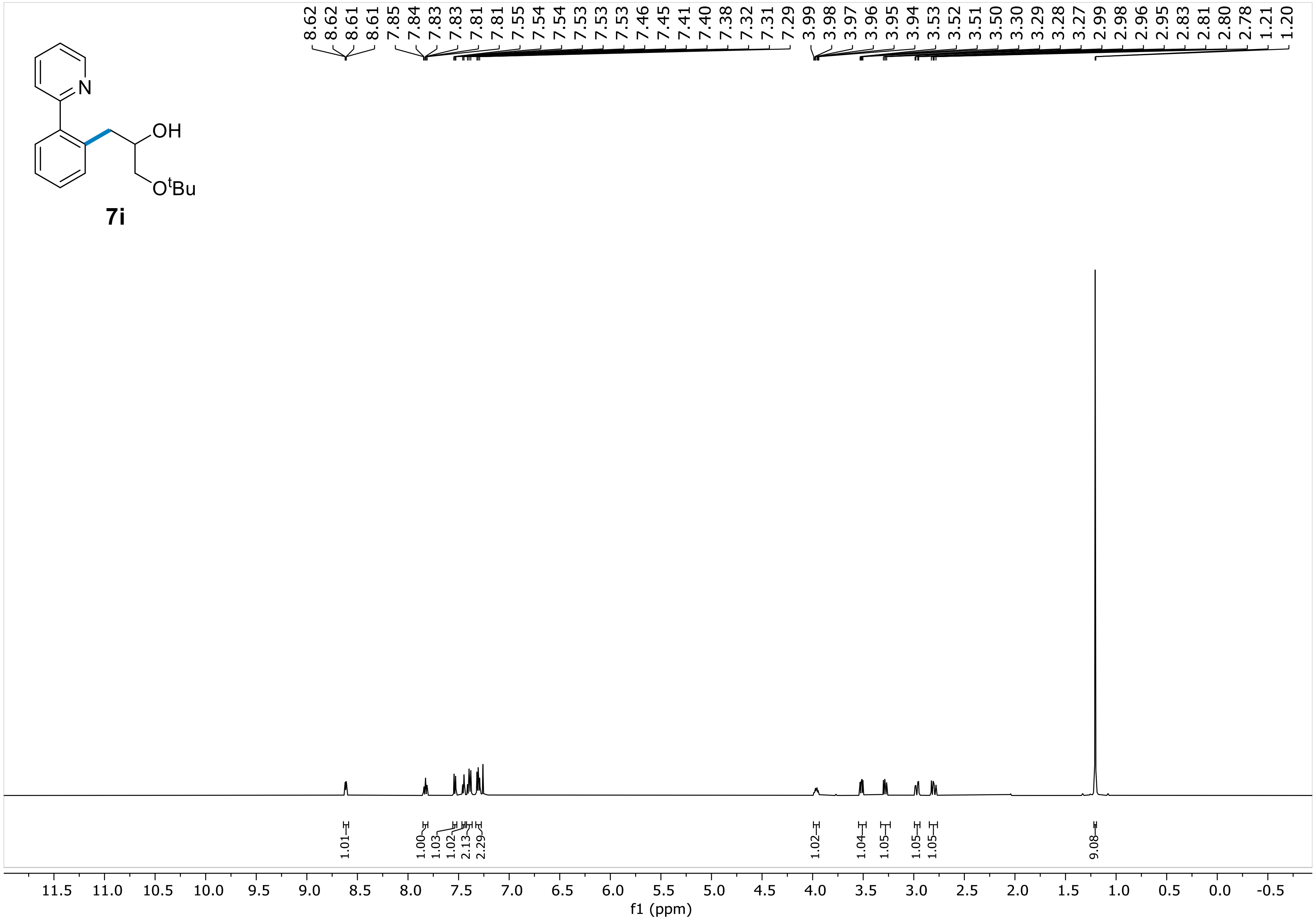

^{1}H NMR spectra (500 MHz, $CDCl_3$) of 1-(tert-butoxy)-3-(2-(pyridin-2-yl)phenyl)propan-2-ol (**7i**)

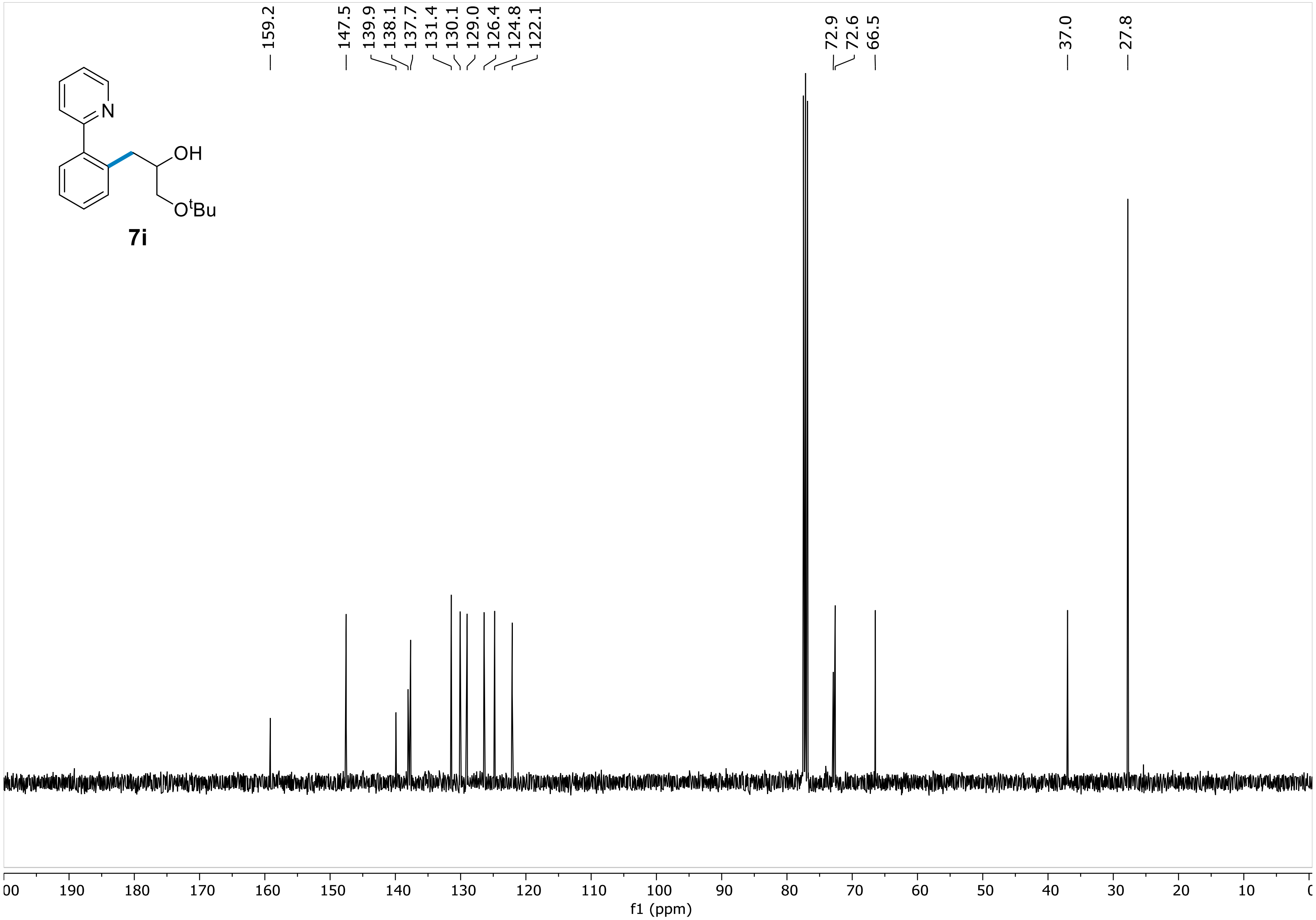

^{13}C NMR spectra (101 MHz, $CDCl_3$) of 1-(tert-butoxy)-3-(2-(pyridin-2-yl)phenyl)propan-2-ol (**7i**)

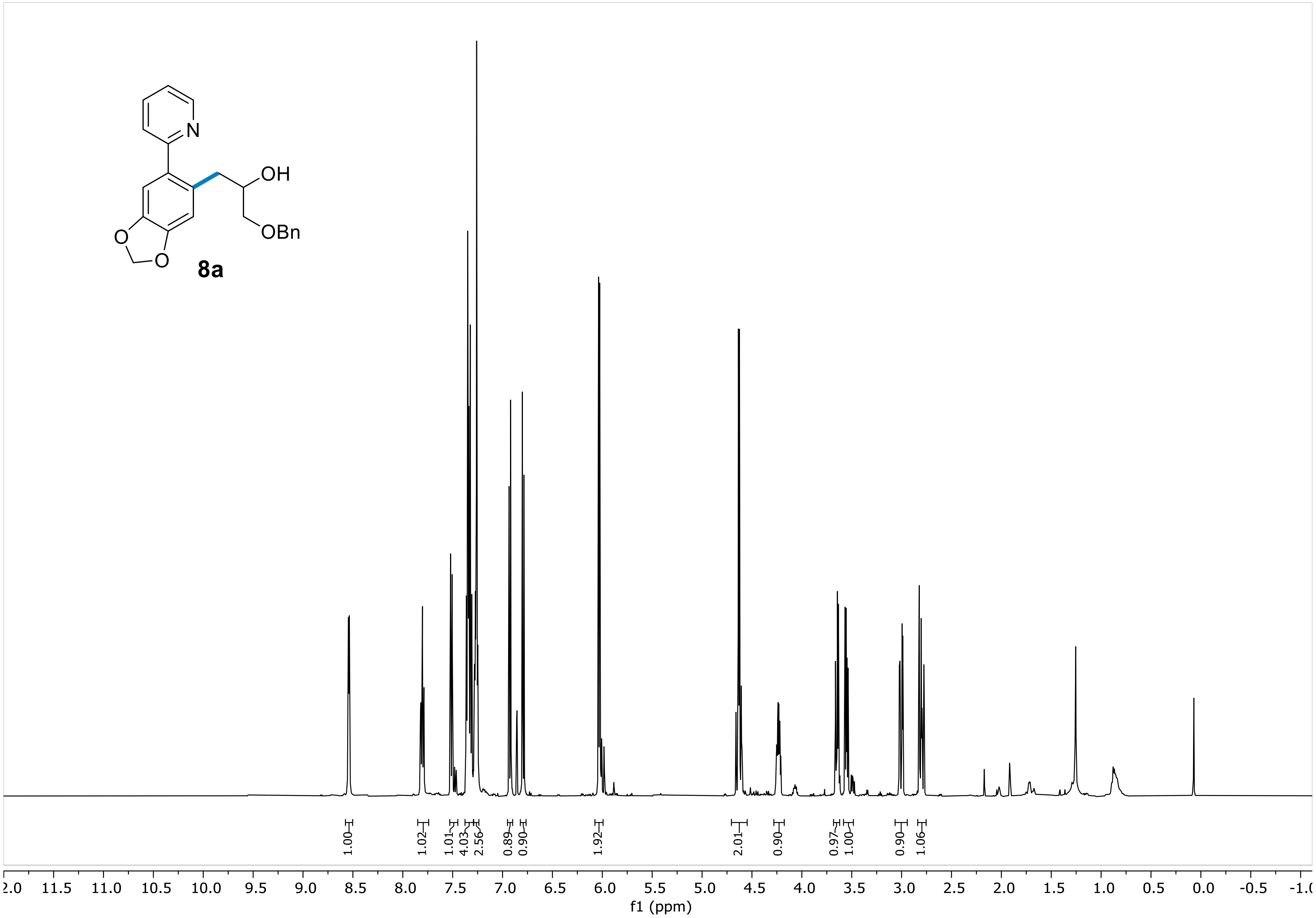

^{1}H NMR spectra (400 MHz, $CDCl_3$) of 1-(benzyloxy)-3-(6-(pyridin-2-yl)benzo[d][1,3]dioxol-5-yl)propan-2-ol (**8a**)

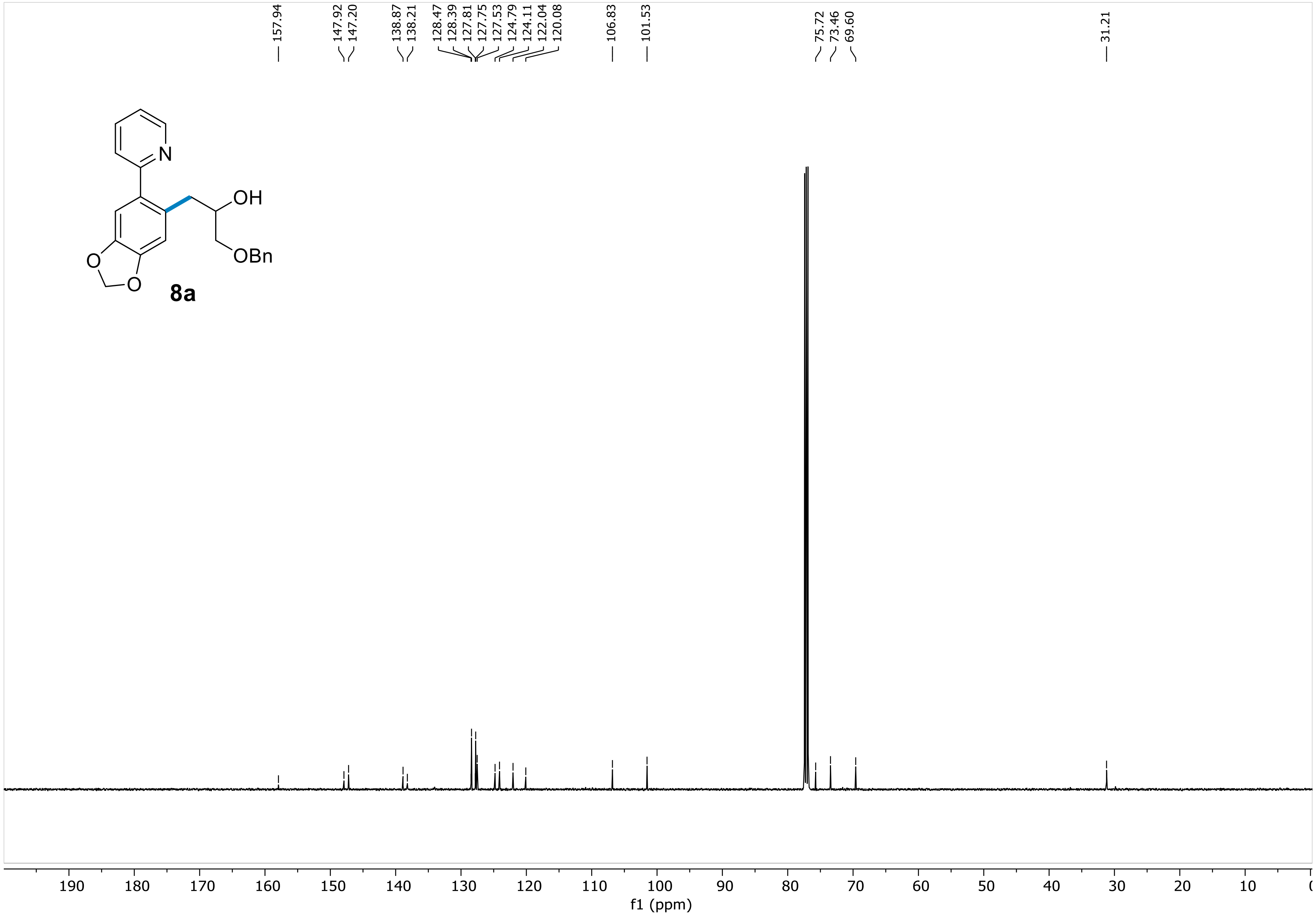

^{13}C NMR spectra (126 MHz, $CDCl_3$) of 1-(benzyloxy)-3-(6-(pyridin-2-yl)benzo[d][1,3]dioxol-5-yl)propan-2-ol (**8a**)

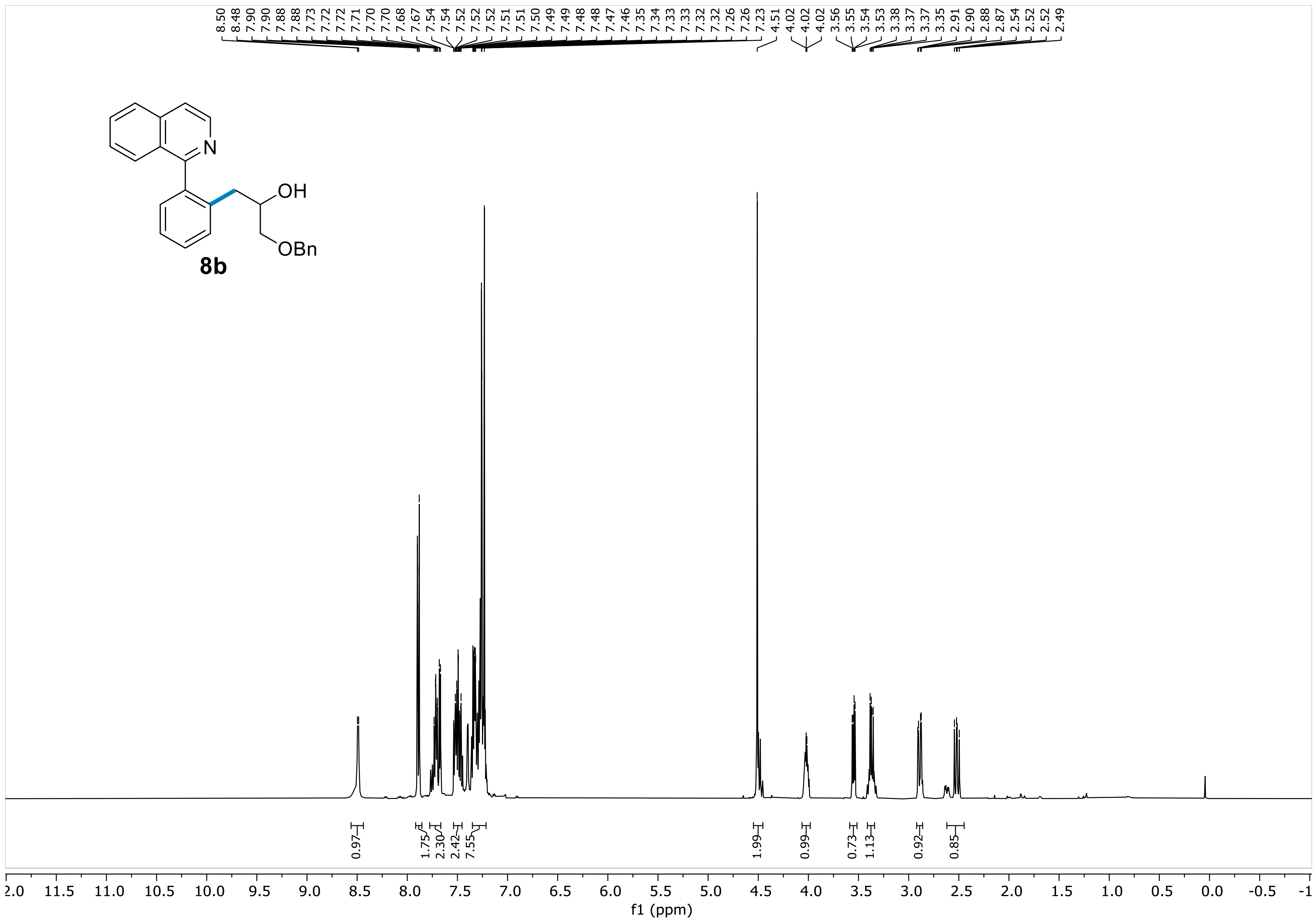

^{1}H NMR spectra (400 MHz, $CDCl_3$) of 1-(benzyloxy)-3-(2-(isoquinolin-1-yl)phenyl)propan-2-ol (**8b**)

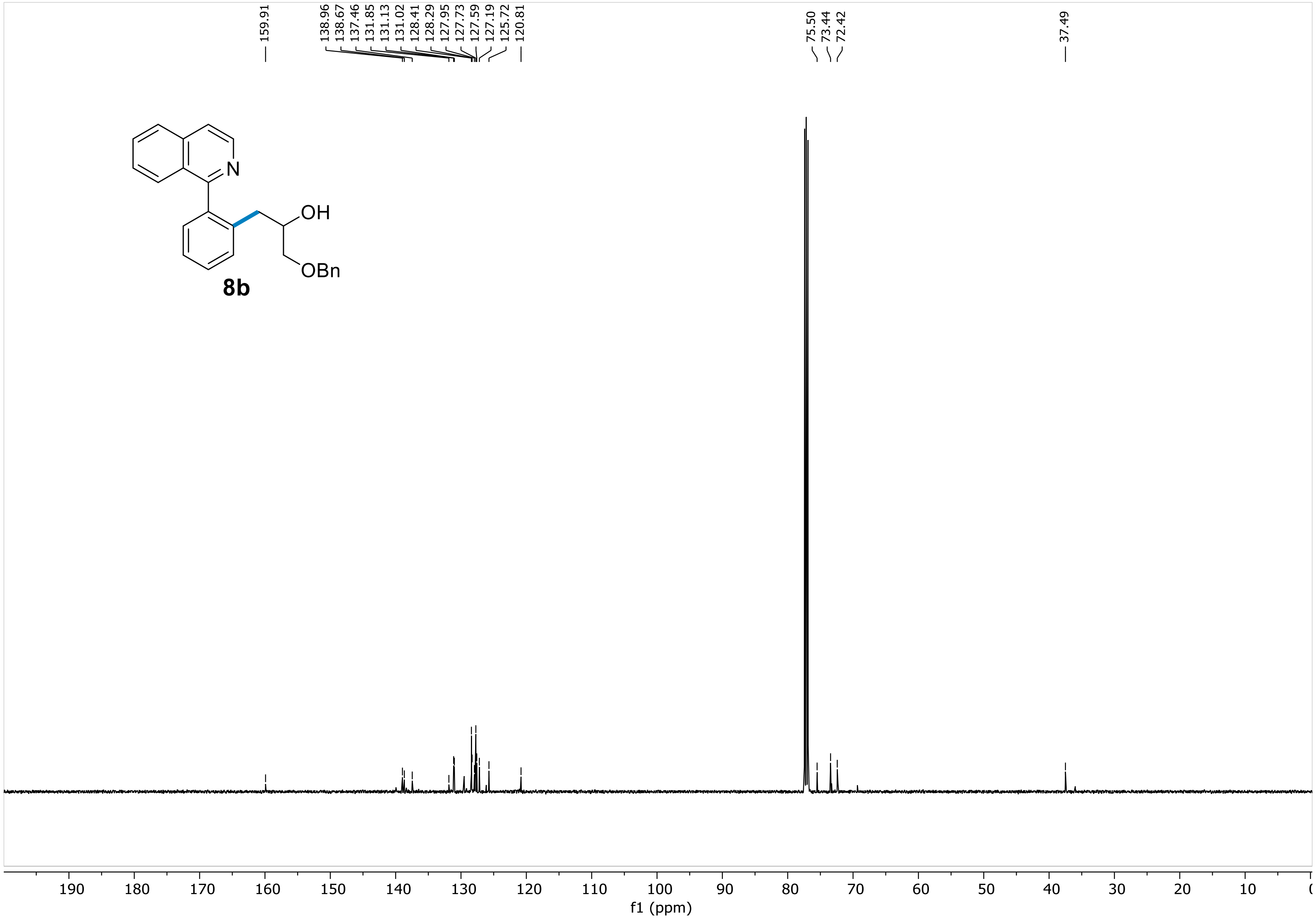

^{13}C NMR spectra (126 MHz, $CDCl_3$) of 1-(benzyloxy)-3-(2-(isoquinolin-1-yl)phenyl)propan-2-ol (**8b**)

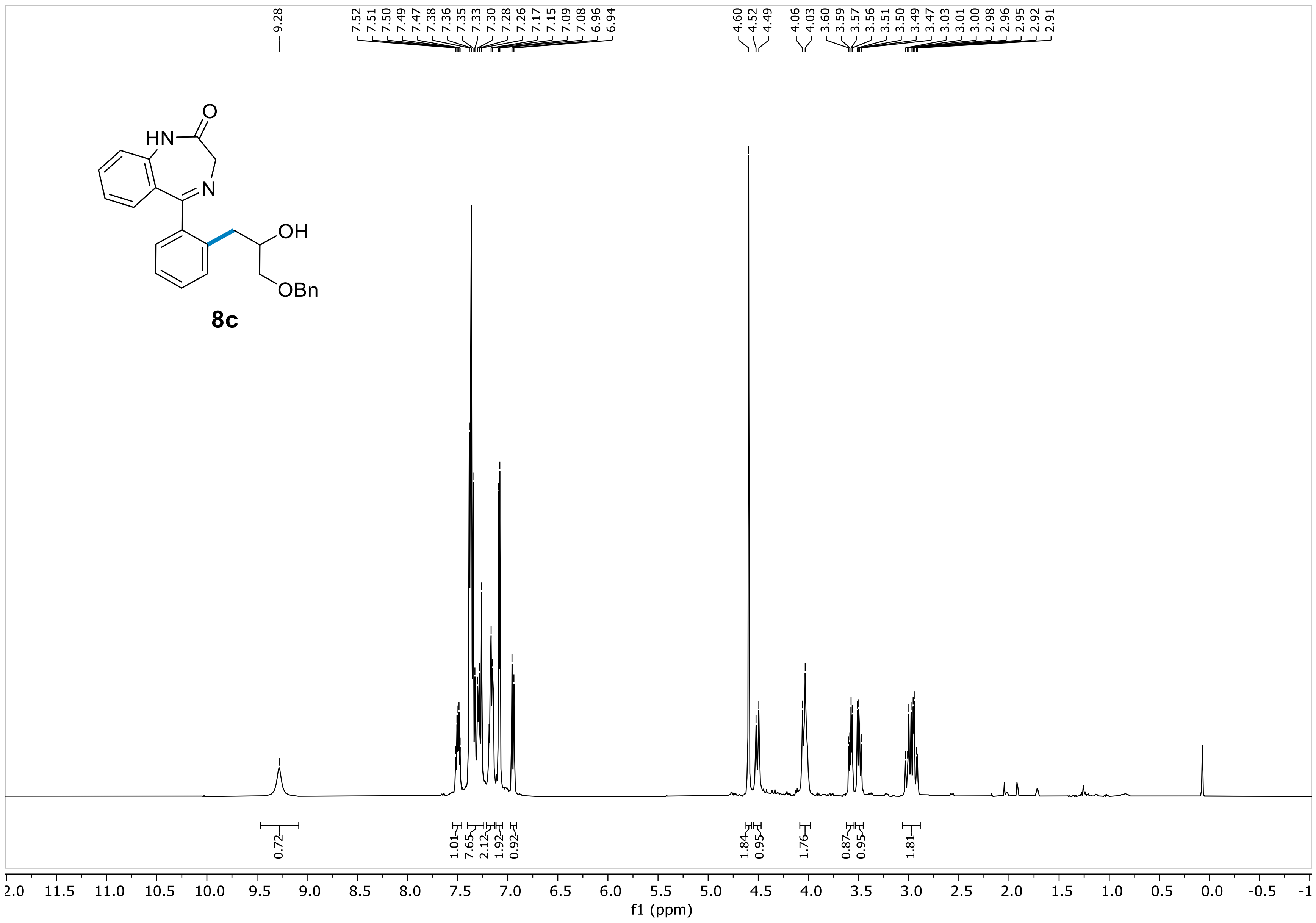

^{1}H NMR spectra (400 MHz, $CDCl_3$) of 5-(2-(3-(benzyloxy)-2-hydroxypropyl)phenyl)-1,3-dihydro-2H-benzo[e][1,4]diazepin-2-one (**8c**)

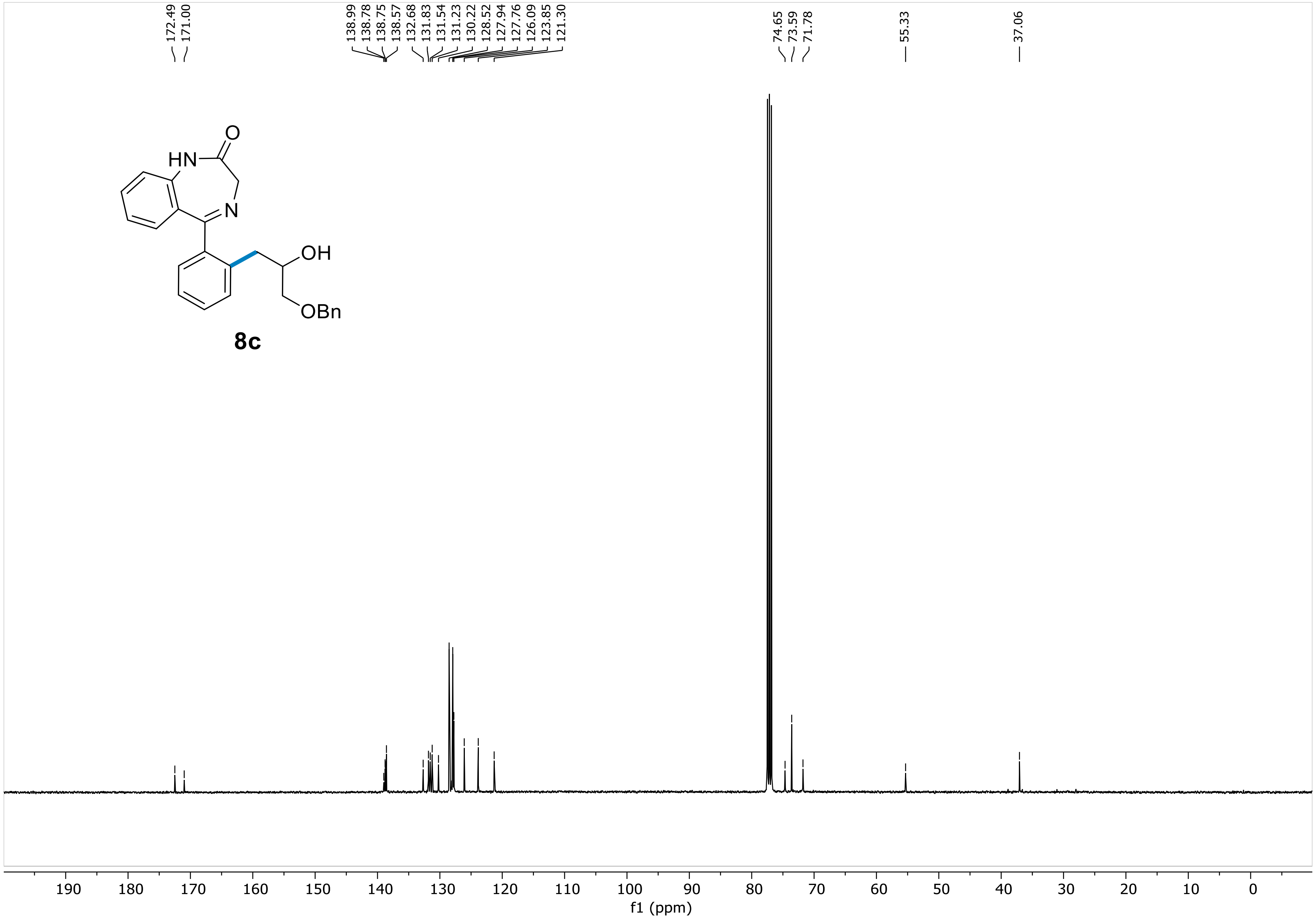

^{13}C NMR spectra (101 MHz, $CDCl_3$) of 5-(2-(3-(benzyloxy)-2-hydroxypropyl)phenyl)-1,3-dihydro-2H-benzo[e][1,4]diazepin-2-one (**8c**)

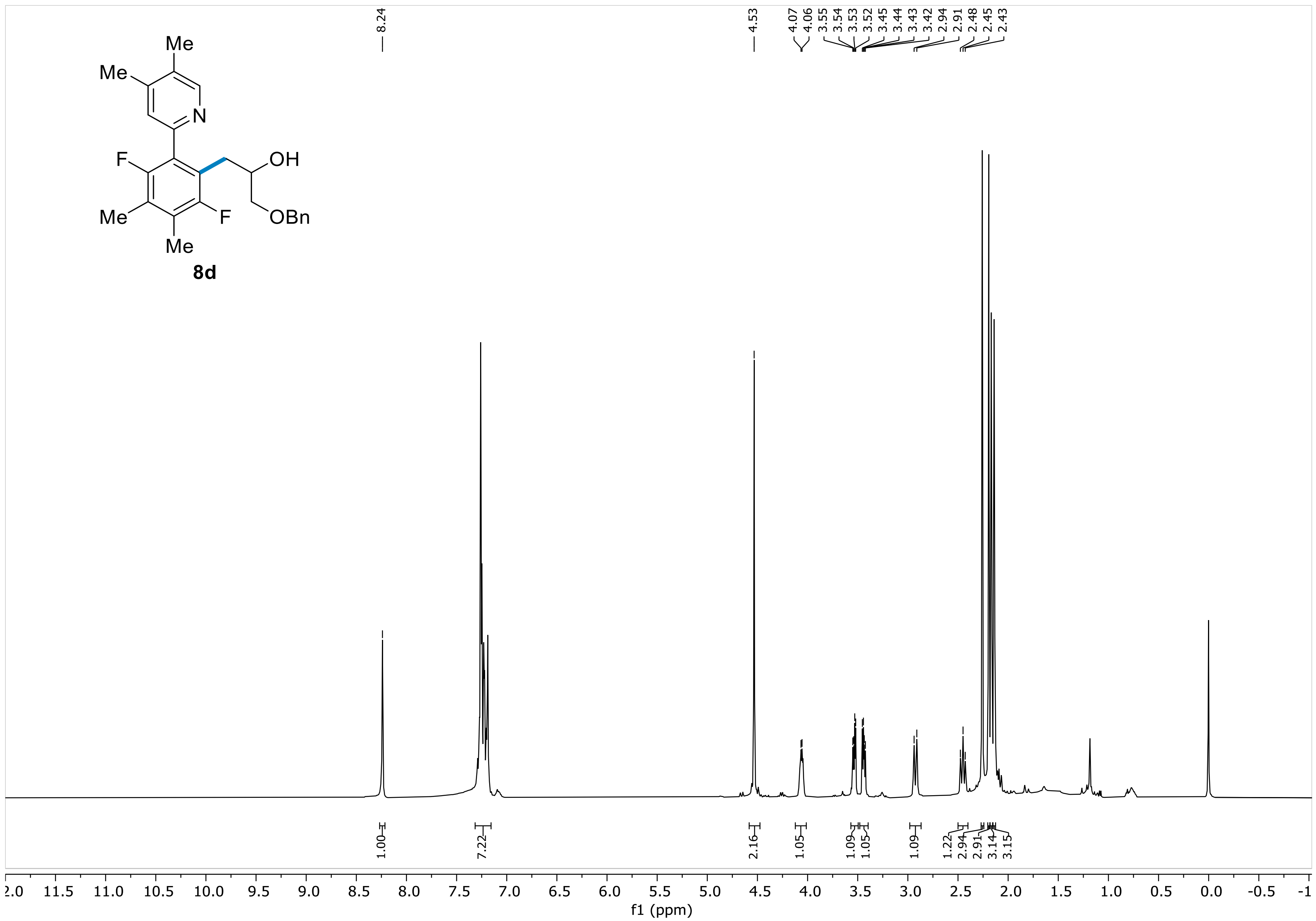

^{1}H NMR spectra (500 MHz, $CDCl_3$) of 1-(benzyloxy)-3-(2-(4,5-dimethylpyridin-2-yl)-3,6-difluoro-4,5-dimethylphenyl)propan-2-ol (**8d**)

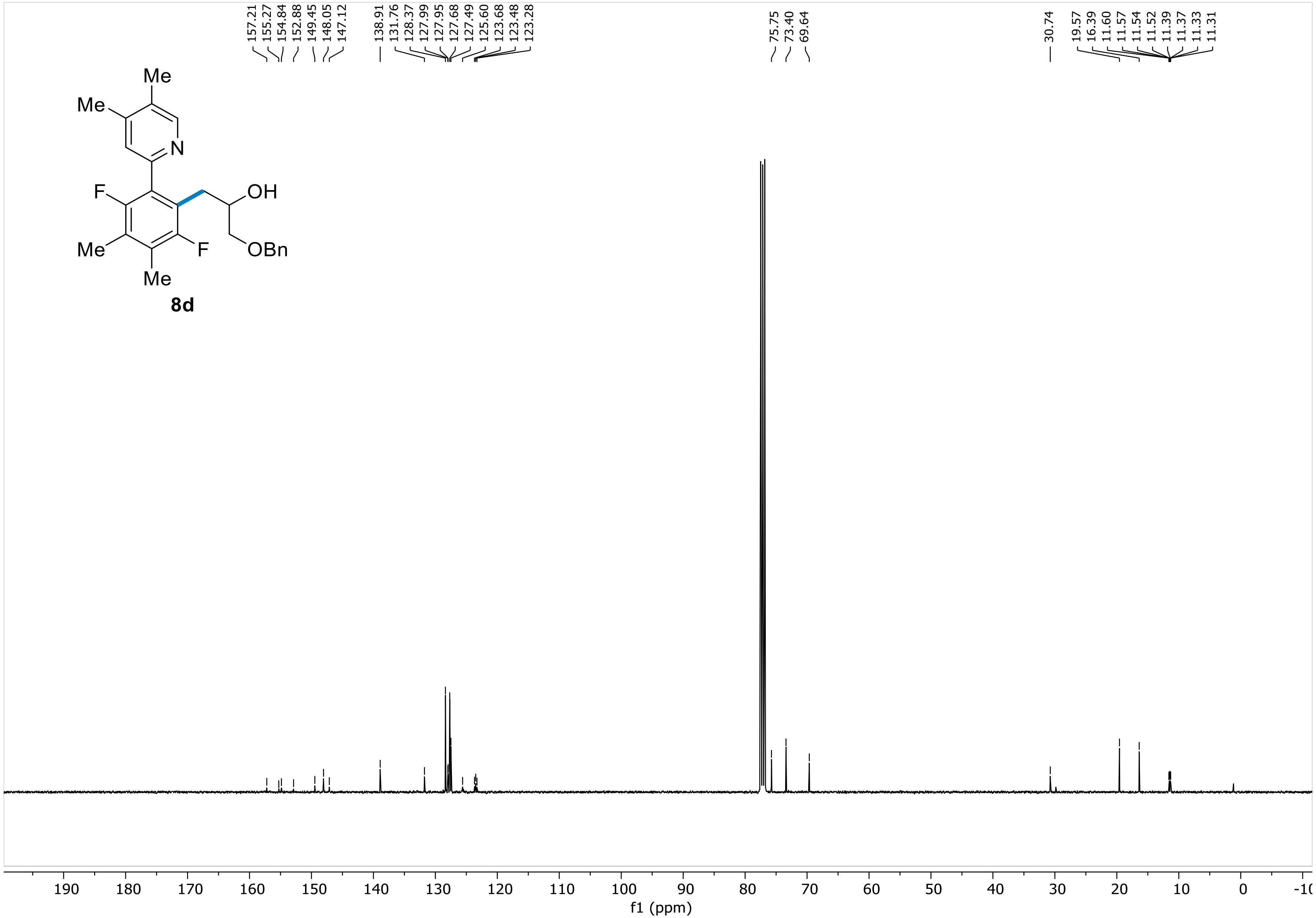

^{13}C NMR spectra (101 MHz, $CDCl_3$) of 1-(benzyloxy)-3-(2-(4,5-dimethylpyridin-2-yl)-3,6-difluoro-4,5-dimethylphenyl)propan-2-ol (**8d**)

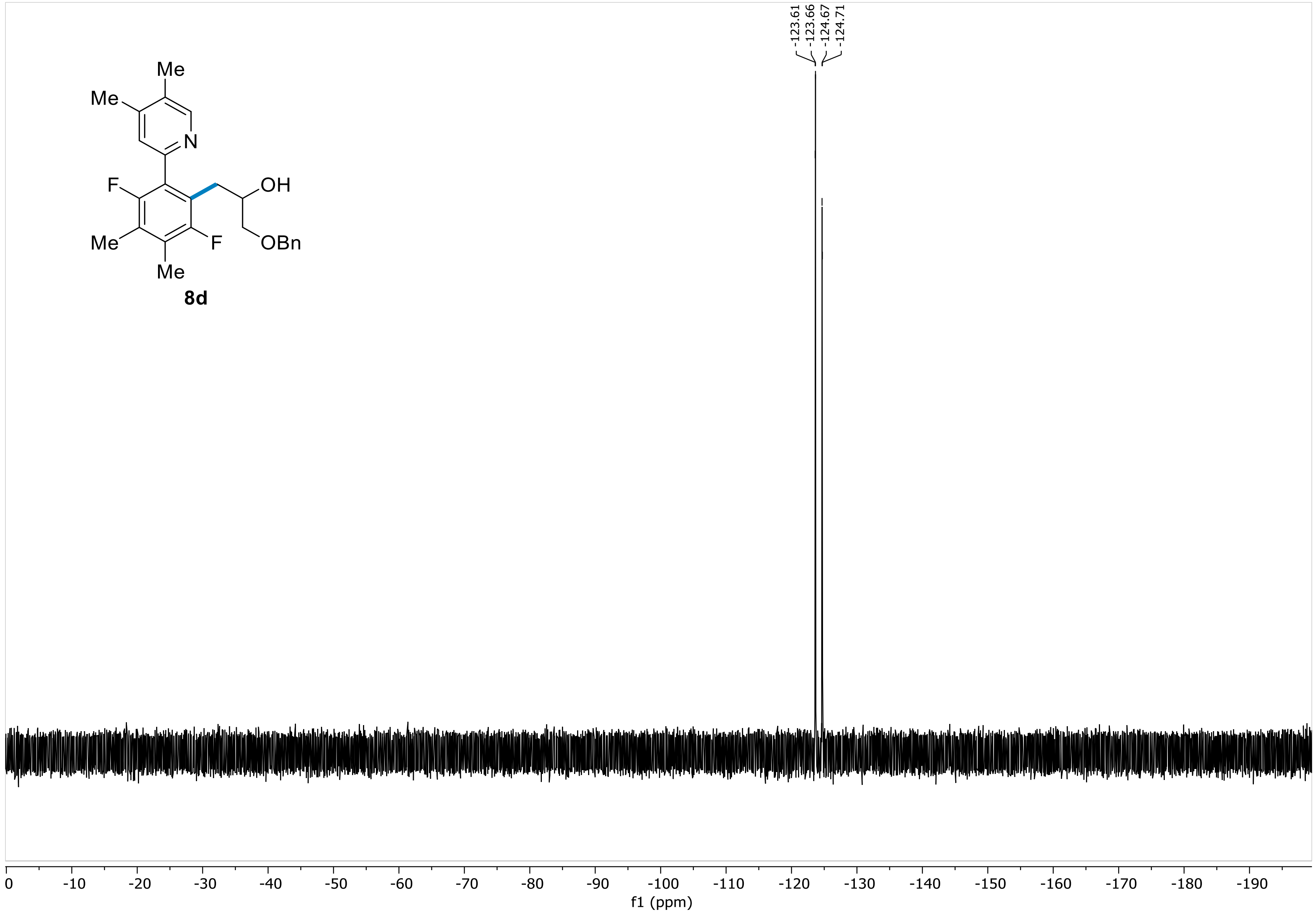

^{19}F NMR spectra (376 MHz, $CDCl_3$) of 1-(benzyloxy)-3-(2-(4,5-dimethylpyridin-2-yl)-3,6-difluoro-4,5-dimethylphenyl)propan-2-ol (**8d**)

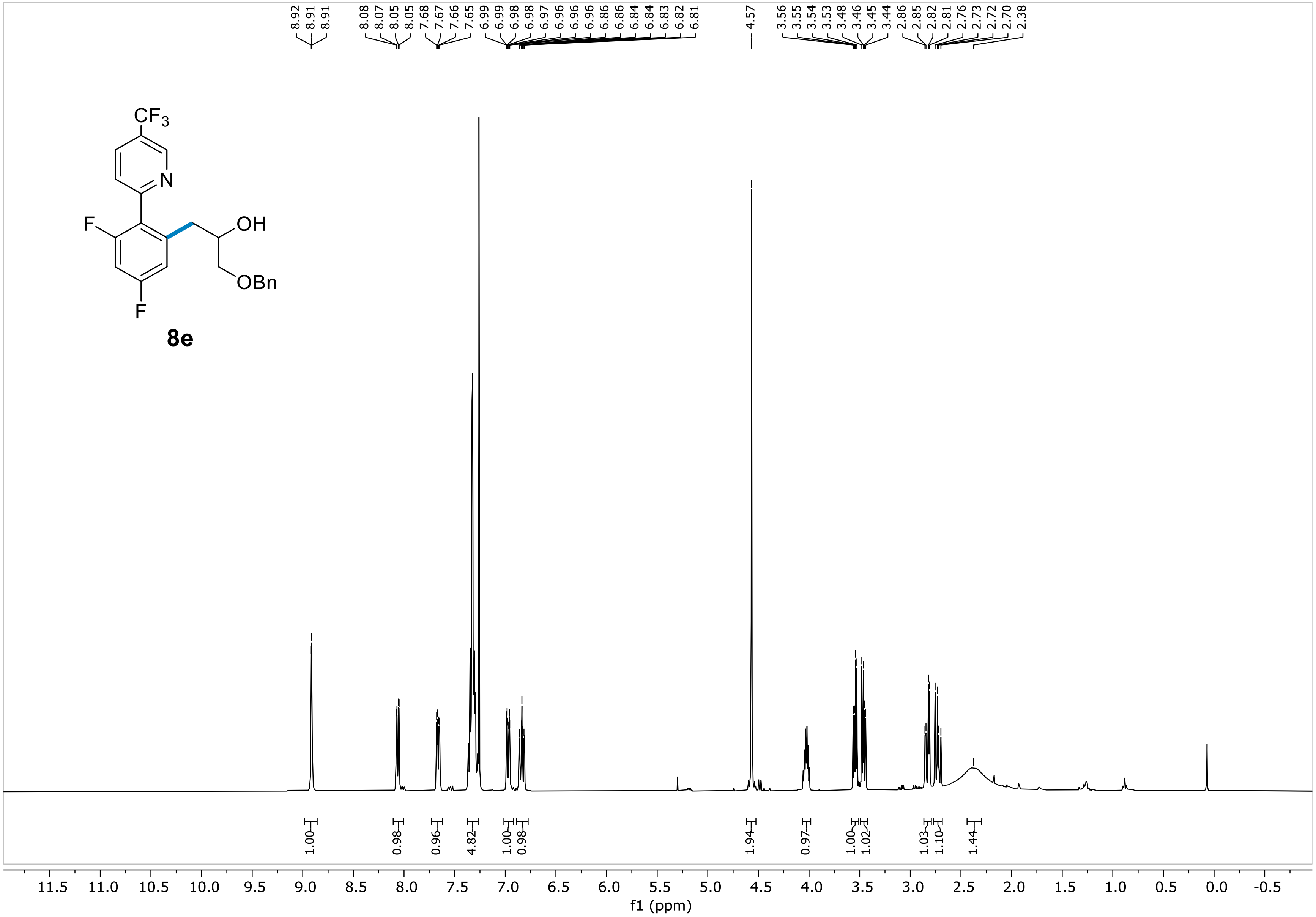

^{1}H NMR spectra (400 MHz, $CDCl_3$) of 1-(benzyloxy)-3-(3,5-difluoro-2-(5-(trifluoromethyl)pyridin-2-yl)phenyl)propan-2-ol (**8e**)

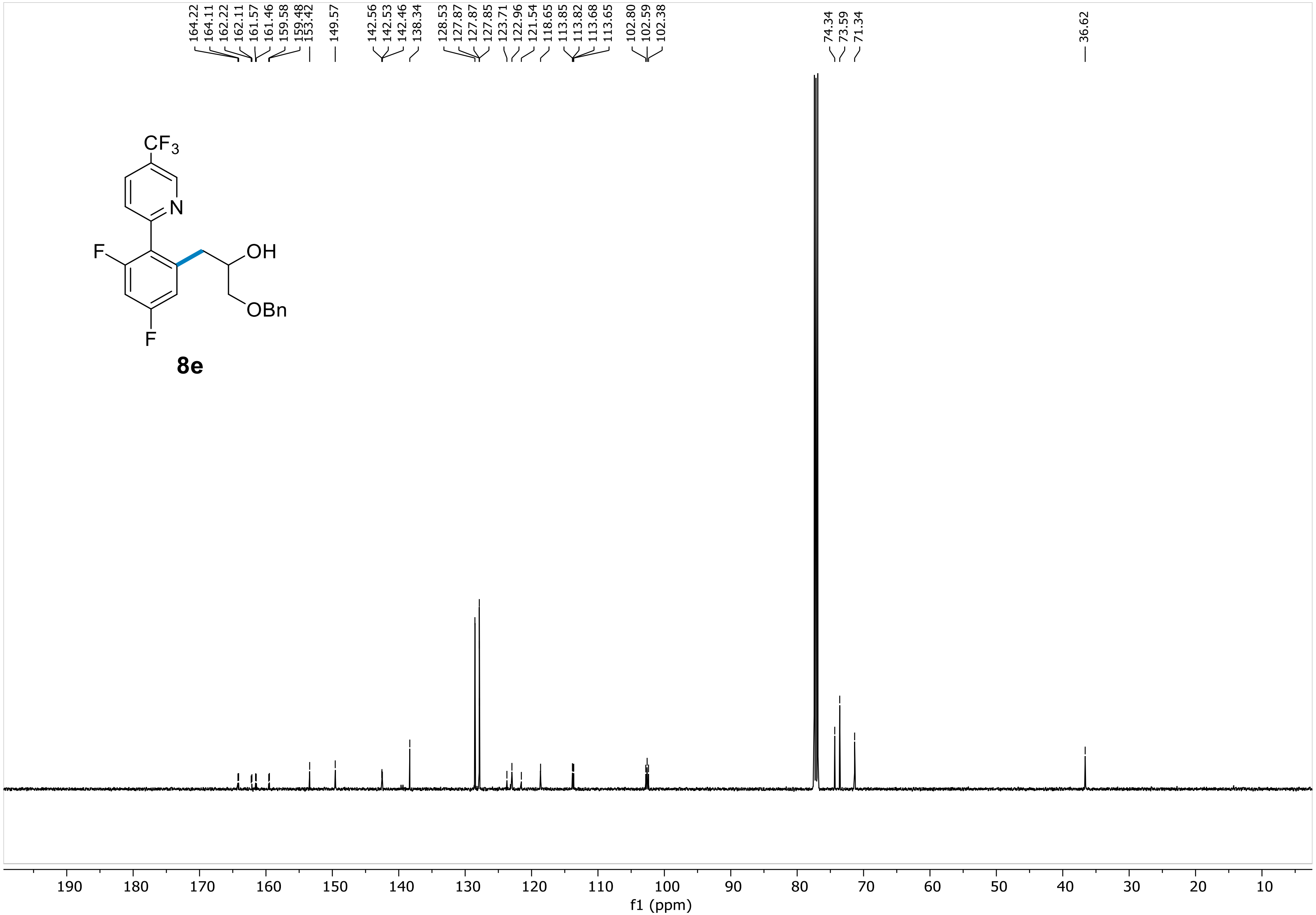

^{13}C NMR spectra (126 MHz, $CDCl_3$) of 1-(benzyloxy)-3-(3,5-difluoro-2-(5-(trifluoromethyl)pyridin-2-yl)phenyl)propan-2-ol (**8e**)

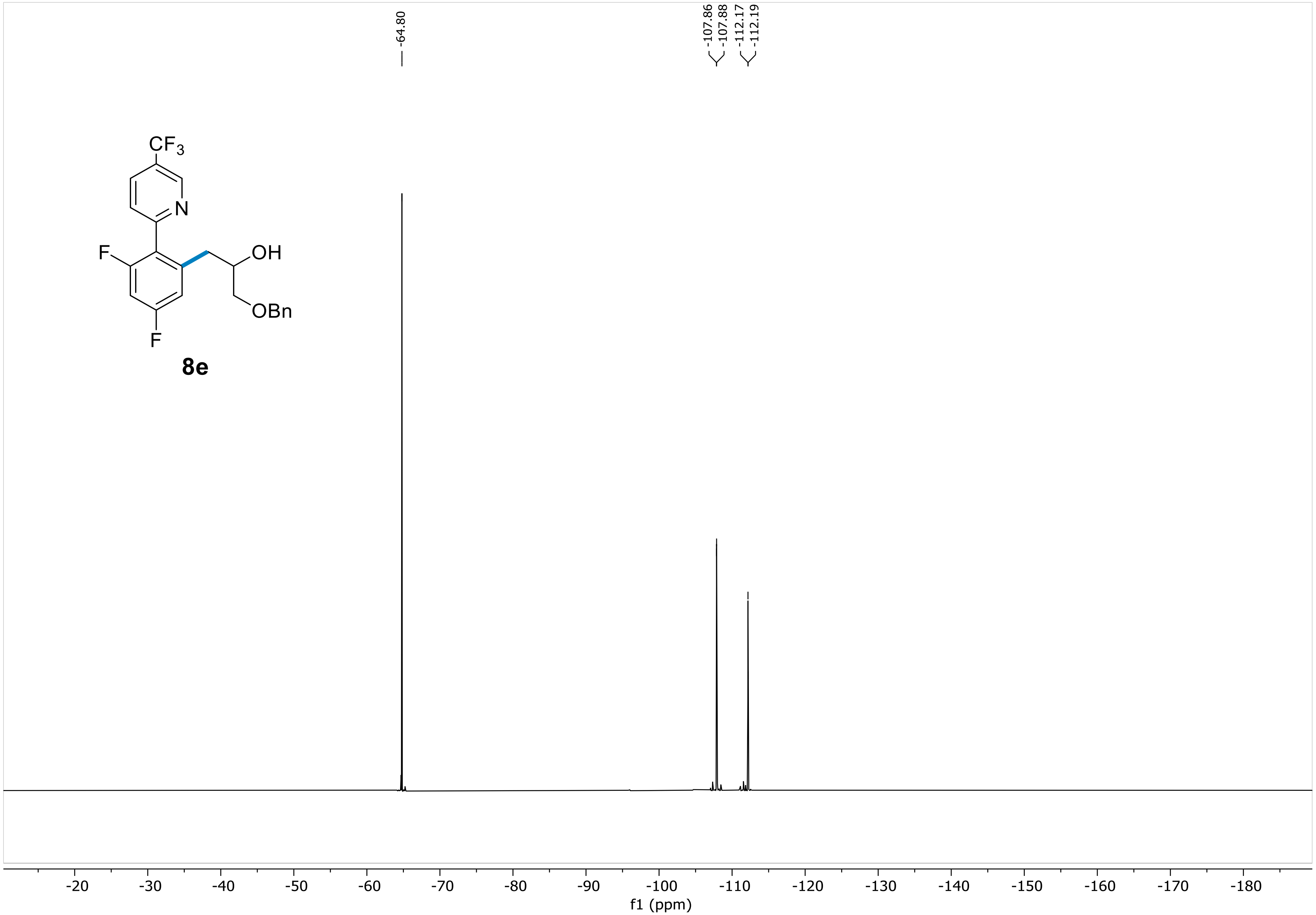

^{19}F NMR spectra (471 MHz, $CDCl_3$) of 1-(benzyloxy)-3-(3,5-difluoro-2-(5-(trifluoromethyl)pyridin-2-yl)phenyl)propan-2-ol (**8e**)

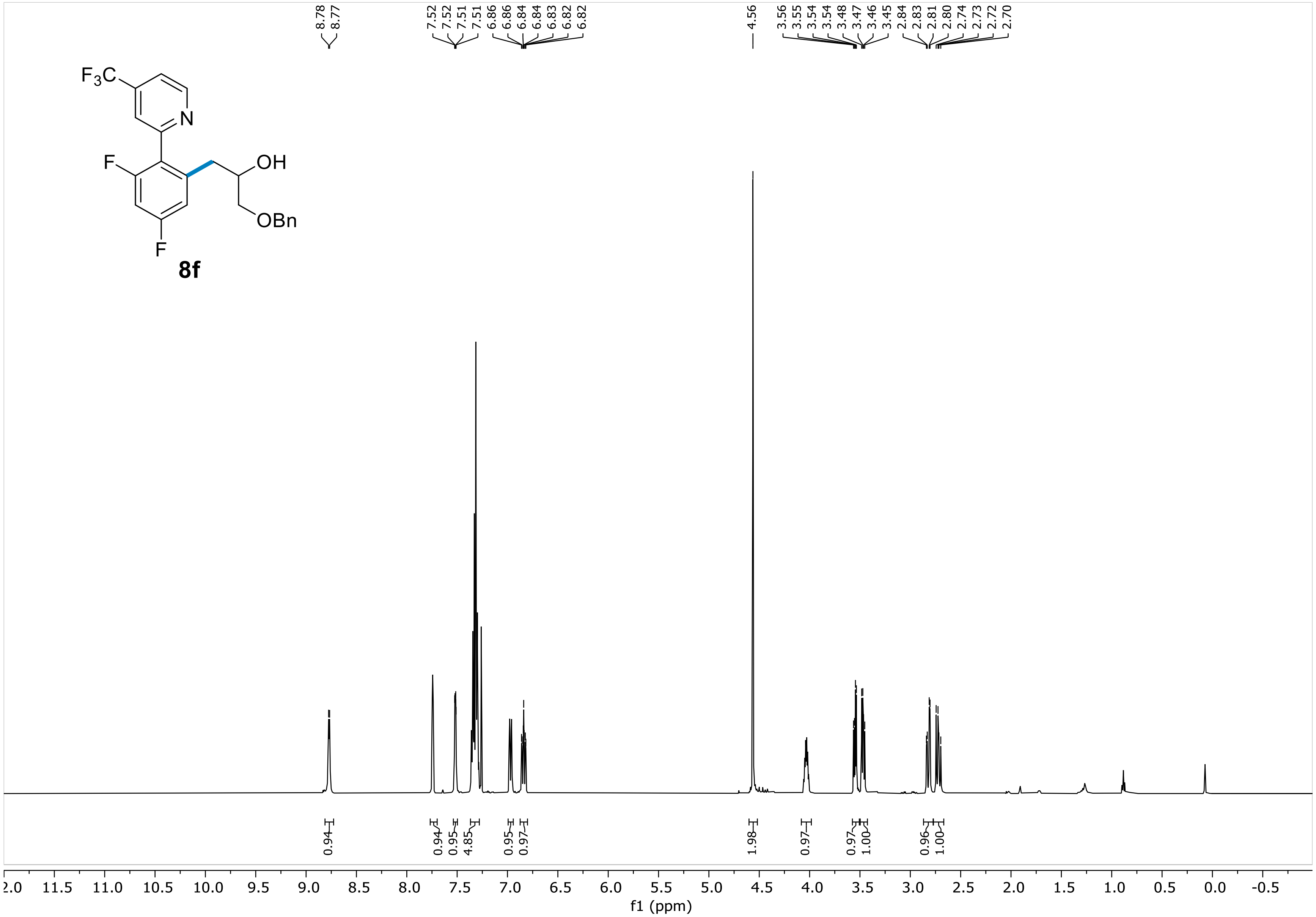

^{1}H NMR spectra (400 MHz, $CDCl_3$) of 1-(benzyloxy)-3-(3,5-difluoro-2-(4-(trifluoromethyl)pyridin-2-yl)phenyl)propan-2-ol (**8f**)

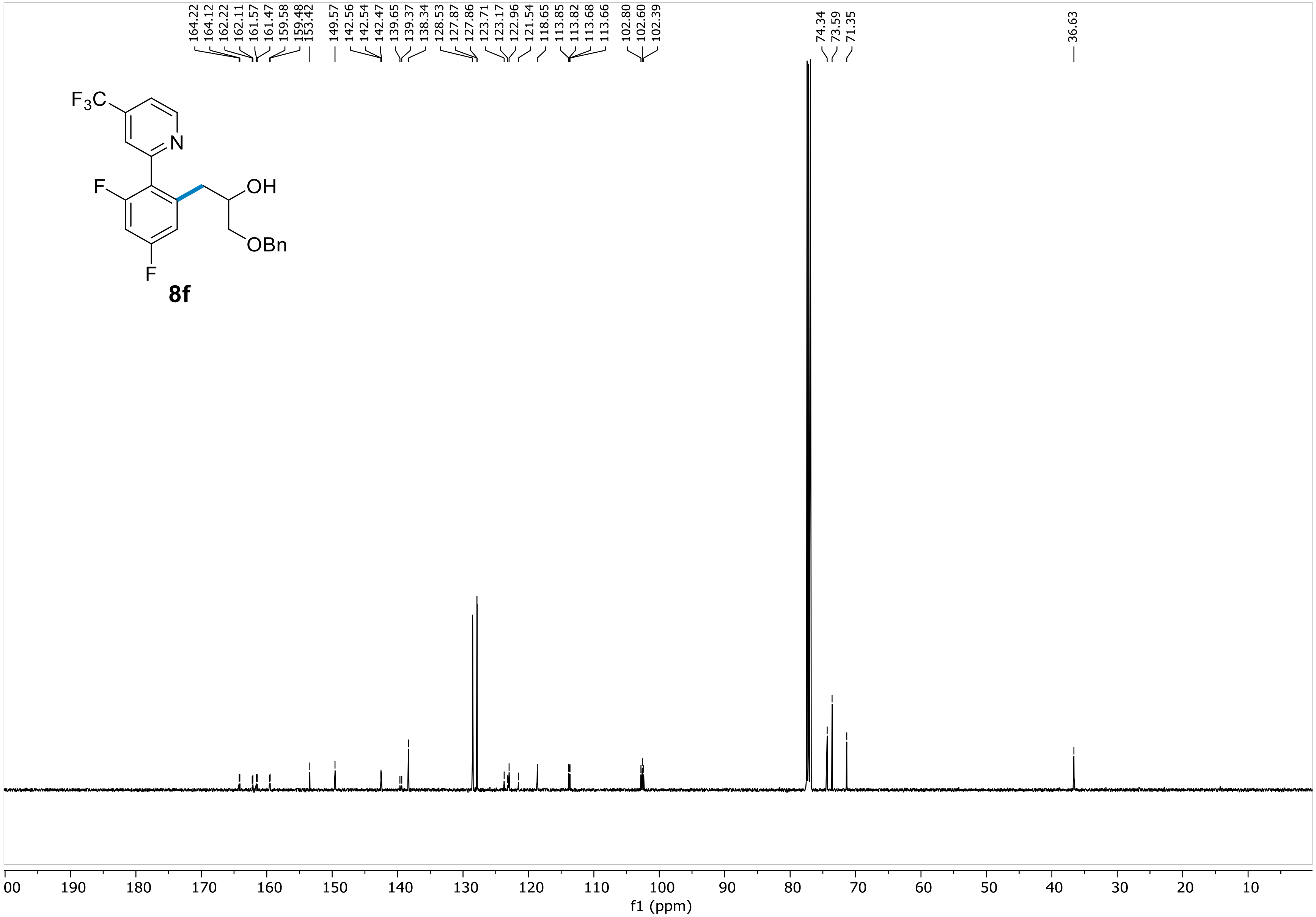

^{13}C NMR spectra (126 MHz, $CDCl_3$) of 1-(benzyloxy)-3-(3,5-difluoro-2-(4-(trifluoromethyl)pyridin-2-yl)phenyl)propan-2-ol (**8f**)

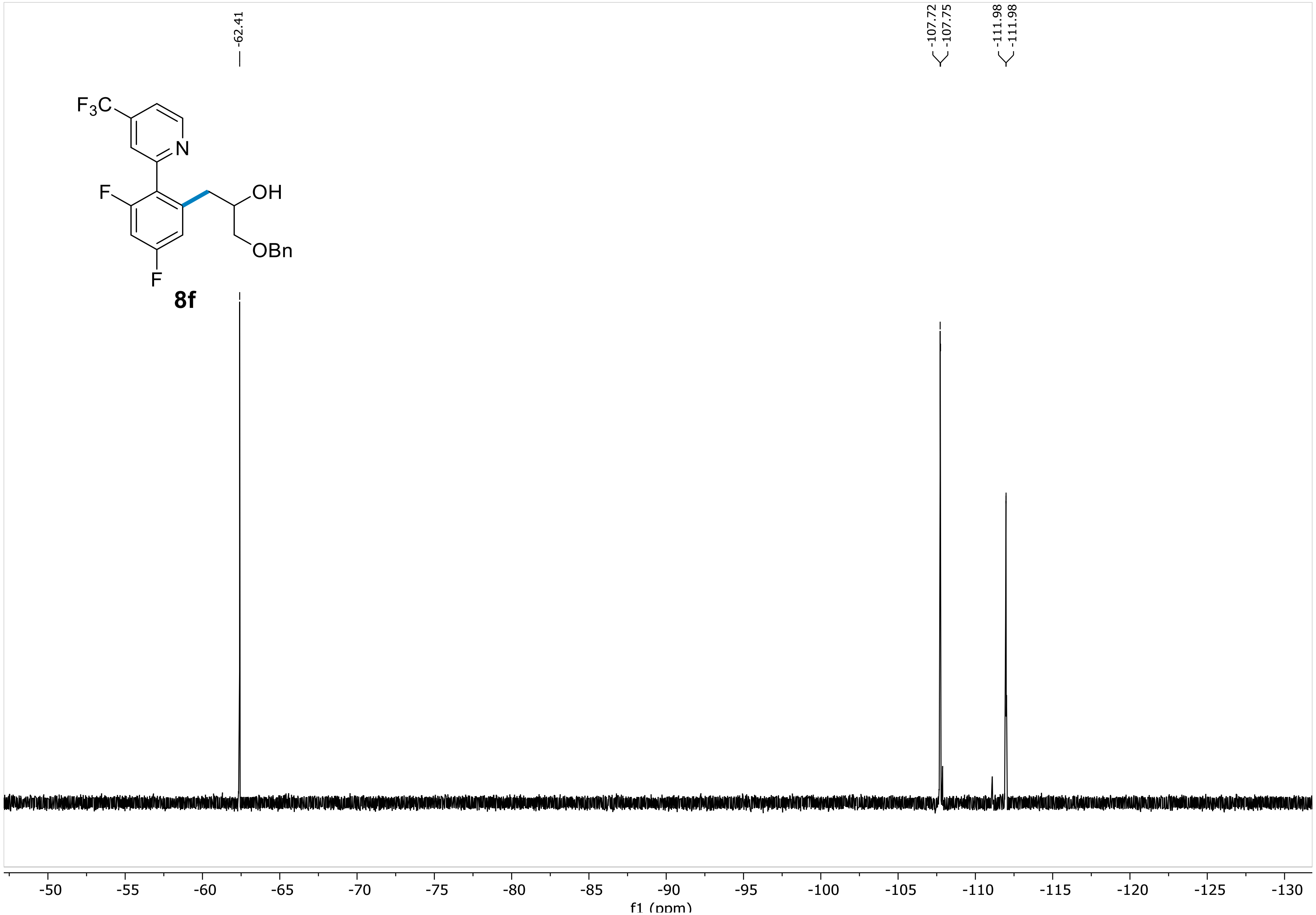

^{19}F NMR spectra (376 MHz, $CDCl_3$) of 1-(benzyloxy)-3-(3,5-difluoro-2-(4-(trifluoromethyl)pyridin-2-yl)phenyl)propan-2-ol (**8f**)

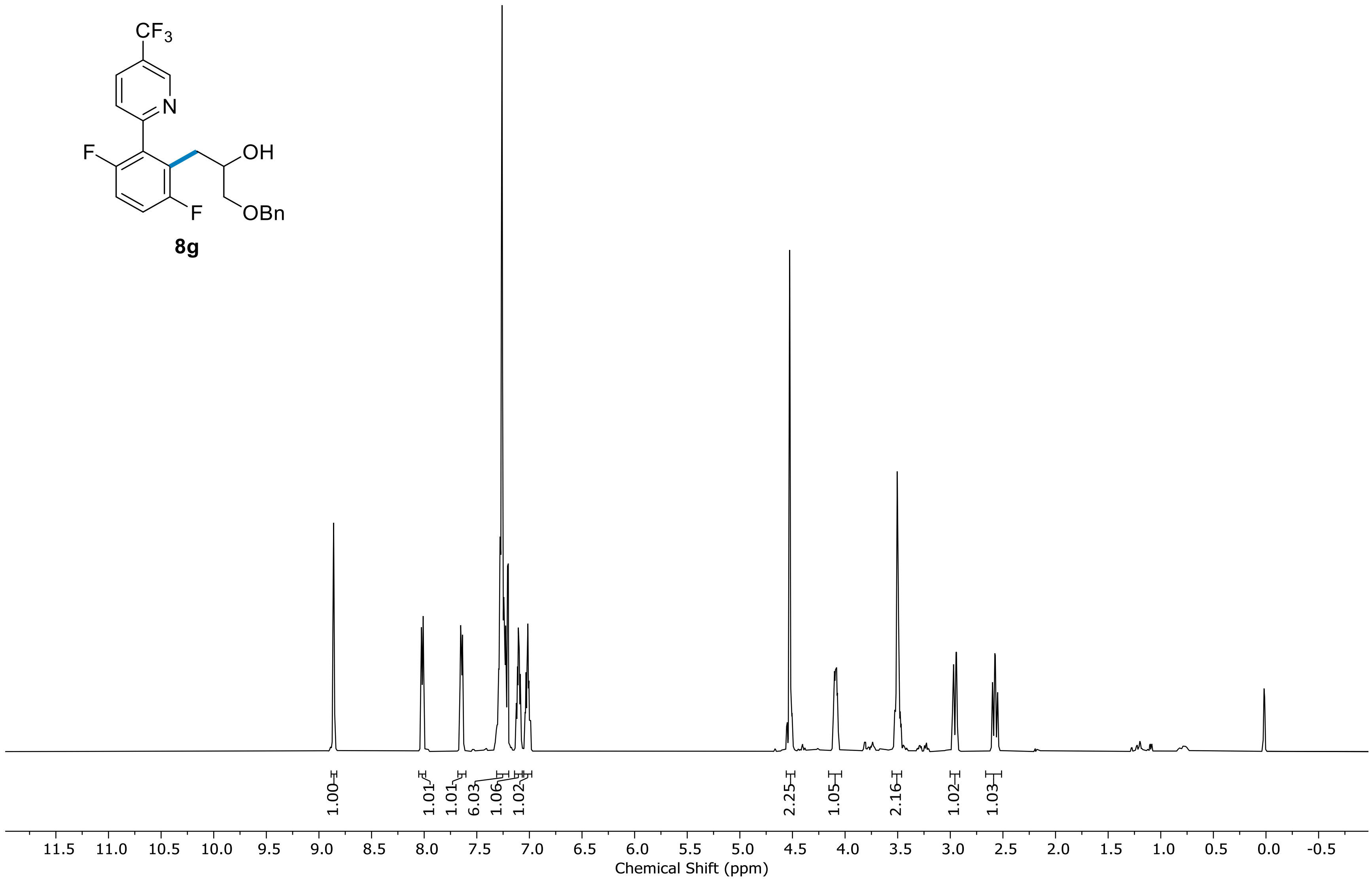

^{1}H NMR spectra (400 MHz, $CDCl_3$) of 1-(benzyloxy)-3-(3,6-difluoro-2-(5-(trifluoromethyl)pyridin-2-yl)phenyl)propan-2-ol (**8g**)

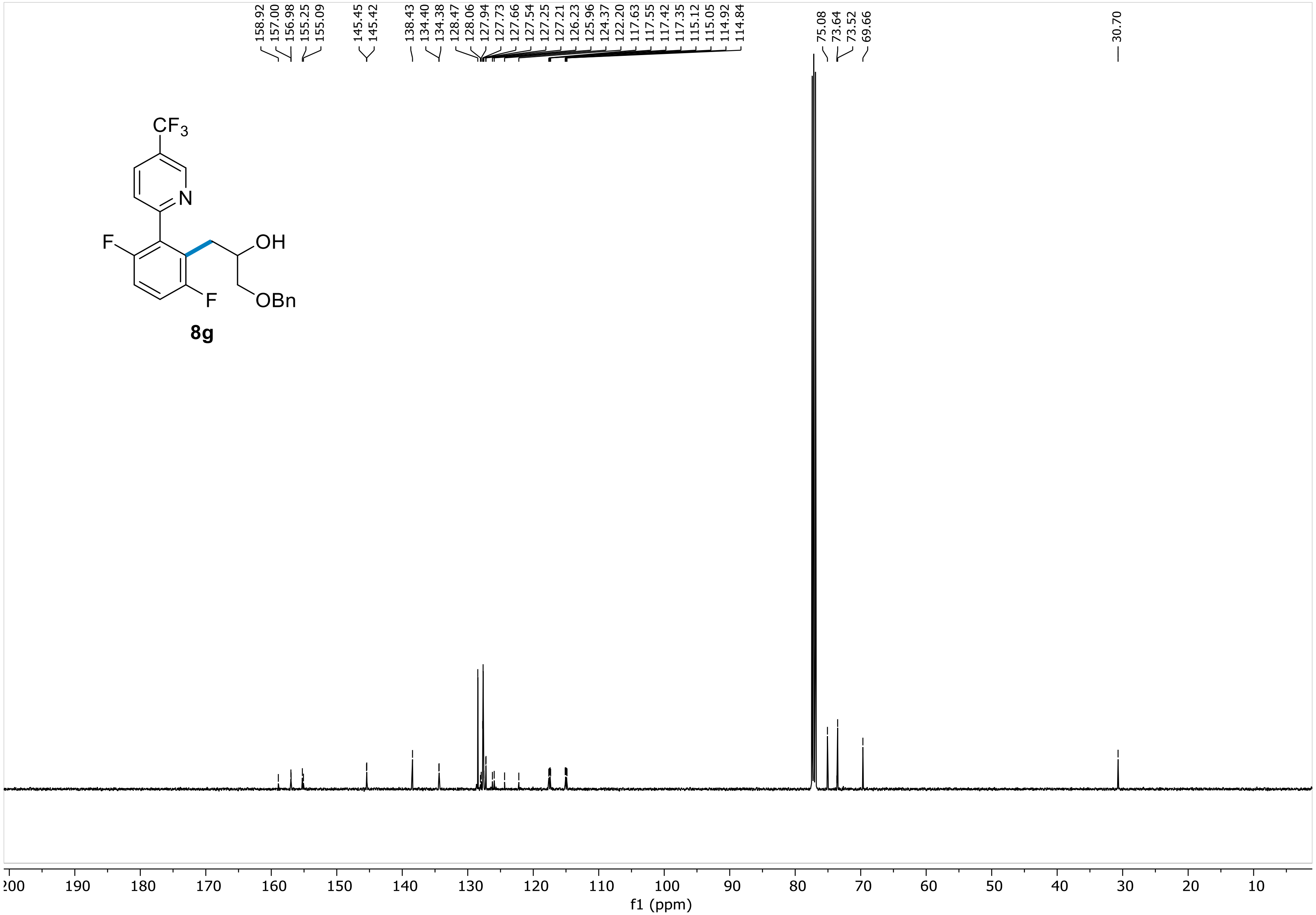

^{13}C NMR spectra (126 MHz, $CDCl_3$) of 1-(benzyloxy)-3-(3,6-difluoro-2-(5-(trifluoromethyl)pyridin-2-yl)phenyl)propan-2-ol (**8g**)

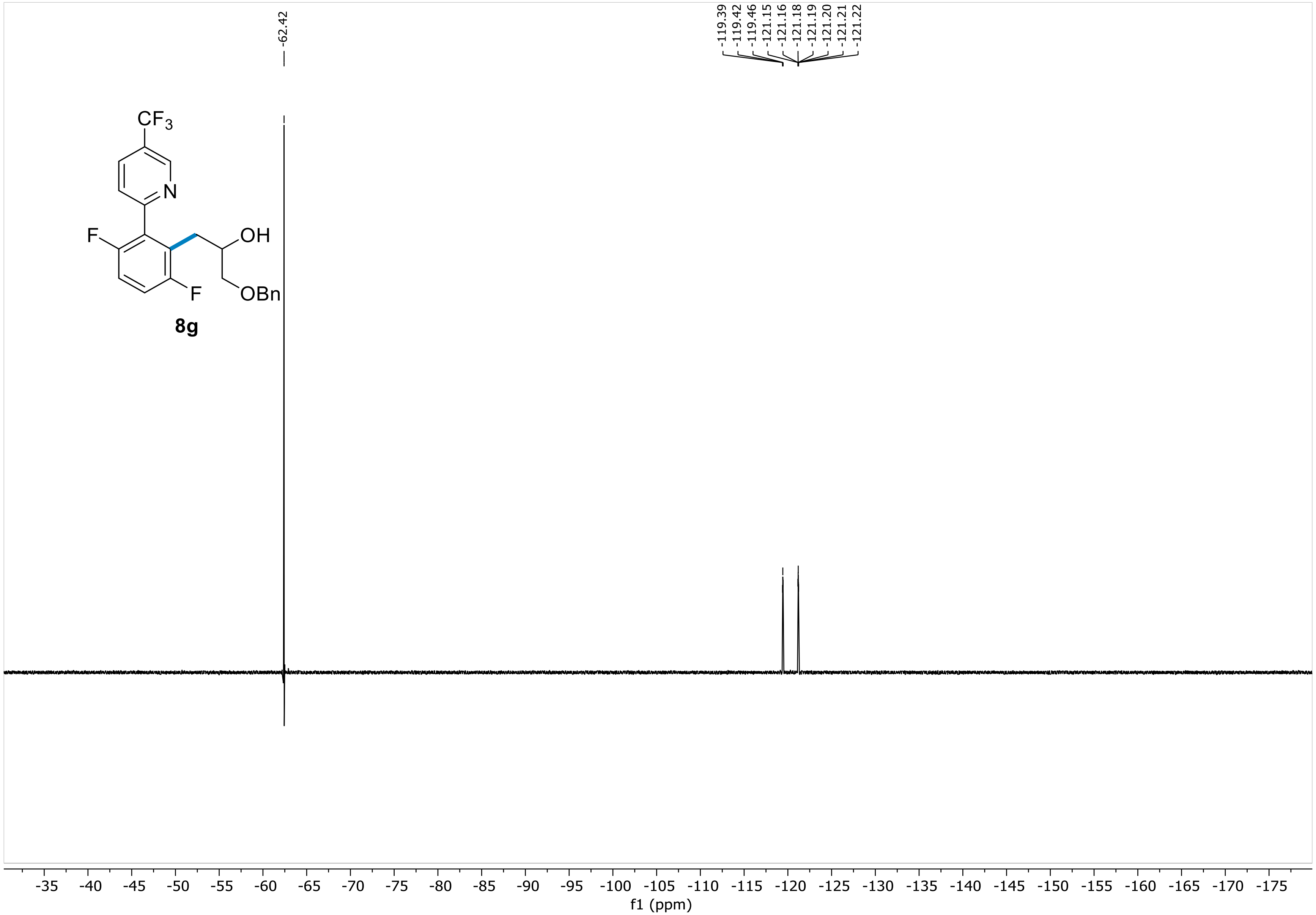

^{19}F NMR spectra (376 MHz, $CDCl_3$) of 1-(benzyloxy)-3-(3,6-difluoro-2-(5-(trifluoromethyl)pyridin-2-yl)phenyl)propan-2-ol (**8g**)

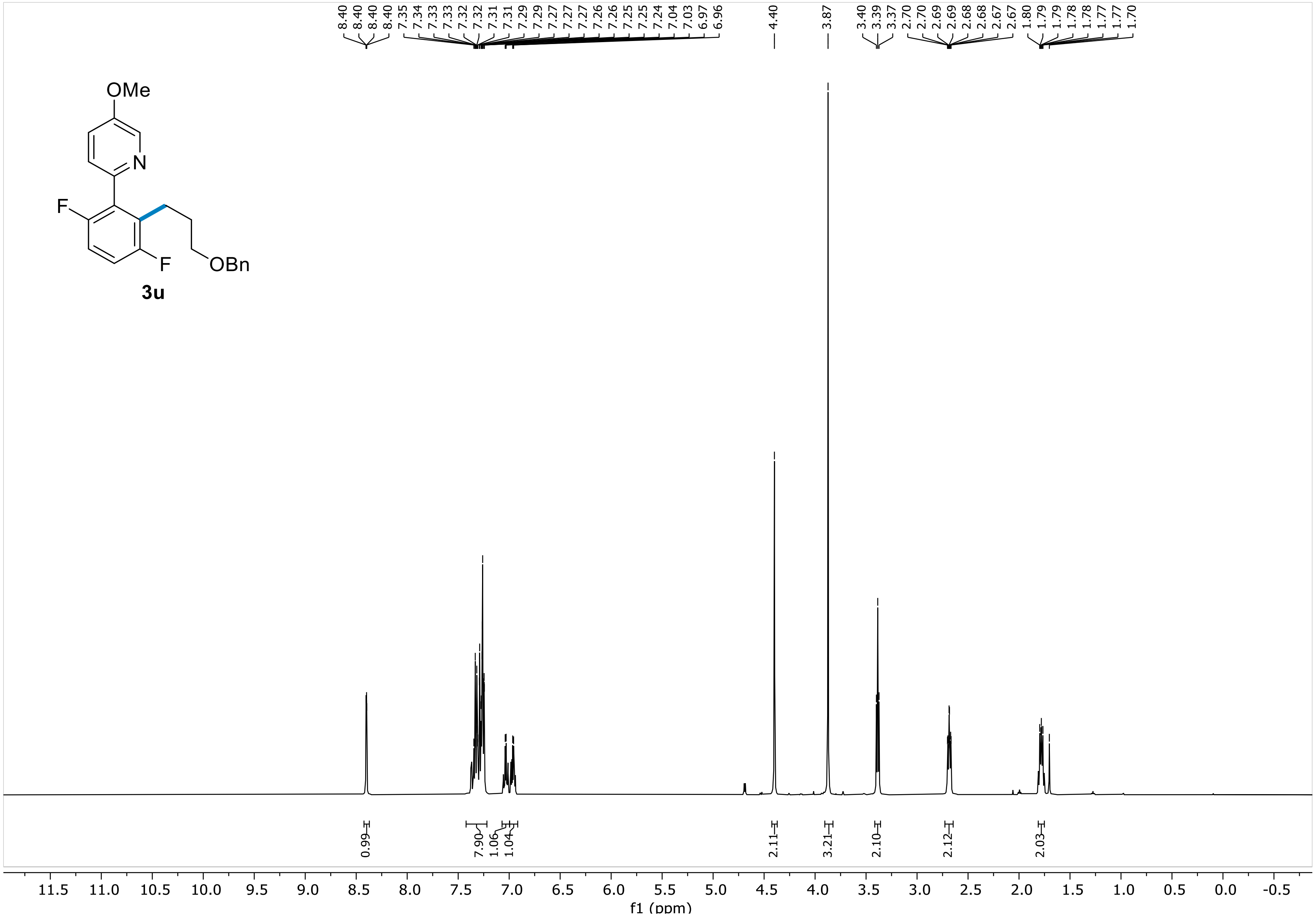

^{1}H NMR spectra (500 MHz, $CDCl_3$) of 2-(2-(3-(benzyloxy)propyl)-3,6-difluorophenyl)-5-methoxypyridine (**3pa**)

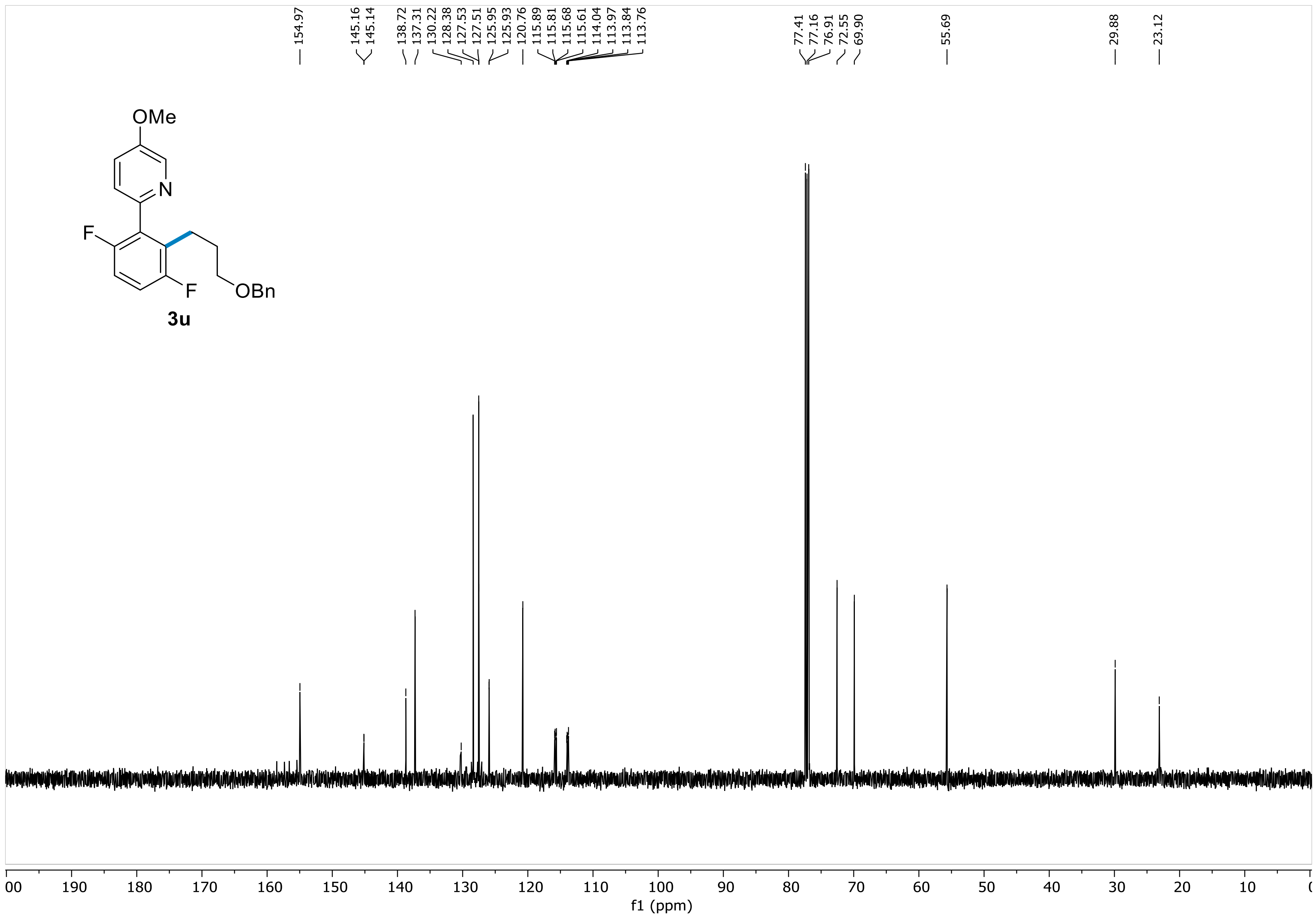

^{13}C NMR spectra (126 MHz, $CDCl_3$) of 2-(2-(3-(benzyloxy)propyl)-3,6-difluorophenyl)-5-methoxypyridine (**3pa**)

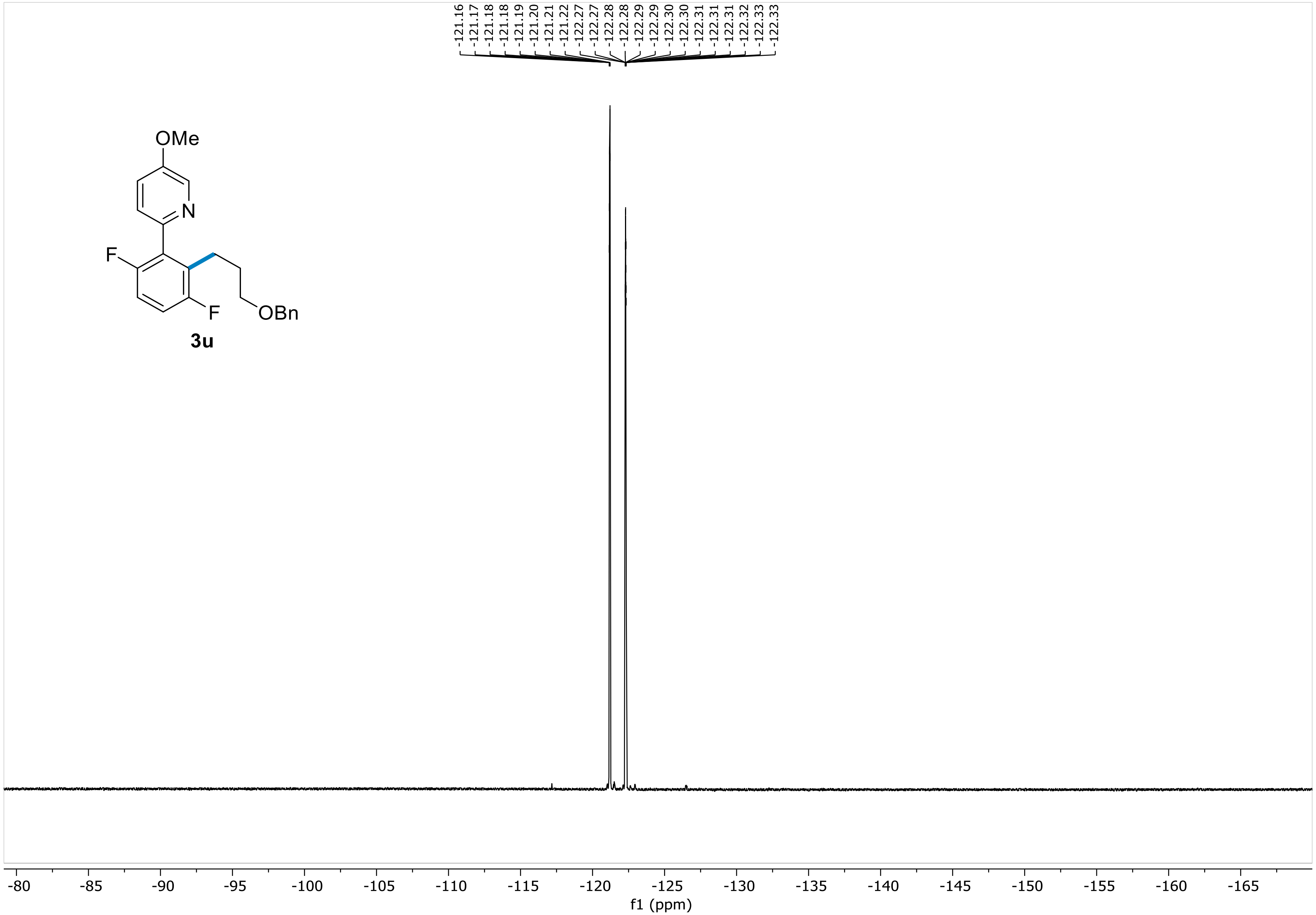

^{19}F NMR spectra (471 MHz, $CDCl_3$) of 2-(2-(3-(benzyloxy)propyl)-3,6-difluorophenyl)-5-methoxypyridine (**3pa**)

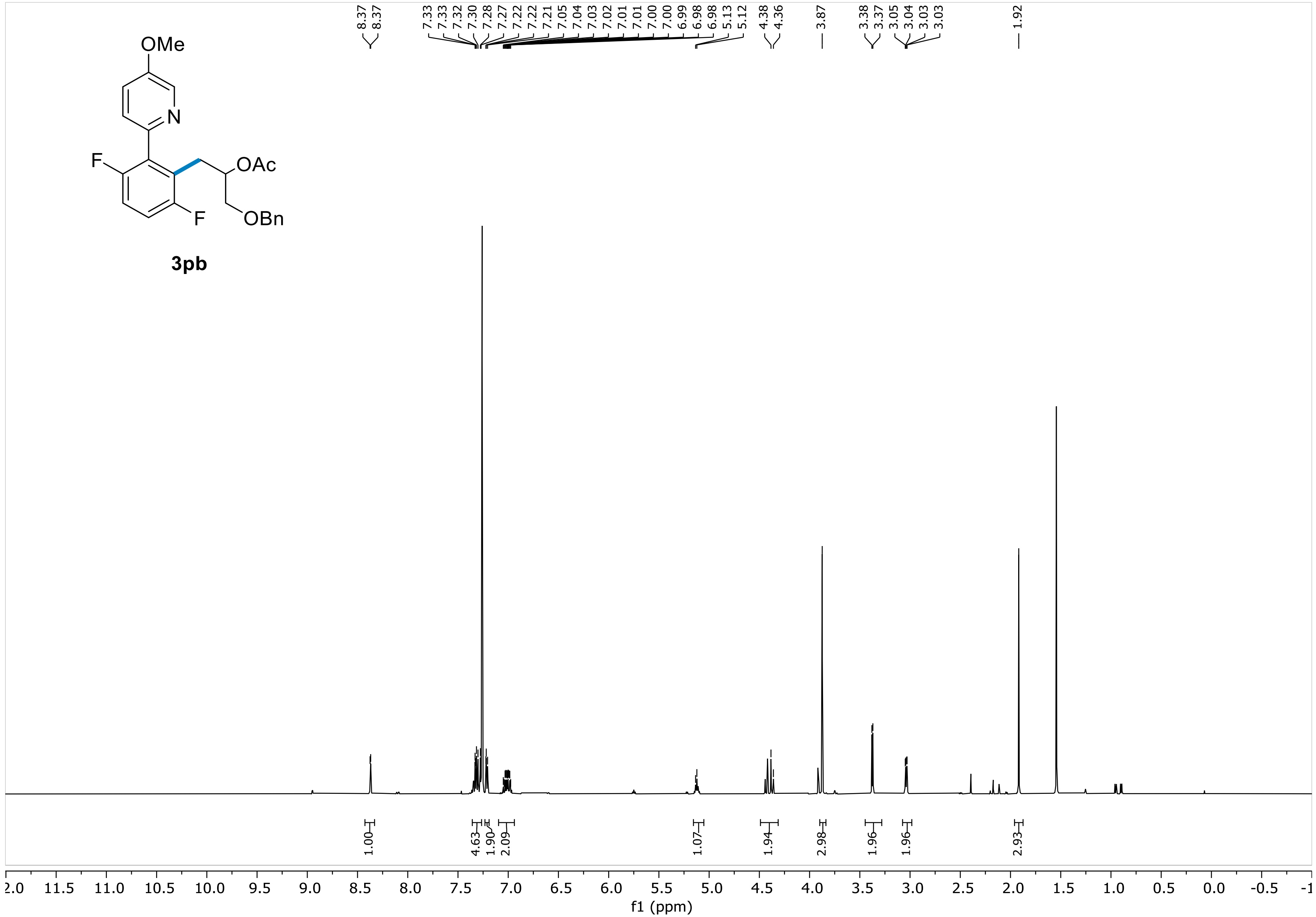

^{1}H NMR spectra (500 MHz, $CDCl_3$) of 1-(benzyloxy)-3-(3,6-difluoro-2-(5-methoxypyridin-2-yl)phenyl)propan-2-yl acetate (**3pb**)

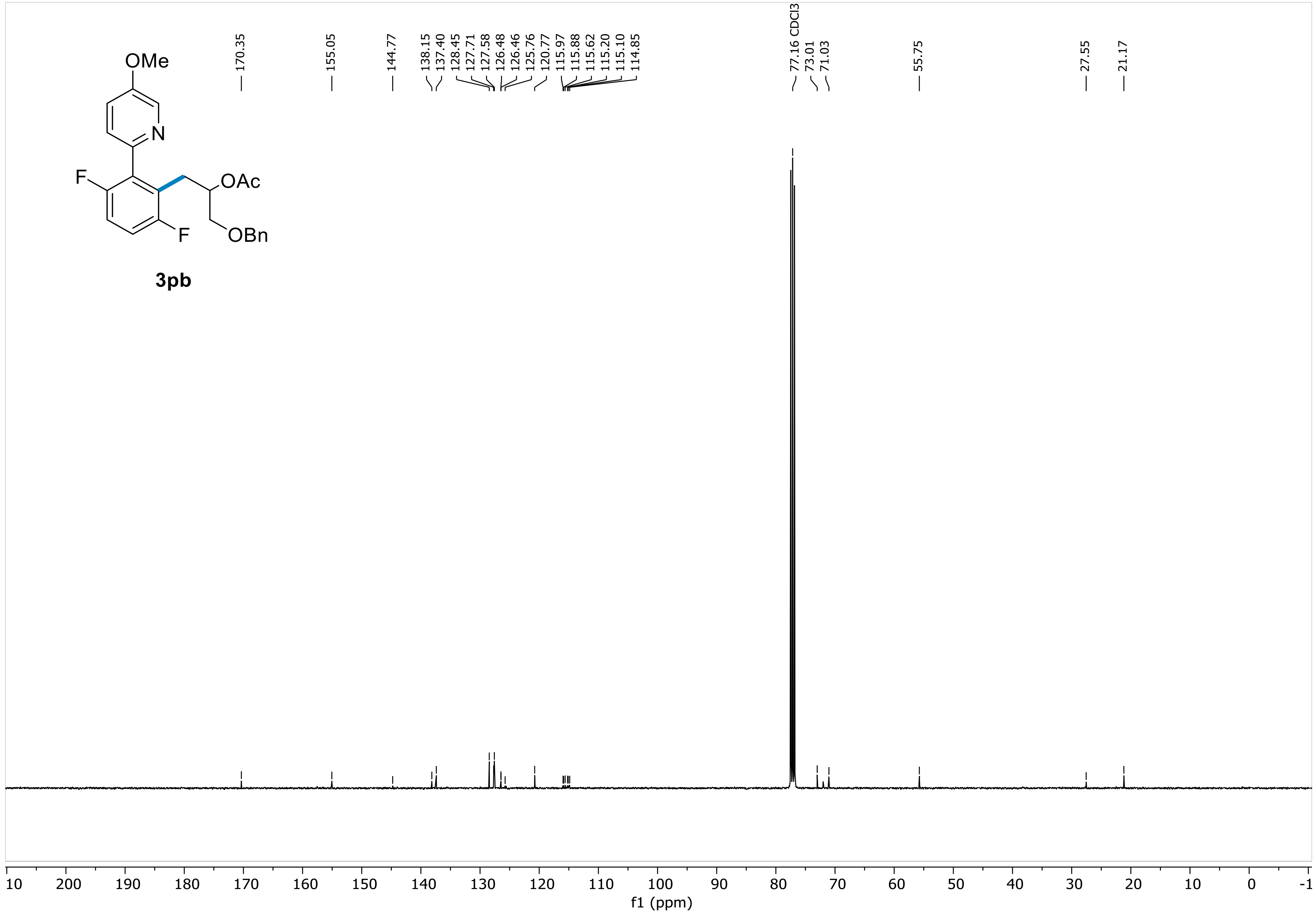

^{13}C NMR spectra (400 MHz, $CDCl_3$) of 1-(benzyloxy)-3-(3,6-difluoro-2-(5-methoxypyridin-2-yl)phenyl)propan-2-yl acetate (**3pb**)

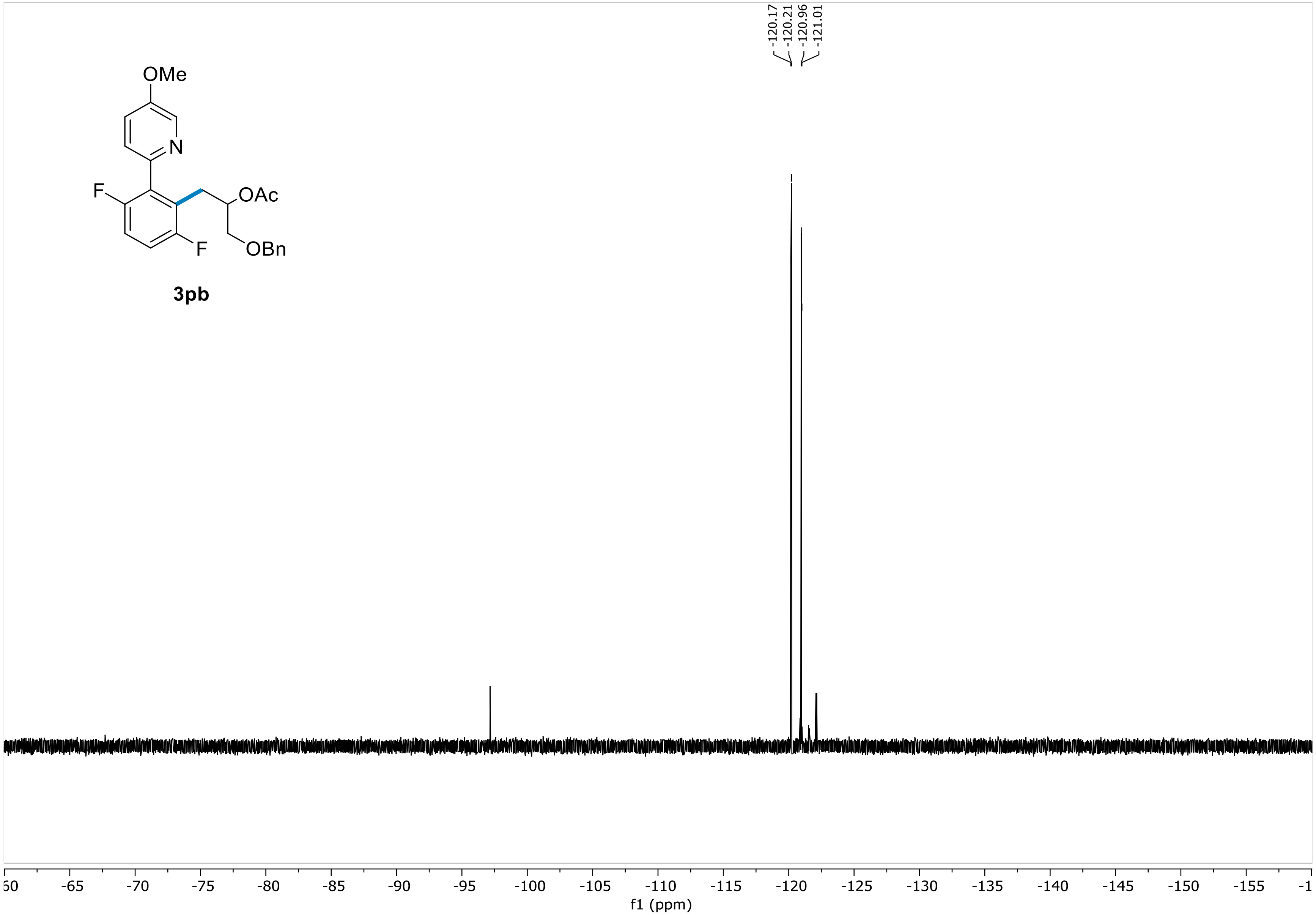

^{19}F NMR spectra (471 MHz, $CDCl_3$) of 1-(benzyloxy)-3-(3,6-difluoro-2-(5-methoxypyridin-2-yl)phenyl)propan-2-yl acetate (**3pb**)

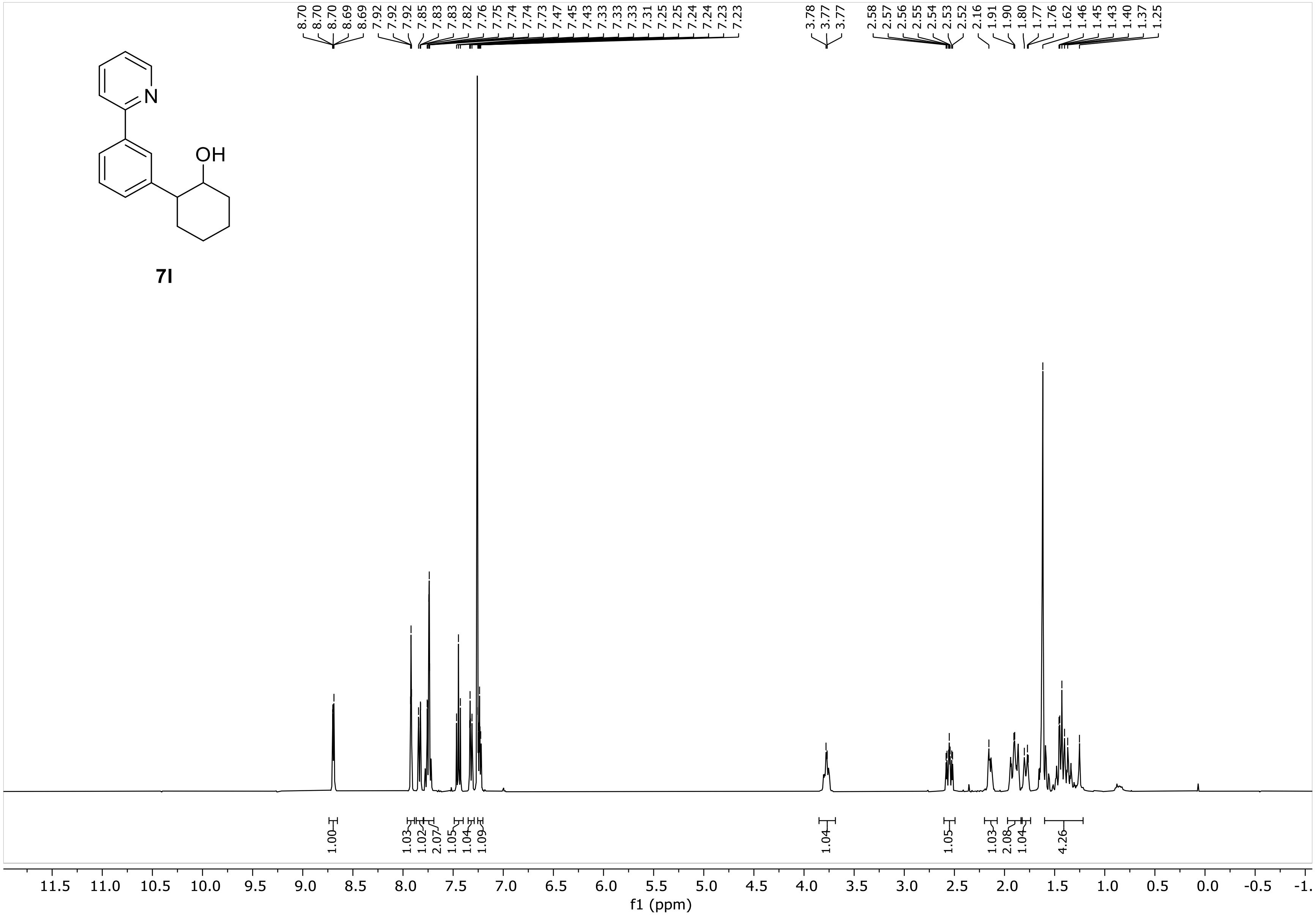

^{1}H NMR spectra (400 MHz, $CDCl_3$) of 2-(3-(pyridin-2-yl)phenyl)cyclohexan-1-ol (**7l**)

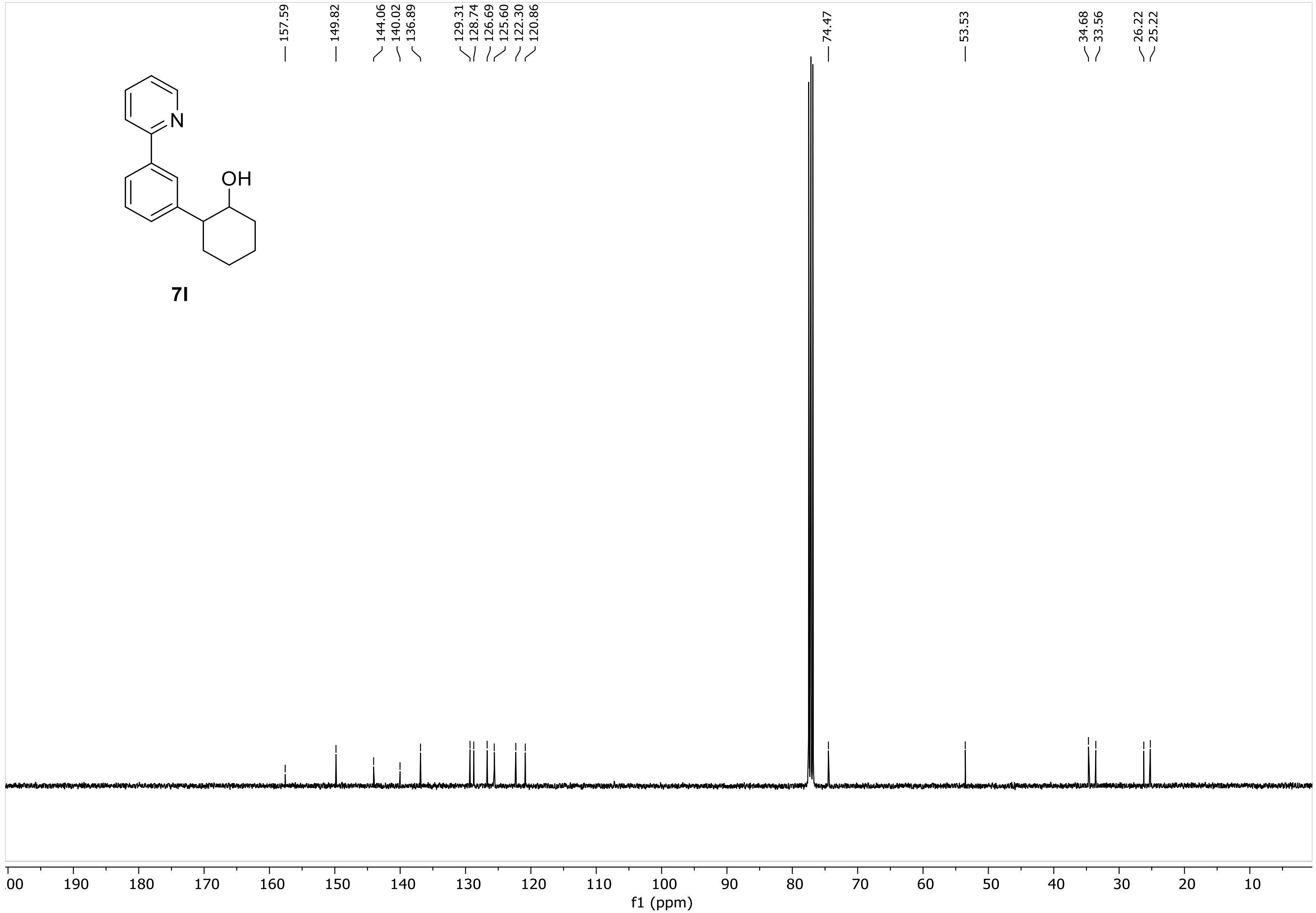

^{13}C NMR spectra (101 MHz, $CDCl_3$) of 2-(3-(pyridin-2-yl)phenyl)cyclohexan-1-ol (**7l**)

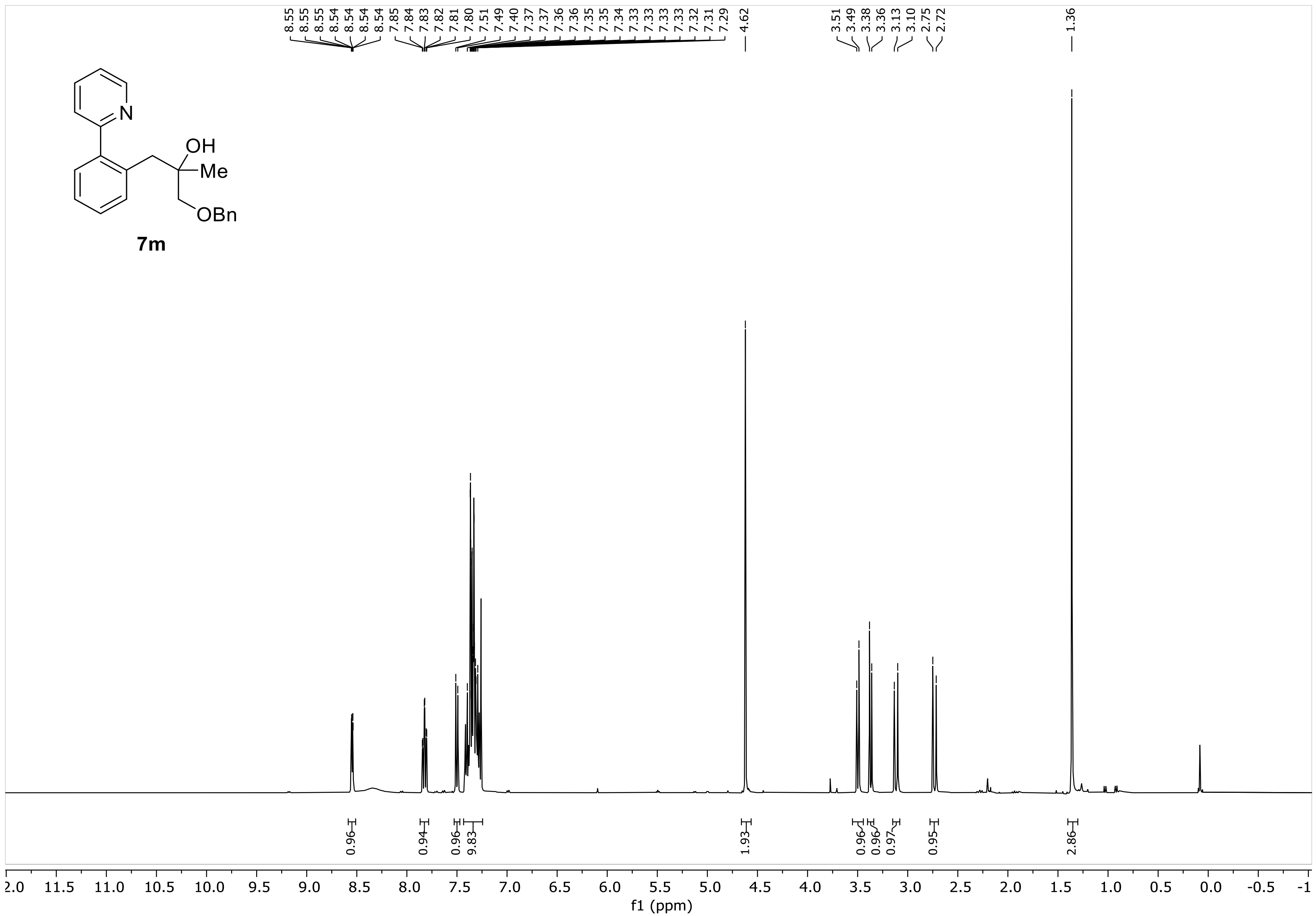

^{1}H NMR spectra (400 MHz, $CDCl_3$) of 1-(benzyloxy)-2-methyl-3-(2-(pyridin-2-yl)phenyl)propan-2-ol (**7m**)

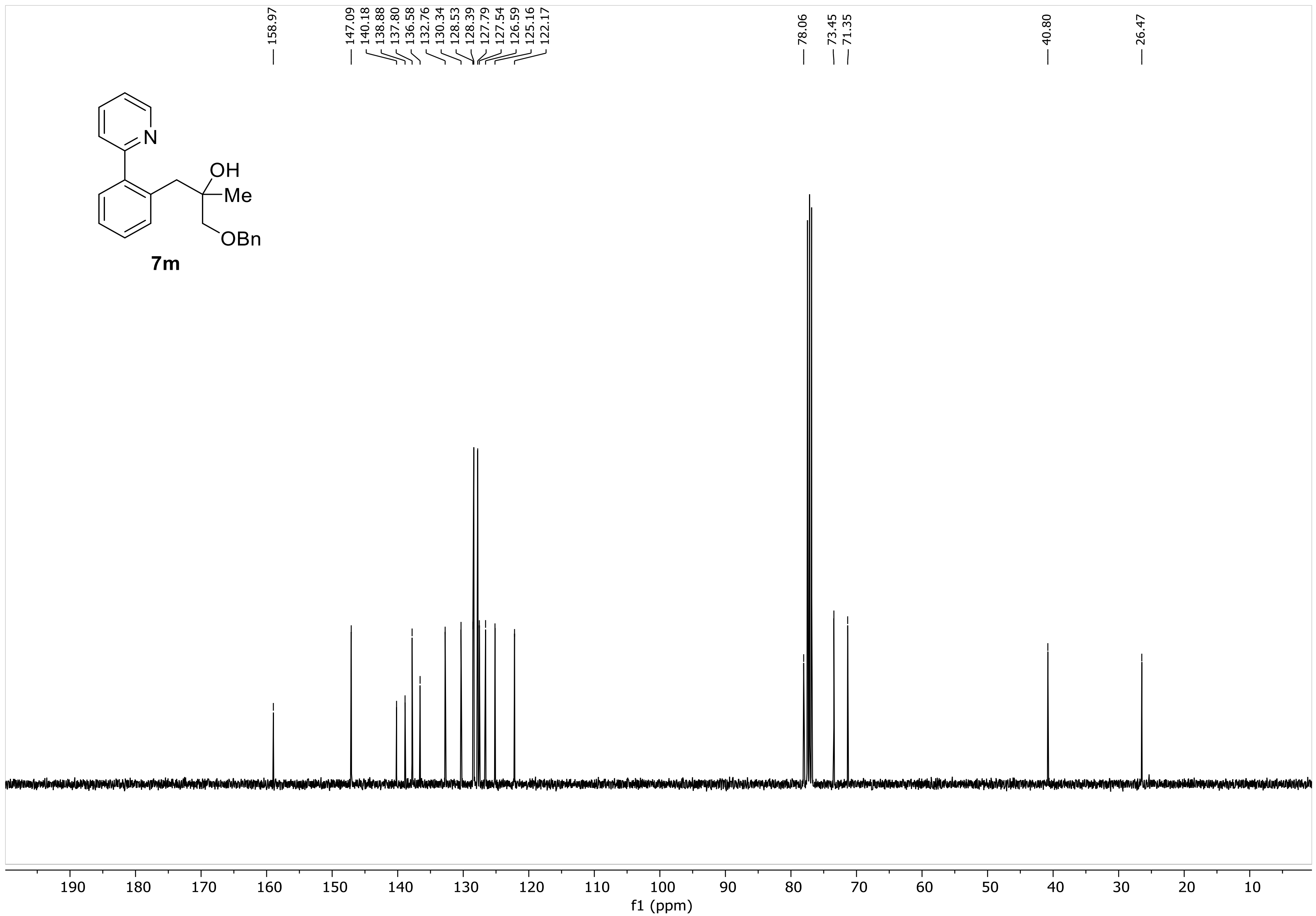

^{1}H NMR spectra (400 MHz, $CDCl_3$) of 1-(benzyloxy)-3-(3,6-difluoro-2-(5-methoxypyridin-2-yl)phenyl)-2-methylpropan-2-ol (**7n**)

10.0. References

(1) Bramham, J. E.; Golovanov, A. Sample illumination device facilitates in situ light-coupled NMR spectroscopy without fibre optics. *Commun. Chem.* **2022**, 5, 90.

(2) Zhuo, C. X.; Zheng, C.; You, S. L. Transition-Metal-Catalyzed Asymmetric Allylic Dearomatization Reactions. *Acc. Chem. Res.* **2014**, *47*, 2558-2573.

(3) Choi, G. J.; Zhu, Q.; Miller, D. C.; Gu, C. J.; Knowles, R. R. Catalytic alkylation of remote C-H bonds enabled by proton-coupled electron transfer. *Nature* **2016**, *539*, 268-271.

(4) Zheng, J.; Swords, W. B.; Jung, H.; Skubi, K. L.; Kidd, J. B.; Meyer, G. J.; Baik, M.-H.; Yoon, T. P. Enantioselective Intermolecular Excited-State Photoreactions Using a Chiral Ir Triplet Sensitizer: Separating Association from Energy Transfer in Asymmetric Photocatalysis. *J. Am. Chem. Soc.* **2019**, *141*, 13625-13634.

(5) Sanderson, R. *Chemical Bonds and Bond Energy*; Academic Press Inc., 1976.

(6) Frisch, M. J.; Trucks, G. W.; Schlegel, H. B.; Scuseria, G. E.; Robb, M. A.; Cheeseman, J. R.; Scalmani, G.; Barone, V.; Petersson, G. A.; Nakatsuji, H.; Li, X.; Caricato, M.; Marenich, A. V.; Bloino, J.; Janesko, B. G.; Gomperts, R.; Mennucci, B.; Hratchian, H. P.; Ortiz, J. V.; Izmaylov, A. F.; Sonnenberg, J. L.; Williams-Young, D.; Ding, F.; Lipparini, F.; Egidi, F.; Goings, J.; Peng, B.; Petrone, A.; Henderson, T.; Ranasinghe, D.; Zakrzewski, V. G.; Gao, J.; Rega, N.; Zheng, G.; Liang, W.; Hada, M.; Ehara, M.; Toyota, K.; Fukuda, R.; Hasegawa, J.; Ishida, M.; Nakajima, T.; Honda, Y.; Kitao, O.; Nakai, H.; Vreven, T.; Throssell, K.; Montgomery, J. A., Jr.; Peralta, J. E.; Ogliaro, F.; Bearpark, M. J.; Heyd, J. J.; Brothers, E. N.; Kudin, K. N.; Staroverov, V. N.; Keith, T. A.; Kobayashi, R.; Normand, J.; Raghavachari, K.; Rendell, A. P.; Burant, J. C.; Iyengar, S. S.; Tomasi, J.; Cossi, M.; Millam, J. M.; Klene, M.; Adamo, C.; Cammi, R.; Ochterski, J. W.; Martin, R. L.; Morokuma, K.; Farkas, O.; Foresman, J. B.; Fox, D. J. Gaussian 16, Revision A.03, Gaussian, Inc., Wallingford CT, 2016.

(7) Becke, A. D. Density-functional thermochemistry. III. The role of exact exchange. *J. Chem. Phys.* **1993**, *98*, 5648-5652.

(8) Stephens, P. J.; Devlin, F. J.; Chabalowski, C. F.; Frisch, M. J. Ab Initio Calculation of Vibrational Absorption and Circular Dichroism Spectra Using Density Functional Force Fields. *J. Phys. Chem.* **1994**, *98*, 11623-11627.

(9) Krishnan, R.; Binkley, J. S.; Seeger, R.; Pople, J. A. Self consistent molecular-orbital methods basis set for correlated wave functions. *J. Chem. Phys.* **1980**, *72*, 650-654.

(10) Andrae, D.; Häußermann, U.; Dolg, M.; Stoll, H.; Preuß, H. Energy-adjusted ab initio pseudopotentials for the second and third row transition elements: Molecular test for M2 (M=Ag, Au) and MH (M=Ru, Os). *Theo. Chim. Acta,* **1991**, *78*, 247-266.

(11) Martin, J. M. L.; Sundermann, A. Correlation consistent valence basis sets for use with the Stuttgart-Dresden-Bonn relativistic effective core potentials: the atoms Ga-Kr and In-Xe. *J. Phys. Chem.* **2001**, *114*, 3408-3420.

(12) Zhao, Y.; Truhlar, D. G. A new local density functional for main-group thermochemistry, transition metal bonding, thermochemical kinetics, and noncovalent interactions. *J. Chem. Phys.* **2006**, *125*, 194101-194101-194118.
(13) Zedler, L.; Kupfer, S.; Schmidt, H.; Dietzek-Ivanšić, B. Oxidation-state sensitive light-induced dynamics of Ruthenium-4H-Imidazole complexes. *Chem. Eur. J.* **2024**, *30*.
(14) Sheldrick, G. M. Crystal structure refinement with SHELXL. *Acta Cryst.* **2015**, *71*, 3-8.
(15) Dolomanov, O. V.; Bourhis, L. J.; Gildea, R. J.; Howard, J. A. K.; Puschmann, H. OLEX2: a complete structure solution, refinement and analysis program. *J. Appl. Cryst.* **2009**, *42*, 339-341.
(16) Hogg, A. A.; Wheatley, M. M.; Domingo-Legarda, P. P.; Carral-Menoyo, A. A.; Cottam, N. N.; Larrosa, I. I. Ruthenium-Catalyzed Monoselective C–H Methylation and d3-Methylation of Arenes. *JACS Au* **2022**, *2*, 2529-2538.
(17) Li, B.; Roisnel, T.; Darcel, C.; Dixneuf, P. H. Cyclometallation of arylimines and nitrogen-containing heterocycles via room-temperature C–H bond activation with arene ruthenium(ii) acetate complexes. *Dalton Trans.* **2012**, *41*, 10934-10937.
(18) Mahajan, B.; Aand, D.; Singh, A. K. Synthesis of Bi(hetero)aryls via Sequential Oxidation and Decarboxylation of Benzylamines in a Batch/Fully Automated Continuous Flow Process. *Eur. J. Org. Chem.* **2018**, *2018*, 2831-2835.
(19) Caramenti, P.; Nicolai, S.; Waser, J. Indole- and Pyrrole-BX: Bench-Stable Hypervalent Iodine Reagents for Heterocycle Umpolung. *Chem. Eur. J.* **2017**, *23*, 14702-14706.
(20) Xu, C. F.; Shen, Q. L. Palladium-Catalyzed Trifluoromethylthiolation of Aryl C-H Bonds. *Org. Lett.* **2014**, *16*, 2046-2049.
(21) Matthew Wheatley, M. T. F., Rocío López-Rodríguez, Diego M. Cannas, Marco Simonetti, Igor Larrosa,. Ru-catalyzed room-temperature alkylation and late-stage alkylation of arenes with primary alkyl bromides. *Chem Cat.* **2021**, *1*, 691-703.
(22) Gharpure, S. J.; Reddy, S. R. B. Tandem SN2-Michael addition to vinylogous carbonates for the stereoselective construction of 2,3,3,5-tetrasubstituted tetrahydrofurans. *Tet. Lett.* **2010**, *51*, 6093-6097.
(23) Kim, J.; Coric, I.; Vellalath, S.; List, B. The Catalytic Asymmetric Acetalization. *Angew. Chem.* **2013**, *52* (16), 4474-4477.
(24) Matsumoto, C.; Yamada, M.; Dong, X.; Mukai, C.; Inagaki, F. The Gold-catalyzed Formal Hydration, Decarboxylation, and [4+2] Cycloaddition of Alkyne Derivatives Featuring Z-type Diphosphinoborane Ligands. *Chem. Lett.* **2018**, *47*, 1321-1323.
